# Physiological, anatomical and transcriptional alterations in a rice mutant leading to enhanced water stress tolerance

**DOI:** 10.1093/aobpla/plv023

**Published:** 2015-03-27

**Authors:** John Milton Lima, Manoj Nath, Prasad Dokku, K. V. Raman, K. P. Kulkarni, C. Vishwakarma, S. P. Sahoo, U. B. Mohapatra, S. V. Amitha Mithra, V. Chinnusamy, S. Robin, N. Sarla, M. Seshashayee, K. Singh, A. K. Singh, N. K. Singh, R. P. Sharma, T. Mohapatra

**Affiliations:** 1 National Research Centre on Plant Biotechnology, IARI, New Delhi, India; 2 Department of Botany, North Orissa University, Baripada, Odisha, India; 3 Indian Agricultural Research Institute, New Delhi, India; 4 Tamil Nadu Agricultural University, Coimbatore, India; 5 Directorate of Rice Research, Hyderabad, India; 6 University of Agricultural Sciences, Bangalore, India; 7 Punjab Agricultural University, Ludhiana, India; 8 Present address: Central Rice Research Institute, Cuttack, Odisha, India

**Keywords:** Differential gene expression, drought tolerance, EMS-induced mutant, germination, grain chalkiness, root traits, SSR genotyping, stomata

## Abstract

Water stress is one of the most severe constraints to crop productivity. Plants display a variety of physiological and biochemical responses both at the cellular and whole organism level upon sensing water stress. Leaf rolling, stomatal closure, deeper root penetration, higher relative water content (RWC) and better osmotic adjustment are some of the mechanisms that plants employ to overcome water stress. In the current study, we report a mutant, *enhanced water stress tolerant1* (*ewst1*) with enhanced water stress tolerance, identified from the ethyl methanesulfonate-induced mutant population of rice variety Nagina22 by field screening followed by withdrawal of irrigation in pots and hydroponics (PEG 6000). Though *ewst1* was morphologically similar to the wild type (WT) for 35 of the 38 morphological descriptors (except chalky endosperm/expression of white core, decorticated grain colour and grain weight), it showed enhanced germination in polyethylene glycol-infused medium. It exhibited increase in maximum root length without any significant changes in its root weight, root volume and total root number on crown when compared with the WT under stress in PVC tube experiment. It also showed better performance for various physiological parameters such as RWC, cell membrane stability and chlorophyll concentration upon water stress in a pot experiment. Root anatomy and stomatal microscopic studies revealed changes in the number of xylem and phloem cells, size of central meta-xylem and number of closed stomata in *ewst1*. Comparative genome-wide transcriptome analysis identified genes related to exocytosis, secondary metabolites, tryptophan biosynthesis, protein phosphorylation and other signalling pathways to be playing a role in enhanced response to water stress in *ewst1*. The possible involvement of a candidate gene with respect to the observed morpho-physiological and transcriptional changes and its role in stress tolerance are discussed. The mutant identified and characterized in this study will be useful for further dissection of water stress tolerance in rice.

## Abstract (precis)

Water stress is a serious challenge to rice production. Understanding water stress tolerance is essential for precise trait modification. We identified an EMS induced mutant showing enhanced tolerance to water deficit stress at the vegetative stage. Multiple alterations in physiological behaviour, root morphological and anatomical structure, stomatal response and gene expression in various signalling pathways were found to be responsible for increased tolerance in the mutant. The mutant will be useful for dissecting the water stress tolerance mechanism in rice.

## Introduction

Drought or water stress is considered the single most critical threat to food production and hence to food security. Water stress causes severe damage to plant cells, which ultimately affects the growth, development and productivity of a plant. Plants respond to such external stimuli with a series of morphological, physiochemical, cellular and molecular adjustments so as to adapt to the stress environment. Various adaptive mechanisms such as better root architecture, higher leaf water potential, better osmotic adjustment or protective mechanisms such as leaf rolling and stomatal closure have been reported to be associated with water stress tolerance in various crop plants (Tuberosa 2012). Rice is the staple food crop for more than half of the world population. This crop uses ∼2500 L of water throughout its life period to produce 1 kg of rice. It frequently encounters water stress at different stages of its life cycle, viz. germination, seedling, tillering, flowering and grain filling, resulting in huge loss of productivity (Yue *et al.* 2006; Serraj *et al.* 2009). Water stress at the vegetative stage leads to leaf senescence, reduction in photosynthesis, suppression of leaf expansion and tillering, stunted growth and low yield (Bunnag and Pongthai 2013; Rebolledo *et al*. 2013). Identification of genotypes with higher survival rate at vegetative stages can help to overcome yield reduction caused by stunted plant growth. Moreover, such genotypes can serve as a resource material to develop physiological and molecular insights into tolerance mechanisms under water stress. It is well demonstrated that such tolerance mechanisms show multigenic inheritance and hence a greater understanding of the molecular regulation that brings about the differences in water stress tolerance would tremendously help in engineering rice cultivars with superior adaptation to water stress. Rice genotypes with better stress tolerance have been identified through *in vitro* approaches using polyethylene glycol (PEG) in an appropriate nutrient medium (Huang *et al*. 2009; Chutia and Borah 2012).

Use of induced mutants is a potential approach to identify genes affecting trait variation and to understand the underlying molecular mechanisms in plants (Sikora *et al.* 2011), since the mutants and wild type (WT) share more or less the same genetic background and hence can be treated as isogenic or near isogenic lines. Chemically induced mutants have been extensively used for identifying gene(s) involved in various agronomically important traits including water stress tolerance in crop plants including rice (Wu *et al.* 2005; Till *et al.* 2007; Sikora *et al.* 2011). One of the most frequently used mutagenic agent is ethyl methanesulfonate (EMS), which induces chemical modification of nucleotides resulting in various point mutations in different genomic regions (Till *et al.* 2007), the most common being GC to AT transitions. EMS-induced mutants such as drought and salt tolerant (*dst*) and rice salt sensitive2 (*rss2*) have been successfully used to explore the complex mechanism behind water and salt-stress tolerance, respectively, in rice (Huang *et al.* 2009; Zhou *et al.* 2013). Furthermore, morphological, physiological and proteomic characterization of some EMS-induced mutants showing altered response when compared with their respective WT under stress has given valuable information about plant adaptation mechanisms (Nakhoda *et al*. 2012; Ghaffari *et al.* 2014). In India, more than 20 000 stable EMS-induced mutant lines had been generated in the background of upland rice variety Nagina22 in a multi-institutional effort (Mohapatra *et al*. 2014) as a national resource for functional genomic studies in rice. This mutant resource has been used for identification and characterization of mutants for abiotic and yield-related traits (Poli *et al*. 2013; Kulkarni *et al*. 2014). Such a resource has the advantages of being grown, multiplied and screened for various traits of interest without any restriction unlike transposon/T-DNA insertional or genetically engineered mutants.

To explore the complex water stress tolerance mechanism, approaches like genetic mapping, genome-wide association mapping, whole-genome expression analysis using microarray and transcriptome sequencing have been employed in recent decades. Transcriptome profiling using microarray has enabled genome-wide discovery of differentially expressed stress-responsive genes, which give ample information about the changes in cellular, biological and metabolic pathways that occur in water-stressed plants (Galbraith and Edwards 2010). A number of studies have revealed a network of genes responsible for water stress tolerance in different tissues (Wang *et al.* 2011) and contrasting genotypes of rice (Wang *et al.* 2007; Lenka *et al.* 2011). Use of isogenic or near isogenic lines with a variation in the trait of interest for transcriptome profiling might provide trait-specific and more relevant information when compared with using contrasting genotypes with completely different genetic backgrounds (Moumeni *et al*. 2011).

In the above context, we made an effort to identify EMS-induced mutant(s), which have higher tolerance to PEG-induced water stress and soil-water stress at the vegetative stage than the WT, upland rice variety Nagina22. We report here one such mutant characterized for morphological, physiological, cytological and transcriptional changes when compared with its WT, which would provide possible clues to water stress tolerance in rice.

## Methods

### Plant materials

Nagina22 is an upland rice variety known for its tolerance to drought stress and is an international standard in drought breeding programmes and studies on dissection of drought tolerance quantitative trait loci/genes in rice. A set of 1100 M6 EMS mutagenized lines of Nagina22 (referred to as WT throughout the article) were randomly chosen from the national mutant resource (Mohapatra *et al.* 2014) and subjected to drought screening at the research field of Central Rice Research Institute, Cuttack, India in *Rabi* 2010. Thirty-day-old plants were subjected to water stress by withholding irrigation for 10 days followed by watering for recovery. The mutants were scored on a 0–9 scale based on their leaf death or response to water stress, following the standard evaluation system (SES) for rice, developed and adopted by International Rice Research Institute (IRRI) (www.knowledgebank.irri.org). In this study, a total of 500 mutants showing a 0–5 (highly tolerant to moderately susceptible) drought score were selected for further screening under water stress (data not shown).

### Growth conditions and stress treatment

#### Polyethylene glycol screening

Seeds from the 500 selected mutant lines, identified by field screening done at CRRI, and WT were germinated in a magenta box on a blotting sheet, under well-watered conditions, in a dark chamber for 48 h. Seven-day-old seedlings (10 from each line) were transferred to plastic trays containing Hoagland solution. In a thermocol sheet (packing material) equal to the size (upper rim) of the plastic tray, holes were made at 10 × 15 cm intervals and seedlings were placed with a cotton plug. This sheet was placed on the plastic tray containing a hydroponic set-up and grown for 14 days in the National Phytotron Facility, Indian Agricultural Research Institute (IARI), New Delhi. This experiment was carried out under controlled growth conditions at 25 ± 2 °C and 90 % relative humidity for 24 h in a dark and then shifted to a 16/8 h light/dark regime.

Response of Nagina22 to moisture-deficit stress was optimized under three different concentrations (20, 25 and 30 %) of polyethylene glycol (PEG; molecular weight, 6000) in the nutrient medium. After optimization, stress was imposed on 21-day-old mutant seedlings using 25 % (w/v) PEG6000 for 6 days. The PEG solution was changed every alternate day to maintain pH and uniform stress conditions throughout the experiment. The plants were scored for response to PEG-induced drought stress based on a 0–9 scale scoring pattern of SES, IRRI from the third to the sixth day. Mutant lines with a score ≤5 were considered tolerant.

#### Pot screening

To screen for tolerance to soil-water stress, the same set of mutants were grown in pots under well-watered conditions in a rain-sheltered net-house of National Research Centre on Plant Biotechnology (NRCPB), IARI, New Delhi for 2 consecutive years in *Kharif* 2011 and 2012. Mutants were grown in 6-inch pots in three replications under well-watered conditions. Twenty-one-day-old seedlings (50 in each pot) of mutants and WT were exposed to water stress, by withholding water supply for 6 days followed by 4 days of a recovery period. Drought scoring was done on the basis of leaf rolling following the SES of IRRI. The mutants having a higher level of tolerance than the WT were identified. Only those mutants that performed better than WT in both the experiments (PEG and pot) were shortlisted as water stress-tolerant mutants.

### Confirmation of mutant background being true to the WT

#### Distinctness, uniformity and stability characterization

One mutant identified as tolerant under both PEG and pot-screening experiments for enhanced tolerance to water stress, named as ‘enhanced water stress tolerant1’ (*ewst1*), and the WT were grown in three rows each in the research field of IARI, New Delhi at a spacing of 20 cm between rows and 15 cm between plants within a row following recommended agronomic practices. Data on plant height (PH), panicle length (PL), flag leaf length (FL), flag leaf width, number of panicles (NPs) and seed morphology were recorded at the stage of maturity. Distinctness, uniformity and stability (DUS) were noted down at appropriate stages of growth for the mutant and the WT.

#### Characterization using microsatellite markers

For genotyping with microsatellite markers, fresh leaf tissues from five random plants of 1-month-old seedlings of *ewst1* and the WT (field grown) were collected and stored. Within genotypes, the samples were pooled, and DNA was extracted using CTAB, according to the modified protocol of Doyle and Doyle (1990), and quantified using Nanodrop (Thermo Scientific, USA). Polymerase chain reaction was performed according to the standard protocol used by Parida *et al.* (2006). The amplification products were separated on 4 % metaphor agarose gels and photographed using a gel documentation system. A set of 72 rice microsatellite markers **[see Supporting Information—Table S1]** including 36 markers recently used by Tiwari *et al.* (2015), 6 from each rice chromosome, were used for genotyping.

#### Evaluation of stress tolerance in ewst1

For all the following experiments both the genotypes (*ewst1* and WT) were grown under appropriate water stress and control (proper irrigation) conditions in three replications. Any deviation from this is mentioned under the respective experiment.

#### Recovery study in pot experiment

The recovery rate of *ewst1* and WT was calculated from the pot-screening experiment. The percentage of water stress recovery was recorded by calculating the number of revived plants after stress upon the total number of plants (50).

#### Germination study under stress

A germination test was conducted on PEG-infused MS agar plates following the protocol of Verslues *et al.* (2006) with slight modification. Polyethylene glycol-infused MS agar plates (100 × 20 mm round) were prepared by overnight infusion with PEG (molecular weight 8000) overlay at three different concentrations i.e. 25, 40 and 55 %, which create osmotic potentials of −0.5, −0.7 and −1.2 MPa, respectively, and the overlay solution was discarded 14 h after infusion. Healthy dehusked seeds of *ewst1* and WT were surface-sterilized with 70 % ethanol for 2 min followed by 0.1 % mercuric chloride for 10 min and thoroughly rinsed five times in sterile distilled water. The sterilized seeds were blot dried with a sterile wattman no. 1 filter paper and aseptically cultured on PEG-infused media. A set of three replicates comprising 30 seeds each (*ewst1* and WT) were germinated for every treatment of PEG-infused MS agar plates along with control (MS plate without PEG infusion). The plates were made airtight by sealing with parafilm so as to maintain the osmotic potential and stored in a dark chamber at a temperature of 28 ± 2 °C. The percentage of seed germination was recorded on the sixth day.

### Morphological characterization

#### Root phenotyping

To carry out phenotyping for root characters under stress, 21-day-old seedlings of *ewst1* and WT were transplanted in 1.5 m PVC tubes (one healthy plant in each tube) filled with a mixture of sandy clay loam soil and FYM in a 1 : 4 proportion in the rain-sheltered net-house of NRCPB, IARI, New Delhi. The bottom of the tubes was covered with plastic sheet to avoid seepage. Water stress was imposed by withholding water supply for 15 days at the active tillering stage (45-day-old plants). At maturity, soil was removed from the pipes slowly by applying water and roots were collected carefully. Data on maximum root length (MRL), root weight (RW), root volume (RV) and total number of roots on the crown (RN) were recorded under control and stress conditions from three random samples in each replication. The relative effects of stress on these root traits were calculated by using the following formula:

$$\text{Relative}\;\text{root}\;\text{trait}=\frac{\text{root}\;\text{trait}\;\text{in}\;\text{water}\;\text{stress}}{\text{root}\;\text{trait}\;\text{in control}}$$

### Physiological characterization

To study the physiological characters of *ewst1* and Nagina22, 21-day-old seedlings were transplanted in three replications in 6-inch pots under well-watered conditions in a rain-shaded net-house of NRCPB, IARI, New Delhi. Water stress was imposed on 45-day-old plants for 7 days. Leaf samples were collected from three plants per replication both from control and stress treatment. Physiological characters related to water stress tolerance such as relative water content (RWC), total chlorophyll content and cell membrane stability (CMS) were measured. The RWC of rice leaves was measured as given by Barr and Weatherley (1962) using the following formula:

$$\text{RWC}=\frac{\text{Fresh}\;\text{weight}-\text{Dry}\;\text{weight}}{\text{Turgid}\;\text{weight}-\text{Dry}\;\text{weight}}\times 100$$

To understand resistance of *ewst1* to membrane injury during stress, measurement of CMS was carried out following the protocol of Blum and Ebercon (1981) and Tripathy *et al*. (2000). The leaves of plants from control and stress conditions were collected at more than 90 % and 60–65 % of RWC, respectively, and washed five times with deionized water. Then the samples were chopped into segments, washed once with deionized water and kept in a capped vial with 10 mL of deionized water for 24 h at room temperature followed by 20 min in an autoclave. Electrolytic conductance was measured using a conductivity meter both before autoclaving and after cooling of the autoclaved samples. Cell membrane stability was calculated as the reciprocal of cell membrane injury after stress according to the formula CMS% = [(1 − (*T*_1_/*T*_2_))/(1 − (*C*_1_/*C*_2_))] × 100, where *T* and *C* refer to the stressed and control samples, respectively; the subscripts 1 and 2 refer to the initial and final conductance readings, respectively. Chlorophyll was extracted from 0.2 g fresh leaves of samples with dimethyl sulfoxide, and the chlorophyll content was determined by spectrophotometry according to the method of Hiscox and Israelstam (1979).

### Stomata and root anatomy studies

Leaves of 52-day-old plants (45 + 7 days of water stress) of the mutant and the WT grown for physiological characterization were detached (three samples per replication) and immediately fixed in liquid nitrogen. The stomatal analysis was done in an environmental scanning electron microscope (Zeiss EVO MA10) available in the scanning electron microscope facility, IARI, New Delhi at controlled temperature (−4 °C). All images were captured in an identical setting such as 20 µm bar and 6-mm working distance and 20 kV extra high tension with three technical replications per sample. On the basis of the opening of the guard cell, stomata were categorized as completely open (CO), partially open (PO) and completely closed (CC).

The basal region (elongation area) of the crown root of WT and *ewst1* were collected from the irrigated pots of 45-day-old plants. In WT, sections were cut at 1, 5 and 7 cm from the tip of the crown root and three sections of 10 µm length were dissected and their images analysed. Since there was no difference among the images of different root length sections **[see Supporting Information****—Fig. S1****]**, for a comparative root anatomical analysis of WT and *ewst1*, 10 µm root sections were obtained using Ultra-microtome (Leica EM UC7) at 1 cm from the root tip in three replicates. The sections were processed for histochemical analysis following the method of Jensen (1962) and further the sections were stained with 0.1 % safranine O. Root section images were captured using a digital camera connected with an optical microscope (Zeiss Axioplan, Zeiss, Oberkochen, Germany). Root sections were visualized using the microscope under 20× magnification (data not shown), while the vascular bundles (stellar region) were observed at 70× magnification. The root parameters, i.e. shape of central meta-xylem and the number of xylem and phloem vessels, were visually recorded for comparative analysis.

### Statistical analysis

All the experimental data were subjected to Student's *t*-test (*P* ≤ 0.05) for comparative evaluation of changes in the mutant and the WT both under control and stress conditions using graphpad prism 6.0 statistical software (www.graphpad.com).

### Sample preparation, total RNA isolation and genome-wide transcriptome profiling

Twenty-one-day-old seedlings of the mutant and WT grown in hydroponic culture in three replications were subjected to 25 % PEG stress for 1 h. The leaf samples from stressed and control condition seedlings were collected and preserved in liquid nitrogen for RNA isolation. Total RNA from four samples, i.e. mutant control (MC), mutant stress (MS), Nagina22 control (NC) and Nagina22 stress (NS), was extracted by following the manufacturer's instructions provided with the SV Total RNA isolation Kit (PROMEGA, USA). All the steps starting from cRNA preparation to hybridization were conducted following the instructions of Affymetrix (AffymetrixGeneChip Expression Analysis Technical Manual). Chips were washed and stained in the Affymetrix Fluidics Station 450, and then scanned using the Affymetrix Gene Chip Scanner 3000. The cell intensity data files (.CEL) generated by the Gene Chip Operating Software (GCOS 1.2) were imported to GeneSpring Software (Schadt *et al.* 2001). The CEL files are deposited in the array express repository (accession idE-MTAB-3230 in https://www.ebi.ac.uk/arrayexpress/). Normalization of all arrays was carried out using a robust multiarray analysis (RMA) algorithm with input parameters of Post Hoc-Tukey HSD, 1000 permutative *P* value and Benjamini–Hoschberg false discovery rate correction. One-way analysis of variance was performed with a cut-off value of ≥2-fold change and *P* value threshold of <0.05. Differential gene expression was assumed if values above the threshold were obtained in at least any one out of six combinations (MS-MC, MC-NC, NS-MC, MS-NC, MS-NS, NS-NC) examined. The sample- and condition-specific differentially expressed genes (DEGs) were selected through union and intersection of DEGs using a multi-way Venn diagram.

In addition, analysis was also done in Java-based graphical wizard application ROBIN (Lohse *et al*. 2010) using a GCRMA algorithm. The normalized log-transformed intensity values of selected DEGs were used for heatmap by applying average linkage and Euclidean distance matrix as a measurement of similarity test in ggplot2 package of R (2.15.1). All the required affymetrix probe sets and their expression were exported to MS-excel and analysis was performed manually. All the probe sets were converted to TIGR MSU Locus IDs in Oligonucleotide rice array database (www.rad.org) and RiceChip database (www.ricechip.com). The Locus IDs were functionally annotated on TIGR rice pseudomolecules, release 7.0 (www.tigr.org).

### Functional classification, GO annotation and pathway analysis

Each set of DEGs obtained was functionally categorized on the basis of their biological, molecular and cellular functions by analysing them in terms of their enriched Gene Ontology function using GOEAST, a web-based software analysis tool (Zheng and Wang 2008; http://omicslab.genetics.ac.cn/GOEAST/). The GO slim categories significantly overrepresented were calculated by a hyper geometric distribution with a cut-off level of *P* value at 0.05. Pathway analyses of selected DEGs were conducted using online RiceCyc (www.gramene.org/pathway/) and MAPMAN software (Thimm *et al.* 2004).

To identify transcription factors (TFs), the MSU locus IDs of all selected DEGs were analysed using Database of Rice Transcription Factors (DRTF), GRASSIUS and RiceFREND (Gao *et al*. 2006; Yilmaz *et al.* 2009; Sato *et al.* 2013). Enrichment analysis of cis-regulatory elements of the promoter regions was done in an online promoter database of rice named Osiris developed by Morris *et al.* (2008).

### Validation of DEGs

The same RNA samples that were used in the microarray study were used for first-strand cDNA synthesis preparation using the ImProm-II reverse transcription system (Promega) following the manufacturer's instructions. Amplification reactions were carried out on samples containing an aliquot of cDNA synthesized from 100 ng of total cDNA, 1× taq buffer, 1.5 mM MgCl_2_, 200 mM each dATP, dCTP, dGTP and dTTP, 5 pmol of each primer and 1 unit of *Taq* DNA polymerase (Merck, USA) in a final volume of 10 µL. Thermal cycling conditions comprised an initial denaturation at 95 °C for 30 s, 30 cycles of denaturation at 95 °C for 30 s, annealing at 55–60 °C for 30 s and extension at 72 °C for 1 min in a thermal cycler (Eppendorf, Germany). Rice actin gene was used as the endogenous control and the normalized cDNA of all samples were used to validate microarray data with selected uniquely regulated differentially expressed genes (URDEGs). Nucleotide sequences of differentially regulated genes were downloaded from the TIGR rice database (http://rice.plantbiology.msu.edu). Exonic sequences of selected genes were used for primer design using the primer synthesizing tool of IDT SciTools (http://eu.idtdna.com/site). The parameters kept for primer design were: optimum GC content of 50 %, Tm >55–65 °C, length of 18–25 nucleotides and an expected amplicon size of 100–150 bp. All the primers were synthesized from Sigma (Sigma-Aldrich, USA). Polymerase chain reaction products were fractionated on 1.5 % (w/v) agarose gels.

## Results

### Optimization of PEG concentration for screening of mutants

In the PEG experiment, out of the three different concentrations tried, 25 % was the concentration at which the WT Nagina22 started showing leaf rolling within 1 h of stress, and within 48 h of stress, the WT plants dried up completely. In 20 % PEG solution, the WT did not show any sign of stress. However, in 30 % PEG solution, the WT did not survive (data not shown). Therefore, 25 % PEG concentration was considered as an optimum concentration for screening the mutants.

### Identification of water stress-tolerant mutants from PEG and pot-screening studies

**Figure 1.**
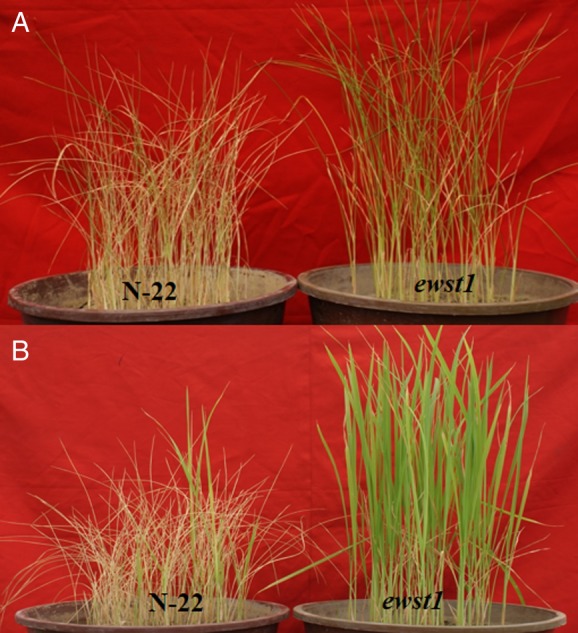
Identification of a gain-of-function mutant under PEG-induced water stress and soil-water stress. (A) Twenty-one-day-old seedlings of the selected mutant screened in pots by withholding irrigation for 6 days. (B) The extent of recovery of the mutant after 4 days of the recovery period.

We identified a mutant, *ewst1* that showed enhanced tolerance with a 90–100 % survival rate when compared with its WT Nagina22 under PEG (6000) stress **[see Supporting Information****—Fig. S2****A and B]**. This mutant exhibited enhanced tolerance in pot screening carried out under soil-water stress than the WT in terms of percentage of leaf rolling, leaf drying and recovery rate. In the drought scoring scale, *ewst1* had a low score (score 0), indicating better tolerance, whereas the WT was susceptible with a higher score (score 7) under stress (Fig. 1A). It also recovered better with a recovery rate of 92.6 % when compared with Nagina22 (15.3 %) (Fig. 1B).

### Higher germination of *ewst1* under PEG stress

**Figure 2.**
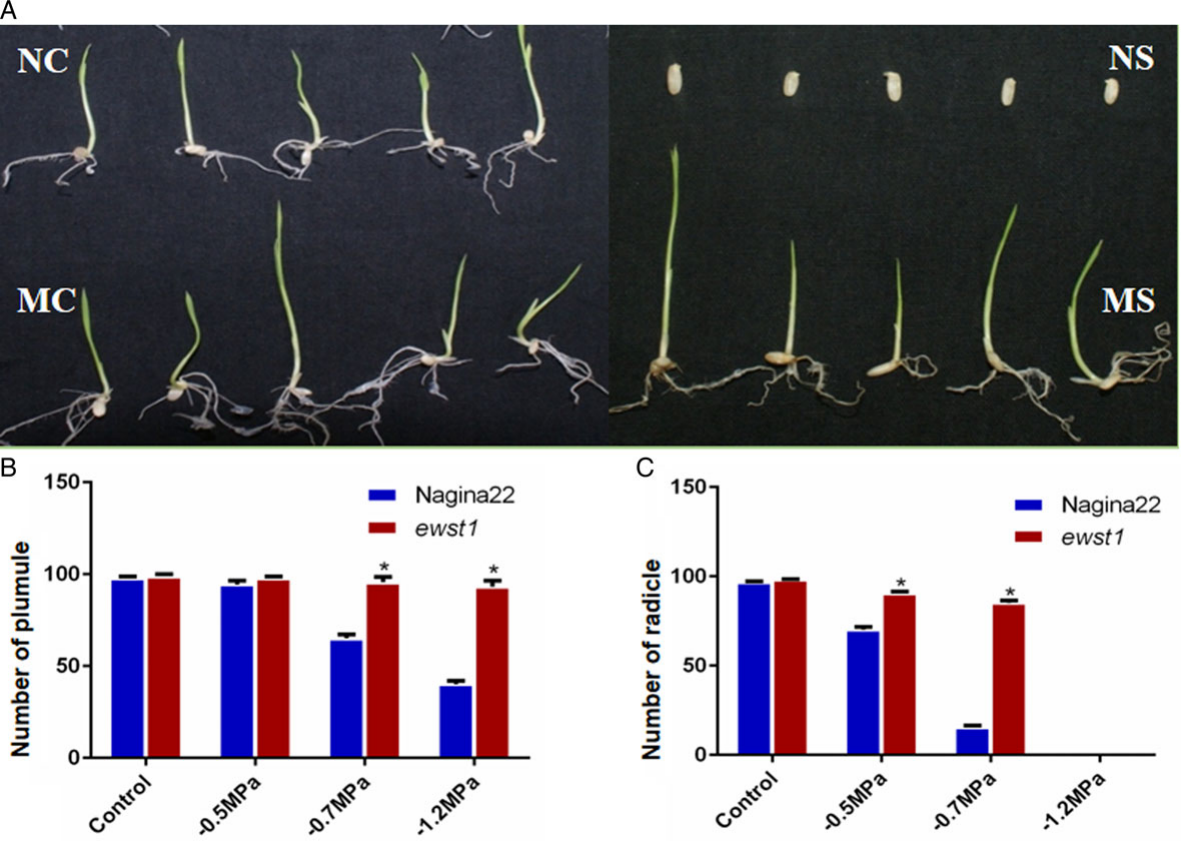
Germination of *ewst1* when compared with its WT Nagina22 (NC, Nagina22 control; NS, Nagina22 stress; MC, mutant control; MS, mutant stress). (A) Germination of *ewst1* and Nagina22 in PEG-infused media under control and −0.7 MPa osmotic stress conditions. (B) Comparison of plumule development between WT and *ewst1* under three different osmotic levels. (C) Comparison of radicle development between WT and *ewst1* under three different osmotic levels. (B and C) Values are mean ± SE of three individual replications having 30 seeds in each plate. Statistical significance was determined using the Holm–Sidak method, with α = 5.000 %. Asterisks indicate significant differences between WT and *ewst1* (Student's *t*-test *P* < 0.05).

The mutant showed significant difference in radicle (*ewst1* = 85.1 % and WT = 15.5 %) and plumule (*ewst1* =65.1 % and WT = 95.5 %) growth at −0.7 MPa when compared with the WT, whereas there was no difference in control plates. At an osmotic potential of −0.5 MPa, there was a significant difference in radicle growth, whereas at −1.2 MPa plumule growth showed a significant difference. These results indicated that the *ewst1* possessed greater germination percentage, and plumule and radicle development when compared with the WT under the water stress conditions (Fig. 2A–C).

### Morphological, physiological and anatomical alterations in the mutant

**Figure 3.**
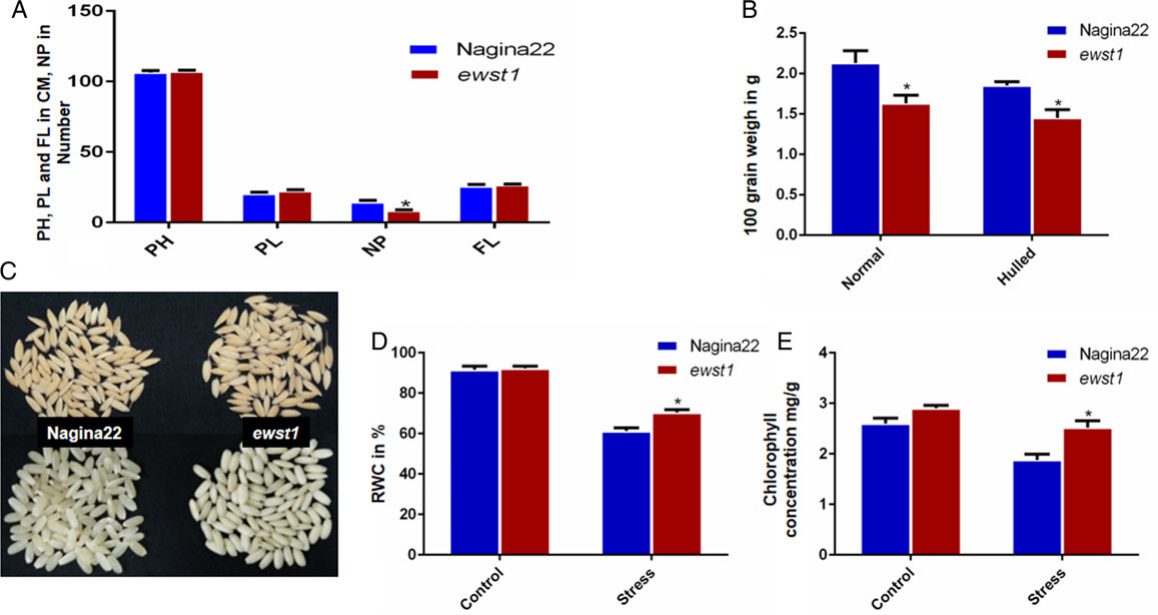
Morpho-physiological changes in the mutant when compared with Nagina22. (A) Comparison of agronomic traits, namely plant height (PH), PL, NP and FL in centimetres. (B) Comparison of 100 grain weight of unhulled and hulled grain. (C) Grain morphology of Nagina22 (left) and mutant (right) showing 100 % grain chalkiness only in the *ewst1*. (D) Percentage of RWC under control and stress conditions. (E) Total chlorophyll concentration under control and stress conditions. Values are mean ± SE of five individual replications for (A) and three individual replications for (B–D). Statistical significance determined using the Holm–Sidak method, with α = 5.0 %. Asterisks indicate significant differences between Nagina22 and the mutant (Student's *t*-test: *P* < 0.05).

The identified mutant, *ewst1,* was found to be morphologically very similar to the parent variety Nagina22. Though there were no significant changes in the values of agronomic traits, namely PH, PL and FL, *ewst1* had significantly fewer panicles when compared with the WT (Fig. 3A). For most of the DUS characters (35/38) *ewst1* was found to be identical to the WT except for decorticated grain colour, grain chalkiness and grain weight (Table 1). Lower grain weight (Fig. 3B) and complete grain chalkiness (100 %) were observed in *ewst1* in contrast to the translucent nature of WT grains (Fig. 3C). The single sequence repeat (SSR) genotyping carried out employing 72 rice microsatellite markers revealed identical fingerprinting patterns confirming a high degree of genetic similarity between *ewst1* and the WT genomes.

**Table 1.** DUS (distinctness, uniformity and stability) characters of Nagina22 and *ewst1* mutant. The DUS parameters in which the mutant differed from the WT are shown in bold font.

S. no.	Characters	Nagina22	*ewst1*
1	Basal Leaf: sheath colour	Light purple	Light purple
2	Leaf: intensity of green colour	Medium	Medium
3	Leaf: anthocyanin colouration	Absent	Absent
4	Leaf: auricles	Present	Present
5	Leaf: anthocyanin colouration of auricles	Colourless	Colourless
6	Leaf: collar	Present	Present
7	Leaf: anthocyanin colouration of collar	Absent	Absent
8	Leaf: ligule	Present	Present
9	Leaf: shape of ligule	Split	Split
10	Leaf: colour of ligule	Light purple	Light purple
11	Leaf: length of blade	Medium	Medium
12	Leaf: width of blade	Medium	Medium
13	Culm: attitude	Semi-erect	Semi-erect
14	Time of heading (50 % of plants with panicles)	Medium	Medium
15	Flag leaf: attitude of blade (early observation)	Semi-erect	Semi-erect
16	Male sterility	Absent	Absent
17	Lemma: anthocyanin colouration of keel	Absent	Absent
18	Lemma: anthocyanin colouration of area below apex	Absent	Absent
19	Lemma: anthocyanin colouration of apex	Strong	Strong
20	Spikelet: colour of stigma	White	White
21	Stem: thickness	Medium	Medium
22	Stem: length (excluding panicle)	Medium	Medium
23	Stem: anthocyanin colouration of nodes	Absent	Absent
24	Panicle: length of the main axis	Medium	Medium
25	Flag leaf: attitude of blade (late observation)	Semi-erect	Semi-erect
26	Panicle: curvature of the main axis	Straight	Straight
27	Panicle: number per plant	Medium	Medium
28	Spikelet : colour of tip of lemma	Purple	Purple
29	Lemma and Palea: colour	Straw	Straw
30	Panicle : awns	Absent	Absent
31	Panicle: presence of secondary branching	Present	Present
32	Panicle: secondary branching	Weak	Weak
33	Panicle: attitude of branches	Semi-erect	Semi-erect
34	Panicle: exertion	Exerted	Exerted
35	Time of maturity	Medium	Medium
36	Decorticated grain: colour	**Light brown**	**White**
37	Polished grain: expression of white core	**Absent**	**Present (large)**
38	Grain: weight of 100 fully developed grains	**Medium**	**Low**

**Figure 4.**
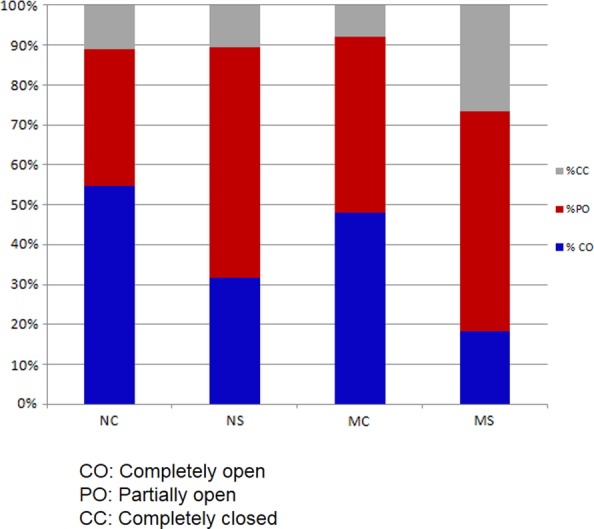
Comparative stomatal behaviour of *ewst1* and WT under control and stress conditions showing CO, PO and CC stomata.

The measurement of various physiological parameters revealed that *ewst1* showed an increased level of RWC, CMS and chlorophyll concentration under water deficit stress over the WT (Fig. 3D and E). Though the RWC of the mutant and WT showed no significant difference under control, upon water stress, the RWC of the mutant was found to be 11 % more than that of WT (Fig. 3D). On the basis of ionic leakage, the CMS of *ewst1* was significantly higher (93.4 ± 4.3) when compared with the WT (78.3 ± 5.4) under stress. Similarly, the total chlorophyll concentration was significantly higher in *ewst1* than the WT under stress without any significant change under control (Fig. 3E). The stomatal movement analyses under ESEM revealed that there were more PO stomata in the mutant under control, but more CC stomata and lesser CO stomata in *ewst1* under stress conditions when compared with the WT (Fig. 4) **[****Supporting Information—Fig. S3****]**.

**Figure 5.**
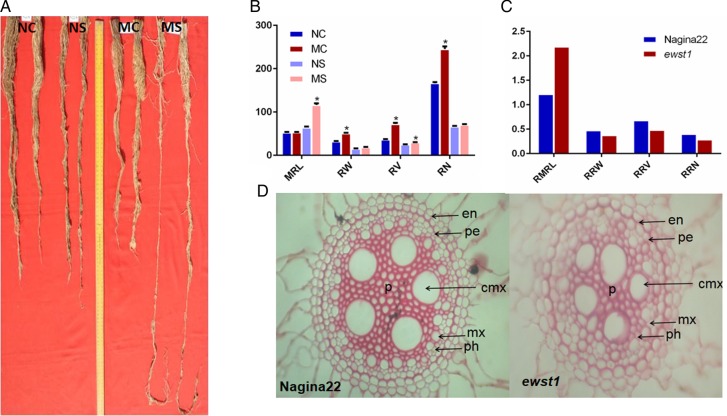
Comparative root study of *ewst1* and the WT. (A) Development of root in a PVC tube under control and stress conditions. (B) Comparison of root traits like MRL, RW, RV and root number (RN) under control and stress conditions. (C) Relative values of maximum root length (RMRL), relative root weight (RRW), relative root volume (RRV) and relative total root number (RRN) under control and stress. (D) Anatomy of root magnifying the stele region (en, endodermis; cb, casparian band; pe, pericycle; cmx, central meta-xylem; mx, meta-xylem; ph, phloem; p, pith). Values are mean ± SE of three individual replications. Statistical significance determined using the Holm–Sidak method, with α = 5.0 %. Asterisks indicate significant differences between *ewst1* and WT (Student's *t*-test: *P* < 0.05).

The root analysis performed in samples taken from plants grown in PVC tubes revealed that there were significant differences in root growth parameters in *ewst1* when compared with WT in response to water stress (Fig. 5A). There was a significant increase in RW, RV and RN of the mutant under well-watered conditions, and MRL and RV of the mutant under stress conditions when compared with its WT. Interestingly, no significant change was found in MRL under control, and RW and RN under stress conditions (Fig. 5B). However, in terms of relative value of root traits under control and stress conditions, the mutant showed a higher value for the relative maximum root length (RMRL) and lesser value for all other root parameters, namely RRW, RRV and relative root number on the crown when compared with the WT (Fig. 5C). Root anatomical studies revealed variations in the size and the number of cells in the stellar region between the mutant and the WT (Fig. 5D). The numbers of central meta-xylem, xylem and phloem cells were 5, 14 and 14 in WT, while these were 5, 9 and 9 in the mutant, respectively. The central meta-xylems were similar in shape and uniformly distributed in the WT, whereas they were of different shapes with reduced diameter and altered arrangement in the mutant.

### Identification and classification of DEGs

**Figure 6.**
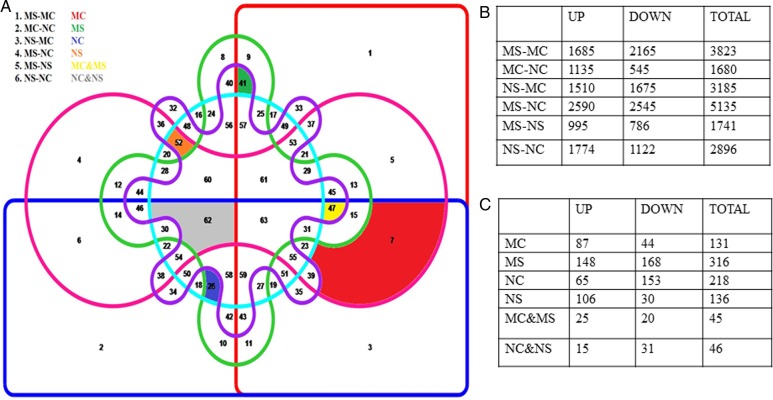
Six-way Venn diagram depicting the number of DEGs in six possible combinations of four samples (NC, Nagina22 control; NS, Nagina22 stress; MC, mutant control; MS, mutant stress). (A) The coloured chambers of the six-way Venn diagram representing uniquely up- and down-regulated differentially expressed genes (URDEGs) out of six combinations (specific colour written for MC, MS, NC, NS, MC and MS and also NC and NS represents specific URDEGs for respective samples). (B) Number of DEGs in six combinations. (C) Number of URDEGs in 12 clusters of similar co-expression.

**Figure 7.**
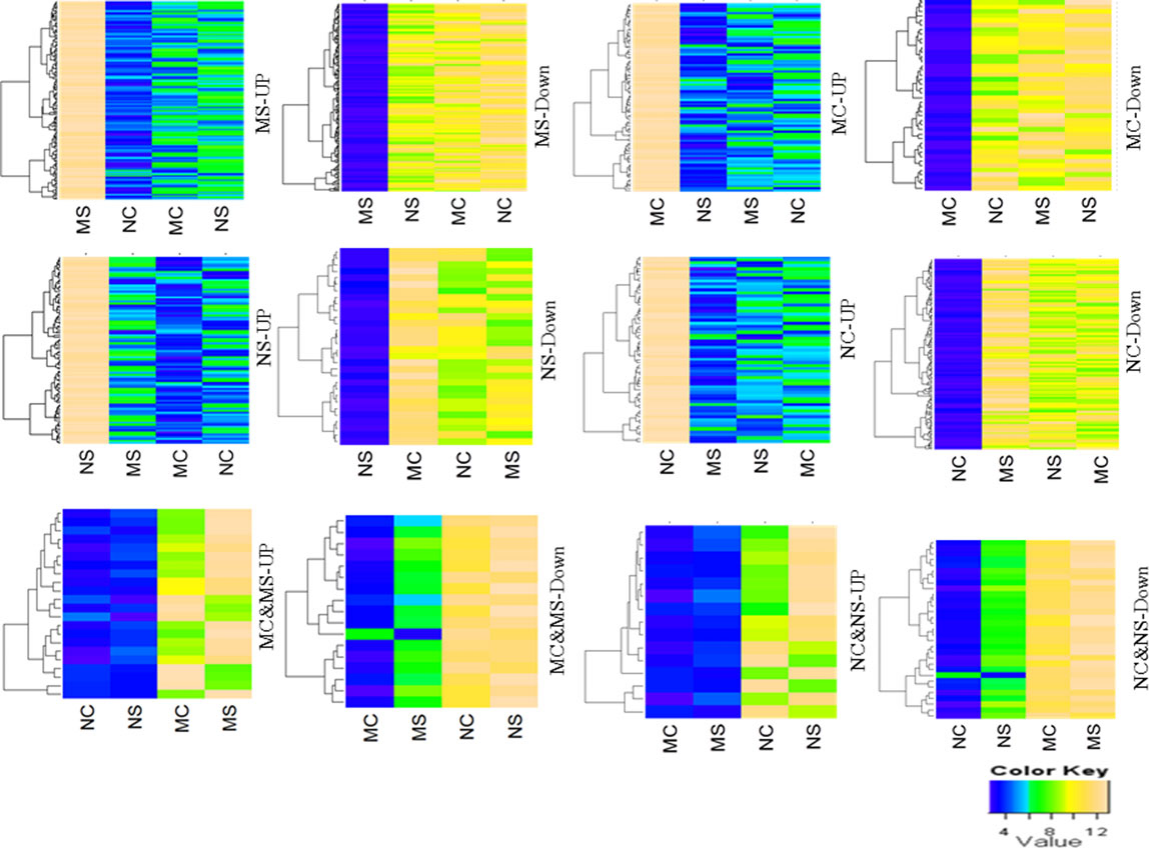
Heatmaps of different clusters of URDEGs on the basis of their expression pattern (MC, mutant control; MS, mutant stress; NC, Nagina control; NS, Nagina stress).

To identify mutant and stress-specific DEGs from the entire gene expression profile generated, a six-way Venn diagram was prepared (Fig. 6A). Out of 57 381 array probes, 16 939 probes (29.5 %) were significantly hybridized and 7534 probes were differentially expressed at ≥2-fold change (*P* < 0.05) in any one of the six possible combinations (MS vs MC, MC vs NC, NS vs MC, MS vs NC, MS vs NS and NS vs NC). The numbers of up- and down-regulated DEGs are presented in Fig. 6B and the detailed gene list is given in **Supporting Information —Table S2**. The Venn analysis identified a total of 873 genes forming 12 clusters of similar expression pattern (up- and down-regulated DEGs in six groups). The number of up- and down-regulated DEGs (Fig. 6C) and the detailed gene list have been given in the **Supporting Information—Table S3**. We have termed these genes as URDEGs. The URDEGs were again subcategorized into up-regulated and down-regulated classes based on their expression in the mutant under control or stress conditions when compared with the WT. The URDEGs, which were up-regulated in the WT were considered as repressed genes in the mutant, while the down-regulated URDEGs in WT were considered as activated genes in the mutant. The heatmaps of URDEGs of these clusters represented the same expression pattern as analysed by our method (Fig. 7). A total of 348 genes showed differential expression specifically under control conditions, while 443 genes did so specifically under stress. However, only 85 genes were found to show differential expression in the mutant under both stress and control conditions when compared with the WT.

### Gene ontology enrichment and pathway analysis of URDEGs

**Figure 8.**
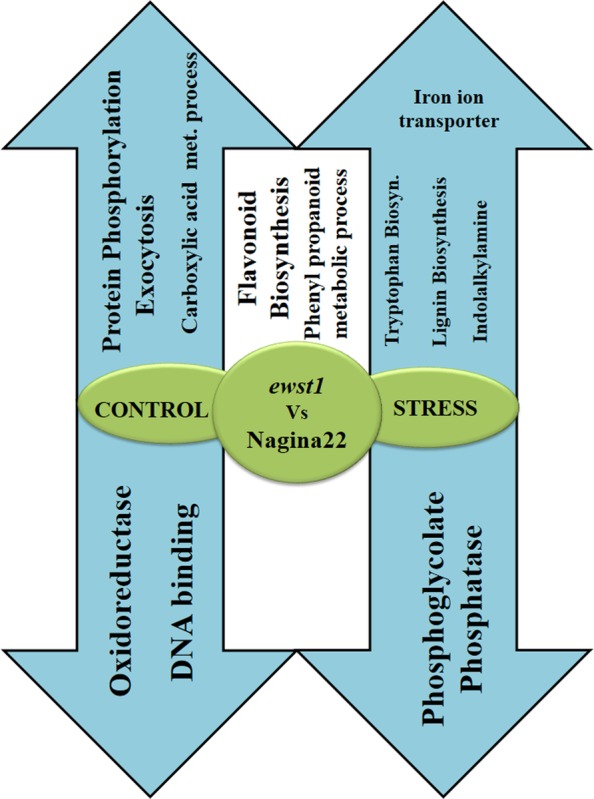
Alterations in biological pathways in the mutant revealed by GO analysis (arrows in the upper and lower direction indicate pathways induced by up-regulated and down-regulated URDEGs, respectively; pathways given in the left, right and middle are altered under control, stress and also both control and stress conditions, respectively).

Functional GO annotations of the identified URDEGs were analysed in terms of GO enrichments for biological, molecular and cellular functions. Under control conditions, biological GO terms of carboxylic acid metabolic process (GO:0019752), protein phosphorylation (GO:0006468) and exocytosis (GO:0006887) were enriched, whereas under stress conditions, genes for tryptophan biosynthesis (GO:0000162), lignin biosynthesis (GO:0009809) and iron ion transport (GO:0006826) were enriched. The GO terms of flavonoid biosynthesis (GO:0009813), phenylpropanoid metabolic process (GO:0009698) and l-phenylalanine catabolic process (GO:0006559) were enriched in the set of up-regulated URDEGs in both control and stress samples. No significant biological function GO term enrichment could be seen in the set of down-regulated genes in *ewst1* either under control or stress conditions. The molecular function GO enrichment analysis of up-regulated URDEGs revealed genes involved in ATP binding (GO:0005524), metal ion binding (GO:0046872), intramolecular lyase activity (GO:0016872), peroxiredoxin activity (GO:0051920), protein serine/threonine kinase activity (GO:0004674) and phosphoheptulonate synthase activity (GO:0003849) under control conditions, whereas genes representing peroxidase activity (GO:0004601), cinamoyl alcohol dehydrogenase (GO:0045551), anthranilate phosphoribosyl transferase (GO:0004048), indole-3-glycerol phosphate synthase (GO:0004425), heme (GO:0020037) and ferric ion binding (GO:0008199) were significantly enriched under stress in *ewst1*. The URDEGs for DNA binding (GO:0003677), Zinc ion binding (GO:0008270) and oxidoreductase (GO:0016702) were down-regulated in *ewst1* under control. Interestingly, there was only one GO term, phosphoglycolate phosphatase (GO:0008967), that was significantly enriched among the down-regulated genes under stress. However, in cellular function GO enrichment analysis, GO terms like exocyst (GO:0000145), intracellular membrane bounded organelle (GO:0043231), membrane (GO:0016020) and extracellular region (GO:0005576) were found to be significant in the up-regulated gene set, whereas nucleus (GO:0005634) GO term was enriched in the down-regulated gene set of URDEGs in control conditions. The overview of GO enrichment analysis is depicted in Fig. 8. Pathway analysis of URDEGs indicated that there were significant expressional alterations in flavonoid biosynthesis, phenylpropanoid biosynthesis, starch and sucrose metabolism and tryptophan biosynthesis-related genes in *ewst1* when compared with WT.

### Modulation of TFs

Out of 873 URDEGs, 74 TFs were differentially regulated in *ewst1* under control and stress conditions when compared with WT. The major stress responsive TF families like the AP2 domain containing EREB, MYB, bHLH, NAC, WRKY, bZIP and ZIM families were differentially expressed. In addition to these, some other TFs such ABI3, Alfin, BZR, C2C2-CO, PLATZ, JUMONJI, GRAS, PHD, Trihelix, homoeobox and G2-like DNA-binding TFs also showed differential expression in *ewst1*. The expression patterns of all TF families in *ewst1* are given in Table 2.

**Table 2.** Expression pattern of TFs in the WT Nagina22 and the mutant *ewst1* under well-watered and water stress conditions. Numbers in parentheses indicate the number of TFs differentially expressed. ^†^↑ and ↓ indicate TFs up-regulated and down-regulated, respectively, under specific treatment (C = under control, S = under stress, C and S = under both control and stress). *Functions of TFs are taken from the online database of rice TF (DRTF), GRASSIUS and RiceFREND.

S. no.	TF family	No. of genes	Expression pattern^†^ of TFs and their TIGR Locus IDs/Gene name	Functions of TF family in plant modification and stress*
1	ABI3-VP1	1	(1)↓S:Os03g06850	Epigenetic regulation, LEA3 regulator
2	Alfin-like	1	(1)↓S: Os02g35600	Histone methylation, associated with drought tolerance
3	AP2-EREBP	7	(4)↑C: Os01g04800, Os01g10370, Os01g54890, Os05g41760, (2)↑S:Os08g36920, Os09g28440, (1)↓S:Os10g22600	Abiotic stress response
4	ARR-B	1	(1)↓S: OsORR22	Cytokinin signalling
5	bHLH	6	(2)↑C: Os01g01870, Os01g06640, (2)↓C: Os01g38610, Os01g72370, (1)↓S: Os02g47660, (1)↓C&S:Os03g53020	Drought tolerance via jasmonate signalling pathway
6	bZIP	4	(2)↑C: Os01g36220, Os02g03960, (1)↓C: Os01g64730, (1)↓S: Os02g10860	Plant development, stress signalling
7	BZR	1	(1)↓S: Os02g03690	Protein phosphorylation and plant development
8	C2C2-GATA	1	(1)↑C: Os02g56250	
9	C2C2-CO-like	2	(1)↑C: Os08g15050, (1)↓S: Os02g05470	
10	C3H	2	(2)↓C: OsC3H-35, Os09g31482	Biotic or abiotic stress and post-transcriptional modification
11	CCAAT-HAP2	1	(1)↓S: OsHAP2C	Photoperiodic flowering, light signalling
12	CCAAT-HAP5	2	(2)↓S: Os03g14669, HTA711	Pollen tube development
13	CPP	1	(1)↓S: Os08g28214	
14	G2-like	3	(2)↓C: PCL1 , Os07g02800, (1)↑S: Os05g40960	Circadian rythm
15	GRAS	2	(2)↑C: Os04g49110, OsCIGR1	Plant phosphorylation, defence and development
16	Homoeobox-zip	3	(2)↓S: OsHOX7, OsHOS66, (1)↑ C&S: Os08g37580	Abiotic stress and plant development
17	HSF	2	(1)↑C:Os05g45410, (1)↑C: HSFC1B	Abiotic stress response
18	JUMONJI	1	(1)↑S: JMJ707	Histone demethylation
19	MYB	9	(1)↑C: Os01g41900, (5)↓C: Os02g09480, Os02g49986, Os12g37970, Os01g09640, Os05g10690, (1)↑S: Os01g18240, (1)↓S: Os01g62410, (1)↑C&S: OsMYB4	Stress and plant development
20	NAC	4	(1)↑C: Os01g64310, (2)↑S: Os03g21030, Os03g56580, (1)↑C&S: OsNAC3	Multiple stress tolerance
21	PHD	2	(2)↓S: Os04g59510, Os11g12650	Histone methylation and post-transcriptional modification
22	PLATZ	1	(1)↓S: Os04g50120	Unknown
23	Trihelix	2	(1)↑S: Os02g01380, (1)↓S: Os04g51320	Stress and cell development
24	WRKY	7	(3)↑C: OsWRKY7, OsWRKY71, OsWRKY76, (1)↓C: OsWRKY77, (2)↑S: OsWRKY11, OsWRKY40, (1)↑C&S: OsWRKY28	Abiotic and biotic stress tolerance
25	ZF-HD	1	(1)↓S: Os08g34010	Regulator of stress-responsive genes
26	ZIM	3	(3)↑C: OsJAZ4, OsJAZ6, Os10g25230	Proteasome degrading jasmonic acid signalling, inhibit apoptosis
27	Orphans	4	(1)↑C: Os03g27080, (1)↑S: Os01g61720, (1)↓S: Os03g06570, (1)↑C&S: Os02g19640	–
	Total	74		

### Enrichment of cis-acting regulatory elements in URDEGs

Out of 873 URDEGs, 680 were found in the Osiris promoter database and subjected to cis-element search. Analysis of the 2000 bp 5′ upstream region of all DEGs revealed that 95 % of genes had the MYBCORE-binding site followed by POLASIG1 (88 %) in their promoter region. Among the top 15 frequently present (>50 % of genes) motifs, three cis-elements, namely MYBCORE, MYCATERD1 and MYCATRD22, were found in the promoter of chymotrypsin inhibitor-like 1 gene. Interestingly, another set of four *cis*-acting regulatory elements, namely POLASIG1, GBOXRELOSAMY3 and PYRIMIDINEBOXOSRAMY1A, all of which are related to the rice α amylase gene, were found to be enriched, being present in more than 65 % of URDEGs **[see Supporting Information—Table S4]**. These results showed that most of the promoter regions of URDEGs had the cis-motif for MYB transcription factor and TF related to α-amylase gene-binding sites.

### Validation of URDEGs in RT-PCR

The DEGs identified by transcriptome analysis were validated using semi-quantitative reverse transcription (RT-PCR) assays in order to check the robustness of the transcriptome results obtained. Out of 24 DEGs tested, 21 genes were successfully amplified, out of which 20 validated the microarray gene expression pattern. Semi-quantitative RT-PCR results of some of the validated DEGs are given in **Supporting Information—Fig. S4**.

## Discussion

The present study screened EMS-induced mutants of an upland rice variety Nagina22 for their response to water stress and characterized the identified mutant(s). Nagina22 has been used in several drought-related studies (Lenka *et al.* 2011; Vikram *et al.* 2011) as it is known to show less water stress-induced spikelet sterility under upland conditions. However, Nagina22 was found to be highly sensitive to dehydration stress induced by 25 % PEG in hydroponic nutrient medium at the seedling stage in our study. Since our objective was to identify mutant(s) having higher tolerance to water stress than the WT, Nagina22, higher level of stress at the early growth and vegetative period was imposed in our study. Nagina22 was found to be susceptible on 40 % PEG-infused MS medium at the germination stage and 25 % PEG in hydroponic nutrient medium at the seedling stage, while *ewst1* performed better under such extreme stress conditions. Moreover, *ewst1* performed better than Nagina22 under soil-water stress at the seedling stage. These findings suggested that although Nagina22 is a known water stress-tolerant variety at the reproductive stage, it exhibits sensitivity at the seedling stage for water stress. Previous studies have shown that PEG at an optimum concentration can be used to screen the seedlings of EMS-induced mutant population (Thang *et al.* 2010; Chutia and Borah 2012) and such screening procedures have helped in identifying a promising EMS-induced *dst* mutant (Huang *et al.* 2009). In the present study, we identified an EMS-induced mutant showing enhanced tolerance to water stress under PEG as well as soil condition when compared with Nagina22.

The mutant showed similarity with regard to most of the DUS characters as well as 100 % identity to the WT based on genotyping using 72 microsatellite markers distributed on 12 different rice chromosomes, establishing it as a true mutant of Nagina22. The undesirable trait of the mutant was 100 % grain chalkiness when compared with the translucent grains of its WT. The mutant however provides scope to study rice grain chalkiness, which is an important grain quality trait (Chun *et al.* 2009), and its relationship with drought tolerance, if any. Interestingly, chalkiness of grains in *ewst1* did not affect its seedling vigour under stress.

Plants respond to environmental stresses through adaptation or avoidance mechanisms by altering a number of morphological and physiological traits (Thapa *et al.* 2011). Such responses can be monitored and recognized by various physiological parameters like RWC (Lawlor and Cornic 2002), CMS (Tripathy *et al.* 2000) and chlorophyll concentration (Moradi and Ismail 2007). Higher RWC, CMS and chlorophyll concentration observed in *ewst1* under water stress indicated that the mutant had greater potential to tolerate water stress, especially at the vegetative stage, than its parent upland variety, frequently used in drought studies in rice. Morphological traits concerning root growth and architecture also play a vital role in environmental adaptations for survival of plants (Gowda *et al.* 2011; Aroca *et al.* 2012). The root growth experiment in a PVC tube indicated that MRL significantly increased in *ewst1* only under stress conditions without having any significant difference with well-watered conditions. Also, in the relative analysis of root growth parameters under control and stress conditions, *ewst1* had a significant increase in RMRL but not in RRW, RRV and RRN, which suggests that the identified mutant possesses deeper root expansion without increasing its biomass, which may be one of the reasons for enhanced stress tolerance (Hsiao and Xu 2000; Serraj *et al.* 2009; Henry *et al.* 2012). Distinct differences were observed in vascular arrangement of cells including xylem and phloem in *ewst1*. The known water stress-tolerant rice varieties like Dular and KDML105 had been reported to have smaller xylem vessel diameter and fewer xylem vessels than the drought-susceptible rice cultivar IR64 under severe water stress (Henry *et al.* 2011). This strengthens our observation that lesser number of xylem vessels in *ewst1* possibly led to enhanced tolerance to water stress. Thus the higher RWC observed in *ewst1* could be a function of either higher water retaining ability during stress or increased water uptake by roots. On the other hand, these traits (RWC, root anatomy and morphology) could be a reflection of enhanced sensitivity in *ewst1* to water stress when compared with its WT. This can be confirmed only when data from plot-based yield studies are generated.

During the last decade, transcriptome analysis using microarray technology has provided an understanding of the genome-wide expression pattern of a species in a trait-specific manner. A number of comparative transcriptome studies have revealed DEGs between contrasting rice genotypes (Lenka *et al.* 2011; Zhang *et al.* 2012*b*) under abiotic stress. In the present study, we employed a strategy to identify mutant and treatment-specific genes, which are termed as URDEGs. Out of 7534 DEGs, we shortlisted only 873 URDEGs, which represented mutant and stress-specific DEGs. This method may also be useful to identify most useful DEGs in comparative studies of contrasting genotypes under stress treatment.

GO enrichment analysis of URDEGs revealed significant alterations in various biological pathways under normal conditions as well as under water stress conditions. Remarkably, under control conditions, genes involved in the exocytosis process were up-regulated in *ewst1*, which might have had a possible role in altered cell division in the mutant (Fendrych *et al.* 2010). The up-regulated URDEGs observed in *ewst1* are involved in the biosynthesis of tryptophan, lignin, indolalkylamine, flavonoids and phenylpropanoid metabolic processes, which have significant roles in protecting the plant from abiotic stresses (Zhao *et al.* 1998; Hernández and van Breusegem 2010; Moura *et al.* 2010; Tounekti *et al.* 2013). Interestingly, genes related to phosphoglycolate phosphatase activity were down-regulated in the mutant under stress. These genes are involved in CO_2_ assimilation and photorespiration (Xu *et al.* 2009), which indicated that *ewst1* had possibly reduced photorespiration, and increased secondary metabolites and osmoprotectants, which might have led to enhanced tolerance to water stress (Voss *et al.* 2013).

A number of TF families are reported to be modulated at the transcriptional and post-transcriptional level under abiotic stresses in plants (Nakashima *et al.* 2009; Ray *et al.* 2011). Many of the differentially expressed TF families of rice like bZIP, AP2/ERF, MYB, ZIM, NAC, HD-ZF, bHLH and WRKY which were modulated under water stress in *ewst1*, might have a possible role in the tolerance mechanism of the same. Some of the previously characterized genes for water stress tolerance and root growth such as OsWRKYs (Xie *et al.* 2005; Wu *et al.* 2009; Peng *et al.* 2010; Yokotani *et al.* 2013), OsNACs (Redillas *et al.* 2012; Jeong *et al.* 2013) and OsJAZs (Seo *et al.* 2011; Yu *et al.* 2012) were enriched in our mutant phenotype, indicating the involvement of these TFs in the enhanced tolerance mechanism of *ewst1*. Since we imposed stress using 25 % PEG 6000, the differential gene expression observed in this study may not be a reflection of the response upon field-based water deficit stress. Hence these results need to be considered with caution while comparing them with other transcriptome data generated under water stress.

*Cis*-regulatory element analysis of the promoter region of URDEGs revealed the presence of elements like MYBCORE, MYCATERD1 and MYCATRD22, which are regulatory binding sites of the MYB transcription factor. Also, *cis*-acting regulatory elements like POLASIG1, GBOXRELOSAMY3 and PYRIMIDINEBOXOSRAMY1A related to the rice α amylase gene were found to be enriched in most of the URDEGs. These α-amylase-related *cis*-elements are regulated by a rice MYB transcription factor called MYBGA or GAMYB (Kaneko *et al.* 2004), which is associated with gibberellin-mediated sugar-signalling pathway (Aya *et al.* 2009). GAMYB shows cross-talk with ABA and gibberellin-signalling pathways (Xie *et al.* 2006) and influences reactive oxygen species (ROS), all of which are known to be involved in stress tolerance in plants (Ishibashi *et al.* 2012). Although the involvement of GAMYB and its possible role in observed transcriptional reprogramming in enhanced water stress of *ewst1* has been suggested by the *cis*-enrichment analysis, this is yet to be confirmed.

In this study, *ewst1* showed multiple morphological, physiological and transcriptomic alterations both under control and stress conditions. Mutation in master regulatory genes, responsible for post-transcriptional modifications, can cause multiple changes in various phenotypic characters like grain number, heading date, plant growth and development, and abiotic stress response accompanied by a large number of transcriptional alterations (Zhang *et al.* 2012*a*; Weng *et al*. 2014). Recently, physiological and proteomic characterization of two chemically induced salt-stress-responsive mutants of rice revealed a number of physiological changes in the mutants corroborated by differential expression of proteins involved in the stress pathway (Ghaffari *et al.* 2014).

The mapping efforts are on in our laboratory, which are expected to give us concrete evidence on the mutated locus. Though the transcriptional profiling indicates that the chalkiness and enhanced stress response could be due to changes in GAMYB, it would be premature to conclude so, without empirical evidence from additional mapping efforts. If these two traits are not due to pleiotropy, then *ewst1* could be a potential resource in rice improvement programmes for drought tolerance.

## Conclusions

In this study, we identified a mutant (*ewst1*) which had enhanced water stress tolerance than the WT Nagina22, a popular upland variety and an international standard in drought tolerance studies in rice. The mutant had expanded root growth, altered root anatomy, chalky endosperm and multiple transcriptional changes without affecting many of the DUS characters and microsatellite genotyping pattern, indicating that *ewst1* was genetically pure and closely related to its WT. Hence, the multiple changes observed in the *ewst1* transcriptome could be due to point mutation in key regulator gene(s) with pleiotropic effects. Therefore, *ewst1* presented in this study can be used as a model mutant to understand the relationship of deeper root penetration, root anatomy, stomatal closure and grain chalkiness in rice in relation to drought tolerance. Genetic mapping, cloning and characterization of *ewst1* will provide deeper insights into water stress tolerance and associated changes in rice.

## Sources of Funding

This work was supported by Department of Biotechnology, New Delhi, Government of India through research grant (BT/PR 9264/AGR/02/406/2007 dated 30 November 2007) for the project ‘Generation, Characterization and use of EMS-induced mutants of upland variety Nagina-22 functional genomics in rice’.

## Contributions by the Authors

T.M. conceived the study and supervised the work. J.M.L. conducted the experiments and analysed the data. M.N. assisted in stomatal and root anatomy study. P.D., K.V.R., K.P.K., C.V. and S.P.S. took part in phenotyping. J.M.L., S.V.A.M. and T.M. drafted and edited the manuscript. U.B.M. participated in work supervision and drafting of the manuscript. T.M., V.C., S.R., N.S., M.S., K.S., A.K.S., N.K.S. and R.P.S. generated the mutant resource used for stress screening in this study.

## Conflict of Interest Statement

None declared.

## Supporting Information

The following additional information is available in the online version of this article –

**Figure S1.** Root transverse sections of Nagina22 at three different regions of root length.

**Figure S2.** (A) Initial screening of mutants in hydroponic culture medium containing 25 % PEG6000. (B) Verification of tolerance behaviour of selected tolerant mutants under PEG stress.

**Figure S3.** Stomatal view of 45-day-old leaves of *ewst1* and WT under the scanning electron microscope.

**Figure S4.** Validation of the microarray result for some URDEGs by semi-quantitative reverse transcription PCR (NC = Nagina22 control, NS = Nagina22 stress, MC = mutant control and MS = mutant stress). First row in gel indicates the expression of actin followed by the expression of DEGs (MSU Locus ID) represented on the right side.

**Table S1.** List of 72 primers distributed on 12 rice chromosomes used for SSR genotyping.

**Table S2.** Sheet 1. Similarity indices of biological replicates of four used samples. Sheet 2. List of differentially expressed (≥2-fold) genes in six possible combinations with their expression. Sheets 3–8. List of DEGs for individual combinations.

**Table S3.** Sheet 1. List of URDEGs with their MSU ID and functional annotation. Sheets 2–13. List of URDEGs for individual clusters obtained from six-way Venn analysis.

**Table S4.** List of cis-elements identified by promoter analysis.

Additional Information
