# Supplementary material for: Physiological, anatomical and transcriptional alterations in a rice mutant leading to enhanced water stress tolerance
Source: AoB Plants. 2015 Mar 27;7:plv023. doi: 10.1093/aobpla/plv023 (PMC4482838; doi:10.1093/aobpla/plv023)
Supplement: Additional Information [file supp_7_plv023_index.html]

Physiological, anatomical and transcriptional alterations in a rice mutant leading to enhanced water stress tolerance — Additional Information 

# Physiological, anatomical and transcriptional alterations in a rice mutant leading to enhanced water stress tolerance

## Additional Information

Additional Information

- Figure S1 - pdf file
- Figure S2 - pdf file
- Figure S3 - pdf file
- Figure S4 - pdf file
- Table S1 - pdf file
- Table S2 - pdf file
- Table S3 - pdf file
- Table S4 - pdf file
