# Supplementary material for: Physiological, anatomical and transcriptional alterations in a rice mutant leading to enhanced water stress tolerance
Source: AoB Plants. 2015 Mar 27;7:plv023. doi: 10.1093/aobpla/plv023 (PMC4482838; doi:10.1093/aobpla/plv023)
Supplement: Additional Information [file supp_plv023_plv023supp_fig1.pdf]

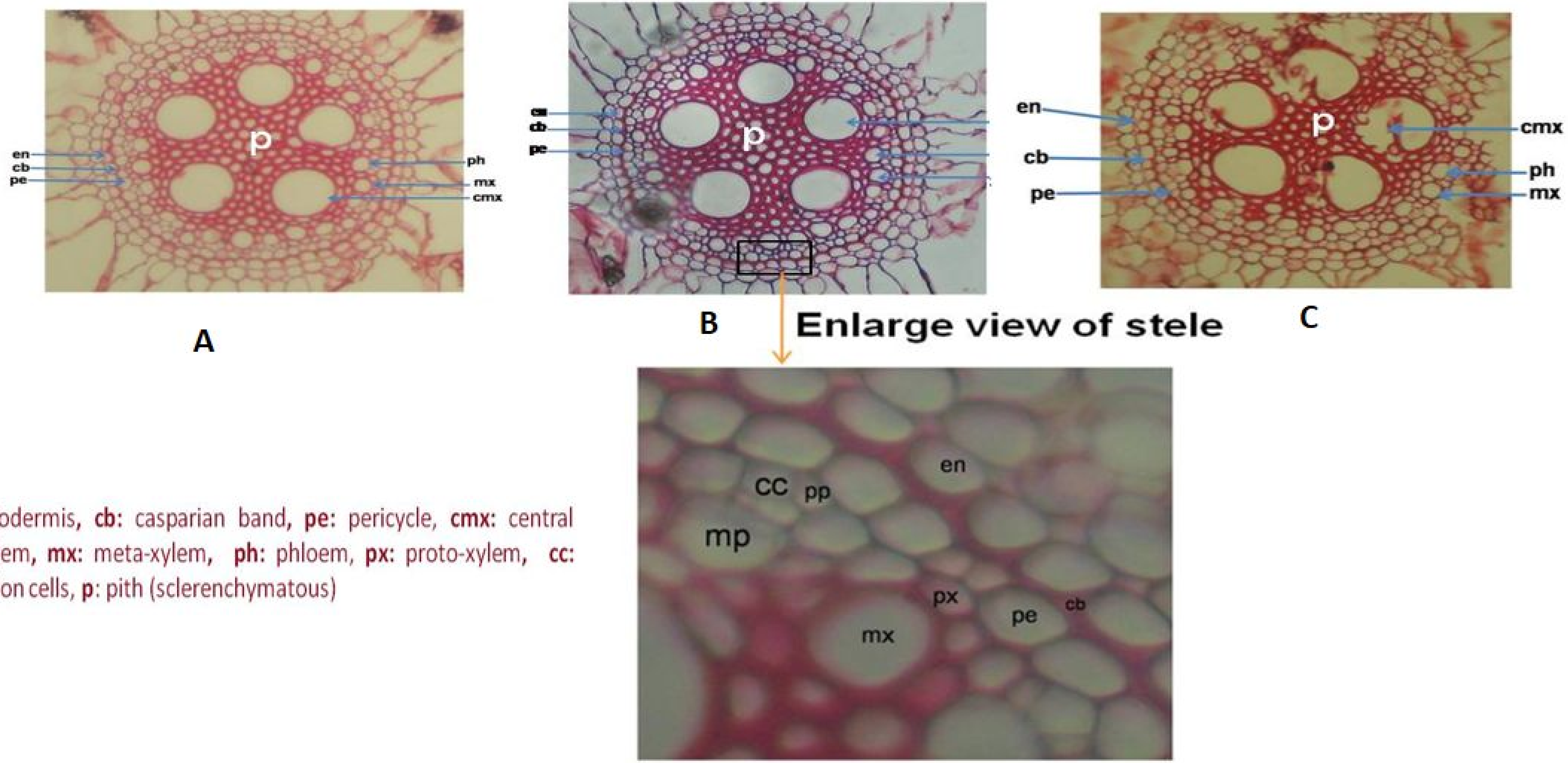

en: endodermis, **cb**: casparian band, **pe**: pericycle, **cmx**: central meta-xylem, **mx**: meta-xylem, **ph**: phloem, **px**: proto-xylem, **cc**: companion cells, **p**: pith (sclerenchymatous)

Supplementary Fig.1. Root anatomical transverse sections of Nagina22 at three different regions of root length (A) 1cm from root tip (B) 3cm from root tip (C) 5cm from root tip
