# Supplementary material for: Physiological, anatomical and transcriptional alterations in a rice mutant leading to enhanced water stress tolerance
Source: AoB Plants. 2015 Mar 27;7:plv023. doi: 10.1093/aobpla/plv023 (PMC4482838; doi:10.1093/aobpla/plv023)
Supplement: Additional Information [file supp_plv023_plv023supp_fig2.pdf]

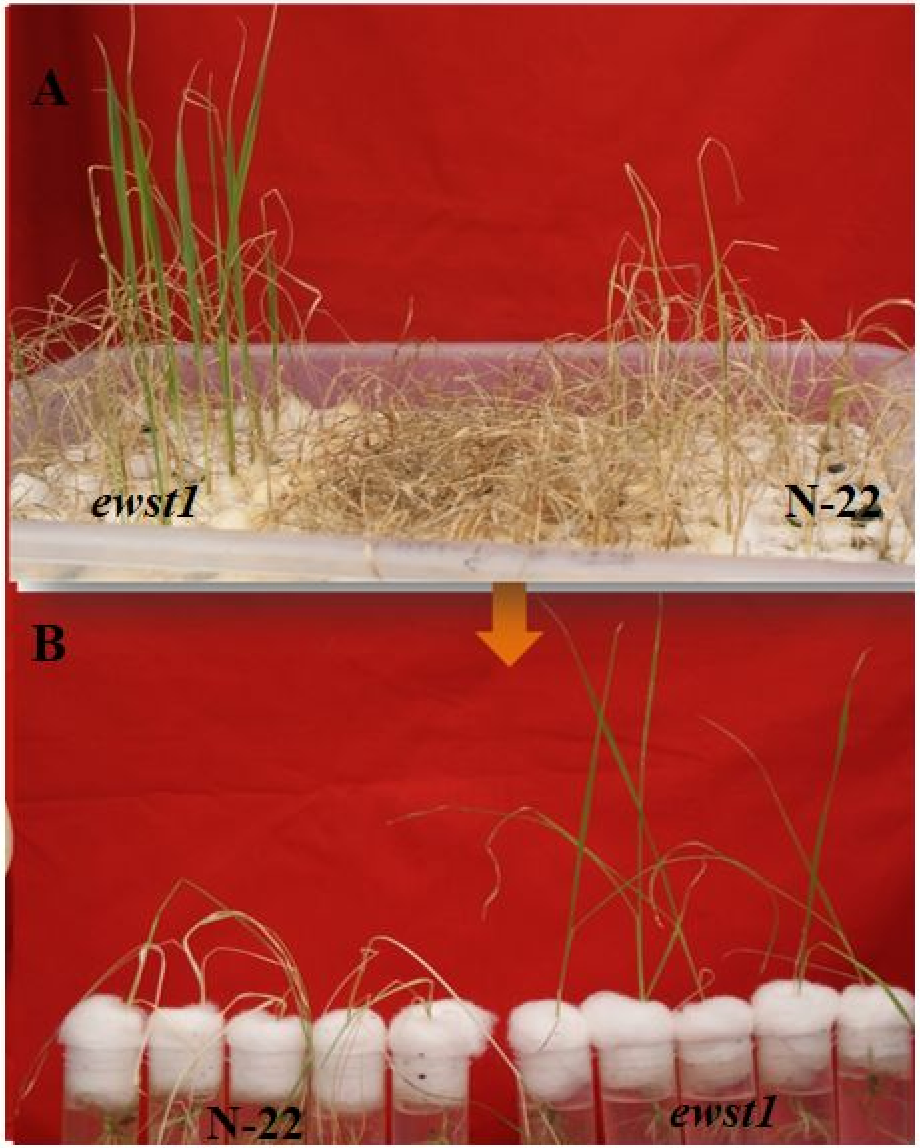

Supplementary Fig. 2. (A) Initial screening of mutants in hydroponic culture medium containing 25% PEG6000. (B) Verification of tolerance behavior of selected tolerant mutant under PEG stress.
