# Supplementary material for: Physiological, anatomical and transcriptional alterations in a rice mutant leading to enhanced water stress tolerance
Source: AoB Plants. 2015 Mar 27;7:plv023. doi: 10.1093/aobpla/plv023 (PMC4482838; doi:10.1093/aobpla/plv023)
Supplement: Additional Information [file supp_plv023_plv023supp_fig4.pdf]

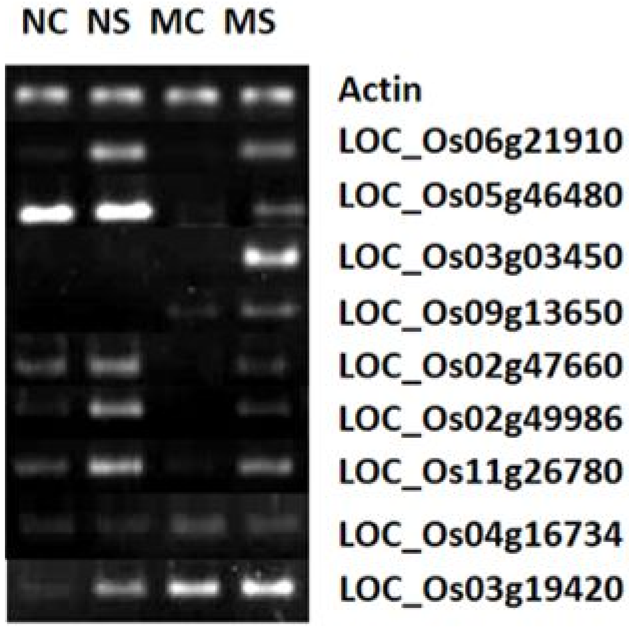

Supplementary Fig. 4 Validation of microarray result for some URDEGs by semi quantitative PCR. (NC=Nagina22 control, NS=Nagina22 stress, MC=Mutant control and MS=Mutant stress). First row in gel indicates the expression of actin followed by the expression of differentially expressed genes (MSU Locus ID) represented on the right side
