# Supplementary material for: Physiological, anatomical and transcriptional alterations in a rice mutant leading to enhanced water stress tolerance
Source: AoB Plants. 2015 Mar 27;7:plv023. doi: 10.1093/aobpla/plv023 (PMC4482838; doi:10.1093/aobpla/plv023)
Supplement: Additional Information [file supp_plv023_plv023supp_table1.pdf]

List of 72 primers distributed on 12 rice chromosomes used for SSR genotyping

S.No.	Primer Names*	Microsatellite repeat-motifs	Forward Primer sequence (5'-3')	Reverse primer sequence (5'-3')
1	RM495	(CTG)7	AATCCAAGGTGCAGAGATGG	CAACGATGACGAACACAACC
2	RM259	(CT)17	TGGAGTTTGAGAGGAGGG	CTTGTTGCATGGTGCCATGT
3	RGNMS5A	(GAG)4	CAGGATCTCTCGACAGGAAA	CTTGTGGTGTTATTGGATTTATT
4	RM5	(GA)14	TGCAACTTCTAGCTGCTCGA	GCATCCGATCTTGATGGG
5	RGNMS135	(GA)26	GAATATCTGTTTCGCCAGATT	ATTGGGAAAGTTGTGTTCTTT
6	RM431	(AG)16	TCCTGCGAACTGAAGAGTTG	AGAGCAAAACCCTGGTTCAC
7	RM154	(GA)21	ACCCTCTCCGCCTCGCCTCCTC	CTCCTCCTCCTGCGACCGCTCC
8	RGNMS13	(AG)25	TTATCAGACAAATTTGAAGCAC	AACATGCACCATTAGTAATCAA
9	RM452	(GTC)9	CTGATCGAGAGCGTTAAGGG	GGGATCAAACCACGTTTCTG
10	RGNMS249	(GA)11	CCCTAGTTGAATCACTCCCTGAGC	TGCTTATGGAAGACGAGAGAAGAGG
11	RGNMS169	(TC)19	CAATGAAATTGGTGGCTCACTGG	TACTAGGTCGGCCCGCTTAGATAGC
12	RGNMS5B	(TTC)6	AGGTTTAATTAGCTCTCTGTCG	ACATTGGAACACCCTAATTAAC
13	RM489	(ATA)8	ACTTGAGACGATCGGACACC	TCACCCATGGATGTTGTCAG
14	OSR13	(GA)n	CATTTGTGCGTCACGGAGTA	AGCCACAGCGCCCATCTCTC
15	RM338	(CTT)6	CACAGGAGCAGGAGAAGAGC	GGCAAACCGATCACTCAGTC
16	RM271	(GA)15	TCAGATCTACAATTCCATCC	TCGGTGAGACCTAGAGAGCC
17	RGNMS1	(GT)8	TACAGAGGATAAAGTGCTTGTG	GTGATCTGCATATATTTGTCGT
18	RM514	(AC)12	AGATTGATCTCCCATTCCCC	CACGAGCATATTACTAGTGG
19	RGNMS20	(AG)18	CTTACTCCATTGGGCTGGAACC	TGTAGGGTGGTAAGAGATCCACTCC
20	RGNMS249	(CT)8	CTACTTCTCCCCTTGTGTCG	TGTACCATCGCCAAATCTCC
21	RM307	(AT)14(GT)21	GTACTACCGACCTACCGTTCAC	CTGCTATGCATGAACTGCTC
22	RM312	(ATTTT)4(GT)9	GTATGCATATTTGATAAGAG	AAGTCACCGAGTTTACCTTC
23	RM237	(CT)18	CAAATCCCGACTGCTGTCC	TGGGAAGAGAGCACTACAGC
24	RM19	(ATC)10	CAAAAACAGAGCAGATGAC	CTCAAGATGGACGCCAAGA
25	RM507	(AAGA)7	CTTAAGCTCCAGCCGAAATG	CTCACCCTCATCATCGCC
26	RGNMS12	(GA)33	GGATCACCAGAGATGAAGAA	CATCCATCCACCATTAATTT
27	RGNMS190	(GA)11	GTCGTCCGAATCGCCTGACTAGG	ACGGCCTCCAGAAGGAAGAGC
28	RM161	(AG)20	TGCAGATGAGAAGCGGCGCCTC	TGTGTCATCAGACGGCGCTCCG

29	RM433	(AG)13	TGCGCTGAACTAAACACAGC	AGACAAACCTGGCCATTCAC
30	RM334	(CTT)20	GTTCAGTGTTCAGTGCCACC	GACTTTGATCTTTGGTGGACG
31	RM133	(CT)8	TTGGATTGTTTTGCTGGCTCGC	GGAACACGGGGTCGGAAGCGAC
32	RGNMS73	(TC)11	GGCTCGTTGACCTAGTCTAAACACC	GTGCAGAAGAAGGCGGAAGG
33	RGNMS141	(TA)40	ATGATTTCACTTGGCGGATGG	ACCATCACGACAGTACGATAGGG
34	RGNMS220	(CT)10	CACTCGCTCCCTCACACAGG	CAACCGGGATTAACATTCAACG
35	RGNMS167	(CT)12	ACCACGCGTCATTGACATCC	ATGGGATGAAACTGCCACAACC
36	RGNMS168	(AG)13	GAGAAGAAATTCAGAGAGCAGAGC	CAACCACATGATCCATATGACG
37	RGNMS151	(AT)15	CGTTCGTCGTTCAGCTTCTCC	ATGCACTGGTGGAGTACGAGAGC
38	RM125	(GCT)8	ATCAGCAGCCATGGCAGCGACC	AGGGGATCATGTGCCGAAGGCC
39	RGNMS193	(GA)10	TTAAGCGCTTTGGTGCTAATCC	CGGGTAGGGTCTCAAGGTAAGG
40	RM11	(GA)17	TCTCCTCTTCCCCCGATC	ATAGCGGGCGAGGCTTAG
41	RM455	(TTCT)5	AACAACCCACCACCTGTCTC	AGAAGGAAAAGGGCTCGATC
42	RM118	(GA)8	CCAATCGGAGCCACCGGAGAGC	CACATCCTCCAGCGACGCCGAG
43	RM152	(GGC)10	GAAACCACCACACCTCACCG	CCGTAGACCTTCTTGAAGTAG
44	RM25	(GA)18	GGAAAGAATGATCTTTTCATGG	CTACCATCAAAACCAATGTTC
45	RM44	(GA)16	ACGGGCAATCCGAACAACC	TCGGGAAAACCTACCCTACC
46	RGNMS165	(TC)10	AGCGGGCCTCTTAACTCACTCG	CCAGGAAAGGAGTGGGAGAGG
47	RM284	(GA)8	ATCTCTGATACTCCATCCATCC	CCTGTACGTTGATCCGAAGC
48	RM447	(CTT)8	CCCTTGTGCTGTCTCCTCTC	ACGGGCTTCTTCTCCTTCTC
49	RM316	(GT)8-((TG)9 (TTTG)4(TG)4	CTAGTTGGGCATACGATGGC	ACGCTTATATGTTACGTCAAC
50	RGNMS56	(CGG)7	AAGAGGATATGCGAAACGGATGG	ACAACCCACCACCACGTCTAGG
51	RGNMS198	(CT)12	TCCATCTTCCTCTCCTAGAGCTTCC	CTCCCTGTCCCGAGTTAGTGC
52	RM105	(CCT)6	GTCGTCGACCCATCGGAGCCAC	TGGTCGAGGTGGGGATCGGGTC
53	RM552	(TAT)13	CGCAGTTGTGGATTTCAGTG	TGCTCAACGTTTGACTGTCC
54	RM215	(CT)16	CAAAATGGAGCAGCAAGAGC	TGAGCACCTCCTTCTCTGTAG
55	RGNMS27	(TA)33	CTATATGACTATGCGAATGG	ACAAATGCAACTAAGGTAGA
56	RM474	(AT)13	AAGATGTACGGGTGGCATTC	TATGAGCTGGTGAGCAATGG
57	RGNMS23	(TC)12	GATGGTAAAGGAAGAACGTGTGC	CACTCATAGACGCATCACATAGCC
58	RGNMS309	(TC)19	TCAACCTCCACTCCTCCTTTGG	TAAATCGCATGAACTCCCAACC

59	RM171	(GATG)5	AACGCGAGGACACGTACTTAC	ACGAGATACGTACGCCTTTG
60	RM484	(AT)9	TCTCCCTCCTCACCATTGTC	TGCTGCCCTCTCTCTCTCTC
61	RGNMS28	(AT)15	GCATGCTAGCTACTAATTGTGTGG	CTTTAGTTACCCAACGTACTCTCTCC
62	RGNMS29	(AG)9	TCACGCACAGCGTGCCGTTCTC	CAAGATCAAGCCATGAAAGGAGGG
63	RGNMS3010	(GA)19	GAGCAAGAGGATGAGGGTGATTGG	CTGTCTCATGTGCCTCCACTCG
64	RM287	(GA)21	TTCCCTGTTAAGAGAGAAATC	GTGTATTTGGTGAAAGCAAC
65	RGNMS130	(GA)10	ACCCATAATCCGTTGGTGTCTGG	GCTCGGCCAAGAGAAAGAAAGAGC
66	RM144	(ATT)11	TGCCCTGGCGCAAATTTGATCC	GCTAGAGGAGATCAGATGGTAGTGCATG
67	RGNMS32	(TC)17	CTTGGTTTGGTTGTGTCCAAGC	GTTCTGATGAACAGGCCGTAGC
68	RGNMS38	(AATA)5	CTGCCTTTCTTACCCCCTTC	AACCCCTCGCTGGATTCTAG
69	RGNMS45	(CGG)8	TGCTCCTCCACCGTCACGTACC	CTATCCGTGCGCCTCAACTTTCC
70	RGNMS175	(CT)13	GGGACTTGGGACCAGTTTATGG	TCAGGTCTGTTGGATTCCATGC
71	RM277	(GA)11	CGGTCAAATCATCACCTGAC	CAAGGCTTGCAAGGGAAG
72	RGNMS60	(TC)18	TTGCTACTACCACAACAGGGTTCC	GCAGCCACAGCTTTGAATAGAGC

RM Rice MicroSatellites [McCouch et al. 2002 (DNA Res 9:199-207) and IRGSP 2005 (Nature 436:793-800)]

RGNMS Rice Genic Non-coding MicroSatellite [Parida et al. 2009 (BMC Genomics 10:140)]

Estimated annealing temperature (0C)	Estimated product size (bp)	Chromosome number	Physical position (bp)
55	150	1	216131
55	190	1	7445813
55	159	1	12657408
55	140	1	23971480
55	155	1	34862016
55	260	1	38893992
60	230	2	1084056
55	190	2	4414015
60	200	2	9563495
55	160	2	17966840
55	256	2	26798239
55	162	2	31456320
55	280	3	4333931
53	120	3	7126082
55	180	3	13221646
55	120	3	17794688
59	196	3	28542771
55	250	3	35281240
55	200	4	177134
55	200	4	6574396
55	190	4	13141945
55	110	4	20233704
55	150	4	25655048
55	250	4	29798552
55	250	5	102742
55	295	5	6826928
58	250	5	11314664
60	180	5	20848244

53	240	5	24813894
55	200	5	28389064
60	240	6	226944
55	150	6	7083449
58	260	6	11449922
56	240	6	19911977
55	170	6	25996122
55	160	6	30348783
55	200	7	154719
60	140	7	5479477
55	190	7	11353903
55	150	7	19257022
57	140	7	22350620
65	160	7	26636604
55	150	8	683095
55	160	8	4377460
55	140	8	11758424
55	150	8	15739760
55	160	8	21142474
55	150	8	26547084
55	200	9	1075107
55	180	9	3664159
55	200	9	9449227
63	140	9	12549315
55	250	9	16149333
55	160	9	21189104
55	200	10	51785
55	290	10	1819032
55	110	10	5352774
60	300	10	13221067

55	347	10	19049104
57	290	10	21066728
56	170	11	2404031
55	150	11	5741183
55	150	11	11103286
55	120	11	16767512
55	200	11	23001251
57	290	11	28281712
55	192	12	2304378
60	200	12	5104306
60	390	12	8831676
60	290	12	15135025
55	120	12	22331236
55	150	12	25965402
