# Supplementary material for: Physiological, anatomical and transcriptional alterations in a rice mutant leading to enhanced water stress tolerance
Source: AoB Plants. 2015 Mar 27;7:plv023. doi: 10.1093/aobpla/plv023 (PMC4482838; doi:10.1093/aobpla/plv023)
Supplement: Additional Information [file supp_plv023_plv023supp_table2.pdf]

Probe Set ID	Fold change([M	Regula	Fold chang	Regulation	Fold chang	Regulation	Fold chang	Regulation
Os.7537.1.S1_at	18.808315	up	2.165742	up	98.44906	up	40.73396	up
Os.22731.1.S1_at	112.53433	up	1.368521	up	130.1769	up	154.0056	up
Os.25639.1.S1_at	97.834496	up	1.005633	up	149.9916	up	98.38556	up
Os.2292.3.S1_x_at	78.04714	up	1.343049	up	82.64259	up	104.8211	up
Os.9950.1.S1_at	39.119576	up	2.285126	up	48.09911	up	89.39318	up
Os.11039.3.S1_at	38.808975	up	-2.18154	down	227.0387	up	17.78969	up
Os.37773.1.S1_at	53.069767	up	1.64926	up	53.75867	up	87.52588	up
Os.519.1.S1_at	53.782055	up	-1.06478	down	83.23041	up	50.50991	up
OsAffx.12954.1.S1_at	268.18048	up	-3.02291	down	218.2883	up	88.71597	up
Os.12257.1.S1_at	13.15643	up	5.165734	up	13.22022	up	67.96262	up
Os.37773.1.S1_x_at	49.577374	up	1.291531	up	51.26835	up	64.03072	up
Os.11046.1.S1_at	16.064854	up	4.25422	up	14.99397	up	68.34344	up
Os.11039.1.S1_s_at	11.195188	up	-1.42063	down	83.48523	up	7.880448	up
Os.8971.1.S1_at	32.78926	up	1.284059	up	44.18456	up	42.10333	up
Os.2292.2.S1_at	60.384754	up	1.207318	up	45.75954	up	72.90358	up
Os.11039.2.S1_x_at	15.788522	up	-1.47787	down	79.60088	up	10.68332	up
Os.22730.1.S1_at	27.031012	up	1.488145	up	34.62421	up	40.22605	up
Os.36307.1.S1_at	4.1960588	up	1.079469	up	46.77172	up	4.529513	up
Os.51460.1.S1_at	26.66265	up	-1.02854	down	44.22883	up	25.92285	up
Os.11112.1.S1_s_at	4.0383945	up	11.12021	up	3.555582	up	44.90781	up
Os.11941.1.S1_at	45.274464	up	-1.19213	down	41.7514	up	37.97793	up
Os.49648.1.S1_s_at	35.209183	up	-1.95029	down	68.00893	up	18.05328	up
Os.11941.2.S1_at	23.098114	up	1.455988	up	23.21511	up	33.63056	up
Os.49648.1.S1_at	55.86394	up	-3.3901	down	105.3598	up	16.47854	up
Os.51789.1.S1_at	33.797443	up	-3.59236	down	107.6244	up	9.408153	up
Os.6043.1.S1_at	2.1815443	up	28.84189	up	1.028829	up	62.91987	up
Os.51546.1.S1_at	2.510443	up	38.01514	up	-1.2867	down	95.43484	up
Os.37217.1.S1_at	4.8989005	up	4.895581	up	5.718612	up	23.98297	up
Os.10942.1.S1_a_at	20.727491	up	1.736151	up	15.86393	up	35.98605	up
Os.50053.1.A1_at	38.583706	up	1.07117	up	25.56113	up	41.32971	up
Os.35463.1.S1_at	17.350578	up	2.081596	up	13.12709	up	36.11689	up
Os.46024.1.S1_at	25.089472	up	-1.24992	down	30.96469	up	20.07283	up
Os.6938.1.A1_at	12.711222	up	1.747707	up	14.10232	up	22.2155	up
OsAffx.10905.3.S1_at	3.7113178	up	1.01311	up	24.19662	up	3.759972	up
Os.50642.1.S1_at	6.435596	up	4.018338	up	5.955673	up	25.8604	up
OsAffx.18681.1.S1_s_	13.550247	up	-1.10537	down	25.63401	up	12.25856	up
Os.55306.1.S1_at	75.87998	up	1.10003	up	18.69895	up	83.47025	up
Os.9238.2.S1_at	38.013004	up	-2.47437	down	50.11258	up	15.36269	up
Os.55509.1.S1_at	10.721935	up	1.546335	up	12.90611	up	16.57971	up
Os.8926.1.S1_at	18.945213	up	-1.01325	down	19.87506	up	18.6974	up
Os.4775.1.S1_at	10.68211	up	-2.22806	down	43.49406	up	4.794362	up
OsAffx.13615.1.S1_at	5.3884797	up	-1.18961	down	23.13581	up	4.529611	up
Os.5574.1.S1_s_at	28.270447	up	-1.57859	down	30.65062	up	17.90866	up
Os.39876.1.S1_at	6.001463	up	3.490325	up	5.40118	up	20.94706	up
Os.55671.1.S1_at	8.194143	up	2.361867	up	7.460967	up	19.35348	up
Os.28217.1.S1_at	30.789854	up	-1.44644	down	25.14834	up	21.28666	up

OsAffx.15339.1.S1_at	20.835619	up	-2.96399	down	49.40418	up	7.029577	up
Os.12221.1.S1_at	2.1689672	up	-2.00226	down	33.10155	up	1.083258	up
Os.23618.1.S1_at	5.674145	up	1.224314	up	13.45258	up	6.946938	up
Os.41109.1.S1_at	3.7169392	up	7.391097	up	2.225251	up	27.47225	up
Os.7366.1.S1_at	27.489622	up	-1.5976	down	26.27297	up	17.20688	up
Os.54535.1.S1_at	4.8202033	up	1.763558	up	9.263081	up	8.500709	up
Os.8481.2.S1_at	10.137801	up	1.372338	up	11.86193	up	13.91249	up
Os.54145.1.S1_at	8.231763	up	-2.01323	down	32.11845	up	4.088839	up
Os.5817.1.S1_at	33.606617	up	-2.08225	down	33.15376	up	16.13959	up
Os.40021.1.S1_a_at	6.064766	up	3.735729	up	4.243577	up	22.65632	up
OsAffx.27442.1.S1_at	16.02695	up	1.221286	up	12.92562	up	19.57349	up
Os.2884.1.S1_at	19.890856	up	1.343503	up	11.56961	up	26.72341	up
Os.5698.1.S1_s_at	15.035804	up	1.221909	up	12.6874	up	18.37239	up
Os.57467.1.S1_x_at	19.62137	up	-1.77485	down	26.36471	up	11.05522	up
Os.23207.1.S1_at	5.4910564	up	4.09285	up	3.61007	up	22.47407	up
Os.4775.2.S1_at	2.3769941	up	-2.23318	down	32.88598	up	1.064397	up
Os.49849.1.S1_at	11.216371	up	2.629641	up	5.547268	up	29.49502	up
Os.12693.3.S1_at	8.4693165	up	1.837074	up	7.875748	up	15.55876	up
Os.10280.1.A1_at	6.935475	up	1.024907	up	14.00628	up	7.108217	up
Os.12812.1.S1_at	2.3344429	up	-2.07065	down	29.72092	up	1.127394	up
Os.37238.1.S1_at	12.535402	up	1.019133	up	13.95369	up	12.77524	up
Os.4385.1.S1_at	7.944053	up	2.418982	up	5.826922	up	19.21652	up
Os.55564.1.S1_at	2.803048	up	-1.33198	down	18.77293	up	2.104415	up
Os.55330.1.S1_at	4.062477	up	1.115114	up	12.58229	up	4.530124	up
OsAffx.13615.1.S1_x_	4.2972746	up	1.101488	up	12.65878	up	4.733396	up
Os.46956.1.S1_at	5.198898	up	6.70509	up	2.068238	up	34.85908	up
Os.14820.1.S2_s_at	-10.659846	down	1.365122	up	-18.5585	down	-7.80871	down
OsAffx.15190.1.S1_at	-8.82866	down	1.345411	up	-17.9408	down	-6.56205	down
OsAffx.20062.1.S1_at	-6.819171	down	-1.0707	down	-12.3005	down	-7.30128	down
Os.55479.1.S1_at	17.867731	up	1.645929	up	7.977617	up	29.40902	up
Os.24611.1.S1_at	20.982292	up	-2.17624	down	28.39519	up	9.641547	up
Os.41109.1.S1_x_at	3.3616834	up	6.573593	up	1.922921	up	22.09834	up
Os.38581.1.S1_s_at	15.574317	up	-1.33022	down	16.75065	up	11.70804	up
Os.12498.2.S1_at	3.0244465	up	9.461882	up	1.32063	up	28.61696	up
Os.54707.1.S1_x_at	18.64937	up	-1.27147	down	15.70882	up	14.66757	up
Os.16659.1.S2_at	13.967342	up	1.013602	up	12.07101	up	14.15733	up
Os.46849.1.S1_at	1.0482291	up	20.80446	up	-1.70111	down	21.80784	up
Os.54146.1.S1_at	5.0450006	up	1.317325	up	9.136794	up	6.645906	up
Os.6162.1.S1_at	10.339598	up	1.164776	up	10.31768	up	12.04332	up
Os.55837.1.S1_at	2.1584277	up	-1.17686	down	13.57877	up	1.834063	up
Os.14105.1.S1_at	1.8512195	up	15.94034	up	-1.38457	down	29.50906	up
Os.27956.1.S1_at	1.9310172	up	-1.82675	down	20.92934	up	1.05708	up
Os.1723.1.S1_at	15.543177	up	1.132668	up	10.0947	up	17.60526	up
Os.56080.1.S1_at	1.6847389	up	20.38831	up	-1.7839	down	34.34898	up
Os.53526.1.S1_at	4.936994	up	-3.55271	down	40.50728	up	1.38964	up
OsAffx.19095.1.S1_at	6.4096236	up	2.803989	up	4.04974	up	17.97251	up
Os.37295.1.S1_at	1.530405	up	6.759543	up	1.662791	up	10.34484	up

Os.14820.1.S1_s_at	-9.317789	down	1.306584	up	-14.5111	down	-7.13141	down
OsAffx.27508.10.S1_s	3.3042455	up	-1.60352	down	17.80529	up	2.060616	up
Os.8481.4.S1_x_at	16.274446	up	-1.04315	down	11.47202	up	15.60121	up
Os.26437.1.A1_s_at	-8.397618	down	1.039207	up	-11.3809	down	-8.08079	down
Os.4463.1.S1_s_at	19.886938	up	-1.00222	down	10.96511	up	19.84287	up
Os.51991.1.S1_at	3.654911	up	2.595443	up	4.187902	up	9.486113	up
Os.14125.1.S1_at	-8.263579	down	1.427655	up	-15.5011	down	-5.78822	down
Os.8724.1.S1_at	31.962612	up	1.114011	up	9.723923	up	35.60672	up
Os.49787.1.S1_at	7.7265983	up	2.630353	up	4.067548	up	20.32368	up
Os.56075.1.S1_at	6.8122673	up	2.331676	up	4.582391	up	15.884	up
Os.28301.1.S2_at	-12.204402	down	-1.11902	down	-9.51806	down	-13.657	down
Os.3280.1.S1_at	15.356533	up	-6.4729	down	68.63326	up	2.372434	up
Os.16245.1.S1_at	11.858995	up	-1.33973	down	14.17076	up	8.851776	up
Os.55944.1.S1_at	8.312033	up	1.213733	up	8.660959	up	10.08859	up
Os.49245.1.S1_at	247.26797	up	-13.8944	down	145.7819	up	17.79622	up
Os.56074.1.S1_at	5.743849	up	2.166445	up	4.84061	up	12.44373	up
Os.27716.2.S1_a_at	14.123787	up	-1.02862	down	10.73392	up	13.73083	up
Os.28441.1.S1_at	22.102314	up	-1.73054	down	18.05417	up	12.77188	up
Os.50831.1.S1_at	7.558611	up	1.833867	up	5.668273	up	13.86149	up
Os.39652.1.S1_at	-4.712888	down	-2.79748	down	-3.69388	down	-13.1842	down
OsAffx.24678.3.S1_at	26.511215	up	1.006499	up	10.24998	up	26.68352	up
Os.27474.1.S1_at	-10.354797	down	-1.51275	down	-6.79465	down	-15.6642	down
Os.9988.1.S1_at	28.565392	up	-16.7573	down	171.6745	up	1.704652	up
OsAffx.18737.1.S1_at	86.60569	up	-13.0156	down	130.623	up	6.653998	up
OsAffx.24099.1.S1_x_	13.422977	up	-1.25235	down	12.50583	up	10.71826	up
OsAffx.13543.1.S1_s_	18.311201	up	1.176641	up	8.439416	up	21.54571	up
Os.3391.1.S1_at	15.580838	up	1.726523	up	5.744505	up	26.90067	up
Os.51758.2.S1_at	1.9230213	up	-1.06503	down	10.52896	up	1.805596	up
Os.53457.1.S1_at	2.4624653	up	-1.29797	down	12.73717	up	1.897164	up
Os.8569.1.S1_at	3.3782866	up	2.010902	up	4.844827	up	6.793405	up
Os.15992.1.S1_at	6.4987335	up	1.374772	up	7.055387	up	8.934278	up
Os.12244.1.S1_at	12.034184	up	-1.82887	down	17.68016	up	6.580133	up
Os.15854.1.S1_at	1.3351625	up	20.40843	up	-2.16124	down	27.24858	up
Os.11029.1.S1_at	3.90097	up	2.500867	up	3.740665	up	9.755806	up
Os.10259.1.S1_at	4.6638236	up	1.980209	up	4.719711	up	9.235344	up
Os.8088.1.S1_at	5.2825146	up	10.88968	up	-1.17248	down	57.52488	up
Os.11179.1.S1_at	-7.2875586	down	-1.18514	down	-7.79327	down	-8.63676	down
Os.8481.2.S1_x_at	11.361558	up	-1.00445	down	9.259031	up	11.31123	up
Os.51226.1.S1_at	7.483332	up	2.807421	up	3.279249	up	21.00886	up
Os.38806.1.A1_x_at	3.021315	up	3.168135	up	2.904136	up	9.571935	up
Os.25311.1.A1_at	9.634462	up	-1.16771	down	10.7342	up	8.250709	up
Os.16317.1.S1_at	10.025966	up	-1.44805	down	13.3068	up	6.923761	up
Os.9311.1.S1_at	1.1130251	up	15.9468	up	-1.73621	down	17.74918	up
Os.18615.1.S1_at	2.5158238	up	2.574284	up	3.561808	up	6.476444	up
OsAffx.15339.1.S1_s_	11.164001	up	-2.51614	down	23.04748	up	4.436951	up
OsAffx.24957.1.S1_at	16.636303	up	-1.75514	down	16.05349	up	9.478642	up
Os.9191.1.S1_s_at	19.666386	up	-5.10146	down	45.56918	up	3.855049	up

Os.31464.1.S1_a_at	17.995464	up	-1.64111	down	14.59152	up	10.9654	up
Os.7664.1.S1_at	3.6656067	up	2.945693	up	3.014245	up	10.79775	up
Os.11952.1.S1_at	2.3834941	up	6.09795	up	1.447903	up	14.53443	up
Os.49636.1.S1_at	6.449309	up	-1.19538	down	10.55065	up	5.395187	up
OsAffx.13014.1.S1_s_	4.4716864	up	1.113571	up	7.916616	up	4.979541	up
Os.50015.1.S1_at	1.3324572	up	20.24944	up	-2.31923	down	26.98152	up
Os.5504.1.S2_at	12.169292	up	-1.2909	down	11.25798	up	9.426948	up
Os.11253.1.S1_at	3.1381557	up	1.417989	up	6.104377	up	4.449871	up
Os.51926.1.S1_at	5.8895326	up	1.903638	up	4.528358	up	11.21154	up
OsAffx.12799.1.S1_s_	26.309834	up	-1.64371	down	14.15898	up	16.00638	up
Os.22596.1.S1_at	-3.9354234	down	-2.14142	down	-4.01996	down	-8.42739	down
Os.11106.1.S1_at	16.7766	up	-3.82967	down	32.93857	up	4.380693	up
Os.55330.1.S1_x_at	3.6031702	up	-1.21747	down	10.46602	up	2.959552	up
Os.38309.1.S1_at	-3.5958302	down	-2.03579	down	-4.20607	down	-7.32035	down
Os.9286.1.S1_s_at	2.973915	up	2.71609	up	3.131055	up	8.07742	up
Os.16044.1.S1_at	6.858857	up	-3.02107	down	25.56261	up	2.270339	up
Os.54163.1.S1_at	3.7423122	up	1.177546	up	7.177005	up	4.406745	up
Os.20079.1.S2_at	-9.782474	down	1.088907	up	-9.10046	down	-8.98375	down
Os.8113.1.S1_at	9.820856	up	1.003571	up	8.323581	up	9.855927	up
Os.51794.1.S1_at	11.6939535	up	-3.60984	down	30.06535	up	3.239465	up
OsAffx.32310.1.A1_a	-1.0244117	down	-6.46295	down	-1.28768	down	-6.62072	down
Os.18615.1.S1_x_at	2.6678832	up	2.321003	up	3.542835	up	6.192164	up
Os.8095.1.S4_at	-7.7165008	down	-1.06362	down	-7.71215	down	-8.20743	down
OsAffx.23978.1.S1_at	16.049654	up	-2.88936	down	23.70082	up	5.554741	up
OsAffx.24678.2.S1_at	13.564353	up	1.33493	up	6.13136	up	18.10746	up
Os.6516.1.S1_at	47.128876	up	-1.66167	down	13.58466	up	28.36238	up
Os.13478.1.S1_at	4.8313203	up	1.108356	up	7.314832	up	5.354821	up
Os.51035.1.S1_at	4.979675	up	1.396704	up	5.763616	up	6.955128	up
Os.8549.1.S1_at	6.64486	up	1.187953	up	6.775764	up	7.89378	up
Os.50985.1.S1_at	12.625766	up	1.808297	up	4.443643	up	22.83114	up
Os.7985.1.S1_at	4.5538044	up	4.067126	up	1.969757	up	18.5209	up
Os.4900.1.S1_at	11.025332	up	1.206286	up	6.582722	up	13.2997	up
Os.11575.3.S1_x_at	2.6237724	up	6.061267	up	1.305239	up	15.90339	up
OsAffx.4176.1.S1_at	2.7154062	up	1.275568	up	6.198537	up	3.463685	up
OsAffx.2760.1.S1_at	4.795826	up	-1.78541	down	14.05949	up	2.686119	up
Os.34873.1.S1_at	4.8389707	up	2.336939	up	3.360372	up	11.30838	up
Os.7772.2.A1_at	3.131155	up	1.281788	up	6.095267	up	4.013477	up
Os.3435.1.S1_at	9.450532	up	1.913098	up	4.081178	up	18.0798	up
Os.6284.1.S1_at	4.1204047	up	2.967209	up	2.629926	up	12.2261	up
Os.12452.1.S1_s_at	4.0380654	up	5.974966	up	1.301749	up	24.1273	up
Os.8010.1.S1_at	12.495061	up	-1.86882	down	14.49324	up	6.686088	up
Os.8095.1.S2_at	-7.238977	down	-1.18523	down	-6.53601	down	-8.57982	down
Os.27140.1.S1_a_at	-1.9920398	down	-2.8056	down	-2.75555	down	-5.58887	down
Os.51749.1.S1_at	2.7553139	up	1.111274	up	6.944305	up	3.061909	up
Os.17228.1.S1_at	22.243673	up	-1.3653	down	10.49583	up	16.29219	up
Os.11788.1.S1_at	1.8155978	up	3.110221	up	2.461975	up	5.64691	up
Os.12240.1.S1_at	5.357354	up	1.973964	up	3.872375	up	10.57523	up

Os.51937.1.S1_a_at	5.4185157	up	1.491414	up	5.118244	up	8.081248	up
Os.31022.1.S1_at	5.0340114	up	-2.254	down	17.12752	up	2.233371	up
OsAffx.30250.1.S1_at	-4.6325245	down	-1.54211	down	-4.91693	down	-7.14388	down
Os.6376.1.S1_at	6.747241	up	3.639368	up	2.083325	up	24.5557	up
Os.9923.1.S1_s_at	1.2200134	up	11.18024	up	-1.4788	down	13.64004	up
Os.3435.2.S1_x_at	10.975024	up	1.808097	up	4.148883	up	19.84391	up
Os.27482.1.A1_at	12.7476225	up	-2.17129	down	16.28191	up	5.870986	up
Os.11365.1.S1_at	3.3553295	up	2.327982	up	3.210149	up	7.811149	up
Os.12946.1.S1_at	11.080299	up	1.172332	up	6.368082	up	12.98979	up
Os.12252.1.S1_at	2.8756216	up	-1.80639	down	13.48455	up	1.591914	up
Os.11997.1.S1_at	-1.8362437	down	-1.55003	down	-4.80981	down	-2.84623	down
Os.6349.1.S1_a_at	8.452952	up	1.088554	up	6.82925	up	9.201497	up
Os.9393.1.S1_a_at	-8.45679	down	1.304083	up	-9.61794	down	-6.48485	down
Os.29290.1.S1_at	7.033712	up	2.992431	up	2.461509	up	21.0479	up
Os.46791.1.S1_at	8.530094	up	-2.66152	down	19.4377	up	3.204968	up
OsAffx.28211.1.S1_at	4.7188025	up	2.159236	up	3.374432	up	10.18901	up
Os.7753.1.S1_at	6.0622487	up	-1.67531	down	12.20097	up	3.618579	up
Os.38381.1.S1_at	-5.6811028	down	1.506769	up	-10.9197	down	-3.77039	down
OsAffx.24138.1.S1_s_	6.629988	up	-1.41321	down	10.2046	up	4.691445	up
Os.15281.1.S1_x_at	3.7786248	up	12.46106	up	-1.73376	down	47.08566	up
Os.1193.1.S1_at	1.7936678	up	2.79474	up	2.570919	up	5.012836	up
Os.37699.2.S1_at	6.41759	up	1.071602	up	6.701644	up	6.877104	up
Os.52381.1.S1_at	-4.6389093	down	-1.63573	down	-4.39002	down	-7.588	down
Os.13461.1.S1_at	8.670823	up	-1.34399	down	9.634526	up	6.45155	up
Os.31521.1.S1_at	1.9143184	up	3.488536	up	2.053637	up	6.67817	up
Os.49042.1.A1_s_at	4.0444155	up	1.05667	up	6.774683	up	4.273611	up
Os.55561.1.S1_at	2.2421567	up	-1.10093	down	7.879787	up	2.036595	up
Os.8149.1.S1_at	26.963259	up	-3.20212	down	22.90418	up	8.420431	up
OsAffx.28017.1.S1_at	4.469467	up	1.71678	up	4.16178	up	7.673091	up
Os.50470.1.S1_at	4.811813	up	1.851279	up	3.843667	up	8.908009	up
Os.11786.2.S1_at	9.442037	up	-1.23344	down	8.756987	up	7.655015	up
Os.53622.1.S1_at	19.841618	up	-4.37445	down	31.03657	up	4.535798	up
Os.54145.2.S1_at	3.3149645	up	-1.31685	down	9.335337	up	2.517343	up
OsAffx.15137.1.S1_at	-3.848272	down	-2.25091	down	-3.14685	down	-8.66213	down
Os.8043.1.S1_at	9.538147	up	1.286473	up	5.505709	up	12.27057	up
Os.11354.1.S1_at	-2.2980344	down	-1.09237	down	-6.44726	down	-2.5103	down
Os.30000.1.S1_at	4.1084933	up	5.677829	up	1.236318	up	23.32732	up
OsAffx.4677.1.S1_s_a	5.2052097	up	1.407309	up	4.984725	up	7.325335	up
Os.12190.1.S1_at	6.6180673	up	-1.07388	down	7.504949	up	6.162791	up
Os.49079.1.A1_at	-4.896659	down	1.13853	up	-7.93731	down	-4.30086	down
Os.6542.1.S1_at	-4.5258884	down	-2.43949	down	-2.83199	down	-11.0409	down
Os.55776.1.S1_at	2.2309344	up	4.462433	up	1.542135	up	9.955395	up
Os.2292.1.S1_a_at	9.777482	up	-1.08628	down	7.468684	up	9.000903	up
Os.11250.1.S1_at	11.40277	up	-2.23454	down	15.28239	up	5.102969	up
Os.28406.1.S1_at	-12.271746	down	2.155988	up	-14.7381	down	-5.69194	down
Os.46941.1.S1_s_at	-10.280855	down	-1.44102	down	-4.74178	down	-14.8149	down
Os.33939.1.A1_at	10.531798	up	1.438088	up	4.749591	up	15.14565	up

Os.5504.1.S1_at	13.645514	up	-1.05649	down	7.205334	up	12.91593	up
Os.53392.1.S1_at	6.058687	up	-1.21994	down	8.297941	up	4.966364	up
Os.56029.1.S1_at	-4.8956804	down	1.022954	up	-6.94328	down	-4.78583	down
Os.9975.1.S1_at	-5.4022193	down	1.037818	up	-7.03553	down	-5.20536	down
Os.12735.1.S1_at	-6.2785926	down	-1.31727	down	-5.14342	down	-8.27063	down
Os.51794.1.S1_x_at	6.407942	up	-2.45792	down	16.60961	up	2.607056	up
OsAffx.30145.1.S1_at	6.695578	up	1.110997	up	6.062926	up	7.438769	up
Os.26437.1.A1_at	-8.611881	down	1.384991	up	-9.29377	down	-6.21801	down
OsAffx.23641.1.S1_at	-4.463962	down	-1.58229	down	-4.23229	down	-7.06329	down
Os.12234.2.S1_s_at	3.8874207	up	1.508228	up	4.434798	up	5.863114	up
Os.37376.1.S1_at	8.249705	up	1.611226	up	4.149398	up	13.29214	up
OsAffx.2403.1.S1_at	1.5755945	up	3.436784	up	1.93848	up	5.414978	up
OsAffx.21790.1.S1_at	6.6746507	up	1.988537	up	3.340725	up	13.27278	up
Os.52993.1.S1_at	-3.1602364	down	-1.25087	down	-5.30547	down	-3.95305	down
Os.9513.1.S1_at	3.8954918	up	1.181098	up	5.61174	up	4.600956	up
OsAffx.24189.1.S1_at	7.266422	up	1.325182	up	4.98827	up	9.629334	up
Os.14007.1.S1_at	6.6483517	up	-1.01148	down	6.674732	up	6.572908	up
Os.11812.1.S1_at	4.3144794	up	26.86554	up	-4.07935	down	115.9108	up
Os.12111.1.S1_at	-6.121084	down	-2.19846	down	-2.99031	down	-13.457	down
Os.41242.1.A1_at	-3.367781	down	-3.25234	down	-2.01917	down	-10.9532	down
Os.27170.1.S1_at	11.991803	up	1.055766	up	6.20502	up	12.66054	up
Os.38848.1.S1_at	-5.3551617	down	-1.46566	down	-4.42427	down	-7.84885	down
OsAffx.3952.1.S1_s_a	2.5509188	up	2.937409	up	2.205748	up	7.493091	up
OsAffx.29084.2.S1_x_	4.550425	up	1.181878	up	5.472992	up	5.378046	up
Os.35808.1.S1_at	3.6513083	up	1.81228	up	3.562418	up	6.617194	up
Os.57412.1.S1_at	1.4200547	up	1.682503	up	3.797119	up	2.389247	up
Os.55316.1.S1_at	2.7673132	up	1.487696	up	4.293155	up	4.11692	up
Os.28435.5.S1_at	-2.0427427	down	-4.19608	down	-1.52025	down	-8.57151	down
Os.12781.1.S1_at	4.564236	up	-1.54895	down	9.866943	up	2.946664	up
OsAffx.32256.1.A1_x_	2.0512981	up	-5.27573	down	-1.20594	down	-2.5719	down
Os.8095.1.S3_at	-6.194533	down	-1.15878	down	-5.44257	down	-7.1781	down
Os.57519.1.S1_x_at	16.035248	up	-5.81661	down	36.63102	up	2.756802	up
Os.3428.2.S1_at	2.8463738	up	1.293594	up	4.86569	up	3.682052	up
Os.40002.1.S1_s_at	8.281133	up	1.624607	up	3.868424	up	13.45358	up
Os.51923.1.S1_at	2.8854382	up	5.833658	up	1.075818	up	16.83266	up
Os.12705.1.S1_at	3.4162042	up	1.505471	up	4.16544	up	5.142997	up
Os.12680.1.S1_at	5.3120856	up	1.020975	up	6.120196	up	5.423506	up
OsAffx.15178.1.S1_s_	-10.331259	down	1.806134	up	-11.2267	down	-5.7201	down
OsAffx.21963.1.S1_s_	5.3229737	up	-7.40356	down	46.00549	up	-1.39087	down
OsAffx.31710.1.S1_x_	5.0619326	up	1.260131	up	4.922232	up	6.378696	up
Os.56018.1.S1_at	4.375335	up	4.471014	up	1.382158	up	19.56219	up
Os.6418.1.S1_at	5.2946043	up	1.684353	up	3.639803	up	8.917982	up
Os.7944.1.S1_at	-4.055698	down	-1.4774	down	-4.13949	down	-5.9919	down
Os.413.1.S1_at	11.218073	up	-1.41499	down	8.602331	up	7.928021	up
Os.53568.1.S1_at	3.959486	up	-1.19272	down	7.249232	up	3.319719	up
Os.27039.1.S1_at	-6.7295284	down	-1.609	down	-3.77653	down	-10.8278	down
Os.17136.1.S1_at	7.8430123	up	-1.55984	down	9.458244	up	5.028088	up

Os.28216.1.S1_a_at	1.6090542	up	6.865312	up	-1.13242	down	11.04666	up
OsAffx.10320.1.S1_at	-3.7557805	down	-2.20158	down	-2.74625	down	-8.26865	down
OsAffx.24280.2.S1_at	3.236024	up	-2.57312	down	15.53902	up	1.257628	up
Os.11775.1.S2_at	2.1981902	up	3.82069	up	1.579385	up	8.398603	up
OsAffx.14523.1.S1_s_	6.5889034	up	-4.77327	down	28.76106	up	1.380376	up
Os.34624.1.S1_at	-2.5439928	down	1.15103	up	-6.91685	down	-2.21019	down
Os.55371.1.S1_at	6.602533	up	1.16141	up	5.163787	up	7.668244	up
Os.54859.1.S1_at	3.5767605	up	1.741656	up	3.44127	up	6.229487	up
Os.20614.2.S1_at	-4.048695	down	-1.50109	down	-3.99249	down	-6.07744	down
Os.22596.1.S1_s_at	-3.138891	down	-2.16196	down	-2.76328	down	-6.78617	down
Os.47792.1.S1_at	6.3685703	up	-1.40291	down	8.368945	up	4.539534	up
OsAffx.3970.1.S1_at	-8.314273	down	1.241096	up	-7.39414	down	-6.69914	down
Os.23065.1.S1_at	2.7456048	up	1.150952	up	5.17228	up	3.160059	up
Os.8027.1.S1_at	6.10513	up	1.016279	up	5.849074	up	6.204515	up
Os.49468.2.S1_at	2.8579993	up	1.015844	up	5.846847	up	2.90328	up
Os.20244.1.S1_at	2.10011	up	1.087252	up	5.421791	up	2.28335	up
Os.37834.1.S1_a_at	-4.3756304	down	1.185394	up	-6.9804	down	-3.69129	down
Os.18098.1.S1_at	3.883546	up	1.352301	up	4.324618	up	5.251721	up
Os.18376.1.S1_at	4.8094378	up	-1.24918	down	7.250772	up	3.850081	up
Os.11575.4.S1_x_at	2.5557003	up	4.837877	up	1.199244	up	12.36416	up
Os.10593.1.S1_at	8.668669	up	-1.67212	down	9.64681	up	5.184248	up
Os.10551.1.S1_at	3.7666597	up	-1.14915	down	6.613724	up	3.277784	up
Os.11471.1.S1_at	10.64277	up	3.213052	up	1.788326	up	34.19577	up
Os.4773.1.S1_at	4.0135837	up	2.624313	up	2.188983	up	10.5329	up
Os.27762.1.A1_at	-9.177563	down	1.800914	up	-10.3358	down	-5.09606	down
Os.16659.1.S1_at	3.009092	up	1.766599	up	3.240785	up	5.315857	up
Os.8095.1.S1_at	-6.163354	down	-1.03007	down	-5.5562	down	-6.34866	down
Os.10502.1.S1_at	-10.299306	down	-1.26615	down	-4.51798	down	-13.0404	down
Os.175.1.S1_at	3.8929317	up	-2.81363	down	16.0865	up	1.383599	up
Os.26695.1.S1_at	2.5991669	up	2.878948	up	1.984003	up	7.482866	up
Os.5338.1.S1_at	1.766747	up	-1.38366	down	7.837902	up	1.276866	up
Os.10580.1.S1_at	-2.836168	down	-1.23911	down	-4.56556	down	-3.51433	down
Os.11899.1.S1_at	-2.1900039	down	-4.17793	down	-1.35329	down	-9.14968	down
Os.27494.1.S1_at	-3.6705182	down	-1.12976	down	-4.98522	down	-4.1468	down
Os.9923.1.S1_at	1.601135	up	9.008657	up	-1.6013	down	14.42408	up
Os.17777.2.S1_at	2.5297797	up	1.861433	up	3.012608	up	4.709016	up
Os.30812.1.S1_at	-3.3170474	down	-1.07486	down	-5.20887	down	-3.56535	down
Os.10339.1.S1_at	11.960426	up	-1.47507	down	8.252375	up	8.10839	up
Os.9172.2.S1_at	-2.0847886	down	-1.04723	down	-5.34221	down	-2.18325	down
OsAffx.19357.1.S1_at	1.5320945	up	-4.11896	down	23.00658	up	-2.68845	down
Os.33687.1.S1_at	8.706422	up	-1.78635	down	9.964693	up	4.873851	up
Os.8908.1.S1_at	3.754762	up	-1.92223	down	10.6759	up	1.953341	up
Os.12286.1.S1_at	-2.069851	down	-3.5677	down	-1.55583	down	-7.38461	down
OsAffx.16737.1.S1_at	-4.4518085	down	-1.53326	down	-3.60875	down	-6.82578	down
Os.21260.1.S1_at	-1.4612508	down	-3.97522	down	-1.38713	down	-5.80879	down
Os.55307.1.S1_at	5.6037254	up	-1.30445	down	7.186229	up	4.295845	up
Os.46836.1.S1_at	4.084226	up	1.591833	up	3.457804	up	6.501406	up

Os.48064.1.S1_at	3.8723402	up	5.634755	up	-1.02479	down	21.81969	up
Os.13972.1.S1_at	1.5293638	up	-1.35004	down	7.417645	up	1.132826	up
Os.50764.1.S1_at	2.3871603	up	2.17898	up	2.51908	up	5.201575	up
Os.7625.1.A1_s_at	4.4796495	up	1.849095	up	2.965276	up	8.283298	up
Os.15849.1.S1_s_at	1.2003267	up	6.280318	up	-1.14762	down	7.538433	up
Os.9238.1.A2_at	4.7694426	up	1.072247	up	5.094636	up	5.11402	up
Os.54555.1.S1_at	1.4141865	up	24.02192	up	-4.39798	down	33.97147	up
OsAffx.31649.1.S1_at	26.822182	up	-3.50535	down	19.08654	up	7.651776	up
Os.17390.1.S1_at	2.2928514	up	1.571459	up	3.453156	up	3.603121	up
Os.5736.1.S1_s_at	4.069424	up	1.382024	up	3.918579	up	5.624041	up
Os.50714.1.S1_x_at	4.5708847	up	-1.00494	down	5.428936	up	4.548409	up
OsAffx.12022.1.S1_s_	5.253057	up	-1.63517	down	8.796014	up	3.212543	up
Os.55984.1.S1_at	5.5213447	up	2.394125	up	2.242293	up	13.21879	up
Os.28435.1.S1_a_at	-2.8302896	down	-3.51067	down	-1.52714	down	-9.93622	down
Os.5599.1.S1_at	2.9269798	up	2.348245	up	2.280191	up	6.873266	up
Os.28435.5.S1_x_at	-2.5921612	down	-3.68206	down	-1.45073	down	-9.54449	down
Os.55096.1.S1_at	8.326646	up	-1.39177	down	7.42639	up	5.982775	up
Os.13835.2.S2_at	4.4739327	up	3.586926	up	1.484552	up	16.04767	up
Os.51926.1.S1_x_at	5.649185	up	1.406159	up	3.763373	up	7.943649	up
Os.8809.1.S1_s_at	7.717336	up	-1.31811	down	6.965872	up	5.854865	up
Os.17180.1.S1_at	-7.835975	down	1.336558	up	-7.06159	down	-5.8628	down
Os.18714.1.S1_a_at	-2.074482	down	-1.93155	down	-2.72889	down	-4.00696	down
Os.38234.1.S1_at	2.878483	up	2.066489	up	2.547803	up	5.948352	up
Os.12986.1.S1_at	7.2601213	up	-1.12641	down	5.927248	up	6.445373	up
Os.37621.3.S1_at	1.7700359	up	3.038538	up	1.728222	up	5.378321	up
Os.49329.1.S1_at	-2.5448909	down	-1.0486	down	-5.00222	down	-2.66857	down
Os.19519.1.S1_s_at	3.0722551	up	2.334937	up	2.24549	up	7.173521	up
Os.55611.1.S1_s_at	3.1087801	up	5.833366	up	-1.11647	down	18.13466	up
Os.14393.2.S1_at	3.25893	up	1.22355	up	4.260191	up	3.987465	up
OsAffx.13437.1.S1_at	6.2383533	up	1.150082	up	4.530253	up	7.174616	up
Os.34373.1.S1_at	6.189668	up	1.561266	up	3.332933	up	9.66372	up
Os.49112.1.S1_at	6.1814127	up	-1.1046	down	5.7221	up	5.596061	up
Os.9103.1.S1_at	5.321823	up	-1.39887	down	7.223869	up	3.804373	up
Os.52331.1.A1_at	5.647519	up	-1.10597	down	5.710713	up	5.106393	up
Os.40001.1.A1_at	1.6769168	up	-8.65722	down	1.676685	up	-5.16258	down
Os.50952.1.S1_at	4.919211	up	-1.08214	down	5.586457	up	4.545834	up
Os.16105.1.S1_at	2.4276989	up	1.775189	up	2.903137	up	4.309623	up
Os.7473.1.S1_at	3.4939897	up	1.097769	up	4.682251	up	3.835595	up
Os.39000.1.S1_at	13.640068	up	-6.4183	down	32.94259	up	2.125184	up
Os.2858.1.S1_at	8.113472	up	1.290365	up	3.974719	up	10.46934	up
Os.52280.1.S1_at	-4.8920655	down	-1.95011	down	-2.62814	down	-9.54007	down
Os.51694.1.S1_at	2.846142	up	1.235244	up	4.148487	up	3.515679	up
Os.49105.1.S1_at	4.683068	up	-1.72734	down	8.850779	up	2.711151	up
OsAffx.16071.1.S1_at	-6.421717	down	1.180294	up	-6.04476	down	-5.44078	down
Os.24834.1.S1_at	-4.5127816	down	1.144428	up	-5.86094	down	-3.94326	down
Os.23040.1.S1_at	5.273596	up	-1.10451	down	5.65489	up	4.774602	up
Os.12715.1.S1_at	4.9505124	up	1.641498	up	3.116911	up	8.126254	up

Os.34624.2.S1_s_at	-2.6132185	down	1.452526	up	-7.42654	down	-1.79909	down
Os.25146.1.A1_at	1.0441648	up	2.76911	up	1.845764	up	2.891407	up
OsAffx.30042.1.S1_at	5.8215857	up	-1.20358	down	6.134968	up	4.8369	up
Os.8306.1.S1_at	-2.8997333	down	-1.59068	down	-3.2043	down	-4.61255	down
Os.31636.1.S1_at	8.09283	up	1.355535	up	3.742876	up	10.97011	up
Os.6269.1.S1_at	2.324782	up	1.408931	up	3.590888	up	3.275457	up
Os.7130.2.S1_x_at	2.9228988	up	2.83244	up	1.785765	up	8.278936	up
Os.18241.2.A1_x_at	-7.648692	down	-1.14124	down	-4.43144	down	-8.729	down
Os.14885.1.S1_at	5.473226	up	1.252262	up	4.037563	up	6.853913	up
Os.23978.1.A1_at	2.0075557	up	1.848868	up	2.733639	up	3.711706	up
Os.14210.1.S1_at	-2.8680801	down	1.501492	up	-7.58037	down	-1.91015	down
Os.8118.1.S1_at	2.9899478	up	1.720057	up	2.934482	up	5.142881	up
Os.9659.1.S1_at	4.121521	up	-1.09827	down	5.54076	up	3.752749	up
Os.15191.1.S1_s_at	-2.3057618	down	-1.23507	down	-4.08347	down	-2.84777	down
Os.12381.1.S1_s_at	-1.110234	down	9.103099	up	-1.80771	down	8.199263	up
Os.5325.1.S1_at	74.11885	up	-32.5176	down	163.6829	up	2.279342	up
Os.43896.1.S1_at	3.6570263	up	2.10891	up	2.385522	up	7.712338	up
Os.17334.1.S1_at	1.3806441	up	1.879179	up	2.677036	up	2.594477	up
OsAffx.5738.1.S1_at	5.0789294	up	1.008345	up	4.985894	up	5.121312	up
Os.38346.1.S1_x_at	-5.0044355	down	1.000178	up	-5.0205	down	-5.00355	down
Os.7487.1.S1_at	1.0984471	up	-6.4515	down	1.287167	up	-5.87329	down
Os.159.1.S1_s_at	-3.086789	down	-3.00833	down	-1.66193	down	-9.2861	down
OsAffx.11866.1.S1_at	6.951272	up	-1.6542	down	8.269759	up	4.202197	up
Os.6314.1.S1_at	9.429281	up	-1.40464	down	7.019355	up	6.712932	up
Os.5940.1.S1_at	11.92466	up	-1.28842	down	6.437049	up	9.255271	up
Os.11053.1.S1_at	4.9520016	up	-3.79686	down	18.95375	up	1.304237	up
Os.51778.1.S1_at	5.829167	up	-2.78857	down	13.85115	up	2.090379	up
Os.9585.1.S1_s_at	2.2117214	up	3.544173	up	1.396601	up	7.838724	up
Os.4627.2.S1_at	2.8450255	up	1.662747	up	2.976262	up	4.730557	up
Os.6724.1.S1_at	4.532656	up	-1.32327	down	6.512291	up	3.425337	up
Os.1715.1.S1_at	4.2333694	up	1.366987	up	3.59814	up	5.786961	up
Os.28216.3.S1_x_at	1.525648	up	5.732348	up	-1.16907	down	8.745545	up
Os.49572.1.A1_at	4.645653	up	1.405456	up	3.486678	up	6.529262	up
Os.10933.1.S1_at	31.637526	up	-12.6437	down	61.85378	up	2.502235	up
Os.52987.1.S1_at	3.2114258	up	1.238565	up	3.945093	up	3.977559	up
Os.408.1.S1_a_at	-1.800967	down	-1.31805	down	-3.69171	down	-2.37376	down
Os.5044.1.S1_at	1.4484282	up	7.401426	up	-1.52327	down	10.72044	up
Os.4914.1.S1_s_at	1.206947	up	1.707792	up	2.844158	up	2.061215	up
Os.6372.1.S1_at	1.0988816	up	-2.45468	down	11.87452	up	-2.2338	down
Os.50134.2.S1_at	2.1726632	up	-1.43464	down	6.909813	up	1.514426	up
Os.14079.1.S1_at	6.5390234	up	11.6484	up	-2.41902	down	76.16915	up
Os.24275.1.S2_at	2.834658	up	-1.18438	down	5.698104	up	2.393371	up
Os.27846.1.S1_at	4.4777412	up	1.474542	up	3.260172	up	6.602618	up
Os.11575.2.S1_a_at	2.3679636	up	4.029258	up	1.192623	up	9.541135	up
Os.15917.1.S1_at	2.376446	up	3.86502	up	1.24276	up	9.18501	up
Os.28098.1.S1_at	16.99239	up	-5.57998	down	26.79597	up	3.045245	up
Os.35583.1.S1_at	1.9663734	up	1.245021	up	3.845847	up	2.448176	up

Os.941.1.S1_at	1.8461576	up	-2.86041	down	13.67107	up	-1.54938	down
Os.25185.1.S1_at	4.7313952	up	1.969979	up	2.424503	up	9.320747	up
Os.36776.1.S1_at	5.703733	up	1.474487	up	3.230347	up	8.410081	up
Os.51586.1.S1_at	-3.84884	down	-1.02198	down	-4.64782	down	-3.93346	down
Os.50957.1.S1_at	4.984306	up	-1.15813	down	5.471141	up	4.303736	up
Os.38045.1.S1_at	5.2432055	up	-1.1463	down	5.401145	up	4.574013	up
Os.7130.1.S1_at	3.5960855	up	2.5671	up	1.834694	up	9.231511	up
OsAffx.15376.1.S1_at	3.0335653	up	4.169752	up	1.126307	up	12.64922	up
Os.5599.1.S1_x_at	2.7612154	up	2.182198	up	2.146565	up	6.025518	up
Os.55197.1.S1_at	9.25753	up	-3.21868	down	15.07569	up	2.876185	up
OsAffx.3463.1.S1_at	-2.3417482	down	-1.59233	down	-2.93989	down	-3.72884	down
Os.18057.1.A1_at	1.7112932	up	2.162047	up	2.162111	up	3.699897	up
Os.51812.1.S1_at	2.2403908	up	1.048801	up	4.455681	up	2.349723	up
Os.49233.1.S1_at	-2.9996881	down	-1.26298	down	-3.6928	down	-3.78854	down
Os.7339.1.S1_at	-5.0839157	down	1.104749	up	-5.15066	down	-4.60187	down
Os.8895.1.S1_at	3.22895	up	-1.29555	down	6.034278	up	2.492338	up
OsAffx.31986.1.S1_at	7.451577	up	-2.58449	down	12.0365	up	2.883189	up
Os.5816.1.S1_at	-6.3119836	down	1.745136	up	-8.11526	down	-3.6169	down
Os.38485.1.S1_at	10.934113	up	1.373318	up	3.385244	up	15.01601	up
OsAffx.29599.2.S1_at	1.2911917	up	1.887806	up	2.458473	up	2.437519	up
Os.8700.1.S1_at	-3.3894277	down	-1.47492	down	-3.14652	down	-4.99912	down
Os.50333.1.S1_at	2.3942094	up	2.110053	up	2.193161	up	5.051909	up
Os.15573.1.A1_s_at	-3.25125	down	-1.11481	down	-4.14674	down	-3.62452	down
Os.11883.1.S1_at	3.2393396	up	1.888224	up	2.447491	up	6.116597	up
OsAffx.12099.1.S1_s_	1.9962423	up	1.396482	up	3.300975	up	2.787717	up
Os.55637.1.S1_at	2.3138013	up	2.421369	up	1.901887	up	5.602566	up
Os.39933.1.S1_x_at	-1.624477	down	-3.2863	down	-1.40123	down	-5.33851	down
Os.56279.1.S1_at	2.1756845	up	1.364004	up	3.373043	up	2.967641	up
Os.7751.1.S1_at	-1.4324743	down	-3.51465	down	-1.30848	down	-5.03464	down
Os.57149.1.S1_at	-3.4251795	down	1.047752	up	-4.81524	down	-3.26908	down
Os.5045.1.S1_at	4.0703535	up	8.323416	up	-1.81113	down	33.87924	up
Os.11639.1.S1_at	9.255243	up	-1.55464	down	7.141638	up	5.953305	up
Os.36969.1.S1_at	-6.4030967	down	-1.12291	down	-4.08907	down	-7.19008	down
Os.10918.1.S1_at	4.1860566	up	-1.21553	down	5.569597	up	3.443798	up
Os.45902.1.A1_x_at	-1.1439617	down	7.365033	up	-1.60956	down	6.438182	up
Os.38163.1.S1_at	3.9310515	up	-1.03914	down	4.751852	up	3.78298	up
Os.9615.1.S1_at	1.2040476	up	1.101364	up	4.150395	up	1.326094	up
Os.12416.1.S1_at	1.3641154	up	15.48479	up	-3.3894	down	21.12305	up
Os.7095.1.S1_at	3.6982265	up	3.637244	up	1.25497	up	13.45135	up
Os.46916.1.S1_at	1.1185269	up	1.855829	up	2.453406	up	2.075795	up
Os.5238.1.S1_at	-3.5807812	down	1.276979	up	-5.81397	down	-2.8041	down
Os.20206.1.S1_at	9.550171	up	-1.12108	down	5.098097	up	8.518712	up
Os.52568.1.S1_at	3.0880089	up	1.036861	up	4.375363	up	3.201835	up
Os.16067.1.S1_s_at	4.096918	up	-1.14166	down	5.179235	up	3.588549	up
OsAffx.28771.1.S1_at	1.9111322	up	9.506679	up	-2.09666	down	18.16852	up
Os.26955.1.S1_at	5.265873	up	-1.15814	down	5.239131	up	4.54682	up
Os.38021.1.S1_at	7.6612744	up	-1.72808	down	7.812646	up	4.433397	up

Os.6308.1.S1_at	7.3716936	up	-2.33403	down	10.53786	up	3.158354	up
Os.54967.1.S1_at	3.203933	up	1.50182	up	3.003207	up	4.811731	up
Os.12063.1.S1_at	2.3426445	up	3.179551	up	1.41841	up	7.448557	up
Os.55776.1.S1_x_at	2.1861248	up	3.838138	up	1.173384	up	8.390649	up
Os.10174.1.S1_at	-5.9320874	down	-2.60207	down	-1.72928	down	-15.4357	down
Os.26957.1.A1_a_at	-4.213285	down	1.26192	up	-5.67051	down	-3.33879	down
Os.9303.1.S1_at	-2.012932	down	-2.01964	down	-2.22241	down	-4.06539	down
Os.49631.2.S1_x_at	-3.4570844	down	1.220722	up	-5.47875	down	-2.832	down
Os.50762.1.S1_at	-2.0255523	down	-1.51483	down	-2.95901	down	-3.06836	down
Os.10244.1.S1_s_at	4.231532	up	1.062825	up	4.216321	up	4.497377	up
OsAffx.25929.1.S1_at	5.6388407	up	1.094274	up	4.085657	up	6.170436	up
Os.20163.1.S1_at	-4.383527	down	-1.14321	down	-3.90724	down	-5.01128	down
Os.4972.1.S1_at	6.171211	up	1.771407	up	2.516411	up	10.93172	up
OsAffx.26343.1.S1_x_	1.6497571	up	1.112433	up	4.003951	up	1.835245	up
Os.12385.1.S1_at	4.6256843	up	1.51375	up	2.941897	up	7.002131	up
OsAffx.21077.1.S1_at	-6.7914867	down	2.001096	up	-8.89141	down	-3.39388	down
Os.53377.1.S1_at	-6.1444125	down	1.751603	up	-7.78035	down	-3.50788	down
OsAffx.28133.1.S1_at	-4.685118	down	-1.37337	down	-3.22957	down	-6.43442	down
Os.7854.1.S2_at	3.2970462	up	1.160963	up	3.813923	up	3.827748	up
Os.25589.1.S1_at	-2.9613023	down	-1.10573	down	-4.00371	down	-3.2744	down
Os.15574.1.S1_at	2.6126277	up	2.290391	up	1.928472	up	5.98394	up
Os.19105.1.S1_at	-3.4782317	down	-3.63335	down	-1.21273	down	-12.6376	down
Os.12381.1.S1_x_at	-1.0161514	down	8.265244	up	-1.87587	down	8.13387	up
Os.55259.1.S1_at	9.682041	up	2.066921	up	2.131391	up	20.01202	up
Os.15281.1.S1_at	3.8379667	up	8.352738	up	-1.90275	down	32.05754	up
Os.6393.1.S1_at	-4.839507	down	1.092739	up	-4.78807	down	-4.42879	down
Os.45888.1.S1_at	3.9626164	up	-1.30003	down	5.692848	up	3.048095	up
Os.32602.1.S1_at	2.3975194	up	2.814755	up	1.555616	up	6.74843	up
Os.9654.1.S1_at	3.1667542	up	1.132458	up	3.861445	up	3.586215	up
Os.52175.1.S1_at	-5.6730595	down	1.233181	up	-5.39091	down	-4.60035	down
Os.53340.1.S1_at	-3.602009	down	-1.47245	down	-2.9685	down	-5.30377	down
Os.49763.2.S1_at	1.2444631	up	5.535307	up	-1.26754	down	6.888487	up
Os.47745.1.S1_at	-7.5560117	down	1.749985	up	-7.60461	down	-4.31776	down
Os.17036.1.S1_x_at	2.3541868	up	2.174567	up	1.994836	up	5.119337	up
Os.37969.1.S1_at	2.2417235	up	5.753457	up	-1.32641	down	12.89766	up
Os.49468.2.S1_x_at	3.759721	up	-1.53417	down	6.654574	up	2.450662	up
Os.15355.1.S1_x_at	-4.1566296	down	2.249032	up	-9.74531	down	-1.84819	down
Os.8019.1.S1_at	-2.7429867	down	-1.29468	down	-3.34687	down	-3.55128	down
Os.22486.1.A1_at	-8.647846	down	-1.64008	down	-2.64089	down	-14.1832	down
Os.27497.1.S1_at	-1.4194969	down	-3.84633	down	-1.12438	down	-5.45985	down
OsAffx.2569.1.S1_s_a	2.3142633	up	-1.26726	down	5.474507	up	1.826188	up
Os.1880.2.S1_a_at	-4.764052	down	1.310616	up	-5.65031	down	-3.63497	down
Os.22374.1.S2_a_at	3.2796135	up	1.178003	up	3.651676	up	3.863395	up
OsAffx.18575.1.S1_x_	2.0816336	up	3.213728	up	1.33677	up	6.689805	up
Os.53365.1.S1_at	4.594713	up	-1.57605	down	6.757617	up	2.915336	up
Os.27828.1.S1_a_at	-6.167245	down	1.006422	up	-4.31199	down	-6.12789	down
Os.50186.1.S1_at	5.427699	up	1.455726	up	2.940121	up	7.901242	up

Os.14921.1.S1_at	-1.0592185	down	12.95672	up	-3.02726	down	12.23234	up
Os.16214.1.S1_at	-1.2988293	down	-3.17243	down	-1.34906	down	-4.12045	down
Os.22949.1.S1_at	2.6732054	up	1.12418	up	3.805908	up	3.005164	up
Os.56918.1.S1_at	-1.3850043	down	-2.7993	down	-1.52665	down	-3.87704	down
Os.53880.1.S1_at	-2.2393854	down	-1.73442	down	-2.4636	down	-3.88404	down
Os.46633.1.A1_at	1.9613758	up	2.041136	up	2.091887	up	4.003434	up
Os.12012.1.S1_at	2.35116	up	2.829897	up	1.507556	up	6.65354	up
Os.47750.1.A1_at	1.2993033	up	1.307167	up	3.26279	up	1.698406	up
Os.55366.1.S1_x_at	3.4257758	up	1.057069	up	4.031731	up	3.621282	up
OsAffx.27267.1.S1_at	-1.2054008	down	1.162172	up	-4.9482	down	-1.0372	down
Os.57443.1.S1_x_at	-2.046536	down	-4.12721	down	-1.03084	down	-8.44649	down
Os.53114.1.S1_at	2.676782	up	1.730869	up	2.454098	up	4.633159	up
Os.14313.2.S1_s_at	1.6414756	up	4.053012	up	1.044134	up	6.652921	up
Os.6623.1.S1_at	3.0212808	up	1.987649	up	2.128937	up	6.005244	up
Os.23964.2.S1_x_at	-4.9986067	down	1.034953	up	-4.37784	down	-4.82979	down
Os.34523.1.S1_at	5.4194145	up	1.12746	up	3.750101	up	6.110175	up
Os.47941.1.A1_at	2.087266	up	1.386149	up	3.049901	up	2.893262	up
Os.34139.1.S1_at	12.798845	up	-1.25301	down	5.291887	up	10.21452	up
Os.54997.1.S1_at	8.799745	up	-5.99605	down	25.31942	up	1.46759	up
Os.16067.1.S1_at	2.7594345	up	1.051458	up	4.013411	up	2.901429	up
OsAffx.26317.1.S2_at	2.596757	up	-7.49447	down	31.58209	up	-2.88609	down
Os.10617.1.S1_at	-2.6760645	down	-1.22378	down	-3.4429	down	-3.27491	down
Os.46800.1.S2_a_at	2.7860942	up	3.512825	up	1.198734	up	9.787059	up
Os.4715.1.S1_at	5.014559	up	-1.564	down	6.582056	up	3.206241	up
Os.19586.1.S1_at	-2.181668	down	-1.7225	down	-2.44097	down	-3.75792	down
Os.15874.1.A1_at	10.238897	up	-1.65091	down	6.939934	up	6.201956	up
Os.5812.1.S1_at	-2.7193131	down	-1.95367	down	-2.15075	down	-5.31264	down
Os.27788.1.S1_at	-6.1399527	down	-1.16579	down	-3.60356	down	-7.15792	down
Os.10416.1.S1_at	-1.4188371	down	12.83815	up	-3.05629	down	9.048362	up
Os.15692.1.S1_at	1.115824	up	-1.73864	down	7.289079	up	-1.55817	down
Os.14181.1.S1_at	5.7641416	up	-1.20726	down	5.052679	up	4.774571	up
OsAffx.27220.1.S1_at	3.183111	up	1.487712	up	2.80598	up	4.73555	up
Os.17294.1.S1_a_at	-6.745804	down	-1.21676	down	-3.42946	down	-8.20799	down
Os.27307.1.S1_at	-5.2420244	down	-1.42821	down	-2.91993	down	-7.48673	down
Os.6354.1.S1_at	1.1300007	up	3.16812	up	1.31613	up	3.579978	up
Os.55332.1.S1_at	1.6673934	up	-6.65888	down	27.74936	up	-3.99358	down
Os.19061.1.S1_s_at	-2.9688065	down	-1.12979	down	-3.67819	down	-3.35413	down
Os.49087.1.A1_at	1.8874478	up	1.390638	up	2.981915	up	2.624757	up
Os.5682.1.S1_at	-1.5553519	down	11.02058	up	-2.65854	down	7.085589	up
Os.35013.1.S1_at	9.5530615	up	-1.88802	down	7.822186	up	5.05982	up
Os.8390.1.S1_s_at	-6.3307176	down	3.10438	up	-12.8584	down	-2.03929	down
OsAffx.21941.1.S1_s_	8.595069	up	2.1781	up	1.901203	up	18.72092	up
Os.54341.1.S1_at	5.994095	up	-1.45188	down	6.010899	up	4.12852	up
OsAffx.26533.1.S1_at	1.6785885	up	14.57876	up	-3.5227	down	24.47174	up
Os.51606.1.S1_at	3.050311	up	-1.07725	down	4.457937	up	2.831559	up
Os.15607.1.S1_at	-6.523233	down	-1.45445	down	-2.84337	down	-9.48774	down
Os.11906.1.S1_at	8.849079	up	2.418294	up	1.708829	up	21.39968	up

Os.8850.1.S1_x_at	-3.0138037	down	1.212343	up	-5.00725	down	-2.48593	down
Os.17356.1.A1_a_at	-3.1614995	down	-2.48563	down	-1.66101	down	-7.85831	down
Os.27423.1.S1_x_at	-3.4480603	down	-1.35602	down	-3.04207	down	-4.67564	down
Os.10982.1.S1_at	2.7247257	up	2.11704	up	1.945118	up	5.768353	up
Os.12112.1.S1_at	6.0517344	up	-2.52086	down	10.38037	up	2.400666	up
Os.55625.1.S1_at	3.0653076	up	1.977747	up	2.081477	up	6.062403	up
Os.17522.1.S1_s_at	-4.713363	down	-1.33743	down	-3.07642	down	-6.30379	down
OsAffx.23225.1.S1_at	5.968426	up	-1.60287	down	6.585555	up	3.723578	up
Os.37865.1.S1_at	6.251903	up	1.761605	up	2.331052	up	11.01338	up
OsAffx.24157.1.S1_s_	1.4387367	up	1.446256	up	2.839318	up	2.080782	up
Os.55604.1.S1_at	2.041696	up	-1.32412	down	5.436161	up	1.541924	up
Os.11089.1.S1_at	13.120171	up	-2.35144	down	9.651564	up	5.579629	up
OsAffx.30138.1.S1_at	5.95969	up	1.029826	up	3.980034	up	6.137443	up
Os.19385.1.S1_at	4.5303	up	-1.48432	down	6.077397	up	3.0521	up
Os.16866.1.S1_at	-3.6873157	down	-1.40794	down	-2.90641	down	-5.19153	down
OsAffx.30275.1.S1_at	1.7054662	up	1.078448	up	3.792233	up	1.839256	up
OsAffx.13348.1.S1_s_	1.6282078	up	6.557727	up	-1.60361	down	10.67734	up
Os.9286.1.S1_at	2.5712826	up	1.374441	up	2.973774	up	3.534077	up
Os.16897.1.S1_at	-1.7728586	down	1.014005	up	-4.14313	down	-1.74837	down
Os.13954.1.S1_x_at	11.352661	up	1.447707	up	2.812116	up	16.43533	up
Os.12705.2.S1_x_at	5.7696733	up	-1.35871	down	5.525297	up	4.246444	up
Os.8999.2.S1_at	3.59039	up	-1.41685	down	5.754314	up	2.534071	up
Os.19061.2.S1_at	-2.1452265	down	-1.82129	down	-2.2276	down	-3.90708	down
Os.32366.1.S1_at	-1.2707579	down	11.29911	up	-2.78559	down	8.891633	up
Os.24786.1.S1_s_at	1.9729137	up	15.78855	up	-3.90451	down	31.14945	up
OsAffx.1916.1.S1_s_a	-3.5290022	down	1.021336	up	-4.12375	down	-3.45528	down
Os.3766.1.S1_at	6.0222006	up	1.537074	up	2.626098	up	9.256571	up
Os.19493.1.S1_at	-2.8979669	down	1.02596	up	-4.13974	down	-2.82464	down
Os.43375.1.S1_at	5.3234854	up	-1.32228	down	5.333911	up	4.025986	up
Os.17777.2.S1_x_at	2.28777	up	1.778907	up	2.266539	up	4.069729	up
Os.51928.1.S1_at	3.2135735	up	-1.10335	down	4.437795	up	2.91255	up
Os.9930.1.S1_at	-2.4511523	down	-1.62245	down	-2.47516	down	-3.97688	down
Os.7861.1.S1_at	-1.3821113	down	2.340344	up	1.7159	up	1.693311	up
Os.46776.1.S1_s_at	1.6921731	up	3.067248	up	1.306992	up	5.190314	up
Os.50342.1.S1_at	2.7055888	up	2.347998	up	1.70707	up	6.352717	up
Os.12134.1.S1_at	7.210563	up	-1.79379	down	7.188174	up	4.019738	up
Os.7738.1.S1_at	4.8508453	up	-1.59935	down	6.385328	up	3.033006	up
OsAffx.5584.1.S1_at	5.3993726	up	1.461357	up	2.731375	up	7.89041	up
Os.7947.1.S1_a_at	-3.2867672	down	-1.28659	down	-3.09538	down	-4.22871	down
Os.7225.1.S1_at	5.038578	up	-1.89831	down	7.552192	up	2.654249	up
Os.27323.1.S1_at	6.6151495	up	-1.03889	down	4.132257	up	6.367524	up
Os.2019.1.S1_at	2.5329409	up	1.631857	up	2.435633	up	4.133398	up
OsAffx.24782.1.S1_s_	2.4792378	up	5.688464	up	-1.43129	down	14.10306	up
Os.57373.1.S1_at	2.990386	up	-1.66643	down	6.621251	up	1.794485	up
Os.27228.2.S1_at	-3.7655299	down	-1.25426	down	-3.1623	down	-4.72296	down
Os.2367.1.S1_at	1.7608707	up	-1.39292	down	5.523145	up	1.264162	up
Os.11675.1.A1_at	-3.3475587	down	-1.94453	down	-2.03513	down	-6.50942	down

OsAffx.27508.82.S1_	-4.5131073	down	-1.18005	down	-3.34971	down	-5.3257	down
Os.26388.3.S1_x_at	5.334575	up	1.679371	up	2.353077	up	8.958731	up
Os.2245.1.S1_at	2.3610644	up	2.083493	up	1.895762	up	4.919261	up
OsAffx.14615.1.S1_at	-2.2572968	down	-1.89869	down	-2.07788	down	-4.28591	down
OsAffx.12887.2.S1_at	4.9783974	up	-9.61442	down	37.84934	up	-1.93123	down
Os.18760.1.A1_at	-3.3447175	down	-1.28623	down	-3.05836	down	-4.30209	down
Os.9592.1.S1_at	3.617781	up	2.221508	up	1.76984	up	8.036927	up
OsAffx.12887.2.S1_s_	7.881046	up	-10.7218	down	42.10862	up	-1.36045	down
Os.50134.1.S1_at	7.8406434	up	-5.73293	down	22.50431	up	1.36765	up
Os.55986.1.S1_x_at	2.6060538	up	1.652443	up	2.373959	up	4.306354	up
Os.53103.1.S1_x_at	-5.8547854	down	-1.07772	down	-3.63668	down	-6.30981	down
Os.12251.1.S1_at	3.7445898	up	-3.13436	down	12.27541	up	1.194689	up
OsAffx.17474.1.S1_s_	-2.3327432	down	-1.5967	down	-2.45098	down	-3.7247	down
Os.32177.1.S1_at	2.8534343	up	1.054925	up	3.705845	up	3.01016	up
Os.38994.1.A1_at	2.9351654	up	1.160924	up	3.36714	up	3.407504	up
Os.11305.1.S1_at	11.195685	up	-2.65604	down	10.37032	up	4.215175	up
OsAffx.29461.1.S1_at	10.258111	up	1.234869	up	3.160244	up	12.66743	up
Os.1311.1.S1_at	5.2228556	up	-1.61645	down	6.305361	up	3.231068	up
Os.2423.1.S1_at	1.6954626	up	1.622508	up	2.403998	up	2.750902	up
Os.36194.1.S1_at	1.8557692	up	1.281112	up	3.044375	up	2.377448	up
Os.50281.1.S1_at	2.5101018	up	1.976206	up	1.972323	up	4.960478	up
Os.5028.1.S1_at	2.3828478	up	3.642775	up	1.069787	up	8.680177	up
Os.45902.1.A1_at	-1.0074168	down	7.167253	up	-1.84078	down	7.114486	up
Os.14849.1.S1_a_at	-2.8157403	down	1.184941	up	-4.6098	down	-2.37627	down
Os.11323.1.S1_at	3.6071918	up	1.297237	up	2.998276	up	4.679383	up
Os.6629.1.S1_at	-2.67648	down	-1.64	down	-2.3713	down	-4.38943	down
Os.5186.1.S1_at	3.0782819	up	3.173753	up	1.224061	up	9.769707	up
Os.18955.1.S1_at	7.2593694	up	-2.01302	down	7.819355	up	3.606214	up
OsAffx.2045.2.S1_at	2.7713406	up	-1.45477	down	5.642547	up	1.905001	up
Os.8821.1.S1_at	1.7897756	up	-1.06352	down	4.12308	up	1.682874	up
Os.23846.1.A1_s_at	2.3233259	up	1.202416	up	3.221057	up	2.793605	up
Os.27592.1.A1_at	1.9929682	up	-1.2078	down	4.675945	up	1.650075	up
Os.46881.1.S1_at	-2.7689428	down	-1.64624	down	-2.34622	down	-4.55833	down
Os.55991.1.S1_at	1.5296506	up	1.716578	up	2.243831	up	2.625764	up
Os.26441.1.S1_s_at	1.4703975	up	1.064034	up	3.618159	up	1.564552	up
Os.49804.1.S1_at	-4.430128	down	-1.05994	down	-3.63162	down	-4.69567	down
Os.6766.1.S1_s_at	1.9071212	up	1.513639	up	2.538532	up	2.886693	up
Os.50108.1.S1_at	2.363495	up	2.221415	up	1.728975	up	5.250303	up
Os.27177.1.S1_at	1.7675787	up	3.223794	up	1.191325	up	5.698309	up
Os.26979.1.S1_x_at	-11.097973	down	1.980822	up	-7.58688	down	-5.60271	down
Os.54340.1.S1_x_at	-3.3450282	down	1.026885	up	-3.93239	down	-3.25745	down
Os.10695.1.S1_s_at	2.6442468	up	2.121911	up	1.804612	up	5.610855	up
Os.12348.4.S1_at	3.0342803	up	-1.21604	down	4.650967	up	2.495219	up
Os.5213.1.S1_at	-4.277789	down	1.010804	up	-3.86379	down	-4.23207	down
Os.39981.1.S1_at	2.875371	up	1.190569	up	3.208196	up	3.423328	up
Os.57382.1.S1_at	1.5677392	up	1.561019	up	2.446205	up	2.44727	up
Os.7800.1.S1_at	2.8240778	up	1.256684	up	3.028658	up	3.548973	up

Os.27724.2.A1_a_at	2.6874402	up	1.282798	up	2.965722	up	3.447442	up
Os.12165.1.S1_at	3.246569	up	1.807819	up	2.103709	up	5.869208	up
Os.428.1.S1_at	-2.3482244	down	1.622471	up	-6.16116	down	-1.44731	down
Os.2364.1.S1_at	-3.8304756	down	1.014669	up	-3.85231	down	-3.7751	down
Os.8561.1.S1_at	1.9671235	up	-1.35865	down	5.157498	up	1.447856	up
Os.49159.1.A1_x_at	-3.08798	down	-1.24708	down	-3.0408	down	-3.85097	down
Os.48017.1.S1_x_at	2.69851	up	1.039787	up	3.642755	up	2.805876	up
Os.54867.1.S1_at	2.4371579	up	2.204974	up	1.715243	up	5.373869	up
Os.11474.1.S1_at	1.8886472	up	2.631767	up	1.436286	up	4.970479	up
Os.12889.1.S1_x_at	-2.6624084	down	-1.20426	down	-3.13769	down	-3.20623	down
Os.51127.1.S1_a_at	-2.2559693	down	1.649071	up	-6.22877	down	-1.36802	down
Os.16761.1.S2_at	1.803763	up	1.285371	up	2.937611	up	2.318505	up
Os.11296.1.S1_a_at	-4.768379	down	1.499453	up	-5.65691	down	-3.18008	down
Os.14614.1.A1_at	-6.4732456	down	1.074108	up	-4.0521	down	-6.02663	down
Os.53133.1.S1_at	-9.531909	down	1.487014	up	-5.60933	down	-6.4101	down
Os.14592.1.S1_at	4.076866	up	-1.3689	down	5.16212	up	2.978213	up
Os.18922.2.S1_at	2.3638535	up	1.897564	up	1.985325	up	4.485563	up
Os.6330.1.S1_at	11.960545	up	-29.0658	down	109.4749	up	-2.43014	down
Os.6539.1.S1_at	9.840713	up	1.144982	up	3.287317	up	11.26744	up
Os.11205.1.S1_at	3.4846587	up	1.050254	up	3.582418	up	3.659776	up
Os.5557.1.S1_at	2.6144803	up	2.334336	up	1.610709	up	6.103075	up
Os.36995.1.S1_at	1.2184951	up	-1.27026	down	4.773978	up	-1.04248	down
Os.37093.1.S1_at	-1.0686686	down	2.37055	up	1.584575	up	2.218228	up
Os.55094.1.S1_at	-2.1184509	down	-2.1149	down	-1.77606	down	-4.48031	down
Os.21803.1.S1_at	-6.3036003	down	-1.31535	down	-2.85332	down	-8.29147	down
Os.55527.1.S1_at	1.3007898	up	-1.35028	down	5.066758	up	-1.03804	down
Os.11477.1.S1_at	8.233699	up	1.344639	up	2.789088	up	11.07136	up
OsAffx.26321.1.S1_at	-4.9964795	down	1.238744	up	-4.64239	down	-4.0335	down
Os.52490.1.S1_at	-2.4659033	down	-1.89137	down	-1.9811	down	-4.66393	down
Os.11820.1.S2_at	-7.3189387	down	1.511632	up	-5.66286	down	-4.84175	down
Os.4755.1.S1_at	1.9494119	up	-1.20516	down	4.512065	up	1.617555	up
OsAffx.14053.1.S1_at	-5.200296	down	1.307715	up	-4.89128	down	-3.97663	down
Os.4799.2.S1_at	2.82469	up	1.07963	up	3.456008	up	3.049621	up
Os.49897.1.S1_at	1.4050105	up	1.480381	up	2.520043	up	2.07995	up
Os.36278.1.S1_x_at	-5.3081856	down	1.937784	up	-7.22133	down	-2.73931	down
Os.32153.1.S1_at	3.670118	up	1.398107	up	2.662981	up	5.131217	up
Os.49583.1.S1_at	1.1290559	up	2.951253	up	1.261437	up	3.332129	up
Os.52508.1.S1_at	2.1234376	up	-1.36525	down	5.072247	up	1.555342	up
Os.53117.1.S1_x_at	6.3675146	up	-1.93068	down	7.153427	up	3.298073	up
Os.7983.1.S1_at	2.864873	up	2.284324	up	1.621377	up	6.544297	up
Os.51795.1.S1_at	1.8624247	up	1.430726	up	2.586382	up	2.66462	up
Os.55837.1.S1_x_at	1.9725597	up	-1.28323	down	4.746084	up	1.537181	up
Os.9842.1.S1_at	-2.9015331	down	1.115229	up	-4.12282	down	-2.60174	down
Os.26151.1.S1_s_at	-4.102174	down	-1.13138	down	-3.26074	down	-4.64113	down
Os.46578.1.S1_at	-1.5331874	down	2.734139	up	1.348754	up	1.783304	up
OsAffx.12920.1.S1_at	17.4938	up	-3.41856	down	12.59781	up	5.117295	up
Os.38022.1.S1_at	1.6740804	up	1.413261	up	2.606292	up	2.365912	up

OsAffx.16770.1.S1_x_	4.836433	up	-1.07139	down	3.945875	up	4.514164	up
Os.10099.1.S1_at	4.177491	up	1.654944	up	2.223035	up	6.913514	up
Os.11897.1.S1_at	-4.6478453	down	-5.34009	down	1.454956	up	-24.8199	down
Os.9022.1.S1_at	6.732118	up	-1.3236	down	4.855732	up	5.086237	up
Os.53133.1.S1_x_at	-7.8139296	down	1.321528	up	-4.84604	down	-5.9128	down
Os.10388.1.S1_at	-2.1550467	down	-1.17269	down	-3.12612	down	-2.52721	down
Os.20504.1.A1_at	9.978117	up	-2.09435	down	7.672082	up	4.764302	up
Os.49607.1.S1_at	2.9473507	up	1.114886	up	3.284531	up	3.28596	up
OsAffx.24066.1.S1_x_	4.189094	up	-1.08727	down	3.980325	up	3.852866	up
Os.6618.1.S1_at	5.9739227	up	-8.20969	down	30.0527	up	-1.37425	down
Os.3241.1.S1_at	-6.6296144	down	1.014458	up	-3.71265	down	-6.53513	down
OsAffx.3672.1.S1_at	-6.813062	down	1.577325	up	-5.7655	down	-4.31938	down
Os.55407.1.S1_at	16.08725	up	-1.27878	down	4.672591	up	12.58018	up
OsAffx.11366.1.S1_at	3.7270405	up	-1.2352	down	4.504681	up	3.017363	up
Os.30696.1.S1_at	3.233746	up	1.590477	up	2.291439	up	5.143199	up
Os.17334.1.S1_s_at	1.0958741	up	1.503318	up	2.421731	up	1.647448	up
Os.32292.1.S1_at	1.6151997	up	-1.31283	down	4.772993	up	1.230315	up
Os.8982.1.S1_at	6.374432	up	-4.35467	down	15.82501	up	1.463815	up
Os.47971.1.A1_at	3.5240176	up	1.50124	up	2.420556	up	5.290398	up
Os.29959.1.S1_at	6.9622836	up	-1.93707	down	7.032713	up	3.594233	up
Os.55263.1.S1_at	6.8381987	up	-1.75161	down	6.355663	up	3.903947	up
Os.11444.1.S1_at	3.2225966	up	1.269769	up	2.857451	up	4.091954	up
Os.52833.1.S1_at	-5.805417	down	-1.0544	down	-3.43901	down	-6.12126	down
Os.2250.1.S1_a_at	1.1587597	up	-1.73796	down	6.297319	up	-1.49985	down
Os.28531.1.S1_at	1.5789822	up	2.464163	up	1.469073	up	3.890869	up
Os.51227.1.S1_s_at	-1.3439853	down	1.98686	up	-7.18301	down	1.478335	up
OsAffx.26492.1.S1_s_	2.0292556	up	7.03223	up	-1.9468	down	14.27019	up
Os.4189.1.S1_at	-5.94244	down	1.491049	up	-5.38532	down	-3.98541	down
Os.14104.1.A1_a_at	-2.7536073	down	-1.20145	down	-3.00596	down	-3.30832	down
Os.51285.1.S1_at	-3.559919	down	1.092926	up	-3.94607	down	-3.25724	down
Os.19061.1.S1_at	-3.4862897	down	1.360942	up	-4.91362	down	-2.56167	down
Os.52607.1.S1_at	3.7242177	up	2.028501	up	1.778358	up	7.55458	up
OsAffx.2361.1.S1_x_a	11.21968	up	1.031944	up	3.495257	up	11.57808	up
Os.10959.1.S2_at	3.29447	up	-1.6569	down	5.974466	up	1.988338	up
OsAffx.21635.2.S1_at	-5.322606	down	1.422771	up	-5.11652	down	-3.74101	down
Os.52594.1.S1_at	-6.396623	down	-1.52171	down	-2.36179	down	-9.73377	down
Os.2117.1.S1_s_at	2.852242	up	1.346197	up	2.668223	up	3.83968	up
Os.15138.1.S1_at	1.5747656	up	2.596653	up	1.383265	up	4.089119	up
Os.53936.1.S1_at	12.28217	up	-1.27048	down	4.560429	up	9.667324	up
Os.48911.1.A1_s_at	-3.6134188	down	1.042654	up	-3.73262	down	-3.4656	down
Os.55534.1.A1_at	-2.6431441	down	-1.40095	down	-2.55532	down	-3.70292	down
Os.56964.1.S1_at	2.211455	up	1.760138	up	2.032245	up	3.892466	up
OsAffx.24831.1.S1_s_	2.8792446	up	-1.21612	down	4.349287	up	2.367558	up
Os.57343.1.S1_at	8.927647	up	-1.07083	down	3.828152	up	8.337136	up
Os.37376.2.S1_x_at	8.470543	up	1.015722	up	3.518958	up	8.603714	up
Os.5638.1.S1_at	1.5343411	up	1.341906	up	2.661727	up	2.058941	up
Os.53724.1.S1_at	1.549859	up	1.116669	up	3.198237	up	1.73068	up

Os.9276.1.S1_s_at	-5.0546627	down	-1.24606	down	-2.86601	down	-6.2984	down
Os.53494.1.S1_x_at	-3.4357245	down	1.691487	up	-6.03637	down	-2.03119	down
Os.7947.1.S1_x_at	-3.1594136	down	-1.15784	down	-3.08185	down	-3.65809	down
Os.5318.1.S1_a_at	-1.4344845	down	-2.23481	down	-1.59642	down	-3.2058	down
Os.12699.1.S1_at	-3.5683055	down	-1.21049	down	-2.94374	down	-4.31939	down
Os.14042.1.S1_at	2.7088044	up	1.162494	up	3.064852	up	3.148969	up
Os.8576.1.S1_at	-1.8604372	down	-1.06801	down	-3.33533	down	-1.98696	down
Os.46819.1.S1_at	1.5572711	up	2.131746	up	1.669049	up	3.319706	up
Os.17902.1.S1_at	-4.4500036	down	-1.2466	down	-2.84977	down	-5.54736	down
OsAffx.14406.1.S1_at	1.1857604	up	1.343773	up	2.643554	up	1.593393	up
Os.13497.1.S1_at	3.5497093	up	1.201601	up	2.955011	up	4.265336	up
Os.4971.1.S1_at	-3.061667	down	-1.07017	down	-3.31704	down	-3.27651	down
Os.6310.1.S1_at	2.3245964	up	1.2899	up	2.751262	up	2.998498	up
Os.11820.1.S1_at	-8.557731	down	2.450901	up	-8.69477	down	-3.49167	down
Os.49865.1.S1_at	2.6671505	up	1.733936	up	2.04425	up	4.624668	up
Os.15428.1.S1_at	-2.0452487	down	1.33867	up	-4.74038	down	-1.52782	down
Os.49667.1.S1_at	-1.785306	down	2.244856	up	1.574507	up	1.257407	up
OsAffx.10944.1.S1_at	1.5855706	up	2.797466	up	1.261144	up	4.43558	up
Os.6450.1.S1_at	7.722781	up	5.171562	up	-1.4663	down	39.93883	up
Os.49746.1.S1_at	-1.77672	down	-2.59824	down	-1.35668	down	-4.61635	down
Os.55227.1.S1_at	5.9914722	up	-4.73536	down	16.66121	up	1.265262	up
Os.12714.1.S1_at	1.7069263	up	1.389014	up	2.533006	up	2.370944	up
Os.12393.1.S2_a_at	4.5508604	up	-1.3574	down	4.773424	up	3.352624	up
Os.8965.1.S1_at	10.235416	up	-2.87736	down	10.10623	up	3.55722	up
Os.19951.1.S1_at	2.4172986	up	1.363466	up	2.575964	up	3.295904	up
Os.7750.1.S1_at	3.5824702	up	1.069513	up	3.281178	up	3.831497	up
Os.52573.1.S1_at	-6.659259	down	1.553983	up	-5.45221	down	-4.28529	down
Os.2915.1.S1_at	3.9052625	up	1.09131	up	3.214157	up	4.261853	up
OsAffx.32313.1.A1_at	-1.0878844	down	-2.0705	down	-1.69379	down	-2.25246	down
Os.51775.1.S1_x_at	18.414785	up	-10.8419	down	37.97251	up	1.69849	up
OsAffx.28760.2.S1_at	1.8243448	up	2.28373	up	1.533528	up	4.166311	up
Os.52915.1.S1_at	-2.5565002	down	1.093956	up	-3.8306	down	-2.33693	down
Os.37699.3.S1_x_at	5.3329353	up	-1.23597	down	4.323567	up	4.314775	up
Os.3428.1.S1_x_at	2.730556	up	1.129146	up	3.097968	up	3.083198	up
Os.33238.2.S1_at	5.1488643	up	1.047676	up	3.334332	up	5.394339	up
Os.56140.1.S1_x_at	1.0316554	up	1.504234	up	2.321306	up	1.551851	up
Os.32086.3.S1_x_at	4.7372327	up	-1.3539	down	4.724996	up	3.498953	up
Os.53103.1.S1_at	-5.1454263	down	-1.03911	down	-3.35778	down	-5.34668	down
Os.10654.1.S1_s_at	2.0853667	up	1.658418	up	2.101381	up	3.45841	up
Os.29825.1.S1_a_at	-2.3534021	down	-1.87881	down	-1.85461	down	-4.42159	down
Os.20239.1.S1_at	-5.129811	down	1.122606	up	-3.91135	down	-4.56956	down
Os.32086.3.S1_s_at	6.9272823	up	-1.69403	down	5.89549	up	4.089239	up
OsAffx.23595.1.S1_at	3.6199713	up	1.138358	up	3.055288	up	4.120822	up
Os.4613.1.S1_at	3.0049675	up	1.679984	up	2.069133	up	5.048297	up
Os.48013.1.A1_at	1.5682371	up	-1.32927	down	4.616178	up	1.17977	up
AFFX-Os_28SrRNA_at	1.3462121	up	-2.96288	down	-1.17012	down	-2.2009	down
Os.7931.1.S1_a_at	1.3785846	up	1.625619	up	2.129078	up	2.241053	up

Os.13835.2.S3_a_at	3.1504264	up	4.40655	up	-1.27452	down	13.88251	up
Os.19375.1.S1_at	2.638759	up	3.255906	up	1.061871	up	8.591551	up
Os.38169.1.S1_a_at	1.7784846	up	3.275195	up	1.054952	up	5.824884	up
Os.7348.1.S1_at	2.7370145	up	1.782249	up	1.935368	up	4.878041	up
Os.10675.1.A1_at	-2.421986	down	-1.03216	down	-3.3399	down	-2.49987	down
OsAffx.1929.1.S1_at	-3.7817895	down	1.06764	up	-3.68017	down	-3.5422	down
Os.23105.1.A1_at	-1.5781274	down	-1.82601	down	-1.88755	down	-2.88168	down
Os.16163.1.S1_at	-1.0257611	down	-3.48489	down	12.00267	up	-3.57466	down
Os.26545.1.A1_at	3.370811	up	1.039622	up	3.305956	up	3.504367	up
Os.27860.1.A1_at	1.9410719	up	1.632344	up	2.104966	up	3.168498	up
Os.51231.1.S1_at	4.5722294	up	1.157236	up	2.968361	up	5.291146	up
Os.48260.1.S1_at	-1.1515701	down	1.290179	up	2.662283	up	1.120365	up
OsAffx.32220.1.A1_s_	-1.1871804	down	-1.58522	down	-2.16634	down	-1.88194	down
Os.17449.1.A1_at	-4.8581095	down	1.123076	up	-3.85418	down	-4.32572	down
Os.51775.1.S1_at	15.372837	up	-9.16877	down	31.44166	up	1.676652	up
Os.4655.1.S1_at	-2.8311028	down	1.23818	up	-4.2379	down	-2.2865	down
Os.49764.1.S1_at	7.5641665	up	-5.84839	down	20.01221	up	1.293375	up
Os.53322.1.S1_at	3.7288642	up	1.071217	up	3.193553	up	3.994422	up
Os.10672.1.S1_at	3.2501042	up	-1.18047	down	4.036169	up	2.753229	up
Os.55366.1.S1_at	3.4043684	up	-1.13964	down	3.89489	up	2.987232	up
Os.2019.2.S1_x_at	3.0747752	up	1.404051	up	2.433791	up	4.317141	up
Os.24906.1.S1_at	8.550242	up	-1.69812	down	5.799881	up	5.035134	up
Os.8979.1.S1_at	-3.3216043	down	-1.05754	down	-3.22953	down	-3.51273	down
Os.52728.1.S1_at	3.969398	up	2.347107	up	1.454872	up	9.3166	up
OsAffx.11956.1.S1_at	-1.0106243	down	-2.33679	down	-1.46052	down	-2.36162	down
Os.15198.1.S1_at	-3.8492537	down	1.153701	up	-3.93496	down	-3.33644	down
Os.22577.2.S1_x_at	-1.8437947	down	1.365656	up	-4.65518	down	-1.35012	down
OsAffx.20681.1.S1_at	-1.5894518	down	-1.0004	down	-3.40625	down	-1.59009	down
OsAffx.15770.1.S1_at	1.3619139	up	2.118188	up	1.607705	up	2.88479	up
Os.14393.1.S1_x_at	1.8737918	up	1.472449	up	2.311639	up	2.759064	up
Os.12230.1.S1_a_at	3.208819	up	1.231414	up	2.763713	up	3.951385	up
Os.7838.1.S1_at	2.862664	up	-1.06581	down	3.626274	up	2.6859	up
Os.14974.1.S1_at	1.121401	up	1.670544	up	2.034915	up	1.87335	up
Os.29800.2.S1_at	-2.2733972	down	-1.24061	down	-2.74007	down	-2.82041	down
Os.7116.1.S1_at	-1.0352975	down	5.841931	up	-1.71976	down	5.642757	up
Os.42285.1.S1_x_at	-3.4304206	down	1.995986	up	-6.7784	down	-1.71866	down
Os.17259.1.S1_at	-4.6352043	down	-1.48056	down	-2.28961	down	-6.86269	down
Os.24017.1.S1_at	2.8576155	up	1.186912	up	2.854526	up	3.391737	up
OsAffx.2489.1.S1_s_a	-2.9401793	down	-1.33534	down	-2.53708	down	-3.92614	down
Os.31293.1.S1_at	2.6887062	up	1.059408	up	3.197761	up	2.848437	up
Os.52678.1.S1_at	1.294716	up	-2.75003	down	-1.23169	down	-2.12404	down
Os.31858.1.S1_at	2.0602665	up	2.067417	up	1.636616	up	4.25943	up
Os.11419.1.S1_at	4.4673967	up	-2.76116	down	9.340283	up	1.617942	up
Os.37969.2.S1_x_at	2.200903	up	4.526057	up	-1.33835	down	9.961411	up
OsAffx.1957.1.S1_at	5.995472	up	-1.95238	down	6.595957	up	3.070849	up
Os.14791.1.S1_at	1.3624103	up	1.575765	up	2.139673	up	2.146838	up
Os.24106.3.S1_at	2.2640524	up	1.156813	up	2.90818	up	2.619086	up

Os.15386.1.S1_at	-5.279275	down	1.641638	up	-5.52218	down	-3.21586	down
Os.4185.1.A1_at	-3.260973	down	1.418618	up	-4.77188	down	-2.2987	down
Os.50646.1.S1_s_at	2.0886166	up	-1.48658	down	5.000011	up	1.404977	up
Os.49676.1.S1_at	-2.8824947	down	-5.27254	down	1.567943	up	-15.1981	down
Os.7862.1.S1_at	2.8306978	up	1.245147	up	2.694898	up	3.524634	up
OsAffx.14098.1.S1_at	-1.7729285	down	-1.12389	down	-2.98095	down	-1.99258	down
Os.11892.1.S1_at	-3.1777005	down	-1.13463	down	-2.95095	down	-3.60552	down
Os.16597.1.S1_s_at	3.4017296	up	1.38974	up	2.408441	up	4.727519	up
Os.27000.1.S1_s_at	3.624595	up	-1.46039	down	4.886559	up	2.481929	up
Os.40018.1.S1_at	1.8430281	up	2.638707	up	1.267727	up	4.863211	up
Os.12392.1.S1_at	1.7204838	up	6.26213	up	-1.87281	down	10.77389	up
Os.27963.1.A1_at	1.4222801	up	-5.19562	down	1.554301	up	-3.65302	down
Os.24050.1.S1_at	3.0929832	up	-1.04166	down	3.479716	up	2.969286	up
OsAffx.26801.1.S1_x_	-1.6827902	down	-1.63483	down	-2.04091	down	-2.75108	down
Os.5675.1.S1_at	-2.9440756	down	-1.11858	down	-2.98101	down	-3.2932	down
Os.28397.1.S1_at	2.1392007	up	3.476589	up	-1.04289	down	7.437122	up
Os.52892.1.S1_at	7.331451	up	1.239104	up	2.68763	up	9.084426	up
Os.34196.1.S1_at	2.0166948	up	1.310957	up	2.540048	up	2.6438	up
Os.10880.1.S1_at	1.3971095	up	1.625796	up	2.048161	up	2.271415	up
Os.34767.2.S1_s_at	-3.8018987	down	-1.10909	down	-3.00214	down	-4.21664	down
Os.9679.1.S1_at	3.2936876	up	-2.60094	down	8.647246	up	1.266345	up
Os.11786.2.S1_x_at	10.505706	up	-1.93105	down	6.416905	up	5.440404	up
Os.22711.1.A1_at	-1.2642685	down	1.514907	up	2.193132	up	1.198248	up
Os.50873.1.S1_x_at	2.4075058	up	1.711532	up	1.939182	up	4.120523	up
Os.51593.1.S1_at	2.2103148	up	2.107625	up	1.574392	up	4.658513	up
Os.51711.1.S1_at	1.7588866	up	-1.23335	down	4.092361	up	1.426109	up
Os.12393.1.S1_at	6.923097	up	-1.46657	down	4.865994	up	4.7206	up
Os.9558.1.S1_s_at	2.570883	up	2.468177	up	1.343816	up	6.345395	up
Os.7711.1.S1_at	4.1465316	up	-2.31161	down	7.66558	up	1.793783	up
Os.23349.1.S1_x_at	-2.1594653	down	-1.41651	down	-2.33397	down	-3.0589	down
Os.14560.1.S1_at	1.4807038	up	1.088667	up	3.036702	up	1.611993	up
Os.21782.1.S1_at	2.3924055	up	1.371986	up	2.408916	up	3.282347	up
Os.26133.1.S1_at	2.1018457	up	2.101497	up	1.572257	up	4.417021	up
Os.36351.1.S1_at	2.6463087	up	-1.19985	down	3.960529	up	2.20554	up
Os.5343.1.S1_at	2.8150165	up	-1.1243	down	3.709544	up	2.503798	up
OsAffx.2475.1.S1_x_a	-2.8988588	down	-1.02787	down	-3.20898	down	-2.97965	down
Os.44821.1.A1_x_at	4.6961484	up	-1.66191	down	5.479816	up	2.825754	up
Os.7228.1.S1_at	3.3733733	up	-1.24391	down	4.100949	up	2.711903	up
Os.25167.1.S1_a_at	-1.8343686	down	1.04213	up	-3.4326	down	-1.76021	down
Os.17642.1.S1_a_at	-2.8941858	down	1.010106	up	-3.3248	down	-2.86523	down
OsAffx.20051.1.S1_at	1.1106098	up	1.316015	up	2.500704	up	1.461579	up
OsAffx.25073.1.S1_x_	-1.1014268	down	1.145286	up	2.872688	up	1.03982	up
Os.5064.1.S1_at	5.02612	up	-1.44516	down	4.751946	up	3.477892	up
Os.7743.1.S1_at	1.7117685	up	3.043233	up	1.079138	up	5.20931	up
Os.49658.1.S1_at	2.3583407	up	1.264174	up	2.597613	up	2.981353	up
Os.50164.1.S1_at	-3.731302	down	-1.22183	down	-2.6869	down	-4.559	down
Os.11867.2.S1_at	2.3166318	up	1.188291	up	2.76059	up	2.752832	up

Os.32123.1.S1_at	-2.2992516	down	-1.48226	down	-2.21306	down	-3.40809	down
Os.5093.1.S1_at	2.7539058	up	2.773639	up	1.181932	up	7.63834	up
Os.12366.1.S1_x_at	3.424797	up	1.048984	up	3.125043	up	3.592556	up
OsAffx.15233.1.S1_at	-1.7665551	down	-2.56837	down	-1.27592	down	-4.53716	down
Os.17042.1.S1_at	-3.3435771	down	-1.01541	down	-3.22689	down	-3.39509	down
OsAffx.7824.1.S1_s_a	-3.187675	down	1.203823	up	-3.94444	down	-2.64796	down
Os.8570.4.S1_at	-2.6355553	down	-1.07284	down	-3.05072	down	-2.82753	down
OsAffx.26722.2.S1_s_	-2.7265038	down	-1.01585	down	-3.21924	down	-2.76971	down
Os.54794.1.S1_at	2.091752	up	1.688094	up	1.934948	up	3.531073	up
Os.3749.1.S1_at	-3.551875	down	-1.2535	down	-2.60239	down	-4.45226	down
Os.10333.1.S1_at	-1.2011169	down	-2.38815	down	-1.36465	down	-2.86845	down
Os.9688.1.S1_at	3.055992	up	-1.16802	down	3.8058	up	2.616381	up
Os.27180.1.S1_at	-2.2569814	down	-1.90703	down	-1.708	down	-4.30414	down
OsAffx.15063.1.S1_s_	2.4335861	up	-1.05743	down	3.442695	up	2.301412	up
Os.23844.1.A1_at	2.7581956	up	-1.11287	down	3.623138	up	2.478459	up
Os.14395.1.S1_at	2.4531825	up	1.338795	up	2.431413	up	3.284309	up
Os.49323.1.S1_at	-7.027756	down	1.273137	up	-4.14225	down	-5.52003	down
OsAffx.32309.1.A1_at	-1.020279	down	-2.57719	down	-1.26222	down	-2.62945	down
Os.5826.2.S1_a_at	2.0983672	up	4.23644	up	-1.30247	down	8.889607	up
Os.7772.2.A1_x_at	2.757637	up	-1.23662	down	4.021918	up	2.229978	up
OsAffx.24328.1.S1_at	1.0593859	up	2.117254	up	1.535836	up	2.242989	up
Os.7335.1.S1_at	1.7129265	up	2.287251	up	1.42117	up	3.917893	up
Os.47958.1.A1_x_at	1.5349613	up	2.091158	up	1.554285	up	3.209847	up
Os.2235.1.S1_a_at	3.1246524	up	-1.08505	down	3.525918	up	2.879725	up
Os.51637.1.S1_x_at	-3.0344474	down	-2.09447	down	-1.54938	down	-6.35557	down
Os.27224.2.A1_at	1.1040317	up	1.959052	up	1.655938	up	2.162855	up
OsAffx.5283.1.S1_at	1.6330919	up	3.898099	up	-1.20207	down	6.365954	up
Os.46725.1.S1_at	1.860108	up	2.581991	up	1.255549	up	4.802781	up
Os.3985.1.S1_at	-2.3898697	down	-1.87217	down	-1.73083	down	-4.47425	down
Os.4281.1.S1_x_at	-1.8260623	down	-3.3582	down	1.036653	up	-6.13229	down
Os.4921.1.S1_at	-3.472179	down	-1.12619	down	-2.87615	down	-3.91035	down
Os.8697.1.S1_at	2.7328014	up	1.401974	up	2.308959	up	3.831317	up
Os.49242.1.S1_at	-3.2443998	down	-1.45162	down	-2.22987	down	-4.70963	down
Os.24890.1.S1_at	2.1794233	up	1.916531	up	1.688624	up	4.176931	up
Os.15089.1.S1_at	-3.6125865	down	-1.2964	down	-2.49342	down	-4.68335	down
Os.51731.1.S1_at	1.8258082	up	-1.73738	down	5.614204	up	1.050899	up
Os.7705.1.S1_at	-2.5341733	down	-2.69603	down	-1.19675	down	-6.8322	down
OsAffx.13479.1.S1_at	2.6506665	up	-1.17554	down	3.792546	up	2.254858	up
OsAffx.31976.1.S1_s_	-1.2085322	down	-4.71301	down	1.461205	up	-5.69583	down
Os.19951.1.S1_x_at	2.359193	up	1.306963	up	2.467029	up	3.083378	up
Os.32086.1.S1_at	3.9032516	up	-1.18084	down	3.804008	up	3.30548	up
OsAffx.4557.1.S1_s_a	10.588208	up	-5.17341	down	16.65004	up	2.046659	up
Os.9653.1.S1_s_at	1.808478	up	1.597252	up	2.014277	up	2.888595	up
Os.10422.1.S1_at	11.942433	up	1.565217	up	2.05544	up	18.6925	up
Os.52820.1.S1_at	1.2314066	up	-1.35424	down	4.356844	up	-1.09975	down
Os.38258.2.S1_at	1.6357429	up	1.214163	up	2.647649	up	1.986059	up
Os.50438.1.S1_at	2.78698	up	2.347435	up	1.368637	up	6.542254	up

Os.14415.1.S1_a_at	2.881393	up	2.357711	up	1.362198	up	6.793491	up
Os.2444.1.S1_at	-1.7778525	down	-1.11108	down	-2.88851	down	-1.97534	down
Os.11266.1.S1_at	-3.8572233	down	2.928426	up	-9.39747	down	-1.31717	down
Os.37723.1.S1_at	-3.1714363	down	1.49356	up	-4.78871	down	-2.12341	down
Os.5395.1.S1_at	-1.6862787	down	-1.36159	down	-2.35408	down	-2.29602	down
Os.27685.1.S1_at	-2.0111482	down	-1.46236	down	-2.18831	down	-2.94102	down
OsAffx.14409.1.S1_at	-2.3263807	down	-1.05347	down	-3.03657	down	-2.45077	down
Os.55208.1.S1_at	-5.294468	down	1.670145	up	-5.3415	down	-3.17006	down
Os.28176.1.S1_at	3.0072398	up	1.50237	up	2.126562	up	4.517986	up
Os.55076.1.S1_at	-3.8116922	down	1.141492	up	-3.64483	down	-3.33922	down
Os.6581.1.S1_at	2.7661188	up	3.348271	up	-1.04864	down	9.261715	up
Os.52056.1.S1_at	2.441587	up	1.087636	up	2.931352	up	2.655559	up
Os.27323.2.S1_x_at	6.8215117	up	1.037876	up	3.070272	up	7.079884	up
Os.36901.1.S1_at	9.663504	up	1.446347	up	2.201237	up	13.97677	up
Os.21319.1.S1_at	-1.3709953	down	-1.23513	down	-2.57631	down	-1.69335	down
Os.43043.1.S1_at	1.9503156	up	2.010979	up	1.581822	up	3.922044	up
Os.7512.1.S1_at	-2.5582943	down	-1.74074	down	-1.82547	down	-4.45334	down
Os.5016.1.S1_at	4.0692196	up	1.493948	up	2.126908	up	6.079202	up
Os.45916.1.S1_s_at	-1.0712371	down	2.353059	up	1.350336	up	2.196581	up
Os.46582.1.S1_at	6.8798633	up	-16.682	down	52.9972	up	-2.42476	down
OsAffx.23810.1.S1_at	4.468909	up	1.355673	up	2.340838	up	6.058377	up
Os.53459.1.S1_at	2.3614328	up	2.17808	up	1.455642	up	5.14339	up
Os.55647.1.A1_at	1.9797857	up	2.296082	up	1.380585	up	4.545751	up
OsAffx.31482.1.S1_s_	4.9200506	up	-1.36903	down	4.333965	up	3.593832	up
Os.56880.1.S1_at	-1.1408355	down	1.169765	up	2.70537	up	1.025358	up
Os.22995.1.S1_at	3.233	up	5.511321	up	-1.74172	down	17.8181	up
OsAffx.11144.1.S1_at	2.1888838	up	1.514563	up	2.088458	up	3.315202	up
Os.54385.1.S1_at	-1.3472195	down	2.065885	up	1.530344	up	1.533444	up
Os.57327.1.S1_at	1.3442769	up	1.035178	up	3.052708	up	1.391566	up
Os.49638.1.S1_at	2.873563	up	1.144902	up	2.759697	up	3.289949	up
Os.9247.2.S1_x_at	-2.2087488	down	-1.07042	down	-2.95104	down	-2.36429	down
Os.32086.2.S1_x_at	5.357041	up	-1.55136	down	4.900397	up	3.453125	up
Os.1205.1.S1_at	1.7028651	up	-2.25973	down	7.13686	up	-1.32702	down
Os.14139.1.S1_at	-3.0846336	down	-1.35241	down	-2.33248	down	-4.17169	down
Os.52682.1.S1_at	-2.6139684	down	-1.01569	down	-3.10058	down	-2.65497	down
OsAffx.26230.1.S1_at	-4.746928	down	1.318788	up	-4.15075	down	-3.59946	down
Os.10006.1.S1_at	1.7652587	up	1.434901	up	2.190755	up	2.532971	up
Os.28197.1.S2_at	-2.2727683	down	-1.31159	down	-2.39562	down	-2.98095	down
Os.11617.1.S1_at	2.9020123	up	2.505958	up	1.25375	up	7.272321	up
Os.7043.1.S1_at	3.5224133	up	-1.03888	down	3.262803	up	3.39058	up
Os.4695.1.S1_s_at	2.0270555	up	2.885893	up	1.088088	up	5.849866	up
Os.11481.1.S1_s_at	-1.9433838	down	-1.27244	down	-2.46756	down	-2.47284	down
Os.25449.1.S1_at	1.2245767	up	-1.72767	down	5.424158	up	-1.41083	down
Os.38507.1.S1_at	2.092034	up	1.226422	up	2.55698	up	2.565716	up
OsAffx.5859.1.S1_x_a	4.6311836	up	-1.34514	down	4.216768	up	3.442895	up
Os.20980.1.S1_at	6.6098256	up	-1.39092	down	4.359913	up	4.752123	up
Os.54115.1.S1_at	1.9478158	up	1.032116	up	3.035467	up	2.010372	up

OsAffx.14410.1.S1_s_	2.3108022	up	-1.13296	down	3.549369	up	2.039613	up
Os.4968.1.S1_at	3.6778085	up	1.240187	up	2.525938	up	4.561172	up
Os.51005.1.S1_at	2.1192634	up	2.032374	up	1.540975	up	4.307136	up
Os.52619.1.S1_at	1.6269778	up	4.729439	up	-1.51101	down	7.694692	up
OsAffx.14888.1.S1_at	1.9599181	up	3.571361	up	-1.14127	down	6.999576	up
Os.51783.1.S1_at	1.926426	up	1.49232	up	2.095549	up	2.874844	up
Os.6400.1.S1_a_at	1.5908339	up	1.220858	up	2.560959	up	1.942182	up
Os.45924.1.S1_at	1.3965833	up	1.040725	up	3.003268	up	1.453459	up
Os.8961.1.S1_s_at	1.8979288	up	3.046183	up	1.025289	up	5.781439	up
Os.25548.1.A1_x_at	-2.7222269	down	1.019514	up	-3.18277	down	-2.67012	down
OsAffx.23482.1.S1_at	1.4800738	up	1.785653	up	1.747933	up	2.642898	up
Os.26827.1.A1_at	4.737956	up	-2.01272	down	6.274392	up	2.354003	up
OsAffx.32032.4.S1_s_	1.0949732	up	1.485221	up	2.095936	up	1.626277	up
Os.50019.1.S1_at	63.160088	up	-48.2948	down	150.3104	up	1.307804	up
Os.19553.1.S1_at	4.587512	up	-1.05419	down	3.280305	up	4.351687	up
Os.23808.2.S1_at	-3.0002146	down	-1.11093	down	-2.79858	down	-3.33303	down
Os.34504.1.S1_at	-2.0410337	down	-1.39039	down	-2.23574	down	-2.83783	down
Os.30473.1.S1_at	1.92916	up	2.317076	up	1.341172	up	4.470012	up
OsAffx.21616.1.S1_s_	-1.4111661	down	2.920465	up	1.063016	up	2.06954	up
Os.16956.1.S1_s_at	2.5167093	up	1.263603	up	2.455056	up	3.180121	up
OsAffx.23960.1.S1_s_	2.1404643	up	1.867241	up	1.66128	up	3.996762	up
Os.45516.1.S1_at	-12.168406	down	2.106935	up	-6.52788	down	-5.77541	down
Os.47908.1.S1_at	-5.592526	down	1.269702	up	-3.93254	down	-4.4046	down
Os.14862.1.S1_a_at	-4.2374263	down	-1.16794	down	-2.65043	down	-4.94907	down
Os.17198.1.S1_at	1.1034645	up	1.652322	up	1.873138	up	1.823279	up
Os.47761.1.S1_at	2.606245	up	1.480719	up	2.088702	up	3.859117	up
Os.27176.1.S1_at	2.100443	up	2.211297	up	1.3982	up	4.644703	up
Os.6990.1.S1_at	1.4840293	up	1.614528	up	1.913725	up	2.396006	up
Os.11961.1.S1_at	-2.2709248	down	-1.38857	down	-2.22376	down	-3.15334	down
Os.4945.1.S1_at	3.4746964	up	1.274169	up	2.422002	up	4.427352	up
Os.7899.1.S1_at	5.792271	up	-1.65215	down	5.097011	up	3.505893	up
OsAffx.27338.1.S1_at	-1.5365844	down	-2.33042	down	-1.32349	down	-3.58088	down
Os.26629.1.S1_a_at	-3.4821894	down	-1.20482	down	-2.55885	down	-4.19543	down
Os.5936.1.S1_at	4.0524516	up	-1.074	down	3.31087	up	3.773216	up
Os.52451.1.A1_at	-1.4332453	down	3.64674	up	-1.18301	down	2.544393	up
Os.22197.1.S1_at	-5.1197295	down	-1.05031	down	-2.93142	down	-5.3773	down
Os.48711.1.A1_at	2.0156944	up	2.188561	up	1.406514	up	4.41147	up
OsAffx.29955.1.S1_at	5.2655816	up	-1.13617	down	3.497048	up	4.634485	up
Os.6142.1.S1_s_at	1.9238832	up	1.418303	up	2.169718	up	2.728649	up
Os.12922.1.S1_at	1.683169	up	1.936014	up	1.588346	up	3.258638	up
Os.53396.1.S1_at	-2.0800328	down	-2.53963	down	-1.20941	down	-5.28252	down
Os.8559.1.S1_at	18.7189	up	-5.77767	down	17.73216	up	3.239871	up
Os.31771.2.S1_at	2.1626887	up	-1.25527	down	3.851956	up	1.722893	up
Os.46600.1.S1_at	-1.8749212	down	-1.30957	down	-2.34254	down	-2.45534	down
Os.37215.1.S1_at	2.309034	up	1.522124	up	2.015337	up	3.514637	up
Os.56026.1.A1_at	2.2800066	up	1.23968	up	2.473263	up	2.82648	up
Os.15747.1.A1_at	4.20295	up	-2.03556	down	6.240467	up	2.064767	up

Os.11203.1.S1_at	7.0005946	up	-1.42366	down	4.363734	up	4.917326	up
Os.14616.1.S1_at	5.3508162	up	2.611265	up	1.170848	up	13.9724	up
Os.51127.2.S1_x_at	-1.7746664	down	1.326154	up	-4.04971	down	-1.33821	down
OsAffx.21738.1.S1_x_	2.3583817	up	-1.27793	down	3.895176	up	1.845474	up
Os.12096.4.S1_s_at	-1.0807922	down	1.380072	up	-4.2057	down	1.276908	up
Os.23227.1.S1_at	-4.537551	down	-1.21354	down	-2.50941	down	-5.50652	down
OsAffx.26540.1.S1_s_	3.6261258	up	1.009705	up	3.014787	up	3.661316	up
Os.30032.2.S1_x_at	-2.5209043	down	-1.186	down	-2.56585	down	-2.98978	down
AFFX-r2-Bs-dap-5_at	-1.5572652	down	1.066882	up	-3.24546	down	-1.45964	down
Os.9995.1.S1_at	1.282586	up	-1.22908	down	3.736886	up	1.043532	up
Os.27527.1.S1_at	1.774484	up	1.986163	up	1.530719	up	3.524415	up
Os.55360.1.S1_at	4.142059	up	1.143554	up	2.657166	up	4.736667	up
Os.22312.2.S1_x_at	-2.5175862	down	-1.45298	down	-2.09014	down	-3.658	down
Os.16999.1.A1_at	-2.9821072	down	-1.51264	down	-2.00314	down	-4.51087	down
OsAffx.17948.1.S1_x_	-2.7104146	down	-1.57405	down	-1.92319	down	-4.26632	down
Os.22928.2.S1_at	4.8136272	up	-1.38458	down	4.187932	up	3.476595	up
Os.53639.1.S1_at	4.211628	up	1.885426	up	1.603657	up	7.940712	up
Os.7229.1.S1_at	2.7893286	up	1.101089	up	2.745623	up	3.071298	up
Os.23153.1.S1_s_at	-3.1316392	down	1.527614	up	-4.61614	down	-2.05002	down
Os.4900.2.A1_x_at	11.219878	up	-1.49099	down	4.503761	up	7.525115	up
OsAffx.25769.1.S1_at	-2.0934098	down	-1.71541	down	-1.75958	down	-3.59105	down
Os.43040.1.S1_at	3.172319	up	1.26547	up	2.383063	up	4.014476	up
Os.37955.2.S1_at	3.0692923	up	-1.00509	down	3.030036	up	3.05374	up
Os.50428.1.S1_at	-3.4815943	down	1.558789	up	-4.69757	down	-2.23352	down
Os.6377.1.S1_at	2.723581	up	1.611914	up	1.867834	up	4.390177	up
OsAffx.10965.1.S1_at	1.1415584	up	1.204672	up	2.498814	up	1.375203	up
Os.34462.1.S1_at	11.57607	up	-1.08356	down	3.258505	up	10.68336	up
Os.834.1.S1_a_at	2.2872324	up	2.017051	up	1.490642	up	4.613463	up
Os.47742.1.S1_at	1.4216604	up	1.639525	up	1.833736	up	2.330848	up
Os.27632.1.S1_at	-3.0783308	down	1.822878	up	-5.47632	down	-1.68872	down
OsAffx.15233.1.S1_x_	-1.1243477	down	-2.37106	down	-1.26668	down	-2.6659	down
Os.54579.1.S1_s_at	3.205561	up	1.407873	up	2.132937	up	4.513022	up
Os.15570.2.S1_at	1.5616978	up	1.170011	up	2.566271	up	1.827203	up
Os.28798.1.S1_at	1.7020787	up	1.915986	up	1.566029	up	3.261159	up
Os.26827.2.A1_x_at	5.6681256	up	-2.39054	down	7.164387	up	2.371061	up
Os.6263.1.S1_at	2.6885035	up	1.483108	up	2.020488	up	3.987341	up
Os.11570.1.S1_at	1.9932262	up	1.849259	up	1.619436	up	3.685991	up
OsAffx.25749.1.S1_s_	3.4518476	up	1.462792	up	2.046042	up	5.049337	up
Os.22968.1.A1_at	2.1993217	up	-2.59019	down	7.751269	up	-1.17772	down
Os.50398.1.S1_at	3.7340658	up	-1.17929	down	3.524279	up	3.166381	up
Os.49054.1.S1_at	4.0171375	up	2.518083	up	1.186083	up	10.11549	up
Os.28300.2.S1_a_at	3.907265	up	1.019424	up	2.929552	up	3.983158	up
Os.30059.1.S1_at	1.5506098	up	2.587969	up	1.153529	up	4.012929	up
Os.5174.1.S1_at	3.9391274	up	1.528379	up	1.953138	up	6.020478	up
Os.8851.1.S1_at	-2.1430402	down	-2.92733	down	-1.01966	down	-6.27339	down
Os.9556.1.S1_at	-4.1749625	down	-1.04352	down	-2.85946	down	-4.35667	down
Os.11907.1.S1_at	1.4767901	up	-2.09359	down	-1.42512	down	-1.41766	down

OsAffx.29026.1.S1_at	1.2645797	up	1.861312	up	1.602753	up	2.353777	up
OsAffx.31572.1.S1_at	2.4722517	up	1.980648	up	1.505417	up	4.896659	up
Os.606.1.S1_at	3.6648598	up	-1.14887	down	3.425089	up	3.189978	up
Os.14380.1.S1_s_at	-3.9832785	down	1.834124	up	-5.46688	down	-2.17176	down
Os.2117.1.S1_at	3.4858139	up	1.192635	up	2.49886	up	4.157306	up
Os.7150.1.S1_at	2.7109406	up	2.179076	up	1.366829	up	5.907346	up
Os.10056.1.S1_at	1.6514966	up	-1.00657	down	2.996226	up	1.640712	up
Os.27945.1.A1_at	-1.8949909	down	-1.53838	down	-1.93345	down	-2.91521	down
Os.26626.1.S1_at	2.4463353	up	1.934233	up	1.535356	up	4.731782	up
Os.27070.1.S1_at	1.3650192	up	1.290057	up	2.301268	up	1.760953	up
Os.51772.1.S1_at	1.9517177	up	-1.40938	down	4.183351	up	1.384806	up
Os.47625.1.A1_s_at	1.7118797	up	34.95593	up	-11.7821	down	59.84035	up
Os.12032.1.S1_at	1.2541869	up	3.506797	up	-1.18282	down	4.398179	up
OsAffx.26173.1.S1_s_	-3.117602	down	1.844971	up	-5.46896	down	-1.68978	down
Os.11905.2.S1_x_at	-2.708984	down	-1.19506	down	-2.47993	down	-3.23739	down
Os.46826.1.S1_at	-3.608164	down	-1.18355	down	-2.50362	down	-4.27045	down
Os.5311.1.S1_at	-2.089401	down	-1.26586	down	-2.3408	down	-2.64488	down
Os.37548.1.S1_at	2.9577827	up	1.677846	up	1.765729	up	4.962705	up
Os.57417.1.S1_s_at	3.01529	up	-1.04649	down	3.098911	up	2.881335	up
Os.47251.1.S1_at	1.6275208	up	1.281032	up	2.310414	up	2.084905	up
Os.7044.1.S1_at	1.7098017	up	-1.30105	down	3.8474	up	1.314173	up
Os.9013.1.S1_at	-2.9557543	down	2.373346	up	-7.01771	down	-1.2454	down
Os.46540.1.S1_at	-2.5061574	down	1.148462	up	-3.39425	down	-2.18219	down
Os.17486.2.A1_at	-2.4705195	down	-1.24878	down	-2.36663	down	-3.08513	down
Os.9709.2.S1_at	2.085723	up	3.070923	up	-1.03928	down	6.405095	up
Os.30044.1.S1_a_at	1.7889136	up	2.349517	up	1.256941	up	4.203082	up
Os.11570.1.S1_s_at	1.8194196	up	1.6092	up	1.835031	up	2.927811	up
Os.14745.1.S1_at	2.1368961	up	7.620454	up	-2.5808	down	16.28412	up
Os.5565.1.S1_at	-2.2961867	down	-1.43599	down	-2.05586	down	-3.2973	down
Os.57563.1.S1_s_at	-1.1184586	down	2.561802	up	1.15237	up	2.290475	up
OsAffx.32196.1.A1_at	-1.1609514	down	-2.61209	down	-1.12925	down	-3.03251	down
OsAffx.23903.2.S1_s_	2.5125043	up	1.211564	up	2.434562	up	3.044059	up
OsAffx.5984.1.S1_s_a	-2.4301875	down	-1.00689	down	-2.92932	down	-2.44692	down
Os.51227.1.S1_x_at	-1.1763296	down	1.724534	up	-5.08623	down	1.466029	up
Os.11883.2.S1_at	1.7545761	up	1.838447	up	1.603211	up	3.225695	up
Os.28435.4.S1_x_at	-1.7438346	down	-2.62906	down	-1.12078	down	-4.58465	down
Os.49347.1.S1_at	-2.9749043	down	-1.10879	down	-2.65541	down	-3.29856	down
Os.24540.1.A1_at	-2.5135942	down	-1.24734	down	-2.35874	down	-3.13531	down
Os.17895.1.S1_a_at	-2.0590403	down	1.13176	up	-3.32914	down	-1.81933	down
Os.11844.1.S1_at	2.1681647	up	1.986153	up	1.480028	up	4.306307	up
Os.12191.1.S1_s_at	-1.0538336	down	-8.64106	down	2.940848	up	-9.10624	down
Os.51943.1.S1_at	2.7196338	up	1.478647	up	1.986268	up	4.021378	up
Os.14271.1.S1_at	-1.6952677	down	1.155529	up	-3.39317	down	-1.46709	down
Os.57345.1.S1_at	-1.0659208	down	1.377556	up	2.131437	up	1.292363	up
OsAffx.23844.1.S1_at	-2.2594912	down	-1.38965	down	-2.11251	down	-3.13989	down
Os.25067.1.S1_a_at	-2.9327152	down	-1.94537	down	-1.50834	down	-5.70522	down
Os.5403.1.S1_at	2.9233913	up	-1.24136	down	3.64161	up	2.354986	up

Os.11943.2.S1_at	-1.064208	down	1.408649	up	2.082226	up	1.32366	up
Os.9708.1.S1_at	-1.0179119	down	2.192273	up	1.337837	up	2.153696	up
Os.6625.1.S1_x_at	3.0939913	up	-1.17747	down	3.453167	up	2.627652	up
Os.48354.1.S1_at	2.5862377	up	1.136491	up	2.57832	up	2.939236	up
Os.6654.1.S1_at	1.6419884	up	18.51346	up	-6.32377	down	30.39889	up
Os.53435.1.S1_at	5.8556237	up	1.709925	up	1.711802	up	10.01267	up
Os.51797.1.S1_at	-1.9077854	down	-1.65246	down	-1.77117	down	-3.15253	down
Os.7174.1.S1_at	2.0237105	up	-1.13202	down	3.313094	up	1.787695	up
Os.27509.1.S1_at	-1.9162376	down	-1.34984	down	-2.16717	down	-2.58661	down
Os.11092.1.S1_at	3.0625622	up	-1.18993	down	3.479816	up	2.573727	up
Os.51439.1.S1_x_at	3.0560184	up	2.409868	up	1.213357	up	7.3646	up
Os.48981.1.S1_at	1.613927	up	-1.36496	down	3.989651	up	1.182395	up
Os.16401.1.S1_at	1.5816734	up	2.501147	up	1.168374	up	3.955998	up
Os.18314.1.S1_at	1.6003249	up	-1.20469	down	3.518874	up	1.328413	up
Os.37893.1.S1_at	1.3125648	up	3.066838	up	-1.05008	down	4.025424	up
Os.35049.1.S1_a_at	-2.2883945	down	-1.37092	down	-2.12916	down	-3.1372	down
Os.52796.2.S1_x_at	-1.0325112	down	1.782673	up	1.636775	up	1.726541	up
Os.4940.1.S1_at	1.2613657	up	5.425062	up	-1.86085	down	6.842986	up
Os.12937.1.S1_at	1.6425908	up	2.377595	up	1.225768	up	3.905416	up
Os.11781.1.S1_at	3.4165716	up	1.20346	up	2.419866	up	4.111708	up
Os.52999.1.S1_at	-3.1984909	down	-1.10857	down	-2.62202	down	-3.54574	down
OsAffx.12356.1.S1_at	1.8756185	up	1.441493	up	2.015194	up	2.703691	up
Os.23067.1.S1_at	-3.2708225	down	-5.56046	down	1.914492	up	-18.1873	down
Os.11109.1.S1_at	2.1190383	up	1.786588	up	1.625489	up	3.785848	up
Os.22570.1.S1_at	2.5808465	up	1.22211	up	2.375615	up	3.154079	up
Os.35356.1.S1_at	2.0622792	up	1.649853	up	1.758717	up	3.402457	up
Os.32471.1.S1_at	-2.4050972	down	1.669601	up	-4.837	down	-1.44052	down
Os.28300.2.S2_a_at	4.407969	up	-1.26114	down	3.650477	up	3.495219	up
Os.16760.1.S1_at	1.9290475	up	1.012965	up	2.855843	up	1.954058	up
Os.52910.1.S1_at	-4.204179	down	-1.35211	down	-2.13876	down	-5.68449	down
Os.9554.1.S1_at	2.615224	up	1.455838	up	1.985867	up	3.807344	up
Os.54080.1.S1_at	4.1286407	up	-2.23577	down	6.463607	up	1.846627	up
Os.49253.1.S1_at	2.4150681	up	1.591579	up	1.816347	up	3.843772	up
Os.16129.1.S1_at	2.6445887	up	-1.23946	down	3.582883	up	2.133664	up
Os.1437.1.S1_at	1.9373862	up	4.512528	up	-1.56107	down	8.74251	up
Os.24860.1.S1_at	-2.7152698	down	1.11724	up	-3.22893	down	-2.43034	down
OsAffx.23032.1.S1_at	-1.9250969	down	-1.94456	down	-1.48609	down	-3.74347	down
Os.49867.1.S1_at	-3.2779744	down	-1.35499	down	-2.13259	down	-4.44161	down
Os.5755.1.S1_at	1.6312503	up	1.406051	up	2.054181	up	2.293621	up
OsAffx.32227.1.A1_at	-1.0468293	down	-1.62243	down	-1.77962	down	-1.6984	down
Os.28617.1.S1_at	6.2208405	up	-3.27961	down	9.468031	up	1.896825	up
Os.17927.2.S1_x_at	-2.7599225	down	-1.12813	down	-2.5589	down	-3.11355	down
Os.12035.1.S1_at	-1.5725592	down	-1.29575	down	-2.22622	down	-2.03765	down
Os.26821.1.S1_at	-2.8884635	down	1.263843	up	-3.6436	down	-2.28546	down
Os.3927.2.S1_s_at	2.1356144	up	1.777707	up	1.621543	up	3.796496	up
Os.17265.1.S1_at	-2.550796	down	-1.23184	down	-2.33785	down	-3.14218	down
Os.28290.1.S1_at	-3.502999	down	1.494929	up	-4.3046	down	-2.34326	down

Os.6256.2.S1_at	2.3474247	up	1.053075	up	2.732492	up	2.472014	up
Os.27043.1.A1_at	-1.926794	down	-1.45416	down	-1.97875	down	-2.80187	down
Os.50300.1.S1_s_at	1.0071878	up	2.186653	up	1.315536	up	2.20237	up
Os.27387.1.S1_at	1.7534629	up	1.004118	up	2.864755	up	1.760683	up
Os.11666.1.S1_s_at	3.7746277	up	1.208317	up	2.380304	up	4.560946	up
Os.38299.1.S1_at	1.0803617	up	2.391651	up	1.202543	up	2.583848	up
Os.20050.1.A1_at	-6.7546625	down	1.305888	up	-3.75562	down	-5.17247	down
Os.29987.2.S1_at	5.2813125	up	1.562318	up	1.840726	up	8.25109	up
Os.4292.1.S1_at	-1.3083749	down	-1.80866	down	-1.58984	down	-2.36641	down
Os.55612.1.S1_at	-5.426291	down	3.022019	up	-8.68852	down	-1.79558	down
Os.5188.2.S1_s_at	-1.2343104	down	1.560631	up	1.841858	up	1.264375	up
Os.11851.1.S1_at	4.2666574	up	-1.26782	down	3.642781	up	3.365353	up
Os.56922.1.S1_at	4.3689713	up	-1.14233	down	3.282053	up	3.824621	up
Os.6561.1.A1_at	2.6437967	up	1.024255	up	2.805075	up	2.707921	up
Os.25062.2.S1_a_at	-2.0322888	down	-1.21934	down	-2.3562	down	-2.47805	down
OsAffx.12016.1.S1_at	-2.7981863	down	1.038373	up	-2.97977	down	-2.69478	down
OsAffx.24953.1.S1_s_	6.1228027	up	1.024485	up	2.799082	up	6.272716	up
Os.15000.1.S1_a_at	-2.4636385	down	-1.04132	down	-2.75144	down	-2.56545	down
Os.9243.1.S1_at	-3.5341692	down	-1.12016	down	-2.55672	down	-3.95884	down
OsAffx.21010.1.S1_s_	1.2533	up	1.636752	up	1.748599	up	2.051342	up
Os.49540.1.A1_x_at	2.0645661	up	2.511105	up	1.139504	up	5.184341	up
Os.8208.1.S1_at	2.0924299	up	-8.59712	down	24.58516	up	-4.10868	down
Os.10830.1.S1_at	-3.335228	down	-1.53365	down	-1.86418	down	-5.11508	down
Os.19954.1.S1_at	1.4453561	up	1.700597	up	1.680819	up	2.457969	up
Os.23598.1.S1_at	1.8042566	up	1.262641	up	2.263064	up	2.278129	up
Os.39576.1.A1_s_at	-4.7901073	down	-1.32322	down	-2.15532	down	-6.33838	down
OsAffx.29953.1.S1_at	-1.7739594	down	-1.56666	down	-1.81894	down	-2.7792	down
Os.7595.1.S1_at	-3.1517482	down	-1.11189	down	-2.56281	down	-3.50439	down
Os.27484.1.S1_at	1.4966022	up	2.615583	up	1.089109	up	3.914487	up
Os.6965.1.S1_at	-1.3905944	down	3.975527	up	-1.39598	down	2.858869	up
Os.48082.1.S1_at	1.8228538	up	5.139413	up	-1.80541	down	9.368399	up
Os.46793.1.S1_at	1.7718568	up	3.305932	up	-1.16142	down	5.857638	up
Os.12821.1.S1_at	1.0410686	up	-1.44209	down	4.103559	up	-1.38521	down
Os.14803.1.S1_at	-3.057128	down	1.525784	up	-4.33922	down	-2.00364	down
Os.5052.1.S1_at	-2.5209057	down	-1.24039	down	-2.29179	down	-3.12691	down
Os.15814.1.S1_at	1.4924213	up	1.653522	up	1.718633	up	2.467751	up
Os.17201.1.S1_at	-3.0682604	down	1.070919	up	-3.04314	down	-2.86507	down
Os.4796.1.S1_at	-3.1799855	down	-1.16838	down	-2.4319	down	-3.71544	down
Os.11638.1.S1_at	-1.0721049	down	-2.38898	down	-1.18924	down	-2.56123	down
OsAffx.29627.2.S1_at	-2.1865466	down	-1.2342	down	-2.29973	down	-2.69863	down
Os.14348.1.S1_x_at	-3.7072163	down	1.172463	up	-3.32659	down	-3.16191	down
Os.11147.1.S1_at	1.1965715	up	1.102255	up	2.573804	up	1.318927	up
Os.15856.1.S1_at	4.5122066	up	1.225762	up	2.312209	up	5.53089	up
OsAffx.30784.1.S1_s_	2.561808	up	4.453168	up	-1.57176	down	11.40817	up
Os.54944.1.S1_at	2.0561829	up	1.373593	up	2.061808	up	2.824358	up
Os.53887.1.S1_at	1.8400815	up	1.904612	up	1.486779	up	3.50464	up
Os.35465.1.S1_at	-4.551216	down	-1.0822	down	-2.61606	down	-4.92533	down

Os.55394.1.S1_at	1.0738586	up	11.51425	up	-4.06732	down	12.36468	up
Os.14862.1.S1_s_at	-2.4348056	down	-1.34099	down	-2.11047	down	-3.26505	down
Os.51385.1.S1_at	1.5216488	up	2.224878	up	1.271903	up	3.385483	up
Os.21367.1.S1_at	2.0940044	up	2.523792	up	1.120645	up	5.284831	up
OsAffx.27219.1.S1_at	5.278482	up	-1.13638	down	3.213869	up	4.645016	up
Os.8763.1.S1_at	1.3450209	up	1.050661	up	2.691558	up	1.413161	up
OsAffx.11919.1.S1_s_	2.044074	up	1.641109	up	1.722634	up	3.354549	up
OsAffx.17948.1.S1_at	-2.6230435	down	-1.46692	down	-1.92652	down	-3.8478	down
Os.6244.1.S1_x_at	3.9459264	up	1.534968	up	1.841001	up	6.056869	up
OsAffx.31541.1.S1_s_	2.314631	up	2.094536	up	1.348689	up	4.848077	up
Os.8266.1.A1_at	2.1545267	up	4.325665	up	-1.5317	down	9.31976	up
Os.12410.1.S1_a_at	-1.2380805	down	1.4417	up	-4.07061	down	1.164464	up
Os.50234.1.S1_at	-2.9146638	down	1.102718	up	-3.11189	down	-2.64316	down
OsAffx.29248.3.S1_s_	-1.1301119	down	-1.23034	down	-2.29254	down	-1.39042	down
Os.7802.1.S1_at	-2.5091202	down	1.118401	up	-3.15275	down	-2.24349	down
OsAffx.14324.1.S1_at	1.7885424	up	2.631223	up	1.071316	up	4.706055	up
Os.11008.1.S1_at	2.091005	up	1.261832	up	2.233202	up	2.638498	up
Os.24471.1.S1_at	1.7099483	up	4.567207	up	-1.62113	down	7.809687	up
OsAffx.11535.1.S1_at	1.2264524	up	-1.19678	down	3.370073	up	1.024794	up
Os.18434.1.S1_at	1.7270386	up	2.104657	up	1.337881	up	3.634824	up
Os.9366.1.S1_at	2.2343583	up	-1.65891	down	4.668963	up	1.346885	up
Os.1852.1.S1_at	2.5860295	up	1.25863	up	2.235946	up	3.254854	up
Os.51300.1.S1_x_at	-2.2962272	down	-1.12709	down	-2.49666	down	-2.58806	down
Os.47946.1.S1_at	5.7666416	up	1.377473	up	2.041006	up	7.943392	up
Os.28424.1.A1_at	-3.9473457	down	1.27863	up	-3.59474	down	-3.08717	down
OsAffx.23104.1.S1_at	-1.3793343	down	-2.78769	down	-1.00823	down	-3.84516	down
Os.40355.1.S1_at	-1.8731942	down	-1.20418	down	-2.33342	down	-2.25567	down
Os.17020.1.S1_x_at	2.6959014	up	2.196437	up	1.278201	up	5.921377	up
Os.8221.1.S1_a_at	3.3084037	up	-1.59368	down	4.473481	up	2.07595	up
Os.34741.1.S1_at	2.4057062	up	1.682778	up	1.66804	up	4.048269	up
Os.37687.1.A1_at	1.3952996	up	-1.00699	down	2.825945	up	1.385618	up
Os.46306.1.S1_x_at	1.2729949	up	-2.39871	down	-1.16986	down	-1.8843	down
Os.37000.2.S1_x_at	1.5926392	up	-1.11527	down	3.129317	up	1.428027	up
OsAffx.32200.1.A1_x_	1.1707363	up	-2.88007	down	1.026736	up	-2.46005	down
OsAffx.12326.1.S1_at	-1.8717674	down	-1.79011	down	-1.56684	down	-3.35068	down
Os.18211.2.S1_x_at	-2.692671	down	1.350418	up	-3.78506	down	-1.99395	down
Os.4149.1.S1_at	2.6175358	up	1.617433	up	1.732836	up	4.233689	up
Os.32597.1.S1_at	3.1829014	up	-3.91507	down	1.397016	up	-1.23003	down
Os.37710.1.S1_at	-2.6449041	down	2.62963	up	-7.36838	down	-1.00581	down
Os.34696.1.S1_at	1.4767631	up	1.152286	up	2.430736	up	1.701653	up
Os.51092.1.S1_at	-1.4648305	down	-1.2819	down	3.59044	up	-1.87777	down
Os.47707.1.A1_s_at	-3.3028271	down	-1.0478	down	-2.67275	down	-3.46069	down
Os.33834.1.S1_at	-2.5660915	down	-1.0997	down	-2.54641	down	-2.82193	down
Os.1233.1.S1_at	4.6979423	up	-1.45153	down	4.061584	up	3.236548	up
OsAffx.820.1.S1_at	-1.7420113	down	-1.28529	down	-2.17594	down	-2.23899	down
Os.22667.1.S1_at	2.5157688	up	1.174656	up	2.380709	up	2.955163	up
Os.47871.1.S1_at	1.0891145	up	-5.6376	down	15.75423	up	-5.17632	down

Os.209.1.S1_at	1.5694436	up	-1.50093	down	4.193875	up	1.045648	up
Os.10340.1.S1_at	3.1851332	up	1.380697	up	2.022021	up	4.397703	up
Os.39933.1.S1_at	-2.3844483	down	-2.108	down	-1.32365	down	-5.02642	down
Os.26967.1.S1_a_at	6.276611	up	-1.33678	down	3.72952	up	4.695338	up
OsAffx.10756.1.S1_x_	2.4695327	up	-1.2605	down	3.516633	up	1.959167	up
Os.20892.1.S1_at	1.068365	up	2.129121	up	1.310126	up	2.274678	up
Os.5178.1.A1_s_at	-3.3248742	down	1.115296	up	-3.11024	down	-2.98116	down
Os.34564.1.S1_at	1.2601104	up	1.248996	up	2.232371	up	1.573873	up
Os.18211.1.S1_at	-3.474446	down	1.469125	up	-4.09604	down	-2.36498	down
Os.6723.1.S1_at	3.3613675	up	1.863438	up	1.496182	up	6.263698	up
Os.7612.1.S1_at	-1.9350924	down	-1.61668	down	-1.72359	down	-3.12842	down
Os.38297.1.S1_a_at	2.6771777	up	1.405542	up	1.981784	up	3.762885	up
Os.44475.1.S1_at	7.167726	up	2.794231	up	-1.00328	down	20.02828	up
Os.5665.1.S1_at	-3.3811913	down	-1.5065	down	-1.84867	down	-5.09376	down
Os.56275.1.S1_x_at	2.89018	up	-1.21514	down	3.384145	up	2.378479	up
Os.11183.1.S1_s_at	-1.3336277	down	5.348746	up	-1.92164	down	4.010674	up
Os.37909.1.S1_at	-2.1567924	down	-1.2065	down	-2.30444	down	-2.60217	down
Os.27569.1.S2_at	3.070574	up	1.187736	up	2.340261	up	3.647033	up
Os.54493.1.S1_at	1.9081476	up	1.995218	up	1.39312	up	3.80717	up
Os.36435.1.S1_at	-3.558115	down	-1.10075	down	-2.52284	down	-3.91659	down
Os.5581.1.S1_at	2.6859224	up	1.050488	up	2.640722	up	2.82153	up
Os.2117.2.S1_x_at	3.457866	up	1.326607	up	2.090495	up	4.587228	up
OsAffx.31633.1.S1_s_	-3.8542387	down	1.133852	up	-3.1444	down	-3.39925	down
Os.12804.1.S1_at	2.1795163	up	3.575643	up	-1.28982	down	7.793172	up
Os.26542.1.A1_at	-3.206831	down	1.02305	up	-2.83535	down	-3.13458	down
OsAffx.32257.1.A1_a	-1.0019411	down	-2.1976	down	-1.26069	down	-2.20187	down
Os.14427.1.S1_at	3.3559618	up	-1.1504	down	3.186698	up	2.917221	up
Os.27483.1.S1_at	1.0387019	up	3.702481	up	-1.33733	down	3.845774	up
Os.21068.1.S1_x_at	-4.200315	down	1.119804	up	-3.10005	down	-3.75094	down
OsAffx.17366.1.S1_at	1.1147994	up	2.911963	up	-1.05237	down	3.246254	up
Os.2493.1.S2_at	-2.306877	down	-1.33823	down	-2.06767	down	-3.08713	down
Os.7521.1.S1_at	1.2922901	up	2.879648	up	-1.04076	down	3.721341	up
Os.12029.1.S1_a_at	-1.6408082	down	-1.21843	down	-2.27011	down	-1.99921	down
Os.46872.1.S1_at	3.156351	up	-1.13178	down	3.130131	up	2.788836	up
Os.18839.1.S1_at	-4.727084	down	-1.36599	down	-2.02445	down	-6.45717	down
Os.54083.1.S1_at	2.2599676	up	1.550926	up	1.782249	up	3.505043	up
Os.34841.1.S1_at	3.6651762	up	-1.64557	down	4.546945	up	2.227299	up
Os.10002.1.S1_at	-2.881401	down	-1.05463	down	-2.61708	down	-3.03881	down
OsAffx.2438.1.S1_at	-1.064946	down	1.733719	up	1.591559	up	1.627987	up
Os.26482.1.S1_at	1.2399195	up	1.806074	up	1.527368	up	2.239387	up
Os.33604.1.S1_a_at	-2.1714416	down	-1.67924	down	-1.64187	down	-3.64637	down
Os.28129.1.S1_at	5.0620546	up	-1.17159	down	3.229078	up	4.320659	up
Os.50813.1.S1_at	3.693722	up	-1.34351	down	3.701814	up	2.749314	up
Os.14934.1.S1_at	-3.5223618	down	1.03178	up	-2.84264	down	-3.41387	down
Os.57460.1.S1_x_at	2.682318	up	1.021391	up	2.696837	up	2.739696	up
OsAffx.25498.1.S1_at	2.0003273	up	1.410767	up	1.95245	up	2.821995	up
Os.9306.1.S1_at	2.1784832	up	-1.36907	down	3.769129	up	1.59121	up

Os.6861.1.S1_at	2.8320525	up	1.119445	up	2.459251	up	3.170325	up
Os.25613.1.A1_at	-1.0055242	down	-1.5092	down	4.153893	up	-1.51753	down
Os.11094.1.A1_at	-4.0668907	down	1.62973	up	-4.48421	down	-2.49544	down
Os.47575.1.S1_at	-3.2313044	down	1.094974	up	-3.01173	down	-2.95103	down
Os.46160.2.S1_at	1.0676378	up	3.435324	up	-1.24948	down	3.667682	up
OsAffx.24769.1.S1_s_	-1.576373	down	-2.10856	down	-1.30385	down	-3.32388	down
OsAffx.32313.1.S1_at	1.2195057	up	-1.58619	down	-1.7295	down	-1.30069	down
Os.22573.1.S1_x_at	2.3499398	up	1.122391	up	2.444163	up	2.63755	up
Os.39054.1.A1_at	1.3885115	up	1.247586	up	2.198642	up	1.732288	up
Os.14649.1.S1_at	-3.6228774	down	1.063694	up	-2.91768	down	-3.40594	down
Os.50272.1.S1_s_at	-2.3401866	down	-1.36756	down	-2.00435	down	-3.20033	down
Os.7994.1.S1_at	1.286034	up	1.301955	up	2.104362	up	1.674358	up
Os.49082.1.S1_at	-4.010109	down	1.22734	up	-3.3626	down	-3.26732	down
Os.49588.1.S1_at	2.6473603	up	1.154917	up	2.371727	up	3.057481	up
Os.22312.2.S1_at	-1.7436327	down	-1.44841	down	-1.8907	down	-2.52549	down
Os.8482.1.S1_s_at	2.784924	up	-1.01667	down	2.782786	up	2.739251	up
Os.55575.1.S1_at	1.2636057	up	1.67313	up	1.634078	up	2.114176	up
Os.15580.1.S1_at	-1.1361963	down	-2.4179	down	-1.13073	down	-2.74721	down
Os.8540.1.S1_at	-2.4018643	down	-1.16359	down	-2.34957	down	-2.79478	down
Os.54549.1.S1_at	-3.5249584	down	1.040704	up	-2.84513	down	-3.38709	down
Os.15914.1.S1_at	-2.8865867	down	1.075687	up	-2.93918	down	-2.68348	down
Os.17554.1.S1_at	2.1044946	up	2.410624	up	1.133299	up	5.073146	up
Os.55372.1.S1_x_at	2.0724666	up	1.2579	up	2.17178	up	2.606956	up
Os.8922.1.S1_at	3.9314196	up	-2.39982	down	6.553227	up	1.638212	up
Os.53320.2.S1_s_at	-1.4946647	down	-2.45753	down	-1.1109	down	-3.67319	down
Os.45934.1.S1_at	2.3799152	up	2.476662	up	1.102238	up	5.894245	up
Os.11247.1.S1_at	2.4114897	up	1.653038	up	1.650847	up	3.986283	up
OsAffx.12548.1.S1_at	2.1126707	up	1.517096	up	1.798542	up	3.205123	up
Os.5754.1.S1_at	-2.3954546	down	1.221461	up	-3.3319	down	-1.96114	down
Os.18583.2.S1_at	-1.4552336	down	-1.3549	down	-2.01317	down	-1.9717	down
Os.53254.1.S1_at	2.047966	up	-1.10343	down	3.007607	up	1.856	up
Os.45924.1.S1_x_at	1.8205401	up	-1.36016	down	3.707088	up	1.338472	up
Os.36162.1.S1_at	1.7464563	up	3.421426	up	-1.25561	down	5.975371	up
Os.24690.1.A1_s_at	-4.992638	down	1.339216	up	-3.64714	down	-3.72803	down
Os.12819.1.S1_at	2.2868407	up	-1.12292	down	3.055369	up	2.036514	up
AFFX-Os-r2-Bs-dap-5_	-1.5492867	down	1.094832	up	-2.97893	down	-1.41509	down
Os.23778.1.S1_at	2.3243408	up	1.728271	up	1.574162	up	4.017091	up
Os.12856.1.S1_x_at	-2.3637462	down	-1.81092	down	-1.50228	down	-4.28055	down
Os.6080.1.S1_at	1.6924994	up	-1.35399	down	3.682997	up	1.250009	up
Os.9859.1.S1_at	-1.6859205	down	-2.78936	down	1.025845	up	-4.70265	down
Os.11195.1.S1_at	4.1275682	up	1.252867	up	2.169894	up	5.171294	up
Os.18587.1.S1_at	4.244161	up	1.006972	up	2.699678	up	4.273754	up
Os.7854.1.S1_at	2.2859578	up	1.262228	up	2.152692	up	2.8854	up
Os.4404.1.S1_s_at	-2.154154	down	1.236032	up	-3.35655	down	-1.7428	down
Os.25287.1.A1_at	-1.1369253	down	2.10053	up	1.292513	up	1.847553	up
Os.22076.1.S1_a_at	2.4241614	up	2.575191	up	1.054162	up	6.242679	up
Os.55760.1.S1_at	1.5580835	up	1.085479	up	2.499459	up	1.691268	up

Os.48873.1.S1_at	1.2057867	up	-1.1524	down	3.125876	up	1.04633	up
Os.16058.1.S1_at	2.6762595	up	-1.165	down	3.15976	up	2.297225	up
Os.47932.1.S1_at	-4.3999763	down	1.059376	up	-2.87247	down	-4.15337	down
Os.51464.1.A1_at	-2.1346169	down	1.144796	up	-3.10281	down	-1.86463	down
Os.11053.1.S1_s_at	2.5077872	up	-2.12077	down	5.747758	up	1.182489	up
Os.29966.1.S1_at	-3.690384	down	1.33538	up	-3.61838	down	-2.76355	down
Os.7945.2.S1_s_at	3.4669466	up	1.099845	up	2.463025	up	3.813103	up
OsAffx.2070.2.S1_at	1.2041177	up	2.873614	up	-1.06089	down	3.460169	up
Os.26687.1.S1_at	-3.3448205	down	1.059333	up	-2.86782	down	-3.15748	down
OsAffx.26679.1.S1_at	-1.7901458	down	-2.01896	down	-1.33981	down	-3.61424	down
Os.50249.2.S1_at	-1.2005259	down	2.260814	up	1.196287	up	1.883187	up
Os.1978.1.S1_at	2.1419277	up	2.030006	up	1.332004	up	4.348126	up
Os.11757.2.S1_at	2.9621012	up	2.262519	up	1.194838	up	6.701811	up
AFFX-DapX-5_at	-1.5707601	down	1.112496	up	-3.0074	down	-1.41192	down
Os.52628.1.A1_at	-2.1569302	down	-1.18387	down	-2.2834	down	-2.55352	down
Os.54417.1.S1_at	1.4301448	up	2.170882	up	1.244784	up	3.104676	up
Os.46029.1.A1_at	-2.1952298	down	-1.50034	down	-1.80084	down	-3.29359	down
Os.28110.3.S1_at	1.4909412	up	2.819032	up	-1.04352	down	4.20301	up
Os.42796.1.S1_at	-4.122072	down	-1.048	down	-2.57728	down	-4.31992	down
Os.16187.1.S1_at	-3.0697837	down	-1.10421	down	-2.44542	down	-3.38969	down
Os.24787.1.S1_at	1.7841643	up	1.026584	up	2.630112	up	1.831595	up
Os.49582.1.S1_at	-2.6825116	down	-2.24092	down	-1.20414	down	-6.01131	down
Os.50766.1.S1_at	1.6687499	up	-1.00336	down	2.705824	up	1.66316	up
Os.49290.1.S1_at	2.2713892	up	1.681235	up	1.603363	up	3.81874	up
Os.34161.1.S1_at	1.5318328	up	-1.00822	down	2.717459	up	1.519339	up
Os.2426.1.A1_at	1.9628876	up	1.90219	up	1.416863	up	3.733785	up
Os.38044.1.S1_at	-4.073765	down	1.612951	up	-4.34646	down	-2.52566	down
OsAffx.16770.1.S1_at	4.889234	up	-1.1847	down	3.191107	up	4.126991	up
Os.13866.1.S1_at	-1.4587818	down	-1.47173	down	-1.82946	down	-2.14693	down
Os.27538.1.S1_at	1.815163	up	3.337585	up	-1.2398	down	6.05826	up
Os.17542.1.S1_at	-2.9994147	down	1.422426	up	-3.82733	down	-2.10866	down
OsAffx.19953.1.S1_x_	-1.1937736	down	-1.59196	down	-1.68981	down	-1.90043	down
OsAffx.12379.1.S1_at	1.06898	up	2.098317	up	1.281661	up	2.243059	up
Os.6632.1.S1_at	1.3076383	up	1.955288	up	1.374825	up	2.556809	up
Os.45897.1.S1_s_at	5.364145	up	1.112526	up	2.416216	up	5.967752	up
Os.25130.1.S1_at	3.5278337	up	-1.50056	down	4.033435	up	2.351017	up
Os.53444.1.S1_at	2.2013443	up	1.45341	up	1.849276	up	3.199456	up
Os.17546.2.S1_x_at	1.5343862	up	1.212911	up	2.215426	up	1.861074	up
Os.24051.2.S1_at	2.2593098	up	2.918511	up	-1.08654	down	6.593821	up
Os.53320.1.S1_s_at	-1.6021596	down	-2.51935	down	-1.0654	down	-4.0364	down
Os.43344.1.S1_at	2.7730017	up	1.478487	up	1.814901	up	4.099849	up
Os.10547.1.S1_at	2.2159739	up	1.563754	up	1.715517	up	3.465239	up
Os.17125.1.S1_at	-2.7217932	down	1.063755	up	-2.85338	down	-2.55867	down
Os.48131.1.S1_s_at	2.1599596	up	1.895568	up	1.414594	up	4.094349	up
Os.1182.1.S1_x_at	5.802229	up	-1.2991	down	3.483366	up	4.466329	up
OsAffx.23225.1.S1_x_	5.871944	up	-1.93535	down	5.188646	up	3.034045	up
Os.55582.1.S1_at	3.1006224	up	1.419589	up	1.888081	up	4.401608	up

Os.36834.1.S1_at	2.2110105	up	-1.28121	down	3.432381	up	1.725722	up
Os.26705.1.S1_at	1.6361283	up	1.204256	up	2.222744	up	1.970318	up
Os.26879.1.S1_at	2.0206647	up	1.899804	up	1.407904	up	3.838867	up
Os.13708.2.A1_at	6.8081427	up	-1.52278	down	4.071291	up	4.470872	up
Os.51578.1.S1_x_at	3.5562449	up	2.707378	up	-1.0128	down	9.628099	up
Os.15776.1.S1_at	3.874745	up	1.210577	up	2.207111	up	4.690678	up
Os.18374.1.A1_at	2.7714918	up	-1.21677	down	3.251039	up	2.277749	up
Os.16627.1.S1_at	2.4091754	up	2.131509	up	1.2532	up	5.135179	up
Os.27159.1.S1_at	1.6232102	up	2.659432	up	1.004381	up	4.316818	up
Os.53993.2.S1_at	1.8379071	up	1.081343	up	2.469727	up	1.987408	up
Os.54340.1.S1_at	-2.7491326	down	1.212588	up	-3.23788	down	-2.26716	down
Os.53787.1.S1_at	-3.9095564	down	1.427647	up	-3.8097	down	-2.73846	down
Os.32013.2.S1_at	1.4655645	up	1.412718	up	1.888796	up	2.07043	up
Os.36593.1.S1_at	-1.6120509	down	1.802988	up	1.478859	up	1.118444	up
Os.52261.1.S1_at	1.3014781	up	6.407114	up	-2.40339	down	8.338718	up
OsAffx.22608.1.S1_at	-1.8937201	down	-1.30173	down	-2.0471	down	-2.46512	down
Os.46345.1.A1_at	1.6408505	up	5.035475	up	-1.89127	down	8.262461	up
Os.11987.1.S1_at	-1.9316801	down	1.144325	up	-3.04531	down	-1.68805	down
Os.17323.2.S1_at	3.0741143	up	1.372509	up	1.938925	up	4.219251	up
Os.8423.1.S1_a_at	-3.3459144	down	-1.00019	down	-2.66068	down	-3.34655	down
Os.55372.1.S1_at	2.253372	up	1.154182	up	2.304969	up	2.600801	up
Os.50276.1.S1_at	1.5533733	up	1.274584	up	2.086071	up	1.979905	up
Os.51525.1.S1_at	-1.5219297	down	-1.53927	down	-1.72724	down	-2.34266	down
Os.4614.1.S1_a_at	-1.1578244	down	1.988991	up	1.336414	up	1.717869	up
Os.17396.1.S1_at	4.050138	up	-1.03883	down	2.760157	up	3.898758	up
Os.53814.1.S1_at	1.774762	up	6.433999	up	-2.42208	down	11.41882	up
Os.51629.1.S1_at	-2.9962182	down	-1.37393	down	-1.93342	down	-4.11659	down
Os.23349.2.S1_at	-2.0707152	down	-1.29012	down	-2.0577	down	-2.67146	down
Os.22577.1.S1_x_at	-2.7132833	down	1.83151	up	-4.8612	down	-1.48145	down
OsAffx.5607.1.S1_x_a	6.0941973	up	1.437691	up	1.845705	up	8.761576	up
OsAffx.1590.1.S1_x_a	-1.2493913	down	-2.11122	down	-1.2565	down	-2.63774	down
Os.8456.1.S1_at	3.464858	up	2.526011	up	1.05003	up	8.75227	up
Os.1193.2.S1_at	1.3081808	up	1.666014	up	1.591917	up	2.179448	up
Os.26802.2.S1_at	-1.0432456	down	1.989119	up	1.333149	up	1.906665	up
Os.9293.1.S1_at	-2.254428	down	1.107108	up	-2.933	down	-2.03632	down
Os.4631.1.S1_at	-1.9414334	down	1.276643	up	-3.38119	down	-1.52073	down
Os.51582.1.S1_at	-3.602036	down	-1.37037	down	-1.93228	down	-4.93614	down
Os.14496.1.S1_at	-3.1634579	down	1.367555	up	-3.62101	down	-2.31322	down
Os.15682.1.A1_at	3.9128027	up	1.001658	up	2.64326	up	3.919289	up
Os.22590.1.A1_at	1.9475487	up	2.626884	up	1.007806	up	5.115984	up
Os.7934.1.S1_at	-3.6412694	down	1.302391	up	-3.44725	down	-2.79584	down
Os.20476.2.S1_a_at	-3.9471376	down	1.198733	up	-3.17274	down	-3.29276	down
OsAffx.23594.1.S1_at	4.8154793	up	-1.26398	down	3.345417	up	3.80978	up
Os.7478.1.S1_at	1.2378001	up	-1.11603	down	2.953776	up	1.109109	up
Os.148.1.S1_at	2.602225	up	-1.17218	down	3.102099	up	2.219982	up
Os.39944.1.S1_at	1.505042	up	-1.10879	down	2.934004	up	1.357377	up
Os.10873.1.S1_at	1.8215023	up	-1.24149	down	3.282948	up	1.46719	up

OsAffx.24862.1.S1_at	8.871983	up	-1.70758	down	4.513506	up	5.195651	up
Os.55293.1.S1_at	2.747486	up	2.137074	up	1.236729	up	5.871581	up
OsAffx.2386.1.S1_at	1.9727688	up	1.600801	up	1.650884	up	3.158011	up
Os.55778.1.S1_at	-3.495399	down	1.029114	up	-2.71923	down	-3.39651	down
Os.20644.1.S1_at	-2.1870189	down	-1.78665	down	-1.47863	down	-3.90744	down
Os.50906.1.S1_at	1.0866945	up	1.523138	up	1.734331	up	1.655185	up
Os.20292.1.S1_s_at	-1.8499244	down	-1.23355	down	-2.14122	down	-2.28198	down
Os.55991.1.S1_s_at	1.7196921	up	1.378481	up	1.915699	up	2.370562	up
OsAffx.16152.1.S1_at	-3.4272676	down	-1.23664	down	-2.13518	down	-4.23829	down
Os.17215.1.S1_at	-2.4137936	down	1.236741	up	-3.26423	down	-1.95174	down
Os.23145.1.S1_at	-2.9366155	down	-1.06741	down	-2.47211	down	-3.13457	down
Os.3419.1.S1_a_at	14.782467	up	-7.23232	down	19.08136	up	2.043944	up
Os.7505.2.S1_at	1.9863138	up	1.654898	up	1.592934	up	3.287147	up
Os.28435.4.S1_at	-1.2605879	down	-2.39499	down	-1.10056	down	-3.0191	down
Os.28300.2.S2_s_at	2.2374785	up	1.141857	up	2.307153	up	2.554881	up
Os.51760.1.S1_at	1.5553259	up	1.511623	up	1.742158	up	2.351066	up
OsAffx.24777.1.S1_at	1.6712413	up	-1.38753	down	3.653507	up	1.204469	up
Os.40030.1.S1_s_at	-1.2842114	down	-2.02463	down	-1.30045	down	-2.60006	down
OsAffx.32113.1.S1_at	-3.0131469	down	-1.37136	down	-1.91992	down	-4.13212	down
Os.47778.1.A1_s_at	3.0546684	up	2.708774	up	-1.02883	down	8.274407	up
OsAffx.32223.1.S1_x_	-1.5311848	down	-1.34294	down	-1.95948	down	-2.05629	down
Os.6752.1.S1_at	1.8201574	up	1.718068	up	1.530934	up	3.127154	up
Os.15679.1.S1_s_at	-1.5329657	down	1.270187	up	-3.34083	down	-1.20688	down
OsAffx.15438.1.S1_at	2.8280017	up	-1.63344	down	4.295287	up	1.731317	up
Os.55617.1.S1_at	2.6988194	up	1.155502	up	2.27565	up	3.118492	up
Os.51029.1.S1_at	-2.1545372	down	2.304693	up	-6.05936	down	1.069693	up
Os.15007.1.S1_at	-2.1335604	down	1.308652	up	-3.44035	down	-1.63035	down
Os.6671.2.S1_x_at	3.1613815	up	1.217318	up	2.15876	up	3.848407	up
OsAffx.15920.1.S1_at	13.46445	up	-1.27105	down	3.338383	up	10.59316	up
Os.10425.1.S1_at	-1.9651151	down	-1.3922	down	-1.88655	down	-2.73584	down
Os.32071.1.S1_at	2.8852124	up	-1.54185	down	4.04827	up	1.871266	up
Os.18724.1.S1_at	1.6156249	up	7.136817	up	-2.7195	down	11.53042	up
Os.10686.1.S1_at	1.9936804	up	1.559466	up	1.682717	up	3.109077	up
Os.13835.2.S1_a_at	3.1702058	up	3.769985	up	-1.43698	down	11.95163	up
Os.49337.1.S1_at	-2.7537546	down	1.237938	up	-3.24579	down	-2.22447	down
Os.14366.1.S1_at	1.27355	up	2.849116	up	-1.08675	down	3.628491	up
Os.9338.1.S1_at	3.6801846	up	-1.17851	down	3.089321	up	3.122734	up
Os.17609.1.S1_at	2.2076237	up	1.032481	up	2.538448	up	2.27933	up
Os.27797.1.A1_at	-1.1832879	down	-1.30201	down	3.412315	up	-1.54065	down
Os.53368.1.S1_at	1.9703459	up	-1.45858	down	3.819486	up	1.350868	up
Os.7631.1.S1_at	1.2225952	up	1.114262	up	2.350027	up	1.362291	up
Os.20175.1.A1_x_at	-2.2520053	down	1.735402	up	-4.54334	down	-1.29769	down
Os.17814.2.S1_x_at	1.4304944	up	1.07962	up	2.424489	up	1.544391	up
Os.33238.1.S1_x_at	6.407965	up	-1.09817	down	2.874294	up	5.835106	up
OsAffx.12587.1.S1_s_	1.2295544	up	1.104781	up	2.369006	up	1.358389	up
Os.24580.2.A1_at	-9.207002	down	3.043457	up	-7.96431	down	-3.02518	down
Os.10401.1.S1_s_at	-1.4492654	down	3.417646	up	-1.30608	down	2.358192	up

Os.47388.1.S1_s_at	2.0963364	up	1.657928	up	1.578064	up	3.475575	up
Os.27154.1.S1_at	-1.4927934	down	2.150523	up	1.216045	up	1.440603	up
Os.17536.1.S1_at	3.93736	up	-1.41921	down	3.710181	up	2.774336	up
Os.50527.1.S1_a_at	1.4600676	up	1.533888	up	1.703812	up	2.23958	up
Os.21957.2.S1_at	-2.2957518	down	1.673571	up	-4.371	down	-1.37177	down
Os.4291.1.S1_at	-2.9919724	down	1.092635	up	-2.85204	down	-2.73831	down
Os.9489.1.S1_x_at	-2.9177818	down	1.30702	up	-3.41109	down	-2.23239	down
Os.22928.1.S1_x_at	4.435992	up	-1.25251	down	3.268599	up	3.541682	up
Os.8294.1.S1_at	2.2874792	up	2.511548	up	1.039023	up	5.745112	up
Os.20421.1.S1_at	2.4666715	up	-1.75158	down	4.570359	up	1.408259	up
Os.24865.1.A1_at	1.6607907	up	16.90237	up	-6.47914	down	28.0713	up
Os.46107.1.S1_s_at	-4.3428807	down	1.253707	up	-3.27057	down	-3.46403	down
OsAffx.3113.1.S1_s_a	3.6491299	up	2.120417	up	1.228904	up	7.737677	up
Os.38354.1.S1_x_at	-4.3012624	down	1.016041	up	-2.64691	down	-4.23336	down
Os.56047.1.S1_at	1.8259536	up	1.133064	up	2.29881	up	2.068923	up
Os.35365.1.S1_at	1.7778851	up	5.236086	up	-2.01064	down	9.309159	up
Os.27522.1.S1_x_at	-1.8715327	down	-1.16572	down	-2.23344	down	-2.18168	down
Os.26735.1.S1_at	1.5062034	up	1.920682	up	1.354962	up	2.892939	up
Os.8532.1.S1_at	-2.198916	down	1.178169	up	-3.06554	down	-1.86638	down
Os.49446.1.S1_at	-2.5724964	down	-1.75593	down	-1.48116	down	-4.51713	down
Os.7850.1.S1_at	2.5043926	up	10.584	up	-4.07026	down	26.5065	up
Os.10179.1.S1_at	3.359702	up	3.932248	up	-1.51229	down	13.21118	up
Os.7939.1.S1_at	1.2050892	up	1.441684	up	1.80303	up	1.737358	up
Os.21563.1.S2_at	4.048606	up	1.020077	up	2.546955	up	4.12989	up
Os.5577.1.S1_at	-1.813609	down	-1.33077	down	-1.9516	down	-2.4135	down
Os.11894.1.S1_at	2.220238	up	2.034509	up	1.276344	up	4.517094	up
Os.46151.1.S1_at	2.4062457	up	-1.35732	down	3.522998	up	1.772792	up
Os.49213.1.S1_at	3.0322475	up	-1.01005	down	2.621189	up	3.002077	up
Os.51741.1.S1_at	3.0546734	up	1.009655	up	2.568663	up	3.084165	up
Os.37593.1.S1_a_at	-1.4000001	down	-1.40738	down	-1.84124	down	-1.97033	down
Os.10772.1.S1_at	-1.2175143	down	-1.16468	down	-2.2239	down	-1.41801	down
OsAffx.28001.1.S1_at	2.3710747	up	-1.16136	down	3.008035	up	2.041639	up
Os.47708.1.A1_at	-3.220328	down	1.230816	up	-3.18775	down	-2.61642	down
OsAffx.25245.1.S1_at	1.6646934	up	-1.42452	down	3.689336	up	1.168603	up
Os.47292.1.S1_at	1.66113	up	1.495197	up	1.731889	up	2.483717	up
Os.47735.1.S1_at	-1.7693934	down	-3.29677	down	1.273557	up	-5.83328	down
Os.47907.1.S1_at	-2.486221	down	-1.14124	down	-2.26723	down	-2.83737	down
Os.51394.1.S1_at	2.2149498	up	-2.54674	down	6.587854	up	-1.14979	down
Os.10546.1.S1_s_at	4.2874346	up	-1.03115	down	2.665958	up	4.157909	up
Os.6354.1.S1_s_at	1.6599365	up	3.380804	up	-1.30767	down	5.611921	up
OsAffx.27605.1.S1_s_	1.2004701	up	2.656675	up	-1.02859	down	3.189259	up
Os.12851.1.S1_at	1.6124827	up	5.362499	up	-2.07697	down	8.646936	up
Os.8147.1.S1_a_at	2.0621328	up	-1.61419	down	4.165147	up	1.277502	up
Os.19006.1.S1_a_at	1.7097915	up	1.180413	up	2.185678	up	2.01826	up
Os.13236.1.S1_at	1.6532434	up	1.597465	up	1.615042	up	2.640999	up
Os.16068.2.S1_at	-3.862512	down	-1.21493	down	-2.12324	down	-4.69266	down
Os.5154.1.S1_at	-1.0357907	down	3.169306	up	-1.22935	down	3.059794	up

Os.46902.1.S1_at	-1.0667311	down	1.756075	up	1.467941	up	1.646221	up
Os.15938.1.S1_at	6.8505015	up	-6.84953	down	17.65084	up	1.000142	up
Os.16422.1.S1_s_at	1.9321035	up	2.635738	up	-1.02287	down	5.092519	up
Os.26059.1.S1_at	-1.9400342	down	-1.21563	down	-2.11953	down	-2.35836	down
Os.11107.1.S1_at	2.4849386	up	1.047497	up	2.458586	up	2.602965	up
Os.16976.1.S1_at	-1.9552678	down	-1.03994	down	-2.47424	down	-2.03335	down
Os.8845.1.S1_at	-2.0993032	down	-1.26415	down	-2.03449	down	-2.65384	down
Os.11271.1.S1_a_at	-2.9259489	down	-1.00137	down	-2.56716	down	-2.92997	down
Os.11757.1.S1_at	2.662909	up	1.566969	up	1.639336	up	4.172695	up
Os.30597.1.S1_at	5.8544564	up	-1.26223	down	3.241985	up	4.638199	up
Os.27668.1.S1_at	-2.0259588	down	-1.36798	down	-1.87752	down	-2.77147	down
Os.16903.1.A1_at	1.533174	up	2.936003	up	-1.1432	down	4.501404	up
Os.14652.1.S1_at	1.5023745	up	1.283361	up	2.00099	up	1.928089	up
Os.9654.2.S1_x_at	2.8042178	up	-1.03144	down	2.64811	up	2.718736	up
Os.27465.1.S1_at	2.252291	up	2.300225	up	1.116025	up	5.180775	up
Os.5690.1.S1_at	1.6231943	up	1.3293	up	1.930895	up	2.157713	up
Os.41164.1.S1_at	-2.9369314	down	1.333617	up	-3.42235	down	-2.20223	down
Os.53632.1.S1_at	1.2155699	up	-1.77154	down	4.544385	up	-1.45737	down
Os.7338.1.S1_at	2.0308108	up	1.440697	up	1.780134	up	2.925782	up
Os.49797.1.A1_at	-3.3466032	down	1.226794	up	-3.14557	down	-2.72793	down
Os.17679.1.S1_at	2.1784618	up	-1.38747	down	3.556558	up	1.5701	up
Os.25687.1.S1_x_at	1.1005359	up	5.304674	up	-2.07021	down	5.837984	up
Os.46864.1.S1_at	1.1873665	up	1.444162	up	1.773989	up	1.714749	up
Os.9053.1.S1_at	2.2411008	up	1.341172	up	1.910003	up	3.005701	up
Os.20579.1.S1_at	-3.6010122	down	1.33874	up	-3.4289	down	-2.68985	down
Os.12535.1.S1_at	3.0731807	up	1.642658	up	1.559186	up	5.048184	up
Os.12412.1.S1_at	2.6218472	up	1.530481	up	1.672496	up	4.012688	up
Os.8025.1.S1_at	1.596329	up	1.555305	up	1.645068	up	2.482778	up
Os.30516.1.S1_at	-2.921938	down	1.294249	up	-3.31069	down	-2.25763	down
Os.30998.1.S1_at	1.5237292	up	2.779407	up	-1.08698	down	4.235063	up
Os.18696.1.S1_at	1.9158642	up	1.8455	up	1.385317	up	3.535727	up
Os.27186.1.S1_at	-1.1426126	down	2.96079	up	-1.15836	down	2.591246	up
Os.17909.1.S1_a_at	-1.6243156	down	-1.79994	down	-1.41983	down	-2.92366	down
Os.14762.2.S1_s_at	-3.5744677	down	-1.06832	down	-2.39141	down	-3.81866	down
Os.51696.1.S1_at	1.2368455	up	-1.42882	down	3.649183	up	-1.15521	down
OsAffx.20034.1.S1_x_	-1.7541507	down	1.020028	up	-2.6046	down	-1.71971	down
Os.18851.2.A1_at	1.2485217	up	2.609593	up	-1.02241	down	3.258134	up
Os.9945.1.S1_at	-1.5122945	down	1.008758	up	-2.57429	down	-1.49917	down
Os.17347.1.S1_at	-1.7430643	down	-1.18588	down	-2.15163	down	-2.06706	down
Os.28427.1.S2_a_at	-1.1371139	down	2.492795	up	1.023505	up	2.192212	up
OsAffx.32349.1.A1_a	1.6150895	up	-2.79586	down	1.095873	up	-1.73109	down
OsAffx.20913.1.S1_a	-1.53762	down	-1.16166	down	-2.19606	down	-1.78619	down
Os.32943.1.S1_at	1.074514	up	-7.05141	down	17.98376	up	-6.56242	down
Os.22631.1.S1_a_at	1.9844168	up	3.572011	up	-1.40069	down	7.088357	up
Os.51807.1.S1_at	2.0014927	up	-1.16407	down	2.968393	up	1.719398	up
Os.24092.1.S1_at	1.6701126	up	-1.15448	down	2.943517	up	1.446632	up
Os.9549.1.S1_at	-3.8284159	down	-1.19889	down	-2.12639	down	-4.58986	down

OsAffx.23983.1.S1_at	-2.75677	down	-1.5943	down	-1.5977	down	-4.39513	down
Os.52287.2.S1_at	-3.125232	down	-1.0395	down	-2.44928	down	-3.24868	down
Os.26764.1.A1_at	1.7703412	up	1.777695	up	1.432108	up	3.147126	up
Os.27528.1.S1_at	-1.6467217	down	-1.19458	down	-2.13112	down	-1.96714	down
Os.16025.1.S1_s_at	3.33481	up	-1.04716	down	2.665248	up	3.184615	up
Os.55495.1.S1_at	1.6068429	up	-1.18384	down	3.010771	up	1.357313	up
OsAffx.32221.1.A1_s_	1.0205652	up	-2.95215	down	1.161198	up	-2.89266	down
Os.18156.1.S1_at	-2.1220057	down	-1.4872	down	-1.70847	down	-3.15585	down
Os.13965.1.S1_at	2.1455216	up	1.249386	up	2.033409	up	2.680584	up
Os.7938.1.S1_at	-1.2092142	down	-1.39569	down	-1.81915	down	-1.68769	down
Os.22341.1.S1_at	1.9800531	up	2.317436	up	1.095367	up	4.588646	up
Os.49853.1.S1_a_at	-2.4348562	down	1.078488	up	-2.73624	down	-2.25766	down
Os.22312.3.A1_a_at	-3.5645869	down	-1.14619	down	-2.21344	down	-4.08568	down
Os.10695.1.S1_at	3.791271	up	1.406795	up	1.803353	up	5.333542	up
Os.55961.1.S1_at	12.976782	up	1.269703	up	1.998004	up	16.47666	up
Os.17325.1.S1_at	1.6256531	up	2.709215	up	-1.06797	down	4.404244	up
OsAffx.17387.1.S1_at	1.7070456	up	1.367567	up	1.85422	up	2.3345	up
Os.37103.1.S1_at	1.8585765	up	1.389579	up	1.824641	up	2.582638	up
Os.8911.1.S1_at	1.1762475	up	1.285207	up	1.972451	up	1.511721	up
Os.25589.3.S1_x_at	-1.6485465	down	-1.32499	down	-1.91322	down	-2.1843	down
Os.10576.1.S1_at	1.8241568	up	12.30746	up	-4.8553	down	22.45074	up
Os.17424.1.S1_x_at	-2.5576274	down	1.121448	up	-2.8426	down	-2.28065	down
Os.50801.1.S1_at	-2.1761875	down	1.004163	up	-2.54529	down	-2.16717	down
Os.9694.1.S1_at	-1.5495448	down	-1.42395	down	-1.7797	down	-2.20648	down
Os.23052.1.S1_at	-2.4746356	down	1.32985	up	-3.36943	down	-1.86084	down
Os.1411.1.S1_at	-2.408382	down	-1.4479	down	-1.74914	down	-3.48709	down
Os.35149.1.S1_at	-1.7595525	down	1.632913	up	1.550111	up	-1.07755	down
Os.20977.1.S1_at	1.2591445	up	1.493481	up	1.693957	up	1.880509	up
Os.52293.1.S1_at	-3.2034986	down	-1.17915	down	-2.14518	down	-3.77741	down
Os.53101.1.S1_at	2.015733	up	-4.99687	down	1.976086	up	-2.47894	down
Os.11714.1.S1_at	1.2532398	up	1.357703	up	1.862095	up	1.701528	up
Os.7675.1.S1_at	3.265491	up	-1.49462	down	3.777221	up	2.184831	up
Os.57436.1.S1_at	-2.9875968	down	-1.52874	down	-1.6525	down	-4.56726	down
Os.54150.1.S1_at	1.9649673	up	-1.05149	down	2.655376	up	1.868745	up
Os.411.1.S1_at	4.345488	up	-2.48278	down	6.269286	up	1.750253	up
Os.51529.1.S1_at	2.2550006	up	1.913549	up	1.319404	up	4.315054	up
Os.27954.1.A1_at	-1.6487225	down	-1.63936	down	-1.53991	down	-2.70285	down
OsAffx.17463.1.S1_at	3.0370414	up	-1.10389	down	2.786121	up	2.751227	up
Os.23973.1.A1_at	-1.7999871	down	-1.20744	down	-2.08977	down	-2.17337	down
Os.52554.1.S1_at	-2.1966286	down	-1.53371	down	-1.64489	down	-3.36899	down
OsAffx.12017.1.S1_at	2.2234805	up	-1.31769	down	3.322141	up	1.687402	up
Os.5592.1.S1_at	2.6968257	up	1.074126	up	2.345982	up	2.89673	up
Os.33787.1.S1_at	2.7963111	up	1.467395	up	1.717063	up	4.103292	up
Os.11143.1.S1_at	1.1085775	up	1.384374	up	1.819995	up	1.534686	up
Os.6863.1.S1_at	1.3374676	up	11.07103	up	-4.39439	down	14.80714	up
Os.26967.1.S2_at	5.7952027	up	-1.37923	down	3.473994	up	4.201765	up
Os.5943.1.S1_at	1.557579	up	3.436557	up	-1.36456	down	5.35271	up

Os.20722.3.A1_a_at	-3.052047	down	-1.07343	down	-2.3456	down	-3.27616	down
Os.18678.1.S1_at	-1.9027194	down	-1.41487	down	-1.77932	down	-2.69209	down
Os.23415.1.A1_s_at	1.2190094	up	1.162008	up	2.165092	up	1.416499	up
Os.19547.1.S1_at	-1.2681812	down	-2.33142	down	-1.07908	down	-2.95667	down
OsAffx.32313.1.S1_x_	1.1767138	up	-1.49215	down	-1.68567	down	-1.26807	down
Os.4893.1.S1_at	1.6303647	up	1.345655	up	1.868194	up	2.193908	up
Os.55303.1.S1_at	-1.4447773	down	-1.02786	down	2.583189	up	-1.48503	down
Os.9481.1.S1_at	-3.4549723	down	2.16471	up	-5.43992	down	-1.59604	down
OsAffx.31856.1.S1_at	1.2098316	up	-1.7083	down	4.292659	up	-1.41201	down
Os.53619.1.S1_at	1.7396928	up	-1.24256	down	3.121679	up	1.400092	up
Os.11192.1.S1_at	7.727414	up	1.124958	up	2.232925	up	8.693018	up
OsAffx.31754.1.S1_x_	-1.4971492	down	-1.80959	down	-1.38774	down	-2.70922	down
Os.21563.1.S1_a_at	3.4601364	up	1.345462	up	1.865477	up	4.655483	up
Os.11770.1.S1_at	-2.3555408	down	2.153449	up	-5.40434	down	-1.09385	down
Os.12864.1.S1_a_at	-1.5658386	down	-1.1282	down	-2.22439	down	-1.76658	down
OsAffx.32219.1.S1_x_	1.0269638	up	-1.3446	down	-1.86634	down	-1.3093	down
Os.49639.1.S1_at	1.3323424	up	-11.9129	down	29.89466	up	-8.94134	down
Os.5371.1.S1_s_at	-2.092236	down	1.227164	up	-3.07948	down	-1.70494	down
Os.9775.1.S1_at	-1.178223	down	2.94638	up	-1.17413	down	2.500698	up
Os.7348.1.S1_a_at	1.799104	up	1.883792	up	1.332098	up	3.389139	up
Os.47453.1.A1_s_at	-1.6221755	down	-1.13171	down	-2.21573	down	-1.83583	down
Os.15430.1.A1_at	2.0134578	up	1.035555	up	2.421252	up	2.085046	up
Os.34960.1.S1_at	2.3502073	up	1.298897	up	1.929982	up	3.052678	up
Os.36767.1.S1_at	1.981937	up	1.166294	up	2.149315	up	2.311521	up
Os.7645.1.S1_at	1.6807377	up	1.384592	up	1.809237	up	2.327135	up
Os.22886.1.A1_at	1.3761882	up	1.16438	up	2.151378	up	1.602406	up
Os.9172.2.S1_x_at	-1.6953708	down	1.129567	up	-2.82882	down	-1.5009	down
Os.50024.1.S1_at	-2.245542	down	-1.41649	down	-1.76758	down	-3.18078	down
Os.23482.1.S1_at	1.8687286	up	3.165574	up	-1.26451	down	5.915597	up
Os.50961.1.S1_at	1.125749	up	3.070543	up	-1.2276	down	3.456661	up
Os.5279.1.S1_at	-2.483826	down	-1.80333	down	-1.38687	down	-4.47917	down
OsAffx.16767.1.S1_at	2.6792758	up	-1.37485	down	3.438309	up	1.948775	up
OsAffx.32190.1.A1_x_	1.0675637	up	-1.91903	down	-1.30299	down	-1.79758	down
Os.32108.1.S1_s_at	-1.3845762	down	3.186654	up	-1.27452	down	2.301537	up
Os.20638.1.S1_at	-1.6150331	down	-1.46757	down	-1.70347	down	-2.37017	down
Os.11730.1.S1_at	2.330364	up	1.592271	up	1.569844	up	3.710572	up
Os.4947.1.S1_at	2.4565616	up	1.188567	up	2.102408	up	2.919789	up
OsAffx.19137.1.S1_s_	2.7549632	up	-1.07416	down	2.684151	up	2.564758	up
Os.16712.1.S1_at	3.4409556	up	1.087093	up	2.298209	up	3.740638	up
Os.12979.3.S1_x_at	-1.5296277	down	1.294888	up	-3.23492	down	-1.18128	down
Os.7853.3.S1_at	-2.182765	down	-1.38946	down	-1.79789	down	-3.03286	down
Os.23308.1.A1_at	1.7488419	up	1.20509	up	2.07212	up	2.107513	up
Os.10747.1.S1_s_at	2.684959	up	1.338672	up	1.865017	up	3.59428	up
Os.11098.1.S1_at	1.0937419	up	1.51955	up	1.643015	up	1.661995	up
Os.3048.1.S1_at	-3.000112	down	1.143278	up	-2.85275	down	-2.62413	down
Os.51234.1.S1_at	2.8355901	up	1.072732	up	2.325801	up	3.041828	up
Os.8968.1.S1_x_at	-3.0249228	down	1.271474	up	-3.17224	down	-2.37907	down

OsAffx.2593.1.S1_s_a	-2.0075593	down	-1.50334	down	-1.65901	down	-3.01804	down
Os.53726.1.S1_at	-1.9567562	down	-1.71507	down	-1.45324	down	-3.35597	down
Os.52568.1.S1_s_at	3.5602577	up	-1.42783	down	3.557126	up	2.493466	up
Os.14971.1.S1_at	6.766254	up	4.748384	up	-1.90605	down	32.12877	up
Os.23620.1.A1_a_at	-1.3702811	down	-1.01502	down	-2.45394	down	-1.39086	down
Os.11843.1.S1_at	1.3705543	up	2.979967	up	-1.19648	down	4.084207	up
Os.49832.1.S1_at	-1.6738698	down	1.081403	up	-2.69334	down	-1.54787	down
Os.18033.1.S1_at	2.0346682	up	1.959685	up	1.270368	up	3.987309	up
Os.7603.1.S1_at	-1.5428833	down	-1.70587	down	-1.4592	down	-2.63197	down
OsAffx.18625.2.S1_x_	-1.2107143	down	-1.56002	down	-1.59537	down	-1.88874	down
Os.21778.1.S1_at	1.3979846	up	1.543772	up	1.611896	up	2.158169	up
Os.53017.1.S1_at	1.1743741	up	1.249977	up	1.99058	up	1.46794	up
Os.52475.1.S1_x_at	-2.7807379	down	-1.42345	down	-1.74601	down	-3.95825	down
OsAffx.1869.1.S1_at	-1.6025469	down	1.074063	up	-2.66936	down	-1.49204	down
Os.15619.1.S1_at	-2.4229913	down	-1.38412	down	-1.7953	down	-3.35372	down
Os.21710.1.S1_at	1.6648703	up	2.351129	up	1.056781	up	3.914324	up
OsAffx.22650.1.S1_x_	-2.0722141	down	-1.14489	down	-2.16984	down	-2.37246	down
Os.13649.1.S1_at	1.4916699	up	1.136609	up	2.185471	up	1.695445	up
Os.17533.1.S1_at	2.3871722	up	-1.13032	down	2.80681	up	2.111941	up
Os.7831.1.S1_at	5.0528517	up	-1.92455	down	4.778076	up	2.625477	up
Os.11604.2.S1_at	1.1405244	up	1.349309	up	1.839956	up	1.538919	up
Os.3417.1.S1_at	1.1576923	up	-2.86938	down	7.123366	up	-2.47853	down
Os.52337.1.S1_at	1.7711293	up	1.345276	up	1.844294	up	2.382659	up
Os.7779.1.S1_x_at	1.3079268	up	-1.04552	down	-2.37302	down	1.250986	up
Os.12584.1.S1_x_at	-2.4510272	down	-1.2178	down	-2.03674	down	-2.98487	down
Os.27000.2.S1_x_at	4.1514587	up	-1.83676	down	4.554781	up	2.260205	up
Os.25246.1.A1_at	3.3721385	up	1.187929	up	2.087473	up	4.00586	up
Os.54909.1.S1_at	2.4881756	up	-1.35397	down	3.357051	up	1.837696	up
Os.18082.1.S1_at	2.80423	up	1.317105	up	1.881755	up	3.693466	up
Os.19283.1.S1_s_at	-2.5944307	down	1.378481	up	-3.41645	down	-1.88209	down
Os.26494.1.S1_at	-8.433814	down	1.687003	up	-4.18002	down	-4.99929	down
OsAffx.2280.1.S1_at	1.6671115	up	-2.04241	down	5.06056	up	-1.22512	down
Os.11084.2.S1_x_at	-1.4304839	down	-1.60292	down	-1.54561	down	-2.29295	down
Os.7868.1.S1_at	1.9130062	up	1.074528	up	2.305524	up	2.055579	up
Os.23163.1.S1_at	1.3133807	up	1.435935	up	1.724668	up	1.885929	up
OsAffx.2999.1.S1_at	-1.5619161	down	-1.90898	down	-1.29684	down	-2.98167	down
Os.48959.1.S1_at	2.2380912	up	-1.05944	down	2.622159	up	2.112517	up
Os.49743.1.S1_at	-2.6758707	down	-1.06178	down	-2.33085	down	-2.8412	down
Os.52641.1.S1_at	3.7097387	up	2.675583	up	-1.08131	down	9.925712	up
Os.6165.1.S1_a_at	-2.8113499	down	-1.10679	down	-2.23436	down	-3.11158	down
Os.55651.1.S1_at	2.7405884	up	1.040619	up	2.37621	up	2.851908	up
Os.8833.1.S1_at	2.2232022	up	-1.15268	down	2.848613	up	1.928729	up
Os.53458.1.S1_at	-1.5107571	down	-2.91254	down	1.178603	up	-4.40013	down
Os.4149.2.S1_x_at	2.5093153	up	1.795399	up	1.376313	up	4.505223	up
Os.46758.1.S1_at	1.2496871	up	-1.19361	down	2.948285	up	1.04698	up
Os.10839.1.S1_at	2.1751215	up	1.541562	up	1.602079	up	3.353085	up
Os.16320.1.S1_at	2.5511546	up	1.867669	up	1.32104	up	4.764713	up

Os.23770.1.A1_s_at	1.5990256	up	-3.30331	down	8.149099	up	-2.06583	down
OsAffx.2855.1.S1_at	6.8657374	up	-1.52939	down	3.769433	up	4.489211	up
Os.46884.1.S1_at	5.6083264	up	-1.47168	down	3.627194	up	3.810827	up
Os.30823.1.S1_at	-2.580182	down	1.123673	up	-2.76915	down	-2.2962	down
Os.12261.1.S1_a_at	-1.3022157	down	-2.68139	down	1.088843	up	-3.49174	down
Os.35224.1.A1_x_at	-3.1493783	down	-1.22344	down	-2.01234	down	-3.85306	down
Os.8808.1.S1_at	2.5898688	up	-1.07751	down	2.652746	up	2.403569	up
Os.9326.1.S1_at	1.2734381	up	1.788163	up	1.375964	up	2.277114	up
Os.4251.1.S1_at	1.2562273	up	3.670268	up	-1.49212	down	4.61069	up
Os.21068.1.S1_at	-3.4830792	down	1.110078	up	-2.72926	down	-3.13769	down
Os.55382.1.S1_at	3.6364691	up	-1.2112	down	2.976005	up	3.002371	up
Os.10204.1.S1_at	-1.8734483	down	1.112776	up	-2.73382	down	-1.68358	down
Os.55640.1.S1_at	2.823662	up	-1.25192	down	3.075174	up	2.255459	up
Os.10054.1.S1_at	2.3990474	up	2.263162	up	1.08526	up	5.429433	up
Os.51584.1.S1_at	-2.361916	down	-1.3707	down	-1.79158	down	-3.23748	down
Os.12767.1.S1_a_at	-2.7742708	down	1.592633	up	-3.91067	down	-1.74194	down
Os.24035.1.S1_at	-1.9656976	down	-1.3797	down	-1.77969	down	-2.71206	down
OsAffx.32208.1.A1_a	1.1213154	up	-2.18979	down	-1.12087	down	-1.95288	down
Os.13543.1.S1_at	1.343909	up	2.810082	up	-1.14564	down	3.776495	up
Os.53663.1.S1_at	-4.8064866	down	1.923273	up	-4.71704	down	-2.49912	down
OsAffx.12064.1.S1_at	-3.0348322	down	-1.24289	down	-1.97298	down	-3.77197	down
Os.11831.1.S1_at	1.1884514	up	-3.70332	down	1.510208	up	-3.11609	down
Os.48058.1.S1_at	-2.333576	down	1.100735	up	-2.69917	down	-2.12002	down
Os.37745.1.S1_at	1.3503295	up	-1.72339	down	-1.42266	down	-1.27627	down
OsAffx.24262.1.S1_s_	1.4057894	up	1.423216	up	1.722658	up	2.000741	up
Os.27152.1.A1_at	-4.3550715	down	1.017279	up	-2.49351	down	-4.2811	down
Os.38127.1.S1_at	1.5196576	up	1.245404	up	1.967757	up	1.892587	up
Os.5622.1.S1_at	1.3797392	up	4.016582	up	-1.63919	down	5.541834	up
Os.6664.1.S1_at	1.2640824	up	1.115444	up	2.196376	up	1.410013	up
Os.23183.1.S1_at	2.2101681	up	2.38791	up	1.025254	up	5.277682	up
Os.11230.1.S1_s_at	-1.8272375	down	-1.05992	down	-2.30945	down	-1.93672	down
Os.52147.1.S1_at	2.4244506	up	1.337098	up	1.830481	up	3.241727	up
OsAffx.22650.2.S1_at	-1.6217963	down	-1.02557	down	-2.38516	down	-1.66326	down
Os.49453.1.S1_at	4.296668	up	2.30434	up	1.060514	up	9.900984	up
Os.16324.1.S1_at	-3.9428368	down	1.366574	up	-3.33928	down	-2.8852	down
Os.11335.3.S1_x_at	2.170313	up	1.6454	up	1.484269	up	3.571034	up
Os.11804.1.S1_at	1.5659864	up	3.390564	up	-1.3885	down	5.309577	up
Os.51809.1.S1_at	1.1384414	up	3.344278	up	-1.36986	down	3.807265	up
Os.54293.1.S1_at	-1.8897746	down	-1.4018	down	-1.74078	down	-2.64908	down
Os.2436.1.S1_at	1.1579547	up	3.512662	up	-1.44026	down	4.067504	up
Os.51287.1.S1_at	-1.59605	down	1.604451	up	1.520083	up	1.005264	up
OsAffx.31985.1.S1_at	3.257867	up	1.030596	up	2.366108	up	3.357546	up
Os.27829.1.S1_at	-3.383738	down	1.218721	up	-2.97137	down	-2.77647	down
Os.54930.1.S1_at	4.8908587	up	-1.04661	down	2.551648	up	4.673049	up
Os.54033.1.S1_at	2.2643578	up	1.194236	up	2.040831	up	2.704177	up
Os.14828.1.A1_s_at	-1.1510446	down	2.166829	up	1.124293	up	1.882489	up
Os.26059.1.S1_x_at	-1.5578146	down	-1.34822	down	-1.80585	down	-2.10027	down

Os.49126.1.S1_at	-1.3249825	down	-1.50485	down	-1.61743	down	-1.9939	down
Os.5606.1.S1_at	-4.331437	down	1.266563	up	-3.08172	down	-3.41984	down
OsAffx.3056.1.S1_at	-2.1828058	down	-1.09328	down	-2.22517	down	-2.38641	down
Os.13783.1.S1_at	2.040274	up	1.25199	up	1.942015	up	2.554402	up
Os.11222.1.S1_at	2.0050962	up	2.692482	up	-1.10817	down	5.398686	up
Os.17823.1.S1_a_at	-4.0505695	down	-1.35789	down	-1.78749	down	-5.50022	down
Os.27862.1.S1_at	2.6672418	up	-1.27324	down	3.090376	up	2.094845	up
Os.19523.1.S1_at	1.4123416	up	1.239016	up	1.958938	up	1.749913	up
Os.5885.1.S1_at	-4.2053547	down	-1.52926	down	-1.58662	down	-6.43107	down
Os.48829.1.A1_at	3.1445653	up	1.266539	up	1.915703	up	3.982714	up
OsAffx.2326.1.S1_x_a	-2.3846235	down	-1.01742	down	-2.38428	down	-2.42617	down
Os.25153.1.S1_at	1.1804007	up	1.060387	up	-2.57138	down	1.251681	up
Os.11183.1.S1_at	-1.1259907	down	4.078871	up	-1.68217	down	3.622473	up
Os.9684.1.S1_at	2.0629683	up	10.89204	up	-4.49223	down	22.46993	up
Os.1316.1.S2_at	4.92319	up	-6.41092	down	15.54309	up	-1.30219	down
Os.12286.1.S2_a_at	-2.0508137	down	-1.45922	down	-1.66149	down	-2.99258	down
Os.24475.1.S1_at	-2.3324273	down	1.109879	up	-2.69033	down	-2.10152	down
Os.13993.1.S1_at	2.9439106	up	1.976912	up	1.225639	up	5.819851	up
Os.10025.1.S1_a_at	2.5948665	up	-1.69968	down	4.116756	up	1.526682	up
Os.10772.1.S1_x_at	-1.2979885	down	-1.00172	down	-2.41785	down	-1.30023	down
Os.45928.1.S1_at	1.6408722	up	6.233746	up	-2.57611	down	10.22878	up
OsAffx.8236.1.S1_x_a	-1.250525	down	-1.80191	down	-1.34284	down	-2.25334	down
OsAffx.17883.1.S1_at	-1.609583	down	-2.22697	down	-1.08646	down	-3.58449	down
Os.2210.1.S1_at	-1.765403	down	4.045023	up	-1.67185	down	2.291275	up
Os.33966.1.S1_at	-2.190827	down	-1.3285	down	-1.82074	down	-2.91052	down
OsAffx.28932.1.S1_at	1.5244254	up	-1.35417	down	3.275353	up	1.12573	up
Os.51774.1.S1_at	-3.4921303	down	1.425964	up	-3.44846	down	-2.44896	down
Os.4683.2.S1_at	1.0957311	up	2.255582	up	1.072121	up	2.471511	up
Os.20722.2.S1_at	-2.366741	down	-1.27559	down	-1.89574	down	-3.01899	down
Os.12735.1.S1_s_at	-2.9089735	down	-1.00254	down	-2.41191	down	-2.91636	down
Os.10742.1.S1_at	-1.6955143	down	-1.21524	down	-1.98947	down	-2.06046	down
Os.22718.2.S1_at	1.4100194	up	1.660351	up	1.456115	up	2.341126	up
Os.10286.1.S1_x_at	-2.0379488	down	-1.04042	down	-2.32368	down	-2.12032	down
Os.53062.1.S2_at	-1.9863945	down	2.035239	up	-4.9203	down	1.02459	up
OsAffx.18219.1.S1_at	1.1468456	up	1.161788	up	2.080132	up	1.332391	up
Os.3388.2.S1_a_at	1.8416078	up	2.405558	up	1.004368	up	4.430095	up
Os.37603.1.S1_at	-1.6175317	down	-1.57301	down	-1.53569	down	-2.5444	down
Os.25087.1.S1_at	1.5019757	up	1.188164	up	2.032362	up	1.784594	up
Os.9556.1.S2_at	-5.181708	down	1.184412	up	-2.86008	down	-4.37492	down
Os.32498.1.S1_at	1.3364882	up	1.805845	up	1.337099	up	2.413491	up
OsAffx.29848.1.S1_x_	1.4485188	up	-1.00949	down	2.437069	up	1.434907	up
Os.9508.1.S1_at	2.8937612	up	-2.81425	down	6.79376	up	1.028252	up
OsAffx.30647.1.S1_x_	-1.2000242	down	-1.52462	down	-1.58282	down	-1.82958	down
Os.307.1.S2_at	1.1812956	up	1.34243	up	1.796442	up	1.585807	up
Os.8439.1.S1_a_at	-1.4550483	down	-2.0615	down	-1.16971	down	-2.99958	down
Os.17584.1.S1_at	-1.7159574	down	-1.2788	down	-1.88555	down	-2.19437	down
Os.48919.1.S1_at	-1.892033	down	-1.19099	down	-2.02457	down	-2.25339	down

Os.12690.1.S1_at	4.6740193	up	1.663032	up	1.449782	up	7.773045	up
Os.12119.1.S1_at	1.4061753	up	1.547219	up	1.557896	up	2.175661	up
Os.8018.3.S1_a_at	1.7880706	up	2.894842	up	-1.20116	down	5.176182	up
Os.170.1.S1_at	1.6517149	up	1.951982	up	1.233957	up	3.224118	up
OsAffx.5275.1.A1_at	1.191268	up	1.473049	up	1.633822	up	1.754796	up
Os.19622.1.S1_at	-1.7050709	down	-1.24674	down	-1.93023	down	-2.12578	down
Os.7853.3.S1_x_at	-2.8106859	down	-1.10878	down	-2.17004	down	-3.11643	down
Os.7085.1.S1_a_at	-1.2465136	down	-1.37874	down	-1.74512	down	-1.71861	down
OsAffx.32190.1.A1_a	1.0454633	up	-1.78364	down	-1.34887	down	-1.70608	down
AFFX-Os-r2-Bs-phe-M	-1.2579973	down	-1.33086	down	-1.80698	down	-1.67422	down
Os.4796.1.S2_at	-4.0671296	down	-1.03499	down	-2.32343	down	-4.20943	down
OsAffx.12207.1.S1_at	-2.1051297	down	-1.26108	down	-1.90685	down	-2.65474	down
Os.31185.1.S1_at	1.7477162	up	1.801314	up	1.333981	up	3.148185	up
Os.15260.1.S1_s_at	-2.2346592	down	-1.45208	down	-1.65441	down	-3.2449	down
OsAffx.32259.1.A1_a	1.1586353	up	-2.14377	down	-1.1206	down	-1.85025	down
Os.9298.1.S1_at	-1.9577212	down	-1.31409	down	-1.82811	down	-2.57262	down
Os.54934.1.S1_at	12.224327	up	-1.26025	down	3.022945	up	9.699946	up
AFFX-Os-r2-Bs-thr-5_	-2.5464475	down	1.505514	up	-3.61064	down	-1.69141	down
Os.18958.1.S1_at	-4.3477306	down	1.476138	up	-3.53996	down	-2.94534	down
Os.53506.1.S1_at	-3.8586948	down	1.488433	up	-3.56876	down	-2.59246	down
Os.51062.1.S1_at	-2.4002306	down	-1.05528	down	-2.27139	down	-2.53292	down
Os.15927.1.S1_s_at	1.788058	up	1.439185	up	1.665177	up	2.573347	up
Os.9473.2.S2_at	-2.543808	down	1.118348	up	-2.67988	down	-2.27461	down
Os.8774.1.S1_at	1.5588965	up	3.552183	up	-1.48333	down	5.537485	up
Os.47922.1.S2_x_at	2.522452	up	-1.39293	down	3.333285	up	1.8109	up
Os.54865.1.S1_at	1.6519644	up	1.683122	up	1.421387	up	2.780458	up
Os.21839.1.S1_at	1.8230349	up	1.764215	up	1.355558	up	3.216225	up
OsAffx.14221.1.S1_x_	-1.496092	down	-1.57656	down	-1.51671	down	-2.35867	down
Os.17112.1.S1_at	4.308966	up	1.879178	up	1.272113	up	8.097313	up
Os.322.1.S1_at	1.2298027	up	2.715713	up	-1.13624	down	3.339791	up
Os.27451.1.S1_at	1.0273466	up	1.65351	up	1.445467	up	1.698728	up
OsAffx.2612.1.S1_at	1.3795002	up	1.335848	up	1.788721	up	1.842802	up
Os.25221.1.S1_at	-1.93923	down	1.041455	up	-2.48793	down	-1.86204	down
Os.52570.1.S1_at	-1.3974857	down	-1.9444	down	-1.22845	down	-2.71727	down
Os.11569.1.S1_at	-1.4547471	down	1.983155	up	1.204387	up	1.36323	up
Os.54551.1.S1_at	1.4649771	up	2.27427	up	1.050056	up	3.331754	up
Os.49185.1.S1_at	1.0633023	up	1.016851	up	2.348399	up	1.08122	up
OsAffx.25289.1.S1_x_	1.6967225	up	1.2655	up	1.886841	up	2.147202	up
Os.46345.1.A1_s_at	1.8479018	up	3.825612	up	-1.60288	down	7.069355	up
Os.55491.1.S1_at	-2.6329355	down	2.204203	up	-5.26005	down	-1.19451	down
Os.8566.1.S1_at	1.1485708	up	1.668009	up	1.430639	up	1.915826	up
Os.22722.1.A1_s_at	-1.4887861	down	1.144143	up	-2.73016	down	-1.30122	down
Os.46325.1.A1_at	-2.120629	down	1.157221	up	-2.76081	down	-1.83252	down
Os.53210.1.S1_at	2.5355606	up	-5.45577	down	13.01528	up	-2.1517	down
OsAffx.24726.1.S1_s_	-1.731248	down	-1.18056	down	-2.0205	down	-2.04385	down
Os.52744.1.S1_at	-1.9340476	down	-1.28056	down	-1.8623	down	-2.47666	down
Os.50466.1.S1_at	1.4071335	up	1.652103	up	1.442945	up	2.32473	up

Os.8897.1.S1_at	2.6856484	up	-2.97039	down	7.073576	up	-1.10602	down
Os.27682.1.S1_at	2.6367188	up	1.279551	up	1.860187	up	3.373816	up
Os.6328.1.S1_at	2.876781	up	1.16987	up	2.034007	up	3.365459	up
Os.22819.1.S1_at	1.6553937	up	1.614926	up	1.473352	up	2.673339	up
Os.17446.1.S1_at	-1.3118815	down	-2.04757	down	-1.16144	down	-2.68617	down
Os.5361.1.S1_at	-4.384039	down	1.265192	up	-3.00854	down	-3.46512	down
Os.14882.1.S1_at	2.5069706	up	1.982455	up	1.199208	up	4.969957	up
Os.6139.1.S1_at	2.4783695	up	2.087395	up	1.138831	up	5.173335	up
Os.37969.2.S1_at	1.3059366	up	3.720073	up	-1.56566	down	4.85818	up
Os.26698.1.S1_a_at	-4.2404714	down	-1.52569	down	-1.55668	down	-6.46963	down
Os.23591.1.A1_at	2.2373245	up	1.171433	up	2.026763	up	2.620875	up
Os.12096.3.S1_x_at	-1.1672714	down	1.391674	up	-3.30274	down	1.192246	up
Os.12429.1.S1_at	1.7652326	up	-1.69655	down	4.025639	up	1.040487	up
Os.49116.1.S1_at	1.4825752	up	1.898332	up	1.249906	up	2.81442	up
Os.15093.1.S1_at	2.1729567	up	1.06874	up	2.220026	up	2.322325	up
Os.51595.1.S1_at	1.3826811	up	1.178338	up	2.013415	up	1.629266	up
Os.20335.1.S1_at	2.5559888	up	-1.24605	down	2.95597	up	2.051267	up
Os.11914.1.S1_at	1.4227992	up	4.10076	up	-1.72934	down	5.834557	up
Os.33112.1.S1_at	-1.3194305	down	-1.52941	down	-1.55035	down	-2.01796	down
Os.50354.1.S1_at	2.3468986	up	1.768153	up	1.34096	up	4.149674	up
Os.46160.2.S1_x_at	1.0694972	up	2.912623	up	-1.22877	down	3.115042	up
Os.4404.1.S1_at	-2.1463268	down	1.354183	up	-3.20935	down	-1.58496	down
Os.16915.1.S1_at	4.427404	up	1.090424	up	2.173401	up	4.827748	up
OsAffx.14641.1.S1_at	6.566334	up	-1.29134	down	3.060305	up	5.084884	up
Os.2196.1.S1_a_at	-3.4570243	down	-1.45963	down	-1.62345	down	-5.04599	down
Os.54039.1.S1_at	-4.7872486	down	-1.19385	down	-1.98484	down	-5.71524	down
Os.17895.2.S1_x_at	-1.8904611	down	1.084621	up	-2.57	down	-1.74297	down
Os.46759.1.S1_at	1.4033223	up	1.498656	up	1.580537	up	2.103097	up
Os.38447.1.S1_s_at	1.6105708	up	-2.11886	down	-1.11772	down	-1.3156	down
Os.18092.1.S1_at	-2.6361043	down	-1.39349	down	-1.69944	down	-3.67339	down
Os.52184.1.S1_at	-1.4574006	down	-1.58888	down	-1.49044	down	-2.31563	down
OsAffx.14869.1.S1_at	1.9119986	up	1.250665	up	1.892917	up	2.391269	up
Os.55230.1.S1_at	-1.6338825	down	-1.53182	down	-1.54532	down	-2.50281	down
Os.2677.1.S1_at	3.057463	up	1.239191	up	1.909821	up	3.788781	up
Os.28320.1.S1_at	2.955233	up	1.362213	up	1.737343	up	4.025656	up
Os.21068.2.S1_x_at	-2.4992034	down	1.096181	up	-2.5941	down	-2.27992	down
Os.16128.2.S1_at	2.9153268	up	1.065599	up	2.219659	up	3.10657	up
Os.47369.1.A1_at	1.2311451	up	3.40706	up	-1.44098	down	4.194586	up
OsAffx.10973.1.S1_x_	1.986427	up	-1.0003	down	2.364531	up	1.985822	up
Os.52621.1.S1_x_at	-3.353503	down	-1.62101	down	-1.45801	down	-5.43605	down
Os.34827.1.S1_at	-1.9912517	down	1.005548	up	-2.37525	down	-1.98027	down
Os.51359.1.S1_at	-1.4152286	down	-2.06954	down	-1.14065	down	-2.92887	down
Os.17937.1.S1_a_at	-3.201784	down	1.008727	up	-2.3809	down	-3.17408	down
OsAffx.10900.1.S1_at	1.1208179	up	-1.94195	down	-1.21523	down	-1.73261	down
Os.51453.1.S1_x_at	-1.4839948	down	-1.60733	down	-1.46779	down	-2.38527	down
Os.11620.1.S1_a_at	2.3659832	up	1.240878	up	1.90097	up	2.935895	up
Os.11085.1.S1_at	-1.7724472	down	-1.3648	down	-1.7277	down	-2.41903	down

Os.30754.1.S1_at	1.1431869	up	-1.22853	down	2.895742	up	-1.07465	down
Os.6019.1.S1_at	1.3809139	up	3.848912	up	-1.63372	down	5.315016	up
Os.34904.1.S1_at	1.6404402	up	1.352751	up	1.741471	up	2.219108	up
Os.50437.1.S1_at	2.768829	up	1.036489	up	2.272645	up	2.869862	up
Os.3415.1.S1_at	2.4345088	up	-1.76872	down	4.166007	up	1.376423	up
Os.51717.1.S1_at	2.5498762	up	1.03188	up	2.282464	up	2.631165	up
Os.12957.1.S1_at	1.0119934	up	1.371572	up	1.71673	up	1.388022	up
Os.24106.2.A1_at	1.1624339	up	1.197019	up	1.966901	up	1.391455	up
Os.51080.1.S1_at	3.3054962	up	1.77874	up	1.323556	up	5.879617	up
Os.50352.1.S1_at	-2.1260211	down	1.200765	up	-2.82683	down	-1.77056	down
Os.50349.2.S1_at	-1.545745	down	-1.87731	down	4.41893	up	-2.90184	down
Os.52062.1.S1_at	2.262073	up	2.099994	up	1.120623	up	4.75034	up
Os.9279.1.S1_at	1.0180701	up	-1.96697	down	-1.196	down	-1.93206	down
Os.18459.2.S1_x_at	1.4206712	up	1.003923	up	2.342468	up	1.426245	up
Os.17665.2.S1_at	1.442424	up	1.36999	up	1.716212	up	1.976106	up
Os.9829.1.S1_at	1.4108223	up	2.40927	up	-1.02481	down	3.399052	up
Os.52749.1.S1_at	-2.3133538	down	-1.04188	down	-2.25634	down	-2.41024	down
Os.10117.1.S1_at	1.621523	up	1.399241	up	1.679965	up	2.268901	up
Os.5532.1.S1_at	2.1484637	up	1.679136	up	1.399386	up	3.607562	up
Os.5874.1.S1_at	-2.4103405	down	1.185629	up	-2.78576	down	-2.03296	down
Os.55274.1.S1_at	3.8496096	up	-1.55156	down	3.644888	up	2.481119	up
Os.27922.1.S1_at	-1.5377952	down	-1.42592	down	-1.64695	down	-2.19278	down
Os.49803.1.S1_at	-3.3162131	down	1.048132	up	-2.46118	down	-3.16393	down
Os.14645.1.S1_s_at	1.7798059	up	1.415349	up	1.658127	up	2.519046	up
Os.28148.1.S1_at	1.6195894	up	1.417043	up	1.65506	up	2.295028	up
OsAffx.17906.1.S1_x_	2.4963148	up	1.601562	up	1.464196	up	3.998003	up
Os.11546.1.S1_at	-1.1321992	down	-1.65496	down	-1.41693	down	-1.87374	down
Os.19371.1.S1_at	1.0544473	up	1.231597	up	1.903172	up	1.298654	up
Os.28042.1.S1_a_at	3.129663	up	1.420816	up	1.649394	up	4.446675	up
Os.32877.1.S1_x_at	-3.3172722	down	1.240429	up	-2.90623	down	-2.67429	down
Os.8508.1.S1_at	4.387412	up	1.157166	up	2.023668	up	5.076964	up
Os.28420.1.S1_at	-1.6771456	down	-1.48628	down	-1.57495	down	-2.49271	down
OsAffx.15432.1.S1_at	-1.6012875	down	-1.37374	down	-1.70308	down	-2.19976	down
OsAffx.8000.1.S1_at	-1.4449002	down	-1.43982	down	-1.62466	down	-2.0804	down
Os.54317.1.S1_at	-1.1815779	down	2.501426	up	-1.06939	down	2.117022	up
Os.11866.1.S1_at	1.5229733	up	1.356505	up	1.723845	up	2.065921	up
Os.31415.1.S1_at	2.370436	up	1.006547	up	2.322892	up	2.385956	up
Os.7131.1.S1_at	1.6108662	up	1.247867	up	1.873452	up	2.010147	up
Os.38169.1.S1_at	1.9589523	up	2.121548	up	1.101723	up	4.156011	up
Os.39326.1.A1_at	1.0654233	up	-1.76457	down	-1.32441	down	-1.65622	down
OsAffx.15932.1.S1_at	1.7313933	up	1.55871	up	1.49904	up	2.698739	up
Os.11996.1.S1_at	1.3400321	up	1.060838	up	2.201989	up	1.421557	up
Os.10647.2.S1_at	1.2231828	up	1.126322	up	2.073595	up	1.377698	up
Os.5124.1.S1_s_at	2.2177844	up	1.134624	up	2.058408	up	2.516351	up
Os.11023.1.S2_a_at	3.1956034	up	2.921811	up	-1.25109	down	9.33695	up
Os.21957.2.S1_x_at	-2.5611513	down	1.986272	up	-4.63778	down	-1.28943	down
Os.51338.1.S1_at	2.3441458	up	1.241546	up	1.880376	up	2.910365	up

Os.50818.1.S1_at	2.6284807	up	-1.08185	down	2.524522	up	2.429607	up
Os.12167.1.S1_at	2.8462808	up	-1.23606	down	2.884354	up	2.302711	up
Os.46523.2.S1_at	1.2926903	up	-1.00331	down	2.340465	up	1.288426	up
Os.11766.1.S1_at	1.252287	up	2.407521	up	-1.03219	down	3.014907	up
Os.12399.1.S1_at	2.0355701	up	2.409037	up	-1.03298	down	4.903764	up
Os.7632.1.S1_at	1.8518751	up	2.391727	up	-1.02562	down	4.42918	up
Os.50822.1.S1_at	3.066892	up	1.896824	up	1.229285	up	5.817354	up
Os.5404.3.S1_a_at	-1.4471349	down	1.69075	up	-3.94064	down	1.168343	up
Os.11123.1.S1_at	2.1047378	up	1.686933	up	1.381474	up	3.550552	up
Os.43415.1.A1_at	-3.5440016	down	-1.14106	down	-2.04168	down	-4.04393	down
Os.16761.1.S1_at	2.010475	up	1.092773	up	2.131851	up	2.196993	up
Os.56276.1.S1_at	1.2195847	up	1.229642	up	1.894421	up	1.499653	up
Os.4959.1.S1_at	1.3111511	up	1.017241	up	2.288164	up	1.333757	up
Os.28385.3.S1_x_at	-1.4444183	down	-1.64016	down	-1.41889	down	-2.36908	down
Os.7622.1.S1_at	3.8700836	up	-1.04105	down	2.422573	up	3.71749	up
Os.7346.1.S1_at	-2.880617	down	1.561402	up	-3.63299	down	-1.84489	down
Os.46452.2.S1_x_at	-1.6483531	down	-1.08906	down	-2.13603	down	-1.79516	down
Os.5535.2.S1_x_at	1.692969	up	1.39652	up	1.665335	up	2.364265	up
Os.25606.1.S1_at	1.3419107	up	2.412521	up	-1.03739	down	3.237389	up
Os.536.1.S1_at	2.0162692	up	-1.55683	down	3.619734	up	1.295109	up
Os.6933.1.S1_at	-1.5086777	down	-1.64944	down	-1.40951	down	-2.48847	down
Os.7012.1.S1_at	1.3073772	up	2.532696	up	-1.08944	down	3.311189	up
Os.35681.1.S1_at	1.1761367	up	-2.72859	down	1.173744	up	-2.31996	down
Os.8557.1.S1_at	-1.3803424	down	-1.38837	down	-1.674	down	-1.91643	down
Os.27407.1.A1_at	1.4660748	up	2.96688	up	-1.27678	down	4.349668	up
Os.54420.1.S1_at	3.0168865	up	-1.06158	down	2.466134	up	2.841883	up
OsAffx.2326.1.S1_at	-2.2084763	down	-1.14922	down	-2.02027	down	-2.53804	down
Os.778.2.S1_x_at	-3.18102	down	1.410497	up	-3.2748	down	-2.25525	down
Os.51297.1.S1_at	1.5847663	up	1.147772	up	2.022222	up	1.81895	up
Os.51439.1.S1_at	-1.3175588	down	2.527939	up	-1.08946	down	1.918654	up
Os.6405.1.S2_at	2.0865319	up	1.180724	up	1.964789	up	2.463619	up
Os.24183.1.A1_at	-2.5678687	down	1.231917	up	-2.85707	down	-2.08445	down
Os.12995.1.S1_at	1.139117	up	-1.53661	down	3.56245	up	-1.34895	down
Os.27638.1.S1_at	-3.6080692	down	1.329677	up	-3.08236	down	-2.71349	down
OsAffx.27283.2.S1_at	-1.4017881	down	-1.01638	down	-2.28068	down	-1.42474	down
Os.55230.1.S1_x_at	-1.5073445	down	-1.51118	down	-1.53343	down	-2.27787	down
OsAffx.32324.1.A1_a	-1.0239952	down	-1.68311	down	-1.3766	down	-1.7235	down
Os.4857.1.S1_at	1.4468875	up	2.226382	up	1.040671	up	3.221324	up
Os.11194.1.S1_at	-2.6184525	down	-1.22121	down	-1.89706	down	-3.19767	down
Os.28544.1.S1_at	-1.1034877	down	1.60601	up	1.442024	up	1.455395	up
AFFX-PheX-M_at	-1.2830572	down	-1.13379	down	-2.04233	down	-1.45471	down
OsAffx.13012.1.S1_x_	-2.0782933	down	-1.32826	down	-1.74312	down	-2.76051	down
Os.8230.1.S1_at	1.8570787	up	1.038194	up	2.229825	up	1.928008	up
Os.52201.1.S1_at	-3.0160272	down	1.20073	up	-2.77926	down	-2.51183	down
Os.25236.1.S1_x_at	1.594342	up	1.668842	up	1.38678	up	2.660705	up
OsAffx.820.1.S1_x_at	-1.3739539	down	-1.5136	down	-1.52891	down	-2.07961	down
Os.12748.1.S1_at	1.2343855	up	1.424142	up	1.624535	up	1.75794	up

Os.5409.1.S1_a_at	-1.6633064	down	1.043895	up	-2.41504	down	-1.59337	down
Os.10820.1.S1_at	2.4653456	up	-1.35797	down	3.14037	up	1.815458	up
OsAffx.7060.1.S1_s_a	-2.6338108	down	1.212294	up	-2.80211	down	-2.17259	down
OsAffx.22906.1.S1_at	-1.0586144	down	-1.45514	down	-1.58838	down	-1.54043	down
Os.48485.1.S1_at	-1.3174115	down	-1.4819	down	-1.55968	down	-1.95228	down
Os.22666.1.S1_at	1.6574395	up	1.343347	up	1.720543	up	2.226517	up
OsAffx.1874.1.S1_at	-1.4991518	down	-1.24266	down	-1.85979	down	-1.86294	down
Os.5586.1.S1_at	-4.7784405	down	1.239699	up	-2.86488	down	-3.85452	down
Os.35005.1.S1_at	1.0912005	up	1.387428	up	1.66551	up	1.513962	up
OsAffx.25285.1.S1_at	-2.254162	down	1.061653	up	-2.45309	down	-2.12326	down
Os.14525.1.S1_at	1.991818	up	1.546542	up	1.493811	up	3.080431	up
Os.27065.1.S1_a_at	-1.0998838	down	-1.18689	down	-1.94628	down	-1.30544	down
Os.5425.1.S1_at	1.8167311	up	1.356336	up	1.702425	up	2.464098	up
Os.5101.1.S1_at	1.3489149	up	1.787996	up	1.291339	up	2.411854	up
Os.17885.1.S1_s_at	-1.7293664	down	-1.1487	down	-2.00992	down	-1.98653	down
Os.34471.1.S1_at	1.4419794	up	2.787719	up	-1.20749	down	4.019833	up
Os.5167.1.S1_at	-1.0969653	down	-1.53895	down	-1.50011	down	-1.68817	down
Os.5140.1.S1_at	-3.2776303	down	-1.16258	down	-1.98467	down	-3.81051	down
Os.24327.1.A1_at	1.128084	up	1.128557	up	2.043185	up	1.273107	up
Os.35540.1.S1_at	-4.173526	down	1.35695	up	-3.12837	down	-3.07567	down
Os.55247.1.S1_at	1.475359	up	2.406928	up	-1.04464	down	3.551083	up
Os.27207.1.S1_at	-2.4545033	down	-3.50623	down	1.522577	up	-8.60606	down
Os.31771.2.S1_x_at	2.1226497	up	-1.28127	down	2.950042	up	1.656671	up
Os.8253.1.S1_at	1.1130123	up	-1.245	down	2.865726	up	-1.11859	down
Os.13623.1.S1_at	1.4993455	up	2.587437	up	-1.12478	down	3.879461	up
Os.14199.1.S1_at	1.203931	up	2.537691	up	-1.1036	down	3.055205	up
Os.47874.1.S1_at	2.305473	up	1.673065	up	1.374281	up	3.857207	up
OsAffx.23761.2.S1_x_	-2.5721307	down	1.32173	up	-3.03676	down	-1.94603	down
Os.37822.3.A1_s_at	1.6753076	up	2.471888	up	-1.07592	down	4.141172	up
OsAffx.30069.1.S1_at	2.9535084	up	-1.31697	down	3.025286	up	2.242658	up
Os.15062.1.S1_a_at	-1.929283	down	1.544818	up	-3.54859	down	-1.24887	down
Os.2301.1.S1_at	1.3603927	up	1.551856	up	1.480193	up	2.111134	up
Os.27764.1.S1_at	2.582855	up	1.583758	up	1.450355	up	4.090617	up
Os.10785.3.S1_at	-2.232284	down	-1.71315	down	-1.34039	down	-3.82424	down
Os.17777.1.S1_x_at	2.3364155	up	1.151181	up	1.994488	up	2.689638	up
Os.31252.1.S1_at	2.2598076	up	-1.38271	down	3.174446	up	1.634337	up
Os.16786.1.S1_at	2.379558	up	-1.14275	down	2.622528	up	2.082301	up
OsAffx.32304.1.S1_at	-1.5335864	down	-1.06848	down	-2.14709	down	-1.6386	down
Os.11510.1.S1_at	2.3771367	up	-1.28642	down	2.95119	up	1.847872	up
Os.13866.2.S1_at	-2.7864125	down	-1.00325	down	-2.28573	down	-2.79548	down
Os.14762.3.S1_x_at	-2.4984229	down	-1.20362	down	-1.90519	down	-3.00716	down
Os.9677.2.S1_x_at	1.6534228	up	1.676147	up	1.368086	up	2.77138	up
Os.12415.1.S1_at	3.9723737	up	-7.27712	down	16.68346	up	-1.83193	down
Os.45906.1.S1_at	1.6847559	up	1.022108	up	2.242648	up	1.722002	up
Os.53512.1.S1_at	-2.4540946	down	-1.17344	down	-1.95259	down	-2.87973	down
Os.56224.1.S1_at	1.866118	up	4.008716	up	-1.74968	down	7.480737	up
Os.54299.1.S1_at	2.5075285	up	-1.50687	down	3.452325	up	1.664059	up

Os.46594.2.S1_x_at	-3.0479572	down	-1.03293	down	-2.21796	down	-3.14832	down
Os.8507.1.S1_at	3.9145334	up	-1.20585	down	2.761979	up	3.246284	up
Os.13624.1.S1_at	2.309961	up	-1.0385	down	2.378094	up	2.224322	up
Os.20657.1.S1_at	1.0104011	up	1.447898	up	1.580915	up	1.462958	up
Os.27581.1.A1_at	1.2495965	up	4.900516	up	-2.14162	down	6.123668	up
Os.52260.1.S1_at	1.2908956	up	2.199649	up	1.040192	up	2.839517	up
Os.10135.1.S1_at	-2.0701077	down	-1.06227	down	-2.15303	down	-2.19901	down
OsAffx.31642.1.S1_at	-1.4054477	down	-1.8944	down	-1.20705	down	-2.66248	down
OsAffx.31531.1.S1_s_	1.0696943	up	1.643891	up	1.390942	up	1.758461	up
Os.16039.1.S1_s_at	2.143719	up	1.561462	up	1.463402	up	3.347336	up
Os.52654.1.S1_at	1.1927342	up	1.176725	up	1.941282	up	1.40352	up
OsAffx.3275.1.S1_at	1.9989593	up	1.120458	up	2.038408	up	2.239751	up
Os.17301.1.S1_at	-2.086317	down	1.296047	up	-2.95995	down	-1.60975	down
Os.12075.1.S1_a_at	-1.398785	down	1.064867	up	-2.43129	down	-1.31358	down
Os.18468.1.S1_at	1.4858198	up	1.426858	up	1.600134	up	2.120053	up
Os.54463.1.S1_at	-1.7043867	down	-2.05328	down	-1.11194	down	-3.49959	down
Os.9976.1.S1_at	1.3173778	up	2.793781	up	-1.22406	down	3.680465	up
Os.15602.1.S1_x_at	-2.3645425	down	-1.3547	down	-1.6843	down	-3.20324	down
Os.10293.1.S1_at	-3.6260626	down	1.474069	up	-3.36259	down	-2.4599	down
Os.43236.1.S1_at	3.2645004	up	-1.47704	down	3.368998	up	2.210164	up
Os.22000.1.S1_at	-1.2354337	down	-1.49051	down	-1.53006	down	-1.84143	down
Os.5540.2.S1_at	-1.5085105	down	-1.22734	down	-1.85803	down	-1.85145	down
Os.8888.1.S1_at	-4.7376275	down	1.050885	up	-2.39642	down	-4.50822	down
Os.52239.1.S1_at	-1.8209245	down	-1.81374	down	-1.25725	down	-3.30269	down
Os.10937.1.S1_at	3.626279	up	-1.46907	down	3.349526	up	2.468421	up
Os.4186.1.S1_a_at	1.4410473	up	1.821075	up	1.251978	up	2.624255	up
Os.2373.1.S1_at	1.3108466	up	1.96246	up	1.16159	up	2.572483	up
Os.14215.1.S1_at	-2.2831562	down	-1.27195	down	-1.79117	down	-2.90406	down
Os.11800.1.S1_at	3.8276336	up	2.158494	up	1.055421	up	8.261924	up
Os.13479.1.S1_at	-1.1837666	down	-1.59015	down	-1.43214	down	-1.88236	down
Os.10495.1.S1_at	-2.1795125	down	1.085105	up	-2.47106	down	-2.00857	down
OsAffx.22999.1.S1_at	1.7027978	up	2.149322	up	1.059339	up	3.659861	up
Os.30608.2.S1_x_at	2.3629107	up	1.436847	up	1.584521	up	3.395142	up
Os.9043.1.S1_at	-1.0449705	down	-1.32855	down	-1.7136	down	-1.3883	down
OsAffx.30670.1.S1_at	-1.2099812	down	-2.35665	down	1.035189	up	-2.85151	down
Os.5242.1.S1_at	1.640958	up	1.189282	up	1.913864	up	1.951562	up
OsAffx.3968.1.S1_at	1.2252313	up	-1.08154	down	2.461672	up	1.132855	up
OsAffx.23090.2.S1_x_	-3.322703	down	1.530498	up	-3.48277	down	-2.17099	down
Os.34343.1.S2_s_at	-2.470955	down	-1.51089	down	-1.50579	down	-3.73333	down
Os.8948.1.S1_at	-4.8578286	down	1.425464	up	-3.24298	down	-3.40789	down
Os.27864.1.S1_at	-1.8981823	down	1.146004	up	-2.60553	down	-1.65635	down
Os.10031.1.S1_at	-3.2025383	down	-1.33482	down	-1.70252	down	-4.27481	down
Os.52438.1.S1_at	-1.3779056	down	1.126406	up	-2.55982	down	-1.22328	down
Os.11499.1.S1_at	1.9294535	up	1.728533	up	1.314278	up	3.335124	up
Os.17746.1.S1_s_at	1.2362336	up	4.611093	up	-2.02977	down	5.700388	up
Os.24364.1.A1_at	1.7630942	up	2.156412	up	1.052888	up	3.801957	up
Os.12751.1.S1_at	2.4841552	up	3.798591	up	-1.67318	down	9.43629	up

Os.12660.1.S2_at	-3.2776616	down	1.549599	up	-3.51685	down	-2.11517	down
Os.51434.1.S1_at	-1.9029688	down	1.061988	up	-2.40949	down	-1.79189	down
Os.47333.1.S1_at	-1.8949448	down	-1.17766	down	-1.92601	down	-2.23161	down
Os.12263.1.S1_at	-2.4942636	down	-2.45065	down	1.080562	up	-6.11256	down
Os.6266.1.S1_s_at	-1.5271733	down	-1.29417	down	-1.75214	down	-1.97642	down
Os.14118.1.S1_at	-2.9883046	down	-1.11657	down	-2.03076	down	-3.33665	down
OsAffx.29513.1.S1_at	-1.843637	down	-1.29528	down	-1.7495	down	-2.38803	down
Os.10798.1.S1_at	1.4071116	up	1.056992	up	2.14341	up	1.487305	up
Os.12410.1.S1_x_at	-1.276194	down	1.84653	up	-4.18221	down	1.446904	up
OsAffx.3461.1.S1_at	-2.109945	down	-1.65857	down	-1.36553	down	-3.49948	down
Os.49278.1.S1_at	-3.33369	down	1.14954	up	-2.60327	down	-2.90002	down
Os.25027.1.S1_a_at	1.8010548	up	-1.32036	down	2.989349	up	1.364062	up
Os.47019.1.S1_at	1.741591	up	-1.40128	down	3.172172	up	1.242861	up
Os.33892.2.S1_x_at	-2.4597669	down	-1.27966	down	-1.76887	down	-3.14767	down
Os.25200.1.S1_at	-2.0209084	down	-1.27431	down	-1.77528	down	-2.57527	down
Os.52431.1.S1_at	-1.7812722	down	-1.92426	down	-1.17561	down	-3.42763	down
Os.8309.1.S1_at	2.2693894	up	1.063614	up	2.126532	up	2.413755	up
Os.52493.1.A1_s_at	-1.2561265	down	1.675995	up	-3.78993	down	1.334257	up
Os.170.2.S1_at	1.7314644	up	1.484768	up	1.522865	up	2.570822	up
Os.7893.1.S1_at	-1.96585	down	-2.20197	down	-1.02675	down	-4.32874	down
Os.20625.2.S1_at	1.4096745	up	1.014847	up	2.227371	up	1.430604	up
Os.52216.1.S1_at	3.4564123	up	1.27309	up	1.775338	up	4.400325	up
Os.26781.1.S1_at	-2.3180928	down	-1.4486	down	-1.55946	down	-3.35798	down
Os.9199.1.S1_at	1.2316775	up	2.222691	up	1.01632	up	2.737638	up
OsAffx.19579.1.S1_at	1.5120296	up	2.234711	up	1.01056	up	3.37895	up
Os.47558.1.S1_at	-2.1491084	down	1.02608	up	-2.3167	down	-2.09448	down
Os.1503.1.S1_at	2.2329528	up	1.77998	up	1.268427	up	3.974611	up
Os.7439.1.S1_at	1.2175249	up	1.329635	up	1.697837	up	1.618864	up
Os.38312.2.S1_x_at	-2.1916928	down	-1.04799	down	-2.15336	down	-2.29687	down
Os.3049.1.S1_at	1.9910213	up	1.696186	up	1.330278	up	3.377143	up
Os.49702.1.S1_at	2.20676	up	2.298692	up	-1.01896	down	5.07266	up
Os.5810.1.S1_at	-1.4415166	down	-1.35058	down	-1.66987	down	-1.94688	down
Os.12410.3.S1_s_at	-1.4227695	down	1.963042	up	-4.42599	down	1.379733	up
Os.10591.1.S1_at	3.5341046	up	1.048364	up	2.150355	up	3.70503	up
OsAffx.19745.1.S1_at	-1.760713	down	-1.72159	down	-1.30931	down	-3.03122	down
Os.10932.1.A1_at	-3.307034	down	1.149586	up	-2.59023	down	-2.87672	down
Os.19940.1.S1_s_at	1.2633994	up	1.469364	up	1.532808	up	1.856394	up
Os.12710.1.S1_at	1.5665516	up	2.540952	up	-1.12835	down	3.980533	up
Os.23706.1.A1_at	1.3659898	up	-2.47352	down	1.098489	up	-1.81079	down
AFFX-r2-Bs-thr-M_s_a	-1.448302	down	-1.06612	down	-2.11027	down	-1.54406	down
Os.49352.1.S1_at	2.8063986	up	2.798616	up	-1.24405	down	7.854032	up
Os.11798.1.S1_at	2.0855896	up	-1.48364	down	3.337009	up	1.40572	up
Os.18612.1.S1_x_at	-2.1490097	down	1.188429	up	-2.673	down	-1.80828	down
Os.3426.1.S1_at	1.0019636	up	-1.47785	down	3.323009	up	-1.47496	down
Os.18178.1.S1_at	5.1694884	up	1.080513	up	2.080503	up	5.585701	up
OsAffx.31611.1.S1_s_	2.0341096	up	1.810621	up	1.241446	up	3.683002	up
Os.5917.1.S1_a_at	-1.6258891	down	-1.21824	down	-1.84427	down	-1.98073	down

Os.12839.1.S1_at	-1.5341847	down	-1.21792	down	-1.84341	down	-1.86852	down
Os.12183.1.S1_at	-1.459515	down	-1.0443	down	-2.1489	down	-1.52417	down
Os.6058.1.S1_at	1.5498464	up	1.750342	up	1.281735	up	2.712762	up
Os.47136.1.A1_x_at	1.6470582	up	1.390577	up	1.61334	up	2.290361	up
OsAffx.27469.1.S1_at	2.0951536	up	1.133398	up	1.979371	up	2.374644	up
Os.6244.1.S1_at	2.9700286	up	1.436853	up	1.561233	up	4.267495	up
Os.12628.1.S1_at	1.8437512	up	-1.0158	down	2.278006	up	1.815072	up
Os.47600.1.S1_s_at	1.1662244	up	1.234907	up	1.815403	up	1.440178	up
Os.30608.3.S1_at	1.4898875	up	1.754023	up	1.278072	up	2.613298	up
OsAffx.6337.1.S1_x_a	1.1831213	up	-1.35461	down	-1.6521	down	-1.14495	down
Os.7423.1.S1_at	-1.0800949	down	1.230544	up	1.817848	up	1.139292	up
Os.39995.1.S1_x_at	1.3253086	up	-2.88101	down	1.287957	up	-2.17384	down
Os.11916.1.S1_at	-2.3741815	down	1.275036	up	-2.85173	down	-1.86205	down
Os.37693.1.A1_at	2.0089803	up	1.353288	up	1.652412	up	2.718728	up
Os.10689.1.S1_at	-2.2677047	down	1.279133	up	-2.86013	down	-1.77284	down
OsAffx.7413.1.S1_at	-2.4950333	down	1.036683	up	-2.31801	down	-2.40675	down
Os.39985.1.A1_at	-1.4968402	down	-1.09448	down	-2.04254	down	-1.63826	down
Os.10501.1.S1_at	-3.5314643	down	1.164391	up	-2.60233	down	-3.03289	down
Os.53242.1.S1_at	2.8171175	up	1.206812	up	1.851882	up	3.399732	up
Os.18428.1.S1_at	2.5770342	up	1.077797	up	2.073495	up	2.77752	up
Os.11986.3.S1_at	-2.091324	down	5.406006	up	-2.41921	down	2.584968	up
Os.37811.1.S1_at	4.9107246	up	-1.73893	down	3.885478	up	2.823998	up
OsAffx.25417.1.S1_s_	2.7013736	up	-1.61343	down	3.604323	up	1.674306	up
Os.48165.1.A1_at	-2.5924437	down	1.108387	up	-2.47501	down	-2.33893	down
Os.53358.1.S1_at	2.223432	up	-1.0279	down	2.295033	up	2.163076	up
Os.11331.1.S1_at	3.4038002	up	-1.39075	down	3.104824	up	2.447465	up
Os.21244.1.S1_at	-3.673917	down	1.443054	up	-3.22141	down	-2.54593	down
Os.8408.1.S1_at	2.1862044	up	1.607654	up	1.388374	up	3.514661	up
Os.37287.1.S1_at	1.0326015	up	1.257653	up	1.774398	up	1.298654	up
Os.15537.1.S1_at	1.5663849	up	-1.04546	down	2.332012	up	1.498274	up
Os.9898.1.S1_at	-1.4636586	down	-1.5701	down	-1.41993	down	-2.2981	down
Os.48429.1.S1_at	-1.464637	down	-1.37791	down	-1.61782	down	-2.01814	down
Os.49336.1.S1_s_at	-1.3706462	down	-1.46126	down	-1.5253	down	-2.00287	down
Os.15216.1.S1_at	2.4976377	up	1.234043	up	1.806113	up	3.082192	up
Os.26411.1.A1_at	6.959557	up	-1.15355	down	2.570333	up	6.033165	up
Os.24927.1.S1_at	-4.5116982	down	1.565794	up	-3.4874	down	-2.88141	down
Os.13608.1.S1_at	-5.659944	down	1.483063	up	-3.30267	down	-3.81639	down
Os.8813.1.S1_s_at	-1.5328286	down	-1.39214	down	-1.59951	down	-2.13392	down
Os.52439.1.S1_at	-1.2193915	down	-1.80257	down	-1.23515	down	-2.19804	down
Os.10078.1.S1_at	-2.4402182	down	-1.32279	down	-1.68199	down	-3.2279	down
Os.32071.2.S1_x_at	3.002687	up	-1.69776	down	3.77487	up	1.768619	up
Os.15570.1.S1_at	1.061218	up	1.855057	up	1.198344	up	1.96862	up
Os.55664.1.S1_at	1.0838673	up	1.163067	up	1.911265	up	1.26061	up
Os.50179.1.S1_at	1.2398413	up	-1.67483	down	3.72187	up	-1.35084	down
Os.10633.1.S1_at	2.1452513	up	-1.21622	down	2.702246	up	1.763872	up
OsAffx.25045.1.S1_at	-1.5787691	down	-1.33549	down	-1.66355	down	-2.10843	down
OsAffx.12255.1.S1_at	2.155646	up	1.640157	up	1.354528	up	3.535597	up

Os.41723.1.S1_at	-4.9814954	down	2.208973	up	-4.90501	down	-2.25512	down
Os.36875.1.A1_at	-1.4806184	down	1.532314	up	1.449017	up	1.034915	up
Os.24656.1.S1_at	-1.3840269	down	-1.55101	down	-1.43145	down	-2.14664	down
Os.36903.1.S1_at	-3.1574078	down	1.398714	up	-3.10533	down	-2.25736	down
Os.14493.1.S1_at	-2.7623286	down	1.160937	up	-2.57693	down	-2.3794	down
Os.53274.1.S1_at	1.3188018	up	1.243506	up	1.78482	up	1.639939	up
Os.5181.1.S1_a_at	-1.3333457	down	-1.31516	down	-1.68654	down	-1.75356	down
Os.11715.2.S1_x_at	-1.7232418	down	-1.60934	down	-1.37757	down	-2.77328	down
Os.11425.1.S1_at	-1.095876	down	1.562874	up	1.417796	up	1.426142	up
Os.20077.3.S1_x_at	-1.4870554	down	-1.36471	down	-1.62344	down	-2.02939	down
OsAffx.3581.1.S1_at	-2.087328	down	1.152876	up	-2.55383	down	-1.81054	down
OsAffx.13521.1.S1_at	-1.7142537	down	1.14914	up	-2.54544	down	-1.49177	down
Os.3420.1.S1_at	1.5552886	up	1.244327	up	1.780003	up	1.935288	up
OsAffx.13521.1.S1_x_	-1.7748574	down	1.110565	up	-2.45957	down	-1.59816	down
Os.52894.1.S1_at	-1.1337256	down	1.443735	up	1.532512	up	1.273443	up
OsAffx.6446.1.S1_at	1.5393922	up	-2.02362	down	-1.09335	down	-1.31456	down
Os.12761.1.S1_at	-1.1055893	down	-3.659	down	1.654183	up	-4.04535	down
Os.14762.1.S1_x_at	-2.9771323	down	-1.09458	down	-2.02063	down	-3.25872	down
OsAffx.12964.1.S1_at	1.500599	up	-1.32114	down	2.921965	up	1.135834	up
Os.2239.1.S1_at	2.5418856	up	-4.72108	down	10.44108	up	-1.85732	down
OsAffx.32334.1.A1_a	-1.0770056	down	-1.89502	down	-1.16699	down	-2.04095	down
Os.10600.1.S1_a_at	-1.9438878	down	1.189266	up	-2.62968	down	-1.63453	down
OsAffx.31655.2.S1_s_	4.1597376	up	1.056631	up	2.092609	up	4.395309	up
Os.4651.1.S1_at	2.9874172	up	1.91517	up	1.154351	up	5.721412	up
Os.6671.1.S1_a_at	3.4946728	up	1.124824	up	1.965165	up	3.930893	up
Os.8018.2.S1_a_at	1.9962219	up	2.8653	up	-1.29657	down	5.719775	up
Os.23030.1.S1_at	-1.8918626	down	-1.54087	down	-1.43413	down	-2.91511	down
Os.14086.1.S1_s_at	-2.4236794	down	1.267337	up	-2.80048	down	-1.91242	down
Os.10034.1.S1_at	2.4365673	up	1.271709	up	1.737533	up	3.098604	up
Os.52742.1.S1_at	1.8280678	up	3.715361	up	-1.68151	down	6.791931	up
Os.4806.1.S1_at	-2.3298972	down	1.107128	up	-2.4461	down	-2.10445	down
OsAffx.15200.4.A1_a	-1.6449876	down	-1.91863	down	-1.15145	down	-3.15613	down
Os.57449.1.A1_at	2.4935207	up	1.513044	up	1.460037	up	3.772807	up
Os.50286.1.S1_at	-3.8608356	down	-1.09288	down	-2.02128	down	-4.21944	down
Os.12678.1.S1_at	1.8553085	up	1.441473	up	1.531984	up	2.674377	up
Os.49342.1.S1_at	-3.062851	down	1.226237	up	-2.707	down	-2.49777	down
Os.14537.1.S1_at	1.6611824	up	1.632821	up	1.351814	up	2.712414	up
Os.46584.1.S1_at	1.0839058	up	1.930831	up	1.143039	up	2.092839	up
Os.37965.1.S1_at	1.0669258	up	1.360634	up	1.621455	up	1.451695	up
Os.38295.1.S1_at	-2.0331228	down	1.374719	up	-3.03162	down	-1.47894	down
Os.27545.1.S1_at	-2.4395852	down	-1.44196	down	-1.529	down	-3.51778	down
OsAffx.27946.1.S1_at	-1.9882948	down	-1.23528	down	-1.78461	down	-2.45609	down
Os.30306.1.A1_at	-1.4553813	down	-1.70856	down	3.765014	up	-2.48661	down
Os.12096.3.S1_a_at	-1.2420373	down	1.686746	up	-3.71561	down	1.358048	up
Os.20595.1.S1_a_at	-2.4501493	down	-1.34876	down	-1.63302	down	-3.30466	down
Os.17759.1.A1_s_at	-1.1879354	down	1.01264	up	-2.22984	down	-1.17311	down
Os.23924.1.S1_at	1.5329441	up	-1.02371	down	2.253874	up	1.497434	up

Os.36280.1.S1_at	2.437766	up	-1.70332	down	3.749086	up	1.431185	up
Os.10956.1.S1_at	1.517747	up	1.202325	up	1.830384	up	1.824825	up
Os.47651.1.S1_s_at	-2.152739	down	1.008168	up	-2.21862	down	-2.1353	down
Os.25736.1.S1_at	3.3777292	up	-1.39258	down	3.06334	up	2.425519	up
Os.37477.1.S1_at	-1.7373059	down	-1.62636	down	-1.3521	down	-2.82548	down
Os.51105.1.S1_s_at	-1.8358526	down	1.304853	up	-2.86922	down	-1.40694	down
Os.4929.1.S1_at	1.6343741	up	1.871733	up	1.174735	up	3.059112	up
Os.50548.2.S1_x_at	-1.2870761	down	-1.13317	down	-1.93982	down	-1.45848	down
Os.53749.1.S1_at	-2.182016	down	-1.27836	down	-1.71948	down	-2.78941	down
OsAffx.4763.1.S1_at	1.3817713	up	2.007805	up	1.094531	up	2.774327	up
Os.27945.1.A1_x_at	-1.4356378	down	-1.35279	down	-1.62407	down	-1.94212	down
Os.15397.1.S1_at	1.1626129	up	1.310847	up	1.675377	up	1.524008	up
Os.15707.1.S1_at	-2.3146002	down	-1.01491	down	-2.16325	down	-2.34911	down
Os.6723.1.S1_s_at	3.0680566	up	1.748222	up	1.255783	up	5.363642	up
Os.34161.1.S1_a_at	1.1536504	up	1.120368	up	1.958279	up	1.292513	up
Os.52492.1.S1_at	-2.7061837	down	-1.4784	down	-1.48373	down	-4.00083	down
OsAffx.5481.1.S1_at	2.3640585	up	1.142281	up	1.919941	up	2.700419	up
OsAffx.27039.1.S1_at	-1.1896596	down	-1.27166	down	-1.72451	down	-1.51284	down
OsAffx.5721.1.S1_s_a	-1.5480992	down	-1.72309	down	-1.27262	down	-2.66751	down
Os.19114.1.S2_at	-1.6085061	down	1.151636	up	-2.5253	down	-1.39671	down
Os.11911.1.S1_at	-2.3216429	down	1.040929	up	-2.28235	down	-2.23036	down
OsAffx.5617.1.S1_x_a	-1.7065667	down	1.021233	up	-2.23901	down	-1.67108	down
Os.28200.1.S1_x_at	-1.1986557	down	-8.00312	down	3.651195	up	-9.59299	down
OsAffx.3776.1.S1_at	-2.8156474	down	-1.5792	down	-1.38792	down	-4.44646	down
Os.12688.1.S1_at	-3.490331	down	-1.01866	down	-2.15135	down	-3.55545	down
Os.15197.1.S1_at	-2.040218	down	2.300846	up	-5.04197	down	1.127745	up
Os.2237.1.S1_at	-2.6087062	down	1.09262	up	-2.39399	down	-2.38757	down
Os.18446.1.S1_at	-3.1541946	down	1.452529	up	-3.18201	down	-2.17152	down
Os.16784.1.S1_at	-2.615385	down	-1.06403	down	-2.05802	down	-2.78285	down
Os.11186.1.S1_at	-2.0666552	down	-1.21172	down	-1.80693	down	-2.50422	down
Os.9502.1.S1_at	1.2553239	up	1.485579	up	1.473762	up	1.864883	up
Os.37754.1.S1_at	-1.4685533	down	-1.45717	down	-1.50242	down	-2.13993	down
Os.8375.1.S1_at	1.6244534	up	2.433667	up	-1.1117	down	3.953379	up
Os.9219.1.S1_at	2.2988749	up	-1.50834	down	3.301438	up	1.52411	up
Os.11549.1.S1_at	3.2980928	up	-1.2357	down	2.704048	up	2.669007	up
OsAffx.29926.1.S1_s_	-2.2037902	down	-1.54523	down	-1.41508	down	-3.40536	down
OsAffx.11050.2.S1_x_	1.5472971	up	1.965945	up	1.11216	up	3.0419	up
Os.10155.1.S1_at	1.7892005	up	-1.27939	down	2.796869	up	1.398477	up
Os.316.1.S1_at	-3.3010619	down	1.574571	up	-3.44129	down	-2.09648	down
Os.1443.1.S1_a_at	-1.0806448	down	-3.09236	down	1.415015	up	-3.34174	down
Os.2488.1.S1_at	2.1169019	up	1.152921	up	1.894414	up	2.440622	up
OsAffx.29871.1.S1_x_	-2.087799	down	1.771343	up	-3.86867	down	-1.17865	down
Os.19742.1.S1_at	2.2602718	up	2.113197	up	1.033277	up	4.7764	up
Os.32267.1.S1_at	1.5890398	up	2.052436	up	1.063753	up	3.261402	up
Os.11064.1.S1_a_at	-2.524495	down	-1.19265	down	-1.83028	down	-3.01083	down
Os.6681.1.S1_at	1.6835238	up	1.709661	up	1.276741	up	2.878255	up
OsAffx.4296.1.S1_at	3.2180684	up	1.557646	up	1.401152	up	5.012612	up

Os.12663.1.S1_x_at	-2.0473826	down	1.281686	up	-2.79712	down	-1.59741	down
Os.16598.1.S1_at	-2.257338	down	3.305578	up	-1.51468	down	1.46437	up
Os.31128.2.S1_at	-1.8724508	down	-1.56898	down	-1.39071	down	-2.93784	down
Os.17215.1.S2_at	-2.5271766	down	1.136014	up	-2.47863	down	-2.2246	down
Os.41226.1.S1_at	-4.936272	down	1.602913	up	-3.49649	down	-3.07956	down
Os.27239.1.S1_at	1.8948749	up	1.337924	up	1.63009	up	2.535199	up
Os.37577.1.S1_at	-1.9519379	down	-1.22696	down	-1.77726	down	-2.39496	down
Os.55387.1.S1_at	1.6939127	up	1.721951	up	1.266262	up	2.916835	up
Os.52475.1.S1_at	-2.7668567	down	-1.3089	down	-1.66532	down	-3.62153	down
OsAffx.28437.3.S1_at	1.0464035	up	1.654415	up	1.317007	up	1.731185	up
OsAffx.23071.1.S1_at	1.1273873	up	1.680868	up	1.29585	up	1.894989	up
OsAffx.8351.1.S1_at	1.1303531	up	-2.57713	down	1.183508	up	-2.27994	down
Os.11800.1.S1_s_at	3.9300122	up	1.98366	up	1.097606	up	7.795807	up
OsAffx.21421.1.S1_x_	1.256429	up	-1.68052	down	-1.29516	down	-1.33754	down
Os.46638.1.S1_at	-2.4631271	down	1.687053	up	-3.67107	down	-1.46002	down
Os.10754.1.S1_at	2.6899474	up	-1.29973	down	2.826965	up	2.069623	up
Os.51746.1.S1_s_at	-1.0492554	down	1.67645	up	1.297196	up	1.597752	up
Os.25548.1.A1_at	-2.5526588	down	1.269517	up	-2.75991	down	-2.01073	down
Os.39420.1.S1_at	1.7028239	up	1.659713	up	1.309699	up	2.826199	up
Os.35653.1.S1_a_at	2.1960483	up	-1.0216	down	2.21985	up	2.149609	up
Os.10510.1.S1_at	3.9386332	up	1.769004	up	1.228101	up	6.967456	up
Os.54462.1.S1_at	-2.3133466	down	-1.20036	down	-1.8098	down	-2.77685	down
Os.49692.1.S1_at	1.5230589	up	1.606274	up	1.352371	up	2.446449	up
OsAffx.25178.1.S1_at	1.5423884	up	-2.08595	down	4.529424	up	-1.35242	down
Os.483.1.S1_at	3.2834125	up	-1.8379	down	3.990558	up	1.786502	up
Os.47378.1.S1_s_at	-2.3569129	down	-1.47441	down	-1.47256	down	-3.47506	down
Os.8970.1.S1_at	-2.1427283	down	1.068949	up	-2.32038	down	-2.00452	down
Os.22957.1.S1_at	-1.8837922	down	1.45581	up	-3.16003	down	-1.29398	down
Os.38031.2.A1_at	2.1947362	up	-1.85961	down	4.036498	up	1.180211	up
Os.23262.1.A1_s_at	-1.0144604	down	-1.54245	down	3.347987	up	-1.56476	down
Os.15219.1.S1_at	1.2229686	up	-1.308	down	2.838551	up	-1.06953	down
Os.14544.1.S1_at	-2.2744162	down	-1.21807	down	-1.78099	down	-2.7704	down
Os.49309.1.S1_at	-2.3616529	down	-1.17498	down	-1.84626	down	-2.7749	down
Os.158.1.S1_at	-1.2610734	down	-1.38079	down	-1.56999	down	-1.74128	down
Os.40342.1.A1_s_at	1.0670431	up	2.787984	up	-1.28642	down	2.974899	up
Os.3739.1.S1_at	-2.4428973	down	1.715316	up	-3.71669	down	-1.42417	down
OsAffx.23290.2.S1_at	-1.3342322	down	-1.68792	down	-1.28342	down	-2.25207	down
Os.54942.1.S1_at	4.2137713	up	-1.76518	down	3.823838	up	2.387159	up
Os.47890.1.S1_s_at	-2.2145762	down	-1.64921	down	-1.3129	down	-3.6523	down
Os.27292.1.A1_at	2.2841887	up	3.255612	up	-1.50424	down	7.436432	up
OsAffx.22176.2.S1_at	-1.5387539	down	-1.65138	down	-1.31044	down	-2.54107	down
Os.15087.1.S1_at	-3.98441	down	1.184724	up	-2.56349	down	-3.36315	down
Os.4902.1.S1_at	-2.7760477	down	1.028163	up	-2.22462	down	-2.70001	down
Os.49096.1.S1_at	-2.2836046	down	1.391555	up	-3.01014	down	-1.64105	down
Os.21870.1.S1_at	-1.098887	down	3.383156	up	-1.56439	down	3.078712	up
OsAffx.3100.1.S1_at	1.2551545	up	1.04675	up	2.064622	up	1.313833	up
Os.49330.1.S1_at	-2.4580364	down	-1.14213	down	-1.89173	down	-2.8074	down

Os.47363.1.A1_at	1.8207829	up	2.083502	up	1.036893	up	3.793605	up
Os.54593.1.S1_at	-1.9477868	down	1.659756	up	-3.58405	down	-1.17354	down
Os.15402.1.S1_at	-3.0553966	down	1.233269	up	-2.66136	down	-2.47748	down
Os.3066.1.S1_at	1.6980171	up	-1.07765	down	2.325309	up	1.575665	up
OsAffx.11715.1.S1_at	-1.796718	down	-1.62869	down	-1.32464	down	-2.9263	down
Os.53228.1.S1_at	-1.7301867	down	-1.70478	down	-1.26495	down	-2.94958	down
Os.53996.1.S1_at	1.6916586	up	1.132806	up	1.90332	up	1.916322	up
OsAffx.13790.1.S1_at	1.2083973	up	1.468373	up	1.468213	up	1.774378	up
Os.7028.1.S1_at	-3.144526	down	2.542855	up	-5.48141	down	-1.23661	down
Os.253.1.S1_at	1.4848416	up	1.366161	up	1.577467	up	2.028533	up
OsAffx.18566.1.S1_x_	-1.2487267	down	-1.06712	down	-2.01952	down	-1.33255	down
AFFX-r2-Bs-thr-5_s_a	-2.3315725	down	1.683757	up	-3.6286	down	-1.38474	down
Os.14313.1.S1_s_at	1.4359097	up	2.904314	up	-1.34778	down	4.170332	up
Os.4616.1.S1_at	1.3362689	up	1.560289	up	1.381061	up	2.084965	up
Os.17730.1.S1_at	1.7581912	up	1.708289	up	1.261332	up	3.003499	up
Os.48388.1.S1_at	1.0579236	up	1.601828	up	1.345071	up	1.694612	up
Os.53320.2.S1_x_at	-2.4389875	down	-2.07488	down	-1.03833	down	-5.06062	down
Os.48286.1.A1_at	1.5400053	up	1.080407	up	1.993989	up	1.663833	up
Os.23801.1.A1_at	-1.9028629	down	-1.29526	down	-1.66243	down	-2.46471	down
Os.17577.4.A1_x_at	-2.511534	down	-1.07497	down	-2.003	down	-2.69982	down
Os.51737.1.S1_x_at	2.460668	up	-1.43187	down	3.082673	up	1.718494	up
Os.49170.1.S1_at	-1.3099079	down	-1.33417	down	-1.61332	down	-1.74763	down
Os.17939.3.S1_x_at	-1.8894329	down	-1.49479	down	-1.43981	down	-2.82431	down
OsAffx.13553.1.S1_at	1.1115174	up	-1.14688	down	-1.87626	down	-1.03182	down
OsAffx.15154.1.S1_at	2.634458	up	-1.33291	down	2.868141	up	1.976467	up
Os.49460.1.S1_at	1.8590524	up	2.339462	up	-1.08737	down	4.349182	up
Os.50445.1.S1_at	1.1216664	up	1.465277	up	1.468304	up	1.643552	up
Os.30608.3.S1_x_at	2.401262	up	1.965975	up	1.094286	up	4.72082	up
OsAffx.12547.1.S1_at	-1.0740032	down	4.715024	up	-2.19173	down	4.39014	up
Os.2328.1.S1_at	-2.4153683	down	-1.1296	down	-1.90419	down	-2.72841	down
Os.53666.1.S1_s_at	-2.5158496	down	1.010778	up	-2.1739	down	-2.48902	down
Os.19130.1.S1_at	-1.9160237	down	-2.33595	down	1.086212	up	-4.47573	down
OsAffx.19075.1.S1_at	-1.4175006	down	-1.31278	down	-1.63812	down	-1.86086	down
OsAffx.28387.1.S1_s_	-1.5874249	down	-1.13399	down	-1.89611	down	-1.80013	down
Os.11749.1.S1_at	1.7553349	up	1.490549	up	1.442477	up	2.616412	up
Os.38099.1.S1_at	1.1770089	up	-5.18844	down	2.413447	up	-4.40816	down
Os.27393.1.S1_s_at	-2.4655776	down	1.05663	up	-2.27089	down	-2.33344	down
Os.17118.1.A1_at	-1.5488616	down	-1.09157	down	-1.96846	down	-1.6907	down
Os.21635.1.S1_at	-1.2199161	down	1.087786	up	1.975147	up	-1.12147	down
Os.2225.1.S1_at	1.592641	up	1.449884	up	1.481642	up	2.309145	up
Os.8637.1.S1_at	2.148505	up	-1.27459	down	2.7378	up	1.685644	up
Os.20077.1.S1_at	-4.42168	down	1.276806	up	-2.7425	down	-3.46308	down
Os.10474.1.S1_s_at	-2.21759	down	-1.39095	down	-1.54413	down	-3.08455	down
Os.28026.1.S1_at	-1.6907687	down	1.724153	up	-3.70264	down	1.019745	up
Os.2362.1.S1_a_at	-1.476618	down	-1.11036	down	2.384479	up	-1.63958	down
Os.8069.1.S1_at	-2.2854114	down	-1.17422	down	-1.82884	down	-2.68357	down
Os.23101.1.A1_at	3.1837957	up	-1.7678	down	3.795732	up	1.800989	up

Os.7626.1.S1_at	2.0513442	up	-1.30111	down	2.793562	up	1.576614	up
Os.11623.1.S1_a_at	-1.9705013	down	-1.99064	down	-1.0785	down	-3.92256	down
AFFX-Os-r2-Bs-thr-M_	-1.6026171	down	1.034903	up	-2.22142	down	-1.54857	down
Os.27613.1.A1_at	-1.1889881	down	1.221298	up	1.757347	up	1.027174	up
Os.34174.1.S1_at	1.2120638	up	4.76535	up	-2.22046	down	5.775909	up
OsAffx.16618.1.S1_x_	-5.471557	down	3.336682	up	-7.16072	down	-1.63982	down
OsAffx.28093.1.S1_at	1.4319427	up	2.025214	up	1.059585	up	2.899989	up
Os.46461.1.A1_x_at	-1.7670289	down	-1.07541	down	-1.99501	down	-1.90028	down
Os.54727.1.S1_s_at	-1.9325138	down	1.606306	up	-3.4453	down	-1.20308	down
Os.16404.1.S1_at	2.7756066	up	-1.65333	down	3.545352	up	1.678799	up
OsAffx.32346.1.S1_at	1.1931518	up	-2.25806	down	1.053084	up	-1.89252	down
Os.51601.1.S1_at	-1.34116	down	-1.93655	down	-1.10721	down	-2.59723	down
Os.26853.1.A1_at	1.2660041	up	2.862009	up	-1.33508	down	3.623315	up
Os.54936.1.S1_at	10.409446	up	1.070481	up	2.002288	up	11.14311	up
Os.57484.1.S1_x_at	-1.5532837	down	-2.59696	down	1.211649	up	-4.03381	down
Os.15917.2.A1_at	1.5062859	up	1.80371	up	1.188189	up	2.716903	up
OsAffx.25715.1.S1_at	-2.8670466	down	-1.04296	down	-2.05419	down	-2.99022	down
OsAffx.14474.1.S1_at	1.6299901	up	1.637257	up	1.308407	up	2.668712	up
Os.9069.1.S1_at	-2.1877723	down	-1.05176	down	-2.0362	down	-2.30102	down
Os.8188.1.S1_s_at	-3.0644877	down	1.265537	up	-2.70984	down	-2.42149	down
Os.11789.1.S1_at	-1.1743981	down	1.38708	up	-2.96936	down	1.181098	up
Os.17960.1.S1_at	1.6876168	up	1.609249	up	1.330022	up	2.715795	up
OsAffx.18679.1.S1_at	-1.4811939	down	-1.86222	down	-1.14932	down	-2.7583	down
Os.39964.1.S1_at	1.504054	up	1.385457	up	1.544642	up	2.083803	up
OsAffx.18625.2.S1_at	-1.0874068	down	-1.51225	down	-1.41507	down	-1.64444	down
Os.46321.1.A1_s_at	-2.654031	down	-1.06282	down	-2.01325	down	-2.82075	down
Os.37797.1.A1_s_at	-2.138562	down	-1.71825	down	-1.24515	down	-3.67458	down
Os.27598.1.S1_at	1.1322445	up	2.254368	up	-1.05371	down	2.552496	up
Os.18064.1.S1_at	8.597224	up	-2.28044	down	4.877993	up	3.769992	up
OsAffx.2569.1.S1_at	2.756467	up	-2.01062	down	4.299544	up	1.370951	up
Os.14667.2.S1_at	1.1447791	up	2.042455	up	1.046957	up	2.33816	up
Os.4158.1.S1_at	1.7271214	up	1.003874	up	2.129668	up	1.733812	up
Os.55266.1.S1_at	-1.4290899	down	-1.0963	down	2.34334	up	-1.56671	down
Os.9875.1.S1_at	2.0766518	up	-1.27217	down	2.719199	up	1.632366	up
Os.26978.1.S1_a_at	-2.9836526	down	-1.33772	down	-1.59765	down	-3.9913	down
Os.38316.1.S1_s_at	1.8369976	up	2.835366	up	-1.32668	down	5.208561	up
OsAffx.7165.1.S1_at	-1.9632044	down	-1.53744	down	-1.38985	down	-3.0183	down
Os.23410.1.A1_at	1.5527434	up	1.493271	up	1.4307	up	2.318667	up
Os.15471.1.S1_at	-1.394327	down	-1.39116	down	-1.5357	down	-1.93973	down
OsAffx.15997.1.S1_s_	2.2137053	up	1.304795	up	1.637271	up	2.888433	up
Os.6124.1.S1_a_at	1.2513927	up	1.712669	up	1.247332	up	2.143221	up
Os.22652.1.S1_at	2.4413588	up	-1.27776	down	2.729415	up	1.910658	up
Os.8168.1.S1_at	-1.6711909	down	-1.05197	down	-2.03047	down	-1.75805	down
Os.6727.1.S1_at	1.8413156	up	1.2713	up	1.679421	up	2.340865	up
Os.10299.1.S1_at	1.8845153	up	-1.37115	down	2.927372	up	1.374405	up
Os.11864.1.S1_at	1.6332164	up	1.659125	up	1.286478	up	2.70971	up
Os.7959.1.S1_at	-1.6544826	down	-1.7952	down	-1.18859	down	-2.97013	down

Os.51494.1.S1_at	-1.6788465	down	-1.83499	down	-1.1628	down	-3.08066	down
OsAffx.19970.1.S1_x_	-1.7182349	down	-1.14118	down	-1.86973	down	-1.96082	down
Os.22907.1.S1_at	-3.7878473	down	-1.17611	down	-1.81372	down	-4.45492	down
Os.5363.1.S1_at	2.6683211	up	-1.33053	down	2.83816	up	2.005464	up
Os.7629.1.S1_at	2.826863	up	-1.47612	down	3.148057	up	1.915059	up
Os.16142.1.S1_at	1.5975739	up	1.64665	up	1.294527	up	2.630645	up
Os.20361.1.A1_at	-3.1446424	down	-1.01281	down	-2.10464	down	-3.18493	down
Os.23798.1.S1_at	1.6226616	up	1.48324	up	1.437106	up	2.406796	up
Os.54280.1.S1_at	1.082362	up	1.390801	up	1.532502	up	1.50535	up
Os.51760.2.S1_at	-1.8049222	down	-1.172	down	-1.8186	down	-2.11536	down
Os.46534.1.S1_at	1.3146944	up	1.660134	up	1.283577	up	2.182568	up
Os.53162.1.S1_s_at	-2.7731876	down	2.244569	up	-4.78206	down	-1.23551	down
Os.27753.1.S1_at	2.176374	up	1.046053	up	2.036247	up	2.276602	up
Os.7882.1.S1_at	-1.6929978	down	-1.5111	down	-1.40926	down	-2.55829	down
OsAffx.28942.1.S1_at	-2.395775	down	-1.92498	down	-1.10626	down	-4.61182	down
Os.5580.1.S1_at	-1.8038878	down	-1.50681	down	-1.41315	down	-2.71812	down
Os.28385.2.S1_a_at	-2.5180037	down	-1.12728	down	-1.88857	down	-2.83849	down
Os.3390.1.S1_at	4.610169	up	-1.62086	down	3.450349	up	2.844271	up
Os.47946.1.S1_s_at	8.036272	up	-1.52838	down	3.253347	up	5.258026	up
Os.4963.1.S1_at	-1.886107	down	-1.72194	down	-1.23609	down	-3.24777	down
OsAffx.30042.1.S1_x_	3.5000243	up	-1.35849	down	2.890829	up	2.57641	up
Os.55461.1.S1_at	1.4919182	up	-1.46298	down	3.112255	up	1.019783	up
Os.30077.1.S1_at	1.25915	up	1.325881	up	1.604151	up	1.669483	up
Os.6114.1.S1_at	-1.2255207	down	-1.57949	down	-1.34648	down	-1.93569	down
Os.15189.1.S1_at	-2.324484	down	-1.05241	down	-2.02076	down	-2.44631	down
OsAffx.13783.1.S1_at	1.168992	up	2.541344	up	-5.40431	down	2.970811	up
Os.38295.1.S2_at	-2.3315089	down	1.155634	up	-2.45667	down	-2.01752	down
Os.40417.1.A1_at	-1.9159942	down	4.174965	up	-1.96395	down	2.179007	up
Os.27022.1.A1_at	-2.3788328	down	-1.01926	down	-2.08551	down	-2.42464	down
Os.32357.1.S1_x_at	-1.8951979	down	-1.19423	down	-1.77989	down	-2.26331	down
Os.42814.1.S1_x_at	-1.5847495	down	-1.15188	down	-1.84518	down	-1.82544	down
Os.18913.1.S1_x_at	-2.5568457	down	2.130496	up	-4.52677	down	-1.20012	down
Os.5837.1.S1_at	-1.0439303	down	1.865633	up	1.138863	up	1.787124	up
Os.30965.1.S1_at	-1.7905517	down	-1.2844	down	-1.65273	down	-2.29979	down
OsAffx.14505.1.S1_x_	-2.0359375	down	1.248916	up	-2.65062	down	-1.63016	down
Os.8199.1.S1_at	-2.0110505	down	-1.44354	down	-1.46974	down	-2.90303	down
Os.52545.1.S1_at	-2.344598	down	1.171589	up	-2.48521	down	-2.00121	down
Os.29958.1.S1_at	1.02592	up	1.494705	up	1.419098	up	1.533448	up
Os.280.1.S1_at	-1.625659	down	-1.2072	down	-1.75697	down	-1.9625	down
Os.19401.1.S1_at	1.5127429	up	-1.12015	down	2.37496	up	1.350481	up
Os.25492.1.S1_at	2.1763802	up	-1.04813	down	2.222189	up	2.076448	up
OsAffx.26598.1.S1_x_	-1.6165289	down	-1.58192	down	-1.34022	down	-2.55722	down
OsAffx.19377.1.S1_at	-1.1489118	down	-1.38432	down	-1.53089	down	-1.59046	down
Os.35752.2.S1_x_at	1.8407936	up	1.102118	up	1.922719	up	2.028771	up
Os.25734.2.S1_a_at	1.0399811	up	1.155812	up	1.833194	up	1.202022	up
Os.56488.1.S1_at	1.4484167	up	1.035545	up	2.045574	up	1.4999	up
Os.7665.1.S1_at	1.0048538	up	-1.11349	down	2.356979	up	-1.10811	down

OsAffx.5436.1.S1_at	-1.1254201	down	-1.36227	down	-1.55357	down	-1.53313	down
Os.18316.1.S1_at	-2.1195054	down	-1.07843	down	-1.96247	down	-2.28574	down
Os.53676.1.S1_at	2.0701947	up	1.180494	up	1.792436	up	2.443853	up
Os.15498.1.S1_at	-2.724223	down	1.114175	up	-2.35647	down	-2.44506	down
Os.27955.1.S2_at	2.6819897	up	1.153652	up	1.833196	up	3.094084	up
Os.11875.1.S1_a_at	-1.7584612	down	-1.25523	down	-1.68479	down	-2.20727	down
Os.7812.1.S1_at	1.7875026	up	1.438804	up	1.469441	up	2.571866	up
OsAffx.31338.3.S1_at	-2.867369	down	1.611862	up	-3.4078	down	-1.77892	down
Os.11575.1.S1_a_at	1.4000056	up	3.022214	up	-1.4295	down	4.231117	up
Os.7452.1.S1_at	2.7195985	up	7.20025	up	-3.40625	down	19.58179	up
Os.14983.1.S1_a_at	-1.7583891	down	-1.78034	down	-1.18711	down	-3.13054	down
Os.26399.1.S1_at	-2.1136384	down	-1.32727	down	-1.59214	down	-2.80537	down
Os.12406.1.S1_at	1.7488525	up	1.623982	up	1.301091	up	2.840104	up
Os.11260.1.S1_at	52.073586	up	-127.945	down	270.3113	up	-2.457	down
Os.20722.1.S1_a_at	-2.766226	down	-1.04243	down	-2.02622	down	-2.88361	down
OsAffx.12938.1.S1_at	-2.446964	down	-1.41543	down	-1.49212	down	-3.46351	down
Os.5678.2.S1_at	1.8568304	up	1.086706	up	1.942669	up	2.017828	up
Os.14381.1.S1_at	-2.5305252	down	-1.32577	down	-1.59231	down	-3.35489	down
Os.27089.1.S1_at	-1.6925594	down	-1.34405	down	-1.57057	down	-2.27488	down
Os.12693.5.S1_x_at	2.3812566	up	-1.08764	down	2.295792	up	2.189374	up
Os.53004.1.S1_at	1.4259304	up	1.241328	up	1.700346	up	1.770048	up
Os.52590.1.S1_at	1.4910007	up	-1.16653	down	2.461824	up	1.278152	up
Os.53052.1.S1_at	-1.0167522	down	2.573499	up	-1.22005	down	2.531097	up
Os.12092.4.S1_at	1.6066405	up	1.084024	up	1.945451	up	1.741636	up
Os.5697.1.S1_at	-1.0422084	down	4.056003	up	-1.92331	down	3.891738	up
Os.5193.1.S1_at	2.5706434	up	2.090343	up	1.008743	up	5.373526	up
Os.16248.1.S1_at	1.1764063	up	2.033755	up	1.036731	up	2.392522	up
Os.11458.1.S1_at	2.307612	up	1.05918	up	1.990381	up	2.444176	up
OsAffx.10973.2.S1_s_	2.5458229	up	-1.71562	down	3.616603	up	1.48391	up
Os.8810.1.S1_at	-1.9904273	down	-1.54845	down	-1.36108	down	-3.08208	down
Os.53061.1.S1_at	1.9252504	up	1.270366	up	1.658807	up	2.445773	up
Os.8633.1.S1_a_at	1.2945689	up	3.926365	up	-1.86334	down	5.082951	up
Os.27128.1.A1_a_at	-3.4319673	down	1.161611	up	-2.44758	down	-2.95449	down
OsAffx.4254.1.S1_at	-1.674558	down	-1.18645	down	-1.77593	down	-1.98678	down
Os.4604.1.S1_at	2.076961	up	1.329389	up	1.584862	up	2.761088	up
Os.15918.1.S1_x_at	1.1372942	up	-1.551	down	3.266998	up	-1.36377	down
Os.51208.1.S1_s_at	1.2109011	up	1.226246	up	1.717694	up	1.484863	up
Os.50630.1.S1_x_at	-1.6347157	down	-1.43481	down	-1.46783	down	-2.34551	down
Os.13474.1.S1_at	-2.6006086	down	-1.27676	down	-1.64937	down	-3.32034	down
Os.7198.1.S1_at	1.7231647	up	-1.23587	down	2.602343	up	1.394293	up
OsAffx.24172.2.S1_at	-1.5549046	down	-1.71362	down	3.60827	up	-2.66451	down
Os.1082.1.S1_a_at	-1.1923547	down	-1.80137	down	-1.16878	down	-2.14787	down
Os.8600.1.S1_at	1.4487115	up	1.315355	up	1.60046	up	1.90557	up
Os.10614.1.S1_at	-1.8032469	down	-1.10069	down	-1.9125	down	-1.98481	down
Os.51015.1.S1_at	-1.3628541	down	1.564416	up	1.345045	up	1.147897	up
Os.18866.1.A1_at	-2.2333152	down	-1.27173	down	-1.65459	down	-2.84018	down
Os.51063.1.S1_at	5.111158	up	-1.03281	down	2.173076	up	4.948781	up

Os.33758.2.S1_s_at	-1.2461959	down	-1.95953	down	-1.07372	down	-2.44196	down
Os.38046.1.S1_s_at	-1.3022768	down	-1.07492	down	-1.95697	down	-1.39985	down
Os.34459.1.S1_at	2.1057894	up	-1.45225	down	3.054914	up	1.450016	up
Os.21282.1.S1_at	-2.003806	down	1.094669	up	-2.30236	down	-1.83051	down
Os.6676.1.S1_at	-2.4374912	down	-1.21459	down	-1.73157	down	-2.96056	down
Os.44516.1.S1_x_at	-2.0849693	down	-1.35296	down	-1.55439	down	-2.82088	down
Os.32597.1.S1_x_at	1.8981799	up	-2.75323	down	1.309397	up	-1.45046	down
Os.46492.1.S1_at	1.6402531	up	-1.82239	down	3.831198	up	-1.11104	down
Os.18351.2.S1_at	1.0422885	up	1.650517	up	1.273062	up	1.720315	up
OsAffx.12962.1.S1_at	1.452437	up	1.001616	up	2.097794	up	1.454785	up
OsAffx.24206.2.S1_at	1.1808342	up	1.354953	up	1.550593	up	1.599975	up
Os.25687.1.S1_at	1.2502383	up	4.687602	up	-2.23117	down	5.86062	up
Os.4638.1.S1_at	-1.2838312	down	-1.41878	down	2.980766	up	-1.82148	down
Os.55254.1.S1_at	2.441396	up	-1.1182	down	2.349019	up	2.183335	up
OsAffx.9584.1.S1_x_a	4.057087	up	2.486291	up	-1.18362	down	10.0871	up
Os.42422.1.S1_at	-2.5131524	down	1.442958	up	-3.03097	down	-1.74167	down
Os.12701.1.S1_at	-1.9048306	down	1.960354	up	-4.11618	down	1.029149	up
Os.12007.1.S1_at	-7.4302154	down	1.842268	up	-3.86806	down	-4.03319	down
Os.29093.1.S1_at	2.010235	up	-1.9537	down	4.101574	up	1.02894	up
Os.26261.1.S1_at	3.5133002	up	-1.01526	down	2.131086	up	3.460477	up
Os.22629.1.S1_at	1.2459593	up	-1.42499	down	-1.47241	down	-1.14369	down
OsAffx.26443.1.S1_s_	1.770909	up	1.808102	up	1.160042	up	3.201984	up
Os.35708.1.S1_at	-1.8114837	down	1.963212	up	1.068362	up	1.083759	up
Os.54624.1.S1_at	2.1329877	up	-1.0925	down	2.290922	up	1.952391	up
Os.4782.1.S1_at	3.3829026	up	-2.39227	down	5.015865	up	1.414097	up
Os.24338.1.A1_at	-1.9744846	down	-1.05964	down	-1.97855	down	-2.09225	down
OsAffx.23090.9.S1_x_	-2.5591564	down	1.479285	up	-3.10067	down	-1.73	down
Os.53614.1.S1_at	-1.5895611	down	-1.80062	down	-1.16396	down	-2.86219	down
AFFX-OS-5.8SrRNA_a	1.1993082	up	-1.81595	down	-1.1541	down	-1.51416	down
Os.5450.1.S1_at	-2.1903198	down	-1.69269	down	-1.2381	down	-3.70753	down
OsAffx.31667.1.S1_at	-2.0353482	down	-1.454	down	-1.44133	down	-2.9594	down
Os.24863.1.A1_at	1.8965569	up	2.205661	up	-1.05272	down	4.183161	up
Os.52648.1.S1_at	1.8343875	up	1.100555	up	1.903679	up	2.018844	up
Os.12636.1.S1_at	1.2818131	up	1.467389	up	1.426891	up	1.880918	up
OsAffx.30784.1.S1_at	3.2028487	up	4.07692	up	-1.94716	down	13.05776	up
Os.14929.1.S1_at	-4.2354317	down	-1.27037	down	-1.64796	down	-5.38055	down
Os.53409.1.A1_at	1.6751446	up	1.17205	up	1.785999	up	1.963353	up
Os.27457.1.S1_at	-2.2790575	down	-1.20597	down	-1.73568	down	-2.74847	down
Os.6789.1.S1_at	1.8927661	up	1.877556	up	1.114834	up	3.553774	up
Os.8593.1.S1_at	1.1534461	up	2.030444	up	1.030698	up	2.342007	up
Os.10088.1.S1_a_at	-1.8819255	down	1.025299	up	-2.14551	down	-1.83549	down
Os.47949.1.S1_a_at	-3.5790973	down	-1.62536	down	-1.28735	down	-5.81731	down
Os.52722.1.S1_at	-2.5997488	down	-1.88277	down	-1.11117	down	-4.89473	down
Os.6107.1.A1_at	2.1068428	up	1.326038	up	1.577621	up	2.793754	up
Os.49771.1.S1_at	-2.5173407	down	1.352257	up	-2.82872	down	-1.86158	down
Os.9848.1.S1_at	2.0900261	up	1.376034	up	1.519937	up	2.875947	up
Os.55494.1.S1_at	5.1497655	up	-1.19309	down	2.495179	up	4.316343	up

Os.52534.1.S1_x_at	2.1392758	up	-1.02975	down	2.152782	up	2.077467	up
Os.22699.1.S1_at	-1.328104	down	-1.63545	down	-1.27824	down	-2.17205	down
Os.14762.2.S1_x_at	-2.667949	down	-1.13751	down	-1.83778	down	-3.03481	down
Os.165.1.S1_at	2.4702091	up	1.146659	up	1.823005	up	2.832488	up
Os.9538.1.S1_s_at	1.6384017	up	1.280253	up	1.632495	up	2.097568	up
Os.6419.1.S1_at	-1.8518091	down	-1.44567	down	-1.44558	down	-2.67711	down
Os.49182.1.S1_at	1.7456024	up	2.646355	up	-1.26645	down	4.619484	up
Os.48438.1.S1_at	-3.039634	down	-1.30122	down	-1.60565	down	-3.95523	down
Os.5421.1.S1_at	-1.5928653	down	-1.27919	down	-1.63319	down	-2.03757	down
Os.34520.1.S1_at	1.6143228	up	3.246864	up	-1.55449	down	5.241488	up
Os.51736.1.S1_at	-1.6494315	down	1.042986	up	-2.17804	down	-1.58145	down
Os.7158.1.S1_a_at	2.6338983	up	-1.57945	down	3.298241	up	1.667601	up
Os.14765.1.S1_at	-1.5899456	down	-1.35306	down	-1.54322	down	-2.15129	down
Os.50486.1.S1_at	-2.3170874	down	-1.02566	down	-2.03562	down	-2.37655	down
Os.17276.1.S1_at	-1.2595176	down	1.205528	up	1.731493	up	-1.04478	down
OsAffx.4833.1.S1_x_a	-1.0976545	down	2.996729	up	-1.43601	down	2.73012	up
Os.8931.1.S1_x_at	-2.9564016	down	1.637366	up	-3.41522	down	-1.80558	down
Os.7075.1.S1_at	1.439538	up	2.721908	up	-1.30521	down	3.918289	up
Os.11061.1.S1_at	-3.2279432	down	-1.03164	down	-2.02138	down	-3.33008	down
Os.52106.1.S1_at	-1.6929536	down	1.621854	up	1.285417	up	-1.04384	down
Os.47600.2.S1_x_at	1.03436	up	1.383131	up	1.507133	up	1.430655	up
Os.6846.1.S1_at	1.3192195	up	-1.07791	down	2.246745	up	1.223872	up
OsAffx.31976.1.S1_at	-1.1451893	down	-2.2728	down	1.090464	up	-2.60279	down
Os.49529.1.S1_at	-2.867155	down	1.273349	up	-2.6535	down	-2.25166	down
Os.47461.1.S2_at	-2.1905224	down	1.096498	up	-2.28405	down	-1.99774	down
Os.9869.1.S1_at	1.7067267	up	1.315982	up	1.582845	up	2.246022	up
Os.9152.1.S1_at	1.3843334	up	-1.41503	down	2.94728	up	-1.02218	down
OsAffx.27399.1.S1_at	-3.5898283	down	1.56251	up	-3.25359	down	-2.29748	down
Os.8353.1.S1_at	-1.2744818	down	-1.56527	down	-1.3303	down	-1.99491	down
Os.21345.1.S1_at	-4.5008836	down	-1.28703	down	-1.61763	down	-5.79279	down
Os.52596.1.S1_x_at	-2.4831011	down	-1.42178	down	-1.46417	down	-3.53043	down
OsAffx.28964.1.S1_at	1.1883105	up	1.431165	up	1.454399	up	1.700669	up
Os.23672.1.S1_at	-3.2252526	down	-1.01122	down	-2.05736	down	-3.26143	down
OsAffx.17329.1.S1_s_	1.3867624	up	-1.45737	down	3.031298	up	-1.05091	down
Os.26820.1.A1_at	1.279114	up	8.325118	up	-4.0025	down	10.64878	up
Os.52266.1.S1_at	-1.2503026	down	3.256662	up	-1.56599	down	2.6047	up
Os.24302.1.S1_at	-2.0692816	down	-1.08986	down	-1.90809	down	-2.25523	down
Os.34273.1.S1_at	-3.3620281	down	1.350453	up	-2.80817	down	-2.48956	down
Os.19417.1.S1_a_at	-1.6001714	down	-1.49027	down	-1.39521	down	-2.38469	down
OsAffx.28610.1.S1_x_	1.1563666	up	1.447937	up	1.435895	up	1.674346	up
Os.5687.1.S1_at	-1.8336455	down	1.288955	up	-2.679	down	-1.42258	down
Os.46819.2.S1_x_at	1.1886435	up	1.602142	up	1.29723	up	1.904376	up
Os.51071.1.A1_x_at	-3.2605894	down	-1.34619	down	-1.54388	down	-4.38936	down
Os.16982.1.S1_at	1.3096459	up	1.467307	up	1.416221	up	1.921652	up
Os.13846.1.S1_at	-3.1005938	down	1.026358	up	-2.13189	down	-3.02097	down
Os.4801.1.S1_x_at	-1.4036549	down	2.948458	up	-1.4201	down	2.100558	up
Os.14185.2.S1_a_at	-1.7521492	down	1.170387	up	-2.42977	down	-1.49707	down

Os.38092.1.S1_at	-1.6300417	down	-1.27897	down	-1.62321	down	-2.08477	down
Os.46576.1.S1_at	-1.0276749	down	1.40136	up	1.481032	up	1.363622	up
Os.21210.1.S1_at	-1.2268329	down	1.580748	up	1.312369	up	1.288479	up
Os.49974.1.S1_at	-1.3615258	down	-1.4471	down	-1.43303	down	-1.97027	down
Os.8892.1.S1_at	1.3303887	up	1.453192	up	1.426822	up	1.93331	up
OsAffx.23031.1.S1_s_	-1.048702	down	-2.21656	down	1.069232	up	-2.32451	down
Os.52491.1.A1_at	-2.0868838	down	1.57514	up	-3.26506	down	-1.32489	down
Os.23649.1.S1_a_at	1.1543856	up	2.162735	up	-1.04344	down	2.49663	up
Os.3246.1.S1_at	-2.8174477	down	-1.05683	down	-1.96114	down	-2.97756	down
Os.10097.1.S1_at	3.428382	up	-1.26982	down	2.63163	up	2.699895	up
Os.47410.1.S1_at	2.3592598	up	1.594798	up	1.299423	up	3.762543	up
Os.8113.2.S1_x_at	2.9987476	up	1.08166	up	1.91578	up	3.243625	up
Os.51026.1.S1_at	-1.2578427	down	1.758334	up	1.178481	up	1.397897	up
Os.8796.2.S1_a_at	-1.9724088	down	1.104516	up	-2.28846	down	-1.78577	down
OsAffx.32329.1.S1_x_	-1.1924487	down	-1.484	down	-1.39607	down	-1.7696	down
AFFX-r2-Bs-phe-5_at	-1.4108083	down	1.074456	up	-2.22598	down	-1.31304	down
Os.40388.1.S1_at	2.062796	up	-1.14433	down	2.370717	up	1.802624	up
Os.52767.1.A1_at	-3.2270277	down	1.431914	up	-2.96634	down	-2.25365	down
Os.57358.1.S1_at	2.6634085	up	1.318725	up	1.570445	up	3.512302	up
Os.27216.1.A1_at	-2.698327	down	1.148993	up	-2.3788	down	-2.34843	down
Os.57187.1.S1_at	-2.640201	down	-1.30527	down	-1.58558	down	-3.44616	down
Os.11327.1.S1_at	1.6372403	up	-2.46209	down	5.09499	up	-1.50381	down
Os.53706.1.S1_at	-3.5285037	down	1.089712	up	-2.25299	down	-3.23802	down
Os.10428.1.S1_at	-2.4790015	down	2.607979	up	-5.39079	down	1.052028	up
Os.6325.1.A1_x_at	-1.4332191	down	-1.73977	down	-1.18795	down	-2.49347	down
Os.28114.2.S1_at	-3.3324904	down	1.486551	up	-3.07156	down	-2.24176	down
Os.9542.1.A1_at	-2.7645092	down	-1.23719	down	-1.66994	down	-3.42022	down
Os.7168.1.S1_at	1.6802949	up	1.338937	up	1.542958	up	2.249808	up
Os.49742.1.S1_at	2.0175743	up	1.419779	up	1.45493	up	2.864511	up
Os.26271.1.A1_s_at	-2.661831	down	1.04714	up	-2.16296	down	-2.542	down
OsAffx.13444.1.S1_at	-1.4069939	down	-1.55437	down	-1.32871	down	-2.18699	down
OsAffx.6248.1.S1_at	-1.5557657	down	-1.56452	down	-1.31999	down	-2.43402	down
Os.15809.1.S1_at	-1.074526	down	-2.11694	down	4.371525	up	-2.27471	down
Os.46546.1.S1_at	1.0467141	up	2.844765	up	-1.37761	down	2.977656	up
OsAffx.25288.1.S1_s_	-4.7815747	down	1.122653	up	-2.31801	down	-4.25918	down
Os.10197.1.S1_at	1.3679368	up	-1.49606	down	3.088738	up	-1.09366	down
Os.8040.1.S1_a_at	1.0707453	up	1.167179	up	1.768659	up	1.249752	up
Os.51797.1.S1_x_at	-2.2024755	down	-1.13479	down	-1.81899	down	-2.49935	down
Os.53527.1.S1_at	-1.644454	down	-1.68602	down	-1.22411	down	-2.77258	down
Os.54179.1.S1_at	-1.6497442	down	-1.16362	down	-1.77351	down	-1.91968	down
OsAffx.10944.1.S1_x_	1.7145594	up	1.861333	up	1.108562	up	3.191366	up
Os.5367.1.S1_at	3.0257523	up	-1.26496	down	2.60978	up	2.391977	up
Os.50416.1.S1_at	-2.3541882	down	-1.03302	down	-1.99635	down	-2.43194	down
Os.7014.1.S1_at	1.8718598	up	-1.02574	down	2.114968	up	1.82489	up
Os.57511.1.A1_at	-1.0578392	down	-1.25985	down	2.597192	up	-1.33272	down
Os.55524.1.S1_at	-1.5792866	down	-1.15099	down	-1.79077	down	-1.81774	down
Os.50290.1.S1_at	-2.6405058	down	1.114491	up	-2.29698	down	-2.36925	down

Os.18231.1.S1_at	-1.4057549	down	-1.13838	down	-1.81034	down	-1.60029	down
Os.25309.2.A1_a_at	1.5485004	up	-1.13292	down	2.333905	up	1.366825	up
Os.52036.1.S1_at	2.85723	up	-5.9106	down	12.17537	up	-2.06865	down
OsAffx.12051.1.S1_s_	-1.8849559	down	-1.57618	down	-1.30674	down	-2.97102	down
Os.4709.1.S1_a_at	-2.09376	down	-1.26387	down	-1.62935	down	-2.64625	down
Os.12044.1.S1_at	1.1740317	up	1.76596	up	1.165767	up	2.073293	up
Os.54130.1.S1_at	-1.958659	down	-1.04335	down	-1.97311	down	-2.04356	down
Os.48535.1.A1_at	1.0808107	up	1.366454	up	1.506531	up	1.476878	up
Os.57530.1.S1_at	-2.1332312	down	-1.18384	down	-1.73884	down	-2.52541	down
Os.7609.2.S1_a_at	1.9691485	up	3.118809	up	-1.51509	down	6.141398	up
Os.315.1.S1_at	-2.6756837	down	-1.16043	down	-1.77362	down	-3.10495	down
Os.11655.1.S1_at	-2.1408339	down	1.466065	up	-3.01663	down	-1.46026	down
Os.5500.1.S1_s_at	1.0764229	up	2.695032	up	-1.31001	down	2.900995	up
Os.17424.1.S1_at	-2.4879677	down	1.347892	up	-2.7729	down	-1.84582	down
Os.5782.1.S1_at	-1.5511898	down	-1.23554	down	-1.66488	down	-1.91655	down
Os.50736.1.A1_at	-1.2843809	down	-1.25927	down	-1.63317	down	-1.61738	down
Os.52243.1.S1_at	-2.4999692	down	1.032362	up	-2.12313	down	-2.4216	down
Os.39960.1.S1_at	-1.2804757	down	-1.26009	down	-1.63188	down	-1.61351	down
Os.51480.1.S1_at	2.152258	up	-2.41145	down	4.957665	up	-1.12043	down
OsAffx.16075.1.S1_s_	-2.3620222	down	1.370082	up	-2.81638	down	-1.724	down
AFFX-PheX-5_at	-1.4219236	down	1.078484	up	-2.21668	down	-1.31845	down
Os.11602.1.S1_at	-1.1516576	down	-1.12356	down	2.309192	up	-1.29396	down
OsAffx.4527.1.S1_s_a	-1.8580679	down	1.808696	up	-3.71654	down	-1.0273	down
Os.40187.1.S1_at	3.4359016	up	-1.4217	down	2.920535	up	2.416748	up
Os.9653.1.S1_at	2.1463096	up	1.186875	up	1.730681	up	2.5474	up
Os.13625.1.S1_at	-1.592188	down	-1.16641	down	-1.76096	down	-1.85715	down
Os.49256.1.S1_at	3.1114697	up	-1.22093	down	2.507762	up	2.54844	up
OsAffx.18042.1.S1_at	1.6958901	up	1.248718	up	1.644823	up	2.117689	up
OsAffx.6465.1.S1_at	-1.0695295	down	1.686805	up	1.217541	up	1.577147	up
Os.51762.1.S1_at	2.5132422	up	1.159877	up	1.770513	up	2.915051	up
Os.11442.1.S1_at	-1.5756861	down	-1.8204	down	-1.12784	down	-2.86838	down
OsAffx.17158.1.S1_at	1.1557002	up	-1.60293	down	3.290822	up	-1.38697	down
Os.50821.1.S1_at	-3.4370234	down	-1.12217	down	-1.82948	down	-3.85691	down
Os.17939.1.S1_x_at	-2.1442192	down	-1.28042	down	-1.60306	down	-2.7455	down
Os.54932.1.S1_at	6.3461246	up	-1.32116	down	2.711428	up	4.803436	up
Os.8150.1.S1_at	-1.6072462	down	-1.04045	down	-1.97233	down	-1.67226	down
OsAffx.24832.1.S1_at	1.6883674	up	-1.03018	down	2.113946	up	1.638904	up
Os.51241.1.S1_at	-2.439091	down	-1.74734	down	-1.17396	down	-4.26192	down
Os.36152.1.S1_at	3.1204755	up	1.306023	up	1.570607	up	4.075414	up
Os.50233.1.S1_at	-3.0133858	down	-1.12762	down	-1.81885	down	-3.39796	down
Os.39828.1.A1_s_at	-2.0587819	down	-1.25492	down	-1.63421	down	-2.5836	down
Os.10125.1.S1_a_at	1.2879847	up	2.008171	up	1.021043	up	2.586494	up
Os.26469.1.S1_at	1.5083163	up	1.157488	up	1.770848	up	1.745858	up
Os.8241.1.S1_at	2.4560575	up	1.307003	up	1.568207	up	3.210074	up
Os.16682.2.S1_x_at	-1.6732651	down	-1.62984	down	-1.2574	down	-2.72715	down
Os.27525.1.A1_at	-1.4510611	down	-2.03753	down	-1.00578	down	-2.95659	down
Os.12410.3.S1_x_at	-1.2481099	down	1.341219	up	-2.74855	down	1.0746	up

Os.52379.1.S1_s_at	1.0055645	up	2.507641	up	-1.22376	down	2.521594	up
Os.5887.1.S1_a_at	-2.1599858	down	-1.34347	down	-1.52487	down	-2.90188	down
Os.50975.1.S1_at	-2.384647	down	1.273751	up	-2.60941	down	-1.87214	down
Os.6984.1.S1_at	-2.205708	down	1.06564	up	-2.18263	down	-2.06984	down
Os.51544.1.S1_at	-2.3498902	down	-1.20058	down	-1.70493	down	-2.82122	down
Os.7005.1.S1_s_at	-1.3104221	down	-1.6166	down	-1.26597	down	-2.11842	down
Os.35688.1.S1_at	-1.7757304	down	1.162344	up	-2.37843	down	-1.52772	down
OsAffx.22679.1.S1_x_	1.9455863	up	1.385054	up	1.477354	up	2.694742	up
Os.51616.1.S1_at	-1.8978709	down	-1.17352	down	-1.74339	down	-2.2272	down
Os.33878.1.S1_at	-2.0566144	down	1.033415	up	-2.11412	down	-1.99012	down
OsAffx.12645.1.S1_s_	-2.6057396	down	1.160817	up	-2.37434	down	-2.24475	down
Os.22652.2.S1_x_at	1.922241	up	1.290709	up	1.584529	up	2.481054	up
Os.48001.1.A1_at	-1.5685004	down	-1.72073	down	-1.18832	down	-2.69897	down
Os.24275.1.S1_at	1.6860639	up	1.070638	up	1.909832	up	1.805164	up
Os.7187.1.S1_at	2.97456	up	15.17919	up	-7.42387	down	45.1514	up
OsAffx.28085.1.S1_x_	-1.7704527	down	-1.12591	down	-1.81582	down	-1.99337	down
OsAffx.13059.1.S1_s_	1.0887927	up	-1.51847	down	-1.34627	down	-1.39464	down
Os.52577.1.S1_x_at	2.0454783	up	1.109872	up	1.841485	up	2.270219	up
Os.33019.1.S1_at	-2.9408813	down	1.234099	up	-2.5222	down	-2.38302	down
Os.7401.1.S1_a_at	-1.6034652	down	-1.10929	down	-1.84152	down	-1.7787	down
Os.8150.2.S1_x_at	-1.5307893	down	1.037467	up	-2.11901	down	-1.47551	down
Os.10356.1.S1_at	3.9262762	up	1.564518	up	1.305097	up	6.142727	up
Os.15039.1.S1_at	-1.564228	down	-1.04177	down	-1.95944	down	-1.62956	down
OsAffx.7086.1.S1_at	-1.6598586	down	1.344646	up	-2.74402	down	-1.23442	down
Os.6650.1.S1_at	-1.5540879	down	-1.6288	down	-1.2528	down	-2.53129	down
OsAffx.26318.1.S1_at	2.5536418	up	1.399029	up	1.458046	up	3.572619	up
Os.5357.1.S1_at	1.1620609	up	-1.16039	down	2.366095	up	1.001443	up
Os.15802.2.S1_x_at	-1.847958	down	-1.15199	down	-1.77001	down	-2.12883	down
Os.6424.1.S1_at	2.134102	up	-1.35717	down	2.767299	up	1.572465	up
Os.23266.1.S1_at	2.2669978	up	1.247912	up	1.633932	up	2.829015	up
Os.9172.1.S1_x_at	-1.6767687	down	1.213406	up	-2.47409	down	-1.38187	down
Os.5087.1.S1_at	-2.4079587	down	-1.80769	down	-1.12793	down	-4.35283	down
Os.16237.1.S1_at	2.8997836	up	-1.25967	down	2.56822	up	2.302017	up
Os.14823.1.S1_s_at	-1.4107739	down	-1.36048	down	-1.49852	down	-1.91933	down
Os.49632.1.S1_a_at	-1.0742484	down	-2.16722	down	1.063084	up	-2.32814	down
Os.46529.1.S1_a_at	-1.6498555	down	-1.33759	down	-1.52396	down	-2.20682	down
Os.53542.1.S1_x_at	-2.0724015	down	-1.15288	down	-1.76796	down	-2.38923	down
Os.24569.1.S2_at	-1.9694161	down	-1.54168	down	-1.32205	down	-3.03622	down
Os.55822.1.A1_at	-3.396876	down	1.281849	up	-2.61226	down	-2.64998	down
Os.11904.1.S1_at	-1.9271955	down	-1.24073	down	-1.64238	down	-2.39112	down
Os.12498.1.S1_at	1.306386	up	-9.50361	down	4.664955	up	-7.27473	down
Os.9317.1.S1_at	1.1530569	up	1.40686	up	1.447958	up	1.62219	up
Os.6656.1.S1_at	1.5062118	up	2.0867	up	-1.02455	down	3.143011	up
Os.29966.1.S1_x_at	-3.053211	down	1.700461	up	-3.46247	down	-1.79552	down
Os.53817.1.S1_at	-1.1311022	down	2.921324	up	-1.43505	down	2.582724	up
OsAffx.32225.1.A1_a	1.4313816	up	-2.57918	down	1.267002	up	-1.80188	down
Os.53502.1.S1_at	2.7783277	up	1.002034	up	2.031297	up	2.783979	up

Os.5049.1.S1_at	2.1800885	up	1.308838	up	1.555036	up	2.853382	up
Os.13015.1.S1_at	1.0210416	up	-1.4709	down	-1.38365	down	-1.44059	down
OsAffx.6051.1.S1_at	3.4522598	up	-1.26358	down	2.571074	up	2.732118	up
Os.53009.1.A1_x_at	-4.4244103	down	3.450604	up	-7.02098	down	-1.28221	down
OsAffx.3240.1.S1_s_a	2.4316523	up	1.011523	up	2.011102	up	2.459672	up
Os.45516.2.S1_x_at	-5.203689	down	1.883088	up	-3.83059	down	-2.76338	down
Os.28192.1.S1_at	-2.007551	down	-1.02263	down	-1.98901	down	-2.05299	down
Os.10294.1.S1_at	1.4974825	up	1.386892	up	1.466511	up	2.076847	up
Os.12348.2.S1_at	-1.3739597	down	1.06438	up	1.910467	up	-1.29086	down
OsAffx.32241.1.S1_x_	-1.6137358	down	1.106028	up	-2.249	down	-1.45904	down
Os.28055.1.S1_at	-1.774111	down	-1.32773	down	-1.53123	down	-2.35553	down
Os.53177.1.S1_s_at	2.8232534	up	1.153983	up	1.761406	up	3.257987	up
Os.34066.1.S1_at	1.1575819	up	1.231051	up	1.651134	up	1.425042	up
Os.11139.1.S1_at	1.621591	up	1.052228	up	1.931587	up	1.706284	up
Os.11918.1.S1_at	-2.5994022	down	-1.20734	down	-1.68324	down	-3.13836	down
Os.10222.1.S1_s_at	-1.5388062	down	-1.46122	down	-1.39048	down	-2.24853	down
Os.21454.1.S1_at	-2.1231904	down	1.257167	up	-2.55397	down	-1.68887	down
OsAffx.27815.1.S1_s_	2.3970342	up	1.560763	up	1.301343	up	3.741201	up
Os.38258.1.S1_x_at	1.5917915	up	1.093682	up	1.857029	up	1.740915	up
Os.34273.2.S1_x_at	-2.4704201	down	1.011352	up	-2.05362	down	-2.44269	down
Os.26381.3.S1_x_at	-1.3267448	down	-1.38558	down	-1.46546	down	-1.83831	down
Os.35065.1.S1_at	-2.5814033	down	1.237387	up	-2.51244	down	-2.08617	down
OsAffx.25082.1.S1_s_	3.1426098	up	-1.72172	down	3.495635	up	1.825275	up
Os.27778.3.S1_x_at	-2.80662	down	1.083008	up	-2.19826	down	-2.5915	down
Os.16785.1.S1_at	-2.027928	down	1.050922	up	-2.13276	down	-1.92967	down
Os.26698.4.S1_s_at	-2.9193604	down	-1.64781	down	-1.2315	down	-4.81056	down
Os.18513.1.S1_at	1.1760433	up	2.106489	up	-1.03834	down	2.477322	up
Os.55105.1.S1_at	2.1984453	up	1.274259	up	1.59185	up	2.801388	up
Os.20803.1.S1_a_at	-1.887265	down	1.110304	up	-2.25204	down	-1.69977	down
Os.45678.1.S1_at	-2.3766084	down	-1.18036	down	-1.71765	down	-2.80524	down
Os.48500.1.A1_s_at	1.1855677	up	1.615145	up	1.255137	up	1.914863	up
Os.6662.1.S1_at	1.77915	up	-1.07799	down	2.185189	up	1.650438	up
Os.19617.1.S1_at	1.7827127	up	1.952789	up	1.037976	up	3.481262	up
OsAffx.18633.1.S1_s_	-1.0387917	down	-1.28918	down	2.61226	up	-1.33919	down
OsAffx.22569.2.S1_at	-1.1977657	down	1.739668	up	1.164585	up	1.452427	up
Os.17065.1.S1_at	-1.282883	down	1.086712	up	-2.20087	down	-1.18052	down
Os.23810.1.A1_at	-3.1972303	down	-1.00313	down	-2.01865	down	-3.20724	down
OsAffx.14202.1.S1_at	-2.399697	down	2.759924	up	-5.58703	down	1.150113	up
Os.27550.1.S1_at	1.8227746	up	3.228264	up	-1.59485	down	5.884398	up
Os.24995.1.S1_a_at	1.0709524	up	-1.79183	down	3.626956	up	-1.67312	down
Os.18640.1.S1_at	2.5092862	up	1.100692	up	1.838747	up	2.761951	up
Os.15942.2.S1_at	1.3610092	up	1.099666	up	1.840447	up	1.496655	up
Os.15894.1.A1_a_at	-1.2334197	down	1.99046	up	-4.0282	down	1.613773	up
Os.7536.1.S1_at	1.7345958	up	1.995854	up	1.013794	up	3.462001	up
OsAffx.21010.1.S1_at	1.0310162	up	1.531355	up	1.321163	up	1.578852	up
Os.10595.2.S1_at	1.9435222	up	1.085611	up	1.863398	up	2.109908	up
Os.11886.1.S1_at	-1.2210611	down	-1.25006	down	-1.61815	down	-1.5264	down

Os.24698.1.S1_at	-1.1475763	down	-1.9691	down	3.982392	up	-2.2597	down
Os.46642.1.S1_at	1.8090873	up	-1.14455	down	2.313249	up	1.580603	up
OsAffx.32290.1.S1_at	-1.075048	down	-1.23306	down	-1.63892	down	-1.3256	down
Os.6205.2.S1_at	-2.1372466	down	1.03343	up	-2.08802	down	-2.06811	down
Os.17334.2.S1_x_at	1.2231948	up	1.067484	up	1.892496	up	1.305741	up
Os.46647.1.S1_at	-1.1653355	down	-2.07904	down	1.029344	up	-2.42278	down
Os.49268.1.S1_at	4.3002653	up	-1.00271	down	2.024744	up	4.288652	up
Os.56237.1.S1_at	-1.6432201	down	1.708271	up	-3.44923	down	1.039588	up
Os.26511.1.S1_at	-1.0031822	down	-1.82071	down	-1.10894	down	-1.82651	down
Os.33605.2.S1_x_at	1.0566449	up	2.297006	up	-1.13769	down	2.42712	up
Os.52627.1.S1_at	-4.2018213	down	2.544449	up	-5.13717	down	-1.65137	down
Os.17435.1.S1_s_at	-2.1872807	down	-1.00003	down	-2.01857	down	-2.18735	down
OsAffx.2509.1.S1_at	-2.3381867	down	1.50213	up	-3.0321	down	-1.55658	down
OsAffx.8000.1.S1_x_a	-1.7802385	down	-1.33525	down	-1.5117	down	-2.37706	down
Os.54716.1.S1_x_at	1.7430286	up	1.211888	up	1.66543	up	2.112355	up
Os.4679.1.S1_at	1.1444699	up	1.136963	up	1.775094	up	1.30122	up
Os.12994.1.S1_at	1.4998742	up	1.737689	up	1.161367	up	2.606314	up
Os.52244.2.S1_x_at	-2.7916958	down	1.223373	up	-2.4688	down	-2.28197	down
Os.411.1.S1_a_at	5.5716095	up	-3.41736	down	6.89311	up	1.630384	up
Os.5098.1.S1_at	1.472084	up	1.732174	up	1.164476	up	2.549905	up
OsAffx.27501.1.S1_at	1.1517336	up	1.450561	up	1.390525	up	1.67066	up
Os.11867.1.S1_at	1.1453569	up	1.130139	up	1.784193	up	1.294413	up
Os.329.1.S1_at	1.7788471	up	1.881871	up	1.071284	up	3.347561	up
Os.25590.1.S1_at	1.2683965	up	1.438671	up	1.401225	up	1.824806	up
Os.52103.1.S1_at	-1.6833234	down	-1.03284	down	-1.9509	down	-1.7386	down
Os.11382.1.S1_at	-3.0231376	down	-1.08964	down	-1.84886	down	-3.29415	down
Os.12847.1.S1_at	1.5694762	up	1.02521	up	1.963992	up	1.609042	up
OsAffx.15312.1.S1_at	-1.8650846	down	1.392578	up	-2.80384	down	-1.3393	down
Os.7370.1.S1_at	1.3728709	up	-1.53024	down	3.080225	up	-1.11463	down
Os.19340.1.S1_at	-2.166014	down	-1.14835	down	-1.75279	down	-2.48733	down
Os.38804.1.S1_at	2.2519934	up	1.167958	up	1.723293	up	2.630235	up
Os.49563.2.S1_x_at	-1.2737103	down	-1.2903	down	-1.5597	down	-1.64347	down
Os.52468.1.S1_at	-3.073636	down	-1.26132	down	-1.5955	down	-3.87684	down
Os.49874.2.S1_x_at	1.8060632	up	2.793835	up	-1.38847	down	5.045843	up
Os.4867.1.S1_at	1.4495524	up	-3.35908	down	6.758835	up	-2.31732	down
Os.27387.1.S1_x_at	1.8807344	up	-1.06939	down	2.151117	up	1.758702	up
Os.12092.1.S1_at	1.0172175	up	-1.16398	down	2.340989	up	-1.14427	down
Os.9929.1.S1_at	-1.2734526	down	1.441297	up	-2.89855	down	1.131803	up
Os.4377.1.S1_at	1.2024678	up	4.355243	up	-2.16571	down	5.237039	up
OsAffx.24464.1.S1_at	-1.6724807	down	-1.54091	down	-1.30454	down	-2.57714	down
Os.21056.1.S1_at	2.1599874	up	1.154535	up	1.740521	up	2.493781	up
Os.27814.1.S1_at	-2.6151297	down	1.007141	up	-2.0238	down	-2.59659	down
Os.53794.1.S1_at	2.2668633	up	1.527339	up	1.315618	up	3.46227	up
Os.14076.1.S1_s_at	-2.0946782	down	-1.54618	down	-1.29957	down	-3.23874	down
Os.7457.1.S1_a_at	-1.156104	down	-2.30661	down	1.148021	up	-2.66668	down
Os.7475.1.S1_at	1.9402992	up	1.542864	up	1.30199	up	2.993618	up
Os.8139.1.S1_at	-1.0372269	down	2.838526	up	-1.41362	down	2.736649	up

OsAffx.25688.1.S1_at	-1.7275041	down	1.000857	up	-2.00968	down	-1.72602	down
Os.19026.1.S1_at	-1.6095825	down	-1.2499	down	-1.6062	down	-2.01182	down
Os.18072.1.S1_x_at	-2.8219364	down	1.106205	up	-2.22026	down	-2.55101	down
Os.11748.2.S2_a_at	-1.355235	down	-1.4903	down	-1.34661	down	-2.0197	down
Os.53351.1.S1_x_at	1.7799246	up	1.399671	up	1.433761	up	2.491309	up
Os.6426.1.S1_at	-1.8723391	down	-1.28706	down	-1.55916	down	-2.40981	down
Os.20775.1.S1_at	-1.0569006	down	-1.70525	down	-1.17662	down	-1.80228	down
Os.54687.1.S1_at	-2.4086072	down	-1.21155	down	-1.65603	down	-2.91816	down
Os.12255.1.S1_a_at	-2.4744694	down	-1.10342	down	-1.81822	down	-2.73037	down
Os.48079.1.S1_at	-1.782388	down	1.140672	up	-2.28756	down	-1.56258	down
OsAffx.28670.1.S1_s_	-1.5718633	down	-1.34323	down	-1.49292	down	-2.11137	down
Os.11771.1.S1_at	-2.070599	down	3.354945	up	-6.72705	down	1.620277	up
Os.9010.3.S1_x_at	-1.622738	down	-1.29871	down	-1.54382	down	-2.10747	down
Os.54917.1.S1_at	-1.3254449	down	-1.66421	down	-1.20471	down	-2.20582	down
Os.33759.1.S1_at	-1.7489144	down	-1.1609	down	-1.72697	down	-2.03031	down
OsAffx.12526.1.S1_at	1.6695474	up	1.209141	up	1.657925	up	2.018718	up
Os.47974.1.A1_at	-2.7362616	down	1.003007	up	-2.01068	down	-2.72806	down
Os.49795.1.S1_at	2.1615448	up	1.602531	up	1.250862	up	3.463944	up
Os.21394.1.S1_at	1.1845481	up	2.208488	up	-1.10186	down	2.61606	up
Os.9547.1.S1_at	2.2970228	up	1.036361	up	1.933349	up	2.380545	up
OsAffx.26788.1.S1_at	1.7264549	up	-1.69347	down	-1.18316	down	1.01948	up
OsAffx.17396.1.S1_at	-1.2722867	down	-1.56683	down	-1.27871	down	-1.99346	down
Os.8052.1.S1_at	1.5014359	up	1.491141	up	1.343387	up	2.238852	up
Os.17528.2.A1_a_at	1.4454795	up	1.625948	up	1.231871	up	2.350274	up
Os.7298.3.S1_x_at	-2.075275	down	-1.39942	down	-1.43099	down	-2.90418	down
OsAffx.12732.1.S1_s_	2.613475	up	1.316497	up	1.520892	up	3.440632	up
Os.4807.1.S1_at	1.5733944	up	1.877005	up	1.066471	up	2.953269	up
Os.27219.1.S1_at	-1.6307559	down	-1.91075	down	-1.04757	down	-3.11596	down
Os.9820.1.S1_at	2.7571204	up	-47.0195	down	94.10992	up	-17.0539	down
Os.18226.1.S1_a_at	2.6528227	up	1.140986	up	1.753427	up	3.026835	up
Os.53193.1.S1_at	-2.7764735	down	-1.16559	down	-1.71569	down	-3.23624	down
Os.26698.4.S1_x_at	-1.4348063	down	-1.55233	down	-1.28816	down	-2.22729	down
OsAffx.27093.1.S1_at	-3.4679785	down	2.339746	up	-4.67514	down	-1.4822	down
Os.18490.1.S1_x_at	-3.1652484	down	2.97635	up	-5.94518	down	-1.06347	down
OsAffx.3097.1.S1_x_a	-1.7484863	down	-1.21306	down	-1.6465	down	-2.12102	down
Os.32153.2.S1_x_at	3.7391236	up	-1.04945	down	2.095886	up	3.56292	up
Os.16899.1.S1_at	-1.8834394	down	-1.86556	down	-1.0704	down	-3.51367	down
Os.17033.1.S1_at	1.5925251	up	1.291823	up	1.545728	up	2.05726	up
Os.27939.1.A1_at	-1.326668	down	-1.64934	down	-1.21062	down	-2.18812	down
Os.46743.3.S1_a_at	2.6141503	up	1.362573	up	1.465247	up	3.561971	up
Os.11835.3.S1_x_at	-3.0435586	down	1.451544	up	-2.89719	down	-2.09677	down
OsAffx.25580.1.S1_x_	-2.8573787	down	1.212139	up	-2.41928	down	-2.3573	down
OsAffx.6397.1.S1_at	3.029434	up	-3.30236	down	6.589786	up	-1.09009	down
OsAffx.13763.1.S1_at	1.237619	up	1.836801	up	1.086267	up	2.273259	up
Os.7345.1.S1_x_at	-2.2924623	down	-1.37316	down	-1.45279	down	-3.14793	down
AFFX-ThrX-5_at	-2.0910091	down	1.323465	up	-2.64004	down	-1.57995	down
Os.6876.1.S1_at	2.0210357	up	1.74794	up	1.1408	up	3.53265	up

Os.12206.1.S1_at	-2.2778513	down	-1.58403	down	-1.25882	down	-3.60819	down
Os.17730.1.S1_x_at	1.9527671	up	1.533801	up	1.299983	up	2.995155	up
Os.6405.1.S1_at	1.8818915	up	1.291266	up	1.544064	up	2.430023	up
Os.26668.1.S1_at	-1.8969287	down	1.207016	up	-2.40615	down	-1.57158	down
Os.37533.1.S1_at	1.9668479	up	-1.04274	down	2.078465	up	1.886233	up
Os.11611.1.S1_at	-3.8903131	down	1.515835	up	-3.02041	down	-2.56645	down
Os.56019.1.S1_at	3.7293794	up	-1.40746	down	2.804245	up	2.649727	up
Os.27824.1.S1_at	1.2035252	up	1.833241	up	1.086823	up	2.206351	up
Os.23086.2.S1_at	1.2105006	up	-1.1705	down	2.331386	up	1.034174	up
Os.8031.1.S1_at	-1.8599709	down	1.821231	up	-3.62684	down	-1.02127	down
Os.23847.1.S1_at	-2.3808775	down	-1.18787	down	-1.67644	down	-2.82816	down
Os.9330.2.S1_a_at	1.1963388	up	1.964562	up	1.013246	up	2.350282	up
Os.8022.1.S1_at	4.72835	up	1.063881	up	1.87094	up	5.0304	up
Os.26440.1.S1_at	1.3854235	up	2.582796	up	-1.29781	down	3.578266	up
OsAffx.32277.1.S1_x_	-1.2163885	down	1.173021	up	-2.33417	down	-1.03697	down
Os.57179.1.S1_at	-2.3279164	down	1.126691	up	-2.24147	down	-2.06615	down
AFFX-ThrX-M_at	-1.6486883	down	1.056417	up	-2.10165	down	-1.56064	down
Os.16635.1.S1_at	-2.9692347	down	-1.32466	down	-1.50175	down	-3.93324	down
Os.57316.1.S1_at	-2.4916418	down	2.63041	up	-5.23238	down	1.055693	up
OsAffx.16693.1.S1_x_	-2.3570466	down	1.335053	up	-2.6552	down	-1.76551	down
Os.52539.1.S1_at	-2.5228631	down	-1.21588	down	-1.63561	down	-3.0675	down
Os.25613.1.A1_s_at	1.2852701	up	-2.65884	down	5.286315	up	-2.0687	down
Os.20371.1.A1_at	-1.1268382	down	-2.0609	down	1.036645	up	-2.3223	down
Os.5404.1.S1_a_at	-1.9041704	down	-1.3978	down	-1.42205	down	-2.66166	down
Os.12539.1.S1_at	1.5734336	up	1.60684	up	1.236969	up	2.528256	up
Os.27156.1.S1_at	-3.4825091	down	1.499664	up	-2.9803	down	-2.32219	down
Os.37394.1.S1_at	-1.6027852	down	-1.40779	down	-1.41164	down	-2.25639	down
Os.21335.1.A1_at	-2.1792915	down	-1.23573	down	-1.60793	down	-2.69303	down
Os.8395.1.S1_at	-1.320974	down	-1.75017	down	-1.13518	down	-2.31193	down
Os.24799.1.S1_at	-1.634753	down	-1.41933	down	-1.39928	down	-2.32026	down
Os.15259.1.S2_at	2.088248	up	1.012273	up	1.961886	up	2.113877	up
OsAffx.14412.2.S1_s_	-2.6273298	down	1.188842	up	-2.36081	down	-2.20999	down
Os.55731.1.S1_at	-1.9511535	down	-1.26591	down	-1.56867	down	-2.46999	down
Os.52165.1.S1_at	-2.5714931	down	-1.01575	down	-1.95467	down	-2.612	down
Os.26592.1.S1_s_at	1.0836389	up	1.675402	up	-3.32614	down	1.815531	up
Os.12323.1.S1_at	1.3977951	up	2.382122	up	-1.19996	down	3.329718	up
Os.11628.1.S1_at	-2.6461532	down	-1.42151	down	-1.39652	down	-3.76152	down
Os.49611.1.S1_at	-1.4977936	down	-3.99709	down	2.01353	up	-5.98682	down
OsAffx.24382.1.S1_x_	-1.6001921	down	-1.28045	down	-1.5503	down	-2.04896	down
Os.9760.1.S1_at	-1.0913152	down	1.262582	up	-2.50571	down	1.156936	up
Os.52219.1.A1_at	-1.5649449	down	-1.81042	down	-1.0962	down	-2.8332	down
Os.11525.1.S1_s_at	-2.1453335	down	-1.21247	down	-1.63661	down	-2.60114	down
Os.25007.3.S1_x_at	-1.4553453	down	-1.48569	down	-1.33542	down	-2.1622	down
OsAffx.12494.1.S1_at	-2.1304212	down	1.044626	up	-2.07255	down	-2.03941	down
Os.27569.3.S1_at	-2.887481	down	-1.46625	down	-1.3528	down	-4.23378	down
OsAffx.27615.2.S1_at	1.9492114	up	-1.45232	down	2.88039	up	1.342138	up
Os.51738.1.S1_at	-3.1989448	down	1.073849	up	-2.12937	down	-2.97895	down

Os.7206.1.S1_at	1.4420584	up	2.11538	up	-1.06689	down	3.050501	up
Os.15790.1.S1_at	-2.6923087	down	-1.14255	down	-1.73479	down	-3.0761	down
OsAffx.19112.1.S1_at	-1.8686966	down	-1.12956	down	-1.75456	down	-2.1108	down
Os.4746.2.A1_at	-1.2624407	down	-1.73631	down	-1.14062	down	-2.19198	down
Os.15602.1.S1_at	-2.2630172	down	-1.21753	down	-1.62654	down	-2.75528	down
Os.37006.1.S1_at	1.136658	up	2.24578	up	-1.1342	down	2.552684	up
Os.5373.1.S1_at	4.314814	up	1.370628	up	1.443678	up	5.914007	up
Os.10360.1.S1_at	-2.2870126	down	1.068506	up	-2.11372	down	-2.14038	down
Os.6265.1.S1_at	-2.4663274	down	1.108282	up	-2.19225	down	-2.22536	down
Os.11924.1.S1_at	-3.17616	down	-1.01343	down	-1.95152	down	-3.21883	down
OsAffx.14311.1.S1_s_	-1.547657	down	1.063015	up	-2.10196	down	-1.45591	down
Os.29966.2.S1_x_at	-1.4776664	down	1.146972	up	-2.26772	down	-1.28832	down
Os.9885.1.S1_x_at	1.2364366	up	1.801592	up	1.097348	up	2.227555	up
Os.27112.1.S1_at	1.9624548	up	1.759271	up	1.123653	up	3.452489	up
Os.12743.1.S1_at	1.6663713	up	1.443603	up	1.369302	up	2.405579	up
Os.55299.1.S1_at	3.2581632	up	-1.06484	down	2.104356	up	3.05976	up
Os.11705.1.S1_at	2.328616	up	-1.46535	down	2.89566	up	1.589117	up
Os.38192.1.S1_at	-2.3716955	down	-1.01964	down	-1.93789	down	-2.41827	down
Os.9878.1.S1_at	-1.0240369	down	2.354844	up	-1.19191	down	2.299569	up
Os.7624.1.S1_at	1.4413583	up	1.81785	up	1.08652	up	2.620173	up
Os.5422.1.S1_s_at	-1.4948409	down	-1.84981	down	-1.06767	down	-2.76517	down
Os.4292.2.S1_at	-1.9997889	down	-1.02023	down	-1.93511	down	-2.04025	down
Os.47381.1.S1_x_at	1.0156579	up	2.811049	up	-1.42419	down	2.855064	up
Os.4530.1.S1_at	4.547062	up	-2.3242	down	4.5872	up	1.956403	up
OsAffx.18719.1.S1_at	-1.2259773	down	-1.94539	down	-1.01426	down	-2.38501	down
Os.54636.1.S1_at	-2.8819137	down	-1.11399	down	-1.77002	down	-3.21041	down
OsAffx.23573.1.S1_s_	-1.6315151	down	-1.64861	down	-1.19558	down	-2.68974	down
Os.52518.1.S1_at	1.8141274	up	-1.01971	down	2.009167	up	1.779054	up
Os.13876.1.S1_a_at	-2.5885336	down	-1.34514	down	-1.46465	down	-3.48194	down
Os.28351.1.A1_a_at	-2.5093918	down	-1.11437	down	-1.76787	down	-2.79638	down
Os.12728.1.S1_at	1.6696041	up	1.750829	up	1.125188	up	2.923192	up
Os.52335.1.A1_at	-1.981832	down	1.134955	up	-2.23578	down	-1.74618	down
Os.7607.1.S1_at	-1.3980546	down	1.260903	up	1.56224	up	-1.10877	down
Os.9247.1.S1_at	-2.1834939	down	1.075746	up	-2.11889	down	-2.02975	down
Os.20079.1.S1_s_at	-1.753344	down	-1.22455	down	-1.60843	down	-2.14706	down
Os.15307.1.S1_at	-3.851084	down	1.112538	up	-2.191	down	-3.46153	down
Os.27946.1.S1_at	2.3646204	up	1.405463	up	1.401003	up	3.323386	up
Os.19183.1.S1_at	-2.0178475	down	1.033313	up	-2.03461	down	-1.95279	down
Os.10320.1.S1_x_at	-2.00117	down	1.059493	up	-2.08556	down	-1.8888	down
Os.33722.1.S1_at	3.7006562	up	1.004874	up	1.958067	up	3.718693	up
Os.54966.1.S1_at	2.6932657	up	1.959405	up	1.004091	up	5.2772	up
Os.38200.1.S1_at	-1.830439	down	-1.12783	down	-1.74438	down	-2.06443	down
Os.8352.1.S1_at	1.2001815	up	-2.79248	down	1.419528	up	-2.32672	down
Os.13562.1.S1_at	-2.1380038	down	1.129994	up	-2.22286	down	-1.89205	down
Os.49779.1.S1_at	-1.6835666	down	-1.23115	down	-1.59716	down	-2.07272	down
Os.27235.1.S1_at	-3.608403	down	-1.11086	down	-1.76994	down	-4.00844	down
Os.19321.1.S1_at	-1.1850002	down	2.539189	up	-1.29189	down	2.142775	up

Os.11271.2.S1_at	-2.8464978	down	-1.16159	down	-1.69174	down	-3.30646	down
Os.23468.1.A1_at	2.629216	up	-1.21718	down	2.391614	up	2.160082	up
OsAffx.14735.1.S1_at	-2.113387	down	1.199648	up	-2.35578	down	-1.76167	down
Os.32943.2.S1_x_at	1.0621654	up	-3.58932	down	7.047413	up	-3.37925	down
Os.23868.1.S1_at	-1.1332954	down	1.107381	up	1.773009	up	-1.0234	down
Os.57172.1.S1_at	4.5052443	up	-1.08244	down	2.125112	up	4.162137	up
Os.11923.1.S1_x_at	-1.2562208	down	1.88031	up	-3.69021	down	1.496799	up
Os.3582.1.S1_at	-2.264583	down	-1.3655	down	-1.43711	down	-3.09228	down
AFFX-r2-Bs-phe-M_at	-1.4241003	down	1.072518	up	-2.10459	down	-1.32781	down
Os.55352.1.S1_x_at	-1.929988	down	-1.10376	down	-1.7762	down	-2.13024	down
OsAffx.14466.1.S1_at	-1.8346108	down	-1.39658	down	-1.40372	down	-2.56218	down
Os.5593.1.S1_at	4.5478387	up	1.231002	up	1.59248	up	5.598398	up
Os.15708.1.S1_a_at	-2.0525656	down	-1.23682	down	-1.58498	down	-2.53865	down
Os.51373.1.S1_at	-1.7898197	down	-1.53302	down	-1.27869	down	-2.74384	down
OsAffx.28714.1.S1_x_	3.3273616	up	1.117313	up	1.75432	up	3.717703	up
Os.7097.2.S1_a_at	1.1397712	up	3.141887	up	-1.60346	down	3.581032	up
Os.23984.1.S1_at	-1.3549361	down	-1.52557	down	-1.28429	down	-2.06705	down
OsAffx.26691.1.S1_at	-1.7329063	down	-1.55012	down	-1.26382	down	-2.68622	down
OsAffx.3013.1.S1_at	1.2325399	up	1.821499	up	1.075384	up	2.24507	up
Os.26478.1.S1_at	-2.126586	down	-1.04709	down	-1.87071	down	-2.22672	down
Os.26766.1.S1_at	1.9875659	up	1.30296	up	1.503325	up	2.589719	up
Os.23677.1.S1_at	-1.9925606	down	1.02282	up	-2.00284	down	-1.9481	down
Os.50382.1.S1_at	-1.1555411	down	-2.04988	down	1.047033	up	-2.36872	down
Os.4633.1.S1_at	1.2492245	up	-1.28285	down	2.511506	up	-1.02691	down
Os.12778.1.S2_at	-1.6263957	down	1.07586	up	-2.10574	down	-1.51172	down
Os.52358.1.S1_at	-2.5530114	down	-1.11975	down	-1.74791	down	-2.85874	down
Os.22485.1.A1_at	-2.840924	down	-1.43408	down	-1.36472	down	-4.07411	down
OsAffx.15673.1.S1_x_	-1.7115755	down	-1.17638	down	-1.66347	down	-2.01346	down
Os.28823.1.S1_at	2.0961196	up	-1.33198	down	2.606454	up	1.57369	up
Os.53887.2.S1_x_at	2.031957	up	1.661523	up	1.177721	up	3.376143	up
Os.25849.3.S1_x_at	-2.0833685	down	-1.45417	down	-1.34527	down	-3.02958	down
Os.10824.1.S1_at	-1.74563	down	-1.48143	down	-1.32051	down	-2.58603	down
Os.23125.1.S1_at	1.1814327	up	1.979998	up	-1.01258	down	2.339235	up
Os.23397.1.S2_at	3.2351866	up	-1.22655	down	2.398278	up	2.637625	up
Os.5615.1.S1_at	1.4571332	up	1.646044	up	1.187832	up	2.398506	up
Os.5431.1.S1_at	2.1360464	up	1.84171	up	1.061552	up	3.933978	up
Os.5047.1.S1_at	2.5396628	up	1.054811	up	1.853467	up	2.678865	up
OsAffx.10962.1.S1_x_	-1.9444968	down	1.027568	up	-2.0089	down	-1.89233	down
Os.53666.1.S1_x_at	-2.86406	down	1.322289	up	-2.58501	down	-2.16599	down
Os.33309.1.S1_at	4.114329	up	-1.224	down	2.392825	up	3.361379	up
OsAffx.10882.2.S1_x_	-1.9004731	down	1.067065	up	-2.08602	down	-1.78103	down
Os.3206.1.S1_at	1.7437193	up	3.319266	up	-1.69806	down	5.787869	up
Os.47617.1.S1_at	3.658121	up	-1.1303	down	2.208522	up	3.236423	up
Os.52583.1.S1_at	-2.0068562	down	-1.25855	down	-1.55222	down	-2.52572	down
Os.16997.1.S1_at	1.3609203	up	-1.48021	down	2.891162	up	-1.08766	down
Os.17560.1.A1_at	-1.7602717	down	1.034151	up	-2.01984	down	-1.70214	down
OsAffx.27550.1.S1_at	1.1542108	up	2.590955	up	-1.32659	down	2.990508	up

Os.32953.1.S1_at	2.3635824	up	1.190011	up	1.641017	up	2.81269	up
Os.27810.1.S1_at	1.341115	up	-1.27421	down	2.487083	up	1.052503	up
Os.17130.1.S1_at	2.508591	up	1.618408	up	1.205843	up	4.059925	up
Os.9166.4.S1_x_at	-2.069816	down	-1.21156	down	-1.6106	down	-2.5077	down
Os.9991.1.S1_at	-1.652903	down	-1.53027	down	-1.27514	down	-2.52939	down
Os.46627.1.S1_at	1.2450879	up	2.798949	up	-1.43462	down	3.484937	up
OsAffx.4996.1.S1_at	1.3764771	up	2.132802	up	-1.0935	down	2.935753	up
Os.5288.1.S1_a_at	2.0103354	up	1.737708	up	1.122366	up	3.493377	up
Os.10823.2.S1_at	1.6502274	up	2.221983	up	-1.13939	down	3.666778	up
Os.28011.1.S1_at	1.9169225	up	-1.31552	down	-1.48223	down	1.457158	up
Os.6014.2.S1_at	1.598876	up	1.422068	up	1.370607	up	2.27371	up
Os.51540.1.S1_at	2.7760038	up	-1.36797	down	2.666138	up	2.029284	up
Os.2867.1.A1_at	4.1372447	up	-1.30197	down	2.537236	up	3.17767	up
Os.11612.1.S1_at	-3.4932692	down	1.294749	up	-2.52288	down	-2.69803	down
Os.7707.1.S1_at	-2.5110786	down	1.006326	up	-1.96076	down	-2.49529	down
Os.46046.1.A1_at	1.1722432	up	-1.49697	down	2.915572	up	-1.27702	down
Os.54853.1.S1_at	1.5184382	up	1.468694	up	1.324825	up	2.230122	up
Os.15867.1.A1_at	1.1401985	up	-1.3492	down	2.624527	up	-1.18331	down
Os.9566.1.S1_x_at	-3.4246602	down	1.407931	up	-2.73819	down	-2.43241	down
Os.53037.1.S1_at	-2.8990579	down	1.075927	up	-2.09245	down	-2.69447	down
Os.53801.1.S1_at	2.4913347	up	-1.52679	down	2.968593	up	1.631742	up
Os.20717.1.S1_at	2.060925	up	1.984353	up	-1.02065	down	4.089603	up
Os.8059.1.S1_at	-2.1294076	down	-1.12076	down	-1.73445	down	-2.38655	down
OsAffx.30773.1.S1_x_	-1.5347621	down	-1.42831	down	-1.36095	down	-2.19211	down
Os.24385.2.S1_at	-2.2028508	down	-1.17936	down	-1.64813	down	-2.59796	down
Os.13960.1.S1_a_at	-3.2989404	down	-1.08371	down	-1.79323	down	-3.5751	down
Os.17433.1.S1_at	1.2413353	up	-1.19124	down	2.31433	up	1.042055	up
Os.6851.2.S1_x_at	-1.1303401	down	-2.12169	down	1.092121	up	-2.39823	down
Os.10894.1.S1_s_at	1.9846972	up	1.125715	up	1.725646	up	2.234204	up
Os.5823.1.S1_at	3.478065	up	-1.2509	down	2.429892	up	2.780444	up
Os.9118.1.S1_at	-1.1270102	down	1.460493	up	-2.83688	down	1.2959	up
Os.39552.1.A1_at	1.2663244	up	-1.65078	down	3.206264	up	-1.3036	down
Os.5910.1.S1_at	2.0894969	up	1.371942	up	1.415654	up	2.866669	up
Os.14332.1.S1_at	-3.3676746	down	1.172671	up	-2.27755	down	-2.8718	down
Os.18087.1.S1_at	3.400622	up	-1.3185	down	2.559374	up	2.579154	up
Os.24495.1.S1_at	1.0356088	up	2.705227	up	-1.39387	down	2.801557	up
Os.17232.1.S1_at	1.7014312	up	2.918205	up	-1.50366	down	4.965125	up
Os.52727.1.S1_at	-2.726426	down	-1.26994	down	-1.52814	down	-3.46239	down
Os.7203.1.S1_at	1.8205305	up	1.442835	up	1.344609	up	2.626724	up
OsAffx.15484.1.S1_at	-2.8481843	down	1.424443	up	-2.76308	down	-1.99951	down
Os.24715.2.A1_a_at	-1.4625157	down	-1.60902	down	-1.20532	down	-2.35322	down
Os.21893.3.A1_at	-1.668938	down	-1.20748	down	-1.60539	down	-2.01522	down
Os.12163.1.S1_at	-1.2662795	down	-6.02161	down	3.107134	up	-7.62505	down
Os.2365.1.S1_at	2.163038	up	1.151838	up	1.682385	up	2.491469	up
Os.23964.1.S1_at	-11.1052265	down	1.906941	up	-3.6945	down	-5.82358	down
Os.2517.1.S1_a_at	1.998545	up	1.090338	up	1.776812	up	2.179089	up
Os.11912.1.S1_at	1.8906513	up	1.249649	up	1.550108	up	2.362651	up

Os.6090.1.S1_a_at	-2.2902093	down	-1.27027	down	-1.52478	down	-2.90919	down
OsAffx.24678.1.S1_at	2.1894562	up	-1.06869	down	2.069426	up	2.048734	up
Os.10735.1.S1_at	2.3172746	up	1.2175	up	1.590406	up	2.821281	up
OsAffx.27460.1.S1_at	-2.606065	down	1.11175	up	-2.15198	down	-2.34411	down
Os.57549.1.S1_s_at	-1.3500034	down	-1.72932	down	-1.11911	down	-2.33458	down
Os.9292.1.S1_at	-2.0984576	down	-1.01139	down	-1.9133	down	-2.12235	down
OsAffx.26224.1.S1_s_	1.333476	up	2.504899	up	-1.29461	down	3.340222	up
Os.50809.1.S1_at	-2.1688812	down	-1.42122	down	-1.3611	down	-3.08245	down
Os.52552.1.S1_at	-1.7481967	down	-1.84079	down	-1.05007	down	-3.21806	down
Os.11760.1.S1_at	2.101163	up	1.159142	up	1.667407	up	2.435546	up
OsAffx.15538.1.S1_at	-2.9948711	down	2.208952	up	-4.2678	down	-1.35579	down
Os.2694.1.S1_at	6.49781	up	-4.5994	down	8.885311	up	1.412751	up
Os.49078.1.S1_at	-1.9325329	down	-1.11348	down	-1.73464	down	-2.15184	down
Os.49889.1.S1_at	-3.1058674	down	1.16005	up	-2.24061	down	-2.67736	down
Os.17941.1.S1_s_at	1.6391367	up	1.647275	up	1.172496	up	2.700109	up
Os.9885.2.S1_x_at	1.3187827	up	1.689241	up	1.143275	up	2.227742	up
Os.26744.1.A1_at	1.5302584	up	-2.81679	down	1.458582	up	-1.84073	down
Os.5947.1.S1_at	-2.2325673	down	-1.05016	down	-1.83865	down	-2.34456	down
Os.17190.1.A1_x_at	-3.1096406	down	1.210109	up	-2.33583	down	-2.56972	down
Os.38982.1.S1_at	2.5878718	up	-1.52681	down	2.946126	up	1.694952	up
Os.22932.1.S1_at	-2.8046498	down	1.417839	up	-2.73579	down	-1.97812	down
Os.10120.2.S1_at	-3.3666472	down	1.519569	up	-2.93198	down	-2.21553	down
Os.47922.1.S1_x_at	1.9390273	up	-1.33587	down	2.576937	up	1.451505	up
Os.11429.1.S1_at	-1.9103124	down	-1.4036	down	-1.37416	down	-2.68132	down
OsAffx.32234.1.A1_x_	1.2406572	up	-2.04683	down	1.061384	up	-1.64979	down
Os.46452.1.S1_at	-2.3998873	down	-1.15389	down	-1.67096	down	-2.7692	down
OsAffx.23976.1.S1_at	-2.4844549	down	-1.57087	down	-1.22732	down	-3.90277	down
Os.6325.2.A1_x_at	-1.373647	down	-1.66353	down	-1.15895	down	-2.28511	down
Os.34789.1.S1_at	-1.2653869	down	-1.72789	down	-1.11568	down	-2.18645	down
OsAffx.6475.1.S1_at	-2.3087225	down	-1.65749	down	-1.16296	down	-3.82668	down
Os.24008.1.S2_at	-1.3915107	down	1.244749	up	-2.39935	down	-1.1179	down
OsAffx.11619.1.S1_at	3.101063	up	-1.2299	down	2.369921	up	2.521395	up
Os.49651.1.S1_s_at	1.5526468	up	-1.23713	down	2.383292	up	1.255042	up
Os.9485.1.S1_at	-3.4446533	down	-1.21079	down	-1.59096	down	-4.17076	down
Os.10316.1.S1_at	-1.4277261	down	3.344247	up	-1.73621	down	2.342359	up
OsAffx.27867.1.S1_s_	1.269105	up	-2.1956	down	1.140076	up	-1.73004	down
Os.7219.1.S1_at	-2.9583747	down	-1.19962	down	-1.60505	down	-3.54893	down
Os.10585.1.A1_at	-2.2383056	down	-1.17218	down	-1.6424	down	-2.6237	down
Os.11300.2.S1_at	-5.768339	down	2.489422	up	-4.79224	down	-2.31714	down
Os.49198.1.S1_at	1.2235124	up	-2.81762	down	1.463745	up	-2.3029	down
Os.47722.1.S1_at	-1.7244865	down	-1.36921	down	-1.40586	down	-2.36119	down
Os.55638.1.S1_at	-3.2492115	down	1.585981	up	-3.05281	down	-2.04871	down
Os.49432.1.S1_at	-2.2281141	down	-1.00197	down	-1.92103	down	-2.2325	down
OsAffx.9584.1.S1_at	3.5237043	up	2.516474	up	-1.30739	down	8.867311	up
Os.9978.1.S1_at	-1.1690055	down	-1.64557	down	3.167373	up	-1.92368	down
Os.5360.2.S1_at	2.1888592	up	1.276157	up	1.507919	up	2.793328	up
Os.26813.3.S1_x_at	-1.8201524	down	1.067217	up	-2.05336	down	-1.70551	down

Os.38196.1.A1_at	-1.59433	down	1.36033	up	-2.61655	down	-1.17202	down
Os.21255.1.S1_x_at	-2.422712	down	1.158576	up	-2.22837	down	-2.09111	down
Os.12963.1.S1_at	-3.277436	down	1.143491	up	-2.19933	down	-2.86617	down
Os.7064.1.S1_s_at	1.0921608	up	2.820921	up	-1.46697	down	3.0809	up
OsAffx.7470.1.S1_x_a	-1.9090848	down	-1.42565	down	-1.34873	down	-2.72168	down
Os.18776.1.S1_at	-3.4912596	down	1.073695	up	-2.06451	down	-3.25163	down
Os.27069.1.A1_at	-1.7315782	down	-1.32753	down	-1.4483	down	-2.29872	down
Os.25441.1.S1_a_at	1.6720163	up	-1.30175	down	2.501762	up	1.284438	up
Os.8872.1.S1_at	-1.9759078	down	-1.2279	down	-1.56512	down	-2.42621	down
OsAffx.14942.2.S1_at	-1.0976124	down	1.115673	up	-2.14385	down	1.016454	up
OsAffx.15994.1.S1_x_	-2.3207536	down	-1.06718	down	-1.80061	down	-2.47666	down
Os.33154.2.S1_at	-1.8012977	down	-1.24601	down	-1.54139	down	-2.24444	down
Os.4856.1.S1_at	2.005463	up	1.294503	up	1.483567	up	2.596077	up
Os.9805.1.S1_at	1.5101376	up	-8.76883	down	16.83982	up	-5.80664	down
OsAffx.5024.1.S1_at	5.5841403	up	-1.92959	down	3.705618	up	2.893946	up
Os.54206.1.A1_x_at	-1.7553395	down	-1.29604	down	-1.48144	down	-2.27499	down
OsAffx.32346.1.A1_at	1.1203775	up	-2.34275	down	1.220242	up	-2.09104	down
Os.28457.1.S1_at	-2.038749	down	-1.32418	down	-1.4497	down	-2.69967	down
Os.52314.1.S2_x_at	-2.4042547	down	-1.06703	down	-1.79889	down	-2.56541	down
Os.40388.2.S1_x_at	1.931009	up	1.041562	up	1.842814	up	2.011265	up
Os.14368.1.S1_at	1.1850563	up	2.326008	up	-1.21192	down	2.75645	up
Os.33834.1.S1_x_at	-2.4915323	down	1.109697	up	-2.12979	down	-2.24524	down
Os.8439.1.S1_at	-1.752555	down	-1.59269	down	-1.20492	down	-2.79127	down
Os.33170.1.S1_x_at	1.5245008	up	2.136938	up	-1.11397	down	3.257764	up
OsAffx.31140.1.S1_at	2.6802826	up	-10.5631	down	20.26267	up	-3.94105	down
Os.16193.1.S1_at	1.8463058	up	-1.61875	down	3.104271	up	1.140577	up
Os.57548.1.S1_at	-2.4103308	down	-1.80261	down	-1.06366	down	-4.34488	down
Os.10765.1.S1_at	-4.1221867	down	2.543847	up	-4.87742	down	-1.62045	down
Os.46554.1.S1_at	-2.5265737	down	1.121455	up	-2.14984	down	-2.25294	down
Os.12575.2.S1_at	-2.0496886	down	-1.35087	down	-1.41821	down	-2.76886	down
Os.5693.1.S1_at	2.256223	up	-1.07836	down	2.065809	up	2.092281	up
Os.2289.1.S1_at	8.559051	up	2.18673	up	-1.1415	down	18.71633	up
Os.16910.1.S1_at	-2.6087587	down	1.1324	up	-2.16928	down	-2.30374	down
Os.11307.1.S1_at	-1.7139821	down	-1.24368	down	-1.54026	down	-2.13164	down
Os.9392.1.S1_at	-3.2642572	down	1.252118	up	-2.39852	down	-2.60699	down
Os.38367.1.S1_s_at	-2.2756073	down	1.154585	up	-2.21152	down	-1.97093	down
Os.46746.1.S1_at	-3.1134827	down	-1.11181	down	-1.72275	down	-3.4616	down
OsAffx.5214.1.S1_x_a	-1.6336464	down	-1.27055	down	-1.5075	down	-2.07563	down
Os.7745.1.S1_at	2.0849724	up	1.281219	up	1.494597	up	2.671306	up
OsAffx.11145.1.S1_s_	-4.0480056	down	1.50148	up	-2.8751	down	-2.69601	down
OsAffx.12949.1.S1_at	-2.7589936	down	1.390381	up	-2.66136	down	-1.98434	down
OsAffx.13491.1.S1_at	-3.2583907	down	1.68483	up	-3.22456	down	-1.93396	down
Os.53795.1.S1_at	2.3859713	up	2.191158	up	-1.14498	down	5.22804	up
Os.48879.1.S1_at	-1.5207618	down	1.080715	up	-2.06816	down	-1.40718	down
Os.5641.1.S1_a_at	-2.7105992	down	1.070199	up	-2.04793	down	-2.5328	down
Os.33166.1.S1_at	2.1046603	up	1.610857	up	1.187864	up	3.390307	up
Os.47508.1.A1_s_at	-2.6348934	down	1.098615	up	-2.10216	down	-2.39838	down

Os.4023.1.S1_at	-2.0181835	down	1.068745	up	-2.04333	down	-1.88837	down
OsAffx.2617.1.S1_at	-1.9713902	down	-1.12632	down	-1.69721	down	-2.22042	down
OsAffx.23819.2.S1_x_	-1.876221	down	-2.99892	down	1.568839	up	-5.62664	down
Os.52093.1.S1_at	1.4338098	up	-1.38937	down	2.655394	up	1.031989	up
OsAffx.22181.1.S1_s_	1.2472552	up	1.853148	up	1.031331	up	2.311349	up
Os.22918.1.S1_a_at	1.6909915	up	-1.32472	down	2.52963	up	1.276493	up
Os.8820.1.S1_a_at	-1.513354	down	-1.43606	down	-1.32952	down	-2.17326	down
Os.48706.1.S1_at	-2.5257666	down	-1.1845	down	-1.61156	down	-2.99178	down
Os.9596.1.S1_at	-1.8791025	down	-1.20908	down	-1.57873	down	-2.27198	down
Os.17069.1.A1_at	-1.7417462	down	-1.23846	down	-1.54121	down	-2.15709	down
OsAffx.11879.1.S1_at	-2.5148435	down	1.080574	up	-2.06216	down	-2.32732	down
Os.37213.1.S1_at	-1.1813717	down	-1.96853	down	3.756657	up	-2.32556	down
Os.49135.1.S1_at	1.8811859	up	1.652681	up	1.154647	up	3.109	up
Os.14198.1.S1_at	-1.9579787	down	2.08026	up	-3.96923	down	1.062453	up
OsAffx.2904.1.S1_at	-1.8907886	down	-1.30919	down	-1.45723	down	-2.47541	down
OsAffx.25458.1.S1_at	-2.2249458	down	1.199438	up	-2.2879	down	-1.85499	down
Os.18247.1.S1_a_at	2.3391125	up	1.74013	up	1.0961	up	4.07036	up
Os.18241.1.S1_at	-2.1403453	down	-1.14649	down	-1.66335	down	-2.45388	down
Os.37066.1.S1_at	-2.649368	down	1.612897	up	-3.07567	down	-1.64261	down
Os.36283.1.S1_at	1.5254835	up	-7.03862	down	3.691442	up	-4.61403	down
Os.13645.1.S1_at	1.2087605	up	1.708675	up	1.1159	up	2.065378	up
Os.45998.1.S1_at	1.8303484	up	1.809441	up	1.05361	up	3.311907	up
Os.10123.2.S1_x_at	-1.7844967	down	1.080133	up	-2.05812	down	-1.65211	down
Os.26698.2.S1_at	1.6619434	up	-1.65444	down	3.151176	up	1.004536	up
Os.19036.1.S1_at	-2.2162411	down	1.291721	up	-2.45928	down	-1.71573	down
Os.12146.1.S1_at	-2.9792535	down	1.213378	up	-2.31009	down	-2.45534	down
Os.44475.1.S1_x_at	6.654109	up	1.724997	up	1.10353	up	11.47832	up
Os.50776.1.S1_at	-2.3143828	down	-1.14182	down	-1.66714	down	-2.6426	down
Os.57103.1.S1_at	3.8667634	up	7.951527	up	-4.17771	down	30.74668	up
Os.23224.1.S1_s_at	1.5883552	up	-1.75791	down	3.343874	up	-1.10675	down
OsAffx.3874.1.S1_s_a	-1.9537833	down	-1.58841	down	-1.19749	down	-3.1034	down
Os.55252.1.S1_at	2.5446918	up	-1.5483	down	2.944937	up	1.643541	up
OsAffx.15860.1.S1_at	-2.308886	down	1.077825	up	-2.04953	down	-2.14217	down
OsAffx.15952.1.S1_at	-2.1942446	down	-1.15557	down	-1.6453	down	-2.53561	down
Os.50939.1.S1_at	1.4202867	up	-1.09933	down	2.089935	up	1.291958	up
Os.46446.1.S1_at	-3.6065204	down	-1.26808	down	-1.4987	down	-4.57336	down
Os.15218.1.S1_at	-1.9689418	down	-1.17945	down	-1.6111	down	-2.32227	down
Os.12701.1.S2_at	-1.9787312	down	1.397313	up	-2.65518	down	-1.4161	down
Os.11025.1.S1_at	-1.8332938	down	-1.37923	down	-1.37672	down	-2.52854	down
Os.52309.1.S1_at	1.2145029	up	-1.15826	down	2.199173	up	1.04856	up
Os.31290.1.S1_at	-1.760249	down	1.402759	up	-2.66281	down	-1.25485	down
Os.11599.1.S1_at	-1.5547649	down	1.30007	up	-2.46758	down	-1.19591	down
Os.11222.2.S1_x_at	2.1878252	up	2.00477	up	-1.05662	down	4.386086	up
Os.50995.1.S1_at	1.1565224	up	1.771946	up	1.070654	up	2.049295	up
OsAffx.23395.1.S1_s_	-2.1528158	down	1.08299	up	-2.05453	down	-1.98784	down
Os.40015.1.S1_at	1.2291558	up	1.025208	up	-1.94389	down	1.26014	up
Os.32478.1.S1_at	2.5481656	up	1.561271	up	1.214338	up	3.978377	up

Os.46765.1.S1_x_at	-2.0676007	down	-1.1973	down	-1.58343	down	-2.47554	down
Os.11534.1.S1_at	8.079985	up	-5.93735	down	11.25619	up	1.360873	up
Os.11023.1.S1_a_at	2.9637299	up	3.204857	up	-1.69069	down	9.498331	up
Os.4636.1.S1_a_at	1.0103809	up	1.413333	up	-2.679	down	1.428005	up
Os.15409.1.S1_at	-2.129962	down	1.047524	up	-1.98551	down	-2.03333	down
Os.17232.1.S2_at	1.4360853	up	2.560929	up	-1.35113	down	3.677712	up
OsAffx.25696.1.S1_s_	1.2166406	up	2.118265	up	-1.11777	down	2.577167	up
Os.16604.1.S1_at	-2.2709084	down	-1.23142	down	-1.53851	down	-2.79643	down
Os.8575.1.S1_at	1.5256667	up	-1.18311	down	2.241388	up	1.289541	up
Os.14783.1.S1_at	1.2332692	up	1.831544	up	1.034246	up	2.258787	up
Os.51513.1.S1_at	1.1923833	up	2.018605	up	-1.0657	down	2.406951	up
Os.2218.1.S1_at	1.9237115	up	1.195222	up	1.584771	up	2.299262	up
Os.28031.1.S1_at	1.2440623	up	-2.09821	down	1.107849	up	-1.68658	down
Os.52536.1.S1_at	1.0327731	up	14.00305	up	-7.39466	down	14.46198	up
Os.17030.1.S1_at	-1.6029717	down	1.137193	up	-2.15327	down	-1.40959	down
Os.17941.2.S1_x_at	1.5663269	up	1.804787	up	1.049102	up	2.826887	up
Os.15766.1.S1_at	1.2354367	up	2.534367	up	-1.33857	down	3.131051	up
Os.5066.1.S1_at	4.1549344	up	-1.02116	down	1.933333	up	4.068842	up
Os.35921.1.S1_at	2.1823397	up	1.881404	up	1.006294	up	4.105863	up
Os.37849.1.A1_at	-2.713026	down	1.275763	up	-2.41526	down	-2.12659	down
Os.14857.1.S1_at	-1.9823238	down	-1.14173	down	-1.65789	down	-2.26327	down
Os.52310.1.S1_at	-1.8153452	down	1.149648	up	-2.17607	down	-1.57904	down
Os.46883.1.S1_at	-2.9229908	down	1.338709	up	-2.53362	down	-2.18344	down
Os.11460.1.S1_at	-1.4660385	down	-1.47201	down	-1.28529	down	-2.15803	down
Os.25599.1.A1_at	-1.9848647	down	1.307476	up	-2.47338	down	-1.51809	down
Os.53718.1.A1_at	-2.411824	down	1.093859	up	-2.06878	down	-2.20488	down
Os.16649.1.S1_at	-2.0150821	down	-1.39753	down	-1.35325	down	-2.81613	down
OsAffx.26509.1.S1_s_	-3.5556006	down	1.754503	up	-3.31804	down	-2.02656	down
Os.54420.1.S1_x_at	3.4511335	up	-1.31699	down	2.49055	up	2.620462	up
Os.27019.1.S1_at	-1.355889	down	-2.70612	down	1.431078	up	-3.66919	down
Os.7525.1.S1_at	-2.4059424	down	-1.19459	down	-1.58285	down	-2.87412	down
Os.50381.1.S1_at	-3.5856543	down	-1.83874	down	-1.02821	down	-6.5931	down
Os.7278.1.S1_x_at	-2.5696619	down	1.029017	up	-1.94537	down	-2.4972	down
Os.8202.1.S1_at	1.6185186	up	1.251034	up	1.510704	up	2.024822	up
Os.53177.1.S1_at	2.8257322	up	1.270842	up	1.486391	up	3.59106	up
Os.22915.1.S1_at	2.1131232	up	1.360284	up	1.388247	up	2.874448	up
Os.33106.1.S1_at	1.6560357	up	2.226501	up	-1.1792	down	3.687164	up
Os.15812.1.S1_at	2.1589751	up	1.377467	up	1.370663	up	2.973916	up
Os.7441.1.S1_at	5.708644	up	-1.10514	down	2.08576	up	5.165549	up
Os.12943.1.S1_at	1.050364	up	2.62642	up	-1.39169	down	2.758697	up
Os.21066.1.S1_at	1.4026252	up	2.522983	up	-1.33714	down	3.5388	up
Os.24061.1.A1_at	2.2268882	up	1.054565	up	1.789015	up	2.348398	up
Os.11736.1.S1_at	-3.3049712	down	1.203638	up	-2.27077	down	-2.74582	down
OsAffx.16648.1.S1_at	2.0695972	up	-1.00775	down	1.900312	up	2.05369	up
Os.26581.1.S1_at	-2.6276886	down	1.081758	up	-2.03983	down	-2.42909	down
Os.13008.1.S1_at	1.3428986	up	1.946978	up	-1.03255	down	2.614595	up
Os.15991.1.S1_at	1.245799	up	-1.21355	down	2.288086	up	1.026575	up

Os.9039.1.S1_at	-1.4424835	down	1.458584	up	-2.74988	down	1.011162	up
OsAffx.15413.1.A1_a	1.5991532	up	1.498394	up	1.258215	up	2.396161	up
OsAffx.7001.1.S1_at	1.6777468	up	1.426429	up	1.321509	up	2.393187	up
Os.24496.1.S1_at	1.9648513	up	-1.10258	down	2.078377	up	1.782052	up
OsAffx.7123.1.S1_at	-2.4503477	down	-1.22394	down	-1.54007	down	-2.99909	down
Os.46115.1.S1_at	2.084635	up	1.279077	up	1.473641	up	2.666409	up
Os.16523.1.S1_at	-2.9440222	down	1.319364	up	-2.48617	down	-2.23139	down
Os.14645.3.S1_at	2.7570155	up	1.33655	up	1.409717	up	3.68489	up
Os.5301.1.S1_at	-1.4564662	down	-1.49958	down	-1.25607	down	-2.18409	down
Os.37128.1.S1_at	-1.7651567	down	1.589804	up	-2.99439	down	-1.1103	down
Os.37822.1.A1_a_at	2.0468762	up	2.372364	up	-1.25956	down	4.855934	up
Os.17722.1.S1_at	-3.6355865	down	2.483632	up	-4.67709	down	-1.46382	down
Os.68.1.S1_at	-1.7598451	down	1.131689	up	-2.13092	down	-1.55506	down
OsAffx.27816.1.S1_at	3.9933434	up	1.232467	up	1.527718	up	4.921666	up
Os.16279.1.S1_at	1.8346119	up	-1.3001	down	2.447668	up	1.41113	up
OsAffx.10128.1.S1_at	1.3159792	up	-2.32659	down	1.235806	up	-1.76795	down
Os.8544.1.S1_at	2.6082878	up	2.012522	up	-1.06911	down	5.249236	up
Os.49763.1.S1_s_at	1.4805096	up	3.299421	up	-1.75313	down	4.884824	up
Os.7834.1.S1_at	-1.729828	down	-1.91609	down	1.018438	up	-3.3145	down
Os.57456.1.S1_x_at	1.4241585	up	-3.01751	down	1.603903	up	-2.1188	down
Os.50356.1.A1_at	1.6017174	up	1.75351	up	1.072905	up	2.808628	up
Os.25169.1.S1_at	-2.5270789	down	-1.20919	down	-1.55587	down	-3.05573	down
Os.47997.1.A1_at	1.3074172	up	-1.06985	down	2.012734	up	1.222053	up
Os.19899.1.S1_x_at	-2.0751865	down	1.107852	up	-2.08356	down	-1.87316	down
Os.46569.1.S1_at	-2.3912928	down	-1.43635	down	-1.30931	down	-3.43474	down
Os.6274.1.S1_at	2.4724252	up	-7.6787	down	14.43679	up	-3.10573	down
Os.49999.1.S1_x_at	1.8121132	up	2.408756	up	-1.28148	down	4.364938	up
Os.21300.2.S1_at	2.2671208	up	-1.08572	down	2.040783	up	2.088127	up
Os.26729.1.A1_at	1.5410718	up	1.903857	up	-1.01298	down	2.93398	up
Os.11198.1.S1_a_at	2.313	up	-1.205	down	2.264661	up	1.919508	up
Os.10700.1.S1_at	-1.4969673	down	1.233469	up	-2.3178	down	-1.21362	down
OsAffx.19111.1.S1_s_	1.1294353	up	1.976115	up	-1.05164	down	2.231894	up
Os.22413.1.S1_at	2.0319765	up	1.082717	up	1.735529	up	2.200054	up
Os.7913.1.S1_a_at	2.089575	up	1.34195	up	1.400261	up	2.804104	up
Os.53733.1.S1_at	1.1519798	up	-2.65116	down	4.981091	up	-2.3014	down
Os.10099.2.S1_at	3.0991678	up	-1.17372	down	2.205217	up	2.640459	up
OsAffx.15177.1.S1_at	1.285233	up	-3.11769	down	5.854266	up	-2.42578	down
OsAffx.6057.1.S1_s_a	1.42675	up	1.578047	up	1.189691	up	2.251479	up
Os.38599.3.S1_a_at	-1.3124534	down	-1.80242	down	-1.0412	down	-2.36559	down
Os.37986.1.S1_at	-2.5560029	down	-1.36094	down	-1.37883	down	-3.47856	down
Os.5469.1.S1_at	-2.1274083	down	-1.04954	down	-1.78776	down	-2.2328	down
Os.49030.1.A1_s_at	-3.009271	down	1.91729	up	-3.59678	down	-1.56954	down
Os.2319.1.S1_at	-1.974413	down	-1.10192	down	-1.7024	down	-2.17564	down
Os.10398.1.S2_at	-2.5156858	down	1.071118	up	-2.00909	down	-2.34866	down
Os.22508.1.S1_at	-1.7592794	down	-1.60583	down	-1.16805	down	-2.82511	down
Os.5486.1.S1_at	-2.8890162	down	1.56589	up	-2.93676	down	-1.84497	down
Os.15401.1.S1_at	-1.3578115	down	-1.76862	down	-1.06036	down	-2.40146	down

Os.10500.1.S1_at	1.1374407	up	2.448784	up	-1.306	down	2.785346	up
OsAffx.24885.1.S1_at	-3.4415224	down	1.343331	up	-2.51855	down	-2.56193	down
Os.16540.1.S1_at	1.5032212	up	1.672664	up	1.120487	up	2.514384	up
Os.25250.1.S1_at	-3.5892081	down	1.059254	up	-1.98518	down	-3.38843	down
OsAffx.30548.1.S1_at	-3.2487824	down	1.884023	up	-3.53061	down	-1.72439	down
Os.54846.1.S1_s_at	-2.2903352	down	1.031008	up	-1.93175	down	-2.22145	down
Os.49848.1.S1_at	2.5299287	up	2.094429	up	-1.11786	down	5.298756	up
Os.6862.2.A1_at	-2.1713464	down	1.067378	up	-1.99976	down	-2.03428	down
Os.28623.1.S1_at	1.1975801	up	-1.4079	down	2.637047	up	-1.17562	down
Os.20482.1.S1_at	-1.55527	down	1.150466	up	-2.15444	down	-1.35186	down
Os.26927.1.S1_at	3.0610518	up	2.070437	up	-1.10575	down	6.337716	up
Os.47940.1.S1_at	-2.390405	down	-1.31791	down	-1.42065	down	-3.15035	down
OsAffx.5423.1.S1_at	1.6396396	up	-1.73807	down	3.254052	up	-1.06003	down
Os.4671.2.S1_a_at	-3.090268	down	1.015118	up	-1.90035	down	-3.04424	down
Os.50335.1.S1_at	-1.94818	down	-1.35264	down	-1.38384	down	-2.63519	down
Os.50299.1.S1_at	-2.3183484	down	1.100511	up	-2.05995	down	-2.10661	down
Os.16964.1.S1_x_at	-2.6577218	down	2.035142	up	-3.80926	down	-1.30591	down
Os.46312.1.S1_a_at	-1.7720382	down	-1.15655	down	-1.61822	down	-2.04945	down
Os.50568.1.S1_x_at	-2.161554	down	-1.08413	down	-1.72621	down	-2.3434	down
Os.16437.1.S1_at	-1.4527293	down	1.099117	up	-2.0566	down	-1.32172	down
Os.21292.1.S1_at	1.6614581	up	-1.34331	down	2.513449	up	1.236838	up
Os.52133.1.S1_at	-1.8000351	down	-1.35362	down	-1.38205	down	-2.43656	down
Os.26393.1.S1_at	1.23075	up	-1.47352	down	2.756598	up	-1.19726	down
Os.5865.1.S1_at	-4.0971303	down	1.022193	up	-1.91177	down	-4.00818	down
Os.2363.1.S1_a_at	-1.2168936	down	-1.96763	down	1.052438	up	-2.3944	down
Os.11711.1.S1_at	-2.4689963	down	1.351657	up	-2.52654	down	-1.82664	down
Os.41467.1.S1_at	1.9909766	up	1.567042	up	1.192811	up	3.119943	up
Os.10378.1.A1_at	2.0917366	up	1.192125	up	1.567619	up	2.493611	up
OsAffx.7733.1.S1_at	1.4983771	up	1.431716	up	1.305282	up	2.145251	up
Os.39963.1.S1_at	-2.003875	down	1.892183	up	-3.53592	down	-1.05903	down
Os.55193.1.S1_at	1.6636348	up	1.417662	up	1.317666	up	2.358471	up
Os.11491.1.S1_at	2.621756	up	-17.02	down	31.76919	up	-6.49183	down
Os.8279.1.S1_at	1.984854	up	-1.20266	down	2.244775	up	1.650388	up
Os.54563.1.S1_at	-2.192101	down	1.692631	up	-3.15841	down	-1.29509	down
Os.26781.2.S1_x_at	-1.3199445	down	-1.82941	down	-1.01988	down	-2.41473	down
Os.11342.1.S1_at	1.5838387	up	-1.07831	down	2.011843	up	1.468818	up
Os.23915.1.S1_at	-4.6307487	down	1.30922	up	-2.44264	down	-3.53703	down
Os.33610.4.S1_x_at	-2.1268873	down	-1.47597	down	-1.264	down	-3.13922	down
OsAffx.17142.1.S1_s_	-1.7009236	down	-1.43528	down	-1.2998	down	-2.4413	down
Os.49088.2.S1_at	1.6139158	up	1.264446	up	1.475346	up	2.04071	up
Os.5365.1.S1_at	1.0503201	up	2.330532	up	-1.24937	down	2.447805	up
Os.9217.1.S1_at	3.2383807	up	1.196502	up	1.558606	up	3.874729	up
Os.8320.1.S1_at	1.5979455	up	2.080213	up	-1.11604	down	3.324067	up
Os.2357.1.S1_at	1.5038353	up	-1.23992	down	2.311075	up	1.212846	up
OsAffx.25816.1.S1_at	-1.6168252	down	1.458909	up	-2.71914	down	-1.10824	down
Os.51245.1.S1_at	-2.568358	down	-2.06638	down	1.10878	up	-5.30721	down
Os.47897.1.S1_at	-1.5986197	down	-1.37601	down	2.564215	up	-2.19972	down

Os.11062.1.S1_at	2.1089187	up	1.003635	up	1.856623	up	2.116583	up
Os.40907.2.S1_at	-2.6544771	down	-1.29387	down	-1.43938	down	-3.43454	down
Os.48024.1.A1_at	4.690711	up	-1.34012	down	2.49559	up	3.500209	up
Os.8805.1.S1_at	-1.2861749	down	-1.65597	down	-1.12452	down	-2.12987	down
Os.35495.1.S1_at	1.4208304	up	1.985298	up	-1.06623	down	2.820771	up
Os.4008.1.S1_a_at	-2.19755	down	1.170241	up	-2.17881	down	-1.87786	down
Os.43995.1.S1_at	-3.2595396	down	1.095284	up	-2.03908	down	-2.97598	down
Os.49688.1.S1_at	-1.3871405	down	-1.72478	down	-1.07924	down	-2.39252	down
Os.55283.1.S1_at	2.0332592	up	-1.25028	down	2.327154	up	1.626242	up
Os.46466.1.S1_at	-2.3289366	down	-1.49337	down	-1.24632	down	-3.47797	down
Os.11211.1.S1_at	-1.7390198	down	-1.18323	down	-1.57275	down	-2.05766	down
Os.33971.1.S1_at	-4.3152027	down	1.142316	up	-2.12535	down	-3.77759	down
Os.27230.1.A1_at	2.3453004	up	-1.31157	down	2.439836	up	1.788166	up
OsAffx.11507.1.S1_at	-2.382762	down	3.279789	up	-1.7632	down	1.376465	up
Os.7254.1.S1_at	-1.434077	down	-1.5887	down	-1.17079	down	-2.27832	down
OsAffx.24125.1.S1_s_	-2.069535	down	1.019659	up	-1.89533	down	-2.02963	down
Os.57301.1.S1_at	1.9104785	up	1.429721	up	1.29984	up	2.731452	up
OsAffx.22839.1.S1_at	2.0963397	up	1.092396	up	1.701141	up	2.290034	up
Os.7770.1.S1_at	2.1775756	up	1.862696	up	-1.00242	down	4.056161	up
Os.27253.1.A1_at	-2.4073188	down	-1.13703	down	-1.63416	down	-2.73719	down
Os.53824.1.S1_x_at	-1.5363361	down	-1.38998	down	-1.33572	down	-2.13547	down
Os.20904.1.S1_at	-1.2923856	down	-1.6726	down	-1.10994	down	-2.16165	down
Os.50284.1.S1_at	-1.9854559	down	-1.05766	down	-1.75449	down	-2.09994	down
Os.49142.1.S1_at	-2.8587642	down	1.143264	up	-2.12147	down	-2.50053	down
Os.40034.1.A1_s_at	-1.8123295	down	-1.30716	down	-1.41953	down	-2.36901	down
Os.50587.1.S1_at	6.5311127	up	-1.10627	down	2.052575	up	5.903715	up
Os.22379.1.S1_at	-1.6088905	down	-1.48644	down	-1.24794	down	-2.39152	down
Os.27683.2.S1_s_at	2.294947	up	1.130372	up	1.640953	up	2.594142	up
Os.6164.1.S1_at	-1.9174583	down	-1.30675	down	-1.41944	down	-2.50563	down
Os.32357.1.S1_at	-3.223598	down	1.00343	up	-1.86089	down	-3.21258	down
Os.2160.2.S1_x_at	-1.2707276	down	1.265954	up	-2.34763	down	-1.00377	down
Os.11465.1.S1_at	-1.0255003	down	2.037802	up	-1.0989	down	1.98713	up
Os.48102.1.A1_s_at	1.6537372	up	1.522598	up	1.217886	up	2.517977	up
Os.7357.1.S1_at	-2.1938696	down	1.624406	up	-3.01202	down	-1.35057	down
Os.49538.1.S1_at	-2.958518	down	2.030999	up	-3.76543	down	-1.45668	down
OsAffx.2318.2.A1_at	-1.3465004	down	-1.79463	down	-1.033	down	-2.41647	down
Os.4757.1.S2_at	-1.9050112	down	-1.19192	down	-1.55509	down	-2.27062	down
Os.7495.1.S1_at	-2.2334461	down	-1.13856	down	-1.6278	down	-2.54291	down
Os.38249.2.S1_at	-1.5764737	down	1.419288	up	-2.62907	down	-1.11075	down
Os.28394.2.S1_s_at	-1.2351568	down	2.626293	up	-1.41783	down	2.126283	up
Os.8615.1.S1_at	-3.0143044	down	1.606538	up	-2.97556	down	-1.87627	down
Os.54609.1.S1_at	1.3823137	up	-1.10604	down	2.048218	up	1.249786	up
OsAffx.18875.1.S1_at	-2.7936938	down	1.302626	up	-2.41212	down	-2.14466	down
Os.46653.1.S1_at	-1.8091308	down	-1.40059	down	-1.32199	down	-2.53385	down
Os.5124.1.S1_at	2.6362233	up	-1.07503	down	1.990071	up	2.452234	up
Os.12048.1.S1_at	1.4242676	up	1.678209	up	1.103039	up	2.390218	up
Os.16068.3.S1_at	-1.3003767	down	-2.75639	down	1.489324	up	-3.58434	down

Os.51701.1.S1_at	-2.9253259	down	-1.14571	down	-1.61509	down	-3.35156	down
Os.54439.1.S1_at	-1.8785541	down	1.44333	up	-2.67066	down	-1.30154	down
Os.372.1.S1_a_at	-1.7404734	down	1.816345	up	-3.36031	down	1.043592	up
OsAffx.31019.1.S1_at	-1.851039	down	-1.25265	down	-1.47669	down	-2.31871	down
OsAffx.24166.1.S1_at	1.2925706	up	5.027964	up	-2.71866	down	6.498998	up
Os.20336.1.S1_at	-1.7809354	down	-1.37058	down	-1.34905	down	-2.44091	down
Os.27371.1.A1_at	-3.4837673	down	1.124385	up	-2.07881	down	-3.09838	down
Os.51150.1.S1_at	1.6321733	up	-2.1685	down	1.172938	up	-1.32859	down
Os.55262.1.S1_at	-1.2465372	down	-1.91274	down	1.03472	up	-2.3843	down
OsAffx.31257.1.S1_s_	1.7555758	up	1.773017	up	1.04255	up	3.112666	up
Os.53190.1.S1_at	1.285905	up	2.018326	up	-1.0921	down	2.595375	up
Os.36328.1.S1_at	1.2778405	up	1.658567	up	1.114267	up	2.119384	up
Os.53258.1.S1_at	2.5570538	up	1.235084	up	1.496168	up	3.158177	up
Os.4071.2.S1_x_at	-2.3151464	down	-1.17311	down	-1.57517	down	-2.71593	down
Os.7812.2.S1_x_at	2.0392642	up	1.216965	up	1.518139	up	2.481714	up
Os.14276.1.S1_at	2.175569	up	1.019211	up	1.812415	up	2.217365	up
Os.53417.1.S1_at	-1.8664311	down	-1.22155	down	-1.51215	down	-2.27994	down
Os.8684.1.S1_a_at	2.4575121	up	1.853637	up	-1.00354	down	4.555335	up
Os.32022.1.S1_x_at	3.0667489	up	1.030928	up	1.79167	up	3.161598	up
Os.52191.1.A1_at	-1.5168457	down	-1.58466	down	-1.16556	down	-2.40368	down
Os.49104.1.A1_at	-2.5063622	down	1.379402	up	-2.54732	down	-1.81699	down
Os.8509.1.S1_at	1.780967	up	-3.95868	down	7.308703	up	-2.22277	down
Os.10528.1.S1_s_at	-1.168151	down	-1.9804	down	1.072679	up	-2.31341	down
Os.53305.1.S1_at	1.3629694	up	-2.52946	down	1.37008	up	-1.85584	down
Os.32430.1.S1_at	-2.5028758	down	1.232894	up	-2.27616	down	-2.03008	down
OsAffx.25534.1.S1_at	1.3070154	up	-1.70272	down	3.143487	up	-1.30275	down
Os.14750.1.S1_x_at	-3.6667929	down	1.378557	up	-2.54497	down	-2.65988	down
Os.49925.2.S2_at	-2.3152442	down	-1.03738	down	-1.77902	down	-2.40178	down
Os.52440.1.S1_at	-1.1691703	down	-2.21072	down	1.197896	up	-2.58471	down
OsAffx.4947.1.S1_at	-3.1255622	down	-2.45373	down	1.329638	up	-7.6693	down
OsAffx.4214.1.S1_x_a	1.8133645	up	-1.06645	down	-1.73021	down	1.700382	up
Os.24827.1.S1_at	1.4475344	up	-1.10469	down	2.037611	up	1.31035	up
Os.1465.1.S1_at	-2.3703613	down	-1.26967	down	-1.45235	down	-3.00957	down
Os.49022.1.S1_at	-1.9343723	down	-1.06466	down	-1.73198	down	-2.05945	down
OsAffx.28253.1.S1_at	-2.3962116	down	1.511523	up	-2.78714	down	-1.5853	down
Os.8681.1.S1_at	2.8348432	up	-1.45536	down	2.683402	up	1.947869	up
Os.9704.1.S1_at	1.2347211	up	-2.72946	down	1.480455	up	-2.21059	down
Os.6567.1.S1_at	-1.9856083	down	1.204339	up	-2.22039	down	-1.64871	down
OsAffx.25606.1.S1_at	-2.291343	down	1.011333	up	-1.86426	down	-2.26567	down
Os.26889.1.S1_at	1.1656879	up	1.985444	up	-1.07708	down	2.314408	up
Os.12186.2.S1_at	1.8346459	up	-1.22856	down	2.264336	up	1.493326	up
Os.7556.1.S1_x_at	-2.0048442	down	1.625232	up	-2.99506	down	-1.23357	down
Os.46313.1.S1_at	-2.291627	down	-1.00263	down	-1.83785	down	-2.29765	down
Os.50903.1.S1_at	3.0871184	up	1.904007	up	-1.0334	down	5.877896	up
Os.32502.1.S1_at	1.2926649	up	1.587136	up	1.160506	up	2.051635	up
Os.1314.1.S1_at	1.4801595	up	1.828371	up	1.006993	up	2.70628	up
Os.6683.1.S1_at	9.138741	up	1.144305	up	1.608805	up	10.4575	up

Os.28062.1.S1_a_at	-1.4406911	down	1.418071	up	-2.6104	down	-1.01595	down
Os.8899.1.S1_at	-1.0210477	down	2.341168	up	-1.27199	down	2.292907	up
Os.4987.1.S1_at	1.633729	up	-1.27392	down	2.344667	up	1.282443	up
Os.5862.1.S1_at	1.0952877	up	2.509631	up	-1.36358	down	2.748768	up
Os.53874.1.S1_at	-2.1187224	down	1.071955	up	-1.97287	down	-1.9765	down
Os.11330.2.S1_x_at	2.0385842	up	-1.10123	down	2.026682	up	1.851193	up
OsAffx.24597.1.S1_at	-3.0780506	down	1.029982	up	-1.89544	down	-2.98845	down
OsAffx.29702.1.S1_s_	-1.4290155	down	-1.41438	down	-1.30107	down	-2.02118	down
OsAffx.11701.1.S1_s_	-1.6456938	down	-2.75798	down	1.498861	up	-4.53879	down
Os.12838.1.S1_at	1.3974258	up	-1.21323	down	2.232222	up	1.151824	up
Os.5959.1.S1_at	1.7739314	up	1.583636	up	1.16164	up	2.809262	up
OsAffx.14605.1.S1_s_	-1.8521879	down	1.20524	up	-2.21714	down	-1.53678	down
Os.10473.1.S1_at	2.177026	up	1.427833	up	1.287803	up	3.10843	up
Os.4416.1.S1_at	2.121356	up	1.481431	up	1.241197	up	3.142643	up
Os.22277.1.S1_at	-1.5594734	down	-2.2661	down	1.232447	up	-3.53392	down
Os.7307.2.S1_x_at	-1.6492513	down	-1.2456	down	-1.47606	down	-2.05431	down
Os.3395.1.S1_at	-2.2355533	down	1.169009	up	-2.14903	down	-1.91235	down
Os.12363.1.S1_at	-1.4682796	down	1.693631	up	-3.1132	down	1.15348	up
Os.11590.1.S2_at	-2.0363579	down	-1.22964	down	-1.49445	down	-2.50399	down
Os.7307.1.S1_a_at	-1.6586326	down	-1.26712	down	-1.44948	down	-2.10169	down
Os.15967.1.S1_at	-2.1742551	down	-1.14785	down	-1.59992	down	-2.49572	down
Os.55669.1.S1_at	1.5145795	up	1.492962	up	1.229985	up	2.26121	up
Os.53232.1.S1_at	-1.1577036	down	-2.22192	down	1.210158	up	-2.57233	down
Os.49103.1.S1_at	1.3141068	up	2.114697	up	-1.15186	down	2.778937	up
Os.17787.1.S1_at	1.9957588	up	1.567898	up	1.170895	up	3.129146	up
Os.23911.1.S1_at	-4.566416	down	-1.11677	down	-1.64377	down	-5.09963	down
Os.6768.1.S1_at	2.1044266	up	-1.55831	down	2.859908	up	1.350459	up
OsAffx.22754.1.S1_s_	-1.7065333	down	1.158238	up	-2.12533	down	-1.47339	down
Os.27591.1.A1_at	1.3191186	up	-1.6493	down	3.026356	up	-1.25031	down
Os.49498.1.S1_at	-2.1291049	down	-1.18781	down	-1.54471	down	-2.52897	down
Os.11697.1.S1_at	-1.8676596	down	1.688479	up	-3.09803	down	-1.10612	down
OsAffx.17696.1.S1_at	2.0697513	up	1.847202	up	-1.00687	down	3.823249	up
OsAffx.6015.1.S1_at	1.0979805	up	28.53601	up	-15.555	down	31.33198	up
OsAffx.4640.1.S1_x_a	2.1385853	up	1.064126	up	1.723911	up	2.275725	up
Os.37225.1.S1_at	1.690109	up	2.937012	up	-1.60134	down	4.96387	up
Os.12153.1.S1_at	1.6517844	up	1.604875	up	1.142682	up	2.650908	up
Os.8825.1.S1_a_at	1.918128	up	2.018148	up	-1.10069	down	3.871065	up
Os.46739.1.A1_x_at	-2.733487	down	1.268	up	-2.32475	down	-2.15575	down
Os.23622.1.A1_at	-2.3113353	down	-1.28913	down	-1.42188	down	-2.97961	down
Os.11846.1.S1_at	-1.7420573	down	6.089928	up	-3.32262	down	3.495825	up
Os.9960.1.S1_at	-2.162703	down	-1.10417	down	-1.65982	down	-2.388	down
Os.49402.1.S1_at	-2.0201619	down	-1.32118	down	-1.38686	down	-2.66899	down
Os.26932.1.S1_at	1.3263192	up	2.399952	up	-1.31003	down	3.183103	up
OsAffx.27338.1.S1_s_	-1.6872829	down	-1.45747	down	-1.25583	down	-2.45916	down
OsAffx.2803.1.S1_at	5.1867194	up	1.30754	up	1.399536	up	6.781845	up
Os.49894.1.S1_at	-2.4831252	down	1.069618	up	-1.95731	down	-2.32151	down
Os.23100.1.S1_at	1.5605255	up	-1.10854	down	2.02845	up	1.407732	up

Os.37509.1.S1_at	2.1542368	up	1.09257	up	1.67419	up	2.353655	up
OsAffx.20934.1.S1_at	1.217141	up	-1.92821	down	3.526605	up	-1.58421	down
Os.12673.1.S1_at	1.6210911	up	1.568582	up	1.165941	up	2.542815	up
Os.13573.1.S1_at	-3.8877919	down	1.133217	up	-2.07189	down	-3.43076	down
Os.9211.1.S1_at	1.6053283	up	2.337017	up	-1.27847	down	3.75168	up
OsAffx.4002.1.S1_s_a	4.1974993	up	-1.06387	down	1.944668	up	3.945514	up
Os.6098.1.A1_at	-1.5043689	down	2.114553	up	-1.15695	down	1.405608	up
Os.16968.1.S1_at	-1.9661888	down	1.597358	up	1.143938	up	-1.2309	down
OsAffx.16631.1.S1_at	2.197343	up	1.118162	up	1.633416	up	2.456986	up
Os.18524.1.S1_at	-2.1092894	down	1.295533	up	-2.36588	down	-1.62812	down
Os.27293.1.S1_at	1.8054333	up	1.155709	up	1.580122	up	2.086556	up
Os.26697.2.S1_x_at	-1.5776404	down	-1.36386	down	-1.33866	down	-2.15169	down
Os.9824.1.S2_at	-1.0457569	down	-1.86622	down	3.406954	up	-1.95161	down
Os.26771.1.A1_at	-1.706099	down	-1.4543	down	-1.25499	down	-2.48118	down
Os.38240.1.S1_a_at	1.3635426	up	2.107748	up	-1.15493	down	2.874004	up
Os.21133.1.S1_at	-1.4701232	down	-1.77787	down	-1.02634	down	-2.61369	down
Os.16365.1.S1_at	-1.5628697	down	1.306693	up	-2.38399	down	-1.19605	down
Os.51480.1.S1_x_at	2.3732665	up	-2.8569	down	5.21204	up	-1.20378	down
Os.54501.1.S1_at	3.370988	up	1.49283	up	1.221975	up	5.03231	up
Os.7246.2.S1_s_at	-1.2763201	down	1.207445	up	-2.20255	down	-1.05704	down
Os.24550.1.S1_at	-1.8452665	down	-1.09237	down	-1.66986	down	-2.01572	down
Os.20597.1.S1_at	-1.446504	down	-1.6659	down	-1.09478	down	-2.40973	down
Os.51833.1.S1_x_at	1.3939364	up	-1.55144	down	2.829405	up	-1.11299	down
Os.8755.1.S1_at	-1.8371986	down	-1.32067	down	-1.38082	down	-2.42634	down
Os.4731.1.A1_s_at	2.4694285	up	1.246651	up	1.462744	up	3.078517	up
Os.6092.1.S1_at	-1.4919403	down	4.263507	up	-2.33818	down	2.857693	up
Os.28181.1.A1_at	-1.7680163	down	-1.42226	down	-1.28199	down	-2.51458	down
Os.24318.1.S1_at	-1.9797479	down	-1.43168	down	-1.27338	down	-2.83436	down
Os.35606.1.S1_at	2.0911338	up	-2.48929	down	4.537259	up	-1.1904	down
Os.11945.1.S1_at	-2.3880672	down	3.029621	up	-1.66221	down	1.26865	up
Os.56249.1.A1_at	-2.7080832	down	-1.24256	down	-1.46683	down	-3.36495	down
Os.10206.1.S1_at	1.5677608	up	2.773312	up	-1.52191	down	4.347889	up
OsAffx.23086.1.S1_at	-2.2392812	down	1.058931	up	-1.92949	down	-2.11466	down
Os.11837.1.S1_at	-3.1588852	down	2.395188	up	-4.36247	down	-1.31885	down
Os.8271.1.S1_at	2.8282237	up	1.066289	up	1.707786	up	3.015704	up
Os.55280.1.S1_at	2.3326447	up	1.222116	up	1.489676	up	2.850763	up
Os.5873.1.S1_at	-2.0407882	down	1.316744	up	-2.3971	down	-1.54987	down
Os.25019.1.S1_at	-1.5787636	down	-1.37693	down	-1.32207	down	-2.17385	down
Os.10499.1.S1_at	-2.0402966	down	-1.27065	down	-1.43216	down	-2.5925	down
Os.22737.1.S1_at	-2.5309021	down	1.400508	up	-2.54812	down	-1.80713	down
Os.19896.1.S1_at	-1.8237586	down	-1.21876	down	-1.49265	down	-2.22272	down
Os.52185.1.A1_at	-4.1882205	down	2.227493	up	-4.05219	down	-1.88024	down
Os.8626.1.S1_a_at	-1.808643	down	-1.38977	down	-1.30887	down	-2.51359	down
Os.2355.1.S1_at	1.2492502	up	2.633516	up	-1.44778	down	3.28992	up
OsAffx.13826.1.S1_at	1.6802303	up	1.49733	up	1.214741	up	2.51586	up
Os.16297.1.S1_a_at	2.177946	up	-1.18664	down	2.158146	up	1.835396	up
Os.50970.1.S1_at	1.5617785	up	2.917006	up	-1.60401	down	4.555717	up

Os.12192.1.S2_a_at	-2.2843451	down	1.757058	up	-3.19526	down	-1.3001	down
Os.16744.1.S1_at	1.3335845	up	-2.92732	down	5.323299	up	-2.19508	down
Os.36529.1.S1_at	-1.95255	down	-1.23318	down	-1.47455	down	-2.40784	down
Os.12642.1.S1_at	3.492732	up	1.080951	up	1.682101	up	3.775473	up
Os.48332.1.A1_s_at	-2.0716822	down	1.09588	up	-1.99211	down	-1.89043	down
OsAffx.6398.1.A1_at	1.8428577	up	-1.56621	down	2.846478	up	1.176633	up
Os.10590.1.S1_at	1.5795017	up	-3.4781	down	6.320716	up	-2.20202	down
Os.11575.1.S1_at	-1.1568011	down	3.50521	up	-1.92899	down	3.030088	up
Os.54586.1.S1_at	-2.1269693	down	-1.05479	down	-1.72268	down	-2.2435	down
Os.2406.2.S1_a_at	-1.2804326	down	2.704147	up	-1.48837	down	2.111901	up
Os.49818.1.S1_s_at	-3.2767193	down	3.139376	up	-5.70335	down	-1.04375	down
Os.50562.3.S1_x_at	-2.5288813	down	-1.23981	down	-1.4649	down	-3.13533	down
Os.12173.1.S1_at	-1.3638481	down	-1.67989	down	-1.08111	down	-2.29112	down
Os.12795.1.S1_at	-2.1318	down	1.529451	up	-2.77668	down	-1.39383	down
OsAffx.14232.1.S1_x_	2.770265	up	1.052678	up	1.72441	up	2.916196	up
Os.8779.1.S1_at	-1.4863625	down	-1.79543	down	-1.01098	down	-2.66866	down
OsAffx.29710.1.S1_x_	1.1525123	up	-1.00949	down	-1.79764	down	1.14168	up
Os.6489.1.S1_at	-1.7794433	down	-1.18971	down	-1.52525	down	-2.11703	down
Os.6682.1.A1_s_at	5.12224	up	-2.1108	down	3.828984	up	2.426687	up
Os.12248.1.S1_at	1.3860978	up	1.618425	up	1.120714	up	2.243295	up
Os.4458.1.S1_at	-1.7122396	down	1.476615	up	-2.6782	down	-1.15957	down
OsAffx.29775.1.S1_at	2.6581917	up	2.806285	up	-1.5473	down	7.459642	up
Os.18646.1.S1_at	-1.898987	down	-1.17062	down	-1.54926	down	-2.22299	down
Os.50282.1.S1_at	-2.399793	down	-1.27006	down	-1.42794	down	-3.04788	down
OsAffx.21381.1.S1_s_	1.3794703	up	1.812171	up	1.000463	up	2.499836	up
Os.12058.1.S1_at	1.3025173	up	1.666689	up	1.087729	up	2.170891	up
Os.47860.1.S1_at	-2.5543702	down	-1.17686	down	-1.5404	down	-3.00615	down
Os.34596.1.S1_at	-1.4066521	down	1.128597	up	-2.04494	down	-1.24637	down
Os.55551.1.S1_at	1.3626946	up	1.626499	up	1.113793	up	2.216421	up
OsAffx.27278.1.S1_at	2.6277745	up	1.890271	up	-1.04348	down	4.967207	up
Os.55059.1.S1_at	2.4447184	up	2.072424	up	-1.14435	down	5.066492	up
OsAffx.17709.1.S1_at	-1.8521608	down	1.419356	up	-2.5704	down	-1.30493	down
Os.20614.1.S1_at	-1.958835	down	-1.03565	down	-1.7482	down	-2.02867	down
Os.27210.1.S1_at	-2.7575521	down	-1.14158	down	-1.58597	down	-3.14796	down
Os.32682.2.S1_x_at	-2.2927723	down	1.11673	up	-2.0217	down	-2.05311	down
Os.25870.2.S1_a_at	1.691577	up	1.392805	up	1.299714	up	2.356036	up
Os.11371.1.S1_at	4.184729	up	-1.94888	down	3.527908	up	2.147246	up
Os.52495.1.S1_at	-2.7428806	down	1.190059	up	-2.15409	down	-2.30483	down
Os.49290.1.S1_x_at	2.020515	up	1.381259	up	1.310374	up	2.790854	up
Os.16011.1.A1_at	-2.1582801	down	1.004749	up	-1.81816	down	-2.14808	down
Os.7694.1.S1_at	-1.9020826	down	1.477137	up	-2.67245	down	-1.28768	down
Os.18518.1.S1_at	-2.4358346	down	1.001918	up	-1.81236	down	-2.43117	down
Os.5163.1.S1_at	-1.3835447	down	2.520714	up	-1.39381	down	1.821925	up
Os.7454.1.S1_a_at	-2.0661972	down	-1.03346	down	-1.74939	down	-2.13533	down
Os.9465.1.S1_s_at	1.4224924	up	-1.28164	down	2.317066	up	1.109899	up
Os.46761.1.S1_at	-1.467206	down	1.052198	up	1.718058	up	-1.39442	down
Os.57007.1.S1_at	-1.9846511	down	-1.01562	down	-1.77939	down	-2.01566	down

Os.12267.1.S1_at	-3.3876944	down	1.074752	up	-1.94187	down	-3.15207	down
Os.625.1.S1_at	3.466174	up	1.317273	up	1.370805	up	4.565896	up
OsAffx.19484.1.S1_at	-1.5151048	down	-1.57741	down	-1.14472	down	-2.38995	down
Os.5530.3.S1_x_at	-1.5737518	down	-1.38755	down	-1.3013	down	-2.18366	down
Os.38796.1.S1_s_at	2.1112926	up	1.803236	up	1.0011	up	3.807158	up
Os.27759.1.S1_at	-2.764547	down	-1.5931	down	-1.13302	down	-4.40421	down
Os.23028.1.S1_s_at	-2.080979	down	-2.1061	down	1.166808	up	-4.38276	down
Os.13917.1.S1_at	2.3574224	up	-1.695	down	3.059417	up	1.390806	up
Os.8079.1.S1_at	-1.7828333	down	-1.12203	down	-1.60858	down	-2.00039	down
Os.10483.1.S1_at	-1.9510115	down	-1.07645	down	-1.67662	down	-2.10016	down
Os.799.2.S1_x_at	-1.6044909	down	-1.59135	down	-1.13405	down	-2.5533	down
Os.27823.1.S1_at	-1.5197104	down	-1.35661	down	-1.33007	down	-2.06166	down
OsAffx.27284.1.S1_s_	-1.8361316	down	-1.09614	down	-1.64602	down	-2.01265	down
Os.470.1.S1_s_at	2.3306208	up	-1.10874	down	2.000271	up	2.102044	up
OsAffx.4669.1.S1_at	2.1393783	up	-1.05975	down	1.911624	up	2.018755	up
Os.54336.1.S1_at	-1.0365158	down	2.257682	up	-1.25162	down	2.178145	up
Os.51482.1.S1_at	1.3598689	up	1.689241	up	1.067697	up	2.297147	up
Os.5717.1.S1_at	1.1144124	up	-1.28925	down	2.325221	up	-1.15689	down
OsAffx.6449.1.S1_at	-1.3953325	down	-1.47125	down	-1.22567	down	-2.05289	down
Os.5590.1.S1_at	-1.8747123	down	-1.22461	down	-1.47242	down	-2.29579	down
Os.50997.1.A1_x_at	-1.5500127	down	-1.50698	down	-1.19643	down	-2.33584	down
OsAffx.12442.1.S1_at	2.9825106	up	-1.06231	down	1.915217	up	2.807578	up
OsAffx.25081.1.S1_at	-1.6938502	down	-1.3909	down	-1.29596	down	-2.35598	down
Os.9116.1.S1_at	1.2190659	up	2.763048	up	-1.53286	down	3.368337	up
Os.7879.2.S1_at	1.2644591	up	-1.33594	down	2.407928	up	-1.05653	down
Os.4762.1.S1_at	-1.3851285	down	1.178687	up	-2.12447	down	-1.17515	down
Os.8768.1.S1_a_at	-1.3878369	down	-1.5217	down	-1.18438	down	-2.11188	down
Os.7516.1.S1_at	-2.006077	down	-1.44782	down	-1.24482	down	-2.90443	down
Os.15360.1.S1_at	-1.9706628	down	-1.03488	down	-1.74149	down	-2.03941	down
Os.53345.1.S1_at	-3.6275187	down	1.852642	up	-3.33879	down	-1.95802	down
Os.28395.1.S1_x_at	-2.0055819	down	-1.1958	down	-1.50701	down	-2.39828	down
Os.39552.1.A1_s_at	7.0370126	up	-15.3851	down	27.72498	up	-2.18631	down
Os.36460.2.S1_x_at	-2.3065772	down	1.096869	up	-1.97599	down	-2.10287	down
Os.50895.1.S1_at	-2.1452057	down	-1.53875	down	-1.17051	down	-3.30093	down
Os.25348.1.S1_at	-1.1736807	down	-2.61175	down	1.450193	up	-3.06536	down
Os.37509.2.S1_x_at	2.1866763	up	1.241281	up	1.450826	up	2.71428	up
OsAffx.25172.1.S1_at	1.4125465	up	1.958764	up	-1.08769	down	2.766845	up
Os.53589.1.S1_at	1.979111	up	1.670451	up	1.078032	up	3.306008	up
Os.46691.1.A1_x_at	-2.7517285	down	1.138037	up	-2.04912	down	-2.41796	down
Os.33723.1.S1_at	-1.1719191	down	-2.50629	down	1.391954	up	-2.93717	down
Os.13544.1.S1_x_at	1.7886235	up	-1.20458	down	2.1689	up	1.484849	up
Os.53830.1.S1_at	-1.1039629	down	2.346381	up	-1.30321	down	2.125417	up
Os.17299.1.S1_at	-1.8391305	down	-1.39468	down	-1.29029	down	-2.56499	down
OsAffx.15162.1.S1_at	-2.397248	down	1.283758	up	-2.30997	down	-1.86737	down
Os.7727.1.S1_at	-1.9580911	down	-2.50538	down	1.39248	up	-4.90577	down
OsAffx.14941.1.S1_at	1.0599526	up	-1.30853	down	2.353799	up	-1.23452	down
Os.55402.1.S1_at	-3.1476865	down	-1.95661	down	1.088308	up	-6.15879	down

Os.48153.1.S1_at	-3.0535731	down	-1.01772	down	-1.76642	down	-3.10768	down
Os.51405.1.S1_at	-2.3207293	down	1.12202	up	-2.01656	down	-2.06835	down
OsAffx.24232.2.S1_s_	2.2229946	up	1.385991	up	1.296585	up	3.08105	up
Os.6534.1.A1_at	1.596876	up	-1.1505	down	2.067395	up	1.38799	up
Os.12610.1.S1_at	-1.4099857	down	1.187967	up	-2.13461	down	-1.18689	down
Os.1726.1.S1_at	2.353093	up	1.784784	up	1.006509	up	4.199762	up
Os.9687.1.S1_at	-2.865923	down	1.025582	up	-1.84208	down	-2.79444	down
OsAffx.17972.1.S1_at	-1.459659	down	-2.12531	down	1.183302	up	-3.10223	down
Os.54069.1.S1_at	-2.177077	down	-1.30704	down	-1.37401	down	-2.84554	down
Os.19628.1.S1_a_at	-1.4743664	down	-1.54607	down	-1.16155	down	-2.27947	down
Os.1423.1.S1_at	-2.7823396	down	-1.34652	down	-1.33365	down	-3.74647	down
Os.25122.1.S1_at	-1.7375273	down	-1.34393	down	-1.33606	down	-2.33511	down
Os.47963.1.S1_at	-1.2088367	down	-1.73861	down	-1.03264	down	-2.1017	down
Os.37321.1.S1_at	1.2671658	up	1.709935	up	1.049921	up	2.166771	up
Os.16743.2.A1_at	-1.3339525	down	-1.66775	down	-1.07636	down	-2.22471	down
Os.21877.1.S1_at	-1.4505264	down	-1.42816	down	-1.25686	down	-2.07158	down
Os.6548.1.S1_at	1.3243872	up	1.800983	up	-1.00348	down	2.385198	up
Os.24475.1.S2_at	-2.965516	down	1.465264	up	-2.62955	down	-2.02388	down
Os.48834.1.S1_at	-1.5942115	down	-1.47513	down	-1.21641	down	-2.35167	down
OsAffx.23844.1.S1_s_	-2.277731	down	1.005649	up	-1.80441	down	-2.26494	down
Os.26628.1.S1_at	-1.4875667	down	1.175832	up	-2.10938	down	-1.26512	down
Os.46616.1.S1_x_at	-1.2308639	down	1.650286	up	-2.95966	down	1.340754	up
Os.50295.1.S1_at	-1.7003582	down	-1.48545	down	-1.20712	down	-2.5258	down
Os.53074.1.S1_at	-2.4142635	down	1.273477	up	-2.28329	down	-1.89581	down
Os.5303.1.S1_at	-1.3795428	down	-1.6643	down	-1.07695	down	-2.29597	down
Os.53689.1.S1_at	-2.401386	down	-1.01304	down	-1.7691	down	-2.4327	down
OsAffx.27357.1.S1_at	2.0070632	up	1.571866	up	1.140101	up	3.154835	up
Os.27217.1.A1_at	-2.3597882	down	1.043811	up	-1.87029	down	-2.26074	down
Os.47711.1.A1_s_at	-2.38483	down	1.054913	up	-1.88991	down	-2.26069	down
Os.8107.1.S1_at	-1.8636682	down	-1.27504	down	-1.40454	down	-2.37626	down
Os.57527.1.S1_at	-1.3602989	down	1.344936	up	-2.40828	down	-1.01142	down
Os.25110.1.S1_a_at	-1.8930925	down	1.466991	up	-2.62567	down	-1.29046	down
Os.40052.1.A1_s_at	1.1954652	up	1.673105	up	1.069755	up	2.000139	up
Os.47087.1.S1_at	-2.4321282	down	1.010906	up	-1.809	down	-2.40589	down
Os.46757.1.S1_s_at	1.2324659	up	2.026878	up	-1.13269	down	2.498058	up
Os.11983.1.S1_at	1.8557096	up	1.316407	up	1.358986	up	2.442869	up
Os.16994.1.S1_at	-2.0285902	down	-1.06712	down	-1.6755	down	-2.16474	down
Os.46308.1.S1_s_at	-3.023286	down	1.607086	up	-2.87306	down	-1.88122	down
Os.12664.1.S1_at	-1.9503973	down	1.385332	up	-2.4764	down	-1.40789	down
Os.6044.1.S1_at	1.689495	up	2.505824	up	-1.40254	down	4.233577	up
Os.51166.1.S1_at	-2.4862552	down	-1.55873	down	-1.14615	down	-3.87539	down
OsAffx.28001.1.S1_s_	1.7536111	up	-1.19914	down	2.142054	up	1.462389	up
Os.20002.1.S1_at	-2.271957	down	-1.14917	down	-1.55441	down	-2.61087	down
Os.22058.1.S1_at	2.5775168	up	-1.81963	down	3.250014	up	1.416508	up
Os.5277.1.S1_at	3.6120331	up	-2.42033	down	4.321971	up	1.492375	up
Os.52362.1.S1_at	1.1772605	up	1.833894	up	-1.027	down	2.158971	up
OsAffx.12897.1.S1_at	-1.4200583	down	1.200913	up	-2.14444	down	-1.18248	down

Os.38179.2.S1_at	-1.9552876	down	-1.05139	down	-1.69831	down	-2.05577	down
Os.20269.1.S1_at	-2.8246465	down	1.241022	up	-2.21546	down	-2.27606	down
Os.411.2.S1_x_at	4.580067	up	-2.76071	down	4.928227	up	1.659021	up
Os.53334.2.S1_x_at	-3.2065678	down	-1.18026	down	-1.51237	down	-3.78459	down
Os.52326.1.S1_at	-2.603672	down	-1.14457	down	-1.55938	down	-2.98009	down
Os.15037.1.S2_at	-2.6019382	down	-1.1166	down	-1.59786	down	-2.90533	down
Os.18829.1.A1_at	-2.6371233	down	1.522973	up	-2.71718	down	-1.73156	down
OsAffx.15177.1.S1_x_	1.603761	up	-2.38485	down	4.254519	up	-1.48703	down
Os.16884.1.S1_at	2.3634262	up	-1.59191	down	2.839805	up	1.484648	up
Os.27419.1.S1_at	-1.1998799	down	2.061439	up	-1.15584	down	1.718038	up
Os.26062.1.S1_a_at	1.5571618	up	1.345069	up	1.325492	up	2.094491	up
Os.47765.1.S1_at	1.4827645	up	1.474719	up	1.208723	up	2.18666	up
Os.52621.1.S1_at	-1.9968331	down	-1.43597	down	-1.24133	down	-2.86738	down
OsAffx.4776.1.S1_s_a	-2.6926107	down	1.028993	up	-1.83367	down	-2.61674	down
OsAffx.28914.1.S1_at	1.5613137	up	1.411741	up	1.262143	up	2.204171	up
Os.35335.1.A1_s_at	1.0872365	up	3.121199	up	-1.75194	down	3.393481	up
Os.26944.2.S1_x_at	2.2102604	up	-1.06552	down	1.897718	up	2.074349	up
Os.6023.1.S1_s_at	4.1358223	up	-1.20073	down	2.138228	up	3.44442	up
Os.11590.1.S1_at	-1.562017	down	-1.33901	down	-1.3296	down	-2.09155	down
OsAffx.31686.1.S1_s_	-1.5345585	down	-1.70615	down	-1.04348	down	-2.61819	down
Os.12948.1.S1_at	2.7340484	up	2.945749	up	-1.65481	down	8.05382	up
Os.7343.1.S1_at	-1.108914	down	-1.30557	down	2.323931	up	-1.44777	down
Os.16460.1.S1_at	1.6730235	up	-1.13837	down	2.026203	up	1.469667	up
Os.26849.1.S1_at	1.5810076	up	3.285315	up	-1.84595	down	5.194109	up
Os.1741.4.S1_x_at	-1.2512579	down	-1.63824	down	-1.08611	down	-2.04986	down
Os.23209.1.S1_at	1.4383278	up	3.553718	up	-1.99729	down	5.111411	up
Os.28385.1.S1_x_at	-2.3528094	down	-1.08275	down	-1.64322	down	-2.54752	down
Os.8743.1.S1_at	1.5494856	up	1.651952	up	1.076604	up	2.559676	up
Os.54718.1.S1_at	-1.4864656	down	-1.36256	down	-1.30505	down	-2.0254	down
Os.18612.1.S1_s_at	-2.13858	down	1.441315	up	-2.56263	down	-1.48377	down
Os.2759.1.S1_s_at	-1.813363	down	2.323775	up	-4.12986	down	1.281473	up
Os.7930.1.S1_x_at	1.3433456	up	1.783199	up	-1.00341	down	2.395453	up
Os.9535.2.S1_x_at	2.2531178	up	-1.53131	down	2.721315	up	1.471367	up
Os.50187.1.S1_at	-1.8710343	down	-1.30576	down	-1.36075	down	-2.44313	down
Os.9123.1.S1_a_at	1.7590843	up	2.175151	up	-1.22432	down	3.826275	up
Os.56382.1.S1_at	-1.1344682	down	2.524373	up	-1.42094	down	2.22516	up
Os.49208.1.S1_at	-1.6457845	down	1.806444	up	-3.20832	down	1.097619	up
Os.51750.1.S1_at	-2.0463538	down	-1.32079	down	-1.34434	down	-2.70281	down
Os.9717.1.S1_at	-1.8164009	down	1.2069	up	-2.14287	down	-1.50501	down
OsAffx.20071.1.S1_x_	-1.9390917	down	-1.11165	down	-1.5967	down	-2.1556	down
Os.19315.1.S1_s_at	-1.4069141	down	3.119946	up	-1.75788	down	2.217581	up
Os.4415.1.S1_at	1.3644844	up	1.716706	up	1.033831	up	2.342419	up
Os.953.1.S2_at	-1.7915249	down	1.149554	up	-2.04011	down	-1.55845	down
Os.5034.1.S1_at	1.0375264	up	-2.45213	down	1.381869	up	-2.36344	down
Os.12475.1.S1_s_at	-1.6196156	down	2.196096	up	-3.89694	down	1.355937	up
Os.10378.2.A1_s_at	1.7191248	up	1.346324	up	1.317809	up	2.314498	up
Os.14665.1.S1_at	2.8674257	up	-1.26959	down	2.252279	up	2.258544	up

OsAffx.22431.1.S1_s_	1.4855196	up	1.481246	up	1.197152	up	2.20042	up
Os.50890.1.S1_at	2.5593963	up	1.224374	up	1.44808	up	3.133657	up
Os.8899.1.S1_x_at	1.2104481	up	1.909999	up	-1.07729	down	2.311955	up
Os.22508.2.S1_x_at	-1.586246	down	-1.43997	down	-1.23111	down	-2.28414	down
OsAffx.26304.1.S1_s_	-2.439403	down	2.171476	up	-3.84899	down	-1.12338	down
Os.10884.1.S1_at	1.2364949	up	2.283425	up	-1.28844	down	2.823443	up
OsAffx.22776.1.S1_at	1.484059	up	1.744487	up	1.015847	up	2.588921	up
Os.7715.1.S1_a_at	1.5636395	up	1.287755	up	1.375948	up	2.013585	up
Os.14381.2.S1_x_at	-2.447092	down	-1.07158	down	-1.65347	down	-2.62226	down
Os.5356.1.S1_at	-2.051495	down	1.155047	up	-2.04647	down	-1.77611	down
OsAffx.16129.1.S1_at	2.4470804	up	-1.64759	down	2.919057	up	1.485251	up
Os.11335.1.S1_at	1.4746212	up	10.99363	up	-6.20732	down	16.21144	up
Os.12043.1.S1_at	-1.6947845	down	2.409736	up	-1.36062	down	1.421854	up
Os.33755.1.S1_at	1.6569846	up	-1.31538	down	2.329525	up	1.259704	up
Os.17147.1.S1_x_at	-1.1468304	down	7.229733	up	-4.08235	down	6.3041	up
Os.51716.1.S1_at	-3.7438989	down	1.373459	up	-2.43222	down	-2.72589	down
Os.46844.1.S1_at	4.614318	up	-1.1107	down	1.966818	up	4.154433	up
Os.4606.1.S1_at	-1.1596128	down	-1.07688	down	1.906889	up	-1.24876	down
Os.10761.1.S1_at	1.3810574	up	1.694044	up	1.04515	up	2.339572	up
Os.9117.1.S1_at	-1.9381627	down	-1.13829	down	-1.55503	down	-2.2062	down
Os.50896.1.S1_at	3.601153	up	1.783995	up	-1.0079	down	6.424441	up
Os.51683.1.S1_at	-4.1898575	down	1.489875	up	-2.63701	down	-2.81222	down
Os.19198.1.S1_at	-1.1862041	down	-1.02392	down	1.812254	up	-1.21457	down
Os.48315.1.S1_at	-2.8727486	down	-1.07653	down	-1.64327	down	-3.09259	down
Os.46347.1.A1_at	2.457952	up	-1.14634	down	2.027591	up	2.144167	up
OsAffx.18758.1.S1_at	-1.7246819	down	-1.61899	down	-1.09249	down	-2.79225	down
Os.7699.1.S1_at	-5.3882923	down	-2.13368	down	1.206462	up	-11.4969	down
Os.16872.1.S1_at	-1.272123	down	1.186778	up	-2.09792	down	-1.07191	down
OsAffx.24158.1.S1_at	1.3003863	up	1.899331	up	-1.07463	down	2.469864	up
Os.23517.1.A1_at	1.4646883	up	2.431124	up	-1.37632	down	3.560839	up
Os.54784.1.S1_at	1.3135102	up	1.864452	up	-1.05583	down	2.448976	up
Os.21415.1.S1_at	-1.1422992	down	-1.77166	down	1.003767	up	-2.02377	down
Os.38205.1.S1_at	-3.0438201	down	1.058655	up	-1.86843	down	-2.87518	down
Os.18034.1.S1_at	-2.1546006	down	-1.53393	down	-1.15051	down	-3.305	down
OsAffx.24671.1.S1_s_	-3.8118522	down	3.195976	up	-1.81118	down	-1.1927	down
Os.47341.1.A1_at	-1.0355977	down	2.177205	up	-1.23411	down	2.102365	up
Os.33892.1.S1_at	-2.0135765	down	-1.08628	down	-1.62398	down	-2.18731	down
Os.27421.3.S1_x_at	-3.6776552	down	1.661211	up	-2.93018	down	-2.21384	down
Os.10548.1.S1_s_at	-1.3522992	down	-1.54659	down	-1.14044	down	-2.09146	down
Os.282.2.S1_a_at	1.1660138	up	1.858919	up	-3.27873	down	2.167525	up
Os.4354.1.S1_at	-2.0282874	down	-1.09238	down	-1.61444	down	-2.21565	down
Os.3277.1.S1_a_at	-2.4541252	down	-1.08943	down	-1.6182	down	-2.6736	down
Os.40428.1.S1_at	2.5184722	up	2.147246	up	-1.21807	down	5.407779	up
Os.9804.1.S1_at	-1.5073004	down	-1.48257	down	-1.18903	down	-2.23469	down
Os.23760.1.S1_at	-2.574809	down	-1.23499	down	-1.42712	down	-3.17985	down
Os.48005.1.S1_x_at	2.0490375	up	1.237676	up	1.423516	up	2.536045	up
Os.11764.1.S1_a_at	-2.3497458	down	1.090895	up	-1.92173	down	-2.15396	down

Os.49623.1.A1_at	1.770049	up	1.581749	up	1.113633	up	2.799774	up
Os.47310.1.S1_at	-2.2743266	down	1.426619	up	-2.5128	down	-1.59421	down
Os.46800.1.S1_a_at	1.9313958	up	1.889998	up	-1.07337	down	3.650334	up
Os.6764.2.S1_at	-1.155425	down	-2.73698	down	1.554541	up	-3.16238	down
Os.9874.1.S1_s_at	-2.0373967	down	1.196707	up	-2.10666	down	-1.7025	down
Os.2616.1.S1_at	-1.482986	down	-1.39015	down	-1.26633	down	-2.06157	down
Os.7628.1.S1_at	-1.49442	down	-1.52468	down	-1.15446	down	-2.27851	down
Os.20387.1.S1_at	1.1563148	up	3.51174	up	-1.99524	down	4.060678	up
Os.17416.1.S1_at	-2.6686301	down	-1.97168	down	1.120402	up	-5.26169	down
Os.27467.2.S1_at	1.2268035	up	-1.23294	down	2.169665	up	-1.005	down
Os.22678.1.A1_s_at	-2.5815873	down	-1.37849	down	-1.27643	down	-3.55868	down
OsAffx.23396.1.S1_s_	-1.5184501	down	-1.31989	down	-1.33296	down	-2.00419	down
Os.23175.1.S1_at	-1.8548794	down	-1.29169	down	-1.36183	down	-2.39594	down
Os.15398.1.S1_at	1.5083376	up	1.728808	up	1.017311	up	2.607626	up
Os.4513.1.S1_at	-2.1646545	down	-1.11727	down	-1.57398	down	-2.41851	down
Os.11185.1.S1_a_at	1.9433024	up	-1.36499	down	2.400383	up	1.423674	up
Os.49308.1.A1_at	-1.4806318	down	-1.42067	down	-1.23759	down	-2.1035	down
Os.18363.1.S1_at	-1.4519063	down	-2.00887	down	1.142733	up	-2.91669	down
Os.47710.2.A1_s_at	1.9888649	up	1.687038	up	1.04202	up	3.35529	up
OsAffx.24747.1.S1_at	1.1418177	up	1.793168	up	-1.02018	down	2.047471	up
Os.17794.1.S1_at	-1.189134	down	1.257376	up	-2.20959	down	1.057388	up
Os.57096.1.S1_at	-1.5272472	down	-1.32644	down	-1.32467	down	-2.02581	down
Os.5243.1.S1_at	-2.8908298	down	-1.18285	down	-1.48539	down	-3.41941	down
OsAffx.13549.1.S1_at	-1.8766595	down	-1.12477	down	-1.56163	down	-2.11081	down
Os.35451.1.S1_at	-1.8317373	down	-1.26227	down	-1.39145	down	-2.31215	down
Os.18946.2.S1_at	2.1767478	up	-1.55141	down	2.72486	up	1.403078	up
Os.51639.1.S1_x_at	5.614844	up	-2.18309	down	3.834243	up	2.57197	up
Os.16303.3.S1_x_at	1.7809067	up	1.254841	up	1.399545	up	2.234755	up
Os.12346.1.S1_at	1.6195105	up	11.92712	up	-6.79178	down	19.3161	up
Os.25243.1.A1_at	1.5342336	up	1.47447	up	1.190992	up	2.262182	up
OsAffx.23740.1.S1_at	-2.9552317	down	1.208323	up	-2.12127	down	-2.44573	down
Os.32637.3.S1_x_at	-2.7802308	down	1.336227	up	-2.34501	down	-2.08066	down
Os.12158.1.S1_at	1.6471298	up	1.977107	up	-1.12662	down	3.256552	up
Os.17022.1.S1_at	-3.0372074	down	-1.1837	down	-1.48171	down	-3.59515	down
Os.5974.1.A1_at	1.185008	up	2.138184	up	-1.2192	down	2.533765	up
Os.41283.1.S1_at	2.0771124	up	1.46196	up	1.199071	up	3.036654	up
Os.6674.1.S1_a_at	-1.0714902	down	1.236595	up	-2.16734	down	1.154089	up
Os.18172.1.S1_s_at	-1.5188214	down	-1.68892	down	-1.03752	down	-2.56517	down
Os.22857.1.A1_at	-2.2397785	down	-1.37692	down	-1.27225	down	-3.08399	down
Os.45875.1.S1_x_at	-2.3290122	down	1.537137	up	-2.69231	down	-1.51516	down
Os.26592.1.S2_at	1.9359457	up	-1.18036	down	2.067325	up	1.640135	up
OsAffx.24921.1.S1_s_	1.4143214	up	-1.43294	down	2.509043	up	-1.01317	down
Os.15390.1.S1_s_at	-1.8709567	down	-1.26474	down	-1.38442	down	-2.36628	down
Os.11473.1.A1_at	1.9454955	up	-1.41291	down	2.473562	up	1.37694	up
Os.53660.1.S1_at	-1.7641954	down	6.77165	up	-3.86801	down	3.838379	up
OsAffx.7038.1.S1_s_a	-2.4031303	down	1.051474	up	-1.84046	down	-2.28549	down
Os.27228.1.S1_at	-3.0906007	down	1.199014	up	-2.09844	down	-2.57762	down

Os.27567.1.A1_at	-3.4019573	down	1.940304	up	-3.39495	down	-1.75331	down
Os.7805.1.S1_a_at	-1.5446461	down	1.160886	up	-2.03088	down	-1.33058	down
Os.49527.1.S1_at	1.6015023	up	5.436048	up	-3.10795	down	8.705843	up
Os.25001.1.S1_at	1.7182134	up	1.318492	up	1.32651	up	2.265451	up
Os.28061.1.S1_at	-2.2912858	down	1.099696	up	-1.92329	down	-2.08356	down
Os.20313.1.S1_s_at	1.5761552	up	2.52622	up	-1.44505	down	3.981715	up
Os.51414.1.S1_at	-1.7393596	down	-1.29229	down	-1.35235	down	-2.24776	down
Os.50369.1.S1_at	-2.2019022	down	-1.03957	down	-1.6808	down	-2.28903	down
Os.12046.1.S1_at	1.1130639	up	-1.24859	down	2.181637	up	-1.12176	down
Os.46623.1.S1_at	-6.0007787	down	2.051742	up	-3.58486	down	-2.92472	down
Os.7704.1.S1_at	-1.4821141	down	-1.47203	down	-1.18683	down	-2.18172	down
Os.46896.1.S1_at	1.4271514	up	1.512235	up	1.155263	up	2.158189	up
Os.49274.1.S1_at	1.5433823	up	-1.18586	down	2.071637	up	1.301492	up
Os.13921.2.S1_a_at	-1.649547	down	-1.35294	down	-1.29119	down	-2.23174	down
Os.23822.1.A1_at	-1.5091738	down	1.242805	up	-2.17103	down	-1.21433	down
Os.47386.1.S1_at	1.182419	up	-1.17978	down	2.060824	up	1.002238	up
Os.10272.1.S1_at	-1.8586907	down	-1.35718	down	-1.28703	down	-2.52258	down
Os.50235.1.S1_a_at	-2.0185907	down	1.057483	up	-1.84712	down	-1.90886	down
Os.5915.1.S1_at	-1.8706869	down	-1.35386	down	-1.29002	down	-2.53264	down
OsAffx.25193.1.S1_x_	-1.8049989	down	1.239185	up	-2.16377	down	-1.4566	down
Os.5187.1.S1_at	1.660385	up	1.37338	up	1.27083	up	2.28034	up
Os.7255.2.S1_at	-3.4352124	down	1.1871	up	-2.07144	down	-2.89379	down
Os.18248.1.S1_at	2.1203973	up	1.082192	up	1.612395	up	2.294677	up
Os.3356.1.S1_at	1.7377567	up	2.007229	up	-1.15039	down	3.488075	up
Os.50564.1.A1_s_at	-2.4978273	down	1.196998	up	-2.08853	down	-2.08674	down
Os.456.3.S1_s_at	-1.4696643	down	1.938994	up	-3.38275	down	1.319345	up
OsAffx.19236.1.S1_at	1.7741185	up	1.323453	up	1.318077	up	2.347962	up
Os.17895.1.S1_at	-2.1247091	down	1.331435	up	-2.32252	down	-1.5958	down
Os.55658.1.S1_at	2.0212154	up	2.906875	up	-1.66644	down	5.87542	up
Os.5990.1.S1_at	-1.3961736	down	1.150032	up	-2.00603	down	-1.21403	down
OsAffx.27922.1.S1_at	1.3917742	up	-2.021	down	1.158966	up	-1.4521	down
Os.13960.2.S1_x_at	-2.2108624	down	-1.23849	down	-1.40746	down	-2.73813	down
OsAffx.16004.1.S1_s_	-2.461085	down	-1.13558	down	-1.53476	down	-2.79476	down
Os.16964.2.S1_at	-2.0591166	down	1.648738	up	-2.87257	down	-1.2489	down
Os.23264.1.A1_at	-5.71137	down	3.858335	up	-6.72214	down	-1.48027	down
Os.25124.1.A1_at	-1.8214207	down	-1.16044	down	-1.50091	down	-2.11365	down
OsAffx.31120.1.S1_at	-1.2641011	down	-1.61119	down	-1.08099	down	-2.03671	down
Os.31936.1.S1_at	-2.1522148	down	1.116928	up	-1.94513	down	-1.92691	down
Os.11939.1.S1_at	-2.2956831	down	1.206321	up	-2.10055	down	-1.90304	down
Os.26914.1.S1_at	-1.8763852	down	1.895408	up	-3.3004	down	1.010138	up
Os.29866.1.S1_at	3.3789215	up	1.173948	up	1.483237	up	3.966679	up
Os.4196.1.S1_at	1.72155	up	1.248268	up	1.394895	up	2.148956	up
Os.7957.1.S1_at	-1.973246	down	-1.17908	down	-1.47672	down	-2.32661	down
Os.26692.1.S1_at	-1.8983116	down	-1.23571	down	-1.40904	down	-2.34577	down
Os.6075.1.S1_at	1.7215346	up	1.389729	up	1.252838	up	2.392467	up
OsAffx.17798.1.S1_at	-1.6164075	down	1.279824	up	1.360275	up	-1.26299	down
Os.11905.1.S1_at	-3.540418	down	1.127066	up	-1.96201	down	-3.14127	down

Os.35797.1.S1_at	-3.335478	down	1.136682	up	-1.97844	down	-2.9344	down
Os.19040.1.S1_at	-1.4965631	down	-1.4154	down	-1.22971	down	-2.11824	down
Os.648.1.S1_at	1.5760735	up	2.176165	up	-1.25052	down	3.429797	up
Os.24609.1.A1_s_at	-2.7441254	down	-1.01454	down	-1.71524	down	-2.78402	down
Os.53935.1.S1_at	-2.4875748	down	-1.15584	down	-1.50548	down	-2.87525	down
Os.53753.1.S1_at	-2.6307297	down	1.682483	up	-2.92644	down	-1.5636	down
Os.46548.2.S1_x_at	-1.0244086	down	2.210317	up	-1.271	down	2.157652	up
Os.55554.1.S1_at	1.1583111	up	2.193629	up	-1.26147	down	2.540904	up
Os.17681.1.S1_at	1.2977483	up	6.195499	up	-3.56309	down	8.040197	up
OsAffx.14635.1.S1_x_	-2.222479	down	-1.31702	down	-1.31982	down	-2.92705	down
Os.54037.1.S1_s_at	1.0881801	up	3.596168	up	-2.06909	down	3.913278	up
Os.54753.1.S1_at	-1.9280654	down	-1.24277	down	-1.398	down	-2.39615	down
Os.53612.1.S1_at	1.4371598	up	1.983843	up	-1.14204	down	2.851099	up
OsAffx.27776.1.S1_at	-1.5113062	down	-1.32788	down	-1.30804	down	-2.00684	down
Os.18125.1.S1_at	-2.1289468	down	-1.00616	down	-1.72584	down	-2.14206	down
Os.35712.1.S1_at	-1.8155861	down	-1.10835	down	-1.56668	down	-2.01231	down
OsAffx.23090.3.S1_x_	-2.4446795	down	1.751932	up	-3.04151	down	-1.39542	down
Os.10590.1.S1_s_at	6.3318634	up	-21.8515	down	37.93533	up	-3.45104	down
Os.35897.1.A1_at	-1.9719204	down	-1.09629	down	-1.58346	down	-2.16179	down
Os.27615.1.S1_at	1.1428763	up	6.078417	up	-3.50198	down	6.946879	up
Os.54288.1.S1_at	-2.4082649	down	-1.14532	down	-1.51512	down	-2.75824	down
Os.15514.1.S1_at	-2.7883952	down	-1.01678	down	-1.7062	down	-2.83518	down
OsAffx.31902.1.S1_x_	-1.6820258	down	1.298478	up	-2.25256	down	-1.29538	down
Os.42815.1.S1_at	-1.5157729	down	-1.70356	down	-1.01823	down	-2.58221	down
Os.36975.3.S1_x_at	1.0900077	up	2.488601	up	-1.4347	down	2.712594	up
Os.9624.1.S1_a_at	-3.4983416	down	1.06717	up	-1.85099	down	-3.27815	down
OsAffx.18678.1.S1_at	-2.2329552	down	-1.01522	down	-1.70803	down	-2.26693	down
Os.32877.1.S1_at	-2.6843865	down	1.264773	up	-2.19295	down	-2.12243	down
Os.46758.1.S1_s_at	1.4046082	up	-1.68694	down	2.924518	up	-1.20101	down
Os.11936.1.S1_at	-2.1946871	down	1.175196	up	-2.03721	down	-1.86751	down
Os.9359.1.S1_at	-1.7311358	down	-1.26164	down	-1.37382	down	-2.18407	down
Os.46723.1.S1_at	-1.9763076	down	1.42472	up	-2.46926	down	-1.38715	down
OsAffx.12360.1.S1_at	-1.0529824	down	-2.29806	down	1.326163	up	-2.41982	down
Os.8943.1.S1_at	-1.9358352	down	-1.04202	down	-1.66287	down	-2.01717	down
Os.11421.2.S1_x_at	1.9502599	up	-2.95636	down	5.122277	up	-1.51588	down
Os.9115.1.S1_at	1.2599097	up	-1.17108	down	2.028257	up	1.075857	up
Os.53925.1.A1_at	3.8727872	up	-1.12311	down	1.94504	up	3.448277	up
Os.52543.1.S1_at	1.2308155	up	1.894205	up	-1.09379	down	2.331418	up
Os.8957.2.S1_x_at	2.3850408	up	-1.09432	down	1.89507	up	2.17947	up
Os.22713.1.S1_at	-1.0919302	down	-1.07996	down	1.870106	up	-1.17924	down
Os.46654.1.S1_at	-2.460285	down	-1.09179	down	-1.58595	down	-2.68612	down
Os.3340.1.S1_a_at	-2.2931552	down	-1.09239	down	-1.58488	down	-2.50502	down
Os.10131.1.S1_a_at	3.6178799	up	-2.09871	down	3.633362	up	1.723859	up
Os.13595.2.S1_x_at	-1.6810385	down	1.261965	up	-2.18444	down	-1.33208	down
Os.54869.1.S1_at	2.139128	up	-1.8379	down	3.180666	up	1.163901	up
Os.16444.1.S1_at	-1.2950178	down	-1.58799	down	-1.08959	down	-2.05647	down
Os.38301.2.A1_at	-2.2805305	down	-1.26355	down	-1.36868	down	-2.88156	down

Os.3416.1.S1_a_at	2.378697	up	-1.09767	down	1.89815	up	2.16705	up
OsAffx.23807.1.S1_x_	1.6015118	up	2.289098	up	-1.32394	down	3.666017	up
Os.20086.1.S1_at	-1.8759235	down	1.414076	up	-2.44489	down	-1.32661	down
OsAffx.16902.1.S1_at	1.5544987	up	-1.24834	down	2.158294	up	1.245253	up
Os.38399.1.S1_at	3.833817	up	-1.78038	down	3.078053	up	2.153364	up
Os.7940.1.S1_at	1.214748	up	2.09903	up	-1.21423	down	2.549793	up
Os.56273.1.S1_at	2.07673	up	1.253809	up	1.378671	up	2.603824	up
Os.39037.1.S1_at	1.6108668	up	2.918737	up	-1.68869	down	4.701697	up
Os.7832.1.S1_at	2.3455124	up	1.505598	up	1.147723	up	3.531399	up
Os.16612.1.S1_at	-2.8025944	down	1.212405	up	-2.09499	down	-2.3116	down
Os.27751.1.S1_at	1.8312125	up	2.054309	up	-1.18889	down	3.761876	up
Os.12746.1.S1_at	1.095083	up	1.933392	up	-1.11907	down	2.117224	up
Os.19611.1.S1_a_at	1.2309262	up	1.676376	up	1.030321	up	2.063495	up
Os.45018.1.S1_x_at	-1.7153323	down	1.37343	up	-2.37153	down	-1.24894	down
Os.32786.1.S1_at	-1.444251	down	1.354971	up	-2.33921	down	-1.06589	down
Os.37051.1.S1_at	-1.819307	down	1.60359	up	-2.76825	down	-1.13452	down
Os.7126.1.S1_at	1.1432582	up	1.851124	up	-1.07236	down	2.116313	up
OsAffx.18404.1.S1_at	4.683367	up	1.852105	up	-1.07301	down	8.674086	up
Os.54410.1.S1_at	2.7208786	up	-7.23185	down	12.48263	up	-2.65791	down
Os.24917.1.S1_at	-1.8748888	down	-1.82014	down	1.054506	up	-3.41256	down
Os.7348.2.S1_x_at	2.1212938	up	1.371898	up	1.25807	up	2.910198	up
Os.39181.1.A1_at	-1.2452906	down	-1.67889	down	-1.02789	down	-2.0907	down
OsAffx.25530.1.S1_s_	-1.5457306	down	-1.46995	down	-1.17396	down	-2.27215	down
Os.27648.1.S1_at	1.984678	up	1.166718	up	1.479018	up	2.31556	up
Os.33471.1.A1_at	-1.7971287	down	-1.23702	down	-1.39472	down	-2.22308	down
Os.38200.1.S2_at	-3.5053332	down	1.297704	up	-2.2388	down	-2.70118	down
Os.18948.1.S1_at	-2.2263856	down	-1.1666	down	-1.47878	down	-2.5973	down
Os.11198.3.S1_x_at	2.8061504	up	-1.513	down	2.609611	up	1.854698	up
OsAffx.4126.1.S1_at	4.169531	up	-1.18877	down	2.050339	up	3.507441	up
Os.18327.1.S1_at	2.1401544	up	1.33843	up	1.288168	up	2.864446	up
Os.12186.1.S1_at	1.9845455	up	-2.09052	down	3.60111	up	-1.0534	down
Os.49736.1.S1_x_at	-2.5449119	down	-1.07022	down	-1.60948	down	-2.72361	down
Os.37097.1.S1_at	1.8843998	up	1.238145	up	1.390951	up	2.33316	up
Os.51442.1.S1_at	-1.0707839	down	3.245709	up	-1.88484	down	3.031153	up
Os.13992.1.S1_at	-2.141056	down	-1.10853	down	-1.55294	down	-2.37342	down
Os.49697.1.S1_at	-1.7742494	down	-1.73653	down	1.009	up	-3.08104	down
Os.46582.2.S1_x_at	2.556966	up	-4.21392	down	7.251995	up	-1.64801	down
OsAffx.11451.3.S1_x_	2.137577	up	1.00512	up	1.712189	up	2.148522	up
Os.2407.1.S1_at	1.7063076	up	1.298179	up	1.325659	up	2.215093	up
Os.23416.2.S1_at	1.9929875	up	1.326374	up	1.297436	up	2.643447	up
Os.3416.1.S1_at	2.5607944	up	-1.22721	down	2.111265	up	2.086682	up
Os.23103.1.S1_at	1.2163798	up	1.917542	up	-1.11465	down	2.332459	up
Os.11765.1.S1_at	-2.0395024	down	-1.27183	down	-1.35228	down	-2.5939	down
Os.52121.1.S1_at	-1.750999	down	1.256869	up	-2.16093	down	-1.39314	down
Os.23567.1.S1_at	-1.881836	down	-1.25094	down	-1.37414	down	-2.35406	down
Os.21782.1.S1_x_at	2.6120436	up	-1.27724	down	2.19543	up	2.04507	up
Os.47889.1.S1_at	-1.6029093	down	-1.46479	down	-1.17313	down	-2.34793	down

Os.51978.1.S1_at	-1.891012	down	-1.12243	down	-1.53077	down	-2.12254	down
Os.6070.1.A1_at	-1.8943828	down	1.927239	up	-3.31062	down	1.017344	up
Os.44618.1.S1_s_at	-1.5784979	down	-1.70041	down	-1.01017	down	-2.68409	down
Os.25266.1.S1_at	1.570017	up	-1.40149	down	2.406564	up	1.120245	up
Os.51419.1.S1_at	-2.0102317	down	-1.0844	down	-1.58347	down	-2.17991	down
Os.11197.2.S1_x_at	1.8748417	up	1.552086	up	1.106253	up	2.909915	up
Os.8024.1.S1_at	-1.598718	down	-1.28965	down	-1.33128	down	-2.06179	down
Os.49277.1.S1_at	-1.7127237	down	1.344299	up	-2.3078	down	-1.27406	down
Os.37620.1.S1_at	3.626782	up	1.235527	up	1.389083	up	4.480987	up
Os.25094.1.A1_at	-1.437643	down	-1.67167	down	-1.02665	down	-2.40326	down
Os.17826.1.A1_at	-1.5975736	down	-1.84945	down	1.077711	up	-2.95464	down
Os.3383.1.S1_a_at	-2.0618143	down	1.087096	up	-1.86548	down	-1.89663	down
Os.54857.1.S1_at	2.095474	up	1.028158	up	1.668863	up	2.154477	up
Os.17718.1.S1_at	1.9712049	up	1.292997	up	1.326998	up	2.548763	up
Os.18712.1.S1_at	4.509171	up	1.372491	up	1.250017	up	6.188798	up
Os.32022.1.S1_at	2.623557	up	1.043049	up	1.644454	up	2.736497	up
Os.3416.2.S1_x_at	2.0586169	up	-1.07813	down	1.849082	up	1.909424	up
Os.27143.1.S1_at	-2.2161212	down	-1.04098	down	-1.64693	down	-2.30693	down
Os.5152.1.S1_at	1.5096314	up	-1.56586	down	2.684331	up	-1.03724	down
Os.32034.1.A1_s_at	-1.7342124	down	1.211466	up	-2.07613	down	-1.4315	down
Os.46541.2.S1_s_at	-2.0747566	down	1.025594	up	-1.75752	down	-2.02298	down
OsAffx.23277.1.S1_at	-1.5512106	down	-1.36362	down	-1.25667	down	-2.11527	down
Os.49118.1.S1_at	-1.8646014	down	-1.16782	down	-1.46655	down	-2.17752	down
Os.53213.1.S1_x_at	1.0397898	up	3.477211	up	-2.03059	down	3.615569	up
Os.12726.1.S1_at	-1.776001	down	1.19458	up	-2.04546	down	-1.48672	down
Os.6597.2.S1_x_at	-1.9848459	down	-1.19605	down	-1.4314	down	-2.37397	down
Os.44541.1.A1_at	-2.0602863	down	1.273725	up	-2.1804	down	-1.61753	down
OsAffx.11835.1.S1_x_	-1.9657152	down	-1.02257	down	-1.67365	down	-2.01009	down
Os.23507.1.A1_at	-1.4064727	down	1.420023	up	-2.43023	down	1.009634	up
Os.25524.1.S1_at	-2.24988	down	1.410338	up	-2.41358	down	-1.59528	down
Os.31112.1.S1_at	-1.9034417	down	-1.35488	down	-1.2629	down	-2.57893	down
Os.49790.1.S1_x_at	-2.1946304	down	1.020633	up	-1.74627	down	-2.15026	down
Os.49770.1.S1_s_at	2.1467516	up	1.039795	up	1.645471	up	2.232182	up
Os.45933.1.S1_x_at	-1.9542931	down	1.247972	up	-2.13522	down	-1.56597	down
OsAffx.13399.1.S1_at	-1.8861115	down	-1.41995	down	-1.20469	down	-2.67819	down
Os.3407.1.S1_a_at	-2.2434752	down	-1.08747	down	-1.57281	down	-2.43971	down
Os.6601.1.S1_at	-3.3329744	down	1.182728	up	-2.02263	down	-2.81804	down
Os.1653.1.S1_at	-1.0473127	down	2.621812	up	-1.53326	down	2.50337	up
Os.11443.1.S1_a_at	-3.941638	down	1.512883	up	-2.58664	down	-2.60538	down
Os.32936.1.S1_at	2.0096936	up	-1.20563	down	2.061206	up	1.666922	up
Os.9405.1.S1_at	-1.3994166	down	-1.51976	down	-1.12466	down	-2.12678	down
Os.41024.1.S1_x_at	2.6196952	up	-1.21585	down	2.077927	up	2.154626	up
Os.20871.1.S1_s_at	1.1802236	up	1.69866	up	1.006075	up	2.004799	up
Os.54612.1.A1_at	-2.0201843	down	-1.25899	down	-1.35717	down	-2.5434	down
Os.19803.3.S1_at	-1.1966871	down	-1.21374	down	2.073748	up	-1.45246	down
Os.13016.1.S1_a_at	-1.0889918	down	2.175327	up	-1.27349	down	1.99756	up
Os.49544.1.S1_at	1.8227869	up	3.77894	up	-2.21256	down	6.888203	up

Os.20457.1.S1_at	-2.6226046	down	-1.01325	down	-1.68512	down	-2.65734	down
Os.46618.1.S1_at	1.73784	up	-1.47609	down	-1.15672	down	1.177325	up
Os.14775.1.S1_at	1.4767455	up	-1.41332	down	2.412855	up	1.044874	up
Os.9553.1.S1_at	1.6524979	up	1.564167	up	1.091184	up	2.584783	up
Os.27338.1.A1_at	-2.0767572	down	-1.1089	down	-1.53911	down	-2.30292	down
Os.11417.1.S1_at	3.371284	up	1.096613	up	1.555985	up	3.696995	up
Os.27605.1.S1_at	-2.2743347	down	1.217359	up	-2.07705	down	-1.86825	down
Os.9749.1.S1_at	1.4450032	up	1.5362	up	1.110511	up	2.219813	up
Os.32557.1.S1_at	-1.6990633	down	-1.26874	down	-1.34458	down	-2.15567	down
Os.54568.1.S1_x_at	-1.7210793	down	1.242397	up	-2.11832	down	-1.38529	down
Os.6803.1.S1_at	-1.3826855	down	-1.12853	down	1.924048	up	-1.5604	down
Os.46548.2.S1_at	-1.152376	down	2.02826	up	-1.19023	down	1.760068	up
OsAffx.22176.1.S1_x_	-2.4784832	down	-1.4599	down	-1.16702	down	-3.61833	down
Os.50562.2.S1_at	-2.25029	down	1.022943	up	-1.7428	down	-2.19982	down
OsAffx.15319.1.S1_at	-2.079828	down	-1.23936	down	-1.3746	down	-2.57766	down
Os.26408.3.S1_x_at	-1.9414966	down	1.349705	up	-2.29938	down	-1.43846	down
Os.14844.1.A1_at	1.6635208	up	-1.38597	down	2.360693	up	1.20026	up
Os.18721.1.S1_at	-2.3025763	down	-1.06957	down	-1.59245	down	-2.46276	down
Os.22660.2.S1_at	1.7504848	up	1.195759	up	1.424248	up	2.093158	up
OsAffx.30086.1.S1_at	-2.020304	down	-1.09728	down	-1.55179	down	-2.21684	down
Os.7478.2.S1_x_at	1.1656386	up	-1.22927	down	2.09313	up	-1.05459	down
Os.2696.1.S1_at	-1.2096169	down	3.583164	up	-2.10487	down	2.96223	up
Os.34890.1.S1_at	-2.3775268	down	1.438616	up	-2.44876	down	-1.65265	down
Os.9778.1.S1_at	-1.3893254	down	1.703878	up	-2.9	down	1.226407	up
Os.55674.1.S1_at	3.4448535	up	-1.30725	down	2.224557	up	2.635181	up
Os.21880.2.S1_x_at	-2.1529024	down	1.044335	up	-1.77697	down	-2.06151	down
Os.51391.1.S1_at	-1.783378	down	1.831949	up	-3.11693	down	1.027235	up
Os.50013.1.S1_at	-2.5864646	down	-1.10259	down	-1.54288	down	-2.85182	down
Os.8712.1.S1_at	-1.0788207	down	1.568451	up	-2.66775	down	1.453857	up
Os.49796.1.S1_at	-2.4531717	down	2.754627	up	-4.6848	down	1.122884	up
Os.56257.1.S1_at	4.1972694	up	-1.40523	down	2.389738	up	2.986898	up
Os.1625.1.S1_at	-1.8425651	down	-1.48018	down	-1.14877	down	-2.72732	down
Os.55660.1.S1_at	-1.7354214	down	-1.29162	down	-1.31643	down	-2.24151	down
Os.27505.1.S1_at	2.0246866	up	1.132578	up	1.500952	up	2.293115	up
Os.49430.1.S1_s_at	-2.5716915	down	1.295354	up	-2.20196	down	-1.98532	down
OsAffx.32325.1.S1_x_	-2.0054483	down	3.12912	up	-5.31894	down	1.560309	up
Os.34890.1.S1_x_at	-2.0284917	down	1.282853	up	-2.18025	down	-1.58123	down
OsAffx.27166.1.S1_at	1.6181277	up	1.478732	up	1.149153	up	2.392777	up
Os.16140.1.S1_at	-2.0642898	down	1.126139	up	-1.9134	down	-1.83307	down
Os.9330.1.S2_x_at	1.5348659	up	1.728059	up	-1.01709	down	2.65234	up
Os.52161.1.S1_x_at	-1.7028503	down	1.215504	up	-2.06479	down	-1.40094	down
Os.54928.1.S1_at	1.2634715	up	1.596972	up	1.063697	up	2.017728	up
Os.49788.2.S1_x_at	-1.6627911	down	-1.85297	down	1.090936	up	-3.08111	down
Os.20203.1.S1_at	-1.5631411	down	1.247021	up	-2.11782	down	-1.2535	down
Os.17018.1.S1_at	-1.8195716	down	-1.14898	down	-1.4778	down	-2.09064	down
Os.12023.1.S1_at	-2.13543	down	1.285375	up	-2.18221	down	-1.66133	down
Os.24334.1.A1_at	-2.8391511	down	1.095941	up	-1.86043	down	-2.59061	down

Os.12830.1.S1_at	1.8222021	up	1.466574	up	1.157172	up	2.672394	up
Os.20108.1.S1_a_at	-1.834301	down	-1.11628	down	-1.52025	down	-2.0476	down
Os.49788.1.S1_at	-1.5008684	down	-2.05423	down	1.210575	up	-3.08313	down
Os.15715.1.S1_at	-2.7848547	down	-1.00348	down	-1.69083	down	-2.79454	down
Os.23243.1.A1_a_at	-1.6726896	down	-1.39632	down	-1.21468	down	-2.3356	down
Os.10493.1.S1_at	-1.172556	down	1.426644	up	-2.41962	down	1.216695	up
Os.9768.1.S2_at	1.8304735	up	-1.26079	down	2.137866	up	1.451852	up
Os.51078.1.S1_at	1.0104237	up	2.344633	up	-1.38273	down	2.369073	up
Os.51730.1.A1_x_at	-2.2003791	down	-1.1311	down	-1.49909	down	-2.48885	down
Os.49742.2.S1_at	1.4185929	up	1.478889	up	1.146541	up	2.097941	up
Os.16958.1.S1_at	-1.440974	down	-1.50907	down	-1.12357	down	-2.17453	down
OsAffx.23795.1.S1_at	-3.415678	down	1.35404	up	-2.29544	down	-2.52258	down
Os.10274.3.S1_at	1.341913	up	-2.16206	down	1.275489	up	-1.61117	down
Os.32455.1.S1_at	2.4771786	up	3.266522	up	-1.92754	down	8.09176	up
Os.1107.2.S1_s_at	-1.9129246	down	-1.22621	down	-1.3818	down	-2.34564	down
OsAffx.17884.1.S1_s_	-1.6161766	down	-1.6192	down	-1.04635	down	-2.61691	down
Os.9005.1.S1_s_at	-1.9240038	down	-1.16153	down	-1.45842	down	-2.23479	down
Os.38157.1.S1_at	1.876099	up	-1.68934	down	2.861603	up	1.110555	up
Os.57379.1.S1_at	-2.4828517	down	1.215624	up	-2.05909	down	-2.04245	down
Os.27536.1.A1_at	-2.0378325	down	1.446935	up	-2.4506	down	-1.40838	down
Os.11258.1.S1_at	-2.015834	down	-1.27712	down	-1.32581	down	-2.57447	down
Os.14216.1.S1_at	-1.6849815	down	-1.45039	down	-1.1674	down	-2.44387	down
Os.7315.2.S1_x_at	-1.74694	down	-1.66783	down	-1.01506	down	-2.9136	down
Os.4760.1.S1_at	-1.0289503	down	-1.38555	down	2.345624	up	-1.42566	down
Os.46933.1.S1_at	-1.8663777	down	1.369999	up	-2.31921	down	-1.36232	down
Os.34372.1.S1_at	7.8918686	up	-18.4845	down	31.28925	up	-2.34222	down
Os.46711.1.S1_at	3.7979832	up	1.317326	up	1.284594	up	5.003182	up
OsAffx.13565.1.S1_at	1.287399	up	2.224217	up	-1.31454	down	2.863455	up
Os.18927.1.S1_at	-1.7707114	down	1.802129	up	-3.04919	down	1.017743	up
Os.55342.1.S1_at	-1.5889693	down	-1.76589	down	1.043722	up	-2.80595	down
Os.37184.1.S1_at	-2.8509197	down	-1.66548	down	-1.01583	down	-4.74814	down
Os.51751.1.S1_at	-1.5567714	down	-2.03978	down	1.205886	up	-3.17547	down
Os.7530.1.S1_at	2.9076784	up	1.055365	up	1.602698	up	3.068662	up
Os.30598.1.S1_at	-1.3164719	down	2.383638	up	-1.40937	down	1.810626	up
Os.46535.1.S1_at	-2.2842853	down	2.318021	up	-1.37092	down	1.014769	up
Os.51305.1.S1_at	-1.6500276	down	-1.61099	down	-1.0494	down	-2.65818	down
Os.27528.1.S1_x_at	-1.9207501	down	1.435939	up	-2.42744	down	-1.33763	down
Os.33851.2.S1_x_at	-2.2992337	down	-1.10798	down	-1.52557	down	-2.5475	down
Os.32488.1.S1_at	1.6315216	up	1.986234	up	-1.17514	down	3.240583	up
Os.9155.1.S1_at	-1.1743557	down	1.328287	up	-2.24507	down	1.131077	up
Os.8914.1.S1_at	1.2944788	up	1.92918	up	-1.14148	down	2.497282	up
Os.36472.2.S1_at	-1.7374935	down	-1.00718	down	1.702078	up	-1.74996	down
Os.25097.1.S1_at	1.1993207	up	2.062381	up	-1.2208	down	2.473456	up
Os.54258.1.S1_at	-3.1280885	down	1.197772	up	-2.02326	down	-2.61159	down
Os.5247.1.S1_at	1.5233399	up	-1.40765	down	2.377547	up	1.082187	up
Os.52157.1.S1_x_at	-2.1107845	down	2.087068	up	-3.52492	down	-1.01136	down
Os.12383.1.S1_at	-2.2562857	down	-1.03344	down	-1.63372	down	-2.33173	down

Os.18530.1.S1_at	-1.8675582	down	-1.47637	down	-1.14345	down	-2.7572	down
Os.48024.1.A1_x_at	4.5192246	up	-1.4171	down	2.39183	up	3.18906	up
Os.49909.1.S1_at	-2.1910844	down	1.340708	up	-2.26266	down	-1.63427	down
Os.7030.1.S1_s_at	-1.2461436	down	-2.04013	down	1.208865	up	-2.5423	down
OsAffx.31420.1.S1_at	1.7192067	up	2.063351	up	-1.22277	down	3.547328	up
Os.49591.1.S1_at	1.1283351	up	3.681299	up	-2.18169	down	4.153738	up
Os.15087.2.S1_at	2.2224722	up	1.026572	up	1.643539	up	2.281529	up
Os.23086.2.S1_x_at	1.3140603	up	-1.36993	down	2.311261	up	-1.04252	down
Os.54454.1.S1_at	3.1940217	up	1.410352	up	1.196246	up	4.504694	up
Os.19186.1.S1_at	-3.8873303	down	-1.12008	down	-1.50589	down	-4.35412	down
Os.51066.1.S1_at	1.7621665	up	1.682995	up	1.002126	up	2.965717	up
Os.27634.1.S1_at	-2.9159396	down	1.237847	up	-2.08731	down	-2.35565	down
Os.56163.1.S1_at	-3.186622	down	-1.00309	down	-1.68088	down	-3.19647	down
Os.8136.1.A1_at	-2.593566	down	-1.06892	down	-1.57688	down	-2.77232	down
Os.18724.2.S1_x_at	1.4863293	up	3.07547	up	-1.82481	down	4.571161	up
Os.5297.1.S1_at	1.1218965	up	2.604993	up	-1.54574	down	2.922533	up
Os.10522.2.S1_x_at	1.1550508	up	2.399137	up	-1.42361	down	2.771125	up
Os.19114.1.S1_at	-1.465826	down	1.670821	up	-2.8147	down	1.139849	up
Os.22651.1.S1_at	1.0245922	up	-1.53633	down	2.588054	up	-1.49946	down
Os.18609.1.S1_at	2.6903834	up	1.245058	up	1.352851	up	3.349685	up
Os.38157.1.S1_s_at	2.676303	up	-2.22109	down	3.741116	up	1.204949	up
Os.51798.1.S1_at	-2.0021532	down	-1.1466	down	-1.46885	down	-2.29567	down
Os.56121.1.S1_x_at	1.4907855	up	-1.43434	down	2.415427	up	1.039351	up
Os.10522.2.S1_at	1.1505008	up	2.425788	up	-1.44061	down	2.79087	up
Os.24872.1.S1_at	-1.7750717	down	-1.18549	down	-1.42021	down	-2.10433	down
OsAffx.32299.1.A1_at	-1.0960455	down	-1.83526	down	1.090144	up	-2.01153	down
Os.46767.1.S1_at	1.9631555	up	1.167136	up	1.442212	up	2.29127	up
OsAffx.19285.1.S1_at	2.0953357	up	1.677308	up	1.003515	up	3.514525	up
Os.30657.1.S1_at	3.888007	up	-3.08329	down	5.188138	up	1.260994	up
OsAffx.26652.1.S1_at	-2.683618	down	-1.05136	down	-1.60044	down	-2.82145	down
Os.26475.1.S1_at	-1.8076907	down	1.282863	up	-2.15815	down	-1.40911	down
Os.51718.1.S1_at	6.487113	up	-7.00066	down	11.77584	up	-1.07916	down
Os.13245.1.S1_x_at	-2.1889257	down	-1.20266	down	-1.39841	down	-2.63254	down
OsAffx.3183.1.S1_at	-1.1525548	down	-1.86435	down	1.108657	up	-2.14876	down
Os.11301.1.S1_at	1.5328343	up	-2.86304	down	1.702711	up	-1.86781	down
Os.8636.1.S1_at	-1.9862747	down	-1.17637	down	-1.42935	down	-2.33659	down
Os.52179.1.S1_at	-2.1914496	down	-1.32461	down	-1.26923	down	-2.90281	down
OsAffx.30715.2.S1_at	-1.9204546	down	-1.26468	down	-1.32937	down	-2.42876	down
Os.56875.1.S1_at	2.6027455	up	-10.26	down	17.24599	up	-3.942	down
Os.52561.1.S1_x_at	-1.5847625	down	1.35703	up	-2.28098	down	-1.16782	down
Os.15041.1.S1_a_at	-2.1649601	down	1.187483	up	-1.9957	down	-1.82315	down
Os.7573.1.S1_at	-1.494822	down	-1.36975	down	-1.22695	down	-2.04753	down
Os.5659.3.S1_x_at	-2.5098326	down	1.201804	up	-2.01949	down	-2.08839	down
Os.3448.1.S1_a_at	-1.4095347	down	-1.42917	down	-1.17575	down	-2.01447	down
Os.21399.1.S1_a_at	-1.8455948	down	-1.15902	down	-1.44968	down	-2.13908	down
Os.1438.1.S1_at	2.4929192	up	1.356015	up	1.23873	up	3.380437	up
Os.53707.1.S1_x_at	-2.8521771	down	2.376975	up	-3.99231	down	-1.19992	down

Os.52656.1.S1_at	-2.3580322	down	1.060235	up	-1.78074	down	-2.22407	down
Os.22673.1.S1_at	-1.8870441	down	-1.42833	down	-1.17556	down	-2.69532	down
Os.32659.1.S1_at	2.457079	up	-1.80502	down	3.03067	up	1.361245	up
Os.16012.1.S1_at	-1.8302416	down	1.618552	up	-2.71744	down	-1.13079	down
Os.54926.1.S1_at	-3.1545916	down	-1.20064	down	-1.39819	down	-3.78753	down
Os.21423.1.S1_at	-2.1377344	down	-1.13743	down	-1.47572	down	-2.43152	down
OsAffx.4103.1.S1_s_a	2.0735412	up	1.250904	up	1.341719	up	2.593801	up
OsAffx.32235.1.S1_x_	-1.9350774	down	1.204778	up	-2.02202	down	-1.60617	down
Os.34343.1.S1_a_at	-2.318024	down	-1.12874	down	-1.48683	down	-2.61644	down
OsAffx.15140.1.S1_at	1.8060725	up	2.030687	up	-1.21001	down	3.667568	up
Os.49477.1.S1_at	-1.7874035	down	-1.25276	down	-1.33959	down	-2.23918	down
OsAffx.19795.1.S1_s_	-2.8116844	down	1.143238	up	-1.91854	down	-2.4594	down
Os.22278.1.S1_at	3.7224681	up	-1.48005	down	2.483438	up	2.5151	up
Os.35257.1.S1_at	-2.0448897	down	1.166759	up	-1.95748	down	-1.75262	down
Os.47461.1.S1_at	-1.8934062	down	1.304616	up	-2.1878	down	-1.45131	down
Os.9883.1.A1_at	-2.1892617	down	1.020392	up	-1.71101	down	-2.14551	down
Os.15421.1.S1_at	-1.4244183	down	-1.6493	down	-1.01645	down	-2.34929	down
Os.7507.1.S1_at	1.1651376	up	-1.28333	down	2.151021	up	-1.10144	down
Os.16908.1.S1_at	-1.3991421	down	-1.45862	down	-1.1491	down	-2.04082	down
Os.23434.1.S1_s_at	-1.7818974	down	-1.12566	down	-1.48898	down	-2.00582	down
Os.38351.1.A1_at	-1.4033946	down	-1.62506	down	-1.03123	down	-2.2806	down
Os.18550.1.S1_at	-1.709521	down	1.260462	up	-2.11211	down	-1.35627	down
Os.50221.1.S1_at	-2.0355163	down	1.136957	up	-1.9051	down	-1.79032	down
Os.15701.1.S1_x_at	-1.3672246	down	3.455473	up	-2.06226	down	2.527363	up
Os.25518.1.S1_at	-1.9177842	down	-1.05858	down	-1.58278	down	-2.03013	down
Os.21040.1.S1_at	-1.9670975	down	-1.03152	down	-1.62376	down	-2.02909	down
Os.17566.1.S1_at	-1.5923451	down	1.605662	up	-2.68862	down	1.008363	up
Os.46591.1.S1_at	-2.1256573	down	1.256172	up	-2.10312	down	-1.69217	down
Os.10861.1.S1_at	1.9210777	up	1.09801	up	1.524709	up	2.109362	up
OsAffx.24709.1.S1_at	1.6134714	up	1.346251	up	1.242564	up	2.172138	up
OsAffx.26953.1.S1_at	-2.339594	down	1.666529	up	1.003412	up	-1.40387	down
Os.17320.1.S2_at	-1.9608929	down	-1.04225	down	-1.60432	down	-2.04374	down
Os.28055.2.S1_at	-1.9391619	down	-1.04165	down	-1.60523	down	-2.01992	down
OsAffx.6495.1.S1_s_a	-3.4353032	down	-1.05738	down	-1.58111	down	-3.63243	down
Os.45903.1.S1_at	-1.8051975	down	-1.2022	down	-1.39049	down	-2.17022	down
Os.24077.1.S1_at	-1.4986454	down	-1.64116	down	-1.01844	down	-2.45951	down
Os.3141.1.S1_at	-2.0865848	down	1.026391	up	-1.7155	down	-2.03293	down
Os.7514.1.S1_at	-1.3689891	down	-1.51031	down	-1.10663	down	-2.0676	down
Os.8862.1.S1_at	-1.8439904	down	1.209165	up	-2.02092	down	-1.52501	down
Os.12784.1.S1_at	1.4080548	up	-1.31404	down	2.196133	up	1.07155	up
Os.54933.1.S1_at	3.4022455	up	1.435219	up	1.164273	up	4.882968	up
Os.31716.2.A1_at	-2.7464666	down	1.062757	up	-1.77552	down	-2.58429	down
Os.50540.1.S1_at	-1.4760119	down	1.170226	up	1.427633	up	-1.2613	down
Os.2362.2.S1_x_at	-1.3311443	down	-1.27138	down	2.123981	up	-1.69239	down
Os.11935.1.S1_at	2.9095433	up	-1.35947	down	2.270821	up	2.140206	up
OsAffx.27542.1.S1_s_	1.6380439	up	-2.59749	down	1.55511	up	-1.58572	down
OsAffx.31826.1.S1_x_	1.455647	up	-1.46874	down	2.453172	up	-1.009	down

Os.282.1.S1_a_at	-1.026828	down	1.40424	up	-2.34316	down	1.367551	up
OsAffx.28216.1.S1_at	2.6503642	up	1.548316	up	1.077069	up	4.103602	up
OsAffx.12759.1.S1_s_	-1.8383237	down	-1.09657	down	-1.52047	down	-2.01586	down
Os.54450.1.S1_s_at	1.5118691	up	-1.39896	down	2.332388	up	1.080707	up
Os.54965.1.S1_at	2.1903439	up	1.13283	up	1.471661	up	2.481287	up
Os.49161.1.S1_s_at	-2.3207512	down	-1.04292	down	-1.59851	down	-2.42035	down
Os.18354.1.S1_at	-1.8671871	down	-1.28083	down	-1.30153	down	-2.39155	down
Os.18293.1.S1_at	-1.455301	down	-1.41843	down	-1.17523	down	-2.06424	down
Os.11421.1.S1_at	2.2564852	up	-3.21787	down	5.36266	up	-1.42605	down
Os.5053.1.S1_at	1.0287302	up	-1.24744	down	2.078748	up	-1.2126	down
Os.17144.1.A1_at	-1.8574902	down	-1.23433	down	-1.34943	down	-2.29275	down
Os.27279.1.A1_at	2.6985471	up	-1.13229	down	1.885945	up	2.383256	up
Os.44431.1.S1_at	-1.1635151	down	2.786832	up	-1.67341	down	2.395183	up
Os.20028.1.S1_at	-1.9410248	down	-1.03288	down	-1.61228	down	-2.00485	down
Os.8160.1.S1_at	-1.6765953	down	-1.4191	down	-1.17337	down	-2.37925	down
Os.12145.1.S1_at	-5.1700277	down	2.496754	up	-4.15706	down	-2.0707	down
Os.27262.1.S1_a_at	1.5934564	up	1.326174	up	1.255398	up	2.113201	up
Os.56954.1.S1_at	1.575301	up	1.826503	up	-1.0971	down	2.877292	up
OsAffx.25620.1.S1_x_	-2.2544644	down	-1.0762	down	-1.54682	down	-2.42627	down
Os.10082.1.S1_at	2.192939	up	-1.04554	down	1.740083	up	2.09742	up
Os.46909.1.S1_at	-1.346686	down	-1.53191	down	-1.08626	down	-2.063	down
Os.7813.1.S1_at	-2.373728	down	-1.07135	down	-1.55307	down	-2.54309	down
Os.8773.2.S1_a_at	1.979961	up	-1.33892	down	2.227565	up	1.478778	up
Os.52263.1.S1_at	1.1877016	up	1.76878	up	-1.06328	down	2.100783	up
Os.8802.1.S1_at	2.7559762	up	-1.25865	down	2.093516	up	2.189626	up
Os.5031.1.S1_at	1.9297067	up	-1.29551	down	2.154794	up	1.489533	up
OsAffx.15880.1.S1_s_	-2.8063927	down	1.222207	up	-2.03266	down	-2.29617	down
Os.5299.2.S1_a_at	2.5018485	up	-1.62568	down	2.702989	up	1.538957	up
Os.46778.1.S1_at	-1.9567448	down	-1.05208	down	-1.58029	down	-2.05865	down
Os.1977.3.S1_x_at	-2.7308397	down	1.476953	up	-2.45514	down	-1.84897	down
Os.10178.2.S1_a_at	1.2418414	up	2.544983	up	-1.53111	down	3.160466	up
Os.27385.2.S1_x_at	-1.6902454	down	-1.24241	down	-1.3374	down	-2.09997	down
OsAffx.26585.1.S1_s_	-2.0895014	down	1.375773	up	-2.28595	down	-1.51878	down
Os.27760.2.S1_at	-2.3870716	down	-1.07493	down	-1.54568	down	-2.56593	down
Os.7289.1.S1_at	-1.958404	down	-1.14307	down	-1.45343	down	-2.2386	down
Os.17869.1.S1_at	-1.3293651	down	-1.71463	down	1.032344	up	-2.27937	down
Os.47763.1.A1_s_at	-2.4576893	down	1.326923	up	-2.20368	down	-1.85217	down
Os.12393.2.S1_at	1.3422923	up	1.914898	up	-1.15317	down	2.570353	up
Os.18745.1.S1_at	1.509374	up	1.894397	up	-1.14092	down	2.859353	up
Os.4387.1.S1_at	1.3439819	up	1.5666	up	1.059629	up	2.105482	up
Os.11353.1.S1_at	-1.2127118	down	1.233605	up	-2.04763	down	1.017228	up
Os.9462.2.S1_at	-2.960147	down	2.285179	up	-3.79297	down	-1.29537	down
Os.15621.1.S1_at	-1.1154855	down	-1.7044	down	2.828091	up	-1.90124	down
OsAffx.28181.1.S1_at	-2.4372087	down	-1.32084	down	-1.25527	down	-3.21916	down
Os.18272.1.S1_x_at	2.4114964	up	1.009881	up	1.641748	up	2.435324	up
Os.47314.1.S1_at	-2.6145914	down	1.309002	up	-2.16994	down	-1.99739	down
Os.8475.2.S1_at	1.0306987	up	11.67806	up	-7.04596	down	12.03656	up

Os.49780.1.S1_x_at	-1.731227	down	1.360963	up	-2.25551	down	-1.27206	down
Os.49409.1.S1_s_at	-1.935514	down	-1.14649	down	-1.44545	down	-2.21905	down
Os.32039.1.S1_at	1.786778	up	1.295949	up	1.278511	up	2.315573	up
Os.14338.1.S1_x_at	-2.2145178	down	1.021266	up	-1.69177	down	-2.1684	down
Os.12125.1.S1_at	-2.037913	down	1.122019	up	-1.85814	down	-1.81629	down
Os.4417.1.S1_at	-1.5623115	down	1.658363	up	-2.74613	down	1.06148	up
Os.18856.1.S1_at	1.4607279	up	2.568817	up	-1.55155	down	3.752343	up
OsAffx.30940.1.S1_at	1.7055943	up	1.696566	up	-1.02472	down	2.893654	up
Os.5659.1.S1_at	-1.6839125	down	-1.20408	down	-1.37491	down	-2.02757	down
Os.12434.1.S1_at	-1.7529083	down	-1.14706	down	-1.44306	down	-2.01069	down
Os.32682.1.S1_at	-2.9897814	down	1.119374	up	-1.85245	down	-2.67094	down
Os.46468.1.S1_at	-3.4252632	down	1.334123	up	-2.20782	down	-2.56743	down
Os.35606.2.S1_x_at	1.9822779	up	-2.21085	down	3.658123	up	-1.11531	down
Os.49948.1.S2_at	-2.4106207	down	1.135887	up	-1.87945	down	-2.12224	down
OsAffx.2318.2.A1_x_a	-1.7703663	down	-1.52054	down	-1.0881	down	-2.69192	down
OsAffx.24728.1.S1_at	-2.2663693	down	-1.32632	down	-1.24669	down	-3.00594	down
OsAffx.29764.1.S1_x_	1.4646802	up	1.472375	up	1.122977	up	2.156558	up
Os.8011.1.S1_at	1.7933385	up	-1.37038	down	2.265824	up	1.308647	up
Os.12393.2.S1_s_at	1.1850107	up	1.990843	up	-1.20418	down	2.35917	up
OsAffx.7682.1.S1_s_a	-1.8978971	down	-1.30098	down	-1.27069	down	-2.46912	down
Os.25168.2.A1_at	-2.684298	down	1.257643	up	-2.07836	down	-2.13439	down
Os.27100.1.A1_at	-1.9496568	down	1.315164	up	-2.17313	down	-1.48244	down
OsAffx.29964.1.S1_s_	1.728459	up	1.212044	up	1.363241	up	2.094969	up
Os.25231.1.S1_x_at	-2.1385465	down	-1.04911	down	-1.57463	down	-2.24356	down
Os.47918.1.S1_at	1.2915187	up	1.87134	up	-1.13303	down	2.416871	up
Os.3927.1.S1_x_at	2.0837386	up	1.101799	up	1.499019	up	2.295861	up
Os.5448.1.S1_at	-1.8020536	down	-1.57191	down	-1.05054	down	-2.83266	down
OsAffx.26573.1.S1_x_	-1.0662174	down	-2.22231	down	1.346009	up	-2.36947	down
Os.8974.1.S1_at	-1.7369173	down	-1.21524	down	-1.35828	down	-2.11076	down
Os.51944.1.S1_at	-1.5944208	down	-1.51428	down	-1.08985	down	-2.4144	down
Os.32819.1.S1_at	2.7806826	up	2.426883	up	-1.47058	down	6.748392	up
Os.51296.1.S1_at	-2.4667594	down	1.438519	up	-2.3736	down	-1.71479	down
Os.32537.1.S1_at	-1.9725052	down	-1.03766	down	-1.59004	down	-2.04679	down
Os.41098.1.S1_at	-1.3486327	down	-1.51618	down	-1.08804	down	-2.04477	down
Os.8018.1.S1_x_at	2.1636972	up	2.220156	up	-1.34613	down	4.803745	up
Os.26775.1.A1_at	-1.7153175	down	1.506655	up	-2.48452	down	-1.13849	down
Os.55314.1.S1_at	1.8573205	up	2.256511	up	-1.36863	down	4.191065	up
Os.14322.1.S1_at	-1.6590462	down	-1.43572	down	-1.14823	down	-2.38193	down
Os.25621.2.S1_at	1.2847577	up	-2.26805	down	1.376397	up	-1.76535	down
Os.8233.1.S1_s_at	1.341809	up	2.028049	up	-1.23078	down	2.721254	up
Os.15633.1.S2_at	1.7461379	up	1.259672	up	1.307992	up	2.19956	up
Os.20476.1.A1_at	-2.4961126	down	1.385392	up	-2.28219	down	-1.80174	down
Os.1577.2.S1_x_at	1.4999444	up	1.988182	up	-1.20703	down	2.982162	up
Os.52547.2.S1_at	-1.5733633	down	-1.27563	down	-1.29123	down	-2.00703	down
Os.11622.1.S1_at	-1.4915142	down	-1.69871	down	1.031348	up	-2.53365	down
Os.26870.2.A1_x_at	2.3803568	up	1.398815	up	1.177374	up	3.32968	up
Os.24780.1.A1_a_at	-2.2884433	down	-1.02185	down	-1.61161	down	-2.33845	down

Os.46897.1.S1_a_at	-1.7432002	down	-1.6419	down	-1.00276	down	-2.86216	down
Os.25027.3.S1_x_at	1.7791686	up	-1.83209	down	3.01585	up	-1.02975	down
Os.21957.1.S1_x_at	-2.0332642	down	1.728146	up	-2.84472	down	-1.17656	down
Os.51367.2.S1_x_at	-2.4818177	down	1.116934	up	-1.83857	down	-2.22199	down
Os.34343.1.S2_at	-1.6030768	down	-1.36457	down	-1.20616	down	-2.18751	down
Os.46870.1.S1_at	-1.164944	down	1.284847	up	-2.1147	down	1.102926	up
Os.13910.1.S1_at	1.3393213	up	1.918055	up	-1.16558	down	2.568891	up
Os.51829.1.S1_x_at	-2.973412	down	1.274903	up	-2.09758	down	-2.33226	down
Os.12108.1.S1_at	1.26078	up	2.446406	up	-1.487	down	3.084379	up
Os.27299.1.A1_at	-3.140524	down	3.399087	up	-5.5913	down	1.082331	up
Os.5180.1.S1_at	-1.7393032	down	-1.18359	down	-1.38935	down	-2.05862	down
OsAffx.30160.1.S1_at	3.2361016	up	-1.4026	down	2.306233	up	2.307215	up
Os.31191.2.S1_x_at	2.1190882	up	-1.19093	down	1.958192	up	1.779351	up
Os.5409.2.S1_at	-2.7697368	down	1.296252	up	-2.13134	down	-2.13673	down
OsAffx.14373.1.S1_s_	1.335762	up	1.881465	up	-1.14431	down	2.51319	up
Os.7663.1.S1_at	-1.5278696	down	1.129174	up	1.455718	up	-1.35309	down
Os.14848.1.S1_at	2.0611734	up	1.9547	up	-1.18917	down	4.028975	up
Os.14644.1.S1_a_at	-1.2867608	down	-1.6094	down	-1.02115	down	-2.07091	down
Os.15349.1.S1_a_at	2.026093	up	-1.91177	down	1.163432	up	1.059797	up
Os.15137.1.S1_at	-1.8428262	down	-1.08877	down	-1.50918	down	-2.00642	down
Os.47314.1.S2_at	-3.1681285	down	1.236084	up	-2.03104	down	-2.56304	down
Os.5736.2.S1_x_at	3.382777	up	-1.24851	down	2.051176	up	2.709445	up
Os.7912.1.S1_at	-1.1503116	down	2.426845	up	-1.47734	down	2.109729	up
Os.15984.1.S1_at	-1.6534355	down	-1.43489	down	-1.14467	down	-2.37249	down
Os.49091.1.S1_at	1.1844902	up	2.065725	up	-1.25785	down	2.446831	up
Os.47110.1.S1_at	-2.2099664	down	-1.03682	down	-1.58343	down	-2.29135	down
Os.10583.1.S1_at	1.2905015	up	1.028516	up	-1.68802	down	1.327302	up
Os.38856.1.A1_s_at	-1.8926629	down	1.304309	up	-2.14029	down	-1.45109	down
Os.9655.1.S1_s_at	-1.9288087	down	-1.2107	down	-1.35514	down	-2.3352	down
Os.52437.1.S1_at	-2.104957	down	1.100645	up	-1.80566	down	-1.91248	down
Os.57028.1.S1_at	-1.6624469	down	-1.21472	down	-1.35053	down	-2.0194	down
Os.45892.1.S1_at	2.2944982	up	1.30378	up	1.258026	up	2.991521	up
Os.46107.1.S2_at	-2.644048	down	1.490226	up	-2.44413	down	-1.77426	down
Os.9696.1.S1_at	-2.2103148	down	1.652468	up	-2.70988	down	-1.33758	down
Os.53268.1.S1_at	-1.0299428	down	3.139595	up	-1.91455	down	3.048319	up
Os.28176.2.S1_x_at	3.7933166	up	-1.13869	down	1.867	up	3.331297	up
Os.16381.1.S1_a_at	-1.2291446	down	-1.93669	down	1.181381	up	-2.38047	down
Os.13975.1.S1_at	-1.715623	down	-1.18072	down	-1.38832	down	-2.02566	down
Os.15797.1.S1_at	1.5974253	up	1.623979	up	1.009242	up	2.594185	up
Os.5212.2.S1_at	-2.4761848	down	1.414876	up	-2.31887	down	-1.75011	down
Os.32977.1.S1_x_at	-3.1842084	down	1.076782	up	-1.76462	down	-2.95715	down
Os.6220.1.S1_at	-2.4141886	down	1.079456	up	-1.76895	down	-2.23649	down
Os.49736.1.S1_at	-2.3398285	down	-1.03059	down	-1.59009	down	-2.4114	down
OsAffx.23217.1.S1_at	3.0022895	up	-1.29971	down	2.129773	up	2.309966	up
Os.27209.1.S1_at	-2.4239128	down	1.259121	up	-2.06281	down	-1.92508	down
Os.18485.1.S1_s_at	1.7325583	up	-3.89757	down	2.379272	up	-2.2496	down
Os.18205.1.S1_at	-2.4953146	down	1.048253	up	-1.71716	down	-2.38045	down

Os.32752.2.S1_s_at	2.232312	up	-1.40718	down	2.303979	up	1.586377	up
Os.35718.1.S1_at	1.5564586	up	-2.01894	down	1.2332	up	-1.29713	down
Os.17282.1.S1_at	-3.1712933	down	1.352108	up	-2.21345	down	-2.34544	down
Os.46084.1.S1_at	1.8367515	up	1.723815	up	-1.05336	down	3.16622	up
Os.52414.1.S1_at	-1.7271599	down	1.606324	up	-2.62857	down	-1.07522	down
Os.25806.1.S1_s_at	1.869934	up	-1.40161	down	2.293311	up	1.33413	up
OsAffx.3667.1.S1_at	-2.534354	down	1.763343	up	-2.88496	down	-1.43724	down
Os.20936.1.S1_a_at	-1.8039759	down	-2.19924	down	1.344251	up	-3.96738	down
Os.18493.1.S1_at	-1.0166153	down	-1.48755	down	2.433654	up	-1.51226	down
Os.10378.3.S1_s_at	1.4361242	up	1.507858	up	1.084957	up	2.165472	up
Os.22939.1.A1_at	-2.2859259	down	1.347793	up	-2.20456	down	-1.69605	down
Os.49183.1.S1_at	-2.966137	down	1.548049	up	-2.53174	down	-1.91605	down
Os.7582.1.S1_at	1.2765008	up	1.916751	up	-1.17235	down	2.446735	up
Os.10226.1.A1_at	1.1456497	up	2.158854	up	-1.32064	down	2.47329	up
OsAffx.12818.1.S1_s_	-1.607505	down	-1.46244	down	-1.11757	down	-2.35089	down
Os.31104.1.S1_at	-1.7423128	down	-1.50608	down	-1.08516	down	-2.62407	down
Os.15552.2.S1_x_at	-1.8722267	down	-1.23788	down	-1.32	down	-2.31759	down
Os.55270.1.S1_s_at	-2.2309983	down	-1.22856	down	-1.3299	down	-2.74092	down
Os.28875.1.A2_x_at	-1.7683297	down	1.388393	up	-2.26832	down	-1.27365	down
Os.8842.1.S1_at	1.3133072	up	3.444265	up	-2.10845	down	4.523378	up
Os.53319.1.S1_at	-3.131483	down	1.694495	up	-2.76793	down	-1.84803	down
Os.8111.1.S1_at	1.0625093	up	1.886261	up	-1.1551	down	2.00417	up
OsAffx.23041.1.S1_at	-1.2988869	down	1.574341	up	-2.57066	down	1.21207	up
OsAffx.23899.1.S1_at	-3.3438826	down	-1.23436	down	-1.32267	down	-4.12755	down
Os.47933.1.S1_at	1.3294699	up	2.27809	up	-1.39538	down	3.028651	up
Os.17886.1.S1_at	1.4165161	up	1.43855	up	1.134875	up	2.03773	up
Os.4652.1.S1_at	-2.7673662	down	1.045889	up	-1.70737	down	-2.64595	down
Os.7288.1.S1_at	-2.0145257	down	-1.15715	down	-1.41052	down	-2.3311	down
Os.52691.1.S1_at	-2.2855039	down	1.131397	up	-1.84589	down	-2.02007	down
Os.24651.1.S1_at	-1.115354	down	-1.90816	down	1.169887	up	-2.12827	down
Os.11117.1.S1_at	1.8170751	up	-1.28506	down	2.095918	up	1.414001	up
Os.30597.3.S1_x_at	3.4361742	up	-1.10354	down	1.799805	up	3.113761	up
Os.7686.1.S1_at	-2.1443753	down	1.053204	up	-1.71768	down	-2.03605	down
Os.49863.1.S1_at	1.6972164	up	1.450937	up	1.124011	up	2.462554	up
Os.57436.1.S1_x_at	-1.9220104	down	-1.33967	down	-1.21735	down	-2.57487	down
Os.5941.1.S1_at	-2.034119	down	1.083402	up	-1.76673	down	-1.87753	down
Os.50914.1.S1_at	-2.0876465	down	-1.00451	down	-1.62322	down	-2.09706	down
OsAffx.30088.1.S1_at	-2.1920273	down	-1.0419	down	-1.56476	down	-2.28387	down
Os.3399.1.S1_at	-2.0008533	down	1.002777	up	-1.63474	down	-1.99531	down
OsAffx.25579.2.S1_x_	-1.2841682	down	2.127957	up	-1.3054	down	1.65707	up
OsAffx.24667.1.S1_at	1.9929675	up	1.204311	up	1.353306	up	2.400152	up
Os.20441.1.A1_at	-2.2553222	down	-1.03698	down	-1.57136	down	-2.33872	down
Os.17108.1.S1_at	1.153401	up	2.284692	up	-1.4022	down	2.635166	up
Os.33610.3.S1_x_at	-2.5669534	down	1.140015	up	-1.85749	down	-2.25168	down
Os.32105.1.S1_at	-2.6558216	down	1.387197	up	-2.2602	down	-1.91452	down
Os.26063.1.S1_at	1.5920343	up	-13.0048	down	21.18627	up	-8.16865	down
Os.52964.1.S1_at	2.0983129	up	2.35227	up	-1.44396	down	4.935799	up

Os.5495.1.S1_at	1.9404488	up	1.346574	up	1.209166	up	2.612958	up
Os.15047.1.S1_s_at	1.4926445	up	-1.34627	down	2.191511	up	1.108723	up
Os.5560.1.S1_at	1.6161604	up	1.281053	up	1.270313	up	2.070388	up
Os.47903.1.A1_at	1.1857268	up	-1.51948	down	2.471948	up	-1.28148	down
Os.11265.1.S1_at	2.0525503	up	-2.28912	down	3.724003	up	-1.11526	down
Os.45994.1.S1_at	-2.822996	down	1.018314	up	-1.65641	down	-2.77223	down
Os.16741.1.S1_a_at	1.8058965	up	1.159949	up	1.402082	up	2.094748	up
OsAffx.26381.1.S1_at	-1.3027374	down	1.348148	up	-2.19251	down	1.034858	up
Os.8778.1.S1_a_at	1.5188733	up	1.956585	up	-1.20315	down	2.971804	up
Os.38421.1.S1_at	-1.8569286	down	1.766412	up	-2.87252	down	-1.05124	down
OsAffx.12216.1.S1_at	-3.2284544	down	1.148242	up	-1.86719	down	-2.81165	down
Os.15594.1.S1_s_at	-1.5773284	down	-1.34282	down	-1.21091	down	-2.11807	down
Os.52921.1.S1_at	-1.139894	down	1.620006	up	-2.63399	down	1.42119	up
Os.36460.1.S1_at	-2.8111308	down	1.007455	up	-1.63744	down	-2.79033	down
OsAffx.14177.1.S1_at	2.5678577	up	1.233345	up	1.317743	up	3.167054	up
Os.27247.1.S1_at	2.1036108	up	7.2349	up	-4.45276	down	15.21941	up
OsAffx.15562.1.S1_s_	1.3312801	up	-1.05384	down	-1.54176	down	1.263262	up
OsAffx.28637.1.S1_x_	-1.0014632	down	-2.39868	down	1.476395	up	-2.40219	down
Os.50903.2.S1_x_at	3.8196645	up	1.837215	up	-1.13092	down	7.017544	up
Os.27520.1.S1_at	-1.1861244	down	-1.83074	down	1.126974	up	-2.17148	down
Os.8791.1.S1_at	-2.1822774	down	1.515507	up	-2.46186	down	-1.43997	down
Os.39828.1.A1_x_at	-1.527516	down	-1.36879	down	-1.18651	down	-2.09084	down
Os.8957.1.S1_a_at	2.3685207	up	-1.42394	down	2.312219	up	1.663363	up
OsAffx.16857.1.S1_s_	2.0801373	up	1.207178	up	1.345104	up	2.511097	up
Os.10707.3.S1_x_at	-2.020803	down	1.295286	up	-2.10322	down	-1.56012	down
Os.38301.1.S1_a_at	-2.0166621	down	-1.33754	down	-1.21393	down	-2.69737	down
Os.1307.1.S1_a_at	-1.8456068	down	1.393325	up	-2.26184	down	-1.32461	down
Os.7053.1.S1_at	-1.5053716	down	-1.37079	down	-1.184	down	-2.06356	down
Os.12758.1.S1_at	-1.3136209	down	-1.53837	down	-1.05501	down	-2.02084	down
Os.20230.1.S1_at	-1.1177422	down	-3.84865	down	2.371489	up	-4.3018	down
Os.34865.1.S1_s_at	-2.2812192	down	1.144238	up	-1.85696	down	-1.99366	down
Os.27028.1.A1_at	1.595385	up	1.340341	up	1.210762	up	2.13836	up
Os.53884.1.S1_at	3.0040724	up	-1.47544	down	2.394014	up	2.036055	up
Os.17791.1.S1_at	-2.0165226	down	-1.07431	down	-1.51019	down	-2.16636	down
Os.37320.2.S1_at	-2.114981	down	1.101149	up	1.473151	up	-1.9207	down
Os.10324.1.S1_at	-1.6250588	down	-1.62789	down	1.003569	up	-2.64542	down
Os.46183.1.S1_at	-4.134549	down	1.915263	up	-3.10621	down	-2.15874	down
Os.353.1.S1_at	-1.3100606	down	1.527761	up	-2.47769	down	1.166176	up
Os.18822.1.A1_at	-1.7429167	down	-1.15426	down	-1.40499	down	-2.01178	down
Os.7756.2.S1_at	-2.7088952	down	7.071967	up	-4.36209	down	2.610647	up
Os.14176.1.S1_at	-2.1068628	down	1.164756	up	-1.88742	down	-1.80885	down
Os.20460.1.S1_at	-2.4481087	down	-1.05549	down	-1.53498	down	-2.58395	down
OsAffx.28234.1.S1_at	-2.9237955	down	1.932708	up	-3.13117	down	-1.5128	down
OsAffx.21227.1.S1_x_	-2.4662344	down	1.367836	up	-2.21593	down	-1.80302	down
Os.49466.1.S1_at	-1.5982754	down	1.370216	up	-2.21969	down	-1.16644	down
Os.24147.1.A1_at	-2.6465044	down	1.041773	up	-1.68732	down	-2.54038	down
Os.49392.2.S1_at	-3.5883968	down	1.260128	up	-2.04097	down	-2.84764	down

Os.19740.1.S2_at	4.6188693	up	-2.32571	down	3.765994	up	1.986008	up
Os.12420.1.S1_at	1.433328	up	1.831393	up	-1.1314	down	2.624986	up
OsAffx.4280.2.A1_at	-1.4595087	down	3.285966	up	-2.03044	down	2.251419	up
Os.45918.1.S1_x_at	-1.8604563	down	1.635445	up	-2.64639	down	-1.13758	down
Os.50385.1.S1_s_at	-1.4363507	down	-1.5822	down	-1.02266	down	-2.27259	down
Os.55968.1.S1_at	1.2604165	up	-2.03212	down	1.255934	up	-1.61226	down
Os.47401.1.A1_s_at	-1.4080982	down	-1.42225	down	-1.13737	down	-2.00267	down
Os.11437.1.S1_at	-1.8517329	down	-1.67818	down	1.037618	up	-3.10754	down
Os.27462.1.S1_at	-1.6611925	down	1.487178	up	-2.40516	down	-1.11701	down
Os.5201.1.S1_at	2.5402293	up	2.069673	up	-1.27986	down	5.257444	up
Os.26572.1.S1_at	-2.053881	down	1.457643	up	-2.35684	down	-1.40904	down
Os.52344.1.S1_at	-1.5045426	down	2.93735	up	-1.8167	down	1.952321	up
Os.50454.2.S1_at	-2.0727391	down	1.225461	up	-1.98124	down	-1.6914	down
Os.28539.1.S1_at	1.3584146	up	-3.84	down	6.20695	up	-2.82682	down
Os.38354.2.A1_x_at	-3.1867943	down	1.043755	up	-1.68693	down	-3.0532	down
Os.12649.1.S1_at	1.2564039	up	1.654676	up	-1.02387	down	2.078941	up
Os.55444.1.S1_at	-1.5175213	down	-1.46092	down	-1.10608	down	-2.21698	down
Os.11022.1.S1_at	-1.1464278	down	-1.89058	down	1.170013	up	-2.16741	down
OsAffx.27612.1.S1_at	19.744986	up	1.404842	up	1.1502	up	27.73859	up
Os.12737.1.S1_at	-1.5414478	down	1.330732	up	-2.15015	down	-1.15835	down
Os.10238.1.S1_at	-2.0348127	down	1.037467	up	-1.67628	down	-1.96133	down
OsAffx.26492.1.S1_at	1.9644562	up	2.329991	up	-1.44214	down	4.577165	up
Os.51158.1.S1_at	-1.6354353	down	-1.2553	down	-1.28694	down	-2.05296	down
Os.6386.1.S1_at	2.0530224	up	1.352671	up	1.194061	up	2.777063	up
Os.53670.1.S1_at	1.1174837	up	-2.97547	down	1.842266	up	-2.66265	down
OsAffx.14800.1.S1_at	-3.4334388	down	1.962823	up	-3.16917	down	-1.74924	down
Os.14160.1.S1_at	1.454234	up	3.657502	up	-2.2656	down	5.318863	up
Os.24588.1.S1_at	-2.2136178	down	1.036285	up	-1.67278	down	-2.13611	down
OsAffx.18661.1.S1_at	-1.9562274	down	-1.43153	down	-1.12747	down	-2.8004	down
Os.54104.1.S1_at	1.5731014	up	1.883659	up	-1.1671	down	2.963186	up
Os.27322.2.S1_at	-1.0761334	down	-1.23929	down	2.000088	up	-1.33364	down
Os.9772.1.S1_at	-1.1885967	down	2.049262	up	-1.26977	down	1.724102	up
Os.12643.3.S1_x_at	-2.2630804	down	1.140398	up	-1.84045	down	-1.98447	down
OsAffx.4464.1.S1_at	-2.0636587	down	-1.21396	down	-1.32933	down	-2.5052	down
Os.49531.1.S1_at	-1.5500762	down	-1.51037	down	-1.06843	down	-2.34119	down
Os.53334.1.S1_x_at	-1.4761748	down	-1.41956	down	-1.13676	down	-2.09553	down
Os.9572.1.S1_at	-1.9465157	down	-1.45024	down	-1.11268	down	-2.82291	down
OsAffx.24173.1.S1_at	-2.8597155	down	1.592684	up	-2.5696	down	-1.79553	down
Os.23608.1.S1_at	-1.931357	down	-1.2038	down	-1.34005	down	-2.32497	down
Os.49518.1.S1_at	-2.051175	down	-1.12846	down	-1.42909	down	-2.31467	down
Os.24746.1.S1_at	1.078066	up	2.409714	up	-1.49429	down	2.59783	up
Os.8825.2.S1_x_at	1.6531053	up	1.818114	up	-1.12749	down	3.005534	up
Os.48068.1.S1_x_at	-1.7572752	down	-1.45438	down	-1.10864	down	-2.55574	down
Os.9067.1.S1_at	-1.0578785	down	3.634402	up	-2.25422	down	3.435557	up
Os.5567.1.S1_at	1.7767067	up	-1.30386	down	2.101967	up	1.362654	up
Os.40020.1.S1_a_at	1.4999164	up	1.96737	up	-1.22038	down	2.95089	up
Os.16889.1.S1_a_at	-2.1150753	down	-1.07179	down	-1.50409	down	-2.26691	down

Os.6224.2.S1_at	-3.490975	down	-1.43514	down	-1.12304	down	-5.01004	down
Os.11510.1.S1_s_at	2.2447214	up	-1.39712	down	2.251628	up	1.606677	up
Os.48545.1.S1_at	1.5694413	up	3.212587	up	-1.99349	down	5.041966	up
Os.57530.1.S1_x_at	-2.225406	down	-1.01622	down	-1.58579	down	-2.2615	down
Os.19363.1.S1_a_at	-2.4594252	down	1.386809	up	-2.23416	down	-1.77344	down
OsAffx.27281.1.S1_at	2.3815317	up	-1.42354	down	2.292916	up	1.672961	up
Os.7141.1.S1_at	1.2399422	up	2.016816	up	-1.25227	down	2.500736	up
OsAffx.3506.1.S1_at	-2.1630573	down	-1.35968	down	-1.18446	down	-2.94108	down
Os.10433.1.S1_at	-2.3046987	down	1.193605	up	-1.92177	down	-1.93087	down
Os.11860.1.S1_at	-1.2204378	down	-2.25363	down	1.399792	up	-2.75042	down
Os.7571.1.S1_at	-1.6187309	down	-1.28889	down	-1.24905	down	-2.08637	down
Os.9104.1.S1_at	2.1276593	up	-1.13904	down	1.833726	up	1.867936	up
Os.39031.1.S1_at	1.8628291	up	-2.49513	down	1.550172	up	-1.33943	down
Os.27211.1.S1_at	-3.5813906	down	1.310086	up	-2.1086	down	-2.73371	down
Os.10616.2.S1_x_at	1.6272388	up	1.371457	up	1.173571	up	2.231688	up
Os.24883.1.S1_s_at	-1.7936049	down	-1.14622	down	-1.40418	down	-2.05586	down
Os.13708.1.S1_at	1.8869663	up	3.193771	up	-1.98451	down	6.026538	up
Os.40398.1.S1_s_at	1.7840664	up	1.321284	up	1.217957	up	2.357258	up
Os.2881.1.S1_at	1.0195144	up	1.347186	up	-2.16796	down	1.373475	up
Os.27961.1.S1_at	2.0386398	up	2.637423	up	-1.63894	down	5.376756	up
OsAffx.31655.2.S1_at	1.9229171	up	1.130386	up	1.423585	up	2.173638	up
OsAffx.17942.1.S1_at	3.04386	up	1.698157	up	-1.05554	down	5.168952	up
OsAffx.31583.1.S1_at	-1.9540737	down	-1.42511	down	-1.12875	down	-2.78478	down
Os.8261.2.S1_at	-1.3015348	down	-1.81651	down	1.129282	up	-2.36425	down
Os.9456.1.S1_at	2.389967	up	-1.04074	down	1.674051	up	2.296418	up
Os.10168.1.S1_at	1.2884804	up	-1.56295	down	2.513699	up	-1.21302	down
OsAffx.6161.1.S1_x_a	-1.304373	down	-1.56196	down	-1.02962	down	-2.03738	down
Os.50241.1.S1_at	-1.8451632	down	-1.17108	down	-1.37301	down	-2.16083	down
Os.14205.1.S1_at	1.5694904	up	-1.26142	down	2.028035	up	1.244224	up
Os.25411.1.A1_at	2.608674	up	1.056749	up	1.521387	up	2.756713	up
OsAffx.23375.1.S1_at	1.6862783	up	-2.19251	down	3.5235	up	-1.30021	down
Os.11900.1.S1_at	3.3605914	up	-1.23261	down	1.980722	up	2.726403	up
Os.54261.1.S1_at	-2.101205	down	1.032463	up	-1.65852	down	-2.03514	down
Os.27763.1.A1_at	-2.4211838	down	-1.1826	down	-1.35827	down	-2.8633	down
Os.7506.1.S1_at	-1.9699165	down	2.122881	up	-3.40989	down	1.077651	up
OsAffx.29504.1.S1_at	1.2688125	up	-1.33092	down	2.137678	up	-1.04895	down
Os.14691.1.S1_at	-1.7894223	down	-1.34024	down	-1.19826	down	-2.39825	down
Os.50508.1.S1_at	7.1184444	up	-1.2503	down	2.007909	up	5.6934	up
Os.20642.1.S1_at	2.8121889	up	-1.08555	down	1.742938	up	2.590558	up
OsAffx.20211.1.S1_at	1.1811152	up	-1.59255	down	2.556667	up	-1.34835	down
Os.53728.1.S1_at	-1.7469256	down	2.168155	up	-1.35068	down	1.241126	up
Os.25564.1.S1_at	2.1051056	up	1.510174	up	1.062835	up	3.179076	up
Os.11129.1.S1_at	1.0937951	up	2.130578	up	-1.32761	down	2.330416	up
Os.25562.1.S1_at	1.2270153	up	3.354423	up	-2.09027	down	4.115929	up
Os.49548.1.S1_at	-1.3701879	down	-1.52765	down	-1.05028	down	-2.09316	down
Os.17903.1.S1_at	-1.6248193	down	1.346664	up	-2.15984	down	-1.20655	down
Os.34312.1.S1_at	-3.191318	down	1.20801	up	-1.93656	down	-2.6418	down

Os.49598.1.S1_at	-2.8679214	down	1.143746	up	-1.83337	down	-2.50748	down
Os.27322.3.S1_x_at	-1.2058496	down	-1.09204	down	1.750386	up	-1.31683	down
OsAffx.2442.1.S1_x_a	3.065556	up	-1.11373	down	1.785039	up	2.752501	up
OsAffx.15184.1.S1_at	-1.4228244	down	1.459162	up	-2.33848	down	1.025539	up
Os.45938.1.S1_at	1.4829091	up	1.607213	up	-1.00296	down	2.383351	up
Os.16798.1.S1_at	-1.2143506	down	-1.64948	down	1.029488	up	-2.00304	down
Os.46677.1.S1_at	-2.030917	down	-1.19138	down	-1.34484	down	-2.4196	down
Os.27519.1.S1_at	-2.167561	down	1.063895	up	-1.7043	down	-2.03738	down
OsAffx.27508.131.S1_	-1.7507892	down	-1.24128	down	-1.29046	down	-2.17322	down
Os.6325.1.A1_at	-1.8353516	down	-1.24355	down	-1.28786	down	-2.28235	down
Os.53722.1.S1_at	-1.19551	down	-1.05915	down	1.696107	up	-1.26622	down
Os.12700.1.S1_at	2.7263641	up	-1.06799	down	1.710132	up	2.55281	up
Os.27195.1.S1_at	1.3374124	up	1.66495	up	-1.0398	down	2.226725	up
Os.39973.1.S1_s_at	3.6292696	up	-2.96848	down	4.752718	up	1.222604	up
Os.56959.1.S1_at	1.5901492	up	1.891779	up	-1.18164	down	3.008211	up
Os.27153.1.S1_at	-2.3687139	down	1.209527	up	-1.93642	down	-1.95838	down
Os.54604.1.S1_at	-2.2900336	down	-1.10932	down	-1.44316	down	-2.54038	down
Os.7170.2.A1_at	-1.7964604	down	1.829889	up	-2.92942	down	1.018608	up
Os.18078.1.S1_at	2.599606	up	-1.26081	down	2.017915	up	2.061849	up
Os.14554.1.S1_at	-2.0041413	down	-1.31652	down	-1.21554	down	-2.63848	down
Os.8626.2.S1_at	-2.239585	down	-1.07456	down	-1.48876	down	-2.40657	down
Os.4923.1.S1_at	2.2155707	up	1.565708	up	1.021648	up	3.468937	up
Os.17921.1.S1_at	-2.0537186	down	1.513913	up	-2.42157	down	-1.35656	down
Os.6341.1.S1_at	-1.7623123	down	1.370786	up	-2.19243	down	-1.28562	down
Os.7989.1.S1_at	1.4017024	up	1.790309	up	-1.11944	down	2.50948	up
Os.17479.1.S1_at	1.0547649	up	2.586353	up	-1.61742	down	2.727994	up
Os.8044.1.S1_x_at	-3.6902888	down	1.441235	up	-2.30437	down	-2.56051	down
Os.16453.1.S1_at	1.5527885	up	-1.60844	down	2.571016	up	-1.03584	down
Os.53917.1.S1_at	-1.5795661	down	-1.32322	down	-1.20759	down	-2.09011	down
Os.35343.1.A1_at	-1.3974831	down	2.200182	up	-1.37709	down	1.574389	up
Os.46393.1.S1_at	-1.66283	down	1.372903	up	-2.19331	down	-1.21118	down
OsAffx.12010.1.S1_at	2.1454186	up	1.001848	up	1.594261	up	2.149383	up
Os.21046.1.S1_at	1.8928989	up	1.495507	up	1.067877	up	2.830843	up
Os.23302.1.A1_at	-2.524795	down	-1.09424	down	-1.45945	down	-2.76274	down
OsAffx.4150.1.S1_x_a	-1.6005837	down	-1.52138	down	-1.04956	down	-2.43509	down
Os.51810.1.S1_at	-3.6531801	down	1.111641	up	-1.7745	down	-3.2863	down
Os.17247.1.S1_at	3.4649844	up	-1.0049	down	1.60387	up	3.448072	up
Os.8137.1.S1_at	-1.8289559	down	1.34338	up	-2.1439	down	-1.36146	down
Os.18996.1.S1_at	-2.7534106	down	-1.17011	down	-1.36365	down	-3.22179	down
Os.55408.1.S1_at	1.1674935	up	-3.09729	down	1.941246	up	-2.65294	down
Os.19577.1.S1_at	-1.5538281	down	-1.32937	down	-1.20017	down	-2.06561	down
OsAffx.12889.1.S1_s_	1.8304564	up	-1.41921	down	2.264102	up	1.289775	up
Os.50742.1.S1_x_at	-2.0845048	down	1.110705	up	-1.77182	down	-1.87674	down
Os.8558.1.S1_at	-1.0566945	down	2.807933	up	-1.76037	down	2.65728	up
Os.32252.1.S1_at	1.60567	up	1.462335	up	1.090711	up	2.348027	up
Os.27553.2.S1_at	-2.1795084	down	1.130819	up	-1.80352	down	-1.92737	down
Os.52758.1.S1_at	-1.5521057	down	-1.45093	down	-1.09918	down	-2.252	down

Os.7672.1.S1_at	1.68659	up	1.355082	up	1.176881	up	2.285467	up
Os.17435.2.S1_at	-2.458971	down	1.039048	up	-1.65686	down	-2.36656	down
Os.53456.1.S1_at	-1.249258	down	-1.64216	down	1.02984	up	-2.05148	down
OsAffx.3564.1.S1_s_a	-1.3725975	down	-1.5403	down	-1.03509	down	-2.11421	down
Os.52869.1.S1_at	-3.947863	down	1.073306	up	-1.71107	down	-3.67823	down
Os.11301.1.S1_x_at	1.4405762	up	-2.41071	down	1.512262	up	-1.67344	down
OsAffx.18556.1.S1_at	-2.2291124	down	1.062625	up	-1.69379	down	-2.09774	down
Os.50476.1.S1_at	-2.121371	down	1.476534	up	-2.35284	down	-1.43672	down
Os.52435.1.S1_at	-3.5307639	down	2.275328	up	-3.62504	down	-1.55176	down
OsAffx.23999.1.S1_x_	4.2103477	up	1.158069	up	1.375516	up	4.875874	up
Os.51649.1.S1_at	2.042273	up	-1.41429	down	2.25281	up	1.444027	up
Os.7657.1.S1_at	1.6217471	up	1.468042	up	1.084921	up	2.380792	up
Os.22718.1.S1_at	2.0240355	up	1.235172	up	1.289074	up	2.500032	up
Os.27593.1.S1_at	-1.86252	down	-1.1657	down	-1.36574	down	-2.17113	down
Os.189.1.S1_at	1.533515	up	2.029888	up	-1.27517	down	3.112863	up
Os.5441.1.S1_at	-4.041402	down	-1.18122	down	-1.34756	down	-4.77378	down
Os.7278.1.S1_at	-3.050032	down	1.049185	up	-1.6697	down	-2.90705	down
Os.27254.1.S1_s_at	-1.6192981	down	-1.42612	down	-1.11591	down	-2.30931	down
Os.39214.1.S1_at	-3.4721913	down	-1.57815	down	-1.00823	down	-5.47965	down
OsAffx.3097.1.S1_s_a	-2.0288813	down	1.093963	up	-1.74053	down	-1.85462	down
Os.51059.1.A1_at	-2.1015475	down	1.004695	up	-1.59833	down	-2.09173	down
Os.39653.1.S1_s_at	-1.4178053	down	-1.41735	down	-1.12238	down	-2.00952	down
Os.4995.1.S1_at	-2.335963	down	2.391035	up	-3.80362	down	1.023575	up
Os.24785.1.A1_at	-1.7333976	down	-1.17794	down	-1.35036	down	-2.04184	down
Os.10585.1.A1_x_at	-1.9679986	down	-1.13324	down	-1.40362	down	-2.23021	down
Os.23635.1.S1_at	-3.399912	down	1.074853	up	-1.70924	down	-3.16314	down
OsAffx.14656.1.S1_at	1.2188858	up	1.669039	up	-2.6537	down	2.034367	up
Os.12192.1.S1_a_at	-1.3924586	down	1.320441	up	-2.09942	down	-1.05454	down
Os.37916.1.S1_at	1.4824514	up	1.453296	up	1.094016	up	2.154441	up
Os.49443.1.S1_at	-1.3163773	down	-1.60225	down	1.0079	up	-2.10916	down
Os.9305.2.S1_x_at	-1.5921057	down	1.028777	up	1.545037	up	-1.54757	down
Os.7717.1.S1_a_at	-2.782192	down	1.055024	up	-1.67691	down	-2.63709	down
Os.27696.1.S1_at	1.3856387	up	2.927634	up	-1.84245	down	4.056642	up
Os.26592.3.S1_x_at	-1.0581093	down	1.584021	up	-2.51652	down	1.49703	up
OsAffx.23086.1.S1_s_	-2.0615933	down	1.107097	up	-1.75873	down	-1.86216	down
Os.52577.1.S1_at	1.996493	up	1.01391	up	1.566591	up	2.024264	up
OsAffx.7023.1.S1_at	-1.7292969	down	-1.22942	down	-1.29182	down	-2.12602	down
Os.12646.1.S1_at	1.5576038	up	-1.30671	down	2.074731	up	1.192	up
Os.25211.1.S1_at	-1.5678617	down	-1.33824	down	-1.18643	down	-2.09818	down
OsAffx.13276.1.S1_at	1.7563388	up	1.93631	up	-1.21965	down	3.400816	up
Os.52462.1.S1_at	-2.0891898	down	-1.01845	down	-1.55865	down	-2.12774	down
Os.7307.2.S1_s_at	-1.6559433	down	-1.25339	down	-1.26638	down	-2.07555	down
Os.14445.1.S1_at	2.3912418	up	1.237863	up	1.282232	up	2.96003	up
Os.22840.1.A1_at	1.5325513	up	4.386499	up	-2.76376	down	6.722534	up
Os.57073.1.S1_at	2.2635293	up	-1.09315	down	1.734658	up	2.070655	up
Os.54287.1.S1_at	4.9181046	up	-1.76212	down	2.795564	up	2.791017	up
Os.11462.1.S1_at	1.8269912	up	1.645	up	-1.03707	down	3.0054	up

Os.14381.1.S1_a_at	-2.9226067	down	1.097572	up	-1.74027	down	-2.66279	down
OsAffx.4518.1.S1_s_a	2.00267	up	1.186318	up	1.336486	up	2.375804	up
Os.47343.2.S1_at	-3.2013898	down	1.878223	up	-2.97771	down	-1.70448	down
Os.38354.1.S1_at	-5.0838118	down	1.455691	up	-2.30772	down	-3.49237	down
Os.51025.1.S1_at	-2.290021	down	-1.22268	down	-1.29647	down	-2.79997	down
Os.10680.1.S1_at	2.0605052	up	1.195064	up	1.32637	up	2.462435	up
OsAffx.5996.1.S1_at	-1.8497816	down	-1.37229	down	-1.15485	down	-2.53843	down
Os.27442.1.A1_at	-2.2614307	down	1.014853	up	-1.60831	down	-2.22833	down
Os.51888.1.S1_at	-1.8601406	down	-1.14375	down	-1.38518	down	-2.12753	down
Os.20902.1.A1_at	-1.537038	down	-1.5722	down	-1.00752	down	-2.41653	down
Os.13497.1.S2_at	1.382546	up	1.603111	up	-1.01227	down	2.216375	up
Os.26557.1.A1_at	1.4869491	up	1.845914	up	-1.16561	down	2.74478	up
Os.55011.1.S1_x_at	-1.9980662	down	-1.14053	down	-1.38841	down	-2.27885	down
Os.11684.1.S1_at	-2.4025457	down	1.164429	up	-1.8437	down	-2.06328	down
Os.49596.1.S1_at	-2.0301542	down	1.15848	up	-1.8342	down	-1.75243	down
Os.27404.1.A1_at	-1.991568	down	-1.14948	down	-1.37732	down	-2.28928	down
Os.49532.1.S1_s_at	1.3892114	up	1.940969	up	-1.22616	down	2.696416	up
Os.11306.1.S1_at	2.4214053	up	1.102518	up	1.435478	up	2.669643	up
Os.18724.1.S1_a_at	1.695754	up	2.701371	up	-1.70704	down	4.58086	up
Os.23469.1.S1_at	-2.0898545	down	1.067817	up	-1.68968	down	-1.95713	down
Os.37818.1.A1_at	-2.6854386	down	1.130613	up	-1.78885	down	-2.37521	down
Os.39513.1.A1_at	-3.2193222	down	1.146858	up	-1.81436	down	-2.80708	down
Os.35855.1.S1_at	-2.3711083	down	1.033805	up	-1.6345	down	-2.29357	down
Os.50239.1.S1_a_at	-2.061854	down	2.286244	up	-1.44645	down	1.108829	up
Os.52957.1.S1_at	-4.4741035	down	1.233799	up	-1.9501	down	-3.62628	down
OsAffx.23156.1.S1_at	-1.3998317	down	-1.45881	down	-1.08343	down	-2.04209	down
Os.53951.1.S1_at	1.3867595	up	2.088399	up	-1.32135	down	2.896107	up
Os.21022.1.S1_at	1.1585344	up	2.644991	up	-1.67367	down	3.064313	up
OsAffx.14090.1.S1_at	2.2934322	up	-1.57221	down	2.484501	up	1.458729	up
Os.38153.1.S1_at	-1.9331976	down	-1.12808	down	-1.40047	down	-2.1808	down
OsAffx.27235.1.S1_at	1.338547	up	1.649775	up	-1.04427	down	2.208301	up
Os.54185.1.S1_at	-2.3746924	down	1.093043	up	-1.72672	down	-2.17255	down
Os.24599.1.A1_at	-1.7279128	down	-1.50197	down	-1.05136	down	-2.59527	down
OsAffx.24508.1.S1_at	1.1432586	up	1.968944	up	-1.24688	down	2.251012	up
Os.17213.2.S1_at	-2.39226	down	-1.43836	down	-1.09761	down	-3.44093	down
Os.10111.1.S1_at	-1.4576204	down	-1.4538	down	-1.08593	down	-2.11909	down
Os.15815.2.S1_x_at	1.9446926	up	1.614945	up	-1.02295	down	3.140572	up
Os.49400.1.S2_s_at	-1.7699295	down	2.031892	up	-3.20731	down	1.148007	up
Os.8322.1.S2_at	1.1793544	up	1.769532	up	-1.12135	down	2.086905	up
Os.50143.1.S1_at	-2.2382092	down	1.00966	up	-1.59313	down	-2.2168	down
Os.34126.1.S1_at	1.3474753	up	1.862574	up	-1.18048	down	2.509772	up
Os.49501.1.A1_at	-2.2926302	down	1.507702	up	-2.37866	down	-1.52061	down
Os.25849.1.S1_x_at	-2.0239298	down	-1.19725	down	-1.31758	down	-2.42315	down
Os.35196.1.S1_at	1.5560826	up	-1.5722	down	2.480102	up	-1.01036	down
Os.46049.1.S1_at	-1.9841728	down	-1.01085	down	-1.56044	down	-2.0057	down
Os.52132.1.S2_at	-1.5095985	down	-1.44698	down	-1.09001	down	-2.18435	down
Os.23132.1.S2_at	-2.7923963	down	1.374288	up	-2.16754	down	-2.03189	down

Os.49143.1.S1_at	-2.074917	down	1.156578	up	-1.82369	down	-1.79401	down
Os.50014.1.S1_at	-1.1333628	down	1.565131	up	-2.46727	down	1.380962	up
OsAffx.32264.1.S1_x_	1.928541	up	-1.48137	down	-1.06396	down	1.301867	up
Os.6873.1.S1_at	-3.236173	down	1.023248	up	-1.61263	down	-3.16265	down
Os.9536.1.S1_at	2.0601127	up	1.610551	up	-1.02198	down	3.317916	up
OsAffx.7764.1.S1_at	-1.2315968	down	1.277517	up	-2.01273	down	1.037285	up
Os.34358.1.S1_at	1.1437054	up	1.975258	up	-1.25387	down	2.259113	up
Os.31088.1.S1_at	-1.0547918	down	-4.17292	down	2.64897	up	-4.40156	down
Os.15944.2.S1_x_at	-2.2124963	down	1.023244	up	-1.61185	down	-2.16224	down
Os.26537.2.S1_x_at	-2.9145195	down	-1.12013	down	-1.40616	down	-3.26464	down
Os.9902.1.S1_at	-1.8979404	down	1.74195	up	-2.74371	down	-1.08955	down
Os.46002.1.S1_at	-2.0927975	down	-1.34594	down	-1.17015	down	-2.81677	down
Os.11666.1.S1_at	5.4469795	up	-1.66782	down	2.626719	up	3.265925	up
Os.5051.1.S1_x_at	-2.183321	down	1.009862	up	-1.59033	down	-2.162	down
Os.17190.1.A1_s_at	-3.246955	down	1.248558	up	-1.96611	down	-2.60056	down
Os.49226.1.S1_at	-1.9547044	down	1.451704	up	-2.28597	down	-1.34649	down
OsAffx.7246.1.S1_x_a	2.3096728	up	1.615788	up	-1.02611	down	3.731943	up
Os.32590.1.S1_at	-2.2012107	down	1.404259	up	-2.21109	down	-1.56752	down
Os.35302.1.S1_at	-1.8163606	down	1.295237	up	-2.03938	down	-1.40234	down
Os.17777.1.S1_at	2.2328153	up	-1.20134	down	1.891415	up	1.858601	up
Os.15758.1.S1_a_at	-2.2157197	down	-1.20533	down	-1.30613	down	-2.67067	down
Os.54214.1.S1_at	-1.7005851	down	-1.42163	down	-1.10717	down	-2.4176	down
Os.46268.1.S1_at	-2.8765178	down	-1.06849	down	-1.47301	down	-3.07352	down
Os.36919.1.S1_at	-1.6146629	down	3.308423	up	-2.10211	down	2.048987	up
Os.51952.1.S1_x_at	1.4782082	up	1.460382	up	1.077668	up	2.158749	up
Os.6011.1.S1_at	1.5993191	up	4.890831	up	-3.10786	down	7.821999	up
Os.53755.1.S1_at	-1.2803249	down	2.231818	up	-1.41827	down	1.743165	up
Os.26447.1.A1_at	-1.2218698	down	1.642186	up	-2.58374	down	1.343995	up
Os.28576.1.S1_at	-1.5024039	down	-1.67992	down	1.067818	up	-2.52392	down
Os.18658.1.S1_at	1.580602	up	1.486572	up	1.058013	up	2.349678	up
Os.51358.1.S1_at	-1.8145125	down	-1.29087	down	-1.21828	down	-2.34231	down
Os.12938.1.S1_at	-3.1447833	down	1.055577	up	-1.65994	down	-2.97921	down
Os.18817.1.S1_at	-1.977586	down	-1.04513	down	-1.50421	down	-2.06683	down
Os.6205.1.S1_a_at	2.3749554	up	1.040105	up	1.510653	up	2.470204	up
Os.9489.1.S1_s_at	-2.5598605	down	1.429614	up	-2.24585	down	-1.79059	down
OsAffx.19366.1.S1_at	1.6380653	up	-1.45801	down	2.28947	up	1.12349	up
Os.52304.1.S1_at	-2.1591556	down	-1.36824	down	-1.1476	down	-2.95424	down
Os.12092.2.S1_a_at	-1.7635417	down	2.440753	up	-3.8319	down	1.384006	up
OsAffx.18503.1.S1_at	13.328444	up	-1.21798	down	1.911704	up	10.94303	up
OsAffx.4629.1.S1_at	-1.513793	down	-1.4408	down	-1.08924	down	-2.18108	down
OsAffx.30514.2.S1_s_	1.0712506	up	-1.7879	down	2.805787	up	-1.66899	down
Os.9144.1.S1_a_at	-2.0895984	down	-1.0681	down	-1.46922	down	-2.23189	down
Os.10096.1.S1_at	1.9027624	up	-1.44382	down	2.265732	up	1.317864	up
Os.3388.1.S1_x_at	2.1146395	up	1.496628	up	1.048321	up	3.164828	up
Os.24933.1.S1_at	-2.3709996	down	1.077515	up	-1.69049	down	-2.20043	down
Os.51764.1.S1_at	2.0824285	up	1.374643	up	1.141196	up	2.862596	up
Os.14895.1.S1_at	-2.6494596	down	-1.09619	down	-1.43098	down	-2.90432	down

Os.51339.1.S1_at	-2.037224	down	1.501438	up	-2.35518	down	-1.35685	down
Os.38770.1.S1_at	-1.4490849	down	-1.38985	down	-1.12851	down	-2.01402	down
Os.16325.1.S1_at	1.5307649	up	1.569968	up	-1.00111	down	2.403251	up
Os.17213.1.S1_at	-1.0620197	down	-1.91477	down	1.22104	up	-2.03352	down
Os.50248.2.S1_at	-2.309818	down	-1.16647	down	-1.34431	down	-2.69433	down
Os.12663.1.S1_at	-2.074739	down	1.567377	up	-2.45749	down	-1.3237	down
OsAffx.24733.1.S1_s_	1.3175837	up	1.571452	up	-1.00258	down	2.07052	up
OsAffx.8607.1.S1_at	-2.407114	down	1.733386	up	-2.71683	down	-1.38868	down
OsAffx.16658.1.S1_at	-1.8180617	down	-1.11934	down	-1.40012	down	-2.03503	down
OsAffx.15214.1.S1_x_	-2.1742663	down	1.056164	up	-1.65505	down	-2.05865	down
Os.7749.1.S1_at	2.0007331	up	-1.02676	down	1.60792	up	1.948584	up
OsAffx.20201.1.S1_x_	-1.4831592	down	1.333787	up	-2.08846	down	-1.11199	down
Os.17990.1.S1_at	-2.3185852	down	1.120002	up	-1.75282	down	-2.07016	down
Os.56230.1.S1_at	1.3142743	up	1.686291	up	-1.07768	down	2.216249	up
Os.48353.1.S1_at	1.4708872	up	1.568009	up	-1.00235	down	2.306365	up
Os.17510.1.S1_s_at	-2.9139423	down	1.354577	up	-2.11891	down	-2.15118	down
Os.11262.2.S1_x_at	1.9316127	up	1.857421	up	-1.18745	down	3.587817	up
Os.27430.1.S1_at	1.475861	up	-1.74381	down	2.72756	up	-1.18155	down
Os.10505.1.S1_a_at	-1.1628106	down	3.410351	up	-2.18052	down	2.932852	up
Os.56110.1.S1_at	-1.3933324	down	-1.54906	down	-1.00962	down	-2.15835	down
Os.33629.1.S1_a_at	-4.0161676	down	1.937709	up	-3.03006	down	-2.07264	down
Os.33534.1.S1_s_at	1.05635	up	1.899869	up	-1.21505	down	2.006926	up
Os.15688.2.S1_x_at	1.1327833	up	1.454979	up	-2.274	down	1.648176	up
OsAffx.24464.1.S1_s_	-2.5479066	down	-1.30213	down	-1.20007	down	-3.31771	down
Os.11355.1.S1_at	2.2762403	up	-1.34189	down	2.096659	up	1.696296	up
Os.7326.1.S1_at	1.6840267	up	2.196711	up	-1.40596	down	3.69932	up
Os.9301.1.S1_at	-1.6241337	down	1.475207	up	-2.3049	down	-1.10095	down
Os.55550.1.S1_at	2.3595018	up	1.042672	up	1.498474	up	2.460186	up
Os.18736.1.S1_at	1.5058062	up	4.196512	up	-2.68591	down	6.319134	up
Os.50287.1.S1_at	-1.2494689	down	3.291421	up	-2.10684	down	2.634256	up
Os.14813.1.S1_at	2.122119	up	1.030357	up	1.516154	up	2.18654	up
Os.8453.2.S1_at	-1.6620369	down	-1.32489	down	-1.17906	down	-2.20201	down
Os.46107.1.S2_a_at	-3.8365595	down	2.389824	up	-3.7318	down	-1.60537	down
Os.52173.1.S1_at	1.5486429	up	4.164713	up	-2.66708	down	6.449655	up
Os.53498.1.S1_at	-2.0999393	down	1.075066	up	-1.67872	down	-1.95331	down
Os.10023.1.S1_at	-1.8861747	down	-1.07816	down	-1.4481	down	-2.0336	down
OsAffx.12738.1.S1_x_	-1.3428857	down	1.579107	up	-2.4652	down	1.175906	up
Os.49675.1.S1_at	1.0875022	up	-1.40039	down	2.186078	up	-1.28771	down
Os.25409.1.S1_x_at	2.262508	up	-3.26082	down	2.089474	up	-1.44124	down
OsAffx.30114.1.S1_s_	-1.6288959	down	-1.26277	down	-1.2358	down	-2.05692	down
Os.9833.1.S1_at	-2.2249258	down	1.030292	up	-1.60772	down	-2.15951	down
Os.55829.1.S1_at	2.3794117	up	-1.00449	down	1.567453	up	2.368768	up
Os.5014.1.S1_at	-1.5549313	down	-1.34719	down	-1.15809	down	-2.09479	down
Os.2544.1.S1_s_at	1.360364	up	-8.36173	down	13.04535	up	-6.14668	down
Os.17293.1.S1_at	1.7669759	up	1.178933	up	1.323089	up	2.083146	up
Os.2938.1.S1_x_at	4.4873013	up	1.074043	up	1.451029	up	4.819553	up
Os.11568.1.A1_x_at	-1.8354151	down	-1.33201	down	-1.16953	down	-2.44479	down

Os.9330.4.S1_x_at	1.5031942	up	1.638045	up	-1.05155	down	2.4623	up
OsAffx.17627.1.S1_s_	-1.6772459	down	-1.27105	down	-1.22531	down	-2.13187	down
Os.51060.1.S1_at	-3.1962247	down	1.059069	up	-1.64912	down	-3.01796	down
Os.38301.1.S1_s_at	-2.199449	down	-1.05614	down	-1.47423	down	-2.32292	down
Os.4618.1.S1_at	-1.9978697	down	2.139443	up	-3.33085	down	1.070862	up
Os.52955.1.A1_at	-2.0915196	down	-1.29472	down	-1.20243	down	-2.70792	down
Os.18079.1.S1_at	2.5734122	up	1.179744	up	1.319501	up	3.035967	up
Os.10307.1.S1_at	-2.6179485	down	-1.15016	down	-1.3533	down	-3.01106	down
Os.49448.1.S1_at	1.1976142	up	-3.33798	down	5.195448	up	-2.78719	down
Os.8987.1.S1_at	1.5219636	up	-1.35099	down	2.102519	up	1.126557	up
OsAffx.12141.1.S1_at	-1.185314	down	-2.14042	down	1.375479	up	-2.53707	down
Os.7762.1.S1_at	1.2363608	up	3.387189	up	-2.17674	down	4.187787	up
Os.27129.1.S1_at	-2.067473	down	1.015717	up	-1.58054	down	-2.03548	down
Os.25488.1.S1_at	-1.7711291	down	-1.13145	down	-1.37521	down	-2.00395	down
OsAffx.30849.1.S1_at	1.3679909	up	1.909174	up	-1.22756	down	2.611732	up
Os.50841.1.S1_at	-3.1975493	down	-1.07485	down	-1.44665	down	-3.4369	down
Os.27580.1.S1_at	1.2247164	up	10.18424	up	-6.55036	down	12.47281	up
Os.16158.1.S1_at	-2.174083	down	-1.09923	down	-1.41432	down	-2.38982	down
Os.10930.1.S1_at	2.600999	up	1.535078	up	1.012735	up	3.992735	up
Os.24057.1.A1_at	-2.6854591	down	-1.08239	down	-1.43621	down	-2.9067	down
Os.10599.1.S1_x_at	-1.7980692	down	-1.14985	down	-1.35191	down	-2.06751	down
Os.54391.1.S1_at	-1.2908148	down	-1.61056	down	1.036129	up	-2.07893	down
Os.26537.1.S1_at	-3.5255303	down	-1.44269	down	-1.07707	down	-5.08624	down
Os.31151.2.S1_at	-1.1703811	down	1.304503	up	-2.02704	down	1.114597	up
Os.53353.1.S1_at	-1.2499305	down	2.481858	up	-3.85621	down	1.985597	up
Os.47758.1.S1_at	-1.8741232	down	-1.11035	down	-1.3991	down	-2.08094	down
Os.10488.1.S1_at	-1.7971115	down	-1.13351	down	-1.36987	down	-2.03705	down
Os.6667.1.S1_at	-1.7029082	down	-1.36176	down	-1.1402	down	-2.31895	down
Os.4195.1.S1_at	-1.7948186	down	-1.2632	down	-1.22909	down	-2.26722	down
Os.6999.1.S1_at	-2.2157931	down	1.013079	up	-1.57248	down	-2.18719	down
OsAffx.26423.1.S1_at	4.1756845	up	1.009627	up	1.537324	up	4.215881	up
Os.27122.1.S2_at	-2.5322447	down	1.331513	up	-2.06629	down	-1.90178	down
Os.26152.1.S1_at	1.3923246	up	2.136482	up	-1.37685	down	2.974676	up
Os.23760.2.S1_x_at	-1.7703294	down	-1.13579	down	-1.36602	down	-2.01071	down
Os.9996.1.S1_at	-2.4299335	down	1.110319	up	-1.72259	down	-2.1885	down
Os.10616.1.S1_a_at	1.5398736	up	1.310929	up	1.183454	up	2.018664	up
Os.52897.1.S1_at	-2.1467419	down	1.354215	up	-2.10079	down	-1.58523	down
Os.48474.1.S1_at	-2.3312159	down	1.852356	up	-1.19409	down	-1.25851	down
Os.57054.1.S1_at	1.7647808	up	1.388738	up	1.11692	up	2.450818	up
Os.18467.1.S1_at	-1.5002403	down	1.389259	up	-2.15376	down	-1.07989	down
Os.11662.1.S1_at	-1.343808	down	-1.85962	down	1.199728	up	-2.49897	down
Os.11882.1.S1_at	3.7638843	up	-1.80617	down	2.798539	up	2.083899	up
OsAffx.15220.1.S1_s_	1.4576144	up	1.439997	up	1.075969	up	2.09896	up
Os.5727.1.S1_at	-1.5226765	down	-1.36159	down	-1.13791	down	-2.07326	down
Os.23652.1.S1_at	-2.115399	down	-1.19688	down	-1.29446	down	-2.53187	down
Os.7115.1.S1_a_at	-1.7329115	down	2.304509	up	-1.48745	down	1.329848	up
Os.4605.1.S1_at	-2.2422407	down	-1.29227	down	-1.19889	down	-2.89758	down

Os.49732.1.S1_at	-2.267939	down	1.02939	up	-1.59478	down	-2.20319	down
Os.4642.1.A1_s_at	1.445535	up	1.390899	up	1.113819	up	2.010593	up
OsAffx.5147.1.S1_at	1.3890176	up	-2.18842	down	1.41269	up	-1.57551	down
Os.20602.2.S1_x_at	-1.7871792	down	1.363777	up	-2.1125	down	-1.31046	down
Os.52130.1.S1_at	-2.276259	down	1.659654	up	-2.57051	down	-1.37153	down
Os.12421.1.S1_at	-2.3134942	down	1.793362	up	-2.77755	down	-1.29003	down
Os.11552.1.S2_at	-1.987453	down	1.866538	up	-2.89086	down	-1.06478	down
Os.52610.1.S1_at	-2.0030901	down	-1.14002	down	-1.35846	down	-2.28356	down
Os.55804.1.S1_at	-1.3783888	down	2.308312	up	-1.49087	down	1.674645	up
Os.45960.1.S1_at	1.164321	up	-2.3796	down	1.537125	up	-2.04377	down
Os.9788.1.S1_x_at	2.5997908	up	-1.08778	down	1.683844	up	2.389988	up
Os.25479.1.S1_at	-3.2407537	down	1.384919	up	-2.14376	down	-2.34003	down
Os.55497.1.S1_at	-2.3969238	down	1.060623	up	-1.64161	down	-2.25992	down
Os.10825.1.S1_at	-1.3387043	down	-1.49667	down	-1.03392	down	-2.0036	down
Os.53123.1.S1_at	-1.7021763	down	1.645275	up	-2.54543	down	-1.03458	down
Os.8953.1.S1_at	-2.3152606	down	-1.17871	down	-1.3124	down	-2.72903	down
Os.53148.1.S1_at	1.5487753	up	1.441641	up	1.072748	up	2.232779	up
Os.6052.1.S1_at	-2.1219573	down	-1.0402	down	-1.48672	down	-2.20726	down
OsAffx.30966.4.S1_x_	-1.3833745	down	1.570061	up	-2.42728	down	1.13495	up
Os.50590.2.A1_at	-1.1789312	down	1.464697	up	-2.26399	down	1.242394	up
Os.9424.1.S1_at	1.1431887	up	1.915156	up	-1.23913	down	2.189385	up
Os.49463.1.S1_at	1.1789286	up	-1.30052	down	2.009328	up	-1.10313	down
Os.53772.1.S1_at	-2.0761783	down	1.028411	up	-1.58886	down	-2.01882	down
Os.18462.1.S1_at	1.0767263	up	-10.1509	down	6.570973	up	-9.42752	down
OsAffx.2348.1.S1_at	1.950825	up	1.440506	up	1.072359	up	2.810175	up
Os.9730.1.S1_at	-2.146622	down	1.059555	up	-1.63661	down	-2.02597	down
Os.7693.1.S1_s_at	-1.9979056	down	-1.20824	down	-1.27839	down	-2.41395	down
Os.34335.1.S1_x_at	-2.1964452	down	1.173809	up	-1.81294	down	-1.87121	down
Os.36215.1.S1_at	1.4472941	up	-2.13118	down	3.291304	up	-1.47253	down
Os.27958.1.S1_at	-1.3835373	down	1.314644	up	-2.03027	down	-1.0524	down
Os.11090.1.S1_at	1.0942376	up	1.779151	up	-2.74753	down	1.946814	up
Os.24428.1.S1_at	-3.5005393	down	1.061831	up	-1.63946	down	-3.2967	down
Os.27405.1.S1_at	-1.6438463	down	1.537858	up	-2.37436	down	-1.06892	down
Os.34865.3.S1_x_at	-2.3373902	down	1.392744	up	-2.15027	down	-1.67826	down
Os.15185.1.S1_s_at	-1.5639752	down	-1.29002	down	-1.19658	down	-2.01757	down
Os.8260.1.S1_at	-1.3242415	down	-1.722	down	2.658015	up	-2.28035	down
Os.46577.1.S1_at	1.5618702	up	1.437402	up	1.073748	up	2.245036	up
Os.47303.1.S1_s_at	1.2503637	up	-12.8432	down	19.80615	up	-10.2715	down
Os.11584.1.S1_s_at	-1.1907177	down	1.463861	up	-2.25743	down	1.229394	up
Os.17555.1.S1_at	-2.2710543	down	-1.08832	down	-1.41688	down	-2.47163	down
Os.27820.1.S1_at	1.074555	up	2.117123	up	-1.37346	down	2.274966	up
Os.53835.1.S1_at	-1.5913792	down	-1.29274	down	-1.19231	down	-2.05724	down
Os.23232.1.S1_at	-1.7869236	down	-1.19666	down	-1.288	down	-2.13834	down
Os.27196.1.S1_x_at	-1.6884259	down	1.334063	up	-2.05552	down	-1.26563	down
Os.33990.1.S1_at	1.5485233	up	-1.34427	down	2.071041	up	1.151943	up
Os.6507.1.S1_at	-1.9013975	down	-1.07922	down	-1.42751	down	-2.05202	down
Os.15941.2.S1_x_at	2.982864	up	1.385498	up	1.111879	up	4.132752	up

Os.26675.1.S1_at	-1.2977625	down	2.332593	up	-1.51438	down	1.797396	up
Os.26852.1.A1_at	3.3819046	up	2.992928	up	-1.94311	down	10.1218	up
Os.27326.1.A1_at	-2.3679543	down	1.047825	up	-1.61389	down	-2.25987	down
Os.14204.1.S1_at	-2.2230365	down	-1.08373	down	-1.42111	down	-2.40916	down
Os.44812.1.S1_at	-1.5812768	down	-1.37121	down	-1.12288	down	-2.16825	down
Os.21192.2.S1_at	-2.0373297	down	1.068235	up	-1.64396	down	-1.90719	down
Os.17766.1.S1_at	1.1730634	up	2.462279	up	-1.60069	down	2.88841	up
Os.16695.1.S1_at	-3.1277497	down	1.301588	up	-2.00213	down	-2.40303	down
Os.32748.1.S1_x_at	2.2074893	up	-2.42414	down	1.576131	up	-1.09814	down
Os.30439.1.S1_at	1.3313278	up	1.785986	up	-1.16145	down	2.377734	up
Os.23165.1.S1_at	-1.6524408	down	1.153031	up	1.333626	up	-1.43313	down
Os.53634.1.S1_at	-1.9311651	down	-1.23834	down	-1.24116	down	-2.39143	down
Os.49228.1.S1_at	-1.4675732	down	1.319418	up	-2.02752	down	-1.11229	down
Os.9207.1.S1_at	1.0050445	up	-1.96315	down	3.016388	up	-1.9533	down
Os.15671.1.A1_at	-2.3304853	down	1.152771	up	-1.77065	down	-2.02164	down
OsAffx.24904.1.S1_at	-2.2435124	down	-1.33152	down	-1.15333	down	-2.98729	down
OsAffx.27278.1.S1_x_	2.8781648	up	1.624415	up	-1.05778	down	4.675335	up
Os.51916.1.S1_at	2.3082128	up	-3.39976	down	5.220915	up	-1.4729	down
Os.52259.1.S1_at	-2.862425	down	1.041462	up	-1.59894	down	-2.74847	down
Os.10542.1.S1_at	-1.5190663	down	-1.55112	down	1.010441	up	-2.35626	down
Os.27955.1.S1_at	2.512766	up	1.508616	up	1.017372	up	3.7908	up
Os.27916.1.S1_at	-2.0757062	down	1.114713	up	-1.71081	down	-1.8621	down
Os.54108.1.S1_at	-2.5643992	down	-1.11459	down	-1.37667	down	-2.85825	down
Os.16839.1.S1_at	-2.106991	down	1.078666	up	-1.655	down	-1.95333	down
Os.10595.1.S1_at	1.530051	up	1.358331	up	1.12952	up	2.078316	up
Os.13952.1.S1_at	-1.6593194	down	-1.29728	down	-1.18267	down	-2.15261	down
Os.24493.1.A1_at	-1.6903423	down	-1.23853	down	-1.23856	down	-2.09354	down
Os.47332.1.S1_at	-2.0366445	down	1.835998	up	-2.81577	down	-1.10928	down
Os.16435.1.S1_at	1.1259714	up	-2.03297	down	1.325837	up	-1.80553	down
Os.45095.1.S1_at	-4.6671553	down	1.564678	up	-2.39883	down	-2.98282	down
Os.39966.1.A1_at	1.1249037	up	-1.38672	down	2.125602	up	-1.23275	down
Os.11118.1.S1_at	2.1467953	up	-4.3072	down	6.6019	up	-2.00634	down
OsAffx.3180.1.S1_at	-2.6442149	down	3.0061	up	-4.60758	down	1.136859	up
Os.26586.1.A1_at	-1.8826703	down	-1.11643	down	-1.37284	down	-2.10187	down
OsAffx.30401.1.S1_at	1.8806162	up	1.280222	up	1.197149	up	2.407605	up
Os.28087.1.S1_s_at	-2.532117	down	1.553621	up	-1.01376	down	-1.62982	down
OsAffx.12697.1.S1_x_	-1.8554467	down	1.369391	up	-2.09856	down	-1.35494	down
Os.49627.1.S1_at	-1.0652077	down	2.046414	up	-1.33564	down	1.921141	up
Os.22464.1.S1_at	-2.035551	down	-1.20901	down	-1.26726	down	-2.46101	down
Os.26565.3.S1_at	2.0045726	up	-3.26164	down	4.996303	up	-1.6271	down
Os.27667.1.S1_at	-1.4436878	down	-1.40246	down	-1.09213	down	-2.02471	down
Os.52647.1.S1_at	-1.952684	down	1.84847	up	-2.8312	down	-1.05638	down
Os.36162.1.S1_s_at	1.4360654	up	2.363962	up	-1.54357	down	3.394804	up
Os.18717.2.S1_at	1.432108	up	1.445356	up	1.059482	up	2.069906	up
Os.26592.2.A1_x_at	-1.0237836	down	1.386145	up	-2.12257	down	1.353944	up
Os.49657.1.S1_at	1.1765262	up	2.656102	up	-1.73458	down	3.124973	up
Os.36376.1.S1_at	-1.5386652	down	1.807391	up	-2.76742	down	1.174649	up

Os.17025.1.S1_at	1.3765947	up	1.482209	up	1.032699	up	2.040401	up
Os.10894.1.S1_at	2.082674	up	1.071257	up	1.428784	up	2.231079	up
Os.10823.1.S1_x_at	1.8636796	up	1.38191	up	1.107518	up	2.575438	up
OsAffx.28461.1.S1_at	-3.1121366	down	-1.00138	down	-1.52816	down	-3.11643	down
Os.17878.1.S1_at	-2.088259	down	-1.01585	down	-1.50627	down	-2.12135	down
Os.56938.1.S1_at	-1.3401489	down	-2.0372	down	1.331759	up	-2.73016	down
Os.5834.1.S1_at	-1.2433635	down	2.016664	up	-1.31834	down	1.621943	up
Os.17369.1.S1_a_at	-3.3676414	down	1.233471	up	-1.88677	down	-2.73022	down
Os.10908.1.S1_a_at	2.4381897	up	1.614806	up	-1.0557	down	3.937203	up
Os.27290.1.A1_at	-2.576738	down	-1.5505	down	1.013717	up	-3.99524	down
Os.20810.1.S1_at	1.768976	up	1.158478	up	1.320253	up	2.049319	up
Os.49628.1.S1_at	-1.1531974	down	2.300808	up	-1.50432	down	1.995156	up
Os.5112.1.S1_at	3.2945254	up	-1.47013	down	2.248342	up	2.240973	up
Os.52180.1.S1_at	-2.1127224	down	-1.50325	down	-1.01732	down	-3.17595	down
Os.12197.1.S1_x_at	-1.6256711	down	1.345196	up	-2.05687	down	-1.2085	down
Os.23259.2.S1_x_at	-1.9141817	down	-1.14092	down	-1.33997	down	-2.18394	down
Os.49595.1.S1_at	-1.6691747	down	-1.46308	down	-1.04487	down	-2.44214	down
Os.8422.1.S1_at	-1.215582	down	2.699044	up	-1.76557	down	2.220372	up
Os.54637.1.S1_at	-2.842449	down	1.104454	up	-1.68798	down	-2.57362	down
Os.18959.2.S1_x_at	-1.4525096	down	-1.38388	down	-1.10437	down	-2.0101	down
Os.46486.1.S1_at	-1.3525553	down	6.3867	up	-4.17937	down	4.721952	up
Os.25007.1.S2_a_at	-2.0056934	down	-1.13588	down	-1.3453	down	-2.27823	down
Os.52295.1.S1_at	-2.6528978	down	-1.00598	down	-1.51895	down	-2.66876	down
Os.52953.1.S1_at	-2.0287569	down	-1.01236	down	-1.50924	down	-2.05384	down
Os.11469.1.S1_at	2.2913928	up	7.179496	up	-4.69897	down	16.45104	up
Os.9145.1.S1_at	2.130849	up	1.143904	up	1.335115	up	2.437487	up
Os.19228.1.S1_at	2.1124887	up	1.43901	up	1.061284	up	3.039893	up
Os.34698.1.S1_x_at	-1.945198	down	1.673154	up	-2.5548	down	-1.16259	down
OsAffx.3187.1.S1_s_a	1.6626552	up	1.418624	up	1.076347	up	2.358682	up
Os.49942.1.S1_at	-1.7978448	down	-1.15218	down	-1.32522	down	-2.07143	down
Os.18094.2.S1_a_at	2.7457979	up	-1.38753	down	2.118388	up	1.978912	up
Os.37032.1.S1_a_at	-2.3879082	down	1.158576	up	-1.76873	down	-2.06107	down
Os.9473.1.S1_a_at	-2.0304575	down	1.381868	up	-2.10867	down	-1.46936	down
Os.36647.1.S1_x_at	-3.0648444	down	-1.11782	down	-1.3651	down	-3.42594	down
Os.54314.1.S1_at	-2.1395643	down	-1.19903	down	-1.27252	down	-2.56539	down
Os.54373.1.S1_at	-1.8731916	down	-1.32936	down	-1.14742	down	-2.49015	down
Os.2938.1.S1_at	4.6315546	up	1.064404	up	1.432705	up	4.929845	up
Os.55782.1.S1_at	1.8112724	up	1.241179	up	1.228489	up	2.248113	up
Os.21238.1.S1_at	-1.9719661	down	2.674164	up	-1.75385	down	1.35609	up
Os.3818.1.S1_a_at	1.9100509	up	-1.36389	down	-1.11791	down	1.400443	up
Os.17494.2.S1_at	-1.7443684	down	2.279756	up	-3.47581	down	1.306923	up
Os.17708.1.S1_at	-1.6959319	down	1.611732	up	-2.45724	down	-1.05224	down
Os.12844.1.A1_a_at	-1.8937142	down	-1.24656	down	-1.22283	down	-2.36063	down
Os.14294.1.S1_at	1.0321167	up	-9.56168	down	6.274965	up	-9.26414	down
Os.26757.1.A1_at	1.6691153	up	1.604276	up	-1.05286	down	2.677721	up
Os.50757.1.S2_at	-1.8676821	down	-1.09634	down	-1.38967	down	-2.04762	down
Os.20764.1.S1_s_at	-1.368779	down	-2.05648	down	1.34997	up	-2.81487	down

Os.6655.2.S1_at	-1.5692563	down	-1.29421	down	-1.17682	down	-2.03095	down
AFFX-Os-actin-M_s_a	2.3387792	up	-1.26217	down	1.92227	up	1.852986	up
Os.19407.1.S1_at	-1.3629982	down	2.497811	up	-1.64016	down	1.832586	up
OsAffx.13526.1.S1_x_	-1.7507544	down	-1.32022	down	-1.1534	down	-2.31138	down
Os.14935.1.S1_at	-1.0663172	down	-1.23552	down	1.880523	up	-1.31746	down
OsAffx.30513.1.S1_s_	1.2023441	up	-1.7588	down	2.676788	up	-1.46281	down
Os.40003.1.S1_x_at	-1.3182502	down	-2.6025	down	1.71028	up	-3.43074	down
Os.19896.2.S1_x_at	-2.152004	down	-1.02442	down	-1.48512	down	-2.20456	down
Os.8859.1.S1_s_at	1.6520323	up	2.019438	up	-1.32748	down	3.336177	up
Os.23257.1.A1_at	1.3770032	up	1.58439	up	-1.04152	down	2.18171	up
Os.30608.1.S1_x_at	1.4669861	up	1.554173	up	-1.02181	down	2.27995	up
OsAffx.17990.1.S1_at	-1.8006257	down	-1.35586	down	-1.12167	down	-2.4414	down
Os.9883.2.S1_at	-2.6662848	down	1.251558	up	-1.90336	down	-2.13037	down
Os.9113.1.S1_a_at	-1.0331208	down	2.155387	up	-1.41742	down	2.086287	up
Os.14539.1.S1_at	1.5010027	up	3.014157	up	-1.98243	down	4.524257	up
Os.33610.2.A1_at	-2.251402	down	-1.38671	down	-1.09629	down	-3.12205	down
Os.14666.2.S1_x_at	1.2553127	up	2.134109	up	-1.40382	down	2.678973	up
Os.8622.1.S1_at	2.398829	up	-1.41707	down	2.153928	up	1.69281	up
Os.23659.1.S1_at	-2.4061916	down	1.109335	up	-1.68608	down	-2.16904	down
OsAffx.29335.1.S1_at	1.395244	up	-2.28487	down	1.503316	up	-1.63761	down
Os.18653.1.S1_at	-2.1490235	down	1.053967	up	-1.60173	down	-2.03899	down
Os.46561.2.S1_at	-2.221954	down	1.0265	up	-1.55997	down	-2.16459	down
Os.13960.2.S1_s_at	-2.640507	down	-1.06063	down	-1.43274	down	-2.8006	down
OsAffx.25404.1.S1_at	5.353383	up	-1.09527	down	1.664348	up	4.887714	up
Os.11326.1.S1_at	-1.3395492	down	-1.56827	down	1.032095	up	-2.10077	down
OsAffx.15332.1.S1_s_	-2.06306	down	1.403423	up	-2.13232	down	-1.47002	down
Os.51136.1.S1_at	-3.1954174	down	1.189809	up	-1.80766	down	-2.68566	down
OsAffx.17362.1.S1_at	1.6758543	up	1.099735	up	-1.67068	down	1.842996	up
Os.55958.1.S1_at	-1.6299541	down	-1.73194	down	1.140178	up	-2.82299	down
Os.35175.1.S1_at	-2.746129	down	1.032871	up	-1.56887	down	-2.65873	down
Os.30691.1.S1_at	-1.5835348	down	-1.2693	down	-1.19633	down	-2.00998	down
Os.37913.1.S1_at	-2.9192462	down	1.16166	up	-1.76382	down	-2.513	down
OsAffx.9883.1.S1_x_a	-3.66672	down	2.684774	up	-1.76848	down	-1.36575	down
Os.14287.1.S1_a_at	-3.0207126	down	1.247588	up	-1.89368	down	-2.42124	down
Os.46706.1.S1_x_at	-1.493933	down	-1.41318	down	-1.07406	down	-2.1112	down
Os.27789.1.A1_at	-2.4277558	down	2.174126	up	-3.29971	down	-1.11666	down
OsAffx.16669.1.S1_at	5.179902	up	-2.20001	down	3.338899	up	2.354493	up
Os.46393.1.S1_x_at	-1.552581	down	1.613933	up	-2.44901	down	1.039516	up
OsAffx.4104.1.S1_at	-1.2240324	down	1.578647	up	-2.39544	down	1.28971	up
Os.4370.2.S1_x_at	1.5163547	up	1.135124	up	-1.72215	down	1.721251	up
Os.49085.1.S1_at	-2.3105493	down	1.286575	up	-1.95165	down	-1.79589	down
Os.47749.1.S1_at	-1.7121856	down	-1.20935	down	-1.25397	down	-2.07062	down
Os.27570.1.S1_at	-1.0574521	down	2.448311	up	-1.61461	down	2.315292	up
Os.10853.1.S1_at	1.3284199	up	-1.61554	down	2.449513	up	-1.21614	down
Os.49827.1.S1_at	-2.8120835	down	2.477443	up	-3.75589	down	-1.13508	down
Os.30386.1.S1_at	-1.102905	down	-2.22688	down	1.468904	up	-2.45604	down
Os.49736.1.S1_a_at	-1.9562849	down	-1.17923	down	-1.28537	down	-2.30691	down

Os.37396.1.A1_at	-2.2328558	down	1.011614	up	-1.53324	down	-2.20722	down
Os.49794.1.S1_at	-1.9575882	down	-1.13875	down	-1.33077	down	-2.2292	down
Os.51117.1.S1_at	-2.3607419	down	1.415781	up	1.070257	up	-1.66745	down
Os.27647.1.S1_at	-2.1284614	down	1.171978	up	-1.77558	down	-1.81613	down
Os.4717.1.S1_at	1.29168	up	-1.5623	down	2.366586	up	-1.20951	down
Os.19784.1.S1_at	1.47694	up	-1.47175	down	2.228532	up	1.003525	up
Os.52679.1.S1_at	-4.1160064	down	1.465059	up	-2.21834	down	-2.80945	down
Os.54349.1.S1_at	1.3587723	up	-1.35802	down	2.055853	up	1.000557	up
Os.30886.3.S1_x_at	2.6288135	up	-2.35523	down	1.555828	up	1.116158	up
Os.27959.1.A1_at	-2.1065063	down	-1.55388	down	1.026605	up	-3.27325	down
Os.51027.1.S1_at	-2.1584325	down	1.054796	up	-1.59652	down	-2.0463	down
Os.53905.1.S1_at	-2.3709037	down	1.723551	up	-2.60871	down	-1.37559	down
Os.6670.1.S1_at	1.7515582	up	1.381216	up	1.095773	up	2.41928	up
Os.23342.1.S1_at	-2.232566	down	1.072758	up	-1.6232	down	-2.08115	down
Os.20911.1.S1_at	-3.0624473	down	1.230116	up	-1.86129	down	-2.48956	down
Os.49996.1.S1_at	-1.6037673	down	1.376132	up	-2.08223	down	-1.16542	down
Os.27692.1.S1_at	-1.9978702	down	-1.34129	down	-1.12808	down	-2.67973	down
Os.51105.1.S1_at	-1.649863	down	1.519864	up	-2.29937	down	-1.08553	down
Os.48056.1.S1_s_at	-2.127329	down	1.140261	up	-1.72484	down	-1.86565	down
Os.24626.1.A1_at	-2.2986317	down	-1.14062	down	-1.32598	down	-2.62187	down
Os.52792.1.S1_at	-2.1569479	down	1.210916	up	-1.83143	down	-1.78125	down
Os.54384.1.S1_at	-1.9167835	down	-1.09585	down	-1.38012	down	-2.1005	down
Os.17340.1.S1_at	-2.1531978	down	1.054352	up	-1.59438	down	-2.0422	down
Os.54042.1.S1_at	-1.6785885	down	-1.32637	down	-1.13989	down	-2.22643	down
Os.23172.1.S1_x_at	-1.2368613	down	-2.87412	down	1.901055	up	-3.55489	down
Os.7203.2.S1_x_at	1.958499	up	1.370671	up	1.102756	up	2.684459	up
Os.27602.1.S1_at	1.1643459	up	2.244846	up	-1.48525	down	2.613777	up
OsAffx.11451.2.S1_x_	2.2660224	up	-1.21581	down	1.83743	up	1.86379	up
Os.8947.1.S1_at	-2.2346332	down	2.059736	up	-3.11275	down	-1.08491	down
Os.49206.1.S1_at	-2.3783529	down	1.168591	up	-1.766	down	-2.03523	down
Os.50966.1.S1_at	-2.0150037	down	-1.20365	down	-1.25549	down	-2.42537	down
AFFX-Os_Actin_M_s_	2.315973	up	-1.25374	down	1.89462	up	1.847251	up
Os.46937.1.S1_at	-1.9619135	down	-1.33903	down	-1.12801	down	-2.62707	down
Os.10434.1.S1_at	-1.9217597	down	-1.14321	down	-1.32109	down	-2.19698	down
Os.32126.1.S1_x_at	2.3260567	up	-1.37385	down	2.074617	up	1.693095	up
Os.3396.1.S1_at	1.7877688	up	1.450554	up	1.041003	up	2.593255	up
Os.11830.1.S1_at	-2.9039264	down	1.672227	up	-2.52509	down	-1.73656	down
Os.48875.1.S1_at	2.447334	up	-4.57061	down	3.027363	up	-1.86759	down
Os.50499.1.S2_at	-2.432911	down	1.058859	up	-1.59862	down	-2.29767	down
Os.46782.1.S1_at	-2.1148467	down	1.127172	up	-1.70159	down	-1.87624	down
Os.5335.1.S1_at	1.5295572	up	-2.51067	down	1.663141	up	-1.64144	down
Os.15352.1.S1_at	-1.1566314	down	-1.49182	down	2.251436	up	-1.72548	down
Os.24765.1.A1_at	-2.5209298	down	1.366835	up	-2.06278	down	-1.84436	down
Os.24034.3.S1_x_at	-1.6800976	down	1.455235	up	-2.19618	down	-1.15452	down
Os.12560.1.S1_s_at	-1.0301294	down	-1.3611	down	2.054068	up	-1.40211	down
Os.23303.1.A1_at	-2.475618	down	1.359084	up	-2.05101	down	-1.82153	down
Os.14684.1.S1_at	-2.455235	down	1.114596	up	-1.68173	down	-2.2028	down

Os.16171.1.A1_at	-1.9522599	down	-1.42083	down	-1.06189	down	-2.77382	down
Os.49734.1.A1_at	1.9288107	up	-2.25294	down	3.398724	up	-1.16805	down
Os.52764.1.S1_x_at	-1.3757265	down	-1.60871	down	1.066458	up	-2.21315	down
Os.54900.1.S1_at	-2.441691	down	1.454973	up	-2.19365	down	-1.67817	down
Os.10597.1.S1_at	-2.4867423	down	2.107695	up	-3.17714	down	-1.17984	down
Os.14313.2.S1_x_at	1.7319213	up	2.135805	up	-1.41695	down	3.699046	up
Os.9021.2.S1_at	-1.6723847	down	-1.91795	down	1.272525	up	-3.20755	down
Os.50310.1.S1_at	-2.2094204	down	1.560075	up	-2.35081	down	-1.41623	down
Os.22922.1.S1_at	-1.9267056	down	-1.5919	down	1.056484	up	-3.06712	down
Os.15556.1.S1_at	-2.2114327	down	1.000333	up	-1.50723	down	-2.2107	down
OsAffx.14458.1.S1_x_	-1.19154	down	1.585175	up	-2.3884	down	1.330358	up
Os.9622.1.S1_at	-1.9493979	down	-1.29124	down	-1.16675	down	-2.51714	down
Os.54931.1.S1_at	-1.6932724	down	-1.20551	down	-1.24967	down	-2.04126	down
OsAffx.10060.1.S1_at	-2.0252497	down	3.456513	up	-5.20718	down	1.706709	up
Os.50582.1.S1_at	2.3849795	up	2.188929	up	-1.45321	down	5.220551	up
Os.28110.4.S1_x_at	-2.018204	down	4.368647	up	-6.58005	down	2.164621	up
Os.28139.1.S1_at	1.0247544	up	-2.3068	down	1.531607	up	-2.25108	down
Os.21280.1.S1_at	1.3409643	up	7.291094	up	-4.84231	down	9.777097	up
Os.10856.1.S1_at	-2.3903372	down	1.027933	up	-1.54737	down	-2.32538	down
Os.7902.1.S1_at	1.2588904	up	1.857915	up	-1.23425	down	2.338911	up
Os.8589.1.S1_at	-1.3868532	down	2.229482	up	-1.48116	down	1.607583	up
OsAffx.11891.1.S1_at	1.1855257	up	-1.38474	down	2.084299	up	-1.16804	down
OsAffx.19120.1.S1_x_	-1.6963614	down	-1.42509	down	-1.05616	down	-2.41747	down
Os.26728.3.S1_x_at	1.3644869	up	1.676128	up	-1.11392	down	2.287055	up
Os.16563.1.S1_at	2.3661382	up	1.945562	up	-1.29301	down	4.603469	up
Os.18238.1.S1_at	-2.413404	down	1.815378	up	-1.20652	down	-1.32942	down
Os.3710.1.S1_at	1.4075332	up	2.995641	up	-1.99096	down	4.216465	up
Os.51574.1.S1_at	-1.4942777	down	2.606574	up	-1.73253	down	1.744371	up
Os.50807.1.S1_at	1.5703412	up	1.487361	up	1.011485	up	2.335663	up
Os.27122.3.S1_x_at	-2.030924	down	1.033324	up	-1.55439	down	-1.96543	down
Os.50449.1.S1_at	1.6853371	up	1.523687	up	-1.01314	down	2.567927	up
Os.6363.1.S1_at	-2.5147681	down	-1.2946	down	-1.16163	down	-3.25563	down
Os.32721.1.S1_at	-1.5304924	down	2.036674	up	-1.35489	down	1.330731	up
Os.52646.1.S1_at	2.0425384	up	1.473694	up	1.019924	up	3.010077	up
Os.52476.1.S1_at	-1.7673719	down	1.348875	up	-2.02715	down	-1.31026	down
Os.56997.1.S1_at	1.0128102	up	2.990839	up	-1.99031	down	3.029152	up
Os.32506.1.S1_at	-2.3125222	down	1.214584	up	-1.82489	down	-1.90396	down
Os.9330.1.S1_x_at	1.3573009	up	1.959929	up	-1.30451	down	2.660213	up
Os.15729.2.S1_at	-1.404418	down	4.20292	up	-2.79817	down	2.992641	up
Os.52200.1.S1_at	-2.2468984	down	1.248203	up	-1.8747	down	-1.80011	down
Os.49125.1.S1_s_at	-1.845551	down	-1.15343	down	-1.30208	down	-2.12871	down
Os.29063.1.S1_at	-1.9068209	down	1.333183	up	-2.00221	down	-1.43028	down
Os.15204.1.S1_at	-1.4913039	down	2.50137	up	-1.66583	down	1.677304	up
Os.9515.1.S1_x_at	-3.0080261	down	-1.04948	down	1.575773	up	-3.15687	down
OsAffx.11101.1.S1_s_	-1.0235733	down	-1.92277	down	2.886756	up	-1.96809	down
Os.3850.3.S1_at	-2.2542202	down	-1.06335	down	-1.41188	down	-2.39703	down
Os.36458.2.S1_x_at	1.6578833	up	1.611203	up	-1.07325	down	2.671186	up

Os.35778.1.S1_at	-1.9033303	down	-1.06622	down	-1.40793	down	-2.02938	down
Os.30568.1.S1_at	1.0886427	up	3.275544	up	-2.1824	down	3.565897	up
Os.13968.2.S1_a_at	2.501199	up	-2.41838	down	3.629444	up	1.034245	up
Os.17285.2.S1_x_at	-1.5956944	down	-1.32866	down	-1.12943	down	-2.12013	down
Os.46287.1.S1_a_at	-1.2249142	down	2.509858	up	-1.67271	down	2.049007	up
Os.32935.1.S1_at	-1.7711915	down	-1.17972	down	-1.27187	down	-2.08952	down
Os.50904.1.S1_at	1.2615606	up	1.90606	up	-1.27034	down	2.40461	up
Os.10006.2.S1_x_at	1.6500503	up	1.271117	up	1.180343	up	2.097407	up
Os.51193.1.S1_at	-1.2678473	down	1.455027	up	-2.18241	down	1.147636	up
Os.9511.1.S1_at	1.491059	up	2.933599	up	-1.95601	down	4.37417	up
Os.27337.1.S1_a_at	-2.4782856	down	1.079256	up	-1.61856	down	-2.29629	down
Os.14536.1.S1_at	-2.5960727	down	-1.03913	down	-1.44318	down	-2.69766	down
Os.4671.1.S1_a_at	1.3632976	up	-2.54942	down	1.700263	up	-1.87004	down
Os.11691.1.S1_at	-1.8121372	down	-1.47214	down	-1.0185	down	-2.66771	down
Os.10107.2.S1_x_at	-1.1875795	down	-1.72597	down	1.15132	up	-2.04973	down
OsAffx.30183.1.S1_at	-2.1544905	down	-1.03364	down	-1.45022	down	-2.22698	down
Os.37339.1.S1_at	-1.4577277	down	-1.42764	down	-1.04999	down	-2.08112	down
Os.45751.1.A1_x_at	-3.184801	down	1.169186	up	-1.75257	down	-2.72395	down
Os.8493.1.A1_at	-2.0200105	down	1.17807	up	-1.7657	down	-1.71468	down
Os.406.1.S1_a_at	1.6706452	up	1.853094	up	-1.2365	down	3.095862	up
Os.13604.1.S1_at	2.4697933	up	-1.90666	down	2.856957	up	1.295351	up
Os.26406.1.S1_at	4.0635877	up	-1.08927	down	1.632066	up	3.730566	up
Os.48975.1.S1_at	-1.1720942	down	2.083399	up	-1.39062	down	1.777501	up
Os.21320.1.S1_at	-2.18678	down	-1.22408	down	-1.22387	down	-2.67678	down
Os.11986.3.S1_x_at	-1.5442396	down	2.916878	up	-1.94735	down	1.888876	up
Os.14409.1.S1_at	-2.4257853	down	-1.07598	down	-1.39203	down	-2.61009	down
Os.27094.1.S1_at	-1.5222849	down	1.386538	up	-2.07588	down	-1.0979	down
Os.8742.1.S1_at	-2.6294396	down	1.091627	up	-1.63421	down	-2.40873	down
OsAffx.29695.1.S1_s_	2.170318	up	1.794259	up	-1.19885	down	3.894111	up
Os.49324.1.S1_at	1.7409233	up	2.740574	up	-1.83157	down	4.771129	up
Os.26728.2.S1_x_at	1.3995686	up	1.690702	up	-1.13002	down	2.366254	up
Os.5434.1.S1_at	-2.602784	down	1.766201	up	-2.64243	down	-1.47366	down
Os.52733.1.S1_at	-1.3875716	down	1.860433	up	-2.7832	down	1.340784	up
Os.14348.1.S1_at	-4.297496	down	1.939517	up	-2.90119	down	-2.21576	down
Os.57568.1.S1_at	-2.027254	down	-1.4764	down	-1.01308	down	-2.99303	down
Os.20221.1.S1_at	1.024671	up	2.381654	up	-1.59237	down	2.440412	up
Os.53924.1.S1_at	-2.0311036	down	-1.00018	down	-1.49528	down	-2.03146	down
Os.17447.1.S1_at	-2.0466857	down	1.103287	up	-1.64952	down	-1.85508	down
Os.27048.1.S1_at	-1.8346571	down	-1.43853	down	-1.03926	down	-2.63921	down
Os.21906.1.S1_at	1.2028035	up	1.391969	up	-2.08082	down	1.674266	up
Os.49507.1.S1_at	1.6856201	up	1.743766	up	-1.16668	down	2.939327	up
Os.53455.1.S1_at	-3.19782	down	-1.05176	down	-1.42091	down	-3.36333	down
Os.11403.1.S1_at	-1.4728589	down	1.435503	up	-2.14524	down	-1.02602	down
Os.48441.1.S1_at	1.8830143	up	2.463908	up	-1.64877	down	4.639574	up
Os.23940.1.A1_at	-1.8147957	down	-1.11096	down	-1.34499	down	-2.01617	down
OsAffx.26361.1.S1_x_	1.886805	up	1.883611	up	-1.26067	down	3.554006	up
Os.57181.1.S1_at	1.8914939	up	1.183727	up	1.262087	up	2.239012	up

Os.41883.1.S1_at	5.5916348	up	-1.30562	down	1.950366	up	4.28273	up
Os.19361.1.S1_at	1.286226	up	1.072037	up	-1.60098	down	1.378881	up
Os.51223.1.S1_at	1.6498954	up	1.374706	up	1.08623	up	2.268121	up
OsAffx.17611.1.S1_at	2.1243777	up	1.586954	up	-1.06276	down	3.371289	up
Os.54440.1.S1_at	-1.0238299	down	3.124215	up	-2.09242	down	3.051498	up
Os.22962.2.A1_at	-1.447654	down	-1.41348	down	-1.05607	down	-2.04623	down
Os.26875.1.S1_at	-2.1723938	down	1.061302	up	-1.5841	down	-2.04691	down
Os.6270.1.S1_at	-1.4808217	down	-1.1952	down	1.783927	up	-1.76988	down
OsAffx.23395.2.S1_at	-2.0509834	down	1.044313	up	-1.55869	down	-1.96395	down
Os.10209.1.S1_x_at	-2.399632	down	-1.1973	down	-1.24622	down	-2.87309	down
Os.12288.1.S1_at	1.795825	up	2.609676	up	-1.74955	down	4.686523	up
Os.27424.1.S1_at	-2.4332924	down	-1.07182	down	-1.39152	down	-2.60805	down
Os.55757.1.S1_s_at	-1.5324682	down	1.40366	up	-2.09346	down	-1.09177	down
OsAffx.4209.1.S1_at	-2.363536	down	-1.00929	down	-1.47743	down	-2.3855	down
Os.55800.1.S1_at	-3.413625	down	1.91069	up	-2.84879	down	-1.78659	down
Os.49399.1.S1_at	-2.0342884	down	-1.08015	down	-1.38014	down	-2.19734	down
Os.27807.1.S1_a_at	1.1657745	up	1.336931	up	-1.99293	down	1.55856	up
Os.32717.1.S1_at	-2.0621436	down	-1.04181	down	-1.43081	down	-2.14836	down
Os.24889.2.S1_x_at	-2.1930268	down	-1.00877	down	-1.4776	down	-2.21225	down
Os.15584.1.S1_at	-2.6972222	down	1.24396	up	-1.85412	down	-2.16825	down
Os.51643.1.S1_at	-1.8672007	down	-1.19585	down	-1.24626	down	-2.2329	down
Os.35590.1.S1_x_at	-1.7125353	down	-1.2662	down	-1.17687	down	-2.16841	down
Os.15815.1.S1_at	1.6842693	up	1.810159	up	-1.21479	down	3.048795	up
Os.49485.1.S1_at	1.4209031	up	-2.23216	down	1.498074	up	-1.57095	down
Os.30512.1.S1_at	4.070257	up	1.084119	up	1.374271	up	4.412642	up
OsAffx.27304.1.S1_at	1.3563613	up	1.71155	up	-1.14886	down	2.321479	up
Os.27490.1.A1_at	-1.9669124	down	-1.22263	down	-1.21839	down	-2.40482	down
OsAffx.6040.1.S1_at	-2.6948612	down	1.084691	up	-1.61572	down	-2.48445	down
Os.12721.1.S1_at	-2.3061938	down	1.173416	up	-1.74753	down	-1.96537	down
Os.28422.1.S1_at	1.6284943	up	-1.40272	down	2.088833	up	1.160955	up
Os.17690.1.S1_at	2.0785918	up	-1.10519	down	1.645764	up	1.880763	up
Os.23578.1.S1_at	-1.7147541	down	2.207468	up	-3.28673	down	1.287338	up
Os.16133.1.S1_at	-2.0804138	down	1.100777	up	1.35241	up	-1.88995	down
Os.57469.1.S1_x_at	3.8078911	up	-1.70205	down	2.533353	up	2.237235	up
Os.32411.1.S1_at	-2.8513145	down	-1.03007	down	-1.44477	down	-2.93706	down
Os.50827.1.S1_x_at	-1.9019222	down	-1.19674	down	-1.24294	down	-2.27611	down
Os.16221.1.S1_at	1.841323	up	1.867152	up	-1.25552	down	3.43803	up
Os.5961.2.S1_x_at	-1.5692827	down	1.508045	up	-2.24242	down	-1.04061	down
Os.12150.1.S1_s_at	-1.4076242	down	-1.85374	down	1.246869	up	-2.60937	down
Os.57016.1.S1_at	-2.1287827	down	1.328497	up	1.118655	up	-1.6024	down
Os.33244.1.S2_at	1.0998911	up	-2.76831	down	1.862923	up	-2.5169	down
Os.5632.1.S1_a_at	-1.7817284	down	1.53805	up	-2.28506	down	-1.15843	down
Os.10651.1.S1_at	4.528579	up	-1.03977	down	1.543643	up	4.355384	up
Os.50253.2.S1_x_at	-2.3552346	down	1.232535	up	-1.82931	down	-1.91089	down
Os.57350.1.S1_at	-1.7599511	down	-1.3971	down	-1.06221	down	-2.45882	down
Os.17307.1.S1_at	-2.286059	down	-1.28226	down	-1.15734	down	-2.93132	down
Os.9101.1.S1_at	-2.8537858	down	2.297942	up	-3.40974	down	-1.24189	down

OsAffx.27482.2.S1_s_	-4.224154	down	1.794752	up	-2.66295	down	-2.35361	down
Os.27138.1.S1_at	-1.3322154	down	-2.22815	down	1.50174	up	-2.96837	down
Os.8382.1.S1_s_at	-1.2317532	down	2.887167	up	-1.94599	down	2.343949	up
Os.48609.1.S1_s_at	-1.4399148	down	2.018947	up	-1.36087	down	1.402129	up
Os.51883.1.S1_at	1.7763191	up	-1.67992	down	2.492278	up	1.057385	up
Os.46690.1.S1_x_at	1.5526997	up	2.191989	up	-1.47753	down	3.4035	up
Os.57499.1.S1_x_at	2.1345525	up	-1.15961	down	1.720339	up	1.840751	up
Os.51894.1.S1_at	1.90554	up	1.282191	up	1.157007	up	2.443267	up
Os.45923.1.S1_at	-1.3146114	down	2.088326	up	-1.40783	down	1.58855	up
Os.12391.1.S1_a_at	2.033697	up	-1.01157	down	1.500522	up	2.010428	up
Os.8942.2.S1_at	-1.7018781	down	-1.22736	down	-1.2083	down	-2.08882	down
Os.10300.1.S1_x_at	-1.2932484	down	1.491196	up	-2.21146	down	1.153062	up
Os.51742.1.S1_x_at	1.1202767	up	-2.07953	down	3.083884	up	-1.85627	down
Os.7830.1.S1_at	1.3208485	up	1.590598	up	-1.07268	down	2.100938	up
Os.8977.1.S1_at	-1.0711781	down	-1.45217	down	2.153232	up	-1.55553	down
Os.6087.1.S1_at	1.250243	up	1.829806	up	-1.23415	down	2.287702	up
Os.39043.1.S1_s_at	1.6629667	up	1.219685	up	1.215397	up	2.028296	up
OsAffx.27153.1.S1_at	-1.208004	down	2.398318	up	-1.61791	down	1.985356	up
Os.48061.1.A1_x_at	1.1867889	up	10.13199	up	-6.8373	down	12.02454	up
Os.9008.1.S1_at	-2.2105665	down	1.11774	up	-1.65585	down	-1.97771	down
Os.57062.1.S1_at	-3.4808717	down	1.646898	up	-2.43971	down	-2.11359	down
Os.28002.1.S1_at	1.6426463	up	2.077129	up	-1.40233	down	3.411988	up
Os.18128.1.S1_a_at	-2.0767303	down	1.004939	up	-1.48851	down	-2.06652	down
Os.18677.1.S1_at	-1.5134122	down	1.592758	up	-2.359	down	1.052428	up
Os.57426.1.S1_x_at	1.9700106	up	2.320169	up	-1.5667	down	4.570756	up
Os.15547.1.S1_at	-1.971687	down	-1.03644	down	-1.42853	down	-2.04354	down
Os.57154.1.S1_at	-1.7162496	down	-1.31196	down	-1.12826	down	-2.25165	down
OsAffx.24076.1.S1_at	2.2872853	up	-1.22491	down	1.812959	up	1.867313	up
Os.25167.2.S1_at	-2.311711	down	1.275335	up	-1.88741	down	-1.81263	down
Os.10553.1.S1_s_at	1.8593401	up	1.205467	up	1.227573	up	2.241372	up
Os.6728.1.S1_a_at	-1.4453417	down	-1.43811	down	-1.02895	down	-2.07857	down
Os.27587.1.S1_at	-2.2760863	down	1.094508	up	-1.61956	down	-2.07955	down
Os.8786.1.S1_a_at	-1.8855263	down	1.734588	up	-2.56666	down	-1.08702	down
Os.7403.1.S1_at	1.2750632	up	-1.49149	down	2.206216	up	-1.16974	down
Os.10011.1.S1_at	-2.2033432	down	1.527706	up	-2.25967	down	-1.44226	down
Os.54380.1.S1_at	-1.3159701	down	-1.55744	down	1.053038	up	-2.04954	down
Os.21075.1.S1_at	2.7234066	up	1.078338	up	1.37146	up	2.936752	up
Os.6177.3.S1_at	-1.3787088	down	-1.48017	down	1.00102	up	-2.04072	down
Os.17805.1.S1_at	1.3843795	up	1.610498	up	-1.08935	down	2.229541	up
Os.37564.1.S1_x_at	1.5019203	up	1.362662	up	1.084859	up	2.04661	up
Os.18963.1.S1_a_at	-3.427818	down	1.382499	up	-2.04351	down	-2.47944	down
Os.9858.1.S1_at	-2.417155	down	1.02556	up	-1.51588	down	-2.35691	down
Os.56274.1.S1_at	-1.0151442	down	2.082324	up	-1.40892	down	2.05126	up
Os.30033.1.S1_at	-1.5079362	down	2.213776	up	-1.49819	down	1.468083	up
Os.51582.1.S2_at	-1.6569551	down	-1.40102	down	-1.05467	down	-2.32142	down
Os.7128.1.S1_at	1.4004132	up	1.886017	up	-1.27661	down	2.641203	up
Os.9877.1.S1_at	1.4640127	up	1.391825	up	1.061055	up	2.037649	up

Os.24620.1.S2_a_at	-2.3760033	down	1.27526	up	-1.8826	down	-1.86315	down
OsAffx.17500.1.S1_at	3.1071138	up	-1.57321	down	2.322182	up	1.975018	up
Os.15212.1.A1_a_at	-1.5183654	down	1.494019	up	-2.20527	down	-1.0163	down
OsAffx.7712.1.S1_at	1.2929146	up	5.895811	up	-3.99446	down	7.622781	up
Os.26721.1.S1_at	-1.0477042	down	1.372076	up	-2.02496	down	1.309603	up
Os.47387.1.A1_at	-2.9066424	down	1.062415	up	-1.56756	down	-2.73588	down
Os.19406.1.S1_at	-2.4831784	down	-1.33659	down	-1.10351	down	-3.31899	down
Os.46022.1.S1_x_at	-1.2162074	down	-2.54365	down	1.724943	up	-3.09361	down
Os.47421.1.S1_at	-2.130298	down	1.298341	up	-1.91419	down	-1.64078	down
Os.19448.1.S1_at	-1.7409062	down	-1.78974	down	1.214143	up	-3.11577	down
Os.14951.1.S1_at	7.6774874	up	1.647192	up	-1.1175	down	12.64629	up
OsAffx.12975.1.S1_at	2.1352746	up	-1.24268	down	1.831373	up	1.718279	up
Os.8741.1.S1_at	5.6457872	up	-2.73914	down	4.035692	up	2.061157	up
Os.23796.1.S1_at	-2.0178504	down	-1.0579	down	-1.3925	down	-2.13468	down
Os.35855.2.S1_x_at	-2.0864294	down	1.179843	up	-1.73769	down	-1.7684	down
Os.17619.1.S1_at	1.2561667	up	2.018631	up	-1.37065	down	2.535737	up
Os.7304.1.S1_at	-2.3468475	down	1.646323	up	-2.42412	down	-1.42551	down
Os.55490.1.S1_at	-1.7879764	down	-1.26701	down	-1.16208	down	-2.26539	down
Os.11019.1.S1_at	-1.2578387	down	-1.27921	down	1.883012	up	-1.60903	down
OsAffx.23217.1.S1_x_	3.3281941	up	-1.33587	down	1.966022	up	2.4914	up
Os.11863.2.S1_at	-2.2387743	down	1.164309	up	-1.71201	down	-1.92283	down
Os.25340.1.A1_at	-3.123289	down	1.231294	up	-1.81035	down	-2.53659	down
Os.46324.1.S1_at	-2.0203886	down	-1.14519	down	-1.28385	down	-2.31373	down
Os.15821.1.A1_x_at	-1.4714786	down	-1.37242	down	-1.07106	down	-2.01948	down
Os.35502.1.A1_at	-1.4154115	down	2.131404	up	-1.45006	down	1.505855	up
Os.46382.1.S1_at	-2.5487726	down	1.300257	up	-1.91056	down	-1.96021	down
Os.10619.1.S2_at	1.5257306	up	1.438966	up	1.021083	up	2.195475	up
Os.4152.1.S1_at	1.1931905	up	1.596827	up	-2.34607	down	1.905319	up
Os.24634.1.A1_s_at	-2.1970456	down	-1.01891	down	-1.44192	down	-2.23859	down
Os.9705.1.S1_at	2.3163865	up	1.419344	up	1.034824	up	3.287749	up
Os.57036.1.S1_at	1.9241377	up	1.118071	up	1.312607	up	2.151323	up
Os.50943.1.S1_at	-2.1643486	down	-1.15596	down	-1.26948	down	-2.5019	down
Os.52667.1.S1_at	-1.2978446	down	-1.88802	down	1.286849	up	-2.45036	down
Os.8670.1.S1_at	1.7841945	up	1.820141	up	-1.24082	down	3.247486	up
Os.9195.1.S1_at	1.8168818	up	-1.40718	down	2.063696	up	1.291152	up
Os.7756.1.S1_at	-2.9057899	down	2.916613	up	-4.27726	down	1.003725	up
Os.11361.1.S1_at	-1.354686	down	1.451566	up	-2.12803	down	1.071515	up
Os.46338.1.S1_at	-2.083113	down	1.230765	up	-1.80415	down	-1.69254	down
Os.24850.1.S1_x_at	-2.0910168	down	-1.34634	down	-1.08868	down	-2.81523	down
Os.18008.1.S1_at	-2.4066386	down	1.232294	up	-1.80618	down	-1.95297	down
Os.34118.1.S1_at	-4.8163795	down	1.14025	up	-1.67093	down	-4.22397	down
Os.9623.2.S2_x_at	-2.107026	down	-1.02488	down	-1.42937	down	-2.15945	down
Os.429.1.S1_at	-1.6759335	down	-1.31872	down	-1.11067	down	-2.21008	down
Os.8537.1.A1_at	-1.6015849	down	-1.27516	down	-1.14859	down	-2.04227	down
Os.53407.1.S1_x_at	2.2665954	up	1.336969	up	1.09531	up	3.030367	up
Os.14574.1.S1_at	1.5740268	up	1.547866	up	-1.057	down	2.436382	up
Os.9506.1.S1_a_at	-2.0246375	down	1.815861	up	-1.2402	down	-1.11497	down

OsAffx.7294.1.S1_at	1.4597906	up	-1.44878	down	2.121031	up	1.007598	up
Os.12864.4.S1_x_at	-1.8608738	down	1.463267	up	-2.1415	down	-1.27173	down
Os.6085.1.S1_at	2.3993154	up	1.355529	up	1.079622	up	3.252342	up
OsAffx.4511.1.S1_at	-1.8169835	down	2.23351	up	-3.26864	down	1.229241	up
Os.17446.2.S1_at	1.954175	up	-3.39002	down	2.316864	up	-1.73476	down
Os.12336.1.S1_at	-2.2818055	down	-1.06448	down	-1.37451	down	-2.42894	down
Os.17890.1.S1_at	-1.3274101	down	-1.99553	down	1.364232	up	-2.64889	down
Os.9514.1.S1_at	-1.9966581	down	-1.14939	down	-1.27213	down	-2.29493	down
OsAffx.4765.1.S1_s_a	-2.194924	down	-1.06858	down	-1.36806	down	-2.34544	down
Os.52743.1.S1_at	-2.5628047	down	1.302105	up	-1.90331	down	-1.9682	down
Os.28030.1.S1_s_at	-1.1898338	down	1.716738	up	-2.50929	down	1.442839	up
Os.14644.1.S1_at	-1.7177099	down	-1.3567	down	-1.07728	down	-2.33042	down
Os.20557.1.S1_s_at	2.34327	up	-1.44226	down	2.106958	up	1.624716	up
Os.23224.1.S1_at	1.5556736	up	-2.04634	down	2.989052	up	-1.31541	down
Os.16914.1.S2_at	-1.5542066	down	-1.35126	down	-1.08056	down	-2.10013	down
Os.32192.1.S1_at	1.3990359	up	2.269789	up	-1.55456	down	3.175516	up
Os.11152.1.S1_at	1.1196413	up	1.251332	up	-1.82663	down	1.401043	up
Os.54671.1.S1_at	2.7134323	up	-1.01184	down	1.477015	up	2.681671	up
OsAffx.32172.1.S1_s_	2.3098438	up	-1.29519	down	1.890534	up	1.783402	up
Os.49515.2.S1_x_at	-2.4375854	down	-1.06951	down	-1.36351	down	-2.60701	down
Os.5661.1.S1_s_at	-2.0321312	down	-1.13061	down	-1.28962	down	-2.29754	down
Os.8176.1.S1_at	1.5009638	up	1.375112	up	1.060269	up	2.063994	up
OsAffx.23593.1.S1_s_	1.0973833	up	2.06215	up	-1.4144	down	2.262969	up
Os.14365.1.S1_at	1.1203195	up	1.899349	up	-1.30294	down	2.127878	up
Os.31883.1.A1_at	-3.3623633	down	-1.04093	down	-1.40041	down	-3.49997	down
Os.14938.1.S1_at	3.0687141	up	4.674285	up	-3.20661	down	14.34405	up
Os.14173.1.S1_at	-2.5311604	down	-1.16346	down	-1.25289	down	-2.94491	down
Os.25559.1.S1_at	-2.0821726	down	1.148698	up	-1.67434	down	-1.81264	down
Os.5585.1.S1_at	-1.5646435	down	-1.70677	down	1.171293	up	-2.67049	down
Os.50767.1.S1_x_at	-1.5025481	down	-1.3554	down	-1.07505	down	-2.03655	down
Os.38131.1.S1_at	-1.9053346	down	-1.11253	down	-1.30958	down	-2.11975	down
OsAffx.26114.1.S1_at	1.9192177	up	2.36721	up	-1.62482	down	4.543191	up
OsAffx.30833.1.S1_at	-1.152499	down	3.255609	up	-2.2353	down	2.824826	up
Os.50267.1.S1_at	1.622651	up	1.370228	up	1.062811	up	2.223402	up
Os.49826.1.S1_at	1.2654479	up	-1.69034	down	2.461485	up	-1.33577	down
OsAffx.4069.1.S1_at	-2.1337206	down	1.170117	up	-1.70349	down	-1.82351	down
Os.49113.1.S1_s_at	-1.7057487	down	-1.22872	down	-1.18483	down	-2.09588	down
Os.10093.2.S1_at	-1.6285492	down	-1.39487	down	-1.04365	down	-2.27162	down
Os.47485.1.S1_at	1.4841003	up	-1.81874	down	2.647414	up	-1.22548	down
Os.27651.1.S1_at	1.3040057	up	1.82634	up	-1.25488	down	2.381558	up
Os.23131.1.S1_at	1.4492974	up	1.413703	up	1.029425	up	2.048875	up
Os.27325.1.S1_at	-2.3297796	down	-1.11635	down	-1.30361	down	-2.60084	down
Os.46594.2.S1_at	-3.111555	down	1.153548	up	-1.67826	down	-2.69738	down
Os.9757.1.S1_at	-1.8759001	down	-1.24534	down	-1.16817	down	-2.33613	down
Os.51302.1.A1_at	-1.8843987	down	-1.24999	down	-1.16376	down	-2.35547	down
Os.50155.1.S1_at	3.6444533	up	-1.3476	down	1.960293	up	2.704397	up
Os.12016.1.S1_at	1.875624	up	1.235278	up	1.177473	up	2.316916	up

Os.53381.1.S1_at	-2.842099	down	1.559645	up	-2.26842	down	-1.82227	down
Os.11269.1.S1_at	-1.2898206	down	-1.82763	down	1.256713	up	-2.35731	down
Os.15516.1.S1_at	-3.9844277	down	4.363255	up	-3.0003	down	1.095077	up
Os.20667.1.S1_at	-2.2249818	down	-1.13793	down	-1.27759	down	-2.53188	down
Os.50583.1.S1_at	1.3242797	up	-1.38814	down	2.018025	up	-1.04822	down
Os.50180.1.S1_at	1.9360125	up	1.369619	up	1.061378	up	2.651599	up
Os.28983.1.S1_at	-1.7353055	down	1.492425	up	-2.16899	down	-1.16274	down
Os.30324.2.S1_x_at	-1.1319087	down	-2.54966	down	3.705326	up	-2.88598	down
Os.31975.1.S1_x_at	6.262105	up	-5.01506	down	7.28505	up	1.24866	up
Os.27393.1.S1_a_at	-2.721875	down	1.37038	up	-1.99032	down	-1.98622	down
Os.10399.1.S1_at	1.538535	up	2.926611	up	-2.01504	down	4.502693	up
Os.11076.1.S1_at	2.5103583	up	-1.35421	down	1.966766	up	1.853737	up
Os.47335.1.S1_at	-1.6172945	down	-1.29847	down	-1.11835	down	-2.1	down
Os.54327.1.S1_at	1.5585802	up	1.469845	up	-1.01232	down	2.290871	up
Os.52037.1.S1_at	2.0895154	up	-1.13919	down	1.654057	up	1.834209	up
Os.24101.1.A1_at	-2.185345	down	1.200996	up	-1.74379	down	-1.81961	down
Os.53893.1.S1_at	-2.0859017	down	1.53735	up	-2.23197	down	-1.35682	down
Os.6247.1.S1_at	1.3690166	up	1.743225	up	-1.20073	down	2.386504	up
Os.51193.1.S1_x_at	-1.2442155	down	1.479745	up	-2.14822	down	1.1893	up
Os.25065.1.S1_a_at	-2.3592255	down	1.045804	up	-1.51814	down	-2.2559	down
OsAffx.26384.5.S1_x_	-1.3246895	down	-1.59662	down	1.100102	up	-2.11503	down
Os.10845.1.S1_s_at	6.589228	up	-2.60922	down	3.786061	up	2.525359	up
Os.11034.1.S1_at	-2.0590386	down	1.297326	up	-1.88244	down	-1.58714	down
Os.49838.1.S1_at	2.6259825	up	-1.01155	down	1.46756	up	2.595995	up
Os.9035.1.S1_at	-3.0581117	down	1.216515	up	-1.7647	down	-2.51383	down
Os.26818.1.S1_a_at	-1.5132089	down	1.393054	up	-2.02071	down	-1.08625	down
Os.22571.1.S1_at	2.4010253	up	-1.27526	down	1.84978	up	1.882777	up
OsAffx.14990.1.S1_at	2.902777	up	-1.09402	down	1.586744	up	2.653307	up
Os.623.1.S1_x_at	3.2044756	up	-1.45665	down	2.11267	up	2.199889	up
Os.52212.1.S1_at	-2.6690447	down	1.014957	up	-1.4719	down	-2.62971	down
OsAffx.10845.1.S1_s_	1.5097451	up	1.351682	up	1.072747	up	2.040696	up
OsAffx.9156.1.S1_at	-1.2168263	down	2.3557	up	-1.62474	down	1.935938	up
Os.20261.1.S1_at	1.0564184	up	1.890857	up	-2.74121	down	1.997536	up
Os.45749.1.A1_at	-4.9048047	down	1.84221	up	-2.66984	down	-2.66246	down
Os.3300.1.S1_s_at	-1.922467	down	-1.07446	down	-1.34847	down	-2.06562	down
Os.7733.1.S2_at	-1.6796442	down	-1.53258	down	1.057806	up	-2.5742	down
Os.51023.1.S1_at	-1.7594043	down	-1.26757	down	-1.14283	down	-2.23017	down
OsAffx.29976.1.A1_at	1.1508371	up	1.744031	up	-1.20424	down	2.007095	up
Os.24233.2.S1_at	-1.7734845	down	-1.2768	down	-1.13421	down	-2.26438	down
Os.57332.1.S1_at	-2.8901691	down	1.100243	up	-1.59322	down	-2.62685	down
Os.10525.1.S1_at	2.012045	up	1.661027	up	-1.14713	down	3.34206	up
Os.9873.1.S1_at	-1.4162921	down	-1.1858	down	1.716858	up	-1.67944	down
Os.27227.1.S1_at	-1.7767826	down	1.889928	up	-2.73599	down	1.06368	up
Os.51462.1.S1_at	-2.594961	down	1.512965	up	-2.19018	down	-1.71515	down
Os.3048.2.S1_x_at	-3.0065172	down	1.623747	up	-2.35032	down	-1.85159	down
Os.8668.1.S1_x_at	-1.4769235	down	-1.37316	down	-1.0541	down	-2.02806	down
Os.19331.1.S1_at	-1.7379466	down	-1.32391	down	-1.0933	down	-2.30088	down

Os.29053.1.S1_at	1.9297596	up	-1.44599	down	2.092353	up	1.334563	up
Os.11870.1.S1_at	-2.6643472	down	1.433501	up	-2.0742	down	-1.85863	down
Os.18919.1.A1_at	-1.9245892	down	1.772621	up	-2.56487	down	-1.08573	down
Os.45991.1.S1_x_at	-2.1257842	down	1.361376	up	-1.96974	down	-1.5615	down
Os.12402.1.S1_at	-1.7687263	down	-1.22357	down	-1.1824	down	-2.16416	down
Os.54067.1.S1_at	-1.5823667	down	-1.28806	down	-1.12289	down	-2.03818	down
Os.37227.1.S1_at	-2.0866184	down	1.113257	up	-1.60996	down	-1.87434	down
OsAffx.27628.1.S1_s_	-1.7371	down	-1.179	down	-1.2266	down	-2.04803	down
Os.6037.2.S1_x_at	1.0070169	up	4.185644	up	-2.89487	down	4.215014	up
Os.10052.1.S1_at	-2.183925	down	1.51975	up	-2.19731	down	-1.43703	down
Os.27148.1.A1_at	-1.5289203	down	1.535029	up	-2.21922	down	1.003995	up
Os.53480.1.S1_x_at	1.86919	up	1.293342	up	1.117661	up	2.417502	up
OsAffx.27444.1.S1_at	1.1809095	up	-1.46159	down	2.11184	up	-1.23769	down
Os.17448.1.S1_at	-1.6833175	down	-1.40863	down	-1.02567	down	-2.37118	down
OsAffx.4692.1.S1_x_a	-2.105795	down	1.259351	up	-1.81916	down	-1.67213	down
Os.52761.1.A1_at	-3.6437473	down	1.249191	up	-1.80443	down	-2.91689	down
Os.52465.1.S1_at	-1.9255198	down	1.481336	up	-2.13945	down	-1.29985	down
Os.25013.1.S1_x_at	1.1684569	up	1.759381	up	-1.21839	down	2.055761	up
Os.8457.1.S1_at	1.4036133	up	3.195651	up	-2.21306	down	4.485459	up
Os.18071.1.S1_s_at	-1.6786222	down	-1.19169	down	-1.21162	down	-2.00039	down
Os.8271.1.S1_a_at	2.4829774	up	1.061317	up	1.360388	up	2.635225	up
Os.27454.1.S1_x_at	-1.4097577	down	1.463122	up	-2.11239	down	1.037853	up
Os.54117.1.S1_at	-2.4399288	down	1.140075	up	-1.64571	down	-2.14015	down
Os.6306.1.S2_at	-2.1022272	down	1.029675	up	-1.486	down	-2.04164	down
Os.7674.1.S1_at	-2.1205657	down	5.542815	up	-7.99816	down	2.613838	up
Os.32537.1.S1_s_at	-2.3374372	down	1.151953	up	-1.6621	down	-2.02911	down
Os.24543.1.S1_at	1.7040316	up	-1.7311	down	2.497274	up	-1.01588	down
Os.13494.1.S1_at	-1.9920999	down	1.484702	up	-2.14163	down	-1.34175	down
Os.49634.1.S1_x_at	-1.3414128	down	2.248772	up	-3.24331	down	1.676421	up
Os.10575.1.S1_at	-2.2664528	down	1.022438	up	-1.47457	down	-2.21672	down
Os.8714.1.S1_at	-1.3919035	down	1.397428	up	-2.01537	down	1.003969	up
Os.49427.1.S1_at	-2.682904	down	1.14824	up	1.255891	up	-2.33654	down
Os.9093.1.S1_at	1.2451768	up	2.659561	up	-1.84455	down	3.311623	up
Os.47914.1.S1_at	1.0919714	up	2.126014	up	-1.47468	down	2.321546	up
Os.28778.1.S1_at	-2.211878	down	1.504532	up	-2.16901	down	-1.47014	down
Os.34701.1.S1_s_at	-2.1183548	down	1.083544	up	-1.56209	down	-1.95502	down
Os.10169.1.S1_at	-1.6586089	down	-1.67044	down	1.1588	up	-2.7706	down
Os.19444.1.S1_at	-1.6133641	down	1.738455	up	-2.50596	down	1.077534	up
Os.39420.2.S1_x_at	1.4906591	up	1.374753	up	1.04846	up	2.049289	up
Os.10529.1.S1_s_at	-2.1771486	down	-1.31644	down	-1.09483	down	-2.86609	down
Os.33891.1.S1_at	-1.5500911	down	-1.30609	down	-1.10348	down	-2.02456	down
Os.2066.1.S1_a_at	-1.8225329	down	1.148058	up	1.255215	up	-1.58749	down
Os.18521.1.A1_at	-2.060997	down	-1.00025	down	-1.44065	down	-2.06151	down
Os.57506.1.S1_at	1.3937861	up	1.761987	up	-1.2229	down	2.455832	up
Os.27888.1.S1_at	-2.2773151	down	-1.00054	down	-1.44004	down	-2.27854	down
Os.26885.1.A1_at	-1.4816334	down	2.480333	up	-1.72151	down	1.674053	up
Os.30135.1.S1_at	-2.1045418	down	1.228847	up	-1.77031	down	-1.71261	down

Os.6318.1.S1_at	-1.9283682	down	-1.30492	down	-1.10397	down	-2.51636	down
Os.18356.1.S1_x_at	-2.0627487	down	1.374583	up	-1.97987	down	-1.50064	down
Os.8910.1.S1_at	1.7643459	up	1.728751	up	-1.20052	down	3.050115	up
Os.10175.1.S1_at	-1.4723723	down	2.582229	up	-1.79327	down	1.753788	up
Os.18880.1.S1_at	-2.4198534	down	-1.10151	down	-1.30722	down	-2.6655	down
Os.11808.2.S1_at	1.3705806	up	-2.97079	down	2.06422	up	-2.16754	down
Os.10132.1.S1_at	-1.0376601	down	1.661377	up	-2.39085	down	1.60108	up
OsAffx.25262.1.S1_at	1.6919823	up	-2.22101	down	1.543639	up	-1.31266	down
Os.56184.1.S1_at	1.6230764	up	1.232252	up	1.167571	up	2.000039	up
OsAffx.14107.1.S1_at	-1.7666368	down	1.503483	up	-2.16286	down	-1.17503	down
Os.39257.1.S1_at	1.3962452	up	1.45594	up	-1.01235	down	2.03285	up
Os.9029.2.S1_x_at	-1.4432199	down	1.40853	up	-2.0253	down	-1.02463	down
OsAffx.11629.1.S1_at	-1.3296139	down	2.131741	up	-1.48259	down	1.603278	up
Os.15908.1.S1_s_at	1.0394293	up	-2.77958	down	1.933488	up	-2.67414	down
Os.52432.1.S1_at	-1.5322795	down	1.062321	up	1.353149	up	-1.44239	down
Os.23297.1.S1_at	2.3990893	up	-1.65214	down	2.374877	up	1.452108	up
Os.35012.1.S1_at	-2.001406	down	1.27311	up	-1.82979	down	-1.57206	down
Os.52752.1.S1_at	2.9513216	up	1.694682	up	-1.17913	down	5.001554	up
Os.20141.1.S1_at	-1.8676486	down	-1.09076	down	-1.31742	down	-2.03716	down
OsAffx.5756.1.S1_at	-1.482231	down	-1.36547	down	-1.05217	down	-2.02394	down
Os.50639.1.S1_at	2.4056606	up	2.470419	up	-1.71959	down	5.942991	up
Os.34767.1.S1_at	-2.790577	down	1.249281	up	-1.79463	down	-2.23375	down
Os.54121.1.S1_s_at	-3.1312885	down	2.331232	up	-3.34767	down	-1.34319	down
OsAffx.26838.1.S1_at	11.57416	up	1.356763	up	1.058406	up	15.70339	up
Os.27165.1.A1_at	-2.9025164	down	2.004873	up	-2.87895	down	-1.44773	down
OsAffx.25311.1.S1_at	-2.2186887	down	1.236347	up	-1.77535	down	-1.79455	down
Os.57051.1.S1_at	1.1776562	up	-2.82245	down	1.965771	up	-2.39666	down
Os.32277.1.S1_at	-1.9469948	down	1.370683	up	1.047353	up	-1.42046	down
Os.32856.1.S1_at	-2.3323376	down	1.582947	up	-2.27168	down	-1.47341	down
OsAffx.28070.1.S1_at	-2.0562887	down	1.07571	up	-1.54306	down	-1.91156	down
OsAffx.10973.1.S1_s_	2.250896	up	-1.7303	down	2.481327	up	1.30087	up
Os.12454.1.S1_a_at	-2.805167	down	1.985826	up	-2.84749	down	-1.41259	down
Os.10215.1.S1_at	-1.1185933	down	2.469099	up	-1.72208	down	2.207326	up
Os.47859.1.S1_at	-2.007075	down	-1.2124	down	-1.18235	down	-2.43337	down
Os.14045.1.S1_s_at	-1.9306207	down	1.11658	up	1.283782	up	-1.72905	down
OsAffx.1083.1.S1_at	1.9332274	up	1.229761	up	1.165556	up	2.377407	up
Os.40415.1.A1_s_at	-1.3204906	down	1.728848	up	-2.47748	down	1.309246	up
Os.27106.1.S1_at	-2.0473385	down	1.534722	up	-2.19922	down	-1.33401	down
Os.49301.1.A1_at	-4.569868	down	1.265188	up	-1.81285	down	-3.61201	down
Os.6120.1.S1_at	1.9278837	up	1.218347	up	1.175938	up	2.348831	up
Os.22781.1.S1_at	1.5645528	up	-1.60422	down	2.29824	up	-1.02535	down
Os.32526.1.S1_at	-2.1741529	down	-1.08582	down	-1.3193	down	-2.36074	down
OsAffx.15313.1.S1_s_	-1.9236088	down	1.400498	up	-2.00585	down	-1.37352	down
Os.12484.1.S1_at	-1.551689	down	1.078096	up	1.328341	up	-1.43929	down
Os.456.1.S1_a_at	-1.5656238	down	1.879458	up	-2.69068	down	1.200453	up
OsAffx.27508.100.S1_	-1.2310061	down	1.529262	up	-2.18872	down	1.242286	up
OsAffx.15781.4.S1_x_	-2.341604	down	1.167046	up	-1.67022	down	-2.00644	down

Os.46522.1.S1_a_at	-2.0855806	down	1.180378	up	-1.68925	down	-1.76688	down
Os.9324.1.S1_at	-3.3331351	down	-1.03775	down	-1.37893	down	-3.45897	down
Os.4370.1.S1_at	1.6761148	up	1.408252	up	1.015866	up	2.360393	up
Os.4573.1.S1_at	2.4221544	up	-1.57676	down	1.102483	up	1.536157	up
Os.50421.1.S1_at	-1.960827	down	-1.12708	down	-1.26893	down	-2.21	down
Os.27983.1.S1_at	-2.087087	down	1.309965	up	-1.87341	down	-1.59324	down
Os.22821.1.S1_a_at	-1.8870579	down	-1.1255	down	-1.27065	down	-2.12388	down
Os.22241.1.S1_s_at	1.8951764	up	1.711469	up	-1.19696	down	3.243535	up
Os.11958.1.S1_at	-1.0597854	down	2.315075	up	-1.61938	down	2.184475	up
Os.17880.1.S1_a_at	1.5134286	up	6.116327	up	-4.27843	down	9.256624	up
Os.8550.1.S1_x_at	-2.6771388	down	-1.14328	down	-1.25037	down	-3.06071	down
Os.9823.1.S1_at	-2.0315194	down	-1.59417	down	1.115204	up	-3.23859	down
Os.47732.1.S1_s_at	-1.3814323	down	-1.45528	down	1.018085	up	-2.01037	down
Os.19388.1.S1_at	1.8384138	up	1.16494	up	1.226635	up	2.141643	up
OsAffx.26826.1.S1_at	-3.3250601	down	3.09485	up	-4.42208	down	-1.07439	down
Os.11586.1.S1_at	1.3586899	up	1.64953	up	-1.15449	down	2.2412	up
Os.49726.1.S1_at	1.0617787	up	-1.42981	down	2.042462	up	-1.34662	down
Os.55343.1.S1_at	-1.2035472	down	-1.69358	down	1.185911	up	-2.0383	down
Os.27268.1.A1_at	-1.7924603	down	-1.23614	down	-1.15526	down	-2.21573	down
Os.17611.1.S1_at	-1.4536345	down	-1.44607	down	1.012747	up	-2.10205	down
Os.19474.1.S1_a_at	-2.0754669	down	1.295889	up	-1.85022	down	-1.60158	down
OsAffx.14310.1.S1_s_	-1.6073805	down	-1.30825	down	-1.09128	down	-2.10285	down
Os.55357.1.S1_at	-1.5509853	down	1.100486	up	1.297178	up	-1.40936	down
Os.16183.1.S1_at	-1.3392307	down	-1.22262	down	1.745297	up	-1.63737	down
Os.37565.2.S1_at	-1.8162864	down	3.93047	up	-2.75345	down	2.164014	up
Os.27194.2.A1_x_at	-2.1178412	down	1.531496	up	-2.18609	down	-1.38286	down
Os.8907.1.S1_at	-2.1259098	down	-1.1853	down	-1.20425	down	-2.51984	down
Os.14585.1.S1_s_at	-5.9512987	down	1.681256	up	-2.39902	down	-3.53979	down
Os.53987.1.S1_at	2.1087518	up	-1.31027	down	1.868968	up	1.609409	up
Os.54220.1.S1_at	-2.3438928	down	-1.48254	down	1.039408	up	-3.47491	down
Os.5622.2.S1_x_at	1.0163064	up	3.794829	up	-2.66081	down	3.856709	up
Os.6811.1.S1_s_at	-1.5662647	down	-1.28651	down	-1.10827	down	-2.01502	down
Os.26887.2.A1_a_at	-1.3680317	down	3.017427	up	-2.11714	down	2.20567	up
Os.20151.1.S1_at	1.3282806	up	1.692605	up	-1.18782	down	2.248255	up
OsAffx.28169.1.S1_at	-2.8765435	down	1.865823	up	-2.6586	down	-1.5417	down
Os.27141.1.S1_at	-2.1368086	down	-1.06736	down	-1.33485	down	-2.28074	down
Os.52145.1.S1_at	-2.0930212	down	1.5701	up	-1.10219	down	-1.33305	down
Os.32339.1.S1_at	-2.6650038	down	1.249998	up	-1.78041	down	-2.13201	down
Os.6037.1.S1_at	-1.0327804	down	3.907666	up	-2.74381	down	3.783637	up
Os.20169.1.S1_at	1.4194328	up	1.239257	up	-1.76478	down	1.759041	up
Os.55488.1.S1_at	1.9356703	up	4.548699	up	-3.19463	down	8.804782	up
Os.18681.1.S1_at	-2.0363834	down	1.134964	up	-1.61598	down	-1.79423	down
OsAffx.2966.1.S1_at	1.3573948	up	2.0838	up	-1.46376	down	2.828539	up
Os.17889.1.S1_at	1.6517495	up	1.041517	up	-1.48238	down	1.720325	up
Os.6639.1.S1_at	-2.417736	down	1.334102	up	-1.89881	down	-1.81226	down
Os.11716.1.S1_at	-2.4719512	down	-1.05992	down	-1.34234	down	-2.62006	down
Os.48216.1.S1_at	-1.169132	down	3.492801	up	-2.45517	down	2.987517	up

Os.10128.1.S1_at	-2.074568	down	1.114258	up	-1.58518	down	-1.86184	down
Os.9526.1.S1_at	-1.5523633	down	2.223287	up	-1.56311	down	1.432195	up
Os.7235.1.S1_at	3.4800563	up	1.124625	up	1.264509	up	3.913757	up
Os.55477.1.S1_at	-1.8979807	down	-1.22207	down	-1.16357	down	-2.31946	down
Os.54274.1.S1_at	1.3944873	up	-1.69116	down	2.404751	up	-1.21275	down
Os.18993.2.S1_x_at	-1.7122277	down	-1.7311	down	1.217427	up	-2.96403	down
Os.49258.1.S1_at	2.0582075	up	1.289477	up	1.102704	up	2.654011	up
Os.19764.1.S2_at	-1.5518242	down	1.473605	up	-2.09497	down	-1.05308	down
Os.7961.1.S1_at	-2.094652	down	1.405994	up	-1.99868	down	-1.4898	down
Os.10595.3.S1_x_at	1.5955617	up	1.488966	up	-1.0476	down	2.375737	up
Os.7819.1.S1_at	1.7591356	up	1.163598	up	1.221289	up	2.046926	up
OsAffx.32166.1548.S1	-2.2049184	down	2.358786	up	-3.35203	down	1.069784	up
Os.15821.3.A1_at	-1.7062509	down	-1.49907	down	1.055067	up	-2.55778	down
Os.54892.1.S1_at	1.1580925	up	-1.45581	down	2.068402	up	-1.25708	down
Os.51752.2.S1_x_at	1.132147	up	-2.19807	down	1.547393	up	-1.94151	down
Os.51724.1.S1_at	-2.4695165	down	-1.09715	down	-1.29472	down	-2.70942	down
Os.8890.1.S1_at	1.2461982	up	1.654741	up	-1.16508	down	2.062135	up
Os.11745.2.S1_at	-1.4786568	down	6.247865	up	-8.87321	down	4.225365	up
Os.52582.1.S1_at	-2.1779377	down	1.118098	up	-1.58792	down	-1.94789	down
Os.35590.3.S1_x_at	-2.0101998	down	1.085876	up	-1.54207	down	-1.85122	down
Os.28096.1.S1_at	1.482003	up	1.476206	up	-1.03955	down	2.187741	up
Os.32686.1.S1_at	1.0712405	up	-1.56492	down	2.221862	up	-1.46085	down
OsAffx.29354.2.S1_x_	1.6094514	up	-1.7331	down	2.459833	up	-1.07683	down
Os.27389.3.S1_x_at	1.2996941	up	1.647915	up	-1.16128	down	2.141786	up
Os.37996.1.S1_at	-3.2825174	down	2.054012	up	-2.91438	down	-1.5981	down
Os.15004.1.S1_at	-3.102881	down	1.240809	up	-1.75995	down	-2.50069	down
Os.6231.1.S1_at	1.1597165	up	1.809408	up	-1.27595	down	2.0984	up
OsAffx.14113.1.S1_s_	1.0533347	up	-1.92039	down	2.722985	up	-1.82315	down
Os.18412.1.S1_at	1.664078	up	-1.54014	down	2.183517	up	1.080472	up
OsAffx.25032.1.S1_s_	-2.7059004	down	1.856865	up	-1.31	down	-1.45724	down
Os.7725.1.S1_at	-2.2904758	down	-1.32656	down	-1.0684	down	-3.03846	down
Os.8260.2.S1_at	-2.2853527	down	1.077505	up	-1.52707	down	-2.12097	down
OsAffx.15902.1.S1_at	3.0001261	up	-1.28845	down	1.825819	up	2.328474	up
Os.57426.1.S1_s_at	2.2769763	up	2.268825	up	-1.60182	down	5.166061	up
Os.6851.1.S1_at	-1.0005721	down	-1.50004	down	2.123495	up	-1.5009	down
Os.12064.1.S1_at	-2.991972	down	1.717136	up	-2.43051	down	-1.74242	down
Os.2150.1.S1_at	-2.140947	down	1.152748	up	-1.63146	down	-1.85726	down
OsAffx.22053.1.S1_at	2.19065	up	1.259867	up	1.123334	up	2.759928	up
Os.52527.1.S1_s_at	-3.1527598	down	2.914857	up	-4.12493	down	-1.08162	down
Os.50871.1.S1_at	-1.5000896	down	1.443317	up	-2.04228	down	-1.03933	down
OsAffx.9464.2.S1_x_a	2.148612	up	-1.15824	down	1.638711	up	1.855059	up
Os.27319.1.A1_at	-2.792084	down	-1.07984	down	-1.31019	down	-3.01499	down
Os.48002.1.A1_x_at	-3.0448914	down	-1.16057	down	-1.21869	down	-3.53381	down
Os.3426.2.S1_x_at	1.1125207	up	-1.69893	down	2.401609	up	-1.5271	down
Os.5818.1.S1_x_at	-2.329269	down	1.156583	up	-1.63486	down	-2.01392	down
Os.6825.1.S1_at	-1.3428857	down	1.616993	up	-2.28555	down	1.204118	up
Os.52237.1.S1_at	-1.4577364	down	1.84626	up	-2.60817	down	1.266525	up

Os.20617.2.S1_x_at	1.0418591	up	-2.10029	down	1.48698	up	-2.01591	down
Os.49793.1.S1_at	1.0362659	up	-1.43939	down	2.032901	up	-1.38902	down
Os.7160.1.S1_at	-2.0301006	down	1.422547	up	-2.00905	down	-1.42709	down
Os.46551.1.S1_at	1.2213596	up	2.652115	up	-1.87793	down	3.239187	up
OsAffx.19478.1.S1_at	-1.1052774	down	-1.9237	down	1.362304	up	-2.12623	down
Os.5201.3.S1_at	1.6597785	up	1.237105	up	1.141428	up	2.05332	up
Os.27742.2.S1_a_at	-4.0471644	down	1.607935	up	-2.27013	down	-2.517	down
Os.38086.2.S1_x_at	-2.6550786	down	1.306994	up	-1.84524	down	-2.03144	down
Os.778.1.S1_at	-3.718182	down	3.076568	up	-2.17919	down	-1.20855	down
Os.9515.3.S1_x_at	-2.033422	down	-1.53491	down	1.087679	up	-3.12112	down
Os.19519.1.S1_at	2.411972	up	-1.15549	down	1.630529	up	2.087399	up
Os.55282.1.S1_x_at	-1.7253478	down	-1.25398	down	-1.12494	down	-2.16355	down
Os.55691.1.S1_at	-1.5963157	down	-1.52557	down	1.081481	up	-2.43528	down
Os.50010.1.S1_at	-2.0577185	down	1.026461	up	-1.4479	down	-2.00467	down
Os.24551.4.S1_at	-3.7420852	down	2.33659	up	-3.29582	down	-1.60152	down
Os.27341.1.S1_at	-1.8260235	down	-1.22215	down	-1.15409	down	-2.23167	down
Os.49238.1.S1_at	-1.7441885	down	-1.18409	down	-1.19116	down	-2.06527	down
Os.33244.1.S1_at	-1.0264044	down	-4.52583	down	3.208917	up	-4.64534	down
Os.50315.1.S1_at	-3.3844025	down	1.599951	up	-2.25605	down	-2.11532	down
Os.16079.1.S1_at	-1.8647225	down	-1.14316	down	-1.23326	down	-2.13168	down
Os.10208.1.S1_at	-2.2940052	down	-1.15863	down	-1.2166	down	-2.6579	down
Os.47992.1.S1_at	-1.8634858	down	-1.2603	down	-1.11821	down	-2.34856	down
OsAffx.7795.1.S1_at	1.8181561	up	-4.34321	down	3.08185	up	-2.3888	down
Os.56148.1.S1_at	-1.839	down	-1.09364	down	-1.2885	down	-2.01121	down
Os.48817.1.A1_at	1.9974965	up	2.897495	up	-2.05675	down	5.787736	up
Os.26994.1.A1_at	-3.8131053	down	1.513553	up	-2.13223	down	-2.51931	down
Os.14260.1.A1_at	-2.1733472	down	1.074599	up	-1.51358	down	-2.02247	down
Os.52026.1.S1_at	-2.5789003	down	1.326984	up	-1.86902	down	-1.94343	down
Os.34167.1.S1_at	-2.9108253	down	1.194367	up	-1.68218	down	-2.43713	down
OsAffx.22593.2.S1_s_	1.9393032	up	-2.06562	down	1.467019	up	-1.06513	down
Os.16704.1.S1_at	1.4972692	up	2.501369	up	-1.77719	down	3.745222	up
Os.18770.1.S1_at	-2.2330613	down	1.666684	up	-2.3458	down	-1.33982	down
Os.37438.1.S1_at	-3.0886004	down	1.904922	up	-2.68091	down	-1.62138	down
Os.6566.1.S1_at	-2.4542904	down	1.17415	up	-1.6522	down	-2.09027	down
Os.16257.1.S1_at	1.5301096	up	1.337797	up	1.051522	up	2.046975	up
Os.51138.1.S1_at	-1.315427	down	2.384049	up	-1.69477	down	1.812376	up
Os.141.1.S1_at	1.0335729	up	-1.47045	down	2.067396	up	-1.42269	down
Os.623.1.S1_at	2.122674	up	1.084651	up	1.296004	up	2.30236	up
OsAffx.27307.1.S1_at	2.5790818	up	-1.29607	down	1.821531	up	1.989926	up
Os.1224.1.S1_at	1.5395924	up	2.139068	up	-1.52237	down	3.293292	up
OsAffx.3609.1.S1_at	-1.0533475	down	-2.0249	down	1.441135	up	-2.13292	down
Os.53578.1.S1_at	-2.4442513	down	-1.05943	down	-1.32604	down	-2.58951	down
Os.33799.1.S1_at	-2.224301	down	-1.07391	down	-1.30803	down	-2.3887	down
Os.12933.1.S1_at	-1.2178397	down	-1.20762	down	1.696282	up	-1.47068	down
Os.27135.1.A1_at	-2.136209	down	1.091368	up	-1.53296	down	-1.95737	down
Os.14095.1.S1_at	-1.1849877	down	-2.16332	down	1.540261	up	-2.56351	down
Os.21801.1.S1_at	-1.9082012	down	2.062839	up	-2.89713	down	1.081038	up

Os.9695.1.S1_at	-2.308302	down	1.25166	up	-1.75775	down	-1.84419	down
Os.15284.1.S1_at	-1.4568481	down	2.254261	up	-1.60527	down	1.547355	up
Os.14077.1.S1_at	-1.5218145	down	-1.52828	down	1.088349	up	-2.32575	down
Os.35821.1.S1_at	-1.9011511	down	-1.22774	down	-1.14369	down	-2.33412	down
Os.8322.1.S1_at	1.2664737	up	2.214265	up	-1.57728	down	2.804308	up
Os.26642.1.S1_a_at	-1.8703738	down	1.544507	up	-2.16782	down	-1.21098	down
Os.10924.1.S1_at	-2.0359776	down	-1.30777	down	-1.07298	down	-2.66259	down
Os.5406.1.S1_at	-1.1575403	down	1.594499	up	-2.23665	down	1.377489	up
Os.10105.1.S1_at	2.0504856	up	-1.32223	down	1.854647	up	1.550784	up
Os.49994.1.S1_at	-1.6964667	down	1.84494	up	-2.58733	down	1.087519	up
Os.10652.1.S1_at	2.81777	up	-1.77769	down	2.492723	up	1.585077	up
Os.17919.1.S1_at	1.1682353	up	1.762699	up	-1.25718	down	2.059247	up
Os.26152.1.S1_a_at	1.3861395	up	1.937971	up	-1.38279	down	2.686298	up
Os.405.1.S1_a_at	1.5069201	up	5.449154	up	-3.88834	down	8.211439	up
Os.9212.1.S1_at	-1.5715414	down	1.935307	up	-2.71141	down	1.231471	up
Os.11416.1.S1_at	-1.7695621	down	-1.15548	down	-1.21232	down	-2.0447	down
Os.12150.1.S1_at	-1.533975	down	-1.60416	down	1.145365	up	-2.46074	down
Os.48567.1.S1_at	-2.0591166	down	-1.07927	down	-1.29763	down	-2.22235	down
Os.27906.1.S1_a_at	3.0966733	up	-1.55993	down	2.184006	up	1.985131	up
Os.5562.1.S1_at	1.5208536	up	1.367816	up	1.023454	up	2.080248	up
Os.53870.1.A1_at	-1.1657758	down	-1.2264	down	1.716203	up	-1.42971	down
Os.15936.1.S1_at	-2.0928025	down	-1.00079	down	-1.39793	down	-2.09445	down
Os.6122.1.S1_at	-1.6495515	down	-1.27724	down	-1.09519	down	-2.10687	down
Os.21445.1.S1_at	-2.3167107	down	-1.11039	down	-1.25921	down	-2.57246	down
Os.51085.1.S1_at	-2.3808792	down	2.060761	up	-2.88106	down	-1.15534	down
Os.16912.1.A1_at	-2.3825893	down	-1.17003	down	-1.19445	down	-2.7877	down
Os.11693.1.S1_at	-2.2644386	down	1.339083	up	-1.87137	down	-1.69104	down
Os.51185.2.S1_x_at	-3.0285006	down	1.428693	up	-1.99567	down	-2.11977	down
Os.17052.2.S1_at	-2.0308492	down	-1.04054	down	-1.34189	down	-2.11318	down
Os.22415.1.S1_s_at	-2.0648398	down	1.15378	up	-1.61091	down	-1.78963	down
Os.15748.1.A1_a_at	-2.3831034	down	-1.09544	down	-1.2744	down	-2.61055	down
Os.11557.1.S1_at	-1.9925717	down	2.175783	up	-3.03743	down	1.091947	up
Os.53553.1.S1_at	-1.8368915	down	-1.09538	down	-1.27442	down	-2.01209	down
Os.37062.1.S1_at	-1.1018687	down	2.424345	up	-1.73668	down	2.200212	up
Os.14411.1.S1_at	1.6781096	up	6.576436	up	-4.71266	down	11.03598	up
Os.11756.1.S1_at	-1.28613	down	1.633088	up	-2.27826	down	1.269769	up
OsAffx.26840.1.S1_s_	-2.9709034	down	-1.11229	down	-1.25386	down	-3.3045	down
Os.12788.1.S1_at	-1.530452	down	-1.68899	down	1.211058	up	-2.58492	down
Os.43040.1.S1_x_at	2.247469	up	1.100777	up	1.266817	up	2.473962	up
Os.52224.1.S1_at	-1.2807568	down	1.527241	up	-2.12956	down	1.192452	up
Os.4244.1.S1_at	2.1563025	up	1.021222	up	1.365382	up	2.202063	up
Os.56083.1.S1_at	-2.996651	down	1.573888	up	-2.1945	down	-1.90398	down
Os.49296.1.S1_at	-1.5605996	down	-1.56816	down	1.125025	up	-2.44727	down
Os.7051.1.S1_at	12.1342535	up	-6.46462	down	9.010881	up	1.877024	up
Os.27194.1.A1_a_at	-1.8645627	down	1.468656	up	-2.04701	down	-1.26957	down
Os.27322.1.S1_x_at	-2.1401103	down	1.166649	up	-1.6259	down	-1.83441	down
Os.38312.1.S1_at	-2.6488247	down	1.344408	up	-1.87347	down	-1.97025	down

Os.46613.1.S1_s_at	1.1020501	up	1.442424	up	-2.00944	down	1.589623	up
Os.51773.1.S1_at	-1.8040475	down	-1.13059	down	-1.23192	down	-2.03964	down
Os.26059.1.S1_s_at	-2.034063	down	1.228714	up	-1.71124	down	-1.65544	down
Os.22910.1.S1_at	1.1125604	up	1.94055	up	-2.70217	down	2.158979	up
Os.4155.1.S1_at	1.0534134	up	-2.3931	down	1.718827	up	-2.27175	down
Os.40036.1.A1_at	-1.5229443	down	1.600739	up	-2.22794	down	1.051081	up
Os.49367.1.S1_a_at	-1.9583192	down	-1.21611	down	-1.14444	down	-2.38154	down
Os.54587.1.S1_at	-1.9792275	down	1.557398	up	-2.16741	down	-1.27086	down
Os.17377.1.S1_at	-2.172181	down	1.390427	up	-1.93502	down	-1.56224	down
Os.14872.1.S1_at	-2.5122511	down	1.818512	up	-2.53074	down	-1.38149	down
Os.49808.1.S2_at	-2.3069332	down	1.6657	up	-2.31777	down	-1.38496	down
Os.53888.1.S1_at	-2.0537808	down	-1.24031	down	-1.12183	down	-2.54733	down
OsAffx.3623.1.S1_at	-2.2848477	down	1.048867	up	-1.45932	down	-2.1784	down
Os.52977.1.S1_at	-2.637505	down	1.261124	up	-1.75448	down	-2.09139	down
Os.8477.1.S1_a_at	1.4426441	up	2.029046	up	-1.45864	down	2.927192	up
Os.26884.1.S1_a_at	-2.3258388	down	3.502098	up	-2.51774	down	1.505735	up
Os.24850.1.S1_at	-2.0791602	down	-1.2916	down	-1.07679	down	-2.68545	down
Os.22832.1.S1_at	-2.0331473	down	-1.20875	down	-1.15059	down	-2.45757	down
Os.52844.1.S1_at	-2.368629	down	1.440458	up	-2.00302	down	-1.64436	down
Os.10662.1.S1_at	1.2903496	up	1.808916	up	-1.30088	down	2.334134	up
Os.52944.1.A1_at	-2.363785	down	3.745804	up	-2.69381	down	1.584663	up
OsAffx.11044.1.S1_x_	2.1294322	up	1.000976	up	1.389155	up	2.131511	up
Os.18941.1.S1_at	-2.3880472	down	1.188389	up	-1.65221	down	-2.00948	down
Os.9011.1.S1_at	-1.75259	down	-1.15966	down	-1.19875	down	-2.0324	down
Os.7515.1.S1_s_at	-1.9255732	down	-1.09936	down	-1.26438	down	-2.1169	down
Os.35907.1.S1_at	-1.6385729	down	1.446334	up	-2.01039	down	-1.13291	down
Os.23364.2.S1_a_at	-1.7256156	down	-1.23777	down	-1.12281	down	-2.13592	down
Os.11707.1.A1_at	-1.7699631	down	1.672884	up	-2.32401	down	-1.05803	down
Os.19849.1.S1_at	1.0363626	up	1.688178	up	-2.345	down	1.749564	up
Os.47929.1.S1_at	-1.4516746	down	1.708683	up	-2.37332	down	1.177042	up
Os.46525.2.S1_x_at	-2.0787709	down	1.021078	up	-1.4179	down	-2.03586	down
Os.11601.1.S1_at	-3.1388423	down	1.406318	up	-1.95242	down	-2.23196	down
Os.52421.1.S1_at	-2.4416838	down	1.046263	up	-1.45225	down	-2.33372	down
Os.9264.1.S1_at	-2.2219672	down	1.195679	up	-1.65956	down	-1.85833	down
Os.7101.1.S1_at	1.2548312	up	2.877553	up	-3.9925	down	3.610843	up
Os.38760.1.S1_at	-3.5645015	down	1.656264	up	-1.19384	down	-2.15213	down
Os.52476.1.S2_at	-1.6367716	down	1.462999	up	-2.0292	down	-1.11878	down
Os.52644.1.S1_at	-1.5007186	down	2.464781	up	-1.77751	down	1.642401	up
Os.27107.1.S1_at	-2.4967554	down	1.530364	up	-2.12204	down	-1.63148	down
Os.27673.1.S1_at	1.8698916	up	1.707884	up	-1.23183	down	3.193558	up
Os.5583.1.S1_at	-1.1041455	down	3.038623	up	-2.19203	down	2.752013	up
Os.50460.1.S1_at	-2.0032132	down	1.02926	up	-1.42641	down	-1.94627	down
Os.9429.1.S1_at	-2.4671612	down	1.126093	up	-1.56052	down	-2.1909	down
Os.10594.1.S1_at	-1.4736389	down	-1.3712	down	-1.01051	down	-2.02065	down
Os.51759.2.S1_s_at	-2.0757265	down	1.070373	up	-1.48299	down	-1.93926	down
OsAffx.12146.1.S1_at	1.3759369	up	2.344227	up	-1.69239	down	3.225509	up
Os.27826.1.S1_at	1.2334623	up	2.088967	up	-1.50904	down	2.576662	up

Os.11782.1.S1_at	2.1484246	up	-1.04825	down	1.450998	up	2.049542	up
Os.46528.1.S1_at	-2.4970484	down	1.230241	up	-1.70287	down	-2.02972	down
Os.17435.1.S1_x_at	-2.4200816	down	1.244119	up	-1.72206	down	-1.94522	down
Os.18381.1.S1_at	1.0877395	up	-3.0422	down	4.210739	up	-2.79681	down
Os.5808.1.S1_at	-1.4045249	down	2.354166	up	-3.25781	down	1.67613	up
Os.52314.1.S2_at	-2.3295333	down	1.211635	up	-1.6767	down	-1.92264	down
Os.33747.1.S1_at	1.1158324	up	-1.48465	down	2.054461	up	-1.33053	down
Os.29745.1.S2_at	-2.9607642	down	-1.50883	down	1.090401	up	-4.4673	down
Os.8195.1.S1_at	2.1419907	up	-1.45356	down	2.01099	up	1.473621	up
Os.27316.1.S1_at	-2.1884909	down	1.014641	up	-1.40362	down	-2.15691	down
Os.7614.1.S1_at	1.174925	up	2.10549	up	-1.52204	down	2.473794	up
Os.17676.1.S1_at	-2.0203216	down	-1.04922	down	-1.31772	down	-2.11977	down
Os.42024.1.S1_at	-2.9759028	down	2.253659	up	-3.11567	down	-1.32048	down
Os.17211.1.S1_at	-1.9207178	down	-1.18657	down	1.639936	up	-2.27906	down
Os.54221.1.A1_at	-1.5934917	down	-1.32606	down	-1.04216	down	-2.11307	down
Os.16047.1.S1_at	-2.111012	down	1.236046	up	-1.70806	down	-1.70787	down
Os.36919.1.S1_x_at	-1.0881529	down	2.090231	up	-1.51339	down	1.920898	up
Os.20702.1.S1_at	-1.2080575	down	1.551263	up	-2.14238	down	1.284097	up
Os.17405.1.S1_a_at	-1.6506504	down	-3.19798	down	2.315794	up	-5.27874	down
Os.5797.1.S1_at	1.0267332	up	1.548136	up	-2.13746	down	1.589522	up
Os.49525.1.S1_at	1.812381	up	1.670757	up	-1.21023	down	3.028047	up
OsAffx.29066.1.S1_at	-1.5197303	down	-1.53083	down	1.108936	up	-2.32645	down
Os.16024.1.S1_at	-1.4604038	down	-1.56285	down	1.132157	up	-2.2824	down
Os.46639.1.A1_x_at	-2.0646665	down	1.133596	up	-1.56476	down	-1.82134	down
OsAffx.26361.1.S1_at	1.6714264	up	1.651634	up	-1.19655	down	2.760584	up
Os.8937.1.S1_at	-2.0984194	down	-1.36302	down	-1.01254	down	-2.86019	down
Os.49387.1.S1_at	-1.9920719	down	-1.14276	down	-1.20758	down	-2.27647	down
Os.26054.1.S1_s_at	-1.463051	down	2.445388	up	-1.77215	down	1.671431	up
OsAffx.27130.1.S1_at	1.3067658	up	1.744569	up	-1.26434	down	2.279743	up
Os.48053.1.A1_at	5.1726995	up	-103.899	down	143.3569	up	-20.0861	down
Os.48200.2.S1_at	-1.6108425	down	-1.36877	down	-1.00801	down	-2.20487	down
Os.48109.1.A1_x_at	1.0505476	up	1.463056	up	-2.0181	down	1.53701	up
Os.8175.1.S1_at	1.2308394	up	1.626725	up	-1.17943	down	2.002238	up
Os.46372.1.A1_at	-1.4046258	down	1.639105	up	-2.2607	down	1.166933	up
Os.2325.1.S1_at	1.1752157	up	3.005097	up	-2.17907	down	3.531637	up
Os.49422.1.S1_x_at	-2.5540164	down	-1.14501	down	-1.20429	down	-2.92438	down
Os.17961.1.S1_a_at	3.625102	up	4.538509	up	-3.29242	down	16.45256	up
Os.7481.1.S1_at	-1.5290998	down	1.573389	up	-2.16865	down	1.028964	up
Os.17730.2.S1_at	1.5043216	up	1.560315	up	-1.13205	down	2.347215	up
Os.23348.1.S1_at	2.6294801	up	-1.59161	down	2.193287	up	1.652092	up
Os.49109.1.S2_at	-2.2467535	down	2.223626	up	-3.06414	down	-1.0104	down
Os.197.2.S1_x_at	-1.7471997	down	1.686071	up	-2.32257	down	-1.03626	down
Os.1344.1.S1_at	1.4983536	up	2.181881	up	-1.584	down	3.26923	up
Os.50360.1.S1_at	1.6392919	up	1.918271	up	-1.39268	down	3.144606	up
Os.9220.1.S1_at	-1.0820391	down	-1.4007	down	1.928912	up	-1.51561	down
Os.213.1.S1_at	1.5470194	up	1.393454	up	-1.01192	down	2.155701	up
Os.12637.1.S1_at	-2.5775495	down	1.739808	up	-1.26351	down	-1.48151	down

Os.16494.1.S1_at	-1.184199	down	-1.74499	down	1.268114	up	-2.06641	down
Os.1597.1.S1_at	2.2245615	up	-1.17914	down	1.622464	up	1.886599	up
Os.11407.1.S1_at	2.1642978	up	1.379097	up	-1.00238	down	2.984776	up
Os.4892.1.S1_at	1.3305016	up	-1.65727	down	2.279933	up	-1.2456	down
Os.49475.1.S1_at	-1.6887312	down	-2.29194	down	1.66609	up	-3.87046	down
Os.27979.1.S1_at	-1.8823307	down	-1.15145	down	-1.19466	down	-2.16741	down
Os.6258.1.S1_a_at	-1.6889646	down	1.54635	up	-2.12695	down	-1.09223	down
Os.6229.1.S1_at	-1.8350804	down	-1.17196	down	-1.17358	down	-2.15065	down
Os.11193.1.S1_at	-2.4464877	down	1.856776	up	-2.55317	down	-1.3176	down
Os.37413.1.S1_at	-1.6039474	down	-1.38805	down	1.009466	up	-2.22636	down
Os.49694.1.S1_at	-1.6763464	down	-1.26909	down	-1.08342	down	-2.12744	down
Os.49288.1.S1_at	-4.026811	down	1.861156	up	-2.55881	down	-2.16361	down
Os.10312.1.S1_at	2.1821628	up	-1.30076	down	1.78802	up	1.677611	up
Os.2695.1.S1_at	2.0884194	up	-1.33934	down	1.840693	up	1.559295	up
Os.23454.1.A1_at	2.2018855	up	-1.04288	down	1.433253	up	2.111359	up
Os.10792.1.S1_at	-2.1751611	down	1.19602	up	-1.64371	down	-1.81867	down
OsAffx.27559.1.S1_x_	1.7850113	up	1.121285	up	1.224998	up	2.001506	up
OsAffx.14519.1.S1_s_	1.5537056	up	-1.83589	down	2.521482	up	-1.18162	down
Os.24890.1.S2_at	1.4949955	up	1.436853	up	-1.04628	down	2.148089	up
OsAffx.30872.1.S1_s_	-2.2794383	down	3.06261	up	-4.20495	down	1.343581	up
Os.15204.2.S1_x_at	-1.4563076	down	1.747316	up	-2.39819	down	1.199826	up
Os.48758.1.A1_s_at	1.5989877	up	-2.06003	down	1.501201	up	-1.28833	down
Os.49500.1.S1_at	-1.1561105	down	2.591974	up	-1.88889	down	2.241978	up
Os.11773.1.S1_at	-1.5548003	down	1.696033	up	-2.32727	down	1.090837	up
Os.17762.1.S1_at	-3.670885	down	3.061886	up	-2.23158	down	-1.1989	down
Os.10219.1.S1_at	-1.9843332	down	-1.08222	down	-1.26783	down	-2.14748	down
Os.11216.1.S1_at	1.244909	up	2.088725	up	-1.52247	down	2.600272	up
Os.23158.1.S1_at	1.1072553	up	2.814363	up	-2.05195	down	3.116218	up
Os.27845.1.S1_x_at	-2.3239052	down	1.169083	up	-1.60342	down	-1.9878	down
Os.18003.1.S1_s_at	1.6770971	up	1.247269	up	1.099494	up	2.091791	up
Os.30747.1.S1_at	-2.5969667	down	1.258947	up	-1.72584	down	-2.06281	down
Os.11957.1.S1_at	-2.347213	down	1.056577	up	-1.44832	down	-2.22153	down
Os.37652.2.S1_at	-1.1286628	down	-1.31722	down	1.805592	up	-1.4867	down
Os.6428.1.S1_at	-1.4759104	down	-1.79015	down	1.306493	up	-2.6421	down
Os.15988.1.S1_at	1.5222534	up	-1.66261	down	2.277997	up	-1.0922	down
Os.7969.1.S1_x_at	-1.6775262	down	2.628953	up	-3.60189	down	1.567161	up
Os.53775.1.S1_at	-2.0438504	down	1.440404	up	-1.97333	down	-1.41894	down
Os.17306.1.S1_at	-1.8812212	down	-1.1287	down	-1.21331	down	-2.12333	down
OsAffx.13943.1.S1_at	-1.324629	down	-1.54933	down	1.131534	up	-2.05229	down
Os.8555.1.S1_at	1.9928249	up	2.22576	up	-1.62563	down	4.435551	up
OsAffx.16441.1.S1_at	-2.9185312	down	1.114431	up	-1.52558	down	-2.61885	down
Os.26780.1.S1_at	-2.190412	down	-1.08802	down	-1.25809	down	-2.3832	down
Os.52134.1.S1_at	-2.081095	down	-1.14274	down	-1.19777	down	-2.37816	down
Os.33968.1.S1_at	1.7318547	up	-2.99523	down	4.099639	up	-1.72949	down
Os.33614.1.S1_at	-3.2717304	down	1.433949	up	-1.96249	down	-2.28162	down
Os.53349.1.S1_at	1.6389544	up	2.250808	up	-1.64465	down	3.688973	up
OsAffx.26276.1.S1_at	-1.1412712	down	-3.89965	down	5.335691	up	-4.45056	down

Os.16733.1.S1_at	1.0319867	up	2.301435	up	-1.68212	down	2.375051	up
Os.13996.1.S1_at	1.5515838	up	1.882739	up	-1.37618	down	2.921227	up
OsAffx.30830.1.S1_at	1.6332284	up	-1.5717	down	2.149856	up	1.039148	up
Os.23521.1.A1_at	-2.2067013	down	1.154066	up	-1.57852	down	-1.91211	down
Os.22994.1.S1_at	-2.62384	down	1.059858	up	-1.44962	down	-2.47565	down
OsAffx.17259.1.S1_s_	2.6558275	up	-2.43843	down	1.782932	up	1.089155	up
Os.52517.1.S1_at	-2.2174504	down	1.138601	up	-1.55658	down	-1.94752	down
Os.16743.3.S1_at	-1.2571858	down	-1.43801	down	1.965359	up	-1.80784	down
OsAffx.30004.1.S1_x_	1.2061696	up	1.78994	up	-1.30969	down	2.158972	up
Os.53167.1.S1_at	-1.2135832	down	-1.22139	down	1.669242	up	-1.48226	down
Os.3409.1.S1_at	2.421511	up	-1.83156	down	2.502746	up	1.322102	up
Os.9808.1.S1_at	1.9665751	up	1.241108	up	1.100816	up	2.440732	up
Os.37127.1.A1_at	-1.8198327	down	-1.19358	down	-1.14397	down	-2.17211	down
Os.22697.1.S1_at	-1.5370127	down	-1.30482	down	-1.04623	down	-2.00552	down
Os.3427.1.S1_at	1.8709958	up	-1.58396	down	2.161753	up	1.181217	up
Os.50635.1.S1_at	-1.4943221	down	3.307579	up	-2.42356	down	2.213431	up
Os.18412.2.S1_x_at	2.0219944	up	-1.83905	down	2.50952	up	1.099476	up
OsAffx.2784.1.S1_s_a	-2.5506592	down	2.23995	up	-3.05451	down	-1.13871	down
OsAffx.7567.1.A1_at	2.8969934	up	-1.73264	down	2.362535	up	1.672007	up
Os.16682.1.S1_at	-2.0970542	down	1.042068	up	-1.42082	down	-2.0124	down
Os.26472.1.S1_at	1.4162669	up	2.650076	up	-1.94406	down	3.753215	up
Os.51034.2.S1_at	-1.4797428	down	-1.46172	down	1.072422	up	-2.16297	down
Os.9616.1.S1_at	1.1909108	up	1.513465	up	-2.06262	down	1.802402	up
Os.47503.1.S1_at	-2.1786609	down	-1.34115	down	-1.01604	down	-2.92191	down
Os.49346.1.S1_at	-3.3111877	down	1.40936	up	-1.91977	down	-2.34943	down
Os.11629.1.S1_at	1.6668172	up	1.602856	up	-1.17708	down	2.671669	up
OsAffx.13616.1.S1_x_	-1.8337834	down	-1.17223	down	-1.16094	down	-2.14962	down
Os.28949.1.S1_at	-2.5622268	down	1.936278	up	-2.63477	down	-1.32327	down
Os.25255.1.S1_at	-2.1239276	down	1.219776	up	-1.65932	down	-1.74124	down
Os.46093.1.S1_at	-5.846602	down	4.338939	up	-5.90204	down	-1.34747	down
Os.17881.1.S1_at	1.5052513	up	1.352198	up	1.005925	up	2.035398	up
Os.8495.1.S1_at	-2.1221278	down	1.407921	up	-1.91491	down	-1.50728	down
Os.52493.1.A1_at	-1.144004	down	1.95565	up	-2.6598	down	1.709478	up
Os.53852.1.S1_at	-1.3037218	down	-1.79057	down	1.316635	up	-2.33441	down
Os.5182.1.S1_at	1.6606241	up	1.772375	up	-1.3033	down	2.943249	up
Os.18061.1.S1_at	-2.0464928	down	-1.03952	down	-1.30792	down	-2.12737	down
Os.48039.1.A1_at	-1.9405074	down	-1.03253	down	-1.31673	down	-2.00363	down
Os.11387.1.S1_a_at	-1.9234432	down	-1.09448	down	-1.24216	down	-2.10517	down
Os.5255.1.S1_at	-1.8038509	down	1.613292	up	-2.19202	down	-1.11812	down
Os.27473.1.S1_at	-1.786977	down	-1.21897	down	-1.11445	down	-2.17827	down
Os.53717.1.S1_at	-1.0966858	down	2.881072	up	-2.1209	down	2.627072	up
Os.7731.1.S1_at	-1.8788043	down	2.269256	up	-3.08255	down	1.207819	up
OsAffx.4511.1.S1_s_a	-2.8177679	down	3.543133	up	-4.81099	down	1.257425	up
Os.11990.1.S2_at	-1.2155342	down	2.189959	up	-1.61334	down	1.801643	up
Os.11972.1.S1_at	-1.5606076	down	-1.29035	down	-1.05183	down	-2.01373	down
Os.32556.1.S1_at	-1.2952757	down	-1.61293	down	1.188704	up	-2.08919	down
Os.8667.1.S1_at	2.459571	up	-1.09785	down	1.489595	up	2.24036	up

Os.8244.1.S1_at	1.7636974	up	1.777024	up	-1.30978	down	3.134132	up
Os.26991.1.S1_x_at	-1.7620498	down	1.514289	up	-2.05383	down	-1.16362	down
Os.12425.1.S1_at	1.8846514	up	1.285716	up	1.054753	up	2.423125	up
Os.42759.1.S1_at	-1.6723019	down	-1.23597	down	-1.0971	down	-2.06691	down
Os.39427.1.A1_s_at	-1.8464614	down	-1.17694	down	-1.15209	down	-2.17318	down
Os.17111.2.S1_x_at	-2.026783	down	-1.02137	down	-1.32737	down	-2.0701	down
Os.26282.1.S1_at	3.2262704	up	-1.42793	down	1.935829	up	2.259402	up
Os.42801.1.S1_at	-1.918177	down	-1.15939	down	1.571369	up	-2.22392	down
Os.53185.1.S1_at	-2.1683002	down	1.024135	up	-1.38792	down	-2.1172	down
Os.11002.1.S1_at	-1.1387063	down	1.823864	up	-2.47131	down	1.601698	up
OsAffx.28664.1.S1_at	-2.0470514	down	1.462145	up	-1.98086	down	-1.40003	down
Os.3640.1.S1_at	-2.1664498	down	1.110276	up	-1.50376	down	-1.95127	down
Os.5587.1.S1_at	1.3468286	up	-1.58215	down	2.142853	up	-1.17473	down
Os.3415.1.S1_s_at	2.5394473	up	-2.42566	down	3.284655	up	1.046908	up
Os.361.1.S1_at	1.3967924	up	1.531929	up	-1.13145	down	2.139786	up
Os.38278.2.S1_x_at	-1.5462351	down	-1.49674	down	1.105535	up	-2.31431	down
Os.3754.1.S1_at	-1.6448823	down	-1.31776	down	-1.02737	down	-2.16757	down
Os.16952.1.S1_at	1.1097342	up	1.613985	up	-2.18506	down	1.791095	up
Os.18453.1.S1_at	-2.0476	down	1.052978	up	-1.42547	down	-1.94458	down
Os.40007.1.S1_x_at	4.8183303	up	-2.59012	down	3.505571	up	1.860272	up
Os.7119.1.S1_at	-1.9696466	down	-1.09716	down	-1.23313	down	-2.16102	down
Os.46566.1.S1_at	2.0857828	up	-1.19786	down	1.620576	up	1.741262	up
Os.37866.1.S1_at	2.1186442	up	1.356583	up	-1.00288	down	2.874116	up
OsAffx.11050.1.S1_x_	1.6291693	up	1.40125	up	-1.03618	down	2.282874	up
Os.15076.1.S1_at	-1.3760233	down	-1.67567	down	1.239126	up	-2.30576	down
Os.1148.1.S1_at	1.3301905	up	1.66177	up	-1.22885	down	2.21047	up
Os.14978.1.S1_a_at	-1.6389921	down	-1.92256	down	1.422155	up	-3.15105	down
Os.35772.3.S1_at	-3.5836713	down	1.760689	up	-2.38018	down	-2.03538	down
OsAffx.21742.1.S1_at	1.3331971	up	-2.10471	down	1.556979	up	-1.57869	down
Os.10577.1.S1_x_at	-2.150758	down	1.914396	up	-2.58771	down	-1.12347	down
Os.52837.1.S1_x_at	-1.6475033	down	-1.25069	down	-1.08047	down	-2.06052	down
Os.56991.1.S1_at	1.2718428	up	-2.41774	down	1.789214	up	-1.90097	down
Os.2901.1.S1_at	-3.0485337	down	1.909156	up	-2.57959	down	-1.5968	down
Os.26502.1.S1_a_at	1.3206804	up	-2.38279	down	1.763918	up	-1.80422	down
Os.7556.1.S1_at	-2.2338479	down	1.970156	up	-2.66089	down	-1.13384	down
Os.35316.1.S1_at	-2.391396	down	-1.19596	down	-1.12918	down	-2.86002	down
Os.53279.1.S1_at	1.4773456	up	1.572197	up	-1.16422	down	2.322679	up
Os.53840.1.S1_at	-2.3743334	down	1.184034	up	-1.59888	down	-2.00529	down
Os.4183.1.S1_a_at	1.4886427	up	1.607858	up	-1.19074	down	2.393526	up
Os.56310.1.A1_at	-2.0984325	down	1.646432	up	-1.21957	down	-1.27453	down
Os.13960.1.S1_at	-2.1847556	down	-1.09957	down	-1.22749	down	-2.4023	down
Os.17332.1.S1_at	-2.3012302	down	1.210662	up	1.114835	up	-1.9008	down
Os.8178.1.S1_at	1.9713799	up	-2.20106	down	2.970644	up	-1.11651	down
Os.6496.1.S1_a_at	-1.8291285	down	-1.21084	down	-1.11446	down	-2.21478	down
Os.11058.1.S1_x_at	1.7471663	up	1.428936	up	-1.05939	down	2.496589	up
Os.49190.1.S1_at	-2.1936493	down	1.15731	up	-1.56043	down	-1.89547	down
Os.11122.1.S1_at	-2.215722	down	-1.41073	down	1.046603	up	-3.12578	down

Os.21875.1.S1_at	-1.6303015	down	-1.3426	down	-1.00392	down	-2.18885	down
Os.10104.1.S1_at	-1.5556858	down	-1.30406	down	-1.03353	down	-2.02871	down
Os.11910.1.S1_at	-1.4065412	down	-1.07668	down	1.451053	up	-1.51439	down
OsAffx.11451.6.S1_s_	-2.1830955	down	1.16918	up	-1.57567	down	-1.8672	down
OsAffx.19483.1.S1_s_	-1.367905	down	-1.49037	down	1.106134	up	-2.03869	down
Os.37729.1.S1_s_at	3.0764496	up	-1.48834	down	2.005169	up	2.06703	up
OsAffx.11854.1.S1_x_	2.0852375	up	-1.63146	down	2.197861	up	1.278141	up
Os.37820.1.S1_x_at	1.3327931	up	-2.03724	down	2.744191	up	-1.52855	down
Os.9226.1.S1_a_at	1.0967014	up	1.434339	up	-1.93186	down	1.573042	up
Os.57475.1.S1_x_at	2.5341833	up	-2.07191	down	2.79049	up	1.223112	up
Os.1577.1.S1_at	1.5739888	up	1.371644	up	-1.01858	down	2.158952	up
Os.19512.3.S1_x_at	-2.525331	down	1.097143	up	-1.47742	down	-2.30173	down
Os.3577.2.S1_at	-2.0729597	down	1.272074	up	-1.71281	down	-1.62959	down
OsAffx.2947.1.S1_at	1.0869682	up	-13.4426	down	18.09686	up	-12.3671	down
Os.25556.1.S1_x_at	1.3040332	up	4.218241	up	-5.6786	down	5.500726	up
Os.38687.1.S1_at	-2.225133	down	1.316344	up	-1.77182	down	-1.69039	down
Os.27285.1.S1_at	-2.2659366	down	1.180902	up	-1.58947	down	-1.91882	down
Os.52951.1.S1_at	-1.9587443	down	-1.13597	down	-1.1848	down	-2.22507	down
Os.47876.1.A1_x_at	1.5011586	up	2.000694	up	-1.48658	down	3.00336	up
Os.2369.1.S1_a_at	-1.3749933	down	-1.73735	down	2.337949	up	-2.38884	down
Os.15813.1.S1_at	2.8848639	up	-1.0439	down	1.404621	up	2.763535	up
Os.17034.1.S1_at	1.7053683	up	1.348823	up	-1.0026	down	2.30024	up
OsAffx.6331.1.S1_at	-2.7255213	down	2.574	up	-3.46255	down	-1.05887	down
Os.11915.1.S1_at	2.9586651	up	-2.7041	down	3.637528	up	1.09414	up
OsAffx.11075.1.S1_at	-2.5186472	down	1.71173	up	-2.30075	down	-1.4714	down
Os.17420.1.S1_at	-2.0531094	down	-1.26322	down	-1.064	down	-2.59352	down
Os.54231.1.S1_at	-1.8724191	down	-1.09353	down	-1.22905	down	-2.04756	down
Os.24866.1.A1_at	-2.0499465	down	-1.01673	down	-1.32172	down	-2.08424	down
Os.8017.1.S1_at	1.8389891	up	-1.81554	down	2.43951	up	1.012913	up
OsAffx.24703.1.S1_s_	1.4071989	up	-1.75772	down	2.361339	up	-1.24909	down
Os.54008.1.S1_at	-2.1129088	down	1.251884	up	-1.68161	down	-1.68778	down
Os.27187.1.S1_at	-2.3603349	down	-1.09299	down	-1.22874	down	-2.57982	down
Os.54307.1.S1_at	-3.1646206	down	1.166816	up	-1.56666	down	-2.71219	down
Os.54430.1.S1_at	1.1317054	up	4.795359	up	-3.57179	down	5.426934	up
Os.10149.1.S1_x_at	-2.0353541	down	1.39795	up	-1.87671	down	-1.45596	down
OsAffx.27980.1.S1_at	-1.8015534	down	1.617491	up	-2.17077	down	-1.11379	down
Os.10860.2.S1_at	-1.4736298	down	1.563192	up	-2.09777	down	1.060777	up
Os.53124.1.S1_at	-1.1521386	down	-1.78789	down	1.332493	up	-2.0599	down
Os.35524.1.S2_at	-2.8218796	down	1.924187	up	-2.5817	down	-1.46653	down
Os.12014.1.S1_s_at	-1.3990031	down	-1.48212	down	1.104709	up	-2.0735	down
Os.20516.1.S1_at	-2.7186024	down	2.426676	up	-1.80877	down	-1.1203	down
Os.24911.1.S1_at	-2.88696	down	1.057496	up	-1.41842	down	-2.73	down
Os.8447.1.S1_at	-2.1265988	down	-1.33612	down	-1.00333	down	-2.8414	down
Os.48217.1.A1_at	-1.6540511	down	2.292444	up	-1.71009	down	1.385957	up
Os.11719.1.S1_at	1.1501997	up	2.095191	up	-1.56303	down	2.409888	up
Os.9515.1.S1_at	-2.3037632	down	-1.23762	down	1.658765	up	-2.85118	down
Os.31975.1.S1_at	6.3247547	up	-5.02268	down	6.729147	up	1.25924	up

Os.17414.1.S1_at	-2.3991606	down	1.058812	up	-1.41853	down	-2.2659	down
Os.36597.1.S1_at	-2.5960026	down	1.022485	up	-1.36981	down	-2.53891	down
OsAffx.18790.1.S1_at	1.7392664	up	1.409602	up	-1.05237	down	2.451674	up
OsAffx.27153.2.S1_s_	-1.0965406	down	2.166704	up	-1.61784	down	1.975945	up
Os.7016.1.S1_at	1.046943	up	1.533432	up	-2.05353	down	1.605416	up
Os.9681.1.S1_at	2.08955	up	1.927104	up	-1.43927	down	4.02678	up
Os.6767.1.S1_at	-2.2836506	down	2.158743	up	-2.89023	down	-1.05786	down
Os.9262.1.S1_at	1.7663383	up	1.73557	up	-1.29661	down	3.065604	up
Os.47303.1.S1_at	-1.0558724	down	-7.56319	down	10.12274	up	-7.98576	down
Os.4624.1.S1_at	-1.5801618	down	-1.39662	down	1.0437	up	-2.20689	down
Os.18825.1.S1_at	-2.286189	down	1.302614	up	-1.7427	down	-1.75508	down
Os.47660.1.S1_s_at	-1.4655998	down	-1.92734	down	1.440909	up	-2.8247	down
Os.16004.1.S1_at	-2.2053745	down	1.028338	up	-1.37539	down	-2.1446	down
Os.22594.1.S1_at	1.8100731	up	-2.43107	down	1.817735	up	-1.34308	down
Os.14573.1.S1_at	-1.865115	down	1.661979	up	-2.22219	down	-1.12223	down
Os.11920.1.S1_s_at	-2.889554	down	3.283123	up	-4.38942	down	1.136204	up
Os.17143.1.S1_at	-2.3142242	down	1.360734	up	-1.81886	down	-1.70072	down
Os.37320.2.S1_x_at	-1.4525809	down	-1.0566	down	1.412291	up	-1.5348	down
Os.55569.1.S1_at	-1.5727519	down	-1.41231	down	1.056665	up	-2.22122	down
OsAffx.28288.1.S1_at	-3.2399085	down	-1.06414	down	-1.25585	down	-3.44771	down
Os.13743.1.S1_at	1.494324	up	1.666708	up	-1.2474	down	2.490602	up
Os.28976.1.S1_at	-1.6139168	down	1.622592	up	-2.1678	down	1.005375	up
Os.50844.1.S1_at	-1.7415317	down	-1.04652	down	1.397966	up	-1.82254	down
Os.49157.1.S1_s_at	-1.1265562	down	-3.74017	down	4.996066	up	-4.21351	down
Os.53166.1.S1_at	-1.5636857	down	1.778131	up	-2.3752	down	1.137141	up
Os.27111.1.S1_at	-1.5350822	down	-1.48152	down	1.109349	up	-2.27426	down
Os.49843.1.S1_at	-2.6204643	down	1.277791	up	-1.70645	down	-2.05078	down
Os.54675.1.S1_x_at	-2.0467727	down	1.197932	up	-1.59977	down	-1.70859	down
Os.52990.2.S1_at	-1.2772177	down	2.072972	up	-1.55259	down	1.623037	up
Os.27288.2.S1_x_at	-1.5783125	down	1.534325	up	-2.04722	down	-1.02867	down
Os.10701.1.S1_at	1.0705295	up	2.055074	up	-1.54048	down	2.200017	up
Os.41980.1.S1_at	-2.3398266	down	1.121423	up	-1.49601	down	-2.08648	down
Os.4875.1.S1_at	-1.5076578	down	-2.10548	down	1.578662	up	-3.17434	down
Os.46453.1.S1_a_at	1.851096	up	1.160796	up	1.148669	up	2.148745	up
Os.21083.1.S1_at	-2.0908704	down	1.03095	up	-1.37444	down	-2.0281	down
Os.15781.1.S1_at	-1.7153097	down	1.092543	up	1.220176	up	-1.57002	down
Os.52507.1.S1_at	-1.9043745	down	1.628947	up	-2.1715	down	-1.16908	down
Os.3451.1.S1_at	-1.3151081	down	1.534434	up	-2.04478	down	1.166774	up
Os.48856.1.S1_at	2.9406176	up	1.346656	up	-1.01067	down	3.960002	up
Os.8109.1.S2_a_at	-1.7269422	down	-1.34112	down	1.006692	up	-2.31604	down
Os.9751.1.S1_at	-2.3105533	down	-1.08737	down	-1.22479	down	-2.51243	down
Os.18739.1.S1_at	-2.5236273	down	1.279859	up	-1.70424	down	-1.9718	down
Os.7676.1.S1_at	-1.5968229	down	2.081091	up	-2.77108	down	1.30327	up
Os.23413.1.A1_at	2.0225155	up	-1.41494	down	1.883895	up	1.429397	up
Os.3769.1.S1_a_at	-1.5648384	down	-1.34409	down	1.010118	up	-2.10328	down
OsAffx.26738.1.S1_at	-1.1131151	down	1.594556	up	-2.12148	down	1.432516	up
Os.21387.3.S1_at	-1.4563996	down	2.083546	up	-1.56615	down	1.430615	up

Os.7535.1.S1_at	2.2322192	up	-4.50787	down	3.388886	up	-2.01945	down
Os.11262.1.S1_a_at	1.9078255	up	1.273162	up	1.044469	up	2.428972	up
Os.21379.1.S1_at	-1.166509	down	2.178469	up	-1.63822	down	1.867511	up
OsAffx.5891.1.S1_s_a	-1.5380139	down	-1.46547	down	1.102226	up	-2.25391	down
Os.53486.1.S1_at	1.0089527	up	-1.93717	down	2.575505	up	-1.91998	down
Os.12253.1.S1_at	-2.980098	down	2.755614	up	-3.66315	down	-1.08146	down
Os.28098.2.A1_at	2.389247	up	-2.06136	down	2.739056	up	1.159065	up
Os.8051.1.S1_at	2.0552456	up	-1.83991	down	2.444569	up	1.117038	up
Os.18186.1.S1_at	-2.020058	down	-1.24295	down	-1.06839	down	-2.51083	down
Os.5649.1.S1_at	1.0657772	up	-1.96007	down	2.602841	up	-1.8391	down
Os.26566.1.S1_at	2.129319	up	-1.3629	down	1.8096	up	1.562344	up
Os.17563.2.S1_a_at	-2.0330336	down	-1.14771	down	-1.15604	down	-2.33332	down
Os.18106.1.S1_at	-2.016274	down	1.124494	up	-1.49186	down	-1.79305	down
Os.49519.1.S1_at	3.7600248	up	1.157626	up	1.145964	up	4.352704	up
Os.54657.1.S1_at	-2.7131832	down	1.42315	up	-1.88791	down	-1.90646	down
Os.17887.1.S1_at	1.0110514	up	2.125977	up	-1.60281	down	2.149472	up
Os.3729.1.S1_at	1.6572497	up	1.600844	up	-1.20699	down	2.652998	up
Os.10497.1.S1_s_at	4.5161448	up	-1.97062	down	1.485828	up	2.291733	up
Os.9167.1.A1_at	-1.2793632	down	-2.2156	down	1.670769	up	-2.83456	down
Os.44676.1.S1_at	-2.0533054	down	-1.00494	down	-1.31943	down	-2.06345	down
Os.51114.1.S1_x_at	-1.2635719	down	2.876941	up	-3.81448	down	2.276832	up
Os.55363.1.S1_at	3.2203238	up	-1.34027	down	1.776476	up	2.402739	up
Os.27456.1.S1_x_at	-2.520899	down	1.311044	up	-1.73767	down	-1.92282	down
Os.53714.1.S1_at	-1.327168	down	1.559923	up	-2.06751	down	1.175378	up
Os.28089.1.S1_at	-1.8393947	down	1.86959	up	-2.47785	down	1.016416	up
Os.20403.1.S1_s_at	1.0213826	up	3.521176	up	-2.65686	down	3.596467	up
Os.57474.1.S1_x_at	1.1508869	up	1.313783	up	-1.74114	down	1.512016	up
Os.20531.1.S1_at	-1.4782073	down	1.654208	up	-2.19194	down	1.119064	up
Os.17173.1.S1_at	-1.3479198	down	2.112979	up	-1.59483	down	1.567585	up
Os.52424.1.S1_x_at	-2.2711232	down	1.088842	up	-1.44256	down	-2.08581	down
OsAffx.15508.1.S1_at	-2.099349	down	-1.00779	down	-1.3146	down	-2.1157	down
Os.6294.1.S1_s_at	-2.020927	down	1.32578	up	-1.75621	down	-1.52433	down
Os.5147.1.S1_at	1.304922	up	1.262161	up	-1.67165	down	1.647022	up
Os.12037.1.S1_at	-2.2931738	down	1.946491	up	-2.57793	down	-1.17811	down
OsAffx.18633.1.S1_at	-1.0451417	down	-1.62382	down	2.150507	up	-1.69712	down
Os.6774.1.S1_at	-2.0550666	down	1.623	up	-2.14924	down	-1.26622	down
Os.49533.1.S1_at	-1.7783325	down	-1.27904	down	-1.03488	down	-2.27455	down
Os.12645.1.S1_at	1.4127015	up	-2.17697	down	2.881125	up	-1.541	down
Os.49107.1.A1_at	-1.1694996	down	2.413631	up	-1.82375	down	2.063815	up
Os.9355.1.S1_at	2.1309426	up	1.441285	up	-1.08947	down	3.071296	up
Os.7081.1.S1_at	-2.141065	down	1.042674	up	-1.37936	down	-2.05344	down
Os.49495.3.S1_at	-1.6147826	down	-1.31141	down	-1.00866	down	-2.11764	down
Os.17486.1.S1_at	-1.8164892	down	2.46527	up	-1.86387	down	1.357162	up
OsAffx.13994.1.S1_at	-1.0063167	down	3.578975	up	-4.73312	down	3.55651	up
Os.26537.1.S1_a_at	-3.7927227	down	-1.18597	down	-1.11507	down	-4.49806	down
Os.34400.1.S1_at	-1.7119411	down	-1.59004	down	1.202591	up	-2.72205	down
Os.54365.1.S1_at	-1.8683512	down	-1.07336	down	-1.23176	down	-2.00542	down

Os.7972.1.S1_at	2.2352743	up	1.092251	up	1.210105	up	2.441482	up
Os.10491.1.S1_at	-2.7028623	down	1.34509	up	-1.01817	down	-2.00943	down
Os.17439.1.S1_at	-2.2581158	down	1.332736	up	-1.76041	down	-1.69435	down
Os.5842.1.S1_at	-2.0731535	down	2.036863	up	-2.69048	down	-1.01782	down
Os.313.1.S1_a_at	1.1284469	up	-1.60014	down	2.113511	up	-1.41801	down
Os.49581.1.S1_at	-2.0362644	down	1.505069	up	-1.98789	down	-1.35294	down
OsAffx.4662.1.S1_at	1.1083816	up	5.625983	up	-4.25991	down	6.235736	up
OsAffx.28306.1.S1_x_	1.7030069	up	-1.00503	down	-1.31383	down	1.694479	up
Os.17133.1.S1_at	-1.7006643	down	1.52496	up	-2.01344	down	-1.11522	down
Os.47937.2.S1_x_at	-1.4452215	down	-1.4213	down	1.076541	up	-2.0541	down
Os.46451.1.S2_at	-1.3467149	down	-1.955	down	1.480925	up	-2.63282	down
Os.7914.2.S1_at	-1.177843	down	1.528156	up	-2.01734	down	1.297419	up
OsAffx.3115.1.S1_x_a	-1.6248007	down	-1.27102	down	-1.03851	down	-2.06516	down
Os.37797.1.A1_at	-2.5900638	down	-1.04374	down	-1.26444	down	-2.70335	down
Os.18652.1.S1_at	-1.9850664	down	-1.02211	down	-1.29108	down	-2.02896	down
Os.23226.1.S1_at	-2.3823264	down	-1.064	down	-1.24001	down	-2.53479	down
Os.4974.1.S1_x_at	-2.349949	down	2.869224	up	-3.7855	down	1.220973	up
Os.55102.1.S1_at	-2.337682	down	-1.09865	down	-1.20048	down	-2.56828	down
Os.5405.1.S1_x_at	-1.97397	down	-1.04992	down	-1.25594	down	-2.0725	down
Os.18627.1.S1_at	-1.1259258	down	1.767612	up	-2.33023	down	1.569919	up
Os.25060.1.S1_at	-2.0233915	down	1.637289	up	-2.15832	down	-1.23582	down
Os.24972.1.S1_at	-1.5666419	down	1.744483	up	-2.29927	down	1.113518	up
OsAffx.18126.1.S1_at	1.156011	up	2.199461	up	-1.66883	down	2.542601	up
OsAffx.21494.1.S1_x_	-1.8099325	down	-1.18658	down	-1.11071	down	-2.14763	down
Os.34673.1.S1_at	-2.2328842	down	1.700896	up	-2.24141	down	-1.31277	down
Os.52666.1.S1_at	-2.8478794	down	1.582675	up	-2.08558	down	-1.79941	down
Os.47355.1.S1_at	-1.4784052	down	-1.35619	down	1.029182	up	-2.005	down
Os.8648.1.S1_s_at	1.0780836	up	2.714215	up	-2.05991	down	2.926151	up
Os.27084.1.A1_a_at	-2.2321455	down	1.754236	up	-2.31136	down	-1.27243	down
Os.22262.3.S1_a_at	-1.9384466	down	-1.05469	down	-1.24876	down	-2.04446	down
Os.11584.2.S1_x_at	1.161481	up	1.49794	up	-1.97281	down	1.739829	up
Os.38638.1.S1_at	1.8013474	up	-1.59233	down	2.096977	up	1.131264	up
Os.10183.1.S2_at	1.2945629	up	2.300322	up	-1.74692	down	2.977911	up
OsAffx.17170.1.S1_at	1.0735283	up	-2.23981	down	2.948806	up	-2.0864	down
Os.37971.1.A1_at	-2.433428	down	1.05914	up	-1.39367	down	-2.29755	down
Os.36597.1.S1_x_at	-2.6301532	down	1.068766	up	-1.40629	down	-2.46093	down
Os.27562.1.S1_at	-2.3396907	down	-1.35719	down	1.031689	up	-3.1754	down
Os.49023.1.S1_x_at	-1.2794948	down	-2.03994	down	1.55084	up	-2.61009	down
Os.10351.2.S1_at	2.5460186	up	1.017328	up	1.292035	up	2.590136	up
Os.55380.1.S1_at	3.083445	up	-5.73619	down	7.539629	up	-1.86032	down
Os.17911.1.S1_at	1.6077077	up	-1.8634	down	2.448861	up	-1.15904	down
Os.32650.1.S1_at	-1.5254724	down	-1.38289	down	1.052282	up	-2.10956	down
Os.24098.1.S1_a_at	-1.6695749	down	1.081594	up	1.215024	up	-1.54362	down
Os.49706.1.S1_at	-2.1136494	down	1.123052	up	-1.47546	down	-1.88206	down
Os.49783.1.S1_at	-1.9762669	down	3.438167	up	-2.61712	down	1.739728	up
Os.5355.1.S1_at	-2.3359888	down	1.253129	up	-1.64615	down	-1.86412	down
Os.29003.1.S1_at	-2.3396246	down	1.883296	up	-1.43387	down	-1.2423	down

Os.11396.1.S1_at	1.476356	up	1.541482	up	-1.17447	down	2.275776	up
Os.9527.1.S1_at	-1.9651883	down	2.184147	up	-1.66418	down	1.111419	up
Os.50496.1.S1_at	1.3527175	up	1.497227	up	-1.14079	down	2.025326	up
Os.6594.1.S1_at	-1.4783928	down	-1.41271	down	1.076435	up	-2.08854	down
OsAffx.29395.1.S1_at	-2.3472786	down	-1.30583	down	-1.00502	down	-3.06514	down
Os.18684.1.S1_at	-2.4255528	down	1.066364	up	-1.39943	down	-2.2746	down
OsAffx.22469.1.S1_x_	2.2498221	up	-2.85068	down	3.740674	up	-1.26707	down
Os.39045.1.S1_x_at	-1.460901	down	2.040875	up	-1.55563	down	1.396997	up
Os.14382.1.S1_at	-1.8575416	down	1.778567	up	-2.33268	down	-1.0444	down
Os.52734.1.S1_at	-2.0388415	down	-1.09817	down	-1.19428	down	-2.23899	down
Os.28686.1.S1_at	-2.1357508	down	1.519391	up	-1.99231	down	-1.40566	down
Os.52392.1.S1_at	-2.6080997	down	1.241677	up	-1.62799	down	-2.10047	down
Os.15045.1.S1_at	1.1936127	up	-1.81272	down	2.376663	up	-1.51869	down
Os.16743.3.S1_x_at	-1.1915247	down	-1.68454	down	2.208591	up	-2.00717	down
Os.49570.1.S1_at	1.0685132	up	2.031091	up	-1.54947	down	2.170247	up
Os.48109.1.A1_s_at	1.107914	up	1.423252	up	-1.86542	down	1.576841	up
Os.26996.1.S1_at	-2.0280683	down	1.211205	up	-1.58737	down	-1.67442	down
OsAffx.4171.1.S1_s_a	-3.132666	down	1.088258	up	-1.42618	down	-2.87861	down
OsAffx.12824.1.S1_at	-2.3910117	down	1.232074	up	-1.61449	down	-1.94064	down
Os.14191.1.S1_at	-2.2554939	down	1.752858	up	-2.29664	down	-1.28675	down
Os.22624.1.A1_at	1.067426	up	-2.09916	down	1.602184	up	-1.96656	down
Os.27003.1.S1_a_at	-2.372709	down	1.469753	up	-1.92564	down	-1.61436	down
Os.6981.1.S1_at	-1.4853003	down	-1.38737	down	1.058983	up	-2.06066	down
Os.9411.1.S1_at	-1.6964872	down	-1.8772	down	1.433123	up	-3.18465	down
Os.6193.1.S1_at	-2.186123	down	1.116541	up	-1.46248	down	-1.95794	down
Os.55703.1.S1_at	1.884647	up	2.003543	up	-1.52971	down	3.775971	up
Os.49255.1.S1_at	-1.9436865	down	2.164647	up	-1.65292	down	1.113681	up
Os.10753.1.S1_a_at	-2.1913896	down	1.369498	up	-1.79339	down	-1.60014	down
Os.52136.1.S1_at	-1.3772393	down	-1.12496	down	1.473102	up	-1.54934	down
OsAffx.25718.1.S1_at	-2.1243408	down	1.951981	up	-2.55559	down	-1.0883	down
Os.2612.1.S1_at	-1.5519025	down	1.999431	up	-2.61735	down	1.288374	up
Os.46037.1.S1_x_at	-1.5591105	down	-1.29673	down	-1.00945	down	-2.02175	down
Os.2690.1.S1_a_at	1.370774	up	1.268235	up	-1.66009	down	1.738464	up
Os.42200.1.S1_at	-2.0712183	down	1.357049	up	-1.77626	down	-1.52627	down
Os.7455.1.S1_at	-2.1402397	down	1.158195	up	-1.51588	down	-1.84791	down
Os.31379.1.S1_s_at	-1.2448468	down	-1.47189	down	1.925375	up	-1.83228	down
Os.12003.1.S1_at	-2.5065231	down	1.356396	up	-1.77419	down	-1.84793	down
Os.27301.1.S1_at	1.9470782	up	1.513469	up	-1.15794	down	2.946843	up
Os.49738.1.S1_at	-2.0085163	down	-1.02533	down	-1.2747	down	-2.05939	down
OsAffx.30783.1.S1_at	2.3966959	up	1.456067	up	-1.11415	down	3.48975	up
OsAffx.15530.1.S1_at	-1.4977618	down	-1.80181	down	1.37877	up	-2.69869	down
Os.46107.1.S2_x_at	-2.4283054	down	1.607608	up	-2.10068	down	-1.51051	down
OsAffx.4139.1.S1_at	1.5213006	up	1.317311	up	-1.0082	down	2.004025	up
Os.51942.1.S1_at	-2.824376	down	1.065102	up	-1.39136	down	-2.65174	down
Os.53062.1.S1_at	-1.4773777	down	1.643853	up	-2.14734	down	1.112683	up
Os.12633.1.S1_at	1.8973671	up	-7.58964	down	9.913964	up	-4.00009	down
Os.53284.1.S1_at	-1.2996763	down	-2.34602	down	1.796002	up	-3.04906	down

OsAffx.6359.1.S1_s_a	1.8489211	up	1.465971	up	-1.12237	down	2.710465	up
Os.55755.1.S1_at	-2.046294	down	-1.2717	down	-1.02698	down	-2.60227	down
Os.51849.1.S1_at	-3.0196354	down	1.065477	up	-1.39111	down	-2.83407	down
Os.53407.1.S1_at	2.30834	up	1.171287	up	1.114571	up	2.703729	up
Os.25112.1.S1_at	-1.7130114	down	-1.25842	down	-1.03731	down	-2.15569	down
Os.5200.1.S1_at	-2.028162	down	-1.28406	down	-1.01652	down	-2.60428	down
Os.15507.1.S1_at	-2.1383681	down	-1.00112	down	-1.30372	down	-2.14076	down
Os.50853.1.S1_at	-2.2564487	down	1.535204	up	-2.00367	down	-1.4698	down
Os.52949.1.S1_s_at	-2.185904	down	1.027088	up	-1.3405	down	-2.12826	down
Os.26772.2.S1_at	-2.735403	down	1.360677	up	-1.04275	down	-2.01032	down
Os.24708.1.S1_at	-2.5311003	down	1.039572	up	-1.35633	down	-2.43475	down
Os.17761.1.S1_a_at	-2.0925193	down	1.872662	up	-2.44322	down	-1.1174	down
OsAffx.24724.1.S1_x_	-1.783014	down	1.574869	up	-2.05454	down	-1.13217	down
Os.6764.1.S1_at	-1.9581306	down	-2.10292	down	1.612348	up	-4.11779	down
Os.28397.1.S3_at	1.6203744	up	1.648966	up	-1.26431	down	2.671942	up
Os.54333.1.S1_at	-2.851106	down	1.24115	up	-1.61874	down	-2.29715	down
OsAffx.12915.1.S1_at	1.8520613	up	1.295195	up	1.006885	up	2.398779	up
Os.47337.1.S1_at	-1.6314801	down	-1.23643	down	-1.05471	down	-2.01721	down
Os.11568.2.S1_at	-1.8458394	down	-1.26917	down	-1.02748	down	-2.34268	down
Os.48371.1.S1_at	-2.0250678	down	1.007501	up	-1.31373	down	-2.00999	down
Os.52788.1.S1_at	-2.4000711	down	1.027086	up	-1.33921	down	-2.33678	down
Os.52689.1.S2_at	-1.0340204	down	-3.94216	down	3.023542	up	-4.07628	down
Os.26791.1.S1_a_at	-2.2597187	down	1.418468	up	-1.8492	down	-1.59307	down
OsAffx.26387.1.S1_at	2.4945815	up	-1.5248	down	1.987756	up	1.636005	up
Os.34674.1.S1_at	-2.0464957	down	-1.47525	down	1.131858	up	-3.0191	down
Os.7463.1.S1_at	-2.199746	down	1.250571	up	-1.62998	down	-1.75899	down
Os.12373.1.S1_at	2.3861716	up	1.712998	up	-1.31436	down	4.087508	up
Os.14843.1.S1_at	-1.2275091	down	1.579962	up	-2.05904	down	1.287128	up
Os.25027.3.S1_at	1.7215389	up	-1.61071	down	2.098994	up	1.06881	up
Os.11835.4.S1_x_at	-2.2124474	down	1.155713	up	-1.50604	down	-1.91436	down
OsAffx.13519.1.S1_at	-1.0594456	down	1.756119	up	-2.28845	down	1.657583	up
Os.1445.1.S1_at	-1.0502379	down	-1.54683	down	2.0157	up	-1.62454	down
Os.16076.1.S1_s_at	-2.790428	down	1.191324	up	-1.55234	down	-2.34229	down
Os.54698.1.S1_at	-3.5526133	down	-5.92395	down	4.547498	up	-21.0455	down
Os.27800.1.A1_at	1.2333385	up	2.318095	up	-1.7796	down	2.858996	up
Os.15633.1.S1_at	2.5245667	up	1.156435	up	1.126358	up	2.919498	up
Os.22835.1.S1_at	-1.627469	down	-1.25467	down	-1.03807	down	-2.04193	down
Os.17682.1.S1_at	-1.2769715	down	-1.56716	down	1.203329	up	-2.00122	down
Os.2698.3.S1_x_at	-1.6215745	down	-1.63786	down	1.257699	up	-2.65591	down
Os.32856.1.S2_at	-4.8136497	down	2.122497	up	-2.76377	down	-2.26792	down
Os.54048.1.S1_at	-2.078724	down	1.213873	up	-1.5806	down	-1.71247	down
Os.53243.1.S1_x_at	-2.0077162	down	-1.31397	down	1.009114	up	-2.63808	down
Os.50865.1.S1_at	-2.123103	down	1.300995	up	-1.69324	down	-1.63191	down
Os.14092.1.S1_at	-2.4635062	down	1.509951	up	-1.96508	down	-1.63151	down
Os.23416.1.S1_x_at	1.9057041	up	1.59107	up	-1.22292	down	3.032109	up
Os.8382.1.S1_x_at	-1.2328268	down	2.258359	up	-1.73583	down	1.831854	up
Os.30886.2.S1_x_at	2.4899485	up	-1.64306	down	1.263866	up	1.515433	up

Os.19374.2.S1_x_at	1.4059507	up	1.460465	up	-1.12365	down	2.053342	up
Os.56162.1.A1_x_at	-1.7485402	down	-1.18799	down	-1.09404	down	-2.07726	down
OsAffx.17667.1.S1_at	-1.9287317	down	-1.31705	down	1.013553	up	-2.54024	down
Os.22584.1.S1_at	-1.4043461	down	2.279787	up	-1.75474	down	1.62338	up
Os.53856.1.S1_at	-3.2142212	down	1.423084	up	-1.84888	down	-2.25863	down
Os.12524.1.S1_at	-1.6831107	down	-1.19946	down	1.558319	up	-2.01882	down
Os.27569.4.S1_x_at	-2.3495817	down	1.087675	up	-1.41309	down	-2.16019	down
Os.10303.1.S2_at	-2.334695	down	1.471273	up	-1.91126	down	-1.58685	down
OsAffx.4464.1.S1_s_a	-3.6296463	down	1.119389	up	-1.4539	down	-3.24253	down
Os.53699.1.S1_at	-2.0373342	down	1.287848	up	-1.6724	down	-1.58197	down
Os.32462.1.S1_a_at	2.796748	up	1.027785	up	1.263168	up	2.874457	up
Os.8582.1.S1_at	1.0393022	up	4.983323	up	-3.83889	down	5.179179	up
Os.51061.1.S1_at	-2.1905887	down	-1.48075	down	1.140752	up	-3.24372	down
Os.49627.2.S1_x_at	1.073131	up	1.554106	up	-2.01708	down	1.667759	up
Os.50457.2.S1_at	-2.6012957	down	1.039576	up	-1.34907	down	-2.50227	down
Os.5268.1.S1_at	-2.3242521	down	1.166539	up	-1.5138	down	-1.99243	down
Os.10612.1.S1_at	2.3169668	up	-1.08732	down	1.410605	up	2.130895	up
Os.46585.1.S1_at	-1.570316	down	1.01578	up	1.277066	up	-1.54592	down
Os.7767.1.S1_at	1.2874272	up	-16.8434	down	21.84475	up	-13.083	down
Os.8012.1.S1_at	-2.362075	down	1.299127	up	-1.68487	down	-1.8182	down
Os.53164.1.S1_at	-1.0591954	down	2.17567	up	-1.67767	down	2.054078	up
Os.8446.1.S1_at	-1.4611914	down	2.026869	up	-1.56324	down	1.387135	up
Os.3740.4.S1_at	-2.2876089	down	1.032733	up	-1.33886	down	-2.2151	down
Os.35642.2.S1_x_at	-2.2985368	down	2.413608	up	-3.129	down	1.050063	up
Os.27918.1.A1_at	-1.1379828	down	5.439689	up	-7.04954	down	4.780116	up
Os.10024.1.S1_at	-1.1776437	down	2.143899	up	-1.65514	down	1.820499	up
Os.37867.1.S1_at	-1.7167197	down	-1.23864	down	-1.04538	down	-2.1264	down
OsAffx.17196.1.S1_at	-1.8878019	down	-1.12752	down	1.459456	up	-2.12853	down
Os.10841.1.S1_at	1.92881	up	1.699396	up	-1.31303	down	3.277812	up
Os.7078.1.S1_at	8.478166	up	1.080934	up	1.197224	up	9.164341	up
Os.8454.1.S1_at	-1.8441854	down	-1.29075	down	-1.00244	down	-2.38038	down
Os.17502.1.S1_at	-2.4987426	down	1.328241	up	-1.71801	down	-1.88124	down
Os.17370.2.S1_x_at	-2.009021	down	1.074805	up	-1.39008	down	-1.8692	down
Os.12686.1.S1_at	2.2965322	up	1.049061	up	1.232754	up	2.409202	up
Os.50635.1.S1_x_at	-1.1083783	down	2.540865	up	-1.96526	down	2.292417	up
Os.49566.1.S1_at	-2.4954643	down	-1.04876	down	-1.23274	down	-2.61715	down
Os.52616.1.A1_at	-1.6256974	down	2.549847	up	-3.2963	down	1.568463	up
Os.5860.1.S1_at	1.3555995	up	1.746937	up	-1.35179	down	2.368147	up
Os.6805.1.A1_at	1.4248343	up	1.407339	up	-1.08906	down	2.005225	up
Os.10797.1.S1_at	-3.1586335	down	1.141093	up	-1.47417	down	-2.76808	down
Os.53946.1.S1_at	-3.0688257	down	1.670015	up	-2.15719	down	-1.8376	down
OsAffx.11931.1.S1_at	-3.2938905	down	-1.12496	down	-1.14818	down	-3.70551	down
Os.5133.1.S1_at	1.3824418	up	1.461697	up	-1.13174	down	2.020711	up
OsAffx.13499.1.S1_at	-2.0339806	down	1.029333	up	-1.32919	down	-1.97602	down
Os.26772.1.S1_x_at	-2.1745543	down	1.119291	up	-1.44523	down	-1.9428	down
Os.36409.1.S1_x_at	1.3560867	up	-3.15009	down	4.066752	up	-2.32293	down
OsAffx.5759.1.S1_at	-2.0508628	down	1.085689	up	-1.40143	down	-1.889	down

Os.47882.1.A1_at	-1.7170411	down	-1.25354	down	-1.02964	down	-2.15239	down
Os.10908.4.S1_at	2.226065	up	1.028641	up	1.254707	up	2.289822	up
Os.49718.1.S1_at	-2.4305336	down	1.28684	up	-1.66041	down	-1.88876	down
Os.46443.1.S1_at	1.722306	up	1.687134	up	-1.30775	down	2.905761	up
Os.24395.1.S1_a_at	-2.0637538	down	-1.0115	down	-1.27514	down	-2.08748	down
OsAffx.16409.1.S1_s_	3.3366573	up	-1.32417	down	1.707802	up	2.519815	up
Os.9868.1.S1_at	-1.4735119	down	-2.02749	down	1.572175	up	-2.98753	down
Os.5707.1.S1_at	-1.8590875	down	-1.07933	down	-1.19483	down	-2.00656	down
Os.7419.1.S1_a_at	-2.5089176	down	1.062234	up	-1.36973	down	-2.36193	down
Os.27551.1.S1_at	1.5787957	up	1.272736	up	1.013151	up	2.00939	up
Os.38164.1.S2_at	1.3933073	up	1.640371	up	-1.27221	down	2.285542	up
Os.4660.1.S1_s_at	-1.2794935	down	2.040004	up	-1.58253	down	1.594384	up
OsAffx.21798.1.S1_x_	1.6983575	up	-2.31211	down	1.794082	up	-1.36138	down
Os.52569.1.S1_at	-1.8010731	down	1.596185	up	-2.05685	down	-1.12836	down
Os.11422.1.S1_at	-2.1343365	down	1.257587	up	-1.61914	down	-1.69717	down
Os.17721.1.S1_at	-1.5469875	down	-1.37225	down	1.065888	up	-2.12285	down
Os.49405.2.S1_at	-2.1808963	down	1.02432	up	-1.31865	down	-2.12912	down
Os.5246.1.S1_at	-1.2954708	down	-1.3967	down	1.797923	up	-1.80938	down
Os.19038.1.S1_at	2.137068	up	-1.02264	down	-1.25862	down	2.089757	up
Os.32042.1.S1_at	-2.1270413	down	1.06246	up	-1.36729	down	-2.002	down
Os.8545.1.S1_at	1.5383184	up	1.512358	up	-1.1752	down	2.326487	up
Os.6242.1.S1_s_at	1.6037965	up	2.124647	up	-1.65209	down	3.407502	up
Os.52709.1.S1_at	1.4827386	up	-3.12594	down	2.432486	up	-2.10822	down
Os.53623.1.S1_at	-1.7679877	down	-1.2613	down	-1.01874	down	-2.22997	down
Os.11488.1.S1_at	-1.8082114	down	-1.11768	down	1.435795	up	-2.021	down
Os.53661.1.S1_s_at	-1.7049237	down	-1.34572	down	1.047593	up	-2.29434	down
Os.439.1.S1_at	-2.0729344	down	1.104334	up	-1.41858	down	-1.87709	down
Os.8653.1.S1_at	-1.6657926	down	-1.21666	down	-1.05577	down	-2.0267	down
Os.52825.1.S1_at	2.0749257	up	-1.27108	down	1.632554	up	1.63241	up
Os.48088.1.S1_at	-1.5848007	down	-1.41432	down	1.101177	up	-2.24142	down
Os.47490.1.S1_at	-2.0668051	down	1.441828	up	-1.12267	down	-1.43346	down
Os.52186.1.S1_at	-2.7346528	down	1.079655	up	-1.38656	down	-2.5329	down
Os.50897.1.S1_at	-3.0550418	down	-1.04982	down	-1.22322	down	-3.20724	down
Os.5601.1.S1_at	-2.8631644	down	2.62481	up	-3.37055	down	-1.09081	down
Os.15998.1.S1_at	-2.068284	down	1.060401	up	-1.36154	down	-1.95047	down
Os.51299.1.S1_at	1.2352272	up	2.129127	up	-1.65836	down	2.629956	up
OsAffx.4594.1.S1_at	2.5883164	up	-1.41169	down	1.812321	up	1.833487	up
Os.35827.1.S1_at	-2.259021	down	1.205808	up	-1.54672	down	-1.87345	down
Os.7914.1.S1_at	-1.3739147	down	1.613634	up	-2.06966	down	1.174479	up
Os.18235.1.S1_at	1.0270487	up	2.133775	up	-1.66369	down	2.19149	up
Os.53730.1.S2_at	-2.025464	down	-1.00479	down	-1.27597	down	-2.03517	down
Os.8775.1.S1_at	-1.9224246	down	1.025107	up	1.250487	up	-1.87534	down
Os.11018.1.S1_at	2.5090294	up	-1.2735	down	1.632261	up	1.97019	up
Os.18467.1.S1_s_at	-1.8303883	down	2.318887	up	-1.80954	down	1.266883	up
Os.17174.1.S1_at	-2.058076	down	3.610732	up	-2.8177	down	1.754422	up
Os.11786.1.S1_at	6.264501	up	-2.49737	down	3.200225	up	2.508445	up
Os.23097.1.A1_at	-2.2784507	down	-1.16925	down	-1.09593	down	-2.66409	down

Os.38245.1.S1_at	2.0979092	up	2.918936	up	-2.27803	down	6.123662	up
Os.33145.1.S1_at	2.7684197	up	1.118687	up	1.145333	up	3.096995	up
Os.18502.1.S1_at	-2.2698157	down	1.033673	up	-1.32438	down	-2.19587	down
Os.7287.2.S1_x_at	-2.1424603	down	1.121975	up	-1.43682	down	-1.90954	down
Os.8265.1.S1_at	-1.597901	down	2.112887	up	-1.64991	down	1.322289	up
Os.52470.1.S1_at	-2.4303715	down	1.104128	up	-1.41389	down	-2.20117	down
Os.11132.1.S1_s_at	-1.206298	down	-2.13011	down	1.663804	up	-2.56955	down
Os.31992.1.S1_at	1.8662629	up	1.232207	up	-1.57739	down	2.299622	up
Os.54469.1.S1_at	-2.0089767	down	-2.07384	down	1.620425	up	-4.1663	down
Os.14686.1.S1_at	-3.9837077	down	1.535722	up	-1.96538	down	-2.59403	down
Os.26691.1.A1_at	-2.7936754	down	1.328668	up	-1.70032	down	-2.10261	down
Os.54232.1.S1_at	2.2782764	up	2.498248	up	-1.95266	down	5.6917	up
Os.46674.1.S1_at	1.366437	up	1.919086	up	-1.50008	down	2.62231	up
Os.10251.1.S1_at	-1.3993452	down	-2.31122	down	1.806919	up	-3.23419	down
Os.7839.1.S1_at	-1.8335036	down	-1.15539	down	-1.10675	down	-2.11842	down
Os.38110.1.S1_at	3.9251819	up	-2.80631	down	2.194691	up	1.398698	up
Os.22935.1.S1_at	2.6888692	up	1.323201	up	-1.03489	down	3.557915	up
Os.46308.1.S2_s_at	-2.0226338	down	1.194965	up	-1.52742	down	-1.69263	down
Os.50271.1.S1_at	-2.0056014	down	1.243694	up	-1.58935	down	-1.61262	down
Os.30916.1.S1_at	-1.5015328	down	1.670595	up	-2.13448	down	1.112593	up
OsAffx.13558.1.S1_x_	-2.1215346	down	1.391906	up	-1.77824	down	-1.52419	down
Os.7666.1.S1_at	-2.2538567	down	1.154376	up	1.106476	up	-1.95245	down
Os.7864.1.S1_at	1.1160578	up	1.627	up	-2.07714	down	1.815826	up
Os.10555.1.S1_a_at	1.8194	up	1.612833	up	-1.26466	down	2.934388	up
Os.52102.1.A1_at	-2.097134	down	-1.039	down	-1.22666	down	-2.17892	down
OsAffx.19825.1.S1_x_	2.211102	up	1.258513	up	1.012333	up	2.7827	up
Os.11783.1.S1_at	-1.8158919	down	-1.16685	down	-1.09182	down	-2.11887	down
OsAffx.3286.1.S1_x_a	1.6093245	up	1.31284	up	-1.03058	down	2.112786	up
Os.11802.1.S1_at	1.6965737	up	1.192953	up	1.067572	up	2.023932	up
Os.51176.1.S1_at	1.192316	up	-2.11233	down	2.689848	up	-1.77162	down
Os.28300.4.S1_x_at	-1.8629383	down	-1.31403	down	1.032042	up	-2.44797	down
AFFX-Os_Actin_5_f_a	2.4516747	up	-1.18741	down	1.511786	up	2.064724	up
OsAffx.27916.1.S1_at	-2.4045894	down	1.137714	up	-1.44832	down	-2.11353	down
Os.14115.1.S1_at	1.277346	up	2.46929	up	-1.93981	down	3.154137	up
Os.54886.1.S1_at	-2.4748533	down	1.247495	up	-1.58785	down	-1.98386	down
Os.47763.1.A1_at	-2.0809708	down	1.294113	up	-1.64681	down	-1.60803	down
OsAffx.30148.1.S1_at	2.2788045	up	-1.44688	down	1.137024	up	1.574983	up
Os.11221.1.S1_at	-2.1573002	down	1.246975	up	-1.5867	down	-1.73003	down
Os.24080.1.A1_at	-1.9326973	down	-1.18869	down	-1.07041	down	-2.29737	down
Os.46450.1.S1_at	1.1621871	up	-2.62417	down	3.338566	up	-2.25796	down
Os.15359.1.S1_at	-1.7872146	down	-1.26057	down	-1.00904	down	-2.25291	down
Os.7513.1.S1_at	-2.09318	down	1.673265	up	-2.12764	down	-1.25096	down
Os.3494.1.S1_at	-1.5076417	down	-1.67834	down	1.319942	up	-2.53033	down
Os.37457.1.S1_at	2.014291	up	1.679089	up	-1.32068	down	3.382173	up
Os.18003.1.S1_at	2.0321484	up	1.276802	up	-1.00434	down	2.59465	up
Os.34868.1.S1_at	-2.5859852	down	1.066681	up	-1.35593	down	-2.42433	down
Os.21442.1.S1_x_at	-3.229858	down	1.941548	up	-2.46777	down	-1.66355	down

Os.23200.1.S1_x_at	-2.2050872	down	1.664993	up	-2.11622	down	-1.32438	down
Os.54529.1.S1_at	-1.8693962	down	1.175242	up	1.081404	up	-1.59065	down
Os.8708.1.S1_at	-1.3074201	down	1.695658	up	-2.15488	down	1.296949	up
Os.8117.2.S1_at	-2.1667078	down	1.609595	up	-2.0455	down	-1.34612	down
Os.35288.1.S1_at	-1.3668205	down	-1.60763	down	1.26505	up	-2.19734	down
OsAffx.3925.1.S1_at	-1.6212807	down	-1.23819	down	-1.02629	down	-2.00746	down
Os.8642.1.S1_a_at	-2.0330133	down	1.322214	up	-1.68005	down	-1.53758	down
Os.6501.1.A1_at	1.1808676	up	1.717988	up	-1.35214	down	2.028716	up
Os.8259.1.S1_at	1.4957724	up	2.085241	up	-1.64172	down	3.119045	up
OsAffx.7303.1.S1_at	2.0603027	up	1.101172	up	1.153247	up	2.268747	up
Os.49600.1.S1_at	-2.6711357	down	1.192547	up	-1.51434	down	-2.23986	down
Os.52750.1.S1_x_at	2.175024	up	-1.10099	down	1.398034	up	1.975511	up
Os.27837.2.S1_x_at	-1.2746342	down	-1.29084	down	1.638986	up	-1.64535	down
OsAffx.11002.1.S1_at	-1.7646818	down	-1.14804	down	-1.1057	down	-2.02593	down
OsAffx.5112.1.S1_s_a	-1.416088	down	-1.42622	down	1.123556	up	-2.01965	down
Os.51697.1.S1_at	-1.4089493	down	2.054292	up	-1.61857	down	1.458031	up
Os.7314.1.S1_at	-1.373604	down	-2.14386	down	1.689238	up	-2.94482	down
Os.19472.1.S1_at	1.5823454	up	1.711669	up	-1.34874	down	2.708452	up
OsAffx.28339.1.S1_at	-2.1623824	down	1.096321	up	-1.39124	down	-1.9724	down
Os.26657.1.A1_at	-1.5717392	down	-1.10495	down	1.401925	up	-1.73669	down
Os.27983.1.S2_at	-2.0218375	down	1.129988	up	-1.43354	down	-1.78926	down
Os.50017.1.S1_at	-2.0212677	down	1.53029	up	-1.20631	down	-1.32084	down
Os.53208.1.S1_at	-1.8423359	down	2.326754	up	-1.83431	down	1.262937	up
Os.10669.1.S1_at	1.6584532	up	1.587112	up	-1.2515	down	2.632152	up
Os.38669.1.A1_s_at	-1.0348098	down	-2.25621	down	2.860772	up	-2.33475	down
Os.14901.1.A1_a_at	-1.3299489	down	-2.40971	down	1.90085	up	-3.2048	down
Os.34697.1.S1_at	-1.9447848	down	-1.31571	down	1.037908	up	-2.55878	down
Os.14555.1.S1_at	-1.7908485	down	-1.254	down	1.589515	up	-2.24572	down
Os.17509.1.S1_at	1.6591499	up	1.561926	up	-1.23238	down	2.59147	up
Os.53079.1.S1_at	-2.072179	down	-1.05763	down	-1.19824	down	-2.1916	down
Os.27271.1.S1_a_at	-1.1104827	down	1.664847	up	-2.10982	down	1.49921	up
Os.55653.1.S1_at	1.6712549	up	1.332097	up	-1.68799	down	2.226273	up
Os.11682.1.S1_at	-2.0505652	down	-1.00619	down	-1.25908	down	-2.06326	down
Os.7298.2.S1_at	-2.8339934	down	1.07208	up	-1.35798	down	-2.64345	down
Os.26460.2.S1_x_at	1.2763906	up	1.608367	up	-1.26988	down	2.052905	up
Os.3141.2.S1_at	-1.9724959	down	-1.20675	down	-1.04928	down	-2.3803	down
Os.55681.1.S1_x_at	1.7457585	up	1.240634	up	1.0205	up	2.165848	up
Os.5354.1.S1_at	-1.1541797	down	3.165054	up	-2.50007	down	2.742254	up
Os.12223.1.S1_at	1.7702581	up	1.341404	up	-1.05967	down	2.374632	up
Os.17425.1.S1_at	-2.1259267	down	1.252543	up	-1.58547	down	-1.69729	down
Os.14287.1.S1_at	-2.488621	down	1.114294	up	-1.41	down	-2.23336	down
Os.4164.2.S1_a_at	2.838154	up	2.576063	up	-3.25935	down	7.311265	up
OsAffx.15314.1.S1_at	-2.477171	down	1.126958	up	-1.42558	down	-2.1981	down
Os.26728.1.S1_a_at	1.2662064	up	1.890152	up	-1.49456	down	2.393323	up
Os.2960.1.S1_at	-2.1691911	down	1.015662	up	-1.28421	down	-2.13574	down
Os.57534.1.S1_x_at	2.243001	up	-1.13976	down	1.441051	up	1.967962	up
Os.10381.1.S1_at	1.5127975	up	-1.95809	down	2.47536	up	-1.29435	down

Os.51190.1.S1_at	1.5755013	up	1.57473	up	-1.24591	down	2.480989	up
Os.38812.1.S1_at	2.4482732	up	-8.11313	down	6.420624	up	-3.31382	down
Os.3380.1.S1_at	-1.4116071	down	1.608495	up	-2.03219	down	1.139478	up
Os.18993.2.S1_at	-1.5148963	down	-1.62218	down	1.2842	up	-2.45743	down
Os.8487.1.S1_at	1.1015478	up	-2.70443	down	2.141075	up	-2.45512	down
Os.6389.1.S1_at	-1.0245419	down	-5.91902	down	4.686765	up	-6.06429	down
Os.27769.1.S1_a_at	-1.2626082	down	-1.61413	down	1.278849	up	-2.03801	down
Os.10560.1.S1_at	-1.394942	down	-1.57707	down	1.249591	up	-2.19993	down
Os.23948.1.A1_at	-1.9067961	down	-1.14714	down	-1.10002	down	-2.18736	down
Os.21636.2.S1_a_at	1.2078518	up	3.214522	up	-2.5478	down	3.882666	up
Os.53054.1.S1_at	1.1759595	up	1.456006	up	-1.83675	down	1.712205	up
Os.27926.1.A1_s_at	1.084599	up	-2.27089	down	1.800372	up	-2.09376	down
Os.49095.1.S1_at	-2.6804326	down	1.611054	up	-2.03133	down	-1.66378	down
Os.7067.1.S1_at	-1.330449	down	1.615222	up	-2.03614	down	1.214043	up
Os.53969.1.S1_at	-2.6530147	down	-1.28273	down	1.017658	up	-3.4031	down
OsAffx.25131.1.S1_at	1.3686754	up	-3.28586	down	4.141601	up	-2.40076	down
Os.12052.1.S1_at	-2.0300004	down	-1.37269	down	1.730079	up	-2.78657	down
Os.28112.1.S1_at	-1.9005226	down	-1.23302	down	-1.02191	down	-2.34339	down
Os.27968.1.S1_x_at	-2.0106785	down	1.256958	up	-1.58335	down	-1.59964	down
Os.49168.1.S1_s_at	-2.265466	down	-1.09114	down	-1.15402	down	-2.47194	down
Os.52202.1.S1_at	-1.669693	down	-1.21458	down	-1.03634	down	-2.02798	down
Os.50417.1.S1_at	-1.1119933	down	1.867426	up	-2.35033	down	1.67935	up
Os.57319.1.S1_at	-3.0793698	down	1.514256	up	-1.90536	down	-2.03359	down
Os.14189.1.S1_s_at	2.6323247	up	-1.7019	down	2.141367	up	1.546701	up
Os.10628.1.S1_at	-1.8727664	down	-1.1113	down	-1.13202	down	-2.0812	down
OsAffx.2983.1.S1_x_a	3.944061	up	1.089543	up	1.154415	up	4.297225	up
Os.4862.1.S1_at	1.7733848	up	-2.25701	down	1.794475	up	-1.27272	down
Os.57041.1.S1_at	1.8142093	up	1.321758	up	-1.05114	down	2.397945	up
Os.11341.1.S1_a_at	-1.414692	down	2.113705	up	-2.6576	down	1.49411	up
OsAffx.16741.1.S1_s_	-2.7438962	down	-1.05519	down	-1.19103	down	-2.89532	down
OsAffx.24612.1.S1_at	-2.8200676	down	2.875538	up	-2.28844	down	1.01967	up
Os.53317.1.S1_x_at	-2.6635597	down	1.091475	up	-1.37136	down	-2.44033	down
Os.35474.1.S1_at	-2.729382	down	1.286764	up	-1.61631	down	-2.12112	down
Os.7435.1.S1_at	-2.1449025	down	1.125863	up	-1.41419	down	-1.90512	down
OsAffx.11826.1.S1_at	-2.1340766	down	1.25568	up	-1.57702	down	-1.69954	down
Os.14232.1.S1_at	-1.0489844	down	1.780811	up	-2.23612	down	1.697653	up
Os.15490.1.S1_at	-1.3688021	down	-1.82744	down	1.45541	up	-2.50141	down
Os.9108.2.A1_a_at	-3.3162835	down	1.384911	up	-1.73888	down	-2.39458	down
Os.7710.1.S1_a_at	-1.4960699	down	-1.67561	down	1.334896	up	-2.50683	down
Os.18147.1.S1_at	-2.2351649	down	1.20861	up	-1.51673	down	-1.84937	down
Os.14949.1.S1_at	-3.6675704	down	-1.14087	down	-1.09976	down	-4.18422	down
Os.52854.1.S1_at	-1.8815917	down	1.80414	up	-2.26358	down	-1.04293	down
Os.14998.1.S1_at	1.2020774	up	1.329639	up	-1.66814	down	1.598329	up
Os.17844.1.S2_at	-1.9175345	down	-1.23209	down	-1.01778	down	-2.36258	down
Os.26990.1.S1_at	-1.907945	down	2.164253	up	-2.71368	down	1.134337	up
Os.11117.1.S2_at	-2.1125097	down	1.295358	up	-1.62414	down	-1.63083	down
Os.54545.1.S1_at	2.7750778	up	1.121877	up	-1.4065	down	3.113297	up

Os.12323.2.S1_at	1.3522538	up	1.517408	up	-1.21038	down	2.051921	up
Os.2507.1.S1_at	-1.9300779	down	-1.05487	down	-1.18795	down	-2.03599	down
OsAffx.4266.1.S1_at	1.654935	up	1.242232	up	1.008492	up	2.055813	up
Os.56345.1.S1_at	-1.0972315	down	-1.47151	down	1.843165	up	-1.61459	down
Os.12633.1.S1_s_at	1.8216103	up	-4.91511	down	6.155639	up	-2.69822	down
Os.38834.1.S1_at	-1.0353957	down	-1.65691	down	2.074187	up	-1.71556	down
Os.8967.1.S1_a_at	-1.304521	down	-1.48342	down	1.856869	up	-1.93515	down
Os.55287.1.S1_at	-2.2647	down	-1.10236	down	-1.1355	down	-2.49651	down
Os.12656.1.S1_s_at	1.3977126	up	-1.6166	down	2.023529	up	-1.15661	down
Os.17308.1.S1_at	-2.4918952	down	1.143366	up	-1.43085	down	-2.17944	down
Os.11040.1.S1_x_at	1.136925	up	-1.74687	down	2.185715	up	-1.53649	down
Os.11838.1.S1_at	1.5399368	up	-1.77602	down	2.221841	up	-1.15331	down
Os.32596.1.S1_x_at	2.241515	up	-1.10588	down	1.383457	up	2.026907	up
Os.53188.1.S1_at	1.4329007	up	2.044681	up	-2.55753	down	2.929824	up
Os.23290.2.S1_x_at	1.4000258	up	1.232069	up	-1.54052	down	1.724928	up
Os.18308.1.S1_at	-2.1146631	down	1.312316	up	-1.64041	down	-1.6114	down
OsAffx.31289.1.S1_at	-1.5276015	down	-1.33912	down	1.071297	up	-2.04564	down
Os.11863.1.S1_x_at	-2.1038265	down	1.378899	up	-1.72336	down	-1.52573	down
Os.52635.1.S1_at	-2.62151	down	1.670784	up	-2.08809	down	-1.56903	down
Os.24958.1.A1_at	-2.0102684	down	1.063892	up	-1.32945	down	-1.88954	down
Os.35123.1.S1_at	1.299298	up	5.424032	up	-4.34117	down	7.047434	up
Os.30886.1.S1_x_at	2.5452018	up	-1.83576	down	1.469333	up	1.386455	up
Os.5420.1.S1_at	1.6736032	up	1.206865	up	1.035137	up	2.019813	up
Os.17677.1.S1_at	-1.3515706	down	2.861226	up	-2.29124	down	2.116964	up
Os.39989.1.S1_s_at	-1.9997808	down	-1.0627	down	-1.17497	down	-2.12516	down
Os.37890.1.S1_s_at	1.0869795	up	-2.77927	down	2.226149	up	-2.55687	down
Os.37148.1.S1_at	1.0099316	up	1.609364	up	-2.00905	down	1.625348	up
Os.5230.1.S1_at	2.2313952	up	1.082409	up	1.152688	up	2.415281	up
Os.22919.1.S1_at	-2.1760674	down	1.102489	up	-1.37539	down	-1.97378	down
Os.20341.1.S1_s_at	1.4869946	up	1.508382	up	-1.20928	down	2.242955	up
Os.9778.1.S2_at	-1.3752515	down	2.45341	up	-1.96732	down	1.783972	up
Os.46552.1.S1_at	-2.1534946	down	1.171148	up	-1.46004	down	-1.83879	down
OsAffx.16365.1.S1_at	2.105418	up	-2.34228	down	2.919858	up	-1.1125	down
Os.16880.1.S1_at	-2.0803916	down	1.506318	up	-1.8768	down	-1.38111	down
Os.18030.1.S1_at	-1.9463055	down	1.198661	up	1.039313	up	-1.62373	down
Os.54202.1.S1_at	-1.5826832	down	-1.47089	down	1.181122	up	-2.32795	down
OsAffx.18799.1.S1_at	1.1228111	up	1.628849	up	-2.02844	down	1.82889	up
Os.1000.1.S1_at	1.9983299	up	2.754574	up	-2.21259	down	5.504547	up
Os.11567.1.S2_a_at	-2.0075815	down	1.357881	up	-1.69027	down	-1.47847	down
OsAffx.26488.1.S1_at	1.4292827	up	1.486671	up	-1.19474	down	2.124873	up
Os.35837.1.A1_at	-2.4594746	down	1.279414	up	-1.592	down	-1.92234	down
Os.23431.1.A1_at	-1.6579862	down	-1.25593	down	1.009506	up	-2.08232	down
Os.54566.1.S1_at	1.4262202	up	-1.68442	down	2.094976	up	-1.18104	down
Os.49835.2.S1_at	-1.5987176	down	-1.11463	down	1.386248	up	-1.78198	down
Os.1479.1.S1_at	-1.4170879	down	4.00723	up	-4.98371	down	2.827792	up
Os.6381.1.S1_at	1.6867641	up	1.33291	up	-1.07197	down	2.248305	up
Os.5680.1.S1_at	-1.8456945	down	-1.15878	down	-1.07291	down	-2.13876	down

OsAffx.15007.1.S1_at	1.2850032	up	1.906945	up	-1.53395	down	2.45043	up
Os.39543.1.A1_at	-1.802586	down	-1.17303	down	-1.05971	down	-2.11448	down
Os.49496.1.S1_at	-1.3658104	down	1.614332	up	-2.00666	down	1.181959	up
Os.27703.1.S1_s_at	2.3225718	up	17.29264	up	-13.9145	down	40.1634	up
Os.27431.1.A1_at	-2.7707024	down	2.87046	up	-3.56691	down	1.036005	up
OsAffx.16682.1.S1_at	2.7785769	up	-1.23911	down	1.539464	up	2.242395	up
Os.6170.1.S1_at	1.1371901	up	2.276129	up	-1.83211	down	2.588391	up
OsAffx.15533.1.S1_at	-1.7458382	down	1.616381	up	-2.00727	down	-1.08009	down
Os.7163.1.S1_at	-1.1932609	down	2.06065	up	-1.6601	down	1.726906	up
Os.12990.1.S1_x_at	3.8460815	up	2.288918	up	-1.84413	down	8.803364	up
Os.4788.1.S1_at	-2.2401786	down	-1.15201	down	-1.0774	down	-2.58071	down
Os.55098.1.S1_at	-2.3057272	down	1.277309	up	-1.58535	down	-1.80514	down
Os.51392.1.S1_at	1.6598916	up	-2.10525	down	1.696452	up	-1.2683	down
Os.21455.1.A1_at	-2.026179	down	1.321376	up	-1.63959	down	-1.53339	down
Os.5388.1.S1_at	1.2560288	up	-1.84655	down	2.291175	up	-1.47015	down
Os.13482.1.S1_x_at	2.273922	up	1.497802	up	-1.20717	down	3.405885	up
Os.55790.1.S1_at	2.1765687	up	1.097737	up	1.130063	up	2.3893	up
Os.7468.1.S1_at	-2.1082683	down	-1.31732	down	1.061945	up	-2.77727	down
Os.51759.1.S1_at	-1.9531397	down	-1.10879	down	-1.11837	down	-2.16561	down
Os.7002.1.S1_at	-1.0399704	down	2.389955	up	-2.96278	down	2.298099	up
Os.4901.1.S1_at	-2.500571	down	1.051631	up	-1.30342	down	-2.3778	down
Os.13010.1.S1_a_at	-1.4456358	down	-1.58424	down	1.278422	up	-2.29024	down
Os.6038.1.S1_at	-1.8281062	down	-2.51335	down	2.028417	up	-4.59468	down
Os.623.2.S1_x_at	2.7827787	up	-1.90882	down	2.3649	up	1.457857	up
OsAffx.30176.1.S1_at	-1.2427068	down	2.6661	up	-3.30295	down	2.145397	up
Os.19410.3.S1_x_at	-1.9739437	down	-1.22402	down	-1.01206	down	-2.41614	down
Os.1227.2.S1_x_at	-2.1300223	down	1.09543	up	-1.35699	down	-1.94446	down
Os.10994.1.S1_at	-1.8542249	down	-1.21418	down	-1.02014	down	-2.25137	down
Os.51250.1.S1_at	1.1149518	up	3.078311	up	-2.48544	down	3.432169	up
Os.418.1.S1_at	-3.240587	down	1.740739	up	-2.15487	down	-1.86162	down
Os.13854.1.S1_at	-1.2965138	down	-1.75323	down	1.416327	up	-2.27308	down
Os.46525.1.S1_at	-2.3116922	down	1.077	up	-1.33295	down	-2.14642	down
Os.18463.1.S1_at	1.447988	up	1.204641	up	-1.49047	down	1.744306	up
Os.27243.1.S1_at	2.2535276	up	1.004178	up	1.232006	up	2.262942	up
OsAffx.25117.1.S1_at	-2.3429916	down	1.582143	up	-1.95703	down	-1.4809	down
Os.50721.1.A1_at	1.3455046	up	-2.9094	down	2.352277	up	-2.16231	down
Os.32933.1.S1_at	-1.5437055	down	-1.39265	down	1.125998	up	-2.14983	down
Os.17676.3.S1_x_at	-2.3029118	down	1.019731	up	-1.26115	down	-2.25835	down
Os.27387.2.S1_x_at	2.2292137	up	-1.39332	down	1.723151	up	1.599926	up
Os.52298.1.S1_at	-2.4886105	down	3.949824	up	-4.88293	down	1.587161	up
Os.10963.1.S1_at	-2.4838443	down	-1.02754	down	-1.20289	down	-2.55224	down
Os.3740.1.S1_x_at	-1.6144779	down	-1.28914	down	1.042989	up	-2.08128	down
Os.5401.1.S1_at	1.2138373	up	1.8014	up	-2.22533	down	2.186606	up
Os.55386.1.A1_at	-1.2858554	down	3.768001	up	-3.05033	down	2.930346	up
Os.26798.2.S1_a_at	-1.5381691	down	1.888131	up	-2.33192	down	1.227518	up
Os.50424.1.S2_at	-2.0454214	down	-1.02127	down	-1.20872	down	-2.08892	down
Os.52632.1.S1_at	-2.125038	down	-1.18522	down	-1.04134	down	-2.51864	down

OsAffx.14201.1.S1_at	-1.1510278	down	5.22716	up	-4.23576	down	4.541298	up
Os.16037.1.S1_at	-1.7014749	down	2.292541	up	-2.8284	down	1.347384	up
Os.16286.1.S1_at	1.4805644	up	1.827003	up	-1.48093	down	2.704995	up
Os.6246.1.S1_at	-1.8772157	down	-1.11228	down	-1.10908	down	-2.08799	down
Os.10305.1.S1_at	1.6379118	up	2.809986	up	-2.27814	down	4.60251	up
Os.10620.1.S1_at	2.6462169	up	-1.724	down	2.126424	up	1.534927	up
Os.3927.3.S1_x_at	2.0450096	up	1.164527	up	1.059063	up	2.381469	up
OsAffx.14314.1.S1_x_	-3.0003374	down	1.340394	up	-1.65307	down	-2.2384	down
Os.34089.1.S1_at	-2.028364	down	1.149293	up	-1.41713	down	-1.76488	down
Os.50782.1.S1_a_at	-2.1017556	down	1.134091	up	-1.39838	down	-1.85325	down
Os.23434.1.S1_at	-2.3316417	down	1.364625	up	-1.68243	down	-1.70863	down
Os.49601.1.S1_at	2.2380784	up	-1.02417	down	1.262451	up	2.185258	up
Os.50887.1.A1_x_at	-2.1332824	down	1.085828	up	-1.33816	down	-1.96466	down
Os.17160.1.S1_s_at	-1.808166	down	-1.12221	down	-1.09796	down	-2.02913	down
Os.53241.1.S1_at	-2.6225982	down	-1.1133	down	-1.10619	down	-2.91974	down
Os.31867.1.S1_at	-1.7475066	down	-1.29957	down	1.599812	up	-2.27101	down
Os.8061.1.S1_at	1.4953994	up	-1.65816	down	2.040361	up	-1.10884	down
Os.53975.1.S1_at	-1.6238939	down	1.800075	up	-2.21494	down	1.108493	up
Os.55600.1.S1_at	2.2402701	up	1.053778	up	1.167356	up	2.360746	up
Os.50778.1.S1_at	-1.8069052	down	-1.15859	down	-1.06159	down	-2.09345	down
Os.27778.2.S1_at	-4.093771	down	1.308458	up	-1.60901	down	-3.1287	down
Os.57176.1.S1_at	-1.822561	down	-1.32743	down	1.079713	up	-2.41932	down
Os.551.1.S1_at	-1.4644608	down	-1.49067	down	1.832479	up	-2.18302	down
Os.10452.1.S1_at	-1.8754532	down	-1.06759	down	-1.15047	down	-2.00221	down
Os.49697.2.S1_at	-1.3187996	down	-1.34817	down	1.655209	up	-1.77797	down
Os.34481.1.A1_at	1.2479252	up	1.990288	up	-1.6219	down	2.483731	up
Os.53449.1.A1_at	-2.151251	down	1.042452	up	-1.27919	down	-2.06364	down
Os.21278.1.S1_at	-1.8269272	down	2.386896	up	-2.92865	down	1.306509	up
Os.17167.1.S1_at	-1.7523912	down	2.495098	up	-2.03369	down	1.423825	up
Os.9773.1.S1_at	1.1609261	up	1.498096	up	-1.8379	down	1.739178	up
Os.19112.1.S1_at	-1.7313555	down	-1.40941	down	1.149378	up	-2.44019	down
OsAffx.13548.1.S1_at	1.5017394	up	1.221112	up	-1.49734	down	1.833792	up
Os.50592.1.S1_x_at	-2.0592074	down	1.054241	up	-1.29197	down	-1.95326	down
Os.23087.1.S1_at	2.5310705	up	-2.52406	down	3.092072	up	1.002779	up
Os.57432.1.S1_a_at	-1.9137774	down	-1.10888	down	-1.10455	down	-2.12216	down
Os.19501.1.S2_a_at	-2.61515	down	1.099078	up	-1.34606	down	-2.3794	down
OsAffx.28880.1.S1_s_	1.411026	up	-1.71062	down	2.094502	up	-1.21232	down
Os.9894.1.S1_at	1.1698686	up	1.917884	up	-2.34821	down	2.243672	up
OsAffx.30125.1.S1_at	-1.3807113	down	2.223091	up	-2.72127	down	1.610106	up
Os.27915.1.S1_at	1.0013564	up	2.345519	up	-1.91658	down	2.348701	up
Os.21566.1.S1_at	-2.1218524	down	1.080225	up	-1.32172	down	-1.96427	down
Os.53723.1.A1_s_at	-2.3937669	down	-1.01776	down	-1.20182	down	-2.43627	down
Os.21178.1.S1_at	-2.5580323	down	1.372147	up	-1.12187	down	-1.86425	down
Os.50990.1.S1_at	1.4858226	up	1.942478	up	-1.58846	down	2.886177	up
Os.16305.1.S1_at	1.9883794	up	1.40629	up	-1.15042	down	2.796238	up
Os.15732.1.S1_s_at	-2.1833625	down	2.991782	up	-2.44751	down	1.370264	up
Os.50956.1.S1_at	1.344557	up	-1.73461	down	2.119355	up	-1.2901	down

Os.9283.1.S1_at	-1.8806889	down	-1.53061	down	1.252779	up	-2.87861	down
Os.33461.2.S1_x_at	-1.5885178	down	-1.37774	down	1.128104	up	-2.18857	down
Os.46074.1.A1_at	-2.736013	down	1.19575	up	-1.46023	down	-2.28812	down
Os.11393.1.S2_at	1.1841966	up	-1.79126	down	2.187367	up	-1.51264	down
Os.51740.1.S1_at	-1.7443353	down	1.049168	up	1.163619	up	-1.66259	down
Os.17160.1.S1_at	-2.464457	down	-1.1194	down	-1.09042	down	-2.75872	down
Os.51744.1.A1_at	2.7002628	up	1.81959	up	-1.49092	down	4.913371	up
Os.51065.1.S1_at	1.2239994	up	2.870542	up	-3.50303	down	3.513541	up
Os.52555.1.S1_at	2.4165006	up	-1.96454	down	2.397393	up	1.230057	up
Os.12105.1.S1_a_at	-1.0146487	down	2.176151	up	-1.7835	down	2.144733	up
Os.14184.1.S1_at	-2.2582006	down	-1.28943	down	1.056913	up	-2.91179	down
Os.49381.1.S1_at	-4.8805027	down	4.020639	up	-4.90407	down	-1.21386	down
Os.45997.1.S1_x_at	-1.0147889	down	-3.74745	down	3.072696	up	-3.80287	down
Os.8275.1.S1_at	1.1445855	up	1.866846	up	-1.53089	down	2.136765	up
Os.9650.1.A1_at	-1.9729486	down	-1.1241	down	-1.08416	down	-2.2178	down
Os.49294.1.S1_at	1.4476854	up	1.486877	up	-1.81125	down	2.15253	up
Os.55007.1.S1_x_at	-1.5564643	down	-1.31025	down	1.07587	up	-2.03936	down
Os.20316.1.S1_at	-2.7956104	down	1.341944	up	-1.63421	down	-2.08325	down
Os.11969.1.S1_at	-1.3715873	down	-1.91282	down	1.570919	up	-2.6236	down
Os.8511.1.S1_s_at	1.2829324	up	6.392592	up	-5.25117	down	8.201263	up
OsAffx.10908.1.S1_at	-1.0154066	down	-1.61986	down	1.971546	up	-1.64482	down
Os.37876.1.S1_at	2.7606807	up	-2.49555	down	3.037148	up	1.106242	up
Os.49872.1.S1_at	1.1913507	up	1.764933	up	-1.4503	down	2.102654	up
Os.49555.1.S1_at	-2.2012599	down	1.529706	up	-1.86154	down	-1.43901	down
Os.50411.1.S1_at	-2.3438628	down	1.521078	up	-1.85099	down	-1.54092	down
OsAffx.24742.1.S1_x_	-1.1977717	down	2.359663	up	-1.9391	down	1.970044	up
Os.20078.1.S1_at	-2.1715221	down	1.547926	up	-1.88361	down	-1.40286	down
Os.25846.1.S1_at	-1.652171	down	1.931717	up	-2.35002	down	1.169199	up
OsAffx.28288.1.S1_s_	-2.4257133	down	1.098041	up	-1.33557	down	-2.20913	down
Os.13521.1.S1_at	2.623173	up	-1.13555	down	1.380433	up	2.310039	up
Os.48101.1.S1_at	1.2901428	up	2.02045	up	-1.66207	down	2.606669	up
Os.24364.1.A1_s_at	1.6670907	up	1.178792	up	-1.43296	down	1.965152	up
OsAffx.481.1.S1_at	-2.009391	down	1.259779	up	-1.53139	down	-1.59504	down
Os.11119.1.S1_a_at	-2.2451127	down	1.234078	up	-1.50008	down	-1.81926	down
Os.51252.1.S1_at	-2.117984	down	1.409375	up	-1.71316	down	-1.50278	down
Os.5927.1.S1_at	3.5805671	up	-1.03729	down	1.260792	up	3.451853	up
Os.27564.1.S1_a_at	1.4663191	up	1.757254	up	-2.13539	down	2.576695	up
Os.10240.1.S1_at	-1.1588471	down	3.472317	up	-2.85751	down	2.996355	up
Os.41335.2.S1_x_at	1.4146957	up	1.632203	up	-1.34344	down	2.309071	up
Os.38052.1.S1_at	-2.7685103	down	1.211478	up	-1.47152	down	-2.28523	down
Os.16867.1.S1_at	-2.44859	down	1.124313	up	-1.36563	down	-2.17785	down
Os.17761.2.S1_x_at	-1.6632429	down	1.791093	up	-2.1751	down	1.076868	up
Os.57449.1.S1_x_at	1.6513548	up	1.835883	up	-2.22859	down	3.031694	up
Os.20404.1.S1_at	1.1836153	up	2.179708	up	-1.79582	down	2.579936	up
Os.21199.1.S1_s_at	-3.1255887	down	1.509189	up	-1.83175	down	-2.07104	down
Os.54001.1.S1_at	-2.0496037	down	-1.216	down	1.001905	up	-2.49232	down
Os.8545.1.S1_s_at	1.8382351	up	1.32016	up	-1.08819	down	2.426765	up

Os.30528.1.S1_at	2.033923	up	-3.16808	down	2.612282	up	-1.55762	down
Os.54880.1.S1_at	-1.2439811	down	-1.39593	down	1.692776	up	-1.73651	down
Os.27660.1.S1_at	-1.870664	down	2.324495	up	-2.8186	down	1.242604	up
Os.17177.1.S1_at	-2.2357767	down	1.691494	up	-2.05096	down	-1.32178	down
Os.49173.1.S1_at	-3.5104077	down	1.039451	up	-1.25996	down	-3.37718	down
Os.14423.1.S1_x_at	1.2285416	up	1.56112	up	-1.89097	down	1.917901	up
Os.49169.1.S1_at	-1.6337456	down	-1.19387	down	1.445852	up	-1.95049	down
Os.12623.1.S1_at	-1.5836565	down	1.748674	up	-2.11754	down	1.1042	up
Os.41771.1.S1_at	-2.461695	down	1.248324	up	-1.51145	down	-1.972	down
Os.49188.1.S1_at	-2.0675147	down	1.173594	up	-1.42084	down	-1.76169	down
OsAffx.16526.1.S1_x_	-1.6800748	down	-1.22018	down	1.008087	up	-2.05	down
Os.49059.1.A1_at	-1.421345	down	-1.85191	down	1.530018	up	-2.6322	down
Os.48846.1.S1_at	-1.5020055	down	-1.2166	down	1.472406	up	-1.82734	down
Os.32813.1.A1_at	-1.6216694	down	1.683799	up	-2.03748	down	1.038312	up
Os.37936.2.S1_a_at	-2.0225306	down	1.354883	up	-1.63938	down	-1.49277	down
Os.4871.1.S1_at	-1.004045	down	1.835063	up	-2.22002	down	1.82767	up
Os.15135.1.S1_at	-2.0529037	down	1.182675	up	-1.43042	down	-1.73581	down
Os.24026.1.S1_at	-2.4752505	down	1.377874	up	-1.66591	down	-1.79643	down
Os.50799.1.S1_at	1.2538506	up	2.192221	up	-1.81331	down	2.748717	up
Os.3663.1.S1_at	-1.0237616	down	2.043277	up	-1.69079	down	1.995852	up
Os.27175.1.S1_a_at	-1.2938371	down	2.491273	up	-3.00992	down	1.925492	up
Os.53953.1.S1_at	-2.627122	down	1.324845	up	-1.6005	down	-1.98297	down
Os.27003.1.S2_a_at	-2.6855385	down	1.622351	up	-1.95962	down	-1.65534	down
Os.51096.1.S1_x_at	-1.5908364	down	1.899631	up	-2.29445	down	1.194108	up
OsAffx.29237.1.S1_at	-1.8435535	down	1.883617	up	-2.27501	down	1.021732	up
Os.17437.1.S1_at	-2.1449609	down	2.454603	up	-2.03271	down	1.144358	up
Os.55033.1.A1_at	-1.5026323	down	2.164759	up	-1.79316	down	1.440645	up
Os.23416.1.S1_at	1.8886852	up	1.455636	up	-1.20661	down	2.749238	up
Os.20865.1.S1_at	-2.1831255	down	1.080293	up	1.11659	up	-2.02086	down
Os.24712.1.A1_s_at	2.7464502	up	-1.51156	down	1.82194	up	1.816969	up
OsAffx.15414.1.S1_x_	1.1440275	up	1.473612	up	-1.7751	down	1.685853	up
Os.8597.1.S1_at	1.4991875	up	1.576745	up	-1.309	down	2.363837	up
Os.12200.1.S1_s_at	-1.3895805	down	1.918363	up	-2.30939	down	1.380534	up
OsAffx.15035.1.S1_at	1.7421488	up	1.250774	up	-1.03907	down	2.179034	up
Os.27058.1.S1_at	-2.0580444	down	-1.0772	down	-1.11728	down	-2.21693	down
Os.8324.1.S1_a_at	1.6255888	up	1.154958	up	-1.38952	down	1.877487	up
Os.7905.1.S1_at	-1.0282508	down	-2.15716	down	1.793057	up	-2.2181	down
Os.27617.1.S1_at	-2.2688746	down	2.322504	up	-2.79297	down	1.023637	up
Os.12281.1.S1_at	1.2162088	up	-1.86165	down	2.238707	up	-1.5307	down
Os.18473.1.S1_at	-2.5516984	down	-1.54995	down	1.289264	up	-3.955	down
Os.26989.1.S1_a_at	-1.5618845	down	-1.09858	down	1.320678	up	-1.71585	down
OsAffx.27868.1.S1_at	-1.5367485	down	-1.38842	down	1.155029	up	-2.13366	down
Os.27330.1.A1_at	2.0604734	up	1.48303	up	-1.78231	down	3.055744	up
Os.46718.1.S1_at	1.5868534	up	1.273015	up	-1.0594	down	2.020088	up
Os.46132.1.S1_at	-2.1633258	down	-1.03717	down	-1.15831	down	-2.24373	down
Os.5843.1.S1_at	1.0160854	up	1.807458	up	-2.17134	down	1.836532	up
Os.24495.2.S1_x_at	-1.1464355	down	2.292293	up	-1.90846	down	1.999496	up

Os.20810.3.S1_x_at	1.9259055	up	1.066127	up	1.126589	up	2.053259	up
Os.7147.1.S1_at	-2.2033737	down	-1.11518	down	1.338966	up	-2.45716	down
Os.6863.1.S1_s_at	1.586006	up	6.003128	up	-5.00067	down	9.520997	up
Os.10755.1.S1_at	1.5180798	up	2.002202	up	-1.66787	down	3.039502	up
OsAffx.19428.1.S1_s_	-3.5526712	down	2.121246	up	-2.54626	down	-1.6748	down
Os.20521.1.S1_at	1.3529897	up	4.101314	up	-4.92165	down	5.549036	up
Os.21231.1.S1_at	-3.027265	down	-1.25638	down	1.047134	up	-3.80339	down
OsAffx.18105.1.S1_at	-2.1542683	down	1.862502	up	-2.23466	down	-1.15665	down
Os.6226.1.S1_at	-1.5496273	down	-1.10267	down	1.322859	up	-1.70872	down
OsAffx.30214.1.S1_x_	-2.1989496	down	1.256268	up	-1.50638	down	-1.75038	down
OsAffx.15781.5.S1_x_	-2.697709	down	1.412985	up	-1.69384	down	-1.90923	down
Os.36122.2.S1_at	1.5294147	up	1.232868	up	-1.47786	down	1.885567	up
Os.46290.1.A1_at	-2.2532737	down	1.32237	up	-1.58491	down	-1.70397	down
Os.57295.1.S1_at	1.4449749	up	-3.8238	down	3.191943	up	-2.64627	down
Os.52966.1.S1_at	-2.1909285	down	1.273751	up	-1.5254	down	-1.72006	down
Os.22627.1.S1_at	2.038563	up	2.545139	up	-2.12583	down	5.188426	up
Os.10746.1.S1_a_at	1.785896	up	1.219419	up	-1.01881	down	2.177756	up
Os.140.3.S1_x_at	-2.0624638	down	4.12589	up	-3.4475	down	2.000467	up
Os.30433.1.S1_at	2.5991673	up	-2.10667	down	2.520166	up	1.23378	up
Os.6057.1.A1_at	1.2324797	up	-1.74187	down	2.083016	up	-1.41331	down
Os.53046.1.S1_s_at	-1.799836	down	-1.1435	down	1.367374	up	-2.0581	down
Os.55694.1.S1_at	-2.386255	down	1.08211	up	1.104901	up	-2.20519	down
Os.27703.1.S1_a_at	3.0837214	up	19.45943	up	-23.2658	down	60.00745	up
Os.54874.1.S1_at	-1.9411618	down	2.212124	up	-2.64469	down	1.139588	up
Os.21318.1.S1_at	-1.2920789	down	2.402665	up	-2.8724	down	1.859535	up
OsAffx.14364.1.S1_s_	3.1935954	up	-1.17703	down	1.40706	up	2.713272	up
Os.6334.1.S1_at	-2.0897415	down	1.843917	up	-1.54271	down	-1.13332	down
Os.27807.1.S1_at	1.0381037	up	2.019558	up	-2.41342	down	2.09651	up
Os.10373.1.S1_at	-1.3900493	down	2.846343	up	-2.38214	down	2.047656	up
Os.14861.2.S1_x_at	-1.9050802	down	-1.10343	down	-1.08272	down	-2.10211	down
Os.4787.1.S1_s_at	-2.098805	down	1.329683	up	-1.58847	down	-1.57843	down
Os.27982.1.S1_at	1.4766039	up	-2.30295	down	1.928018	up	-1.55963	down
Os.6157.1.S1_at	-1.8160688	down	1.966784	up	-2.34898	down	1.08299	up
Os.45998.1.S1_x_at	1.5962273	up	1.575509	up	-1.31953	down	2.51487	up
Os.18633.1.S1_at	-1.4708263	down	-1.3367	down	1.595503	up	-1.96605	down
Os.27312.1.A1_a_at	-2.1791139	down	1.014494	up	-1.21024	down	-2.14798	down
AFFX-Os-actin-5_s_at	2.426228	up	-1.3733	down	1.638208	up	1.766708	up
Os.15436.1.S1_at	-1.0760846	down	2.191313	up	-1.83716	down	2.036376	up
Os.51854.1.S1_at	1.2282821	up	1.770989	up	-1.48494	down	2.175274	up
Os.9132.1.A1_at	-2.2700129	down	1.205065	up	-1.43673	down	-1.88373	down
OsAffx.23371.1.S1_x_	-1.3258864	down	1.72979	up	-2.06093	down	1.304629	up
Os.49679.1.S1_at	1.6922741	up	1.351889	up	-1.13511	down	2.287767	up
Os.7527.1.S1_at	-2.4702911	down	1.045342	up	-1.24487	down	-2.36314	down
OsAffx.27508.7.S1_s_	-2.9713576	down	1.790756	up	-2.13243	down	-1.65928	down
Os.1005.1.S1_at	1.1983063	up	1.404033	up	-1.67106	down	1.682461	up
OsAffx.12401.1.S1_at	-2.541806	down	1.113645	up	-1.32524	down	-2.28242	down
Os.27698.1.A1_x_at	-2.0794046	down	-1.03941	down	-1.14483	down	-2.16136	down

OsAffx.4480.1.S1_at	1.4294567	up	1.547537	up	-1.30058	down	2.212138	up
OsAffx.21096.1.S1_at	-1.5551444	down	1.80606	up	-2.14898	down	1.161346	up
Os.22676.1.S1_at	-1.1218911	down	2.30076	up	-1.93374	down	2.050787	up
Os.12629.1.S2_at	1.0059255	up	-13.6681	down	11.48799	up	-13.5876	down
Os.21636.1.S2_a_at	1.4328873	up	1.439902	up	-1.21028	down	2.063218	up
Os.24665.1.S2_at	-2.4978	down	1.400541	up	-1.66601	down	-1.78345	down
Os.49111.1.S1_at	-1.0717655	down	-2.02225	down	1.700303	up	-2.16738	down
Os.27504.1.S1_at	-2.3544927	down	1.498888	up	-1.78216	down	-1.57083	down
Os.44431.2.S1_x_at	-1.0312383	down	2.0478	up	-1.72295	down	1.985768	up
Os.17938.1.A1_s_at	-1.49411	down	-1.94923	down	1.64066	up	-2.91237	down
Os.51491.1.S1_at	2.148562	up	-28.1238	down	23.67235	up	-13.0896	down
Os.20617.1.S1_at	1.0850726	up	-2.47487	down	2.083503	up	-2.28083	down
Os.8570.3.S1_s_at	1.44865	up	1.458419	up	-1.22807	down	2.112738	up
Os.6230.1.S1_at	-1.4500532	down	1.707281	up	-2.02746	down	1.177392	up
Os.8398.1.S1_a_at	1.1485091	up	1.93775	up	-2.30048	down	2.225524	up
Os.51363.1.S1_at	-3.542751	down	1.789312	up	-2.1241	down	-1.97995	down
Os.5651.1.S1_a_at	-1.0544579	down	2.049055	up	-1.72633	down	1.943231	up
Os.50753.1.S1_at	-2.9484837	down	1.55697	up	-1.84713	down	-1.89373	down
Os.15931.1.S1_x_at	2.2001832	up	1.02589	up	1.156311	up	2.257146	up
OsAffx.5257.1.S1_at	-1.3089776	down	1.882983	up	-2.23359	down	1.438515	up
Os.17181.1.S1_at	-1.6978624	down	-1.17349	down	1.391361	up	-1.99242	down
Os.8721.1.S1_at	-2.259276	down	-1.13772	down	-1.04192	down	-2.57043	down
Os.20857.1.S1_at	1.170876	up	2.071938	up	-1.7481	down	2.425983	up
Os.49439.1.S1_at	-1.6620214	down	2.072404	up	-1.74851	down	1.246918	up
Os.26772.3.S1_at	-2.3016362	down	1.178835	up	-1.3971	down	-1.95247	down
Os.20863.1.S1_at	2.1849723	up	-1.99162	down	1.681507	up	1.097083	up
OsAffx.8593.1.A1_at	2.065869	up	-1.08597	down	1.286238	up	1.902328	up
Os.50455.1.S1_at	2.959879	up	5.086209	up	-4.29429	down	15.05456	up
Os.14285.2.S1_at	-1.3240966	down	2.111117	up	-1.78264	down	1.594383	up
Os.17058.1.S1_at	-1.6487021	down	3.789444	up	-3.20147	down	2.298441	up
Os.54961.1.S1_at	1.7049822	up	1.268874	up	-1.07201	down	2.163408	up
Os.623.3.S1_x_at	4.0077057	up	-2.29628	down	2.71669	up	1.745301	up
Os.11399.1.S1_at	-1.192272	down	2.620295	up	-2.21518	down	2.197732	up
Os.51134.1.S1_at	1.3588191	up	1.562387	up	-1.32084	down	2.123001	up
Os.19541.1.S1_at	-2.353709	down	1.287072	up	-1.52216	down	-1.82873	down
OsAffx.28769.2.S1_at	-1.1735777	down	1.834135	up	-2.16891	down	1.562858	up
Os.8454.1.S1_s_at	-1.8393693	down	-1.14652	down	-1.03103	down	-2.10888	down
Os.50126.1.S1_at	4.728852	up	-1.0792	down	1.275606	up	4.38183	up
Os.49335.1.S1_x_at	-2.1707647	down	1.299372	up	-1.53524	down	-1.67063	down
Os.10829.2.S1_x_at	-1.5265794	down	2.58196	up	-2.18592	down	1.691337	up
Os.9764.1.S1_at	-2.2434235	down	1.943015	up	-2.29497	down	-1.15461	down
Os.42069.1.S1_x_at	-1.2255826	down	2.19622	up	-2.59367	down	1.79198	up
Os.12528.1.S1_x_at	-1.9042166	down	-2.69819	down	3.18552	up	-5.13793	down
OsAffx.29114.1.S1_at	-1.1538001	down	1.815549	up	-2.14297	down	1.573539	up
Os.9540.1.S1_at	-2.731301	down	1.448904	up	-1.70991	down	-1.88508	down
Os.27964.1.S1_at	1.0148107	up	2.220643	up	-1.88204	down	2.253533	up
Os.18134.1.S1_at	-2.4153354	down	1.427856	up	-1.68473	down	-1.69158	down

Os.23925.1.S1_at	-2.3814046	down	1.194765	up	-1.40921	down	-1.9932	down
Os.12814.2.S1_at	-2.7209628	down	1.397045	up	-1.64746	down	-1.94766	down
Os.24674.1.S1_at	-2.3129852	down	1.690565	up	-1.43373	down	-1.36817	down
Os.27098.2.A1_s_at	-1.4782022	down	2.267554	up	-2.673	down	1.533994	up
Os.50391.2.S1_at	-2.1967175	down	1.953143	up	-1.6569	down	-1.12471	down
Os.15904.1.S1_at	-2.0272143	down	1.338877	up	-1.57811	down	-1.51412	down
Os.19378.1.S1_at	-2.17218	down	-1.00859	down	-1.16825	down	-2.19084	down
Os.30032.1.S1_at	-6.228989	down	2.084614	up	-2.45619	down	-2.98808	down
Os.27382.2.S1_at	-2.5400245	down	1.879195	up	-2.21413	down	-1.35166	down
Os.21254.1.S1_at	-1.668131	down	1.894764	up	-2.23203	down	1.13586	up
Os.38992.1.A1_x_at	9.426829	up	-3.79206	down	4.465855	up	2.485942	up
Os.14145.1.A1_at	-3.0374086	down	1.241274	up	-1.4615	down	-2.44701	down
Os.34984.1.A1_at	-1.8533101	down	-1.04303	down	1.227837	up	-1.93305	down
Os.51668.1.S1_at	-1.6287178	down	2.314785	up	-1.96644	down	1.421231	up
Os.49114.1.S1_at	-1.795972	down	-1.17461	down	-1.00215	down	-2.10957	down
Os.7801.1.S1_at	-1.471681	down	1.891303	up	-2.22541	down	1.285132	up
Os.49503.1.S1_at	-3.7226245	down	3.205506	up	-3.77119	down	-1.16132	down
Os.27449.1.S1_at	-1.8189647	down	1.779149	up	-2.09273	down	-1.02238	down
OsAffx.25749.1.S1_at	2.8287146	up	-1.11577	down	1.312421	up	2.535205	up
Os.15417.1.S1_at	-1.1188257	down	2.109289	up	-2.48059	down	1.88527	up
Os.15931.1.S1_at	2.342958	up	-1.14656	down	1.34807	up	2.043464	up
Os.23252.1.S1_at	-1.0043826	down	4.678854	up	-3.98026	down	4.658438	up
Os.22585.1.S1_at	-2.1771634	down	1.453662	up	-1.70852	down	-1.49771	down
Os.33131.1.A1_at	2.2827637	up	-1.97323	down	2.318695	up	1.156869	up
OsAffx.21988.1.S1_at	2.6455958	up	-1.19527	down	1.404184	up	2.213385	up
Os.14384.1.S1_at	-1.225553	down	2.218026	up	-2.60549	down	1.809817	up
Os.20700.1.S1_at	2.4664407	up	-1.00207	down	1.176793	up	2.461344	up
Os.55253.1.S1_at	54.08567	up	-1.13932	down	1.337871	up	47.4721	up
Os.16028.1.S1_at	-2.0378077	down	1.084136	up	-1.27297	down	-1.87966	down
Os.27571.1.S1_at	-2.0911028	down	1.251652	up	-1.46963	down	-1.67067	down
Os.52882.2.S1_at	-2.0751178	down	1.261566	up	-1.48104	down	-1.64487	down
Os.11213.1.S1_at	-2.125799	down	1.139989	up	-1.33812	down	-1.86475	down
Os.9072.1.S1_at	1.8810471	up	1.560872	up	-1.3298	down	2.936074	up
Os.18490.3.S1_at	-1.5060116	down	3.202662	up	-2.72863	down	2.126585	up
Os.42784.1.S1_at	-1.311871	down	-6.14405	down	7.211269	up	-8.0602	down
Os.7232.1.S1_at	-2.1444225	down	-1.10195	down	-1.0648	down	-2.36305	down
Os.7285.1.S1_at	1.1098247	up	2.016246	up	-1.71855	down	2.23768	up
Os.15985.1.S1_at	-2.1965256	down	1.047089	up	-1.22781	down	-2.09775	down
Os.17497.1.S1_a_at	-2.3090038	down	-1.16896	down	-1.00297	down	-2.69913	down
Os.10355.1.S1_at	1.2796704	up	1.677337	up	-1.43087	down	2.146439	up
Os.11879.1.S1_at	-2.793807	down	1.331442	up	-1.56077	down	-2.09833	down
Os.8494.1.S1_at	-2.003203	down	1.092414	up	-1.28012	down	-1.83374	down
Os.53784.1.S1_at	-2.0208247	down	1.566905	up	-1.83502	down	-1.28969	down
Os.49785.1.S1_at	1.1738951	up	1.690773	up	-1.97972	down	1.98479	up
Os.52408.1.S1_at	-2.2243917	down	1.118666	up	-1.30969	down	-1.98843	down
Os.19289.1.S1_a_at	-1.6470072	down	-1.26696	down	1.082428	up	-2.0867	down
Os.20501.1.S1_at	1.1007813	up	-1.89166	down	2.213692	up	-1.71847	down

Os.8314.1.S1_at	1.3490902	up	1.580248	up	-1.84893	down	2.131898	up
Os.12643.1.S1_a_at	-2.4633522	down	1.33602	up	-1.56313	down	-1.8438	down
Os.7786.1.S1_at	-1.2617964	down	-1.6839	down	1.43926	up	-2.12474	down
Os.37728.1.S1_x_at	1.0673976	up	9.217673	up	-7.88147	down	9.838923	up
Os.13489.1.S1_at	-1.9146471	down	-1.14662	down	-1.01989	down	-2.19538	down
Os.51235.1.S1_at	1.5048636	up	-2.17111	down	2.538825	up	-1.44273	down
Os.8281.1.S1_at	-1.8259451	down	-1.11535	down	-1.04842	down	-2.03657	down
Os.21442.1.S1_at	-3.0169313	down	2.011486	up	-2.35178	down	-1.49985	down
Os.52604.1.A1_at	-1.1526582	down	2.101841	up	-1.79787	down	1.823473	up
Os.26887.1.A1_at	-1.4177274	down	2.362329	up	-2.02099	down	1.666279	up
Os.53467.1.S1_at	-1.6059049	down	2.530925	up	-2.1658	down	1.576012	up
Os.17149.1.S1_at	-2.9345703	down	1.691703	up	-1.97686	down	-1.73468	down
Os.10096.1.S1_a_at	2.3038168	up	-2.31889	down	1.984882	up	-1.00654	down
Os.9066.1.S1_s_at	-1.2339315	down	2.440488	up	-2.08972	down	1.977814	up
Os.9796.1.S1_at	-2.2562697	down	1.282975	up	-1.49636	down	-1.75862	down
Os.15822.1.S1_s_at	1.6116234	up	-2.34847	down	2.737766	up	-1.45721	down
Os.53970.1.S1_at	-3.036807	down	1.063754	up	-1.24	down	-2.8548	down
Os.8531.1.S1_at	-1.0774946	down	1.792721	up	-2.08963	down	1.663786	up
Os.19863.1.S1_a_at	2.0013013	up	-1.44949	down	1.689341	up	1.380696	up
OsAffx.13735.1.S1_at	-1.1842594	down	2.026386	up	-1.739	down	1.7111	up
Os.12672.1.S1_a_at	-2.3875413	down	1.034992	up	-1.20575	down	-2.30682	down
OsAffx.8599.1.S1_at	2.06251	up	1.081722	up	1.076798	up	2.231063	up
Os.11718.1.S1_at	1.6238865	up	1.64387	up	-1.41198	down	2.669459	up
Os.50585.1.S1_at	-1.9022164	down	1.745261	up	-2.03137	down	-1.08993	down
Os.47928.1.S3_at	-2.177279	down	1.217279	up	-1.41676	down	-1.78864	down
Os.17804.1.S1_at	-2.0373003	down	1.491923	up	-1.73624	down	-1.36555	down
Os.34494.1.S1_at	-2.3426669	down	4.432497	up	-3.81065	down	1.892073	up
Os.35024.1.S1_at	1.1411254	up	1.760493	up	-2.04733	down	2.008943	up
Os.26657.1.A1_s_at	-1.9376674	down	-1.08771	down	-1.06897	down	-2.10763	down
Os.53399.1.S1_at	-2.024602	down	1.403778	up	-1.63212	down	-1.44225	down
Os.19423.1.S1_at	-2.0398097	down	1.096162	up	-1.27446	down	-1.86086	down
Os.33639.1.S1_at	-2.0726867	down	1.666924	up	-1.93805	down	-1.24342	down
Os.47343.2.S1_x_at	-2.4227371	down	2.036273	up	-2.36735	down	-1.18979	down
Os.275.1.S1_at	-3.0561447	down	1.613476	up	-1.87513	down	-1.89414	down
Os.37890.1.S1_at	1.0917305	up	-2.24456	down	1.931445	up	-2.05596	down
Os.5378.2.S1_at	-2.177846	down	1.09137	up	1.06474	up	-1.99552	down
OsAffx.24682.1.S1_s_	1.8987025	up	1.444404	up	-1.24312	down	2.742494	up
Os.47923.2.A1_at	-1.5665249	down	-1.34709	down	1.15941	up	-2.11026	down
Os.52189.1.S1_at	-2.044957	down	2.714404	up	-3.15356	down	1.327365	up
Os.49410.1.A1_at	2.5898328	up	1.130879	up	1.027034	up	2.928788	up
Os.27817.1.A1_at	-1.6019156	down	-1.42405	down	1.22618	up	-2.28121	down
Os.32115.1.S1_at	-1.3349109	down	1.846811	up	-2.14471	down	1.383471	up
Os.12403.1.S1_a_at	-2.1171672	down	1.258772	up	-1.4617	down	-1.68193	down
Os.11963.1.S1_at	1.3117923	up	1.653296	up	-1.42431	down	2.168781	up
Os.55392.1.S1_at	1.4371258	up	2.182898	up	-1.88131	down	3.137098	up
Os.25141.1.S1_at	-1.2809957	down	2.53241	up	-2.1847	down	1.976908	up
Os.9660.1.S1_at	-4.1453176	down	13.55628	up	-15.7131	down	3.270264	up

Os.26436.1.S1_at	-1.0712833	down	1.804158	up	-2.09081	down	1.684109	up
Os.51261.1.S1_at	1.8298641	up	1.287747	up	-1.11168	down	2.356403	up
Os.25355.1.A1_at	-1.508634	down	-1.49421	down	1.290065	up	-2.25421	down
OsAffx.28166.1.S1_at	-1.007882	down	-3.89851	down	3.366231	up	-3.92923	down
Os.49524.1.S1_at	2.4500537	up	1.392412	up	-1.20242	down	3.411484	up
Os.27967.1.A1_at	-1.1229047	down	3.434138	up	-2.96581	down	3.058263	up
Os.10803.1.S2_at	-1.9430853	down	2.090942	up	-1.80581	down	1.076094	up
Os.15065.1.S1_at	-1.0522398	down	2.457743	up	-2.12368	down	2.335726	up
Os.51145.1.S1_at	1.8030548	up	1.379906	up	-1.1925	down	2.488046	up
Os.8574.1.S1_at	2.2073092	up	-1.18874	down	1.375511	up	1.856845	up
Os.55328.1.S1_at	-1.4132015	down	-1.42241	down	1.645799	up	-2.01016	down
Os.5742.1.S1_a_at	-2.0064483	down	-1.06586	down	-1.08539	down	-2.13858	down
Os.49441.1.S1_at	-1.6962065	down	-1.20287	down	1.040126	up	-2.04032	down
Os.11960.1.S1_at	-1.1876729	down	-1.74477	down	1.509171	up	-2.07222	down
Os.10176.1.S1_at	1.4322811	up	1.953126	up	-1.68967	down	2.797426	up
Os.8957.1.S1_at	2.4559011	up	-1.83731	down	2.12372	up	1.336684	up
Os.32602.2.S1_x_at	1.8319926	up	1.209584	up	-1.04655	down	2.21595	up
Os.4327.1.S1_at	1.1064516	up	2.349459	up	-2.03396	down	2.599562	up
Os.12176.1.S1_at	1.0017418	up	2.918957	up	-2.52776	down	2.924042	up
Os.23458.1.A1_x_at	-1.3576286	down	-1.36199	down	1.572671	up	-1.84907	down
Os.15794.1.S1_at	-2.0156438	down	1.137203	up	-1.3131	down	-1.77246	down
Os.8488.1.S1_s_at	1.2585475	up	1.63025	up	-1.41217	down	2.051747	up
Os.49573.1.S1_at	-1.6464974	down	-1.56691	down	1.3584	up	-2.57991	down
Os.6297.1.S1_at	-1.1523837	down	-1.87224	down	2.159596	up	-2.15753	down
Os.14831.1.S1_at	-2.3803189	down	1.153089	up	-1.33006	down	-2.0643	down
Os.8413.2.A1_a_at	3.8775072	up	1.089519	up	-1.25671	down	4.224617	up
OsAffx.16128.1.S1_s_	2.1522558	up	-1.382	down	1.593952	up	1.557347	up
Os.27051.1.A1_at	-2.4864347	down	1.456167	up	-1.67949	down	-1.70752	down
OsAffx.14375.1.S1_at	1.542909	up	1.40911	up	-1.22316	down	2.174128	up
OsAffx.11631.1.S1_x_	1.4221829	up	1.533631	up	-1.33143	down	2.181104	up
Os.15252.2.S1_a_at	-1.2464867	down	-1.69674	down	1.473179	up	-2.11497	down
Os.12209.1.S1_a_at	-1.6360096	down	-1.11902	down	1.288709	up	-1.83073	down
Os.25811.1.S1_at	-1.9358007	down	-1.18187	down	1.026547	up	-2.28786	down
Os.50253.1.S1_at	-3.0416253	down	1.670877	up	-1.92358	down	-1.82038	down
Os.5201.2.S1_x_at	1.4862481	up	1.666936	up	-1.44806	down	2.47748	up
Os.17491.1.S1_at	2.4002047	up	-1.79343	down	2.064465	up	1.338335	up
Os.5189.1.S1_at	-2.2493472	down	1.293216	up	-1.12353	down	-1.73934	down
Os.5095.1.S1_at	1.6329025	up	1.247152	up	-1.0842	down	2.036478	up
Os.49405.1.A1_at	-2.5784414	down	1.422626	up	-1.23744	down	-1.81245	down
Os.49819.1.S1_at	-2.182592	down	3.454398	up	-3.97113	down	1.582705	up
Os.20202.1.S1_at	1.2503148	up	1.636506	up	-1.42404	down	2.046148	up
OsAffx.25846.1.S1_s_	-1.343428	down	2.486399	up	-2.85714	down	1.850786	up
Os.27169.1.S1_s_at	-2.3487737	down	1.536512	up	-1.7654	down	-1.52864	down
Os.4160.1.S1_at	1.6533116	up	1.345281	up	-1.17092	down	2.224169	up
Os.9416.1.S1_at	1.2373793	up	1.707024	up	-1.48588	down	2.112236	up
Os.8144.1.S1_at	-2.1434226	down	-1.02311	down	-1.12279	down	-2.19296	down
Os.9331.1.S1_at	1.1157526	up	2.059766	up	-1.79383	down	2.298189	up

OsAffx.13282.1.S1_s_	1.0819185	up	-2.65438	down	2.312161	up	-2.4534	down
Os.5039.1.S1_at	2.23349	up	-1.19927	down	1.376755	up	1.862377	up
Os.51954.1.S1_at	-1.1239241	down	2.058536	up	-1.79339	down	1.831561	up
Os.15302.1.S1_at	-1.4559273	down	-1.48824	down	1.707759	up	-2.16678	down
Os.33575.1.S1_at	-1.1702826	down	2.158774	up	-1.8815	down	1.844661	up
Os.52597.1.S1_at	-1.4670665	down	-3.83324	down	4.398035	up	-5.62361	down
Os.5334.2.S1_a_at	1.4657463	up	1.408152	up	-1.22733	down	2.063994	up
Os.49397.1.S1_x_at	-2.1851344	down	1.642098	up	-1.43132	down	-1.3307	down
Os.5476.1.S1_at	1.9471712	up	1.030246	up	1.113493	up	2.006065	up
Os.8517.1.S1_at	-1.4195342	down	2.423083	up	-2.11225	down	1.706956	up
OsAffx.24704.1.S1_s_	1.5545692	up	-1.88495	down	2.162077	up	-1.21252	down
Os.15696.1.S1_a_at	-1.0940086	down	-2.28269	down	1.990646	up	-2.49728	down
Os.8290.1.S1_at	-2.351044	down	-1.20159	down	1.048631	up	-2.825	down
Os.10505.1.S1_at	1.0847123	up	2.404927	up	-2.0992	down	2.608654	up
Os.140.1.A1_s_at	-2.3531153	down	1.172308	up	-1.02334	down	-2.00725	down
Os.33587.1.S1_x_at	-2.1008825	down	1.059768	up	-1.21381	down	-1.9824	down
Os.5051.1.S1_at	-1.9505911	down	-1.05966	down	-1.08074	down	-2.06696	down
Os.38236.1.S1_at	-2.552916	down	1.862459	up	-2.13253	down	-1.37072	down
Os.51115.1.S1_x_at	-1.4641728	down	-1.3056	down	1.494895	up	-1.91163	down
Os.7971.2.S1_s_at	1.1704587	up	1.757286	up	-1.53489	down	2.056831	up
OsAffx.22867.1.S1_x_	-1.5919701	down	-1.35556	down	1.184049	up	-2.15802	down
Os.52645.1.S1_at	1.2483302	up	3.304324	up	-2.88672	down	4.124888	up
Os.38999.1.S1_at	1.2597402	up	-4.90368	down	5.611989	up	-3.89261	down
OsAffx.18839.1.S1_at	-2.0196831	down	1.363624	up	-1.19178	down	-1.48112	down
Os.10355.1.S1_x_at	1.242375	up	1.888243	up	-2.16044	down	2.345905	up
Os.6901.1.S1_at	3.1549985	up	-4.08507	down	3.570696	up	-1.29479	down
Os.17532.2.S1_s_at	-2.0571566	down	1.368557	up	-1.56497	down	-1.50316	down
Os.21387.2.S1_s_at	-2.1183474	down	1.221494	up	-1.3967	down	-1.73423	down
Os.46470.2.S1_x_at	1.0504683	up	-1.82594	down	2.086787	up	-1.73821	down
Os.6010.1.S1_at	-2.0165057	down	-1.01779	down	-1.12236	down	-2.05239	down
Os.11580.1.S1_at	2.070585	up	3.0112	up	-2.63656	down	6.234946	up
Os.53704.1.S1_x_at	1.6608405	up	3.022362	up	-3.45124	down	5.019661	up
Os.8521.1.S1_at	1.0817283	up	2.034513	up	-1.78176	down	2.20079	up
Os.6210.1.S1_at	-2.8167827	down	1.41014	up	-1.60999	down	-1.99752	down
Os.4506.3.S1_x_at	-2.4416602	down	1.432922	up	-1.25563	down	-1.70397	down
OsAffx.12986.1.S1_at	6.402714	up	-2.12336	down	2.422899	up	3.015373	up
Os.24952.1.S1_at	1.0448971	up	-2.34862	down	2.058296	up	-2.24771	down
Os.34466.1.S1_s_at	1.8886669	up	2.871362	up	-2.51696	down	5.423046	up
Os.2010.1.S1_at	-1.7917097	down	1.837208	up	-2.09588	down	1.025394	up
Os.3655.1.S1_at	-1.4405766	down	2.282431	up	-2.60363	down	1.584387	up
Os.25071.1.A1_at	-4.124207	down	1.92303	up	-2.19354	down	-2.14464	down
Os.19034.1.S1_at	-1.5729935	down	-1.43077	down	1.254688	up	-2.25059	down
Os.11150.1.S1_at	-1.5816792	down	-1.79047	down	2.041437	up	-2.83194	down
Os.49502.1.S1_at	-1.3289492	down	1.969959	up	-2.24486	down	1.482343	up
OsAffx.17384.1.S1_s_	1.3760937	up	1.854993	up	-1.62811	down	2.552644	up
Os.24041.1.A1_at	1.2789271	up	4.855981	up	-5.53147	down	6.210445	up
Os.32955.1.A1_at	-2.015875	down	1.0138	up	-1.15449	down	-1.98843	down

Os.50318.1.S1_at	-2.0241287	down	1.314308	up	-1.15426	down	-1.54007	down
Os.37902.1.S1_at	1.741149	up	2.540648	up	-2.23193	down	4.423647	up
Os.6610.1.S1_at	1.1672653	up	1.567812	up	-1.78435	down	1.830052	up
Os.6592.1.S1_a_at	1.3765382	up	4.256063	up	-3.73988	down	5.858633	up
Os.14920.1.S1_s_at	-1.3099159	down	2.195212	up	-1.92922	down	1.675842	up
Os.24889.1.S1_at	-2.0324929	down	1.110293	up	-1.26334	down	-1.83059	down
Os.48739.1.S1_x_at	1.8771292	up	1.165145	up	-1.02405	down	2.187128	up
Os.11309.1.S1_x_at	1.0797495	up	2.287474	up	-2.01161	down	2.469899	up
Os.7309.2.S1_at	-1.7559956	down	2.478768	up	-2.17994	down	1.411603	up
OsAffx.2319.1.S1_at	-1.7910268	down	-1.14213	down	1.004928	up	-2.04559	down
Os.7063.1.S1_at	-2.077929	down	1.357388	up	-1.54203	down	-1.53083	down
Os.50402.1.S1_at	1.5620779	up	1.648365	up	-1.45119	down	2.574875	up
Os.12591.2.S1_x_at	1.4536265	up	-2.61305	down	2.300683	up	-1.79761	down
Os.55618.1.S1_at	-2.2595983	down	4.243166	up	-4.81578	down	1.877841	up
Os.52137.1.S1_at	1.3462917	up	1.588825	up	-1.39997	down	2.139022	up
Os.14766.1.S1_at	-1.8869373	down	-1.24135	down	1.093895	up	-2.34234	down
Os.14831.3.S1_x_at	-2.202564	down	1.296778	up	-1.47151	down	-1.69849	down
Os.17158.1.S1_at	-1.7059224	down	2.376432	up	-2.69555	down	1.393048	up
Os.53950.1.S1_at	1.1019162	up	4.241302	up	-4.80959	down	4.673559	up
Os.23606.1.S1_at	-1.313534	down	3.028574	up	-2.67104	down	2.305668	up
Os.18482.1.S1_at	1.2358009	up	-2.1249	down	1.874155	up	-1.71945	down
Os.1818.1.S1_at	1.1050352	up	-4.15816	down	3.667576	up	-3.76292	down
Os.19046.1.S1_at	-1.4489081	down	-1.39233	down	1.228575	up	-2.01736	down
Os.11202.1.S1_at	1.3471614	up	2.181667	up	-1.92544	down	2.939058	up
OsAffx.31409.1.S1_s_	-1.4066032	down	3.029203	up	-2.6761	down	2.153559	up
Os.53962.1.S1_at	-2.500057	down	-1.18309	down	1.045337	up	-2.9578	down
Os.35921.2.S1_x_at	2.40411	up	1.114765	up	-1.26163	down	2.680017	up
OsAffx.6171.1.S1_at	2.253501	up	-22.6803	down	25.66281	up	-10.0645	down
OsAffx.23247.1.S1_at	2.4868813	up	1.927792	up	-1.70466	down	4.79419	up
Os.28079.2.S1_s_at	-1.6078042	down	1.909506	up	-2.15897	down	1.187649	up
Os.1043.1.S1_at	1.3901615	up	-3.12659	down	2.765341	up	-2.24908	down
Os.17070.1.S1_at	1.343065	up	1.900996	up	-2.14912	down	2.553161	up
Os.23033.1.S1_s_at	-2.3803344	down	1.48597	up	-1.31483	down	-1.60187	down
Os.28203.1.S1_a_at	-1.1472622	down	2.215719	up	-1.96178	down	1.93131	up
Os.33102.1.S1_at	-3.35026	down	1.896799	up	-1.67984	down	-1.76627	down
Os.38849.1.S1_at	-1.341552	down	2.163741	up	-1.91672	down	1.612864	up
Os.10705.1.S1_at	-1.650419	down	-2.07445	down	1.837738	up	-3.42372	down
Os.9893.1.S1_at	2.3449242	up	1.300489	up	-1.46789	down	3.049548	up
OsAffx.19451.3.S1_x_	-2.9787061	down	1.970834	up	-2.22427	down	-1.51139	down
Os.32889.1.S1_at	-2.1269255	down	1.170095	up	-1.32055	down	-1.81774	down
Os.24913.1.A1_at	-1.88008	down	-3.24284	down	3.659789	up	-6.0968	down
Os.9216.1.S1_at	2.3396194	up	-9.8891	down	8.763855	up	-4.2268	down
Os.35273.1.S1_at	-1.3162819	down	1.78847	up	-2.01751	down	1.358729	up
Os.10348.1.S1_at	-1.1652577	down	2.182441	up	-2.46173	down	1.872926	up
Os.15830.1.S1_at	2.6177847	up	-4.66729	down	5.263609	up	-1.78292	down
Os.23276.1.S2_at	-2.2548242	down	1.27257	up	-1.43479	down	-1.77187	down
Os.53940.1.S2_at	-1.7633692	down	-1.13178	down	1.275982	up	-1.99575	down

Os.7144.2.S1_a_at	-1.361438	down	3.073638	up	-2.72636	down	2.257641	up
Os.820.1.S1_s_at	1.6316614	up	2.059259	up	-1.82682	down	3.360014	up
Os.16902.1.S1_at	1.2044585	up	1.60767	up	-1.81221	down	1.936372	up
Os.9913.1.S1_at	2.2055426	up	-2.61797	down	2.950969	up	-1.18699	down
Os.17419.1.S1_at	1.0161234	up	-2.98955	down	3.369792	up	-2.94211	down
Os.54111.1.S1_at	-2.7816525	down	1.245133	up	-1.40317	down	-2.23402	down
Os.25164.1.A1_at	-1.7663734	down	-1.36031	down	1.207268	up	-2.40281	down
Os.49562.1.A1_at	-2.2141988	down	1.766963	up	-1.99067	down	-1.25311	down
Os.13980.1.S1_at	-2.1467037	down	2.229669	up	-1.97923	down	1.038648	up
Os.27178.1.S1_s_at	-2.6139486	down	1.236265	up	-1.39265	down	-2.11439	down
OsAffx.7843.1.S1_at	1.4413946	up	-1.80807	down	2.036283	up	-1.25439	down
Os.20403.2.S1_x_at	1.1019093	up	3.036287	up	-2.69623	down	3.345712	up
Os.4978.1.S2_at	-1.3826288	down	-1.69567	down	1.506217	up	-2.34448	down
Os.55279.1.S1_at	3.6569276	up	-2.1524	down	1.912069	up	1.699004	up
Os.27822.1.A1_at	-3.0086198	down	1.688216	up	-1.50045	down	-1.78213	down
Os.15210.2.S1_x_at	2.7614446	up	1.090668	up	1.031498	up	3.011821	up
Os.14809.1.S1_at	-1.047535	down	2.42609	up	-2.15661	down	2.315999	up
OsAffx.22964.2.S1_at	2.1632044	up	-1.59822	down	1.420712	up	1.353513	up
OsAffx.7566.1.S1_s_a	1.6153163	up	1.412509	up	-1.25605	down	2.281649	up
Os.27052.1.S1_at	-2.511532	down	1.551286	up	-1.74441	down	-1.619	down
Os.11330.1.S2_at	1.1292392	up	2.618089	up	-2.94351	down	2.956449	up
OsAffx.16271.1.S1_at	1.54221	up	-2.97331	down	2.644654	up	-1.92795	down
Os.17768.1.S1_at	-2.2502682	down	1.194118	up	-1.3421	down	-1.88446	down
Os.21108.1.S1_at	1.4498663	up	1.599057	up	-1.79666	down	2.318419	up
Os.47730.2.S1_x_at	3.0617113	up	1.279609	up	-1.43767	down	3.917794	up
Os.50825.1.S1_s_at	-1.9173888	down	-1.59686	down	1.421307	up	-3.0618	down
Os.10926.1.S1_at	2.0502515	up	1.531196	up	-1.36293	down	3.139337	up
Os.21264.1.S1_s_at	-2.4202416	down	1.06594	up	-1.19743	down	-2.27052	down
Os.35901.1.S1_at	-2.4202201	down	1.251491	up	-1.40546	down	-1.93387	down
Os.37320.1.S1_at	-2.0507724	down	-1.18338	down	1.328963	up	-2.42685	down
Os.50391.1.S1_at	-2.4879131	down	1.430257	up	-1.60576	down	-1.73949	down
Os.38086.1.S1_at	-2.2092116	down	1.277922	up	-1.4341	down	-1.72875	down
Os.47022.1.A1_at	-2.9601989	down	2.189698	up	-1.95148	down	-1.35188	down
Os.1310.1.S1_a_at	1.2489004	up	-3.50179	down	3.120952	up	-2.8039	down
Os.30886.1.S1_at	2.1679895	up	-1.32222	down	1.483356	up	1.639655	up
Os.57408.1.S1_at	-1.4414337	down	-1.43542	down	1.279539	up	-2.06906	down
Os.23405.1.S1_at	2.6237283	up	-1.36057	down	1.52612	up	1.9284	up
Os.16327.1.S1_at	-1.9778322	down	2.714092	up	-2.42011	down	1.372256	up
Os.19070.1.S1_at	-2.0602612	down	-1.39613	down	1.245611	up	-2.87638	down
Os.7870.1.S1_at	1.7347047	up	1.158277	up	-1.29823	down	2.009269	up
OsAffx.25027.1.S1_s_	1.676048	up	1.187644	up	-1.33108	down	1.990548	up
Os.37563.1.S1_at	-1.3431215	down	2.307305	up	-2.05873	down	1.717868	up
Os.54497.1.S1_at	-2.4372044	down	-1.03545	down	-1.08191	down	-2.5236	down
Os.23550.1.S2_at	-1.6273338	down	-1.25256	down	1.118159	up	-2.03833	down
Os.27524.2.S1_at	-1.0706574	down	2.093116	up	-2.34465	down	1.954982	up
Os.7447.1.S1_a_at	-3.0391998	down	1.202711	up	-1.347	down	-2.52696	down
Os.37466.1.S1_s_at	1.2262083	up	1.842272	up	-1.64502	down	2.259009	up

Os.22271.2.S1_x_at	2.3098238	up	2.00327	up	-1.789	down	4.627201	up
Os.10146.1.S1_at	1.9348221	up	-1.83647	down	2.056198	up	1.053553	up
Os.17804.2.S1_at	-2.006238	down	1.556036	up	-1.38979	down	-1.28933	down
Os.7151.1.S1_at	-1.7226183	down	-2.15819	down	1.927823	up	-3.71773	down
Os.19410.3.S1_at	-1.497531	down	-1.19773	down	1.340347	up	-1.79364	down
OsAffx.14113.1.S1_at	1.1264919	up	-2.41129	down	2.698397	up	-2.14053	down
Os.16898.2.S1_at	-2.1764326	down	1.132794	up	-1.0123	down	-1.9213	down
Os.49559.1.S1_at	1.0894703	up	1.750811	up	-1.95909	down	1.907456	up
Os.13595.1.S1_at	-2.0047526	down	1.521604	up	-1.7026	down	-1.31753	down
Os.9015.1.S1_at	3.0841901	up	3.269404	up	-2.92186	down	10.08346	up
Os.51753.1.A1_at	1.966314	up	-2.00739	down	1.794043	up	-1.02089	down
OsAffx.6372.1.S1_s_a	-2.0388052	down	4.880853	up	-4.36312	down	2.393977	up
OsAffx.3504.1.S1_at	2.2007182	up	-2.68322	down	2.399245	up	-1.21925	down
Os.7969.2.S1_at	-1.9257846	down	3.432706	up	-3.83879	down	1.782497	up
Os.53237.1.S1_at	2.637788	up	2.523016	up	-2.25653	down	6.655181	up
Os.9073.1.S1_at	2.0385444	up	1.18781	up	-1.06271	down	2.421404	up
Os.12106.1.S1_at	-1.0615964	down	-2.4847	down	2.223676	up	-2.63775	down
Os.29404.2.S1_x_at	-2.2091906	down	1.441859	up	-1.61084	down	-1.53218	down
OsAffx.4611.1.S1_s_a	-1.7012141	down	2.183348	up	-1.95437	down	1.283406	up
Os.28433.2.S1_at	-1.2260932	down	-1.70506	down	1.526587	up	-2.09056	down
Os.51989.1.S1_at	-1.1288604	down	1.889605	up	-2.10959	down	1.673905	up
Os.50806.1.A1_at	-2.0311477	down	1.515923	up	-1.6923	down	-1.33988	down
Os.51171.1.S1_at	1.8083073	up	1.701306	up	-1.52429	down	3.076484	up
Os.27618.1.S1_at	-1.9413996	down	-1.03784	down	-1.07529	down	-2.01485	down
OsAffx.29571.1.S1_at	-1.4657269	down	2.125928	up	-1.90501	down	1.450426	up
Os.7794.1.S1_at	1.1647593	up	-2.56537	down	2.299222	up	-2.20249	down
Os.24954.1.A1_s_at	-3.4352021	down	1.084897	up	-1.20991	down	-3.16639	down
Os.52598.1.S1_at	-2.2821105	down	1.512579	up	-1.68681	down	-1.50875	down
Os.53217.1.S1_x_at	3.2654362	up	1.025666	up	1.086506	up	3.349248	up
OsAffx.22483.1.S1_at	-2.2854745	down	1.144343	up	-1.27476	down	-1.99719	down
Os.56369.1.S1_at	2.3023946	up	-1.4214	down	1.583073	up	1.619807	up
Os.14955.1.S1_at	-1.77516	down	-2.47244	down	2.752074	up	-4.38897	down
Os.52469.1.S1_at	1.3505428	up	2.428942	up	-2.18216	down	3.280391	up
Os.7074.1.S1_at	-2.0464778	down	1.085175	up	-1.20688	down	-1.88585	down
Os.51866.1.S1_at	3.9460132	up	-2.80529	down	3.11898	up	1.406632	up
Os.20863.2.S1_x_at	2.605809	up	-2.20024	down	1.97939	up	1.18433	up
Os.52764.1.S1_at	-1.929256	down	-1.50272	down	1.352135	up	-2.89914	down
Os.35391.1.S1_at	-2.4704173	down	1.614083	up	-1.45259	down	-1.53054	down
Os.46358.1.A1_at	-1.3599819	down	2.021143	up	-2.24539	down	1.486155	up
Os.9563.1.S1_at	1.6946609	up	1.30743	up	-1.17701	down	2.21565	up
Os.26648.1.S1_at	-1.9979134	down	-1.04868	down	1.164872	up	-2.09518	down
OsAffx.28166.1.S1_s_	-1.1291387	down	-2.44055	down	2.710289	up	-2.75572	down
Os.6517.1.S1_at	-1.4341731	down	2.231631	up	-2.0098	down	1.55604	up
Os.56238.1.S1_at	-1.4290739	down	-1.49967	down	1.664599	up	-2.14313	down
Os.12551.1.S1_s_at	4.3386254	up	-16.2564	down	18.0396	up	-3.7469	down
Os.49920.2.S1_at	-2.5017571	down	1.084213	up	1.02345	up	-2.30744	down
Os.12432.1.S1_a_at	-2.5017118	down	1.118646	up	-1.24123	down	-2.23638	down

Os.8081.1.A1_at	-2.5236673	down	1.088281	up	-1.20741	down	-2.31895	down
Os.51465.1.S1_at	1.593432	up	1.15527	up	-1.28121	down	1.840844	up
Os.17954.1.S1_at	1.3883404	up	1.573489	up	-1.74498	down	2.184538	up
Os.18412.1.S1_a_at	1.4706093	up	-1.80569	down	2.002416	up	-1.22785	down
Os.609.3.S1_a_at	1.5831642	up	1.36241	up	-1.22864	down	2.15692	up
Os.26933.2.S1_x_at	-1.0002614	down	2.666182	up	-2.40532	down	2.665485	up
Os.19815.1.S1_at	-3.2883053	down	2.25835	up	-2.50278	down	-1.45607	down
Os.26152.2.S1_x_at	1.3202323	up	1.676204	up	-1.51252	down	2.212979	up
Os.19616.1.S1_at	-2.1161466	down	1.096891	up	1.010281	up	-1.92922	down
Os.15671.1.A2_at	1.0350184	up	-2.68411	down	2.974423	up	-2.59329	down
Os.18671.1.S1_at	-2.070105	down	1.20747	up	-1.33784	down	-1.71442	down
Os.11640.1.S1_at	1.1362991	up	1.776362	up	-1.60347	down	2.018479	up
Os.46162.1.S1_at	-2.4933572	down	1.111253	up	-1.23085	down	-2.24373	down
Os.30989.1.S1_at	1.5730052	up	1.659653	up	-1.49867	down	2.610643	up
OsAffx.18899.1.S1_at	1.5228842	up	1.997787	up	-2.21221	down	3.042398	up
Os.18296.1.S1_x_at	-2.0687368	down	1.856539	up	-1.67659	down	-1.1143	down
Os.3568.1.S1_at	1.031091	up	-2.79095	down	3.086737	up	-2.70679	down
Os.49817.1.S1_at	-2.0085027	down	2.265272	up	-2.04832	down	1.127841	up
OsAffx.19804.1.S1_at	2.4353766	up	-1.24356	down	1.124709	up	1.958394	up
Os.27360.1.A1_at	-1.7193348	down	-1.19442	down	1.08051	up	-2.05361	down
Os.47980.1.S1_at	-2.9672906	down	1.665594	up	-1.84086	down	-1.78152	down
Os.18279.1.S1_at	2.110471	up	-1.21665	down	1.344445	up	1.734656	up
Os.14494.1.S1_at	-1.4907329	down	-1.25103	down	1.38234	up	-1.86495	down
OsAffx.20436.1.S1_x_	3.4796667	up	1.10941	up	-1.00408	down	3.860375	up
OsAffx.7132.1.S1_at	-1.5523567	down	1.931955	up	-2.13419	down	1.24453	up
Os.18196.1.S1_at	-4.032504	down	1.4719	up	-1.62594	down	-2.73966	down
Os.34549.1.S1_at	-1.9047831	down	2.038176	up	-2.25132	down	1.070031	up
OsAffx.19104.1.S1_at	2.254519	up	-1.07736	down	1.190003	up	2.092626	up
Os.14831.2.S1_at	-1.4495474	down	-1.58992	down	1.755624	up	-2.30466	down
OsAffx.31685.1.S1_at	-1.752399	down	-1.4616	down	1.324104	up	-2.5613	down
Os.37565.1.S1_x_at	-1.5532064	down	2.581768	up	-2.33997	down	1.662219	up
Os.10900.1.S1_at	-2.71547	down	1.428724	up	-1.57628	down	-1.90063	down
Os.17892.1.S1_at	-1.4637529	down	2.071446	up	-2.28533	down	1.415161	up
Os.21461.1.S1_at	-2.0222785	down	1.487074	up	-1.63995	down	-1.3599	down
Os.26079.1.S1_at	1.7787702	up	1.261798	up	-1.14442	down	2.244449	up
Os.10156.1.S1_a_at	1.4568622	up	1.730495	up	-1.56994	down	2.521093	up
Os.49840.1.S1_at	-3.504456	down	2.028517	up	-2.23568	down	-1.7276	down
Os.9836.1.S1_at	1.4797685	up	1.511271	up	-1.37168	down	2.236332	up
Os.4169.1.S1_at	1.2511587	up	-1.9836	down	2.185395	up	-1.58541	down
Os.11218.1.S1_at	-2.4002237	down	2.062154	up	-2.27047	down	-1.16394	down
Os.22882.1.S1_at	-2.2190468	down	1.330607	up	-1.46443	down	-1.6677	down
Os.53350.1.S1_at	-2.0535693	down	1.727812	up	-1.90132	down	-1.18854	down
OsAffx.18020.1.S1_at	2.0871577	up	-1.34626	down	1.223528	up	1.550339	up
Os.9450.1.S1_at	2.4472468	up	-1.71475	down	1.886371	up	1.427171	up
Os.12809.1.S1_s_at	-1.317507	down	2.043338	up	-1.85762	down	1.550913	up
Os.25496.1.S1_at	-1.2388637	down	-1.50252	down	1.652626	up	-1.86142	down
Os.17741.1.S1_at	1.164788	up	2.019913	up	-2.2217	down	2.35277	up

Os.26859.1.S1_at	-1.6421468	down	-1.28719	down	1.170863	up	-2.11376	down
Os.28642.1.S1_at	-1.0991251	down	-1.68818	down	1.855845	up	-1.85552	down
Os.26807.1.S1_at	-4.376969	down	3.297222	up	-2.99985	down	-1.32747	down
Os.7609.1.S1_at	1.5533785	up	1.920425	up	-1.7476	down	2.983147	up
Os.47936.1.A1_at	-2.1333387	down	1.20045	up	-1.31892	down	-1.77712	down
Os.31778.2.S1_at	8.393523	up	-1.48265	down	1.628843	up	5.661147	up
OsAffx.11075.1.S1_x_	1.2414285	up	1.771368	up	-1.94586	down	2.199027	up
OsAffx.25798.1.S1_at	3.0814612	up	-1.11448	down	1.222979	up	2.76494	up
Os.49721.1.S1_at	1.0069157	up	2.182213	up	-2.39408	down	2.197304	up
OsAffx.27698.1.S1_at	-1.2011513	down	2.06692	up	-1.88407	down	1.720782	up
Os.2242.1.S1_at	1.0137596	up	1.986756	up	-1.81135	down	2.014093	up
Os.11908.1.S1_s_at	2.7806168	up	1.735303	up	-1.58242	down	4.825213	up
OsAffx.12970.1.S1_s_	2.0101194	up	-1.15287	down	1.264223	up	1.743575	up
Os.33563.1.A1_at	-2.2090492	down	1.276016	up	-1.39902	down	-1.73121	down
OsAffx.17977.1.S1_at	2.3586876	up	-1.02735	down	1.126225	up	2.295894	up
Os.34815.1.S1_at	-2.2837617	down	1.295736	up	-1.42039	down	-1.76252	down
Os.5369.1.S1_at	-2.2310007	down	-1.01068	down	1.107886	up	-2.25482	down
OsAffx.19871.1.S1_at	1.5063308	up	2.436766	up	-2.22334	down	3.670576	up
Os.4700.1.S1_at	2.7244458	up	-2.45411	down	2.239394	up	1.110159	up
Os.16615.1.S1_at	-1.2241976	down	2.049807	up	-2.24624	down	1.674409	up
Os.53020.1.S1_at	1.5699638	up	1.619687	up	-1.47812	down	2.54285	up
Os.14423.1.S1_at	1.0470915	up	2.591342	up	-2.36548	down	2.713372	up
Os.33704.3.S1_x_at	-1.2037475	down	2.204282	up	-2.01218	down	1.831183	up
Os.10743.1.S1_at	-2.3926787	down	-1.04537	down	-1.04761	down	-2.50123	down
Os.8468.1.S1_s_at	-2.1466677	down	1.07534	up	-1.1776	down	-1.99627	down
Os.26802.1.S1_at	-1.6248866	down	1.961418	up	-2.14765	down	1.207111	up
Os.22783.1.S1_s_at	1.6546432	up	2.511867	up	-2.29624	down	4.156243	up
Os.49711.1.S1_at	-1.9480689	down	2.014661	up	-1.84173	down	1.034184	up
Os.14847.1.S1_at	-1.6591583	down	-1.30854	down	1.196282	up	-2.17107	down
Os.1678.1.S1_at	-1.1970688	down	2.01431	up	-1.84184	down	1.682702	up
Os.172.1.S1_a_at	-1.5205532	down	-3.25005	down	2.972577	up	-4.94188	down
Os.26857.1.S1_at	-1.6827266	down	-1.24714	down	1.140841	up	-2.09859	down
Os.27053.3.A1_s_at	-1.7227943	down	2.395475	up	-2.61856	down	1.390459	up
Os.15657.1.S1_at	-2.243281	down	1.333398	up	-1.45751	down	-1.68238	down
Os.10829.1.S1_at	-2.2579434	down	3.011	up	-2.75484	down	1.333514	up
Os.52496.1.S1_at	-1.9923023	down	2.534598	up	-2.76865	down	1.272195	up
Os.33081.1.S1_at	-2.1506963	down	-1.15936	down	1.266407	up	-2.49343	down
Os.27067.2.A1_at	1.0516524	up	2.162172	up	-1.97955	down	2.273853	up
Os.49855.1.S1_at	-2.7473845	down	2.097523	up	-2.29064	down	-1.30982	down
Os.57519.1.A1_at	2.2627864	up	-3.10176	down	3.386906	up	-1.37077	down
Os.8521.2.S1_x_at	1.1205426	up	2.178492	up	-1.99562	down	2.441093	up
Os.15841.1.S1_a_at	-1.3795867	down	3.108171	up	-2.84762	down	2.252972	up
Os.134.1.S1_at	2.2555478	up	-1.6009	down	1.747318	up	1.408929	up
Os.38282.1.S1_at	-1.6321306	down	-1.41854	down	1.299697	up	-2.31524	down
Os.10596.1.S1_at	2.7319136	up	4.389689	up	-4.02209	down	11.99225	up
OsAffx.3994.1.S1_at	-1.0350974	down	1.902286	up	-2.0761	down	1.837784	up
Os.50700.1.S1_at	1.7534157	up	1.046705	up	-1.14204	down	1.83531	up

Os.46021.1.S1_at	1.2199973	up	-3.86601	down	3.544201	up	-3.16887	down
Os.26742.1.S1_at	-1.8547722	down	-1.23918	down	1.351443	up	-2.29841	down
Os.11334.1.S1_x_at	2.2276547	up	-1.60202	down	1.746923	up	1.39053	up
OsAffx.15389.1.S1_at	-1.1928011	down	4.848291	up	-4.44873	down	4.064627	up
Os.2243.1.S1_a_at	1.1010278	up	2.244648	up	-2.06023	down	2.47142	up
OsAffx.11838.1.S1_at	-2.1334796	down	1.70898	up	-1.56871	down	-1.24839	down
Os.6672.1.S1_at	1.3367913	up	1.705826	up	-1.56599	down	2.280333	up
Os.33762.1.S1_at	-1.5085744	down	2.469796	up	-2.68839	down	1.637172	up
Os.8256.1.S1_at	-1.7569951	down	3.224112	up	-2.96477	down	1.835015	up
Os.57464.1.S1_x_at	1.681162	up	-2.21591	down	2.409473	up	-1.31808	down
Os.27688.1.A1_at	-2.063246	down	1.127966	up	-1.22642	down	-1.82917	down
Os.8786.3.S1_x_at	-1.4857816	down	1.986164	up	-2.15861	down	1.33678	up
Os.14243.1.S1_at	-2.208327	down	-1.04471	down	-1.04017	down	-2.30707	down
Os.5544.1.S1_at	-1.028739	down	1.891554	up	-2.05522	down	1.838711	up
Os.15784.1.A1_s_at	-1.1865538	down	2.159659	up	-2.34413	down	1.820111	up
OsAffx.30939.2.S1_s_	-1.4353957	down	2.120301	up	-1.95354	down	1.477154	up
Os.15288.1.S1_at	-2.3086896	down	1.306662	up	-1.41706	down	-1.76686	down
OsAffx.25290.1.S1_at	-1.3634593	down	-1.47864	down	1.60352	up	-2.01606	down
Os.8351.1.S1_at	-2.1427636	down	1.056217	up	-1.14538	down	-2.02872	down
Os.17076.1.S1_at	-1.4269037	down	36.35237	up	-39.4161	down	25.4764	up
Os.50750.1.S1_at	1.2674878	up	2.464034	up	-2.67148	down	3.123134	up
Os.54390.1.S1_at	-1.8970534	down	-1.16576	down	1.263634	up	-2.21152	down
Os.20548.1.S1_at	1.378773	up	1.863236	up	-1.71964	down	2.56898	up
Os.18000.1.A1_at	-1.9245468	down	-1.05953	down	-1.02244	down	-2.03912	down
Os.45919.1.S1_at	1.707968	up	1.214276	up	-1.31536	down	2.073944	up
Os.25101.2.A1_s_at	-1.8804127	down	-1.08554	down	1.002352	up	-2.04126	down
Os.24363.1.A1_at	1.1257559	up	12.10839	up	-13.1089	down	13.63109	up
OsAffx.16228.1.S1_at	-2.262932	down	-1.2488	down	1.153566	up	-2.82594	down
Os.49860.1.S1_at	2.161856	up	-1.29556	down	1.196812	up	1.668668	up
Os.2691.1.S1_a_at	-1.0738473	down	2.508913	up	-2.71528	down	2.336378	up
Os.5832.1.S1_at	-1.0415585	down	2.053728	up	-2.22185	down	1.971784	up
Os.12669.1.S1_at	-2.0575383	down	1.852112	up	-1.71251	down	-1.11091	down
Os.409.1.S1_at	1.6286156	up	-17.2507	down	15.95511	up	-10.5923	down
Os.11287.1.S1_at	1.0248426	up	-2.28134	down	2.466166	up	-2.22604	down
Os.18257.1.S1_at	-1.4848118	down	-2.48309	down	2.297091	up	-3.68693	down
Os.7559.1.S1_at	-2.6212409	down	2.069959	up	-1.91499	down	-1.26632	down
Os.52460.1.S1_at	1.0708078	up	3.375432	up	-3.12297	down	3.614439	up
Os.19370.1.S1_at	-2.444064	down	1.887635	up	-1.74718	down	-1.29478	down
Os.8643.1.S1_at	1.2255597	up	1.626044	up	-1.75621	down	1.992814	up
Os.15853.1.S1_at	-1.8621026	down	-1.18392	down	1.27863	up	-2.20458	down
OsAffx.30230.1.S1_at	1.8158125	up	1.271246	up	-1.37278	down	2.308343	up
Os.49489.1.S1_at	3.0152452	up	-1.56637	down	1.450877	up	1.924985	up
Os.22522.1.A1_a_at	-2.1288307	down	-1.11546	down	1.033337	up	-2.37462	down
Os.4636.1.S2_at	1.3778136	up	1.380359	up	-1.48995	down	1.901877	up
Os.15581.1.S1_at	-2.4296625	down	1.855102	up	-2.0017	down	-1.30972	down
Os.12725.1.S1_at	-1.233801	down	-1.82565	down	1.692072	up	-2.25249	down
Os.32637.2.S1_at	-3.250831	down	1.637658	up	-1.76683	down	-1.98505	down

Os.11808.1.S1_at	1.303629	up	-2.95855	down	2.742383	up	-2.26948	down
Os.49292.1.S1_at	-2.6652026	down	1.187857	up	-1.28122	down	-2.24371	down
Os.37571.1.S1_at	1.3288684	up	3.3206	up	-3.07879	down	4.41264	up
Os.54405.1.S1_at	-1.4062623	down	-1.40042	down	1.510037	up	-1.96935	down
Os.8811.1.S1_at	-2.053503	down	-1.13519	down	1.223617	up	-2.33111	down
Os.4666.1.S1_at	2.0797334	up	1.137417	up	-1.22573	down	2.365524	up
Os.52005.1.S1_at	1.8606881	up	-2.19657	down	2.366764	up	-1.18052	down
Os.18672.1.S1_s_at	1.1971726	up	1.727004	up	-1.60338	down	2.067522	up
OsAffx.27604.1.S1_x_	-1.0773622	down	1.983072	up	-2.13549	down	1.840674	up
Os.12809.1.S1_at	-1.5466243	down	2.319604	up	-2.49662	down	1.499785	up
Os.51901.1.S1_at	-1.704435	down	2.424438	up	-2.6078	down	1.422429	up
Os.7594.1.S1_at	1.3400606	up	2.252173	up	-2.09411	down	3.018049	up
Os.35576.1.S1_at	-1.3933554	down	2.052364	up	-2.207	down	1.472965	up
Os.54121.1.S2_s_at	-2.1205106	down	1.46395	up	-1.57388	down	-1.44849	down
Os.8045.1.S1_at	-3.1109333	down	3.079837	up	-2.86477	down	-1.0101	down
Os.17834.1.S1_at	-2.4434984	down	1.082393	up	-1.16332	down	-2.2575	down
Os.50120.1.S1_at	-2.101297	down	1.792983	up	-1.92646	down	-1.17196	down
Os.25117.1.A1_at	-2.9686348	down	-1.10291	down	1.026712	up	-3.27414	down
Os.33788.1.S1_at	-2.0115502	down	1.039982	up	-1.11706	down	-1.93422	down
Os.16015.1.S1_a_at	-2.098335	down	1.060518	up	-1.13874	down	-1.97859	down
Os.27942.1.A1_at	-1.8057382	down	-1.30603	down	1.401665	up	-2.35836	down
Os.51826.1.S1_at	-3.0978987	down	1.363577	up	-1.46299	down	-2.27189	down
Os.26908.1.A1_at	-2.2354357	down	1.436269	up	-1.33885	down	-1.55642	down
Os.7114.1.S1_s_at	1.8170183	up	1.425142	up	-1.32861	down	2.589509	up
Os.52129.1.S1_at	-2.3778782	down	1.359321	up	-1.26754	down	-1.74931	down
Os.23255.1.A1_at	-2.8802838	down	1.863535	up	-1.73808	down	-1.5456	down
Os.6362.1.S1_at	2.4730775	up	-1.6858	down	1.572499	up	1.467005	up
Os.7266.1.S1_at	-2.04565	down	1.111906	up	-1.1919	down	-1.83977	down
OsAffx.32067.1.S1_x_	1.7602206	up	1.332864	up	-1.42862	down	2.346135	up
Os.3105.1.S1_at	-2.100228	down	1.114769	up	-1.19471	down	-1.884	down
Os.50805.1.S1_at	2.6469386	up	1.618824	up	-1.51095	down	4.284927	up
Os.27232.1.S1_at	2.1704712	up	-2.56545	down	2.395549	up	-1.18198	down
Os.8044.1.S1_at	-2.9939396	down	1.441844	up	-1.54404	down	-2.07647	down
OsAffx.14519.1.S1_at	1.6281884	up	-2.44836	down	2.621709	up	-1.50373	down
Os.50791.1.S1_at	1.2725405	up	1.631953	up	-1.5241	down	2.076726	up
Os.1993.1.S1_at	1.4734279	up	1.368895	up	-1.2785	down	2.016968	up
OsAffx.30751.1.S1_at	2.5440738	up	-1.15575	down	1.237122	up	2.201228	up
Os.15171.1.S1_s_at	1.7307386	up	5.392402	up	-5.04182	down	9.332837	up
Os.20224.1.S1_at	2.1692524	up	-1.1512	down	1.230305	up	1.884342	up
Os.55181.1.S1_at	2.3651772	up	-1.1154	down	1.191805	up	2.120467	up
Os.14861.2.S1_at	-2.1454184	down	1.190094	up	-1.11419	down	-1.80273	down
Os.9626.1.S1_at	-1.33308	down	2.097942	up	-1.96445	down	1.573755	up
Os.11291.1.S1_at	-2.7702985	down	1.50715	up	-1.41142	down	-1.8381	down
Os.767.1.S1_at	-2.044318	down	1.526334	up	-1.62932	down	-1.33936	down
Os.17090.1.S1_at	2.073317	up	1.206766	up	-1.13076	down	2.502009	up
Os.12856.2.S1_at	-1.7882978	down	-1.1924	down	1.117375	up	-2.13237	down
Os.24753.1.S1_at	1.2399927	up	1.700764	up	-1.81405	down	2.108935	up

Os.5439.1.S1_a_at	1.0202507	up	2.125939	up	-1.99358	down	2.16899	up
Os.34012.1.S1_at	-2.0535102	down	1.393811	up	-1.48579	down	-1.47331	down
Os.9184.1.S1_at	-1.746862	down	-1.26481	down	1.186845	up	-2.20944	down
Os.2558.1.S1_a_at	1.168799	up	2.047808	up	-1.92167	down	2.393476	up
Os.9790.2.S1_x_at	1.3778366	up	2.424203	up	-2.27645	down	3.340156	up
Os.12488.1.S1_at	1.511552	up	1.628304	up	-1.73358	down	2.461266	up
OsAffx.16332.1.S1_x_	1.3718207	up	-2.51842	down	2.680547	up	-1.83582	down
Os.8251.1.S1_at	-3.2684836	down	1.329942	up	-1.24965	down	-2.45761	down
OsAffx.5108.1.S1_s_a	1.3961498	up	-2.0299	down	2.159793	up	-1.45392	down
OsAffx.27480.1.S1_at	1.4706482	up	1.451329	up	-1.54402	down	2.134395	up
Os.29003.1.S1_x_at	-2.0314806	down	1.598287	up	-1.50257	down	-1.27104	down
Os.50550.1.S1_at	-2.2226396	down	1.109566	up	-1.04312	down	-2.00316	down
Os.5881.1.S1_at	-1.0422763	down	1.885656	up	-2.00527	down	1.809171	up
Os.12228.1.S1_at	1.1650816	up	-2.02911	down	1.908948	up	-1.7416	down
Os.28124.1.S1_at	-1.263513	down	-1.82639	down	1.719129	up	-2.30767	down
Os.46823.1.S1_at	-3.024282	down	2.236425	up	-2.37591	down	-1.35228	down
Os.15713.1.S1_a_at	-1.886644	down	2.035912	up	-2.16159	down	1.079118	up
OsAffx.30676.2.S1_x_	1.1791502	up	-2.93314	down	3.114171	up	-2.4875	down
Os.27955.2.S1_x_at	2.346804	up	1.001175	up	1.060239	up	2.349561	up
Os.26411.2.A1_x_at	4.851004	up	-1.10104	down	1.168617	up	4.405845	up
Os.32748.1.S1_at	2.1515281	up	-1.43439	down	1.522088	up	1.49996	up
Os.53016.1.S1_at	-2.165357	down	-1.02088	down	-1.03925	down	-2.21056	down
Os.26384.1.S1_at	-1.3467361	down	2.341916	up	-2.48301	down	1.738957	up
Os.14529.1.S1_x_at	1.2473264	up	1.820048	up	-1.71736	down	2.270194	up
Os.10162.1.S1_at	-2.2509284	down	2.306609	up	-2.44408	down	1.024737	up
Os.5616.1.S1_at	-1.500893	down	2.207905	up	-2.33914	down	1.471061	up
Os.37067.1.S1_at	-1.2961215	down	3.726831	up	-3.51775	down	2.875372	up
Os.54527.1.S1_at	-2.0189333	down	1.256994	up	-1.33163	down	-1.60616	down
Os.12732.2.S1_x_at	-2.136957	down	1.321401	up	-1.39983	down	-1.61719	down
Os.10178.1.S1_a_at	-1.162546	down	3.280304	up	-3.09789	down	2.821655	up
Os.42305.1.S1_s_at	1.7037897	up	-2.14517	down	2.27126	up	-1.25906	down
Os.55224.1.S1_at	-1.3253766	down	-3.60794	down	3.819429	up	-4.78188	down
Os.57325.1.S1_at	-1.6623889	down	-1.22828	down	1.160311	up	-2.04189	down
Os.20313.1.S1_x_at	1.5152099	up	1.270911	up	-1.34523	down	1.925697	up
Os.16863.1.S1_at	-2.1154983	down	1.325723	up	-1.40282	down	-1.59573	down
Os.37630.1.S1_x_at	-1.1838999	down	-1.63361	down	1.728275	up	-1.93403	down
Os.52009.1.S1_at	-2.3293128	down	1.448451	up	-1.36918	down	-1.60814	down
Os.7017.1.S1_at	-2.2389839	down	2.844185	up	-3.00765	down	1.270302	up
Os.14870.1.S1_at	1.2896972	up	1.653789	up	-1.74865	down	2.132887	up
Os.46617.1.S1_at	-1.2055335	down	-2.0039	down	1.896042	up	-2.41577	down
Os.10816.1.S1_at	-2.614121	down	1.50759	up	-1.4269	down	-1.73397	down
Os.50721.1.S1_at	1.4346286	up	-2.19275	down	2.316078	up	-1.52844	down
Os.26784.1.S1_a_at	-1.6798553	down	-1.60064	down	1.51676	up	-2.68884	down
Os.17075.1.S1_at	-2.4490182	down	1.720219	up	-1.81373	down	-1.42367	down
Os.23112.1.A1_at	-2.1841695	down	1.367002	up	-1.29661	down	-1.59778	down
Os.6089.1.S1_at	2.2170472	up	1.891381	up	-1.99227	down	4.193279	up
Os.19325.1.S1_at	-2.0442803	down	1.155523	up	-1.21677	down	-1.76914	down

Os.52717.1.S1_at	-2.8405614	down	3.119262	up	-3.28401	down	1.098115	up
Os.7756.2.S1_x_at	-2.5713248	down	5.496622	up	-5.78649	down	2.137662	up
Os.17783.1.S1_at	-2.8193698	down	1.488247	up	-1.56658	down	-1.89442	down
Os.12025.1.S1_a_at	-1.1769586	down	2.163228	up	-2.27691	down	1.837981	up
Os.27241.1.S1_at	-2.503258	down	1.659498	up	-1.74654	down	-1.50844	down
OsAffx.19033.1.S1_at	-2.1634665	down	1.158592	up	-1.21912	down	-1.86732	down
Os.38278.1.S1_at	-2.2220135	down	-1.55122	down	1.475453	up	-3.44682	down
Os.24073.1.S1_at	-2.0963428	down	1.142659	up	-1.20127	down	-1.83462	down
Os.37915.1.S1_at	2.0165856	up	-1.52671	down	1.604984	up	1.320871	up
Os.2448.1.S2_at	1.5106356	up	1.583659	up	-1.50695	down	2.392332	up
Os.23290.1.S1_at	1.3941542	up	2.117208	up	-2.01603	down	2.951714	up
Os.24051.1.S1_x_at	1.7418922	up	1.609944	up	-1.5332	down	2.804349	up
Os.15426.1.S1_at	-1.7922244	down	2.302438	up	-2.19271	down	1.284681	up
Os.16401.1.S2_at	-1.4552974	down	2.029129	up	-1.93357	down	1.394305	up
Os.57569.2.A1_s_at	-1.053234	down	17.99945	up	-18.8873	down	17.0897	up
Os.23660.1.S1_at	-1.2048632	down	-2.51952	down	2.401126	up	-3.03568	down
Os.53355.1.S1_s_at	-3.1635988	down	2.80033	up	-2.66962	down	-1.12972	down
Os.16051.1.S1_at	2.031725	up	-1.89775	down	1.810113	up	1.070595	up
Os.5179.1.A1_at	-2.0346866	down	1.707502	up	-1.62881	down	-1.19162	down
Os.22451.1.A1_at	-1.4067017	down	-1.43099	down	1.365057	up	-2.01298	down
Os.24698.2.S1_x_at	1.0887765	up	-2.75232	down	2.625588	up	-2.5279	down
Os.7826.2.A1_at	-2.0710175	down	1.135561	up	-1.18985	down	-1.82378	down
Os.38017.1.S1_s_at	2.4443076	up	2.418165	up	-2.53369	down	5.910738	up
Os.11388.1.S1_at	-2.1837635	down	1.887341	up	-1.80153	down	-1.15706	down
Os.52657.1.S1_at	-2.5730162	down	1.189371	up	-1.24561	down	-2.16334	down
Os.18172.1.S1_at	-1.7381586	down	-1.27425	down	1.216867	up	-2.21485	down
Os.49571.1.S1_at	-1.288914	down	1.93896	up	-2.0294	down	1.504336	up
Os.57472.1.S1_x_at	1.5734198	up	1.482379	up	-1.41637	down	2.332404	up
OsAffx.26358.1.S1_at	-2.082137	down	-1.43727	down	1.373426	up	-2.9926	down
Os.49677.1.S1_at	2.14248	up	-1.37505	down	1.314202	up	1.558112	up
Os.46831.1.S1_at	-2.3162963	down	1.465924	up	-1.40182	down	-1.58009	down
Os.28110.1.S1_x_at	1.0918866	up	2.104037	up	-2.01223	down	2.29737	up
Os.28514.1.S1_at	-2.5924158	down	2.72222	up	-2.84421	down	1.050071	up
Os.18989.1.S1_at	2.109389	up	-1.16601	down	1.218216	up	1.809065	up
Os.12999.1.S1_at	2.452387	up	-1.16221	down	1.112422	up	2.110114	up
Os.53160.1.S1_at	-2.701265	down	1.483685	up	-1.5488	down	-1.82065	down
Os.38339.1.S1_at	1.6070168	up	-2.24069	down	2.338338	up	-1.39432	down
Os.7916.1.S1_at	1.0869992	up	1.946106	up	-1.86521	down	2.115416	up
Os.48215.1.A1_at	1.7945703	up	1.158255	up	-1.20846	down	2.07857	up
Os.10051.1.S1_at	1.1892515	up	-2.52625	down	2.421532	up	-2.12423	down
OsAffx.32302.1.A1_at	-2.375423	down	1.913827	up	-1.99641	down	-1.24119	down
OsAffx.32325.1.S1_at	-3.4109795	down	4.598758	up	-4.79717	down	1.348222	up
Os.5549.1.S1_at	-2.165257	down	4.457559	up	-4.64928	down	2.058674	up
Os.10083.1.S1_at	1.7120872	up	1.545478	up	-1.48191	down	2.645993	up
Os.14854.1.S1_at	-1.8856248	down	2.403265	up	-2.30566	down	1.274519	up
Os.57422.1.S1_x_at	1.8845105	up	1.36706	up	-1.31186	down	2.576238	up
Os.820.1.S1_at	1.8017339	up	1.446246	up	-1.50708	down	2.605751	up

Os.20060.1.S1_at	-1.763595	down	3.397683	up	-3.2611	down	1.926567	up
Os.48076.1.S1_at	-1.0938448	down	-2.35133	down	2.257044	up	-2.57199	down
Os.4974.1.S1_at	-2.3694055	down	3.089984	up	-3.21902	down	1.304118	up
Os.12329.1.S1_at	2.1122577	up	-1.22336	down	1.273984	up	1.726604	up
Os.49856.1.S1_at	-2.575086	down	1.493937	up	-1.4348	down	-1.72369	down
Os.36647.1.S1_at	-4.3968835	down	1.259984	up	-1.31186	down	-3.48963	down
Os.26491.1.S1_at	-2.0695562	down	1.656037	up	-1.72382	down	-1.2497	down
Os.18070.1.S1_at	-1.2177507	down	5.366196	up	-5.16105	down	4.406646	up
OsAffx.14027.1.S1_at	3.2335684	up	-1.70392	down	1.639866	up	1.897726	up
Os.8752.1.S1_at	-1.6712213	down	-1.17253	down	1.218112	up	-1.95955	down
Os.48784.1.S1_at	1.5706736	up	4.483653	up	-4.31879	down	7.042355	up
Os.47730.1.S1_x_at	2.8184164	up	1.24136	up	-1.1961	down	3.498668	up
Os.5030.1.S1_at	1.6645217	up	1.243785	up	-1.19889	down	2.070307	up
Os.6093.3.S1_at	-2.2174265	down	1.551628	up	-1.60965	down	-1.4291	down
Os.24664.1.A1_at	-2.1311092	down	-1.18489	down	1.142285	up	-2.52512	down
Os.49153.1.A1_x_at	-2.3601146	down	1.210677	up	-1.25578	down	-1.94942	down
Os.8521.1.S1_a_at	1.109433	up	1.807302	up	-1.87458	down	2.005081	up
OsAffx.7025.1.S1_at	2.3734531	up	-1.40251	down	1.454642	up	1.692288	up
OsAffx.21798.1.S1_at	2.1333594	up	-2.55318	down	2.462082	up	-1.19679	down
Os.28034.1.S1_at	-2.2367902	down	-1.18723	down	1.145204	up	-2.65559	down
Os.11565.1.S1_at	-1.2220428	down	2.248579	up	-2.16907	down	1.840016	up
Os.46631.1.S1_x_at	1.1196473	up	2.889061	up	-2.7878	down	3.234729	up
Os.28409.20.A1_a_at	-1.3452822	down	-1.66168	down	1.604361	up	-2.23544	down
Os.22484.1.A1_at	-1.2035137	down	-1.79021	down	1.728584	up	-2.15455	down
OsAffx.18131.3.S1_at	-1.4769436	down	-1.31412	down	1.360738	up	-1.94088	down
Os.12402.2.S1_x_at	-2.0432642	down	1.410002	up	-1.45885	down	-1.44912	down
OsAffx.18426.1.S1_x_	-1.0089732	down	2.731842	up	-2.82628	down	2.707547	up
Os.44710.1.S1_at	-2.5515068	down	1.275153	up	-1.31918	down	-2.00094	down
Os.54186.1.S1_at	-2.4059856	down	1.424144	up	-1.37732	down	-1.68943	down
OsAffx.3569.1.S1_x_a	3.0173063	up	-2.49403	down	2.413866	up	1.209813	up
Os.16957.1.S1_at	-2.3724594	down	1.261093	up	-1.30152	down	-1.88127	down
Os.12430.1.S1_at	4.053709	up	-30.681	down	29.7293	up	-7.56863	down
OsAffx.10929.1.S1_x_	1.2969444	up	-2.17729	down	2.11039	up	-1.67879	down
OsAffx.6932.1.S1_at	-2.1238244	down	1.556351	up	-1.50895	down	-1.36462	down
Os.50964.1.S1_at	-2.5489972	down	-1.23105	down	1.193657	up	-3.13795	down
Os.27080.1.S1_at	-2.107956	down	1.560334	up	-1.60893	down	-1.35097	down
Os.44598.1.S1_x_at	2.30711	up	1.530534	up	-1.57812	down	3.53111	up
Os.3808.4.S1_x_at	-1.1694636	down	2.238801	up	-2.17192	down	1.914383	up
Os.6171.1.S1_at	-1.4136358	down	-1.44464	down	1.402646	up	-2.0422	down
Os.11995.1.S1_at	2.3834822	up	-2.6476	down	2.571054	up	-1.11081	down
OsAffx.30948.1.S1_at	-2.0251534	down	1.137075	up	-1.17078	down	-1.78102	down
Os.13051.1.S1_at	-2.4838893	down	1.055689	up	-1.02537	down	-2.35286	down
OsAffx.13380.1.S1_at	1.6420842	up	-2.25953	down	2.325492	up	-1.37601	down
Os.28578.1.S1_at	1.2916924	up	-2.64521	down	2.571544	up	-2.04786	down
OsAffx.5509.1.S1_at	-2.0286105	down	1.384682	up	-1.42435	down	-1.46504	down
Os.338.1.S1_x_at	1.2419624	up	1.617581	up	-1.66359	down	2.008974	up
Os.52292.1.S1_at	-2.7938566	down	-1.333	down	1.296312	up	-3.7242	down

Os.27421.1.S1_at	-2.0748904	down	1.468429	up	-1.50967	down	-1.413	down
Os.37700.1.S1_at	-2.1051745	down	1.363066	up	-1.32607	down	-1.54444	down
Os.19361.1.S2_at	1.4909477	up	1.890974	up	-1.83969	down	2.819343	up
Os.9618.1.S1_at	-2.7989142	down	1.217252	up	-1.25089	down	-2.29937	down
Os.12629.1.S1_at	1.8774122	up	-10.6323	down	10.9217	up	-5.66327	down
OsAffx.9503.1.S1_at	1.6803602	up	1.426101	up	-1.38854	down	2.396364	up
Os.47694.1.S1_at	-2.947628	down	1.260045	up	-1.29409	down	-2.3393	down
Os.11561.2.S1_a_at	2.013164	up	1.482811	up	-1.44401	down	2.985142	up
Os.43124.1.S1_at	2.2304487	up	-1.3753	down	1.411894	up	1.621794	up
Os.33183.1.S1_at	-1.5835797	down	-1.50857	down	1.548325	up	-2.38894	down
Os.55250.1.S1_at	-3.568935	down	3.81814	up	-3.72436	down	1.069826	up
Os.7276.1.A1_s_at	-2.1376636	down	1.014788	up	-1.0403	down	-2.10651	down
Os.12082.1.S1_at	-3.1013358	down	2.405877	up	-2.46608	down	-1.28907	down
Os.10534.1.S1_at	2.1671858	up	1.275658	up	-1.30744	down	2.764589	up
Os.1054.1.A1_at	1.2686719	up	1.951701	up	-1.9043	down	2.476068	up
Os.55624.1.S1_at	-1.4360938	down	2.888971	up	-2.81913	down	2.011687	up
Os.56058.1.S1_at	-2.0560997	down	-1.15902	down	1.131466	up	-2.38305	down
Os.53275.1.S1_at	2.1507404	up	-1.1257	down	1.153054	up	1.910574	up
Os.48704.1.S1_at	-2.0948188	down	1.352841	up	-1.38556	down	-1.54846	down
Os.25324.1.A1_at	-2.7232265	down	1.533226	up	-1.49732	down	-1.77614	down
Os.26406.2.S1_x_at	4.075366	up	-1.1156	down	1.141762	up	3.653061	up
OsAffx.27346.1.S1_at	3.292466	up	-1.28007	down	1.309815	up	2.572099	up
Os.9321.1.S1_a_at	-5.1791506	down	-1.22917	down	1.201443	up	-6.36605	down
OsAffx.28078.1.S1_x_	1.1761234	up	1.856424	up	-1.89848	down	2.183384	up
Os.29084.1.S1_at	5.086082	up	-2.76732	down	2.707605	up	1.837908	up
Os.15252.1.S1_a_at	-1.2639586	down	-1.7262	down	1.689325	up	-2.18185	down
Os.50564.2.S1_at	-2.103241	down	1.262647	up	-1.29002	down	-1.66574	down
Os.8567.1.S1_at	-1.0564575	down	2.484749	up	-2.53858	down	2.351963	up
Os.31778.2.S1_x_at	12.39472	up	-1.56343	down	1.596587	up	7.927891	up
Os.46726.1.S1_x_at	-1.3355821	down	-1.66872	down	1.634548	up	-2.22872	down
Os.27867.1.S1_at	-2.1299222	down	1.358381	up	-1.38631	down	-1.56799	down
Os.55587.1.S1_at	-2.1518703	down	1.159081	up	-1.18279	down	-1.85653	down
Os.50539.1.S1_at	-2.0497582	down	1.127503	up	-1.14993	down	-1.81796	down
Os.39073.1.S1_at	-2.1310558	down	1.106732	up	-1.12817	down	-1.92554	down
Os.17797.1.A1_at	-1.6843852	down	2.366476	up	-2.32273	down	1.404949	up
Os.11967.1.S1_at	1.742897	up	2.082307	up	-2.0441	down	3.629247	up
Os.40003.1.S1_at	-1.2326462	down	-2.25142	down	2.293229	up	-2.7752	down
Os.2128.1.S1_at	1.1431694	up	2.050102	up	-2.08795	down	2.343614	up
OsAffx.1987.1.S1_at	-1.1452019	down	2.240612	up	-2.20003	down	1.956521	up
Os.11445.1.S1_at	-1.5963902	down	2.139573	up	-2.10101	down	1.340257	up
Os.49557.1.S1_at	1.7516985	up	1.406801	up	-1.43212	down	2.464291	up
Os.19172.1.S1_at	-1.5018803	down	-1.50345	down	1.529999	up	-2.258	down
Os.7687.1.S1_at	1.0292077	up	1.974065	up	-2.00809	down	2.031723	up
Os.54045.1.S1_at	-2.1008108	down	1.467895	up	-1.44306	down	-1.43117	down
Os.7539.1.S1_at	1.6847088	up	2.243555	up	-2.2057	down	3.779737	up
Os.31778.1.S1_x_at	11.763217	up	-1.51155	down	1.486058	up	7.782244	up
OsAffx.24981.1.S1_at	-1.0778296	down	4.12808	up	-4.19829	down	3.829993	up

Os.49388.1.S1_at	-2.0008821	down	1.269144	up	-1.29044	down	-1.57656	down
Os.52183.1.S1_at	-2.4589953	down	-1.2285	down	1.208699	up	-3.02087	down
Os.18597.1.S1_at	-1.6116725	down	3.014895	up	-2.96862	down	1.870662	up
Os.12196.1.S1_at	-1.0107826	down	2.094695	up	-2.06334	down	2.07235	up
Os.52588.1.S1_at	1.0054286	up	2.643216	up	-2.60394	down	2.657565	up
OsAffx.23247.1.S1_x_	3.3008573	up	1.889413	up	-1.8616	down	6.236683	up
Os.27624.1.S1_at	2.9767528	up	-1.19498	down	1.177852	up	2.491047	up
Os.50498.1.S1_at	-2.2292945	down	1.986726	up	-1.95833	down	-1.12209	down
Os.19755.2.S1_a_at	-1.2749312	down	-2.0165	down	2.045335	up	-2.5709	down
Os.44937.1.S1_at	1.1842057	up	2.054349	up	-2.08309	down	2.432771	up
Os.32454.1.S1_at	1.3160932	up	1.614649	up	-1.63699	down	2.125029	up
Os.18299.1.S1_at	2.3931859	up	-1.94966	down	1.924442	up	1.227488	up
Os.6173.2.S1_x_at	1.3399264	up	1.536395	up	-1.51682	down	2.058657	up
OsAffx.3569.1.S1_at	3.1503465	up	-2.51571	down	2.545806	up	1.252269	up
Os.9993.1.S1_at	-2.3157513	down	1.509827	up	-1.52736	down	-1.53379	down
Os.47358.1.A1_at	-2.0131176	down	1.047571	up	-1.05966	down	-1.9217	down
OsAffx.30204.1.S1_at	2.0348074	up	-1.05199	down	1.064101	up	1.934251	up
Os.26820.1.A1_s_at	1.1393273	up	7.651951	up	-7.56573	down	8.718077	up
Os.11808.2.S1_x_at	1.370244	up	-2.18303	down	2.207414	up	-1.59317	down
Os.9655.1.S1_at	-2.32191	down	1.349652	up	-1.36425	down	-1.72038	down
OsAffx.25830.1.S1_at	1.8376102	up	1.288398	up	-1.30083	down	2.367574	up
Os.53270.1.S1_at	1.4080733	up	1.438819	up	-1.4251	down	2.025963	up
Os.53236.1.S1_at	-1.2400749	down	2.06561	up	-2.04722	down	1.665714	up
Os.2957.1.S1_at	1.5815881	up	1.824326	up	-1.84045	down	2.885332	up
Os.15738.1.S1_at	-1.3785626	down	2.391997	up	-2.37239	down	1.735139	up
Os.47878.1.S1_at	-2.009814	down	1.098418	up	-1.10696	down	-1.82974	down
OsAffx.12602.1.S1_at	1.7374451	up	1.232568	up	-1.24211	down	2.141519	up
Os.4627.1.S1_x_at	2.0260942	up	-1.23727	down	1.227856	up	1.637555	up
Os.7611.1.S1_at	-1.0155888	down	2.193244	up	-2.20998	down	2.159579	up
Os.4619.1.S1_at	1.0348815	up	2.177562	up	-2.16151	down	2.253518	up
Os.18700.1.S1_at	1.2540922	up	2.153526	up	-2.13832	down	2.70072	up
Os.53581.1.S1_at	1.1887764	up	1.964938	up	-1.97836	down	2.335872	up
Os.37668.1.S1_at	-1.199158	down	2.275855	up	-2.29047	down	1.897878	up
Os.24797.1.A1_at	-1.905073	down	-1.13494	down	1.142057	up	-2.16215	down
Os.5236.1.S1_at	-2.7454114	down	1.098084	up	-1.09143	down	-2.50018	down
Os.28397.1.S2_x_at	1.514127	up	1.711421	up	-1.70105	down	2.591308	up
Os.37617.1.S1_at	1.0377501	up	-2.92539	down	2.908399	up	-2.81897	down
Os.13580.1.A1_at	-2.2181983	down	1.298225	up	-1.30502	down	-1.70864	down
Os.4159.1.S1_at	2.0162683	up	1.226102	up	-1.23231	down	2.472151	up
Os.57191.1.S1_at	3.7100246	up	-1.33647	down	1.3428	up	2.77598	up
Os.2106.1.S2_at	-2.1799757	down	1.115882	up	-1.12116	down	-1.95359	down
Os.53413.1.S1_at	2.5598102	up	1.130176	up	-1.13496	down	2.893036	up
Os.18101.1.S1_at	-2.079774	down	1.135457	up	-1.13105	down	-1.83166	down
OsAffx.27304.1.S1_x_	1.1298397	up	1.918199	up	-1.91128	down	2.167257	up
Os.7169.1.S1_at	-2.3489256	down	1.445586	up	-1.4409	down	-1.62489	down
OsAffx.3309.1.S1_s_a	-2.079329	down	1.709188	up	-1.70397	down	-1.21656	down
Os.17140.1.S1_x_at	-2.3383076	down	1.092067	up	-1.08882	down	-2.14118	down

OsAffx.2900.1.S1_at	1.0325781	up	2.61807	up	-2.61039	down	2.703362	up
Os.23397.1.S1_at	2.4370615	up	-1.64351	down	1.638884	up	1.48284	up
Os.26697.1.S1_at	-2.0470083	down	1.197342	up	-1.19408	down	-1.70963	down
Os.8427.1.S1_s_at	1.1090326	up	-2.43594	down	2.429641	up	-2.19646	down
Os.48986.1.S1_s_at	3.2742083	up	-1.48186	down	1.485694	up	2.209526	up
Os.10564.1.A1_at	-2.23901	down	-1.56845	down	1.564915	up	-3.51178	down
OsAffx.7844.1.S1_at	2.396828	up	-1.51713	down	1.513979	up	1.579846	up
Os.8247.1.S1_at	-1.3336245	down	2.289162	up	-2.28495	down	1.716496	up
Os.5094.1.S2_at	-2.037387	down	-1.29222	down	1.289859	up	-2.63276	down
Os.22598.1.S1_at	-2.0180306	down	1.205452	up	-1.20656	down	-1.67409	down
Os.29815.1.S1_at	-1.2117642	down	-3.06007	down	3.059443	up	-3.70808	down
Os.27684.1.S2_at	-2.51911	down	-1.59558	down	1.595323	up	-4.01945	down
OsAffx.7566.1.S1_at	1.619159	up	1.79024	up	-1.78998	down	2.898682	up
Os.18692.1.S1_at	1.0767335	up	2.482054	up	-2.48218	down	2.67251	up
Os.11621.1.S1_at	-2.2932854	down	1.193162	up	-1.19314	down	-1.92202	down

Fold chang	Regulation	Fold chang	Regulation([NS] vs [NC])
-5.23434	down	213.2153	up
-1.15677	down	178.1498	up
-1.53312	down	150.8365	up
-1.05888	down	110.993	up
-1.22954	down	109.9126	up
-5.85016	down	104.0725	up
-1.01298	down	88.66203	up
-1.54755	down	78.1666	up
1.228561	up	72.21126	up
-1.00485	down	68.29211	up
-1.03411	down	66.21467	up
1.071421	up	63.78766	up
-7.45724	down	58.76641	up
-1.34753	down	56.73556	up
1.31961	up	55.24628	up
-5.04169	down	53.86204	up
-1.28091	down	51.52585	up
-11.1466	down	50.48859	up
-1.65883	down	43.00162	up
1.13579	up	39.53883	up
1.084382	up	35.02264	up
-1.93157	down	34.87114	up
-1.00507	down	33.80091	up
-1.88601	down	31.07864	up
-3.18439	down	29.95926	up
2.120414	up	29.67338	up
3.230177	up	29.54477	up
-1.16733	down	27.99593	up
1.30658	up	27.54217	up
1.509468	up	27.38031	up
1.321738	up	27.3253	up
-1.23417	down	24.7733	up
-1.10944	down	24.64673	up
-6.51968	down	24.51383	up
1.080583	up	23.93191	up
-1.89177	down	23.19043	up
4.05798	up	20.56941	up
-1.3183	down	20.25264	up
-1.20371	down	19.95717	up
-1.04908	down	19.61507	up
-4.07167	down	19.52107	up
-4.29357	down	19.4482	up
-1.08419	down	19.41645	up
1.111139	up	18.85188	up
1.098268	up	17.62181	up
1.22433	up	17.38638	up

-2.37114	down	16.66811	up
-15.2614	down	16.53207	up
-2.37086	down	16.47019	up
1.670346	up	16.44705	up
1.046308	up	16.44533	up
-1.92172	down	16.33598	up
-1.17007	down	16.27857	up
-3.90177	down	15.95372	up
1.013659	up	15.92211	up
1.429164	up	15.85286	up
1.239937	up	15.78588	up
1.719233	up	15.5438	up
1.185098	up	15.50285	up
-1.34367	down	14.8546	up
1.521038	up	14.77548	up
-13.8351	down	14.72605	up
2.021963	up	14.58732	up
1.075367	up	14.46833	up
-2.01951	down	14.35514	up
-12.7315	down	14.3534	up
-1.11314	down	14.22067	up
1.363336	up	14.09522	up
-6.69733	down	14.09396	up
-3.0972	down	14.03069	up
-2.94577	down	13.94349	up
2.513685	up	13.86772	up
1.74097	up	-13.5947	down
2.032111	up	-13.3348	down
1.803814	up	-13.1702	down
2.239733	up	13.13059	up
-1.35329	down	13.04784	up
1.748217	up	12.6405	up
-1.07553	down	12.59235	up
2.290154	up	12.49565	up
1.187192	up	12.35484	up
1.157098	up	12.2352	up
1.783154	up	12.22992	up
-1.81106	down	12.03613	up
1.002124	up	12.01779	up
-6.29105	down	11.53818	up
2.563141	up	11.51285	up
-10.8385	down	11.45717	up
1.539737	up	11.43394	up
3.0054	up	11.42909	up
-8.20485	down	11.40179	up
1.582724	up	11.35543	up
-1.0865	down	11.23971	up

1.557355	up	-11.1061	down
-5.38861	down	11.10386	up
1.418621	up	10.99745	up
1.355251	up	-10.9515	down
1.813657	up	10.94081	up
-1.14583	down	10.86946	up
1.87583	up	-10.8577	down
3.287007	up	10.83256	up
1.899571	up	10.69909	up
1.486618	up	10.68465	up
-1.28224	down	-10.6509	down
-4.46932	down	10.60317	up
-1.19494	down	10.57732	up
-1.04198	down	10.51209	up
1.69615	up	10.49213	up
1.186596	up	10.48692	up
1.315809	up	10.43528	up
1.224222	up	10.43265	up
1.333495	up	10.39486	up
-1.27586	down	-10.3335	down
2.586464	up	10.3166	up
-1.52396	down	-10.2786	down
-6.00988	down	10.24475	up
-1.50825	down	10.03589	up
1.073338	up	9.985916	up
2.169724	up	9.930164	up
2.712303	up	9.918018	up
-5.47522	down	9.886033	up
-5.17253	down	9.813136	up
-1.43411	down	9.742474	up
-1.08566	down	9.699549	up
-1.46916	down	9.667279	up
2.885604	up	9.442935	up
1.042855	up	9.354903	up
-1.01198	down	9.346012	up
6.193665	up	9.287697	up
1.069394	up	-9.2361	down
1.227079	up	9.21802	up
2.282026	up	9.206233	up
1.040349	up	9.200695	up
-1.11415	down	9.192496	up
-1.32723	down	9.189445	up
1.932448	up	9.184818	up
-1.41576	down	9.169104	up
-2.06445	down	9.159843	up
1.036304	up	9.146583	up
-2.31711	down	8.932572	up

1.233283	up	8.891233	up
1.216095	up	8.879039	up
1.64617	up	8.82924	up
-1.63594	down	8.826179	up
-1.77039	down	8.815715	up
3.090279	up	8.731094	up
1.080948	up	8.720997	up
-1.94521	down	8.655941	up
1.300589	up	8.620357	up
1.858174	up	8.614039	up
1.02148	up	-8.60842	down
-1.96336	down	8.600896	up
-2.90467	down	8.596526	up
1.169707	up	-8.56267	down
-1.05284	down	8.504226	up
-3.72695	down	8.461439	up
-1.9178	down	8.451253	up
-1.07494	down	-8.35742	down
1.179884	up	8.353303	up
-2.57102	down	8.328718	up
1.256993	up	-8.3222	down
-1.32796	down	8.222929	up
-1.00056	down	-8.2028	down
-1.47672	down	8.202785	up
2.212291	up	8.184938	up
3.469271	up	8.175314	up
-1.51404	down	8.107435	up
-1.15743	down	8.050063	up
-1.0197	down	8.049288	up
2.84131	up	8.035428	up
2.311861	up	8.011251	up
1.674889	up	7.940643	up
2.010185	up	7.911404	up
-2.28273	down	7.906654	up
-2.93161	down	7.874654	up
1.44001	up	7.852986	up
-1.94665	down	7.812841	up
2.315638	up	7.807695	up
1.566738	up	7.803541	up
3.102031	up	7.777905	up
-1.15992	down	7.755308	up
-1.10755	down	-7.74665	down
1.383278	up	-7.73096	down
-2.52033	down	7.717025	up
2.119287	up	7.687585	up
-1.35601	down	7.657286	up
1.383481	up	7.643929	up

1.058667	up	7.633419	up
-3.40236	down	7.598733	up
1.061393	up	-7.58246	down
3.238688	up	7.581988	up
1.804151	up	7.560363	up
2.645296	up	7.501583	up
-1.27725	down	7.498723	up
1.045226	up	7.473171	up
1.739974	up	7.465504	up
-4.68927	down	7.464906	up
2.619373	up	-7.45535	down
1.237757	up	7.434008	up
1.137304	up	-7.37525	down
2.85748	up	7.365895	up
-2.27872	down	7.303225	up
1.398399	up	7.286193	up
-2.01262	down	7.282808	up
1.922106	up	-7.24709	down
-1.53916	down	7.220876	up
6.551223	up	7.187307	up
-1.43333	down	7.18505	up
-1.04426	down	7.181497	up
-1.05669	down	-7.18088	down
-1.11114	down	7.168596	up
-1.07278	down	7.164187	up
-1.67507	down	7.158602	up
-3.51438	down	7.157363	up
1.17722	up	7.15281	up
1.073932	up	7.144861	up
1.251881	up	7.115701	up
1.078229	up	7.09962	up
-1.56422	down	7.09497	up
-2.81612	down	7.089139	up
-1.2229	down	-7.08329	down
1.73241	up	7.082948	up
2.805556	up	-7.0428	down
3.323168	up	7.019603	up
1.044232	up	7.015046	up
-1.13401	down	6.988661	up
1.620965	up	-6.97155	down
-1.59813	down	-6.90863	down
1.446653	up	6.881675	up
1.309131	up	6.875482	up
-1.34023	down	6.839175	up
1.200975	up	-6.83587	down
-2.16814	down	-6.833	down
2.217412	up	6.83033	up

1.893808	up	6.820087	up
-1.36959	down	6.801902	up
1.418247	up	-6.78748	down
1.30234	up	-6.77915	down
-1.2207	down	-6.77529	down
-2.59204	down	6.757582	up
1.104348	up	6.735894	up
1.07918	up	-6.71035	down
-1.05474	down	-6.69672	down
-1.14081	down	6.688684	up
1.988169	up	6.685619	up
-1.23032	down	6.662136	up
1.997965	up	6.643154	up
1.678822	up	-6.63647	down
-1.44057	down	6.628013	up
1.456702	up	6.610368	up
-1.00397	down	6.598988	up
17.60029	up	6.585736	up
-2.04697	down	-6.57409	down
-1.6679	down	-6.56702	down
1.932597	up	6.551048	up
-1.21041	down	-6.48448	down
1.156487	up	6.479182	up
-1.20274	down	6.468407	up
1.024952	up	6.4561	up
-2.67392	down	6.388665	up
-1.55138	down	6.386907	up
-1.34369	down	-6.3791	down
-2.1618	down	6.370084	up
2.473746	up	-6.36223	down
-1.13816	down	-6.30674	down
-2.28441	down	6.297653	up
-1.70943	down	6.294227	up
2.140699	up	6.284668	up
2.682087	up	6.275956	up
-1.21932	down	6.27095	up
-1.15213	down	6.248566	up
1.086677	up	-6.2159	down
-8.64282	down	6.213969	up
1.028382	up	6.202655	up
3.165583	up	6.179647	up
1.45464	up	6.130714	up
1.020659	up	-6.11569	down
1.304073	up	6.07943	up
-1.83085	down	6.077913	up
-1.78193	down	-6.07646	down
-1.20595	down	6.063599	up

1.822121	up	6.062529	up
-1.3676	down	-6.04608	down
-4.80189	down	6.038987	up
1.391801	up	6.034341	up
-4.36508	down	6.025446	up
2.718895	up	-6.00927	down
1.278622	up	5.997272	up
1.039372	up	5.993509	up
-1.01408	down	-5.99308	down
-1.13593	down	-5.9741	down
-1.3141	down	5.965407	up
-1.12444	down	-5.95775	down
-1.88384	down	5.953046	up
1.043777	up	5.94429	up
-2.04578	down	5.939481	up
-2.58167	down	5.894855	up
1.59529	up	-5.88867	down
-1.11357	down	5.848183	up
-1.50761	down	5.804433	up
2.131092	up	5.801796	up
-1.11284	down	5.769219	up
-1.75586	down	5.755329	up
5.951249	up	5.745984	up
1.833538	up	5.744577	up
1.126205	up	-5.73921	down
-1.077	down	5.725166	up
-1.10928	down	-5.72325	down
-2.27963	down	-5.72042	down
-4.13223	down	5.71735	up
1.310062	up	5.71184	up
-4.43635	down	5.664622	up
1.609763	up	-5.65724	down
-1.61828	down	-5.65396	down
1.35818	up	-5.6321	down
2.563895	up	5.625846	up
-1.19086	down	5.607769	up
1.570333	up	-5.59878	down
1.449331	up	5.594573	up
2.562471	up	-5.59451	down
-15.0164	down	5.585529	up
-1.14452	down	5.578231	up
-2.8433	down	5.553924	up
-1.33038	down	-5.55075	down
-1.23361	down	-5.53316	down
-1.05343	down	-5.51415	down
-1.2824	down	5.508999	up
1.181162	up	5.504248	up

3.968341	up	5.498441	up
-4.85015	down	5.494378	up
-1.05526	down	5.489025	up
1.510702	up	5.483078	up
1.377515	up	5.472486	up
-1.06818	down	5.462707	up
6.219558	up	5.46204	up
1.405294	up	5.444965	up
-1.50605	down	5.426492	up
1.038495	up	5.415569	up
-1.18772	down	5.402241	up
-1.67446	down	5.379262	up
2.462366	up	5.368329	up
-1.85332	down	-5.3613	down
1.283656	up	5.354447	up
-1.78679	down	-5.34169	down
1.121224	up	5.335933	up
3.013659	up	5.324978	up
1.501096	up	5.291899	up
1.107878	up	5.284756	up
-1.10966	down	-5.28342	down
1.315457	up	-5.27098	down
1.12979	up	5.265006	up
1.224873	up	5.262077	up
1.024195	up	5.251268	up
1.965593	up	-5.24533	down
1.368189	up	5.243077	up
3.470868	up	5.224819	up
-1.30724	down	5.212558	up
1.377043	up	5.210162	up
1.857124	up	5.203596	up
1.08027	up	5.180244	up
-1.35741	down	5.164074	up
-1.01119	down	5.163532	up
1.000138	up	-5.16329	down
-1.13564	down	5.162436	up
-1.19584	down	5.153616	up
-1.34009	down	5.140032	up
-2.41513	down	5.132603	up
2.04127	up	5.128839	up
-1.86142	down	-5.12516	down
-1.45758	down	5.124393	up
-1.88995	down	5.123949	up
-1.06236	down	-5.1214	down
1.298743	up	-5.12129	down
-1.0723	down	5.119819	up
1.588275	up	5.116402	up

2.841915	up	-5.11285	down
-1.76769	down	5.111124	up
-1.05383	down	5.097275	up
1.105031	up	-5.09701	down
2.162196	up	5.073597	up
-1.54461	down	5.059313	up
1.636777	up	5.058074	up
-1.72601	down	-5.05734	down
1.355577	up	5.056087	up
-1.36168	down	5.054138	up
2.64301	up	-5.04856	down
1.018902	up	5.047476	up
-1.34435	down	5.045001	up
1.770984	up	-5.04335	down
1.628224	up	5.035708	up
-2.20838	down	5.033663	up
1.533009	up	5.030851	up
-1.93898	down	5.030629	up
1.01866	up	5.027501	up
1.003209	up	-5.0196	down
-1.17181	down	-5.01217	down
-1.85736	down	-4.99963	down
-1.18968	down	4.999251	up
1.343326	up	4.997247	up
1.852504	up	4.996087	up
-3.82749	down	4.99196	up
-2.37618	down	4.967118	up
1.583646	up	4.949797	up
-1.04613	down	4.94877	up
-1.43675	down	4.92135	up
1.176544	up	4.918611	up
1.783593	up	4.903329	up
1.332401	up	4.900374	up
-1.95508	down	4.89206	up
-1.22846	down	4.886253	up
2.049848	up	-4.86585	down
2.206351	up	4.858898	up
-2.35649	down	4.85723	up
-10.806	down	4.837502	up
-3.18034	down	4.816393	up
15.81805	up	4.815331	up
-2.01016	down	4.811048	up
1.373468	up	4.807261	up
1.985508	up	4.805387	up
1.912233	up	4.80329	up
-1.57694	down	4.802166	up
-1.95581	down	4.78816	up

-7.40515	down	4.779413	up
1.95149	up	4.77622	up
1.765672	up	4.763105	up
1.207589	up	-4.75	down
-1.09767	down	4.724098	up
-1.03012	down	4.711794	up
1.960047	up	4.709842	up
2.693373	up	4.696422	up
1.286342	up	4.684228	up
-1.62848	down	4.683806	up
1.255424	up	-4.68128	down
-1.26344	down	4.674586	up
-1.9888	down	4.673121	up
1.231061	up	-4.66392	down
1.013128	up	-4.66229	down
-1.8688	down	4.657694	up
-1.6153	down	4.657203	up
1.285691	up	-4.65022	down
3.229933	up	4.649017	up
-1.90403	down	4.641118	up
-1.0772	down	-4.64085	down
1.091671	up	4.627686	up
1.27543	up	-4.62282	down
1.323535	up	4.62141	up
-1.65359	down	4.609752	up
1.216582	up	4.60517	up
-1.15932	down	-4.60485	down
-1.55034	down	4.600843	up
-1.09477	down	-4.59883	down
1.405835	up	-4.59578	down
7.371954	up	4.595694	up
1.295955	up	4.59376	up
-1.5659	down	-4.59165	down
-1.33051	down	4.582013	up
1.407009	up	4.575794	up
-1.2088	down	4.572863	up
-3.44704	down	4.571094	up
4.623538	up	4.568589	up
2.946864	up	4.564633	up
-2.19343	down	4.553103	up
1.62366	up	-4.55291	down
1.873281	up	4.547481	up
-1.41689	down	4.536642	up
-1.26418	down	4.536566	up
4.006994	up	4.534203	up
1.005104	up	4.523729	up
-1.01976	down	4.520993	up

-1.4295	down	4.514876	up
1.066837	up	4.510276	up
1.651599	up	4.509907	up
1.863094	up	4.503609	up
-3.43037	down	-4.49971	down
1.345864	up	-4.49355	down
1.104065	up	-4.48846	down
1.584789	up	-4.48812	down
1.46084	up	-4.48238	down
1.003608	up	4.48121	up
1.380155	up	4.470828	up
-1.1219	down	-4.46679	down
2.452386	up	4.457587	up
-2.42699	down	4.454128	up
1.572347	up	4.453299	up
1.309199	up	-4.44327	down
1.266248	up	-4.44184	down
-1.45069	down	-4.43541	down
-1.15677	down	4.427822	up
1.352011	up	-4.42702	down
1.354766	up	4.416956	up
-2.8681	down	-4.40627	down
1.846055	up	4.406084	up
4.542592	up	4.405418	up
7.302703	up	4.389818	up
-1.01074	down	-4.38172	down
-1.43664	down	4.379011	up
1.541203	up	4.378677	up
-1.21937	down	4.372923	up
-1.05234	down	-4.37155	down
-1.21341	down	-4.37095	down
1.577406	up	4.366971	up
1.006432	up	-4.34553	down
1.180141	up	4.337905	up
2.973436	up	4.337628	up
-1.76997	down	4.337587	up
2.344523	up	-4.33311	down
1.220156	up	-4.33311	down
-3.2746	down	-4.33127	down
-1.26247	down	-4.32473	down
-2.36555	down	4.319939	up
1.186031	up	-4.31119	down
-1.11345	down	4.301686	up
1.557211	up	4.296017	up
-1.47074	down	4.287694	up
-1.43026	down	-4.28448	down
1.84608	up	4.28001	up

2.858017	up	4.280007	up
1.038672	up	-4.27979	down
-1.42372	down	4.278525	up
1.102268	up	-4.27353	down
1.100122	up	-4.27292	down
-1.06654	down	4.269825	up
1.559584	up	4.266226	up
-2.51118	down	4.26501	up
-1.17688	down	4.261819	up
4.105022	up	-4.25771	down
-1.9853	down	-4.25451	down
1.09074	up	4.247722	up
1.572093	up	4.231888	up
1.41915	up	4.231578	up
-1.1418	down	-4.22999	down
1.445138	up	4.22809	up
-1.46119	down	4.227618	up
2.418578	up	4.223355	up
-2.87729	down	4.222684	up
-1.45443	down	4.219931	up
-12.1621	down	4.214052	up
1.286553	up	-4.21335	down
2.324198	up	4.210941	up
-1.31259	down	4.208478	up
1.118853	up	-4.20457	down
1.475359	up	4.203691	up
-1.26436	down	-4.20186	down
-1.70386	down	-4.20101	down
2.154078	up	4.200573	up
-6.53246	down	4.1924	up
1.140809	up	4.18525	up
1.134402	up	4.17449	up
-1.96702	down	-4.17282	down
-1.79526	down	-4.17028	down
-1.16472	down	4.169658	up
-16.6424	down	4.167274	up
1.238945	up	-4.15559	down
-1.57987	down	4.146764	up
1.709288	up	4.145345	up
1.221278	up	4.143054	up
2.031118	up	-4.14203	down
4.520858	up	4.14101	up
-1.0028	down	4.140093	up
5.913166	up	4.138518	up
-1.46147	down	4.138238	up
-2.29419	down	-4.13554	down
5.178447	up	4.132451	up

1.661439	up	-4.13023	down
-1.90336	down	-4.12866	down
-1.13346	down	-4.12511	down
1.400802	up	4.117892	up
-1.71527	down	4.117796	up
1.47266	up	4.116634	up
-1.53209	down	-4.1145	down
-1.1034	down	4.108592	up
2.68201	up	4.106391	up
-1.97348	down	4.106381	up
-2.66257	down	4.105482	up
1.359383	up	4.104532	up
1.497397	up	4.098742	up
-1.3415	down	4.094393	up
-1.26868	down	-4.09206	down
-2.22358	down	4.089724	up
2.611007	up	4.089358	up
-1.15653	down	4.087277	up
2.336979	up	-4.08591	down
4.037053	up	4.07112	up
1.044229	up	4.066585	up
-1.6027	down	4.061352	up
1.038398	up	-4.0571	down
2.192073	up	4.056266	up
7.703261	up	4.043671	up
1.16853	up	-4.0376	down
2.293212	up	4.036508	up
1.428497	up	-4.03499	down
-1.00196	down	4.03387	up
1.009367	up	4.031961	up
-1.38095	down	4.022097	up
1.009794	up	-4.01583	down
-2.37156	down	4.015797	up
1.294708	up	4.008868	up
1.584932	up	4.008196	up
1.003115	up	4.007257	up
-1.31633	down	3.992446	up
1.976796	up	3.991514	up
-1.06183	down	-3.98248	down
-1.49887	down	3.978383	up
1.600856	up	3.977573	up
1.039952	up	3.974605	up
3.548511	up	3.974359	up
-2.21418	down	3.973311	up
-1.19076	down	-3.96636	down
-3.1366	down	3.96517	up
-1.64489	down	-3.95737	down

-1.34731	down	-3.95283	down
2.267063	up	3.95169	up
1.245444	up	3.949807	up
-1.08635	down	-3.94525	down
-7.60271	down	3.936726	up
-1.09363	down	-3.93377	down
2.044129	up	3.931712	up
-5.34303	down	3.927393	up
-2.87021	down	3.925445	up
1.097767	up	3.92283	up
-1.60993	down	-3.91932	down
-3.27817	down	3.916397	up
1.050685	up	-3.91348	down
-1.29873	down	3.909389	up
-1.14717	down	3.908993	up
1.079589	up	3.904427	up
3.245987	up	3.902488	up
-1.20726	down	3.900749	up
-1.4179	down	3.900507	up
-1.64049	down	3.900186	up
1.272662	up	3.897717	up
2.227405	up	3.896992	up
1.827227	up	3.893597	up
1.637153	up	-3.89032	down
1.203089	up	3.889474	up
-1.1287	down	-3.88892	down
2.51481	up	3.884868	up
-1.07714	down	3.884396	up
-2.03604	down	3.87865	up
-2.30369	down	3.876813	up
-1.3864	down	3.873052	up
-2.34622	down	3.871441	up
-1.18017	down	-3.86243	down
-1.46689	down	3.851711	up
-2.46067	down	3.849843	up
-1.21988	down	-3.8493	down
-1.33108	down	3.842421	up
1.366992	up	3.84077	up
1.483708	up	3.840587	up
-1.46279	down	-3.83017	down
1.175593	up	-3.82944	down
1.465272	up	3.829225	up
-1.53281	down	3.82469	up
-1.10715	down	-3.8225	down
-1.11575	down	3.819579	up
-1.56034	down	3.818572	up
-1.07244	down	3.806066	up

-1.10355	down	3.804422	up
1.54326	up	3.803124	up
2.623753	up	-3.79739	down
1.005701	up	-3.79662	down
-2.62185	down	3.796058	up
-1.01552	down	-3.79213	down
-1.34991	down	3.78769	up
1.420882	up	3.782066	up
1.314952	up	3.77997	up
1.178515	up	-3.77859	down
2.761015	up	-3.77714	down
-1.6286	down	3.775921	up
1.186337	up	-3.77265	down
-1.5975	down	-3.77253	down
-1.6993	down	-3.77221	down
-1.2662	down	3.771007	up
1.190663	up	3.767281	up
-9.153	down	3.766453	up
2.993539	up	3.76392	up
-1.02805	down	3.762447	up
1.623186	up	3.759936	up
-3.91793	down	3.758279	up
-1.69339	down	3.756316	up
-1.19278	down	-3.75619	down
-2.20921	down	-3.75313	down
-3.89514	down	3.752384	up
2.952111	up	3.750318	up
-1.07627	down	-3.74766	down
-1.24471	down	-3.747	down
-1.29245	down	-3.74619	down
-2.31458	down	3.743957	up
-1.06318	down	-3.74033	down
-1.2235	down	3.731211	up
-1.79361	down	3.730623	up
1.360414	up	-3.72659	down
1.378199	up	3.723132	up
-1.11725	down	3.722818	up
-2.3887	down	3.71524	up
-1.12343	down	3.705138	up
1.766938	up	3.70375	up
-1.38872	down	3.700404	up
-2.40605	down	3.698539	up
1.420911	up	-3.69684	down
-1.25805	down	-3.68914	down
-2.06789	down	3.68768	up
1.388638	up	3.685117	up
-1.55685	down	3.68337	up

1.225694	up	3.682947	up
1.879184	up	3.678998	up
-6.76241	down	-3.67027	down
1.386427	up	3.668593	up
-1.61244	down	-3.667	down
1.450606	up	-3.66598	down
1.300575	up	3.663228	up
-1.1144	down	3.661878	up
1.05245	up	3.660853	up
-5.03065	down	3.660638	up
-1.78568	down	-3.65974	down
-1.18169	down	-3.65524	down
3.442898	up	3.653951	up
-1.20865	down	3.646931	up
1.41123	up	3.644482	up
-2.20986	down	3.640633	up
-2.95505	down	3.635639	up
-2.48258	down	3.634032	up
1.455871	up	3.633837	up
-1.01012	down	3.63059	up
1.075922	up	3.628466	up
1.127787	up	3.628303	up
-1.68811	down	-3.62611	down
-5.43453	down	3.623388	up
1.074815	up	3.620036	up
5.34456	up	-3.61526	down
3.95056	up	3.612195	up
-1.10345	down	-3.61177	down
1.091644	up	-3.61151	down
1.108473	up	-3.61056	down
1.409412	up	-3.61045	down
2.094189	up	3.6074	up
3.209972	up	3.60691	up
-1.81348	down	3.605817	up
-1.04028	down	-3.59616	down
-2.70838	down	-3.59395	down
1.068967	up	3.591954	up
1.138441	up	3.591859	up
2.693206	up	3.589523	up
1.032989	up	-3.57992	down
-1.03437	down	-3.57989	down
1.088183	up	3.577033	up
-1.51057	down	3.576351	up
2.332104	up	3.574943	up
2.407117	up	3.574281	up
-1.73477	down	3.571786	up
-2.06357	down	3.571372	up

-1.76366	down	-3.57122	down
1.756941	up	-3.56868	down
-1.02517	down	-3.56829	down
1.112887	up	-3.5677	down
-1.21217	down	-3.56336	down
-1.13144	down	3.562872	up
1.792767	up	-3.56215	down
-1.07178	down	3.557989	up
-1.56153	down	-3.55251	down
-2.22942	down	3.552338	up
1.201251	up	3.550745	up
1.083411	up	-3.54981	down
-1.18354	down	3.548853	up
1.016013	up	-3.54758	down
1.304708	up	3.544599	up
2.317754	up	-3.54111	down
-2.81098	down	3.534541	up
1.257248	up	3.528007	up
11.32388	up	3.526956	up
-1.30961	down	-3.52498	down
-2.78082	down	3.518467	up
-1.48396	down	3.518379	up
-1.04891	down	3.516586	up
1.012783	up	3.512323	up
-1.06564	down	3.512239	up
1.091825	up	3.50926	up
-1.22139	down	-3.50854	down
1.215019	up	3.507642	up
1.556953	up	-3.50698	down
-2.06207	down	3.502399	up
1.189639	up	3.502164	up
1.498376	up	-3.5016	down
1.233457	up	3.498114	up
-1.13456	down	3.498059	up
1.544197	up	3.493298	up
-2.25008	down	3.491786	up
1.00259	up	3.489914	up
-1.53239	down	-3.48911	down
-1.00768	down	3.484968	up
-1.26895	down	-3.48445	down
-1.31152	down	-3.48417	down
1.175014	up	3.480163	up
1.184822	up	3.478011	up
1.452283	up	3.476111	up
-2.94355	down	3.472707	up
1.575231	up	-3.46693	down
-1.54439	down	3.461069	up

4.015279	up	3.457421	up
2.48501	up	3.457351	up
1.685844	up	3.455174	up
1.414209	up	3.449307	up
1.378993	up	-3.4473	down
-1.02761	down	-3.44702	down
1.19607	up	-3.44669	down
-12.3119	down	3.444203	up
1.019618	up	3.436943	up
-1.08443	down	3.436029	up
1.540321	up	3.435093	up
-3.06581	down	3.43482	up
1.824774	up	-3.43412	down
-1.26048	down	-3.43181	down
-2.04527	down	3.429213	up
1.496906	up	-3.42268	down
-2.64566	down	3.421831	up
1.167622	up	3.420988	up
-1.24186	down	3.419121	up
-1.14409	down	3.41765	up
1.263369	up	3.417166	up
1.47421	up	3.415479	up
-1.02851	down	-3.41535	down
2.728348	up	3.41474	up
1.445169	up	-3.41294	down
1.022265	up	-3.41073	down
2.524781	up	-3.40875	down
2.143037	up	-3.40763	down
-1.18048	down	3.405422	up
-1.23367	down	3.403772	up
1.161054	up	3.403276	up
-1.26675	down	3.402359	up
-1.81462	down	3.399416	up
1.205277	up	-3.39938	down
1.661127	up	3.396944	up
1.975969	up	-3.39602	down
-2.02446	down	-3.38989	down
1.001082	up	3.38807	up
-1.15888	down	-3.38787	down
-1.18933	down	3.387734	up
1.594691	up	-3.38719	down
1.258858	up	3.383567	up
-2.09077	down	3.382738	up
2.945586	up	3.38181	up
-1.10016	down	3.378414	up
-1.57051	down	3.371622	up
-1.2845	down	3.364222	up

1.046011	up	-3.36382	down
1.463332	up	-3.36376	down
-2.39393	down	3.363423	up
-4.51959	down	-3.36271	down
1.050392	up	3.355543	up
1.681369	up	-3.35027	down
-1.07684	down	-3.34823	down
1.41242	up	3.347106	up
-1.34817	down	3.346055	up
1.453805	up	3.345161	up
3.222141	up	3.343706	up
-1.09282	down	-3.34274	down
-1.12504	down	3.340552	up
1.212812	up	-3.33654	down
1.012544	up	-3.33451	down
2.230952	up	3.33361	up
2.72785	up	3.330252	up
-1.25951	down	3.329893	up
-1.466	down	3.329891	up
-1.2664	down	-3.32964	down
-2.6254	down	3.324661	up
1.637192	up	3.323009	up
-2.77271	down	3.32239	up
1.241506	up	3.318972	up
1.403916	up	3.318228	up
-2.32668	down	3.318096	up
1.422751	up	3.317938	up
1.913122	up	3.316775	up
-1.84867	down	3.316118	up
1.080808	up	-3.30608	down
-2.05085	down	3.305957	up
-1.0069	down	3.304998	up
1.336834	up	3.304093	up
-1.49662	down	3.300864	up
-1.31777	down	3.299429	up
1.106981	up	-3.29842	down
-1.16687	down	3.2973	up
-1.21568	down	3.296812	up
1.871271	up	-3.29383	down
1.148785	up	-3.29153	down
-2.25165	down	3.290965	up
-3.16406	down	3.290049	up
1.057697	up	3.288174	up
1.586237	up	3.284069	up
-1.10146	down	3.283835	up
-1.3887	down	-3.28293	down
-1.19164	down	3.280383	up

-1.03895	down	-3.28033	down
2.330003	up	3.278253	up
1.09592	up	3.278118	up
-1.38454	down	-3.27702	down
-1.03616	down	-3.2766	down
1.237404	up	-3.2766	down
1.157526	up	-3.27294	down
1.180722	up	-3.27026	down
1.081038	up	3.266373	up
-1.36485	down	-3.26209	down
1.136151	up	-3.25899	down
-1.24536	down	3.258328	up
-1.32142	down	-3.25721	down
-1.41466	down	3.255713	up
-1.31359	down	3.255679	up
1.008954	up	3.255164	up
-1.6966	down	-3.25358	down
1.237128	up	-3.25297	down
2.733067	up	3.252612	up
-1.45847	down	3.252345	up
-1.44974	down	3.251754	up
1.205293	up	3.250572	up
-1.01259	down	3.250256	up
-1.12842	down	3.249537	up
-1.95849	down	-3.24515	down
-1.4999	down	3.244067	up
1.963087	up	3.242828	up
1.48151	up	3.241815	up
-1.38076	down	-3.24042	down
-1.89299	down	-3.23947	down
-1.20723	down	-3.23911	down
1.183564	up	3.2371	up
-1.45497	down	-3.23692	down
1.29065	up	3.2363	up
-1.44885	down	-3.23246	down
-3.07492	down	3.231424	up
-2.11754	down	-3.22648	down
-1.43079	down	3.226228	up
-1.76591	down	-3.22543	down
-1.04571	down	3.224315	up
1.026089	up	3.221435	up
-1.57251	down	3.218387	up
-1.1138	down	3.217307	up
5.810159	up	3.21721	up
-3.5381	down	3.21718	up
-1.61862	down	3.214678	up
2.036319	up	3.212785	up

2.115253	up	3.211668	up
1.624716	up	-3.20936	down
2.436331	up	-3.20905	down
1.509951	up	-3.20624	down
1.39602	up	-3.20528	down
1.088088	up	-3.20008	down
1.305275	up	-3.19893	down
1.008883	up	-3.19823	down
1.414132	up	3.194882	up
-1.04578	down	-3.19304	down
2.900672	up	3.192954	up
-1.20059	down	3.188245	up
2.221793	up	3.186562	up
4.390033	up	3.183752	up
1.879156	up	-3.18207	down
1.232955	up	3.181011	up
-1.40144	down	-3.17768	down
1.913209	up	3.17749	up
-1.44653	down	3.17742	up
-7.70323	down	3.176905	up
1.909107	up	3.17341	up
1.622262	up	3.170506	up
1.434019	up	3.169937	up
1.135231	up	3.165728	up
-3.08638	down	3.164647	up
5.630995	up	3.16429	up
1.048086	up	3.1631	up
-2.06171	down	3.161515	up
-2.27089	down	3.160096	up
1.04126	up	3.159583	up
1.33607	up	-3.15886	down
1.093185	up	3.158774	up
-4.19109	down	3.158278	up
-1.32247	down	-3.15447	down
1.18616	up	-3.14922	down
-1.14363	down	-3.1474	down
-1.24104	down	3.143517	up
1.054055	up	-3.14208	down
2.314665	up	3.141846	up
1.079567	up	3.140686	up
1.862951	up	3.140107	up
1.269722	up	-3.13982	down
-4.42941	down	3.139573	up
-1.22225	down	3.135936	up
1.098278	up	3.134812	up
1.516045	up	3.134553	up
-1.5584	down	3.132955	up

-1.53599	down	3.132824	up
1.456017	up	3.132636	up
1.375274	up	3.131838	up
2.458374	up	3.129993	up
2.236787	up	3.129299	up
-1.08779	down	3.12723	up
-1.60982	down	3.126567	up
-2.15044	down	3.125576	up
1.851116	up	3.123218	up
1.169179	up	-3.12185	down
-1.18098	down	3.121201	up
-1.32428	down	3.117364	up
-1.91414	down	3.112928	up
-2.37983	down	3.112354	up
1.398502	up	3.111678	up
-1.07205	down	-3.10903	down
1.095396	up	-3.10855	down
1.438413	up	3.107599	up
-1.50009	down	3.104502	up
1.025113	up	3.102214	up
1.288442	up	3.102011	up
-1.86407	down	-3.09828	down
-1.42212	down	-3.09721	down
-1.59877	down	-3.09555	down
-1.69751	down	3.095027	up
1.247783	up	3.09278	up
1.502248	up	3.091835	up
-1.28955	down	3.089763	up
-1.02121	down	-3.08785	down
1.434638	up	3.08604	up
1.136406	up	3.085072	up
-1.16101	down	-3.08429	down
-1.36084	down	-3.08296	down
1.223984	up	3.082733	up
-1.21152	down	3.082589	up
-1.7465	down	-3.07889	down
1.433114	up	3.078242	up
1.505722	up	3.077916	up
-1.12778	down	3.077316	up
1.059699	up	3.07506	up
-1.71987	down	-3.07146	down
1.055647	up	3.069085	up
-1.7811	down	3.068638	up
1.249406	up	-3.06772	down
1.145731	up	3.067593	up
-1.08476	down	3.066056	up
-1.48478	down	3.065731	up

1.604267	up	3.065154	up
4.570034	up	3.057395	up
2.281955	up	-3.05372	down
-1.65163	down	3.048042	up
3.891317	up	-3.04745	down
-1.80821	down	-3.04528	down
1.20278	up	3.044045	up
1.017829	up	-3.04309	down
2.084076	up	-3.042	down
-2.91356	down	3.040388	up
1.159249	up	3.040258	up
1.558826	up	3.038612	up
-1.20451	down	-3.03693	down
-1.48872	down	-3.03003	down
-1.40934	down	-3.02718	down
1.149404	up	3.024692	up
2.626265	up	3.023576	up
1.015918	up	3.023174	up
1.474032	up	-3.0218	down
2.491224	up	3.020649	up
-1.18972	down	-3.0184	down
1.331194	up	3.015696	up
1.012956	up	3.014682	up
1.349257	up	-3.0136	down
1.458149	up	3.010787	up
-2.18895	down	3.01025	up
3.552571	up	3.00722	up
1.534394	up	3.006701	up
-1.28985	down	3.006456	up
1.778992	up	-3.00422	down
1.126595	up	-3.00338	down
1.502886	up	3.002904	up
-1.64326	down	3.002564	up
1.086876	up	3.00049	up
-1.26398	down	2.996969	up
1.330621	up	2.996602	up
1.230815	up	2.994757	up
1.687086	up	2.992934	up
-3.52439	down	2.992551	up
1.059526	up	2.988488	up
3.386894	up	2.986656	up
1.333742	up	2.986454	up
1.344231	up	2.985297	up
2.01682	up	2.985134	up
-2.10172	down	-2.98488	down
-1.46005	down	-2.98391	down
2.1046	up	-2.98361	down

-1.26742	down	2.983223	up
1.642237	up	2.981701	up
1.070004	up	2.981275	up
1.372458	up	-2.98065	down
1.394962	up	2.980229	up
1.98338	up	2.978423	up
-1.81425	down	2.97666	up
1.020293	up	-2.97437	down
1.593335	up	2.969735	up
-1.68589	down	2.968767	up
-2.14342	down	2.96822	up
20.16946	up	2.966879	up
1.483477	up	2.964778	up
1.754222	up	-2.96425	down
-1.09236	down	-2.96365	down
-1.44118	down	-2.96317	down
1.120322	up	-2.96312	down
1.675106	up	2.962621	up
-1.02773	down	2.961242	up
-1.41959	down	2.959713	up
-2.2502	down	2.957155	up
2.374255	up	-2.95689	down
1.354365	up	-2.95548	down
-1.0439	down	-2.95539	down
2.167649	up	2.954858	up
1.423228	up	2.953203	up
-1.00858	down	2.952933	up
5.51491	up	2.952744	up
-1.1169	down	-2.9522	down
-1.28888	down	2.952144	up
-1.02807	down	-2.94971	down
1.032015	up	2.949627	up
1.205388	up	-2.94949	down
4.323813	up	-2.94934	down
1.094414	up	2.947418	up
-1.55592	down	-2.94659	down
-1.12032	down	-2.9443	down
-1.06565	down	-2.94216	down
1.616838	up	-2.94156	down
1.464948	up	2.939563	up
-3.09916	down	-2.93829	down
1.369218	up	2.936989	up
2.001554	up	-2.93646	down
-2.27194	down	2.936174	up
-1.06958	down	-2.93564	down
-1.94434	down	-2.93427	down
-1.24568	down	2.933559	up

-2.21592	down	2.933126	up
-1.3618	down	2.932903	up
-1.11609	down	2.932692	up
1.003071	up	2.930238	up
10.38355	up	2.927601	up
3.420738	up	2.927051	up
-1.07713	down	-2.92678	down
-1.63714	down	2.926704	up
1.130952	up	-2.92533	down
-1.13624	down	2.92438	up
2.518648	up	2.924029	up
-2.47201	down	2.922898	up
1.353739	up	2.922274	up
-2.19885	down	2.92098	up
1.378299	up	2.920575	up
-1.07479	down	-2.91891	down
-1.68999	down	2.917836	up
2.347208	up	2.915373	up
1.34005	up	2.91438	up
1.411884	up	2.912213	up
-1.21986	down	-2.90668	down
-1.07442	down	2.904888	up
-6.26196	down	-2.9044	down
1.303631	up	2.904079	up
1.086391	up	2.903264	up
1.172604	up	2.901625	up
2.011144	up	-2.8971	down
1.207505	up	2.89458	up
-1.48044	down	2.89287	up
-1.96571	down	-2.89183	down
1.316918	up	2.891102	up
-1.56555	down	2.890992	up
1.329629	up	2.89086	up
-1.3548	down	2.890684	up
3.024396	up	2.890664	up
1.189176	up	-2.8901	down
-1.29541	down	-2.8898	down
-1.53708	down	-2.88964	down
-1.25927	down	2.888283	up
1.700012	up	-2.88731	down
-1.52199	down	2.886941	up
-1.07856	down	-2.88677	down
1.415664	up	-2.88462	down
1.261432	up	-2.88295	down
1.317026	up	2.882627	up
-1.09109	down	-2.87986	down
1.228833	up	-2.87947	down

-1.16404	down	2.877519	up
1.026967	up	-2.87742	down
-1.30615	down	2.87662	up
-1.63377	down	2.87655	up
1.585776	up	2.87616	up
-1.11309	down	2.876063	up
-1.79855	down	-2.87591	down
2.869146	up	2.8758	up
1.215128	up	-2.87549	down
1.60119	up	-2.87507	down
-2.27342	down	2.87446	up
1.171264	up	2.873266	up
1.33117	up	2.873127	up
-1.061	down	2.873112	up
1.15938	up	-2.873	down
1.064894	up	-2.86965	down
2.187433	up	2.867616	up
1.116819	up	-2.86514	down
-1.38231	down	-2.86394	down
-1.3952	down	2.862024	up
1.811812	up	2.861413	up
-11.7496	down	2.859698	up
-1.78912	down	-2.859	down
-1.16291	down	2.858396	up
-1.25429	down	2.857438	up
-2.22245	down	-2.85197	down
1.025359	up	-2.84967	down
-1.2298	down	-2.84955	down
1.374153	up	2.848655	up
1.003875	up	2.847833	up
3.29099	up	2.846681	up
2.05787	up	2.846456	up
-3.94168	down	2.845555	up
1.419377	up	-2.84393	down
-1.09997	down	-2.84271	down
-1.15157	down	2.841797	up
-1.00825	down	-2.84162	down
-1.30761	down	-2.84139	down
1.10926	up	-2.84107	down
1.051763	up	-2.83832	down
-1.11442	down	-2.83726	down
-2.15098	down	2.836988	up
1.95147	up	2.834216	up
4.026541	up	2.833242	up
-1.00274	down	2.832085	up
1.23763	up	2.831736	up
-1.73972	down	-2.8311	down

4.367728	up	2.830917	up
-1.15368	down	-2.83012	down
1.196356	up	2.829829	up
1.86857	up	2.828276	up
1.642408	up	2.828175	up
-2.00113	down	2.827915	up
1.186598	up	2.82703	up
-1.36154	down	-2.82606	down
2.143359	up	2.825877	up
1.716209	up	2.824877	up
3.300091	up	2.824092	up
3.287839	up	-2.82348	down
1.067668	up	-2.82202	down
2.028594	up	-2.8206	down
1.256515	up	-2.81898	down
1.669483	up	2.818871	up
-1.068	down	2.817926	up
2.772049	up	2.817298	up
-2.74782	down	2.815951	up
1.290876	up	2.815782	up
-2.08962	down	2.81448	up
1.156571	up	2.814228	up
1.08729	up	-2.81397	down
2.825393	up	2.811429	up
-1.09809	down	-2.8114	down
-1.36807	down	-2.81063	down
1.245693	up	-2.80987	down
2.109137	up	2.807488	up
-1.35216	down	2.80701	up
1.442235	up	2.80694	up
-2.02533	down	2.806335	up
1.489231	up	-2.80616	down
-1.96486	down	2.805876	up
1.140251	up	-2.80507	down
-1.19461	down	-2.80483	down
1.405689	up	-2.80288	down
1.510551	up	2.802746	up
2.278357	up	-2.80245	down
2.785878	up	-2.80206	down
-1.64599	down	2.800903	up
-5.25939	down	2.800866	up
-1.23574	down	-2.8005	down
-1.00773	down	-2.80029	down
1.156678	up	2.798142	up
1.249095	up	-2.79671	down
1.056731	up	2.796513	up
-14.4652	down	2.794493	up

-2.67221	down	2.794185	up
1.575223	up	2.791797	up
-1.80142	down	-2.79025	down
1.682954	up	2.789938	up
-1.42401	down	2.789869	up
-1.22629	down	2.789417	up
-1.06901	down	-2.78871	down
-1.77157	down	2.788223	up
1.178906	up	-2.78808	down
2.24663	up	2.788041	up
-1.12271	down	-2.7865	down
1.350893	up	2.785481	up
7.191232	up	2.785098	up
-1.82898	down	-2.78502	down
-1.17091	down	2.784988	up
1.440913	up	2.783425	up
1.068456	up	-2.78031	down
1.312065	up	2.779613	up
1.369694	up	2.779578	up
-1.41036	down	-2.77702	down
1.017117	up	2.774048	up
1.65409	up	2.773265	up
-1.22575	down	-2.7732	down
2.811193	up	2.772194	up
-1.13102	down	-2.77147	down
1.258248	up	-2.7705	down
1.053116	up	2.770086	up
1.389085	up	2.768567	up
-1.35492	down	-2.76838	down
1.173176	up	2.767064	up
-1.11569	down	-2.76701	down
1.344967	up	2.766864	up
1.383531	up	-2.76597	down
1.008377	up	2.765668	up
-2.335	down	-2.76539	down
1.268043	up	2.764136	up
-1.24058	down	2.763143	up
-1.101	down	-2.76005	down
-1.69492	down	2.759315	up
-1.23183	down	2.758539	up
-1.32254	down	-2.7571	down
1.567647	up	2.756143	up
-1.00219	down	2.755337	up
-1.23912	down	-2.75508	down
-1.00541	down	2.754526	up
1.024522	up	2.754451	up
-1.73016	down	2.753051	up

1.151591	up	2.752995	up
-4.17684	down	2.752386	up
1.102615	up	-2.75151	down
-1.07291	down	-2.75051	down
1.33399	up	2.749409	up
-1.20901	down	-2.74925	down
2.109133	up	-2.74332	down
-1.0401	down	2.743306	up
-1.58345	down	2.742996	up
-1.2417	down	-2.74297	down
-1.16756	down	-2.74105	down
-1.63632	down	2.739785	up
-1.19256	down	-2.73975	down
1.116216	up	2.739147	up
1.084348	up	-2.73851	down
1.000768	up	2.737149	up
-1.29319	down	2.734024	up
-1.00483	down	-2.734	down
-1.02226	down	-2.73393	down
-1.23894	down	-2.73385	down
1.018219	up	-2.73237	down
1.856963	up	2.731959	up
-1.04792	down	2.731882	up
-1.66689	down	2.730712	up
-1.34546	down	-2.73007	down
2.159166	up	2.729871	up
1.46076	up	2.728911	up
1.174658	up	2.728559	up
1.390928	up	-2.7278	down
1.383397	up	-2.72765	down
-1.46858	down	2.725689	up
-2.03626	down	2.725473	up
2.192863	up	2.724918	up
-1.36892	down	-2.72334	down
-1.33607	down	2.720915	up
1.922773	up	-2.7209	down
1.476558	up	2.720579	up
-1.57344	down	-2.7205	down
-2.17607	down	2.720106	up
-1.72949	down	-2.71909	down
1.902198	up	2.718588	up
1.572099	up	2.718502	up
1.061907	up	2.717188	up
1.558178	up	-2.71559	down
-1.46949	down	2.714962	up
2.299611	up	2.714668	up
-1.60419	down	2.713111	up

-2.5924	down	2.7125	up
-1.18066	down	2.712248	up
-1.53177	down	-2.71148	down
1.453567	up	-2.71036	down
-2.29196	down	2.710221	up
-1.0199	down	-2.70963	down
1.407597	up	2.708944	up
1.277437	up	2.708681	up
-1.16633	down	-2.70719	down
-1.33612	down	-2.70502	down
-1.43617	down	2.704583	up
1.608049	up	2.703975	up
2.479081	up	2.703344	up
1.914616	up	-2.70329	down
1.058634	up	-2.70324	down
1.14891	up	2.702279	up
-1.21901	down	-2.70187	down
1.55583	up	2.701458	up
-1.59939	down	-2.70098	down
-1.25532	down	-2.70026	down
-1.47414	down	2.700031	up
-2.22774	down	-2.69838	down
-1.62147	down	2.69676	up
1.416641	up	2.69563	up
-1.77399	down	2.695295	up
1.385376	up	2.695142	up
1.066938	up	-2.69472	down
1.532144	up	2.693606	up
1.254098	up	-2.69246	down
2.250441	up	2.692032	up
1.276025	up	-2.69071	down
1.415517	up	-2.6901	down
-1.19896	down	2.689332	up
-1.05138	down	2.688179	up
2.220061	up	2.688103	up
-1.14332	down	2.687959	up
1.190382	up	2.687756	up
-1.44385	down	2.687115	up
2.454826	up	2.686064	up
-1.50381	down	-2.68411	down
1.527908	up	2.683308	up
1.291724	up	2.682648	up
1.048346	up	-2.68237	down
1.526912	up	2.681458	up
1.665696	up	2.681359	up
1.131691	up	2.680983	up
1.642209	up	2.680298	up

-1.5524	down	2.679017	up
-1.35854	down	2.676754	up
1.435229	up	2.674743	up
1.672232	up	2.673596	up
3.601776	up	2.673153	up
1.755573	up	2.671878	up
-1.17303	down	2.671865	up
1.92242	up	2.671206	up
1.61613	up	2.671083	up
-1.34377	down	2.670622	up
1.177783	up	-2.67022	down
-1.02621	down	-2.66852	down
-1.28878	down	2.668336	up
-2.384	down	2.666365	up
3.127964	up	2.665862	up
1.080995	up	-2.66478	down
3.103287	up	2.662487	up
1.576506	up	-2.66122	down
1.585474	up	2.661192	up
-1.25754	down	-2.66118	down
-1.0229	down	2.660353	up
-1.34293	down	2.658873	up
1.134898	up	-2.65868	down
-1.54733	down	2.658114	up
1.467358	up	2.656992	up
4.298616	up	2.656395	up
-1.5497	down	-2.65638	down
-1.00633	down	-2.65467	down
1.791631	up	-2.65421	down
3.301826	up	2.653555	up
1.00569	up	-2.65275	down
3.299772	up	2.652386	up
-1.21689	down	2.652155	up
-1.3908	down	2.651793	up
1.300994	up	-2.64924	down
1.741594	up	-2.6485	down
-1.86414	down	-2.64795	down
1.144638	up	-2.6478	down
1.480294	up	2.647642	up
1.932465	up	2.647389	up
-1.05628	down	-2.64686	down
-1.24408	down	-2.64675	down
1.439426	up	2.646735	up
-2.38631	down	2.646678	up
-1.19209	down	2.646429	up
-1.94945	down	2.646138	up
-1.80233	down	2.644361	up

1.965652	up	2.64322	up
2.221575	up	2.642982	up
1.194977	up	2.642738	up
-1.28544	down	-2.6423	down
-1.47908	down	-2.6418	down
-1.59597	down	2.641624	up
1.157464	up	-2.64131	down
-1.11398	down	2.640754	up
-1.60514	down	-2.64045	down
1.352323	up	-2.63938	down
-1.1879	down	-2.63876	down
-1.29081	down	2.638345	up
1.246953	up	2.636143	up
-1.14541	down	-2.63582	down
-1.03114	down	2.63444	up
-1.12012	down	2.633486	up
-2.1861	down	2.633093	up
1.012646	up	-2.63294	down
-1.56942	down	-2.6329	down
3.142749	up	2.632856	up
1.279716	up	-2.63147	down
1.188919	up	2.630249	up
2.179322	up	-2.63018	down
-1.51884	down	2.629597	up
1.185955	up	2.629519	up
2.812371	up	-2.62914	down
1.612491	up	-2.62892	down
1.464443	up	2.627898	up
4.033225	up	2.626475	up
-1.04165	down	-2.62646	down
-1.40311	down	2.625592	up
4.39369	up	2.624313	up
1.184798	up	2.624141	up
4.555533	up	2.623542	up
1.178677	up	-2.62193	down
1.384026	up	2.621693	up
1.19126	up	2.621371	up
-1.14986	down	2.6209	up
-4.03775	down	2.620806	up
-1.93849	down	2.618638	up
-1.92216	down	2.618546	up
2.017464	up	-2.61803	down
-1.69486	down	2.617527	up
2.229405	up	2.617338	up
-1.92672	down	2.617234	up
-1.15603	down	-2.61686	down
-1.10963	down	2.616712	up

1.328422	up	2.616317	up
-1.8153	down	2.615133	up
1.061231	up	2.614262	up
-1.16694	down	2.613457	up
1.903949	up	-2.61178	down
-1.04906	down	-2.61024	down
1.16907	up	-2.60982	down
1.357154	up	2.60964	up
2.201568	up	2.609555	up
-1.85284	down	2.609284	up
10.76049	up	2.608739	up
-1.32787	down	-2.60872	down
2.969418	up	2.605789	up
-1.62501	down	-2.60513	down
-1.25896	down	2.604699	up
3.574678	up	2.604195	up
1.193377	up	-2.60357	down
1.11162	up	2.602453	up
1.394115	up	-2.60196	down
-1.73681	down	-2.60081	down
10.19353	up	2.600326	up
5.080843	up	2.600195	up
-1.49618	down	2.599399	up
1.589586	up	2.598091	up
1.076089	up	-2.59715	down
1.739529	up	2.596735	up
-1.46411	down	2.595556	up
1.156821	up	2.595109	up
1.189208	up	2.593462	up
1.315168	up	-2.59131	down
1.826589	up	-2.59012	down
-1.26864	down	2.590101	up
-1.01022	down	-2.58995	down
-2.21623	down	2.589887	up
-1.0426	down	2.589515	up
-2.25342	down	-2.58863	down
-1.09659	down	-2.58744	down
-2.97427	down	2.586781	up
1.608215	up	2.585418	up
2.170652	up	2.585362	up
1.234793	up	2.582829	up
3.349082	up	2.581882	up
-2.01983	down	2.580329	up
-1.27833	down	2.580002	up
1.023653	up	2.579974	up
-1.81916	down	-2.57957	down
1.186867	up	2.578043	up

-1.5659	down	2.577815	up
-2.57658	down	2.576942	up
1.976287	up	2.576811	up
1.09252	up	-2.57655	down
1.010719	up	2.57536	up
1.265425	up	-2.57306	down
-1.03186	down	-2.57191	down
-1.13976	down	-2.57069	down
1.624383	up	2.568788	up
1.805825	up	2.568466	up
-1.07906	down	-2.56841	down
1.752728	up	2.568228	up
-1.33189	down	2.567994	up
1.058951	up	2.567387	up
2.018136	up	2.56711	up
-1.18956	down	2.566739	up
1.165281	up	-2.56622	down
-3.73848	down	2.565219	up
1.140819	up	2.564632	up
-1.06391	down	-2.56406	down
-1.6326	down	2.563345	up
2.278338	up	2.562387	up
-1.49405	down	2.561928	up
1.173349	up	2.561642	up
-1.05019	down	-2.56129	down
1.971017	up	2.561208	up
1.567626	up	2.559723	up
-1.03053	down	2.558583	up
1.133048	up	-2.558	down
1.656265	up	2.556996	up
1.382979	up	2.556602	up
1.01378	up	2.556024	up
-1.14402	down	-2.5556	down
-1.49471	down	-2.55478	down
-2.95039	down	2.553988	up
1.484823	up	-2.55346	down
1.2765	up	2.552396	up
1.70224	up	-2.55194	down
1.234394	up	-2.55157	down
-1.16384	down	2.551388	up
1.473793	up	-2.55126	down
1.42822	up	-2.55108	down
-16.7366	down	2.550377	up
2.779547	up	2.550185	up
-1.48309	down	2.550021	up
-1.76247	down	2.54964	up
-1.80043	down	-2.54931	down

-1.72546	down	-2.54722	down
-1.27598	down	-2.54603	down
1.236179	up	2.545851	up
1.294159	up	-2.5458	down
1.251219	up	2.545209	up
-1.87372	down	2.543223	up
-1.1378	down	-2.54233	down
-1.24205	down	-2.54084	down
1.055135	up	2.540512	up
1.504408	up	-2.53898	down
1.807661	up	2.538443	up
1.12378	up	-2.53711	down
-1.61043	down	-2.53702	down
2.102346	up	2.536948	up
6.494872	up	2.536872	up
1.736152	up	2.536785	up
-1.08622	down	2.53577	up
1.018599	up	2.535481	up
-1.6769	down	2.535007	up
1.16055	up	-2.53499	down
8.856831	up	2.53485	up
1.111422	up	-2.53476	down
1.169611	up	-2.53474	down
1.14853	up	-2.53421	down
1.361586	up	-2.53369	down
-1.3769	down	-2.53257	down
-2.7275	down	2.531196	up
-1.34532	down	2.529892	up
-1.49335	down	-2.52949	down
1.020063	up	-2.52867	down
-1.48582	down	2.528173	up
-1.15671	down	2.527213	up
-1.80793	down	-2.52624	down
-1.35136	down	2.525346	up
-1.44271	down	2.52511	up
1.709106	up	2.524744	up
-1.07066	down	-2.52447	down
1.090061	up	2.52392	up
1.16099	up	-2.52326	down
-1.33542	down	-2.52279	down
-1.49412	down	2.521177	up
1.149551	up	2.51988	up
1.628543	up	2.519609	up
-1.64174	down	2.519553	up
5.877354	up	2.519355	up
1.668167	up	2.518791	up
2.125418	up	2.518427	up

-1.30118	down	-2.51783	down
-1.06935	down	-2.51749	down
-1.77611	down	2.515855	up
-1.17525	down	-2.51578	down
1.983547	up	-2.51527	down
-1.14587	down	2.513943	up
-3.73213	down	2.513163	up
1.574518	up	-2.513	down
-3.54815	down	2.512832	up
-1.79439	down	2.512305	up
3.46067	up	2.511947	up
-1.07884	down	-2.51123	down
1.854827	up	2.509928	up
2.294312	up	-2.50962	down
1.420573	up	-2.50956	down
1.916663	up	-2.50948	down
-22.4377	down	2.50943	up
1.47186	up	-2.50943	down
-1.00349	down	2.509414	up
1.350579	up	2.509397	up
1.365898	up	-2.50756	down
-1.20253	down	2.507339	up
1.217735	up	2.506849	up
-1.08445	down	2.506733	up
-1.07645	down	2.505055	up
-1.56329	down	2.505022	up
1.668552	up	-2.50434	down
-1.2704	down	-2.50375	down
2.36303	up	2.503395	up
1.381964	up	2.501266	up
-1.79095	down	-2.501	down
-1.2833	down	2.500859	up
1.391026	up	-2.50048	down
-1.08635	down	2.500279	up
1.054759	up	-2.49996	down
1.484456	up	2.499618	up
1.168451	up	2.498854	up
1.026382	up	2.498835	up
1.497234	up	2.498366	up
2.114839	up	-2.49822	down
-1.21407	down	-2.49809	down
-1.18485	down	2.497091	up
1.439643	up	2.496647	up
-1.5022	down	2.496643	up
-1.05166	down	-2.49523	down
1.219189	up	2.494961	up
1.048701	up	-2.49493	down

-1.21009	down	-2.49405	down
-1.34648	down	-2.49241	down
1.000881	up	2.491273	up
12.89685	up	2.491211	up
1.79083	up	-2.49079	down
1.639841	up	2.490612	up
1.60905	up	-2.4906	down
1.601637	up	2.489521	up
-1.05735	down	-2.48922	down
1.317708	up	-2.4888	down
-1.15301	down	2.4884	up
-1.69501	down	2.488179	up
-1.59262	down	-2.48536	down
1.665698	up	-2.48529	down
-1.34963	down	-2.48491	down
1.575416	up	2.484628	up
1.04711	up	-2.48423	down
-1.46512	down	2.484026	up
-1.17579	down	2.483196	up
1.057508	up	2.482703	up
-1.61325	down	2.482668	up
-6.15307	down	2.482547	up
-1.04131	down	2.481086	up
3.103741	up	-2.48104	down
-1.20341	down	-2.48035	down
-1.09715	down	2.479787	up
1.615416	up	2.479769	up
-1.3492	down	2.479422	up
1.490221	up	2.478469	up
1.316839	up	-2.47842	down
-2.01765	down	-2.47778	down
-3.03553	down	2.47774	up
1.080482	up	-2.47749	down
-1.20518	down	2.477351	up
-1.31315	down	2.476511	up
-1.2044	down	-2.47565	down
-1.1716	down	2.475035	up
-1.14802	down	-2.47486	down
4.011366	up	2.474397	up
-1.25823	down	-2.47297	down
1.153345	up	2.472728	up
-1.28131	down	2.471301	up
-1.78058	down	-2.47118	down
1.823216	up	2.471031	up
-2.35922	down	2.470055	up
1.357687	up	2.469704	up
1.931171	up	2.467267	up

-5.09629	down	2.466946	up
1.821424	up	2.464671	up
1.546189	up	2.464658	up
1.073238	up	-2.46437	down
-1.41791	down	-2.4626	down
-1.56503	down	-2.46197	down
-1.02428	down	2.461922	up
-1.08051	down	2.460447	up
1.874444	up	2.459764	up
-1.2762	down	-2.45862	down
1.22193	up	2.457073	up
1.459245	up	-2.45676	down
-1.08907	down	2.456359	up
2.210575	up	2.456118	up
-1.31834	down	-2.45572	down
1.409622	up	-2.45548	down
-1.10452	down	-2.45543	down
1.256844	up	-2.45447	down
1.539632	up	2.452856	up
-1.01896	down	-2.45261	down
-1.5382	down	-2.45221	down
-1.27074	down	-2.45219	down
1.156666	up	-2.45215	down
1.921063	up	-2.4518	down
-1.2254	down	2.451714	up
-1.74657	down	-2.45115	down
-1.29487	down	2.450652	up
2.261648	up	2.450353	up
-1.73753	down	2.449934	up
2.155727	up	2.448215	up
1.263904	up	-2.44783	down
1.324488	up	2.447532	up
1.470691	up	-2.44614	down
4.051497	up	2.443784	up
-1.18074	down	-2.44354	down
1.462211	up	2.442216	up
2.174366	up	2.441897	up
1.559503	up	2.441332	up
-1.08559	down	-2.44022	down
1.667751	up	2.438915	up
-2.42613	down	2.438899	up
1.376889	up	2.438502	up
-1.13878	down	-2.43811	down
1.916745	up	2.438013	up
1.109527	up	2.437233	up
-1.29411	down	2.436149	up
1.159217	up	-2.43467	down

1.220721	up	-2.434	down
-1.40552	down	-2.43314	down
1.019406	up	-2.43272	down
1.050596	up	2.431383	up
2.22199	up	2.429663	up
-2.26607	down	-2.42721	down
-1.15864	down	2.427174	up
-1.38701	down	2.427155	up
-2.65051	down	-2.42635	down
1.641468	up	2.426312	up
-1.00015	down	-2.42582	down
3.035257	up	-2.42494	down
1.493948	up	2.424765	up
9.267331	up	2.424639	up
-3.15712	down	2.424472	up
-1.23433	down	-2.42447	down
1.153447	up	-2.42399	down
2.40194	up	2.422979	up
-1.5865	down	2.422081	up
1.862768	up	-2.42202	down
4.227061	up	2.419833	up
1.073822	up	-2.41968	down
-1.48149	down	-2.41951	down
-1.05596	down	2.419491	up
-1.20326	down	-2.41886	down
-2.14858	down	2.418722	up
-1.01266	down	-2.41834	down
1.022022	up	2.418257	up
-1.24845	down	-2.41819	down
-1.20609	down	-2.41804	down
1.17337	up	-2.41768	down
-1.03269	down	2.417661	up
1.140203	up	-2.41759	down
2.476999	up	-2.41755	down
-1.81379	down	2.416672	up
1.833599	up	2.416066	up
-1.0533	down	-2.41566	down
-1.35313	down	2.414779	up
-1.81174	down	-2.41476	down
-1.00046	down	2.414594	up
-1.68246	down	2.414168	up
-2.34773	down	2.414054	up
1.318991	up	-2.4132	down
-1.52074	down	2.411598	up
-1.24394	down	-2.41136	down
1.098832	up	-2.41124	down
1.07005	up	-2.41123	down

3.223946	up	2.411035	up
-1.1079	down	2.410406	up
2.147756	up	2.410042	up
1.338552	up	2.408662	up
-1.3715	down	2.4067	up
1.132055	up	-2.4065	down
-1.29522	down	-2.4061	down
1.400003	up	-2.40606	down
1.410198	up	-2.40591	down
1.436391	up	-2.40483	down
-1.75049	down	-2.40472	down
-1.10398	down	-2.4047	down
1.310151	up	2.402919	up
-1.35073	down	-2.40233	down
1.298368	up	-2.40231	down
-1.0709	down	-2.4023	down
4.043847	up	2.398693	up
1.417912	up	-2.39828	down
-1.22819	down	-2.39812	down
-1.08124	down	-2.39766	down
-1.05673	down	-2.39695	down
1.073795	up	2.396498	up
1.053492	up	-2.39629	down
2.312356	up	2.394738	up
-1.32145	down	2.393007	up
1.16222	up	2.392368	up
1.344859	up	2.391496	up
1.01378	up	-2.39118	down
3.38725	up	2.390527	up
1.397346	up	2.390096	up
-1.40699	down	2.390094	up
-1.29664	down	2.389459	up
1.282946	up	-2.38889	down
-1.13761	down	-2.38859	down
-1.75208	down	2.388487	up
1.395142	up	2.388111	up
-2.20859	down	2.387971	up
-1.11205	down	2.387797	up
2.961973	up	2.386705	up
1.997788	up	-2.38637	down
-1.24558	down	2.386319	up
1.833817	up	-2.38621	down
1.301883	up	-2.38573	down
-5.1331	down	2.385599	up
1.167079	up	-2.38533	down
-1.03853	down	-2.38478	down
-1.02545	down	2.383894	up

-2.63384	down	2.381365	up
1.417448	up	2.380204	up
1.414342	up	2.379523	up
1.123556	up	2.379355	up
-1.12953	down	-2.37813	down
-1.4572	down	-2.37793	down
2.090522	up	2.377376	up
2.17624	up	2.37719	up
2.044649	up	2.376046	up
-2.72405	down	-2.37501	down
1.10389	up	2.374217	up
2.829456	up	-2.37322	down
-2.28051	down	2.372845	up
1.186149	up	2.372737	up
-1.02166	down	2.372629	up
-1.45617	down	2.372483	up
-1.15649	down	2.372265	up
2.460497	up	2.371292	up
1.175015	up	-2.37113	down
1.750163	up	2.371021	up
1.31417	up	2.37035	up
1.495276	up	-2.36996	down
2.037086	up	2.369929	up
2.145647	up	2.369861	up
-2.12943	down	-2.36965	down
-2.41191	down	-2.36959	down
1.359458	up	-2.36949	down
-1.12628	down	2.368681	up
1.800166	up	-2.36829	down
-1.55116	down	-2.36816	down
1.022668	up	-2.36812	down
1.01008	up	2.367405	up
-1.05731	down	-2.36715	down
1.600916	up	2.366634	up
1.701008	up	2.36663	up
1.03797	up	-2.36649	down
1.313412	up	2.365266	up
1.774059	up	2.364401	up
-1.19034	down	2.363811	up
-2.30006	down	-2.36344	down
1.192841	up	-2.36214	down
-1.24072	down	-2.36061	down
-1.34478	down	-2.3603	down
1.362047	up	-2.3599	down
-1.01104	down	-2.35922	down
1.244619	up	2.358871	up
-1.0259	down	-2.35796	down

-2.53304	down	2.357088	up
2.256026	up	2.35592	up
-1.06159	down	2.355777	up
1.218329	up	2.355573	up
-1.71123	down	2.355378	up
1.117159	up	2.355228	up
-1.69638	down	2.354619	up
-1.69205	down	2.354416	up
2.497435	up	2.354262	up
1.329634	up	-2.35419	down
-6.83054	down	2.353862	up
2.018586	up	2.353301	up
1.217615	up	-2.35251	down
-1.64885	down	2.351657	up
-1.18981	down	2.351193	up
1.44582	up	2.350952	up
-1.02527	down	-2.35084	down
-1.03604	down	2.350675	up
1.53529	up	2.349759	up
1.155754	up	-2.3496	down
1.056167	up	2.349173	up
1.070984	up	-2.34843	down
-1.34741	down	-2.34816	down
1.073383	up	2.346828	up
-1.0219	down	2.34529	up
1.704905	up	2.345001	up
1.251481	up	-2.34495	down
-1.8049	down	2.34394	up
1.897462	up	2.343485	up
-1.14144	down	-2.34292	down
2.168049	up	2.34172	up
-1.06489	down	-2.34082	down
1.063568	up	-2.33959	down
1.124409	up	-2.33922	down
-1.1049	down	2.339105	up
-1.13189	down	2.338404	up
1.020468	up	2.338101	up
-1.16301	down	2.337819	up
1.778082	up	2.337357	up
1.411061	up	-2.33703	down
1.155002	up	2.336568	up
-1.64324	down	2.335953	up
-1.69525	down	2.335537	up
1.077427	up	2.335519	up
3.997992	up	2.33541	up
1.810817	up	-2.33492	down
1.246637	up	2.334573	up

1.04118	up	2.333513	up
-1.01338	down	2.333513	up
-1.81054	down	2.332745	up
1.292602	up	2.332433	up
2.102698	up	2.332129	up
1.899326	up	2.331974	up
2.494859	up	2.331737	up
2.723065	up	-2.33071	down
1.523545	up	2.330455	up
-1.73582	down	-2.32969	down
-1.06037	down	2.32963	up
-1.55333	down	2.32946	up
-1.74516	down	2.327615	up
-1.01799	down	-2.3272	down
1.59751	up	2.327053	up
1.261186	up	-2.32675	down
1.295857	up	-2.32627	down
1.016594	up	2.325674	up
1.392079	up	2.325579	up
-1.79526	down	2.325061	up
-1.07035	down	-2.32491	down
1.424308	up	2.324771	up
1.002038	up	-2.32469	down
1.21274	up	-2.32413	down
1.871857	up	2.323718	up
1.223326	up	2.323079	up
-1.09316	down	-2.32175	down
1.029482	up	-2.32174	down
-1.27604	down	2.321049	up
-1.20937	down	2.320356	up
1.061963	up	2.319873	up
1.112621	up	-2.3192	down
-3.12738	down	2.318387	up
-1.17055	down	-2.31813	down
1.626978	up	-2.31803	down
1.017303	up	-2.31728	down
1.344344	up	-2.31697	down
1.390342	up	2.31693	up
-1.38027	down	-2.31671	down
-1.59126	down	2.315906	up
1.591769	up	-2.31556	down
-1.19228	down	-2.31532	down
-1.20072	down	2.314991	up
-1.08519	down	-2.31464	down
1.149672	up	2.314317	up
1.112779	up	-2.31415	down
-1.31607	down	2.313568	up

1.451953	up	-2.31349	down
-1.27381	down	2.31254	up
1.063898	up	-2.31141	down
1.500437	up	-2.31132	down
1.183896	up	-2.31129	down
-1.03807	down	2.311287	up
1.240561	up	-2.31109	down
-1.66794	down	-2.31095	down
-1.52631	down	2.310776	up
1.088248	up	-2.31063	down
1.33338	up	2.310242	up
1.769533	up	-2.31002	down
1.067143	up	2.30906	up
1.044586	up	2.308909	up
1.16223	up	-2.3088	down
1.741177	up	2.308688	up
1.36751	up	-2.30859	down
-1.65148	down	-2.30734	down
-1.8112	down	2.305851	up
-1.33409	down	-2.30544	down
1.541221	up	2.304072	up
-3.73717	down	-2.30283	down
-1.38979	down	2.302428	up
-2.57475	down	2.301785	up
1.686439	up	2.300387	up
1.328657	up	2.299468	up
1.677585	up	2.299262	up
1.180641	up	-2.29757	down
1.802493	up	2.297469	up
-1.0243	down	2.29716	up
1.839329	up	-2.29709	down
-1.08806	down	2.297046	up
1.780843	up	2.297011	up
-1.6654	down	-2.29629	down
1.171437	up	2.296017	up
-1.40474	down	2.295821	up
-1.10211	down	2.294919	up
1.400043	up	-2.29411	down
-1.24149	down	2.294113	up
-1.21905	down	-2.29317	down
-1.31138	down	-2.29313	down
1.208566	up	2.293114	up
-4.19987	down	2.292591	up
-1.33114	down	2.292227	up
-1.25684	down	-2.29125	down
3.265112	up	2.291112	up
-1.37678	down	2.291049	up

-1.37422	down	-2.29099	down
1.417293	up	2.290482	up
-1.0295	down	2.289928	up
-1.56464	down	2.289004	up
2.676163	up	2.288227	up
1.241016	up	2.288057	up
1.040055	up	-2.28709	down
-1.16436	down	-2.28664	down
-1.30032	down	2.286557	up
1.464887	up	2.285047	up
-1.62759	down	2.284355	up
-1.01973	down	2.283951	up
1.418744	up	-2.28383	down
1.738143	up	-2.28319	down
-1.07694	down	2.283163	up
-1.5328	down	-2.28313	down
1.612551	up	2.282387	up
-1.40387	down	-2.28172	down
-1.07836	down	-2.28116	down
-1.03201	down	2.280912	up
1.238483	up	-2.28058	down
1.231696	up	-2.28042	down
-1.97696	down	-2.28038	down
-1.44833	down	-2.28034	down
1.082625	up	2.280034	up
1.151017	up	2.279945	up
1.128493	up	2.279574	up
-1.27467	down	-2.27829	down
3.626641	up	2.27812	up
1.209815	up	-2.27731	down
1.133766	up	-2.27725	down
1.607416	up	2.276861	up
1.491246	up	2.276715	up
1.639859	up	-2.27661	down
-1.25256	down	-2.27654	down
-1.16631	down	2.276125	up
-2.00915	down	2.276074	up
1.048175	up	-2.27558	down
-1.64097	down	-2.27507	down
-1.49795	down	-2.27503	down
1.372643	up	-2.27358	down
-1.88106	down	-2.27256	down
1.85776	up	-2.27255	down
1.468072	up	2.271772	up
2.509265	up	2.271736	up
1.674532	up	2.270459	up
4.156447	up	2.270279	up

1.072974	up	-2.26952	down
1.266175	up	-2.26885	down
1.016393	up	-2.26819	down
-2.69521	down	-2.26794	down
1.147307	up	-2.26755	down
-1.47152	down	-2.26749	down
-1.05381	down	-2.2661	down
-1.52327	down	2.265567	up
3.277093	up	-2.2649	down
-1.54515	down	-2.26481	down
-1.28058	down	-2.26462	down
-1.65978	down	2.264038	up
-1.82142	down	2.263774	up
-1.39058	down	-2.26356	down
-1.13836	down	-2.26227	down
-1.5152	down	-2.26217	down
1.067179	up	2.26181	up
3.017158	up	-2.2613	down
1.136978	up	2.261101	up
-1.91463	down	-2.26088	down
-1.58006	down	2.260441	up
1.946904	up	2.260165	up
-1.48647	down	-2.25903	down
1.211899	up	2.258965	up
1.496229	up	2.25831	up
1.077981	up	-2.25781	down
1.760411	up	2.257775	up
-1.3945	down	2.257504	up
-1.0178	down	-2.2567	down
1.496696	up	2.256399	up
2.248601	up	2.255918	up
1.158409	up	-2.25528	down
3.11083	up	-2.25466	down
1.643498	up	2.254356	up
-1.34477	down	-2.25409	down
-1.27673	down	-2.25318	down
-1.21324	down	2.252253	up
1.767619	up	2.251918	up
1.243517	up	-2.25175	down
1.457067	up	-2.2498	down
3.491304	up	2.249599	up
-1.60003	down	2.249197	up
1.243831	up	-2.24919	down
-3.3165	down	2.248537	up
2.48473	up	2.248011	up
1.638501	up	2.247788	up
1.134315	up	-2.24677	down

1.201555	up	-2.24513	down
1.472339	up	-2.2441	down
1.209179	up	2.243475	up
1.0209	up	2.243473	up
1.058494	up	2.243416	up
1.902361	up	2.243263	up
-1.23553	down	2.242572	up
-1.55665	down	2.241854	up
1.16573	up	2.241768	up
1.954638	up	-2.23795	down
-1.96345	down	2.236942	up
1.029001	up	-2.23688	down
1.201142	up	-2.23659	down
1.215787	up	2.236188	up
1.261244	up	-2.23599	down
-1.07637	down	-2.23599	down
1.364566	up	-2.23552	down
-1.35704	down	-2.23492	down
1.521219	up	2.234874	up
1.242845	up	2.234807	up
1.156783	up	2.234619	up
1.263866	up	2.234412	up
-1.33426	down	2.233952	up
-1.04745	down	-2.23298	down
-1.0322	down	2.232733	up
1.096294	up	2.232489	up
-1.14047	down	-2.23236	down
1.574651	up	2.232025	up
-1.71838	down	2.231577	up
-1.48879	down	2.230609	up
-1.03079	down	-2.22944	down
1.104591	up	-2.22922	down
1.112832	up	-2.22886	down
1.38288	up	2.228821	up
2.707649	up	2.228194	up
-1.29371	down	-2.22724	down
-1.71375	down	-2.22693	down
1.043501	up	-2.22674	down
1.012921	up	-2.22644	down
-1.45079	down	-2.22493	down
-1.25716	down	2.223444	up
-1.12922	down	2.222997	up
-1.76338	down	2.222928	up
-3.00189	down	2.222239	up
-1.25964	down	2.221845	up
1.0537	up	-2.22166	down
1.591437	up	2.221639	up

-1.01559	down	-2.2205	down
-2.14544	down	2.220349	up
1.034266	up	-2.2202	down
-1.01677	down	-2.22013	down
-1.07194	down	-2.2197	down
-1.35336	down	2.219435	up
1.26489	up	-2.21806	down
-1.25093	down	-2.21697	down
-1.55373	down	2.215837	up
1.091714	up	-2.21552	down
1.223493	up	-2.21518	down
1.484867	up	-2.21508	down
-1.14448	down	2.214906	up
1.385784	up	-2.2147	down
-1.73745	down	2.212541	up
1.68309	up	-2.21252	down
-1.82885	down	-2.21197	down
-1.47337	down	-2.21175	down
-1.9472	down	2.211696	up
-4.10761	down	2.211585	up
1.083554	up	-2.21148	down
1.352795	up	-2.21118	down
1.987824	up	2.211116	up
2.587964	up	2.210777	up
1.77831	up	2.210466	up
2.588246	up	2.209904	up
-1.31917	down	-2.20981	down
1.155468	up	-2.20974	down
1.402314	up	2.209636	up
3.073919	up	2.209535	up
1.049876	up	-2.20941	down
-1.42862	down	-2.20921	down
1.707848	up	2.2091	up
-1.91009	down	-2.20902	down
1.211049	up	2.208313	up
-1.13146	down	-2.20757	down
1.228855	up	2.20727	up
-1.05456	down	2.207014	up
-1.51974	down	2.206206	up
1.491117	up	-2.20527	down
-1.59554	down	-2.20475	down
-1.11413	down	-2.20449	down
-5.47953	down	2.203617	up
2.991546	up	-2.20283	down
-1.50038	down	-2.20255	down
1.877071	up	-2.20201	down
-1.47029	down	2.201663	up

-1.53792	down	2.201045	up
-1.20599	down	2.200715	up
1.030604	up	-2.20065	down
1.102629	up	2.199759	up
-1.2849	down	-2.19899	down
1.562881	up	-2.19888	down
1.391271	up	2.19879	up
1.507151	up	-2.19815	down
-1.269	down	-2.19812	down
1.262433	up	2.197604	up
1.131254	up	-2.19703	down
-1.44104	down	2.196163	up
-1.06997	down	-2.19549	down
2.443142	up	2.195388	up
-1.69746	down	2.193993	up
-1.82391	down	-2.19355	down
1.231319	up	2.193112	up
1.449584	up	-2.19299	down
-1.21647	down	-2.19284	down
1.569964	up	-2.19279	down
-1.01721	down	-2.19261	down
1.311998	up	-2.19246	down
-4.37653	down	-2.19192	down
-2.02868	down	-2.1918	down
-1.62239	down	-2.19149	down
2.471288	up	-2.19135	down
-1.08969	down	-2.19105	down
1.008819	up	-2.19067	down
-1.27083	down	-2.18979	down
-1.14374	down	-2.1895	down
-1.17401	down	2.189389	up
1.02306	up	-2.18928	down
1.805901	up	2.189145	up
-1.43611	down	2.18879	up
1.219687	up	2.188271	up
-1.55737	down	-2.18661	down
1.391255	up	2.186444	up
-1.56319	down	2.186093	up
1.042481	up	-2.18555	down
-1.52913	down	-2.18539	down
1.117444	up	2.184111	up
1.852989	up	-2.18403	down
2.187479	up	2.183518	up
1.493806	up	2.183284	up
-1.37929	down	-2.18288	down
1.31861	up	2.182795	up
2.296731	up	2.182499	up

1.366192	up	-2.18237	down
-1.49031	down	2.182361	up
-1.3464	down	-2.182	down
-1.01958	down	-2.18187	down
-1.41178	down	-2.18134	down
1.162436	up	2.180936	up
-1.09828	down	-2.18064	down
1.337727	up	2.180441	up
-1.66146	down	-2.17973	down
-1.2586	down	2.178876	up
-1.14943	down	2.178153	up
-1.04702	down	-2.17754	down
3.58053	up	2.177277	up
1.627275	up	-2.17654	down
1.49041	up	-2.17602	down
-1.05094	down	2.175043	up
-1.36109	down	2.174685	up
1.081188	up	-2.17398	down
1.300164	up	2.173725	up
-1.01084	down	2.172908	up
3.207091	up	2.172516	up
-1.27823	down	-2.17242	down
1.126214	up	2.172278	up
-2.93663	down	2.171395	up
-1.21537	down	2.17126	up
-1.60055	down	-2.17116	down
1.082909	up	-2.17071	down
1.677481	up	-2.17063	down
-1.83917	down	2.17061	up
-3.3964	down	2.170564	up
-2.32103	down	2.170149	up
-1.27705	down	-2.16938	down
-1.27915	down	-2.16932	down
1.244962	up	-2.16783	down
1.372663	up	2.167247	up
1.521428	up	-2.16677	down
-1.03959	down	-2.1663	down
1.101975	up	2.166256	up
-1.68678	down	-2.16525	down
3.435969	up	2.16429	up
-1.17422	down	-2.16404	down
-1.55429	down	-2.16379	down
-1.24788	down	-2.16368	down
1.318151	up	-2.16315	down
1.423609	up	2.16261	up
-1.64491	down	2.161143	up
-1.29936	down	-2.1606	down

1.755998	up	2.160369	up
1.840065	up	-2.15939	down
-1.14806	down	-2.15798	down
-1.36943	down	2.157756	up
-1.35638	down	-2.15744	down
-1.36779	down	-2.15646	down
-1.12512	down	2.156093	up
-1.21501	down	2.155884	up
1.743158	up	-2.15561	down
-1.06238	down	2.155075	up
1.61726	up	-2.15507	down
1.556289	up	-2.15506	down
1.935295	up	2.154882	up
-1.03352	down	2.154854	up
1.393917	up	2.154719	up
-1.27143	down	2.154573	up
-2.34896	down	-2.15441	down
-1.29479	down	2.15432	up
-1.14463	down	-2.15329	down
-1.25389	down	-2.15316	down
-1.25278	down	2.152893	up
1.231629	up	-2.15244	down
-1.31228	down	-2.15221	down
2.085493	up	-2.15185	down
-1.0887	down	2.151784	up
2.02147	up	2.151495	up
-1.30904	down	2.151472	up
2.194364	up	2.151339	up
2.040714	up	2.151276	up
-1.26845	down	-2.15098	down
-1.1573	down	-2.15072	down
-2.08121	down	-2.15054	down
1.155639	up	-2.15048	down
1.194454	up	-2.15017	down
1.21689	up	2.150082	up
-2.05049	down	-2.14981	down
-1.08573	down	-2.14919	down
1.270907	up	-2.14872	down
-2.40951	down	2.148538	up
1.074916	up	2.148209	up
-1.27428	down	2.147985	up
-1.61228	down	-2.14794	down
-1.43614	down	-2.1478	down
2.189915	up	-2.14751	down
-3.52097	down	2.147474	up
-1.24965	down	-2.14746	down
-1.1922	down	2.147145	up

-1.36182	down	2.147064	up
-1.82707	down	-2.14691	down
1.38612	up	-2.1465	down
-2.08946	down	2.146244	up
2.691339	up	2.14611	up
1.308718	up	-2.14606	down
1.351419	up	2.145885	up
1.129021	up	-2.14546	down
1.782809	up	-2.14486	down
-1.27733	down	2.144373	up
1.133008	up	-2.14424	down
-1.2113	down	-2.14417	down
1.690214	up	2.143702	up
5.198775	up	2.143411	up
-1.88203	down	-2.14333	down
1.267716	up	2.143148	up
-1.3957	down	-2.14245	down
1.245782	up	2.142198	up
-1.07444	down	-2.1416	down
-1.13088	down	-2.14125	down
2.528411	up	-2.14073	down
1.268864	up	2.140337	up
-1.28875	down	-2.14029	down
-1.02699	down	2.140036	up
1.301329	up	-2.13995	down
-1.31828	down	-2.13972	down
-1.71751	down	-2.13948	down
1.193061	up	2.139452	up
1.762451	up	2.139063	up
-1.5598	down	2.138412	up
1.093435	up	2.138362	up
-1.23307	down	2.137918	up
-3.34884	down	2.137501	up
-1.30941	down	2.137444	up
-1.86752	down	-2.13722	down
2.43711	up	2.137187	up
-1.41253	down	-2.13681	down
1.085303	up	2.136423	up
1.101391	up	-2.1364	down
1.352071	up	2.136303	up
1.003255	up	2.136267	up
-1.11799	down	2.136097	up
1.214982	up	-2.136	down
1.096399	up	2.135048	up
-1.55338	down	2.134976	up
1.269526	up	2.134427	up
-1.39197	down	-2.13376	down

-1.44379	down	-2.13373	down
1.088171	up	-2.13371	down
-2.08844	down	-2.13313	down
-1.06365	down	2.133112	up
-1.11362	down	2.132651	up
1.234099	up	2.131632	up
-1.49415	down	-2.1316	down
1.129117	up	2.131573	up
-1.41589	down	2.131405	up
1.007577	up	-2.13139	down
1.024243	up	2.130909	up
1.72439	up	-2.1305	down
1.068816	up	2.130022	up
-1.20134	down	-2.12953	down
-2.16566	down	-2.12952	down
-1.2765	down	-2.12936	down
-1.33328	down	-2.12894	down
1.336146	up	2.128712	up
2.470156	up	2.128622	up
-1.52587	down	-2.12847	down
1.210734	up	2.127974	up
-2.08608	down	2.127346	up
-1.274	down	2.126914	up
1.098699	up	-2.12674	down
-1.1503	down	-2.12667	down
6.317597	up	-2.12656	down
1.053683	up	-2.12582	down
1.025032	up	2.125795	up
-1.14065	down	-2.12567	down
-1.06478	down	-2.12561	down
1.164336	up	-2.12543	down
1.770453	up	-2.12475	down
-1.18889	down	2.1247	up
-1.08339	down	-2.12277	down
1.301916	up	-2.12234	down
-1.3683	down	-2.12163	down
1.059975	up	-2.12123	down
-1.38324	down	2.121133	up
1.080774	up	-2.12102	down
-1.56997	down	2.120214	up
-1.02105	down	2.120154	up
-1.20617	down	-2.12012	down
1.332472	up	-2.11925	down
-1.04451	down	2.119062	up
-1.76272	down	2.118827	up
-1.41228	down	2.118283	up
-2.34559	down	2.116745	up

1.380435	up	-2.11638	down
-1.08002	down	-2.11638	down
1.154962	up	2.11596	up
-1.15606	down	-2.11499	down
1.463013	up	2.114871	up
-1.04373	down	-2.11479	down
1.216451	up	2.114237	up
1.188477	up	-2.1142	down
2.001305	up	2.114178	up
9.26364	up	2.113833	up
-1.48124	down	-2.11346	down
-1.32755	down	-2.1132	down
1.344143	up	2.112948	up
-5.19095	down	2.112714	up
-1.36522	down	-2.1122	down
-1.63993	down	-2.11199	down
-1.04623	down	2.11111	up
-1.58922	down	-2.11104	down
-1.07767	down	-2.11092	down
1.037227	up	2.110797	up
-1.19245	down	2.110687	up
-1.65112	down	2.110385	up
1.19995	up	2.109336	up
-1.21088	down	2.108915	up
1.845422	up	2.108861	up
2.548363	up	2.108619	up
1.134727	up	2.108457	up
1.159382	up	2.108172	up
-1.4206	down	2.108047	up
-1.46239	down	-2.10757	down
1.160623	up	2.107293	up
2.412216	up	2.10717	up
-1.40219	down	-2.10705	down
1.060537	up	-2.10705	down
1.3105	up	2.106897	up
-2.87261	down	2.106377	up
-1.41853	down	2.106315	up
-1.11369	down	-2.10606	down
-1.57672	down	-2.10585	down
-1.51021	down	2.105678	up
-5.61052	down	2.105646	up
-1.02017	down	-2.1054	down
-1.10475	down	2.105172	up
1.060585	up	-2.10506	down
-1.8331	down	2.10421	up
-1.34977	down	-2.1042	down
2.352038	up	2.104039	up

-1.16064	down	-2.10398	down
1.502731	up	-2.1036	down
-1.45072	down	2.10357	up
1.148995	up	-2.10325	down
-1.40767	down	-2.10316	down
-1.34135	down	-2.10303	down
1.44966	up	-2.10267	down
-2.33574	down	2.102299	up
-1.22141	down	2.10121	up
-1.44433	down	2.101185	up
-1.31313	down	2.100982	up
2.789496	up	2.10096	up
-3.8268	down	2.100934	up
1.039326	up	2.100723	up
4.802067	up	2.100574	up
1.206043	up	-2.10052	down
2.160916	up	-2.09971	down
-1.92092	down	-2.09962	down
-2.04035	down	2.099393	up
1.648596	up	2.099044	up
1.834566	up	-2.09817	down
1.526591	up	2.097474	up
-1.93532	down	2.097422	up
-1.07404	down	2.096953	up
-1.48271	down	2.096696	up
1.002059	up	-2.09655	down
1.2116	up	-2.09606	down
-1.36564	down	-2.09585	down
1.384122	up	-2.09578	down
-1.7691	down	-2.09571	down
-1.41213	down	-2.09569	down
1.996539	up	2.095207	up
-1.03777	down	2.095103	up
-1.11318	down	2.093804	up
6.236457	up	2.093778	up
-2.5701	down	-2.09352	down
-1.06618	down	2.093281	up
-1.31307	down	-2.09317	down
1.697802	up	2.093162	up
1.119092	up	2.092774	up
1.140063	up	-2.09257	down
-2.78022	down	-2.0924	down
-2.33965	down	-2.09208	down
1.335456	up	2.091986	up
1.123695	up	-2.09185	down
1.375074	up	2.091485	up
2.063886	up	2.091367	up

-1.00631	down	2.090582	up
-1.03901	down	-2.09051	down
-1.45172	down	-2.09049	down
1.355021	up	2.090365	up
1.003618	up	2.090006	up
-1.28101	down	-2.08984	down
2.210714	up	2.08959	up
-1.89308	down	-2.08931	down
1.025313	up	-2.08915	down
2.50945	up	2.088699	up
1.32048	up	-2.08827	down
-1.25223	down	2.088217	up
-1.03028	down	-2.08806	down
-1.13827	down	-2.08786	down
-2.18085	down	2.087364	up
1.30825	up	2.086849	up
1.155193	up	-2.0858	down
1.878898	up	2.085419	up
-1.5969	down	-2.08535	down
-2.17615	down	2.084759	up
-1.45707	down	2.084562	up
-1.70309	down	2.08436	up
-1.24879	down	-2.08425	down
-1.08052	down	-2.08388	down
1.042699	up	-2.08304	down
1.078265	up	2.082996	up
-2.12903	down	2.082836	up
-1.10334	down	-2.08229	down
1.0438	up	-2.08228	down
-2.78239	down	-2.08195	down
-1.69591	down	-2.08173	down
-1.22392	down	2.081485	up
-1.56766	down	-2.08044	down
-2.18588	down	2.079981	up
5.119659	up	2.079978	up
1.252485	up	2.079625	up
-1.08448	down	-2.07956	down
-1.19723	down	-2.07943	down
-1.1469	down	-2.07924	down
-1.24173	down	2.079086	up
1.461024	up	-2.07843	down
-1.09135	down	2.078346	up
-2.11195	down	-2.07834	down
-1.08138	down	2.07803	up
-1.45438	down	-2.07714	down
1.011718	up	2.076229	up
1.386738	up	-2.07604	down

-1.00421	down	-2.07603	down
-1.52202	down	2.07546	up
-1.61006	down	2.074524	up
1.052521	up	-2.07375	down
-1.07249	down	2.073447	up
-1.12131	down	-2.07304	down
1.564562	up	-2.07287	down
1.204529	up	2.072703	up
-1.43664	down	-2.07258	down
1.30276	up	2.072443	up
1.815621	up	2.072317	up
1.565288	up	2.072223	up
-1.48234	down	2.072163	up
1.160238	up	-2.07192	down
1.170759	up	-2.07177	down
1.577807	up	-2.07173	down
-1.14927	down	2.071708	up
-1.08788	down	-2.07159	down
1.695958	up	2.070984	up
-1.13432	down	-2.07033	down
-1.66513	down	-2.06961	down
-3.11194	down	2.069373	up
-1.56615	down	-2.06751	down
2.174582	up	-2.06704	down
-1.20647	down	-2.06675	down
-1.08495	down	-2.06623	down
-1.65545	down	-2.06603	down
1.089009	up	2.065923	up
1.386716	up	2.06568	up
-1.23064	down	-2.06559	down
-1.05891	down	-2.06532	down
-1.17862	down	-2.06515	down
-4.69732	down	2.065021	up
1.441966	up	2.064998	up
-2.06279	down	-2.06476	down
-2.25795	down	2.064577	up
-1.6518	down	2.064341	up
-1.21082	down	-2.06418	down
-1.34339	down	-2.06387	down
1.07502	up	-2.06369	down
1.546652	up	2.063403	up
1.15939	up	2.063135	up
-1.17925	down	-2.06228	down
-1.12988	down	2.061897	up
-2.74741	down	2.061508	up
1.13391	up	-2.06115	down
-1.14955	down	-2.06101	down

1.287807	up	-2.06086	down
-1.5072	down	2.060083	up
-4.26125	down	2.059921	up
-1.44249	down	-2.05965	down
-1.28503	down	-2.05929	down
1.00709	up	2.058697	up
1.007378	up	-2.05864	down
-1.39389	down	2.058606	up
-1.22681	down	-2.05851	down
2.983429	up	2.058503	up
-1.5086	down	-2.05816	down
1.40909	up	-2.05764	down
1.41013	up	2.057253	up
1.114523	up	-2.05721	down
1.07329	up	-2.05702	down
1.271564	up	-2.0566	down
-1.17749	down	-2.05657	down
1.274434	up	-2.05631	down
-2.30347	down	2.055889	up
1.192359	up	-2.05563	down
1.558933	up	-2.05537	down
-2.6594	down	2.055245	up
2.000215	up	-2.05482	down
1.176463	up	2.054249	up
1.240153	up	2.054102	up
1.106003	up	-2.05401	down
1.240736	up	2.053975	up
1.031048	up	2.05392	up
-1.3022	down	2.053755	up
1.419499	up	2.053577	up
-1.39708	down	-2.05312	down
-2.84747	down	2.053008	up
-1.87868	down	-2.05298	down
-1.33758	down	-2.05259	down
2.34051	up	2.052303	up
1.227147	up	-2.05211	down
-1.25207	down	2.052014	up
-2.07767	down	-2.0513	down
1.986796	up	2.051249	up
-1.65675	down	-2.05098	down
-1.2598	down	-2.0508	down
1.26144	up	2.05043	up
-1.17406	down	2.049735	up
1.566157	up	2.049651	up
-1.33073	down	-2.04936	down
-1.44273	down	-2.0493	down
2.202172	up	-2.0493	down

1.230571	up	2.049126	up
-1.41651	down	-2.04861	down
1.094256	up	-2.04861	down
-1.01057	down	-2.04819	down
-1.37829	down	-2.04689	down
-1.03511	down	-2.04656	down
1.33941	up	-2.04624	down
1.31694	up	2.046216	up
-1.08861	down	-2.04591	down
1.027961	up	-2.04576	down
-1.09746	down	-2.0454	down
1.213131	up	2.045166	up
-1.31994	down	-2.04477	down
-1.13272	down	2.04474	up
22.08274	up	2.044647	up
1.025623	up	-2.04445	down
1.465813	up	-2.04428	down
1.110777	up	2.043813	up
-1.166	down	-2.04376	down
1.148464	up	-2.04278	down
1.384259	up	-2.04248	down
3.008417	up	2.041847	up
1.252655	up	-2.04127	down
1.653164	up	-2.0407	down
-1.24049	down	-2.04056	down
1.751414	up	2.039849	up
-2.03612	down	2.039057	up
-1.04404	down	-2.03903	down
-1.2967	down	2.039021	up
1.387449	up	2.039004	up
1.47551	up	-2.03896	down
-2.13485	down	-2.03894	down
1.129103	up	2.038802	up
1.062199	up	-2.03871	down
-1.14202	down	-2.03862	down
-1.08261	down	-2.03842	down
-1.1722	down	-2.03824	down
-1.48967	down	-2.03818	down
-1.30036	down	-2.03789	down
-1.17342	down	-2.03774	down
-3.57089	down	-2.03723	down
-1.25576	down	2.037074	up
1.543187	up	2.036701	up
1.134041	up	-2.03619	down
1.268722	up	2.03569	up
1.129739	up	-2.03566	down
1.36776	up	2.035429	up

1.401954	up	2.03529	up
1.412763	up	-2.03521	down
1.342731	up	2.034748	up
1.586873	up	-2.03471	down
1.209115	up	2.034275	up
-1.35846	down	-2.03421	down
-1.00932	down	-2.03403	down
1.021119	up	2.033892	up
-2.6249	down	2.033462	up
1.39366	up	-2.0334	down
-1.15862	down	-2.03305	down
1.602841	up	2.032633	up
-1.42637	down	2.03263	up
-1.19117	down	2.032471	up
-1.54429	down	-2.03224	down
-1.10667	down	-2.03179	down
1.202895	up	-2.03153	down
1.841969	up	2.031088	up
-1.16663	down	2.031	up
-1.20296	down	-2.03057	down
1.104556	up	-2.03052	down
-1.02745	down	-2.03044	down
-1.11234	down	2.030317	up
-1.27675	down	-2.02977	down
1.051692	up	-2.02942	down
-2.37057	down	-2.02929	down
1.221138	up	2.028699	up
1.381063	up	2.028429	up
1.193281	up	-2.02831	down
-1.38364	down	-2.02743	down
-1.05868	down	2.027228	up
-1.22822	down	2.027101	up
1.717489	up	2.026949	up
-2.71359	down	2.026297	up
-1.3949	down	2.025991	up
1.715566	up	-2.02526	down
-1.58384	down	-2.02498	down
2.328223	up	-2.02434	down
2.907049	up	2.024183	up
-3.38666	down	2.024163	up
1.364672	up	2.023894	up
-1.35227	down	2.023876	up
3.265883	up	-2.02376	down
1.710994	up	2.023386	up
-1.28142	down	2.023169	up
1.042999	up	2.022925	up
1.325204	up	-2.02279	down

-4.5701	down	2.02244	up
-1.27868	down	2.021091	up
1.524507	up	-2.02088	down
-1.02358	down	-2.02047	down
-1.54717	down	2.02021	up
-1.19953	down	-2.01978	down
2.123857	up	2.019276	up
2.099068	up	-2.01914	down
1.105422	up	-2.01906	down
1.202139	up	2.019002	up
1.222606	up	-2.01897	down
-1.08358	down	-2.01863	down
1.296774	up	-2.01853	down
-1.17764	down	-2.01849	down
1.046594	up	2.018314	up
-1.55102	down	2.018215	up
1.291473	up	2.018094	up
-1.13079	down	-2.01803	down
-1.23718	down	2.017086	up
1.26416	up	2.017074	up
-1.20733	down	2.017042	up
-1.55776	down	2.016387	up
1.660482	up	2.016018	up
-1.10472	down	2.015903	up
1.158958	up	-2.01496	down
-1.63514	down	-2.01459	down
-1.25137	down	2.013503	up
1.503332	up	-2.01342	down
-2.24364	down	2.012899	up
-1.23575	down	-2.01281	down
1.306797	up	2.012734	up
1.224529	up	-2.01248	down
-1.92644	down	-2.01244	down
2.507667	up	2.012167	up
-4.66271	down	2.012111	up
-1.14376	down	2.011541	up
-2.30137	down	2.011202	up
2.276132	up	-2.01107	down
2.604199	up	2.010999	up
-1.28205	down	-2.01018	down
1.241	up	2.009492	up
-1.29219	down	-2.00945	down
1.723041	up	2.009395	up
-1.61183	down	-2.00936	down
-1.32723	down	-2.0092	down
1.490257	up	2.008793	up
1.362888	up	2.007978	up

1.163343	up	-2.00796	down
-1.00211	down	-2.00759	down
-1.27099	down	-2.0071	down
-1.00641	down	-2.00685	down
1.241437	up	2.006794	up
-1.20087	down	-2.00673	down
1.113272	up	-2.00643	down
-1.45445	down	-2.00637	down
-1.36093	down	-2.00625	down
1.283423	up	-2.00545	down
-1.05288	down	-2.00533	down
3.248844	up	-2.00512	down
-1.05112	down	-2.00497	down
-1.10022	down	-2.00488	down
-1.01271	down	-2.00483	down
1.00701	up	2.004664	up
-1.36086	down	-2.00465	down
1.728044	up	2.004546	up
1.305209	up	2.004323	up
1.188106	up	2.003647	up
2.042664	up	-2.00363	down
1.005046	up	-2.00352	down
1.117649	up	2.003179	up
1.173402	up	2.002958	up
-1.45023	down	-2.00256	down
1.718383	up	2.00225	up
1.475328	up	2.001771	up
-1.55671	down	-2.00163	down
-34.1334	down	2.001506	up
1.512936	up	2.000636	up
-1.61828	down	-1.9998	down
-1.11384	down	-1.99965	down
1.348088	up	-1.99814	down
1.878268	up	-1.99747	down
-1.06194	down	-1.9973	down
1.78403	up	1.997119	up
-1.75957	down	-1.99689	down
1.030275	up	1.996806	up
-1.09586	down	-1.99673	down
1.784102	up	1.996506	up
-1.05052	down	-1.99593	down
-1.18108	down	-1.99588	down
-2.17525	down	1.99548	up
1.139332	up	1.995256	up
-1.57797	down	-1.99492	down
1.262569	up	-1.9948	down
1.771595	up	1.99405	up

-1.80951	down	-1.99402	down
1.502148	up	1.993915	up
1.218791	up	1.993798	up
1.268446	up	-1.99347	down
-1.05675	down	1.993276	up
-1.28801	down	-1.99257	down
1.329905	up	1.992419	up
1.10738	up	1.992407	up
-1.92597	down	1.991786	up
1.949945	up	-1.99142	down
-1.4202	down	-1.99139	down
1.180699	up	1.990585	up
2.527259	up	1.990457	up
1.798016	up	1.990119	up
1.918937	up	-1.98988	down
-1.03857	down	-1.98943	down
1.274738	up	-1.98941	down
-1.97718	down	-1.98931	down
2.099974	up	-1.98919	down
1.126493	up	-1.98883	down
-1.54246	down	-1.98871	down
-4.113	down	1.988203	up
-1.16813	down	-1.98804	down
-1.33903	down	-1.98774	down
1.272008	up	1.987611	up
-1.16851	down	-1.98731	down
-1.13541	down	-1.98729	down
-1.35534	down	-1.98698	down
-1.16367	down	-1.98677	down
-1.16828	down	-1.98605	down
1.064408	up	1.985964	up
-1.11289	down	-1.98581	down
-1.24383	down	-1.98579	down
-1.31556	down	-1.98547	down
3.604335	up	-1.98528	down
1.677294	up	1.985173	up
-1.89483	down	-1.98515	down
-3.01585	down	-1.98512	down
-1.03218	down	-1.98508	down
2.296047	up	-1.98459	down
-1.42761	down	-1.98458	down
-1.31084	down	-1.98433	down
-1.0898	down	-1.98403	down
-1.02792	down	-1.98401	down
-2.13445	down	-1.98355	down
-1.47772	down	1.983304	up
-1.50229	down	-1.98294	down

1.538522	up	1.982748	up
-1.55195	down	-1.98209	down
-1.06505	down	-1.98188	down
-1.10681	down	-1.98046	down
-1.39131	down	-1.98036	down
1.289193	up	1.980063	up
2.988766	up	1.978746	up
-1.08198	down	-1.9782	down
-1.12502	down	-1.97806	down
-1.62753	down	-1.97773	down
1.358158	up	-1.97736	down
1.53466	up	-1.97713	down
1.12675	up	1.976974	up
1.746495	up	1.97681	up
1.216949	up	1.976729	up
1.548294	up	1.976213	up
-1.24351	down	1.976085	up
-1.22386	down	-1.97594	down
1.16393	up	1.975695	up
1.326582	up	1.975131	up
-1.4001	down	-1.97499	down
-1.03342	down	-1.97426	down
1.446492	up	1.973785	up
-1.00883	down	1.973673	up
-1.20874	down	-1.97314	down
-1.62818	down	-1.97178	down
-1.36462	down	-1.97105	down
-1.10751	down	1.970323	up
-1.76734	down	-1.97016	down
-1.41944	down	-1.97006	down
1.483845	up	1.970012	up
1.128138	up	-1.96993	down
-2.1841	down	1.969833	up
-1.03049	down	-1.9697	down
-1.0901	down	-1.9696	down
-1.75768	down	-1.96937	down
1.687806	up	1.969057	up
1.008306	up	-1.96901	down
1.042168	up	-1.96845	down
1.889954	up	1.96761	up
2.682293	up	1.967421	up
-1.04934	down	-1.96736	down
-1.18276	down	-1.96719	down
1.03969	up	-1.96715	down
-1.0541	down	-1.96634	down
-2.03871	down	-1.96617	down
1.090205	up	1.965479	up

-1.68259	down	-1.96511	down
1.099348	up	1.964875	up
1.114696	up	-1.96373	down
-6.63495	down	1.96344	up
-2.00934	down	1.963396	up
2.120003	up	1.963269	up
2.937547	up	-1.96255	down
-1.57579	down	-1.96238	down
1.477839	up	-1.96229	down
-1.08658	down	-1.9605	down
-1.30696	down	-1.96041	down
2.855823	up	1.960345	up
-1.29501	down	-1.96033	down
-1.39973	down	-1.96026	down
1.896667	up	1.960124	up
1.827572	up	1.959448	up
-1.05501	down	-1.95927	down
-1.37117	down	-1.95907	down
1.146139	up	1.958811	up
-1.13678	down	-1.9588	down
1.322113	up	1.958772	up
1.005156	up	-1.95815	down
-1.20989	down	-1.95779	down
-2.01045	down	1.95776	up
1.294726	up	-1.95726	down
-1.46061	down	-1.95723	down
-2.08169	down	-1.95712	down
-1.02892	down	-1.95687	down
-1.24347	down	1.95683	up
1.72533	up	1.95681	up
-1.54866	down	-1.95626	down
-1.32193	down	-1.95625	down
1.196299	up	1.955394	up
1.348963	up	1.955299	up
1.226716	up	1.955225	up
2.012191	up	1.955072	up
1.370223	up	1.955058	up
1.033119	up	-1.955	down
-1.10795	down	-1.95495	down
1.719444	up	1.954922	up
1.097634	up	-1.95492	down
2.960935	up	1.954744	up
1.656366	up	1.95393	up
-1.2929	down	-1.95354	down
-2.12442	down	1.953204	up
1.147457	up	-1.95314	down
1.531163	up	1.953095	up

1.440316	up	1.952828	up
-1.85449	down	1.951856	up
2.080363	up	1.951546	up
-1.28512	down	-1.95133	down
-1.29625	down	-1.95132	down
1.786223	up	1.951009	up
1.505183	up	1.950429	up
1.791159	up	1.950344	up
1.880254	up	1.95015	up
2.841317	up	-1.9499	down
1.166546	up	1.949096	up
1.041208	up	1.948971	up
1.630611	up	1.948761	up
-1.38463	down	-1.94855	down
-1.28067	down	-1.94843	down
-2.48717	down	1.947643	up
1.146143	up	1.945762	up
-2.30182	down	1.945241	up
-1.2507	down	-1.94483	down
-1.38548	down	-1.94479	down
-1.19157	down	1.944331	up
2.103493	up	1.944196	up
-1.22771	down	-1.9439	down
-1.12771	down	-1.94386	down
-1.33657	down	-1.94375	down
-1.83966	down	-1.94335	down
-1.86439	down	1.942793	up
-1.23447	down	-1.94272	down
1.150118	up	1.942586	up
1.431366	up	1.94251	up
2.517171	up	-1.94241	down
-2.53195	down	1.942271	up
1.475994	up	1.942195	up
-1.47864	down	-1.94219	down
1.328693	up	1.941121	up
1.443503	up	1.940804	up
2.55838	up	1.94073	up
-1.78415	down	-1.94064	down
1.353948	up	1.940049	up
-1.0308	down	-1.93976	down
-1.21338	down	-1.93939	down
-1.03958	down	-1.93849	down
-3.9345	down	-1.938	down
1.285698	up	1.937834	up
-3.00588	down	-1.9374	down
1.124793	up	1.937325	up
1.21969	up	1.937092	up

-1.50199	down	-1.93689	down
1.058002	up	1.936419	up
1.457033	up	1.936319	up
-1.21101	down	-1.93567	down
-1.20632	down	-1.9353	down
-1.09677	down	-1.93509	down
1.726332	up	1.934867	up
-1.59347	down	-1.93442	down
-1.66484	down	-1.93295	down
1.260138	up	1.932761	up
1.425038	up	-1.93205	down
-1.36743	down	1.93184	up
-1.11408	down	-1.93149	down
-1.38617	down	-1.93148	down
1.397989	up	1.931424	up
1.153513	up	1.931267	up
1.049141	up	-1.93118	down
-1.21424	down	-1.93088	down
-1.33128	down	-1.93027	down
-1.13844	down	1.929595	up
-1.02517	down	-1.92955	down
-1.14825	down	-1.92948	down
-1.32898	down	1.929027	up
-1.39017	down	-1.92877	down
1.168905	up	-1.92845	down
-1.43623	down	-1.9281	down
-2.02429	down	-1.92797	down
-1.18525	down	-1.92795	down
-1.13418	down	-1.92777	down
-1.98521	down	-1.92759	down
1.724274	up	-1.92757	down
1.308509	up	1.926922	up
-1.53499	down	1.926473	up
-2.16514	down	-1.92633	down
1.216068	up	1.926175	up
1.113175	up	-1.92584	down
-1.84317	down	-1.92545	down
-1.36282	down	-1.92519	down
-1.20368	down	-1.92504	down
-1.19635	down	-1.92494	down
-1.22664	down	-1.92492	down
-1.06433	down	-1.92487	down
-1.15985	down	-1.92482	down
4.606854	up	1.924808	up
-3.70268	down	1.924792	up
1.451577	up	1.924341	up
1.128125	up	-1.92403	down

1.641159	up	-1.92347	down
-1.08721	down	-1.92337	down
-1.4902	down	-1.92334	down
1.602166	up	1.922959	up
-1.41547	down	-1.92282	down
-1.69108	down	-1.92281	down
-1.19559	down	-1.92266	down
-1.49625	down	1.921846	up
-1.26246	down	-1.92181	down
1.953199	up	-1.92158	down
-1.28887	down	-1.92157	down
-1.16862	down	-1.92059	down
1.351785	up	1.920481	up
-11.1512	down	1.920418	up
1.506939	up	1.920414	up
-1.18489	down	-1.92	down
-1.08913	down	-1.91991	down
-1.40632	down	-1.91967	down
-1.33652	down	-1.91947	down
1.047859	up	1.919404	up
1.436196	up	1.919272	up
-1.16985	down	-1.91925	down
-1.4545	down	-1.91905	down
1.698245	up	1.918312	up
-7.5599	down	1.918246	up
-1.68134	down	1.9177	up
-2.26606	down	-1.91737	down
1.183213	up	-1.91734	down
-1.17524	down	-1.91701	down
-1.44526	down	-1.91582	down
1.092174	up	1.915703	up
9.770154	up	1.915664	up
-1.20259	down	-1.91565	down
-1.11279	down	-1.91558	down
-1.36095	down	-1.91557	down
-1.02898	down	-1.91543	down
-1.80728	down	-1.91537	down
-1.08368	down	-1.91536	down
1.395006	up	1.914906	up
-1.40795	down	-1.91484	down
-1.03669	down	-1.91412	down
-1.01049	down	-1.91388	down
2.731897	up	1.913703	up
1.35995	up	-1.9137	down
-1.32358	down	-1.9136	down
1.771802	up	1.91348	up
-1.25342	down	-1.91346	down

1.012461	up	-1.9119	down
-1.16155	down	-1.91161	down
-2.94349	down	-1.91156	down
-1.85199	down	1.911228	up
1.209365	up	1.911208	up
-1.49594	down	1.909563	up
-1.13827	down	-1.90927	down
-1.56728	down	-1.9089	down
-1.19027	down	-1.9088	down
-1.13012	down	-1.90873	down
-1.21952	down	-1.90839	down
-4.43801	down	1.908359	up
1.629231	up	1.908262	up
2.027209	up	-1.90805	down
-1.29752	down	-1.9078	down
1.028293	up	-1.90747	down
2.134031	up	1.907358	up
-1.28677	down	-1.90701	down
1.160906	up	-1.90692	down
-2.41985	down	-1.90674	down
1.083216	up	1.906709	up
1.737216	up	1.906446	up
1.153331	up	-1.90543	down
-1.89608	down	1.90468	up
1.109661	up	-1.90388	down
-1.28967	down	-1.90385	down
6.029842	up	1.903585	up
-1.38823	down	-1.90357	down
16.15422	up	1.903322	up
-2.10524	down	1.902193	up
-1.63156	down	-1.9021	down
-1.15729	down	1.902047	up
-1.12655	down	-1.90154	down
-1.33365	down	-1.90126	down
-1.47149	down	1.9011	up
-2.40643	down	-1.90048	down
-1.22211	down	-1.90021	down
1.341858	up	-1.9002	down
-1.33164	down	-1.89882	down
-1.81076	down	1.898691	up
1.512746	up	-1.89827	down
1.587105	up	-1.89803	down
2.311703	up	1.89734	up
1.080202	up	1.897141	up
-1.04784	down	-1.89709	down
2.389346	up	-1.8961	down
2.098399	up	1.89591	up

-1.30577	down	-1.89584	down
-1.3931	down	1.895826	up
5.010756	up	1.895589	up
2.706809	up	-1.89552	down
-1.07275	down	-1.89543	down
1.940341	up	1.895395	up
1.359918	up	1.89509	up
-1.47605	down	-1.89454	down
-1.46912	down	1.894491	up
1.192434	up	1.894266	up
1.27072	up	1.894164	up
1.213873	up	1.894153	up
1.122953	up	-1.89395	down
7.637003	up	1.893672	up
1.343296	up	-1.89349	down
1.493017	up	1.893406	up
1.653713	up	1.893346	up
2.149105	up	1.893273	up
2.168691	up	1.893245	up
-1.12328	down	-1.89319	down
-1.19569	down	-1.89285	down
1.198711	up	-1.89282	down
-1.15368	down	-1.89259	down
-1.14063	down	-1.89197	down
1.24612	up	-1.89172	down
-1.16582	down	-1.89126	down
-1.48907	down	-1.8912	down
-1.0716	down	-1.89115	down
1.385692	up	1.891086	up
-1.94038	down	-1.89096	down
-1.52001	down	-1.89085	down
-3.48727	down	-1.89062	down
-1.32091	down	-1.89052	down
1.071367	up	1.889943	up
1.90107	up	1.888968	up
1.522153	up	1.888409	up
1.952796	up	1.888146	up
1.575132	up	1.888043	up
2.736961	up	1.88733	up
1.461785	up	1.887212	up
1.875512	up	1.886845	up
1.244757	up	1.886632	up
-1.45544	down	-1.88659	down
1.089083	up	1.885706	up
-1.28819	down	-1.88566	down
1.38661	up	1.885602	up
-1.83664	down	1.885451	up

1.906351	up	-1.88531	down
1.27097	up	1.885301	up
1.269569	up	1.885039	up
-1.05778	down	1.885016	up
-1.59106	down	-1.88496	down
1.414615	up	1.884901	up
-1.18416	down	-1.88437	down
1.955722	up	1.884158	up
-1.15954	down	-1.88358	down
1.696385	up	-1.88349	down
2.578172	up	1.88348	up
1.286474	up	-1.88317	down
1.210859	up	-1.88296	down
2.613928	up	1.882862	up
-1.33416	down	1.882674	up
1.064875	up	-1.88265	down
2.788547	up	1.882427	up
2.595526	up	1.882017	up
-1.76172	down	-1.8814	down
-1.12621	down	-1.88135	down
1.492879	up	1.88135	up
-1.62422	down	-1.88135	down
-1.53947	down	1.881318	up
1.004035	up	-1.88072	down
-1.82638	down	-1.88063	down
-5.83912	down	1.880109	up
2.322184	up	1.87967	up
1.110907	up	1.879659	up
1.561069	up	1.879468	up
1.021345	up	1.879393	up
1.54833	up	-1.87909	down
1.187755	up	1.879086	up
1.170811	up	1.879086	up
1.492276	up	1.87908	up
-4.32394	down	1.878833	up
1.40538	up	1.878822	up
-4.55502	down	1.877755	up
1.199261	up	1.877389	up
-1.26052	down	-1.87667	down
-1.85375	down	-1.8765	down
-1.18998	down	-1.87633	down
1.195232	up	-1.87597	down
-1.15978	down	-1.87591	down
-1.25215	down	-1.8757	down
-1.50616	down	-1.8757	down
1.016526	up	-1.87546	down
-1.28052	down	-1.87538	down

1.485499	up	1.875025	up
-1.36647	down	-1.87485	down
1.341579	up	1.874197	up
-1.808	down	-1.87413	down
1.086749	up	-1.87398	down
-1.18563	down	-1.87365	down
2.828115	up	1.8736	up
-1.0858	down	-1.87352	down
-2.20198	down	1.873033	up
1.385254	up	-1.87267	down
3.384759	up	1.872428	up
-1.68262	down	-1.87229	down
-1.98461	down	1.872224	up
-1.62616	down	-1.87205	down
-1.40781	down	-1.87183	down
-1.12544	down	-1.87182	down
1.433279	up	-1.87174	down
-1.09505	down	-1.87155	down
-1.25219	down	-1.87143	down
1.415681	up	-1.87114	down
-1.5128	down	1.871085	up
-1.30244	down	-1.87077	down
-2.23977	down	1.870752	up
-2.14311	down	-1.87026	down
-1.2807	down	-1.86959	down
1.023307	up	-1.86922	down
1.669147	up	1.869184	up
1.33434	up	1.868798	up
1.147934	up	1.868793	up
1.764539	up	-1.8687	down
1.262562	up	1.868004	up
-12.1175	down	1.866581	up
-1.13095	down	1.86651	up
1.440813	up	-1.86598	down
-1.29422	down	-1.86578	down
-1.27023	down	1.865739	up
-1.8958	down	-1.86572	down
-1.68266	down	-1.86563	down
-1.3086	down	-1.86557	down
1.093923	up	1.865496	up
1.312235	up	1.865371	up
2.077741	up	1.864876	up
1.783377	up	1.863916	up
-1.53679	down	1.863887	up
1.681779	up	-1.86382	down
-2.84774	down	-1.86366	down
-4.0992	down	1.863509	up

1.13589	up	1.86337	up
-1.84419	down	-1.86236	down
1.8796	up	1.86221	up
-1.14376	down	-1.86217	down
1.514936	up	1.861973	up
-1.0086	down	-1.86185	down
-1.59854	down	-1.86169	down
-1.28529	down	-1.86146	down
-1.14454	down	1.861305	up
-1.86865	down	-1.86122	down
-1.10572	down	-1.86092	down
-2.03035	down	-1.86056	down
-1.04031	down	1.860244	up
-1.35138	down	1.860131	up
-1.22488	down	-1.86004	down
-1.09192	down	-1.85878	down
1.46978	up	1.858409	up
1.232314	up	1.858321	up
2.182841	up	1.858202	up
-1.47313	down	-1.85808	down
-1.15019	down	-1.85662	down
-1.16437	down	-1.8565	down
-1.13164	down	-1.85566	down
-1.34754	down	-1.85563	down
-1.27671	down	-1.85556	down
3.181913	up	1.855398	up
-1.28923	down	-1.855	down
1.398546	up	1.854886	up
-1.35086	down	-1.85485	down
-1.73229	down	-1.85453	down
1.847473	up	-1.85444	down
1.071578	up	1.854396	up
1.357875	up	1.85435	up
1.372927	up	-1.85423	down
1.272744	up	-1.85398	down
-1.30348	down	-1.85386	down
-1.22501	down	-1.85354	down
-1.37207	down	-1.85334	down
1.667688	up	-1.85238	down
1.147898	up	1.852327	up
-1.01302	down	-1.85216	down
-1.48173	down	1.851848	up
-1.15819	down	-1.85173	down
-1.36849	down	-1.85157	down
1.324688	up	1.851178	up
1.291222	up	1.851129	up
-1.93668	down	-1.85076	down

-1.81124	down	-1.85042	down
1.421656	up	-1.85035	down
1.930688	up	-1.85004	down
-1.25351	down	-1.84978	down
3.514064	up	1.849425	up
-1.32014	down	-1.84898	down
-1.67584	down	-1.84884	down
1.391526	up	-1.84877	down
-1.28982	down	-1.84855	down
1.683926	up	1.848458	up
1.404331	up	1.848122	up
1.146799	up	1.848087	up
1.709069	up	1.847893	up
-1.46977	down	-1.84786	down
1.343266	up	1.847522	up
1.20037	up	1.847234	up
-1.23429	down	-1.84717	down
2.466206	up	1.847103	up
1.711671	up	1.847083	up
-1.30139	down	-1.84702	down
1.016341	up	-1.84668	down
-4.10378	down	1.846246	up
-1.25305	down	-1.84622	down
-1.00522	down	-1.84621	down
-1.0996	down	-1.84619	down
-2.40509	down	1.84616	up
-1.4408	down	-1.84611	down
-1.30142	down	-1.84551	down
-1.40054	down	-1.8455	down
-4.15586	down	-1.84542	down
3.137502	up	-1.84517	down
-1.40764	down	1.844504	up
-1.63209	down	-1.844	down
-1.11686	down	-1.84397	down
1.163145	up	-1.84393	down
1.056436	up	1.843811	up
-1.19902	down	-1.84367	down
1.11824	up	-1.84366	down
-1.22909	down	-1.84337	down
1.255537	up	1.843361	up
-1.23421	down	1.843076	up
1.49391	up	-1.84285	down
-1.2469	down	-1.84269	down
3.190221	up	1.842473	up
1.11388	up	1.841882	up
1.469881	up	1.841156	up
5.680453	up	1.840963	up

1.811908	up	-1.84081	down
1.245765	up	1.840562	up
-1.43516	down	1.840514	up
1.493507	up	1.840479	up
-1.07393	down	-1.84044	down
1.005873	up	1.840385	up
-1.62392	down	-1.84027	down
-1.09834	down	-1.84021	down
-2.46667	down	-1.84005	down
-1.59738	down	1.839902	up
1.527092	up	1.839616	up
1.197041	up	-1.83959	down
1.690496	up	1.838769	up
1.709122	up	1.838748	up
-1.92197	down	-1.8387	down
-1.11733	down	-1.83859	down
-1.04026	down	-1.83833	down
2.120305	up	-1.83818	down
-1.36261	down	-1.83764	down
-1.14429	down	-1.83667	down
-1.35898	down	-1.83647	down
1.231381	up	1.836321	up
-1.401	down	-1.83606	down
1.513664	up	1.835902	up
1.704473	up	1.835843	up
-2.77801	down	-1.83571	down
-1.359	down	1.835268	up
1.245405	up	-1.83496	down
-2.29423	down	1.834928	up
-1.37832	down	-1.83482	down
1.658779	up	-1.83481	down
2.083965	up	1.834603	up
17.07908	up	1.834524	up
1.240543	up	1.834459	up
2.706444	up	1.834093	up
1.445532	up	1.833863	up
2.111255	up	1.833538	up
-1.17582	down	-1.8334	down
-1.62555	down	-1.83299	down
1.907294	up	1.832872	up
-1.30298	down	-1.83273	down
-1.45664	down	-1.83229	down
1.737516	up	1.831985	up
-1.34356	down	-1.83033	down
3.706027	up	1.82995	up
-1.26864	down	-1.82991	down
-1.29985	down	1.829841	up

1.286734	up	1.829171	up
-2.89745	down	1.828957	up
1.390371	up	1.828875	up
-1.87645	down	-1.82833	down
2.052362	up	1.827981	up
2.158466	up	1.827925	up
-1.30029	down	1.827693	up
-2.2492	down	1.82728	up
1.345244	up	1.826424	up
1.121647	up	-1.82618	down
1.142591	up	1.826162	up
-1.17852	down	-1.82575	down
-3.56285	down	1.825595	up
-1.35945	down	-1.82514	down
1.574789	up	1.825009	up
-1.4324	down	-1.8247	down
1.525391	up	-1.82444	down
-2.19615	down	1.824371	up
2.758639	up	1.8242	up
1.725705	up	-1.82414	down
-1.10504	down	-1.82411	down
-1.32128	down	-1.82379	down
-2.02979	down	1.823728	up
-1.33051	down	-1.82361	down
1.688217	up	1.823531	up
1.567208	up	1.82343	up
-1.37912	down	-1.82333	down
-1.55472	down	-1.82307	down
-2.16976	down	1.822713	up
-1.43668	down	1.822641	up
-1.84622	down	-1.82262	down
2.385991	up	1.822257	up
-1.16056	down	-1.82211	down
1.381016	up	-1.82135	down
1.656076	up	1.820994	up
1.565874	up	1.820558	up
1.174593	up	-1.82047	down
-1.19416	down	-1.82039	down
-1.42463	down	-1.81978	down
1.006805	up	-1.81943	down
-1.22182	down	-1.81918	down
-1.03357	down	-1.81917	down
-1.38184	down	-1.81902	down
1.808633	up	1.819009	up
1.3832	up	1.818868	up
1.009175	up	1.81871	up
2.505116	up	1.818566	up

1.398764	up	-1.81853	down
-3.99172	down	1.818488	up
-1.32417	down	-1.81838	down
2.076411	up	1.818269	up
-1.03994	down	-1.81782	down
-1.5446	down	1.817427	up
-4.00172	down	1.81729	up
1.667523	up	1.817119	up
-1.23469	down	-1.81706	down
1.162396	up	1.816852	up
1.740567	up	-1.81671	down
-1.72631	down	-1.8162	down
-1.26152	down	-1.81616	down
1.302506	up	-1.81548	down
1.6065	up	1.815248	up
-1.47022	down	-1.81514	down
2.071802	up	-1.8147	down
-1.16666	down	-1.8146	down
1.337754	up	1.814001	up
1.236799	up	1.813791	up
1.564151	up	-1.81374	down
4.113009	up	1.81367	up
-1.22574	down	-1.81359	down
-1.6806	down	-1.81357	down
1.378832	up	1.81301	up
1.197465	up	1.812906	up
-1.65825	down	-1.81284	down
1.453767	up	-1.81194	down
1.223472	up	1.811583	up
2.742023	up	1.811512	up
2.797609	up	1.811008	up
1.387786	up	-1.81096	down
-1.12049	down	-1.81053	down
-1.73871	down	-1.81051	down
-1.13408	down	-1.81037	down
1.301499	up	1.810248	up
1.186179	up	1.810222	up
-1.27334	down	-1.81007	down
1.541938	up	1.809966	up
-1.18707	down	-1.80957	down
1.405011	up	-1.80921	down
-1.34401	down	-1.80889	down
1.007423	up	1.808501	up
-1.18109	down	-1.80793	down
-1.62888	down	1.807889	up
-2.52075	down	1.807737	up
-1.11535	down	-1.80719	down

-1.74455	down	-1.80681	down
2.528568	up	1.805724	up
-1.32356	down	-1.80569	down
-1.20936	down	-1.80563	down
2.108974	up	1.805218	up
-2.43998	down	-1.80502	down
-2.4281	down	-1.80501	down
-1.29778	down	1.804961	up
-1.10833	down	-1.80487	down
-1.16366	down	-1.80479	down
-1.41483	down	-1.80467	down
-1.14258	down	-1.80439	down
-1.1155	down	-1.80427	down
1.165153	up	1.804093	up
1.119142	up	1.803842	up
1.207528	up	1.803805	up
1.273646	up	1.803599	up
-2.0865	down	1.803546	up
-1.13843	down	-1.80327	down
-1.27322	down	-1.80313	down
-1.29553	down	-1.803	down
1.55727	up	1.802884	up
-1.30703	down	-1.80255	down
1.86866	up	1.802541	up
-1.90431	down	1.802424	up
1.533768	up	-1.8024	down
-1.17178	down	-1.80228	down
-1.61154	down	-1.80227	down
-1.1316	down	-1.80224	down
-1.08648	down	-1.80218	down
-1.33084	down	-1.80208	down
-3.93988	down	1.802066	up
-1.1673	down	-1.80148	down
-1.83271	down	-1.80112	down
-1.70206	down	-1.80097	down
1.507194	up	1.800883	up
1.536409	up	1.800852	up
1.835855	up	1.8008	up
-1.34288	down	-1.80057	down
-1.63126	down	-1.80056	down
-1.21261	down	1.80054	up
1.180485	up	1.80046	up
-1.42536	down	-1.79953	down
-1.03778	down	-1.79938	down
-2.7266	down	-1.79922	down
-2.22066	down	1.798813	up
-3.42565	down	-1.79785	down

-1.72868	down	-1.79772	down
-1.15084	down	-1.79726	down
1.7145	up	1.797054	up
-1.29465	down	1.796961	up
1.513924	up	-1.79686	down
2.337876	up	1.796401	up
-1.55581	down	-1.79613	down
-1.72722	down	-1.79609	down
-1.58447	down	-1.79589	down
-1.26931	down	-1.79583	down
-2.08625	down	-1.79579	down
-1.30048	down	-1.79557	down
-1.17063	down	-1.79536	down
1.206915	up	1.795296	up
-1.23932	down	-1.7951	down
-1.15409	down	-1.795	down
1.328991	up	1.794744	up
-1.12776	down	-1.79459	down
-1.31059	down	-1.79436	down
-1.26231	down	-1.79428	down
1.418005	up	-1.79394	down
2.404538	up	-1.79342	down
-1.4086	down	-1.79313	down
-1.05736	down	-1.79296	down
-1.28097	down	-1.79237	down
-1.35741	down	-1.79217	down
1.760425	up	1.792087	up
-1.26172	down	-1.79179	down
-1.26188	down	-1.79153	down
-1.32689	down	-1.79085	down
1.770404	up	-1.79063	down
1.386976	up	-1.78984	down
1.117514	up	1.789812	up
-1.34446	down	-1.78948	down
1.395998	up	1.789443	up
1.365511	up	1.788979	up
-1.21074	down	-1.78795	down
-1.05229	down	-1.78774	down
1.269688	up	-1.78758	down
2.369576	up	1.786639	up
-2.16922	down	-1.78653	down
-1.22151	down	1.786324	up
-1.46162	down	-1.78628	down
-1.26091	down	1.786088	up
-1.19655	down	1.785698	up
1.209047	up	1.785679	up
1.510104	up	-1.78567	down

-1.15132	down	-1.78558	down
-1.27497	down	-1.78519	down
-1.07602	down	1.785134	up
-2.12022	down	-1.785	down
-1.66969	down	-1.78482	down
-1.62839	down	-1.78417	down
1.030356	up	-1.78413	down
-2.65284	down	1.783981	up
-1.20156	down	1.783898	up
-1.0381	down	1.783496	up
1.17478	up	1.782879	up
1.22672	up	1.782526	up
-1.60862	down	-1.78251	down
-1.46843	down	-1.78201	down
1.237034	up	1.78182	up
1.904775	up	1.781566	up
1.164694	up	1.781025	up
1.934229	up	1.780772	up
-1.1748	down	-1.78034	down
-1.47062	down	-1.78033	down
4.52434	up	1.780109	up
-2.57704	down	1.780009	up
-1.2111	down	1.779917	up
2.918459	up	1.779743	up
-1.15205	down	-1.77931	down
2.872754	up	1.779272	up
-1.43183	down	-1.77921	down
1.439235	up	1.778498	up
-1.13901	down	-1.7782	down
1.198285	up	-1.77798	down
2.277458	up	-1.77722	down
1.34792	up	1.777147	up
-1.2078	down	1.777117	up
-1.375	down	-1.77682	down
2.153686	up	1.776617	up
1.252517	up	1.776551	up
1.949416	up	-1.77604	down
-1.5222	down	-1.77559	down
1.179732	up	-1.77551	down
-1.21444	down	-1.77498	down
1.24946	up	1.774831	up
1.319833	up	1.774785	up
1.138755	up	-1.77469	down
-1.33189	down	-1.77451	down
2.406092	up	-1.77449	down
1.304532	up	1.774198	up
1.273122	up	1.77402	up

1.240878	up	1.773277	up
1.767442	up	1.772991	up
1.304008	up	1.772961	up
-1.28846	down	-1.77276	down
1.577842	up	-1.77252	down
1.593153	up	1.772236	up
1.460909	up	1.772131	up
1.136409	up	1.771884	up
-1.47998	down	-1.77182	down
-1.00246	down	-1.77176	down
-1.19287	down	1.771716	up
9.15344	up	1.771076	up
-1.2456	down	1.771059	up
-1.40588	down	1.770995	up
3.559678	up	1.770975	up
-1.53929	down	-1.77087	down
2.346083	up	1.770796	up
-2.21125	down	1.770761	up
1.321397	up	1.770529	up
-1.24638	down	-1.77008	down
3.629605	up	1.770011	up
-1.58886	down	-1.76996	down
-2.1497	down	1.769925	up
-1.74819	down	-1.76902	down
1.212253	up	1.768746	up
-1.57867	down	-1.76874	down
-6.50077	down	-1.76854	down
1.649149	up	-1.76775	down
1.397429	up	1.767435	up
2.015882	up	1.766393	up
1.386849	up	1.765856	up
-1.1466	down	-1.76502	down
-1.62908	down	-1.76491	down
-1.87273	down	-1.76481	down
-2.10463	down	1.764586	up
1.19169	up	1.764189	up
-1.23991	down	-1.7641	down
-1.2551	down	-1.76388	down
-1.18577	down	-1.7638	down
3.823039	up	-1.76378	down
-1.25634	down	-1.76358	down
-1.51658	down	-1.76292	down
3.067679	up	1.762824	up
-1.26768	down	-1.76282	down
-1.8042	down	-1.76248	down
1.43942	up	1.761852	up
-1.22272	down	-1.76161	down

1.589437	up	1.761488	up
1.104854	up	-1.76137	down
2.073099	up	1.760811	up
-1.79616	down	-1.76064	down
1.033998	up	-1.76038	down
-1.17109	down	-1.76038	down
-1.29447	down	-1.76018	down
2.307124	up	1.76006	up
-2.98994	down	-1.7598	down
-1.76855	down	1.759754	up
-2.02251	down	-1.75954	down
-1.13915	down	-1.75937	down
-1.36205	down	-1.75907	down
1.482671	up	1.758735	up
-1.37527	down	-1.75857	down
-1.23521	down	1.758533	up
-1.19639	down	-1.75821	down
-1.65914	down	-1.75795	down
1.908662	up	1.757928	up
1.164864	up	1.757692	up
1.858153	up	-1.75731	down
-1.15293	down	-1.7571	down
-1.94618	down	-1.75698	down
-1.20173	down	-1.75647	down
-1.31642	down	-1.7564	down
-1.2518	down	1.756377	up
1.464395	up	1.756336	up
1.27249	up	1.756207	up
10.99936	up	1.756111	up
1.288199	up	1.756082	up
-1.39315	down	-1.75554	down
-1.18559	down	-1.75495	down
1.855686	up	1.754905	up
-2.04979	down	-1.75391	down
1.444767	up	1.753754	up
1.732268	up	1.752994	up
2.022738	up	-1.75267	down
-1.4639	down	-1.75229	down
-1.76049	down	-1.75178	down
1.155986	up	-1.75151	down
-1.06786	down	1.75144	up
-1.77403	down	1.750971	up
-1.35144	down	-1.75093	down
-1.27143	down	1.750683	up
2.192508	up	1.75068	up
-1.30573	down	-1.75036	down
-1.47281	down	-1.75013	down

-1.00206	down	-1.7497	down
1.314785	up	-1.74942	down
4.977393	up	1.749077	up
1.295288	up	1.748993	up
-1.19134	down	-1.74893	down
2.277618	up	1.748193	up
-1.28618	down	-1.74763	down
-1.31003	down	-1.74731	down
-1.96003	down	1.747282	up
-1.67392	down	-1.74723	down
-1.2488	down	-1.74706	down
1.235348	up	1.74703	up
-1.34227	down	1.746954	up
-1.27754	down	-1.7469	down
1.438555	up	-1.74688	down
-1.74289	down	1.746789	up
-1.44417	down	-1.74674	down
-1.09283	down	-1.74672	down
-1.45012	down	-1.7465	down
1.198764	up	-1.74612	down
1.306536	up	1.745333	up
-1.65837	down	-1.74496	down
1.315061	up	1.744921	up
1.999091	up	1.74483	up
-1.19597	down	-1.74481	down
2.301716	up	-1.74459	down
1.34599	up	1.744413	up
1.093098	up	-1.74437	down
3.368227	up	1.744366	up
1.436807	up	-1.74433	down
1.200875	up	-1.74379	down
-1.57082	down	-1.74312	down
-1.60357	down	-1.74284	down
1.39505	up	-1.74228	down
1.176975	up	-1.74224	down
-1.21354	down	-1.74172	down
-1.16939	down	-1.74168	down
-1.10646	down	-1.7415	down
-1.0929	down	-1.74129	down
1.758915	up	-1.74126	down
2.278072	up	1.741244	up
1.234179	up	1.741202	up
-1.33623	down	-1.74117	down
-1.34724	down	-1.74117	down
1.374108	up	1.741106	up
-2.19876	down	1.740913	up
-1.80448	down	-1.74081	down

-1.68591	down	-1.74054	down
-1.217	down	-1.74054	down
1.970914	up	1.740206	up
-1.59985	down	-1.74017	down
-1.65235	down	-1.7401	down
1.112406	up	-1.73936	down
1.240719	up	1.739034	up
1.461176	up	1.738944	up
4.623991	up	1.7388	up
-1.68392	down	-1.73824	down
2.251544	up	1.738043	up
-1.37916	down	-1.7374	down
1.641292	up	1.737106	up
-1.1554	down	-1.73692	down
-1.23357	down	-1.73647	down
-1.15887	down	-1.73644	down
1.244133	up	-1.73609	down
-5.99118	down	1.736049	up
-1.24532	down	-1.73593	down
4.002332	up	1.735708	up
-1.58948	down	-1.73531	down
-1.63428	down	-1.73483	down
1.339193	up	-1.73477	down
-1.48864	down	-1.73461	down
1.563836	up	1.734577	up
-1.88998	down	-1.73448	down
-1.30733	down	-1.73402	down
-1.2241	down	-1.73387	down
-2.08209	down	1.73362	up
-1.0773	down	-1.7335	down
-1.26009	down	-1.73327	down
1.24943	up	-1.73315	down
-1.39643	down	-1.73286	down
-1.16416	down	-1.73273	down
-2.62646	down	1.732628	up
-1.60984	down	1.73196	up
1.99111	up	1.731837	up
1.346249	up	1.731788	up
1.25855	up	1.731731	up
-2.04202	down	1.731638	up
-1.5513	down	-1.73153	down
-1.44689	down	-1.73131	down
-1.00428	down	1.731236	up
1.299461	up	-1.73099	down
-1.4869	down	1.730603	up
-1.18854	down	-1.73025	down
-1.66623	down	-1.72939	down

1.253166	up	1.72926	up
2.120306	up	1.729004	up
1.303301	up	-1.72897	down
-1.38842	down	1.728932	up
1.245533	up	1.728869	up
1.474988	up	1.728687	up
1.506328	up	1.728591	up
2.72026	up	1.7284	up
2.043623	up	1.728009	up
-1.33776	down	-1.72797	down
2.177106	up	1.727925	up
1.225474	up	1.727678	up
1.194702	up	1.727206	up
1.382546	up	-1.72672	down
1.619669	up	-1.72639	down
1.521597	up	-1.72628	down
1.225983	up	1.726217	up
5.025316	up	1.726078	up
-4.58772	down	1.726064	up
-1.97708	down	-1.72606	down
1.68615	up	1.725943	up
-1.2115	down	-1.72572	down
-1.31668	down	-1.72567	down
1.341889	up	1.725598	up
-1.28853	down	-1.72529	down
-1.56572	down	-1.7252	down
-1.50556	down	-1.72514	down
1.075314	up	1.724797	up
2.033581	up	1.724761	up
1.661394	up	1.724122	up
-1.81458	down	1.722591	up
-1.5812	down	-1.7225	down
1.354757	up	1.722199	up
1.760247	up	1.722004	up
-1.37871	down	-1.72148	down
-1.79022	down	-1.72104	down
-2.83617	down	1.720963	up
1.248447	up	1.720956	up
1.287139	up	1.720944	up
1.536098	up	1.720885	up
1.21292	up	1.720379	up
1.355832	up	1.720316	up
-1.5082	down	-1.71987	down
1.234114	up	-1.7193	down
-1.36947	down	-1.71896	down
1.189764	up	1.718887	up
-1.36635	down	-1.71839	down

-1.23533	down	-1.71819	down
1.747596	up	-1.7178	down
-1.56261	down	-1.71769	down
-1.53283	down	1.717141	up
-1.26951	down	-1.71712	down
1.694767	up	1.717	up
-1.20089	down	-1.71688	down
1.347442	up	-1.71673	down
2.610919	up	1.716249	up
-1.40032	down	-1.71622	down
-1.72172	down	-1.7161	down
-1.10524	down	-1.71602	down
1.25563	up	1.715854	up
1.485462	up	1.715805	up
3.607289	up	1.715637	up
1.595398	up	1.715245	up
1.113319	up	1.715075	up
-1.34561	down	-1.71441	down
-1.77814	down	1.714291	up
1.197159	up	-1.71373	down
-1.1805	down	-1.71367	down
-1.23438	down	-1.71363	down
-1.27142	down	-1.71267	down
2.111387	up	1.712414	up
1.151722	up	-1.71228	down
-1.38665	down	-1.71202	down
1.058301	up	-1.71183	down
-1.17451	down	-1.71142	down
1.727892	up	-1.7114	down
1.07276	up	-1.71135	down
-1.5072	down	-1.71107	down
-1.25675	down	-1.71097	down
1.304643	up	1.710953	up
1.092579	up	-1.71095	down
-1.56564	down	-1.7106	down
-1.42641	down	-1.71038	down
-1.64784	down	-1.71014	down
1.463991	up	1.709963	up
-1.52384	down	-1.70974	down
-1.02563	down	1.709649	up
-1.2443	down	-1.70922	down
1.260725	up	1.709037	up
1.173097	up	1.70898	up
-1.48853	down	-1.70866	down
-2.48163	down	1.708564	up
1.169425	up	1.708156	up
4.03303	up	1.707948	up

-1.55633	down	-1.70744	down
2.010199	up	-1.70743	down
-1.6339	down	1.70722	up
1.514409	up	1.706793	up
-1.34933	down	-1.70672	down
2.166656	up	1.706314	up
-1.09498	down	-1.7062	down
1.301206	up	1.705966	up
-1.26364	down	-1.70592	down
1.230807	up	-1.70502	down
-2.66035	down	1.704913	up
1.032852	up	1.704085	up
-2.12377	down	-1.70373	down
-1.29119	down	-1.70371	down
-1.51304	down	-1.70363	down
1.184332	up	-1.70361	down
-1.41909	down	1.703283	up
-1.44593	down	-1.70323	down
1.22906	up	1.703057	up
-1.30192	down	-1.70275	down
-1.79569	down	1.702742	up
1.740112	up	1.702322	up
1.029962	up	-1.70216	down
2.087342	up	-1.702	down
1.548557	up	1.701701	up
-1.21156	down	-1.70153	down
1.747769	up	-1.70143	down
-1.67638	down	-1.70117	down
2.472838	up	-1.70088	down
1.90969	up	-1.7007	down
1.756372	up	1.700606	up
-1.60395	down	-1.70038	down
-1.31828	down	-1.70033	down
1.348935	up	1.699945	up
-1.16791	down	-1.69989	down
2.652243	up	-1.69982	down
1.074814	up	-1.69953	down
1.408105	up	1.699289	up
-1.07886	down	-1.69908	down
1.561096	up	1.699024	up
1.212551	up	-1.69871	down
1.187812	up	1.698694	up
-1.814	down	-1.69852	down
1.354848	up	-1.6983	down
-1.23127	down	-1.69796	down
1.021905	up	-1.69772	down
-1.52607	down	-1.69756	down

1.574703	up	1.697078	up
-1.20658	down	-1.69703	down
-1.81691	down	-1.6969	down
-1.64703	down	-1.69671	down
-1.37707	down	-1.69607	down
2.063546	up	-1.69603	down
-1.16793	down	1.695663	up
1.397145	up	1.695652	up
-1.46781	down	-1.69562	down
1.23728	up	1.695607	up
-1.2825	down	-1.69554	down
-1.48803	down	-1.69525	down
1.052078	up	-1.69508	down
4.774857	up	1.69466	up
-1.38437	down	-1.69437	down
-1.54459	down	-1.69424	down
-1.31924	down	-1.694	down
-1.52529	down	1.693923	up
-1.2058	down	-1.69386	down
1.202551	up	-1.69365	down
-1.52045	down	-1.69323	down
-1.44336	down	-1.69319	down
-1.72103	down	-1.69294	down
-2.41353	down	1.692921	up
1.242624	up	-1.69285	down
-3.96475	down	1.692727	up
2.956564	up	1.692228	up
1.692342	up	1.692007	up
1.722013	up	-1.69199	down
-1.65844	down	-1.69192	down
-2.8065	down	-1.69184	down
-1.87729	down	-1.69152	down
1.81424	up	1.691431	up
1.070566	up	1.691278	up
-1.66624	down	1.690851	up
-1.57235	down	-1.69058	down
1.263798	up	-1.69049	down
-1.50713	down	-1.6903	down
1.917261	up	1.690215	up
1.911749	up	-1.6902	down
1.477628	up	1.690062	up
-2.95735	down	1.689953	up
1.464132	up	1.689366	up
-1.54607	down	-1.68918	down
-1.56075	down	1.689019	up
1.669956	up	-1.68893	down
-1.38107	down	-1.68834	down

-1.63326	down	-1.68816	down
1.889442	up	1.687832	up
1.032667	up	-1.68766	down
-1.50642	down	-1.68764	down
2.102187	up	1.687447	up
2.461682	up	1.687358	up
1.352248	up	1.687212	up
-1.75887	down	1.687134	up
2.670037	up	1.687128	up
-2.58141	down	-1.68672	down
1.758429	up	1.686572	up
-1.39698	down	-1.68624	down
-1.89581	down	-1.68607	down
-1.64474	down	-1.68557	down
2.712264	up	1.685368	up
1.734156	up	1.685277	up
1.644347	up	1.685244	up
1.920211	up	-1.68462	down
-2.52594	down	1.684566	up
1.988676	up	1.684379	up
-1.39787	down	1.684359	up
-1.36307	down	-1.68418	down
-1.62024	down	1.683995	up
1.657421	up	1.683864	up
-1.24987	down	-1.68364	down
-1.19485	down	-1.6835	down
1.361211	up	1.683258	up
2.087996	up	1.683205	up
-1.3344	down	1.682665	up
-1.6768	down	-1.68264	down
1.193874	up	-1.6823	down
-1.81527	down	1.682105	up
-1.5653	down	-1.68181	down
-1.27779	down	-1.68163	down
-1.11083	down	-1.68146	down
-1.38964	down	-1.68144	down
-1.72659	down	-1.68123	down
-1.44463	down	-1.68123	down
-6.62608	down	1.680892	up
1.439323	up	-1.68087	down
-1.08481	down	-1.68061	down
-1.21832	down	-1.68061	down
-1.24281	down	-1.68038	down
-1.19884	down	-1.68035	down
-1.2731	down	-1.68021	down
2.01248	up	1.679737	up
1.399743	up	-1.67958	down

-1.32419	down	-1.67957	down
-1.60524	down	-1.67908	down
-1.23344	down	1.67902	up
1.484742	up	-1.67893	down
-2.2562	down	-1.67872	down
-1.4486	down	-1.67853	down
1.545437	up	1.678362	up
1.044929	up	-1.67833	down
-1.55904	down	-1.67824	down
2.185373	up	1.678234	up
-1.33429	down	-1.67818	down
-1.46554	down	-1.67816	down
1.498917	up	1.677945	up
-1.04466	down	-1.6777	down
1.155482	up	-1.67697	down
-1.27952	down	-1.67681	down
-1.40136	down	-1.67643	down
-1.84615	down	1.676127	up
-1.2176	down	-1.6761	down
-1.19672	down	-1.67609	down
-1.36089	down	-1.67581	down
1.235496	up	-1.67566	down
-1.06846	down	-1.67561	down
1.508358	up	1.675573	up
-1.21166	down	-1.6755	down
-1.21144	down	-1.67494	down
1.688467	up	-1.67446	down
-1.01071	down	-1.67423	down
1.259964	up	1.674145	up
1.298501	up	1.672804	up
-2.34758	down	1.672215	up
-1.22226	down	-1.6721	down
-1.20802	down	-1.67209	down
-2.17272	down	-1.67184	down
-1.29825	down	-1.67165	down
-1.47151	down	-1.67142	down
-1.21631	down	-1.67139	down
-1.23708	down	-1.67136	down
1.095949	up	-1.67133	down
-1.55969	down	1.671288	up
2.922206	up	1.670987	up
-1.54685	down	-1.67068	down
-2.1072	down	1.670654	up
-2.82732	down	1.670607	up
1.281274	up	1.670373	up
1.05333	up	-1.67029	down
-1.68528	down	1.670254	up

2.281935	up	-1.66863	down
2.460719	up	1.667644	up
-1.20905	down	-1.66731	down
-1.54272	down	1.667227	up
1.488348	up	1.667142	up
-1.45182	down	-1.66712	down
-1.43461	down	-1.66704	down
-1.23831	down	-1.66698	down
-2.37655	down	1.666526	up
-2.02069	down	1.666417	up
-1.3765	down	-1.66564	down
1.430873	up	1.665596	up
1.438237	up	1.665361	up
-1.2039	down	-1.6653	down
-1.42887	down	-1.66513	down
-1.24368	down	-1.66498	down
1.269284	up	1.664876	up
1.728258	up	1.664851	up
-1.45748	down	-1.6647	down
1.26025	up	1.664289	up
-1.23975	down	-1.66405	down
-1.52841	down	-1.66388	down
-1.12506	down	1.663707	up
1.262861	up	1.663511	up
1.316434	up	1.663301	up
-1.11664	down	1.663277	up
-1.38065	down	-1.66311	down
-1.0804	down	1.662684	up
-1.23822	down	-1.66259	down
-1.1123	down	-1.6623	down
1.901398	up	1.66218	up
-1.26383	down	-1.6616	down
1.094018	up	-1.66158	down
-1.54435	down	-1.66149	down
-1.34743	down	-1.66138	down
-1.37236	down	-1.66091	down
-1.11526	down	-1.66075	down
1.547892	up	1.660551	up
1.722073	up	1.660414	up
1.268351	up	1.660015	up
1.688472	up	-1.65988	down
1.281346	up	-1.65981	down
-3.15469	down	1.659286	up
-1.94158	down	-1.65801	down
1.468859	up	1.65797	up
-1.20491	down	-1.65771	down
7.262265	up	1.657412	up

1.302836	up	-1.65729	down
-1.33904	down	-1.6572	down
1.397546	up	1.656885	up
-1.30899	down	-1.65654	down
-1.09675	down	-1.65607	down
1.757736	up	-1.65593	down
2.266387	up	1.65565	up
1.747759	up	1.655637	up
-1.22475	down	-1.6555	down
-1.21471	down	-1.65528	down
-1.61396	down	-1.6549	down
-1.55142	down	-1.65488	down
-1.84541	down	1.65462	up
-1.28262	down	-1.65461	down
-1.62702	down	-1.65451	down
-1.81791	down	-1.65351	down
1.304284	up	1.653442	up
-1.26347	down	1.653432	up
1.426965	up	1.653278	up
-1.4936	down	-1.65314	down
-1.29155	down	-1.65258	down
1.114622	up	-1.65236	down
1.267904	up	1.652309	up
-1.35813	down	-1.65195	down
1.463332	up	1.651622	up
1.390068	up	1.651617	up
-1.71536	down	-1.65135	down
-1.43514	down	-1.65104	down
-1.27877	down	-1.65063	down
-1.46298	down	-1.65033	down
4.08921	up	1.650292	up
-1.03925	down	-1.65003	down
-1.24054	down	-1.64992	down
-1.23951	down	-1.64966	down
2.912613	up	1.649291	up
1.448433	up	-1.64903	down
2.541979	up	1.648741	up
-1.44488	down	-1.64854	down
-1.07133	down	-1.64781	down
1.651475	up	1.647772	up
1.334976	up	1.64764	up
-1.09374	down	-1.64732	down
1.810475	up	1.647171	up
-1.2185	down	-1.64713	down
-1.53827	down	-1.64708	down
2.021751	up	1.646928	up
-1.41998	down	-1.64682	down

-1.73841	down	-1.64642	down
-1.69509	down	1.646122	up
1.399091	up	-1.64611	down
-1.34987	down	-1.64608	down
-1.32907	down	-1.64589	down
1.815283	up	-1.64588	down
1.561085	up	1.645581	up
-1.41754	down	-1.64529	down
1.874782	up	1.645194	up
1.780373	up	-1.64494	down
-1.25189	down	-1.64442	down
1.403198	up	1.644254	up
1.082166	up	1.644249	up
-1.29953	down	-1.64423	down
1.528527	up	1.644191	up
-2.22415	down	1.643759	up
2.451077	up	1.643757	up
-1.26011	down	-1.64344	down
1.74148	up	-1.64322	down
-1.22108	down	-1.64316	down
-1.55985	down	-1.64313	down
1.649189	up	1.642896	up
1.284293	up	1.642717	up
-1.44447	down	-1.64247	down
1.48991	up	1.642268	up
-1.39569	down	-1.64174	down
2.178393	up	-1.64122	down
1.130833	up	-1.64094	down
-1.42333	down	-1.64066	down
-1.16576	down	-1.64055	down
-1.23096	down	-1.64052	down
1.823888	up	1.640189	up
-1.08179	down	-1.64011	down
1.226016	up	-1.6399	down
1.858888	up	1.639862	up
2.031771	up	1.639602	up
-1.45209	down	-1.63934	down
-1.23576	down	-1.63921	down
1.582797	up	1.638988	up
-1.06784	down	-1.63892	down
-1.80447	down	-1.63879	down
-1.36476	down	-1.63874	down
-1.47151	down	-1.63873	down
1.409676	up	1.638651	up
-1.17505	down	-1.63829	down
-1.37327	down	-1.63813	down
-1.45316	down	-1.63811	down

-1.0321	down	1.637307	up
1.26213	up	-1.63715	down
-1.43274	down	-1.63704	down
1.934769	up	1.636485	up
1.5219	up	-1.63639	down
-1.22641	down	1.636195	up
1.138341	up	-1.63607	down
-2.425	down	-1.63604	down
-2.47409	down	1.63602	up
1.323669	up	1.635962	up
-1.03691	down	-1.63568	down
-1.17158	down	-1.63544	down
1.496505	up	1.634966	up
1.512991	up	1.634702	up
-1.4384	down	-1.63438	down
-1.60559	down	-1.63434	down
-1.41836	down	-1.634	down
-1.67756	down	-1.63387	down
1.282748	up	-1.63377	down
2.76904	up	1.633554	up
-1.13134	down	-1.63349	down
1.227305	up	1.632985	up
1.979127	up	-1.63285	down
-2.52813	down	-1.63265	down
1.855111	up	1.632598	up
1.24817	up	1.632574	up
-1.62084	down	-1.63245	down
-1.42822	down	-1.63217	down
-1.23816	down	-1.63152	down
-1.30484	down	-1.63106	down
-1.15346	down	1.630989	up
1.909193	up	1.630931	up
-1.24842	down	-1.63091	down
1.509964	up	1.630869	up
-1.57885	down	-1.63085	down
-1.15135	down	-1.63072	down
-1.28611	down	-1.63054	down
-1.40087	down	-1.63033	down
-1.22396	down	-1.63021	down
1.016534	up	1.630119	up
1.472666	up	1.629801	up
-1.43527	down	-1.62947	down
1.617295	up	1.629367	up
-1.38195	down	-1.62935	down
-1.17504	down	-1.62933	down
-13.3077	down	1.629115	up
3.02987	up	1.629046	up

1.604782	up	1.628232	up
-1.46821	down	1.627834	up
1.272254	up	1.627339	up
-2.08475	down	1.626833	up
-1.81433	down	1.626828	up
-1.70429	down	-1.62662	down
1.288011	up	1.626344	up
1.683	up	-1.62631	down
1.827429	up	1.626221	up
1.546919	up	-1.62619	down
-1.72904	down	-1.62613	down
-1.30259	down	-1.62604	down
2.31073	up	-1.62591	down
-1.71679	down	-1.62532	down
1.948679	up	1.625231	up
9.366871	up	1.624813	up
2.052517	up	-1.62478	down
-1.47856	down	-1.62469	down
4.319721	up	1.624537	up
-1.33673	down	-1.62447	down
1.128114	up	-1.62445	down
-1.2874	down	-1.62409	down
1.02435	up	1.623823	up
1.546451	up	1.62378	up
1.040783	up	-1.62375	down
-1.66127	down	-1.62368	down
1.225527	up	-1.62334	down
-1.27143	down	-1.62302	down
-1.24513	down	-1.623	down
-2.65071	down	-1.62288	down
-1.22847	down	-1.62288	down
1.31767	up	1.622834	up
1.254826	up	1.622579	up
-1.33528	down	-1.62241	down
-3.11569	down	1.622159	up
-1.63086	down	-1.6221	down
-1.33106	down	-1.62182	down
1.891275	up	-1.62178	down
-1.24052	down	-1.62172	down
1.610284	up	1.621233	up
-1.11627	down	-1.62044	down
-1.59488	down	-1.62015	down
1.070926	up	-1.62009	down
-1.11296	down	-1.62003	down
1.388805	up	-1.61996	down
-1.56847	down	-1.61966	down
-1.75818	down	-1.61965	down

1.226468	up	1.619291	up
1.62167	up	1.618693	up
1.391181	up	1.618352	up
1.422441	up	-1.61815	down
-1.40452	down	-1.61806	down
1.003569	up	-1.61801	down
-1.23803	down	-1.61763	down
-1.92139	down	-1.61734	down
1.44785	up	-1.61726	down
3.251127	up	1.617114	up
1.147504	up	-1.61688	down
1.207475	up	1.616863	up
-1.04618	down	-1.61673	down
-4.56926	down	1.616394	up
-1.88911	down	-1.61621	down
1.286393	up	1.616101	up
-1.37198	down	-1.6159	down
-1.34134	down	-1.61586	down
17.16657	up	1.61585	up
1.394888	up	-1.61576	down
-1.21388	down	-1.61575	down
2.833012	up	1.615653	up
-1.2708	down	-1.61549	down
1.719362	up	1.615171	up
-1.64858	down	-1.61512	down
-1.08339	down	-1.6146	down
3.29471	up	1.614365	up
-1.32332	down	-1.6142	down
-1.73507	down	-1.614	down
1.835969	up	1.613963	up
-2.15236	down	1.613902	up
1.068292	up	1.613886	up
-1.22963	down	-1.61387	down
-1.55241	down	-1.61375	down
-1.4508	down	-1.61372	down
-1.29858	down	-1.61371	down
-1.7494	down	-1.61365	down
-1.11291	down	-1.61337	down
-1.44126	down	-1.61315	down
-1.4353	down	-1.61267	down
1.610947	up	1.612611	up
1.863863	up	1.612529	up
-1.58507	down	-1.61239	down
2.130889	up	1.612265	up
-1.18307	down	1.612114	up
1.830473	up	1.612092	up
-1.40622	down	-1.61206	down

-3.1085	down	-1.61172	down
-1.00308	down	1.611621	up
3.12866	up	1.611542	up
-1.40334	down	-1.61151	down
-1.10083	down	-1.61101	down
1.038648	up	1.61071	up
1.552748	up	1.610523	up
-1.8262	down	-1.61049	down
-1.19926	down	-1.61006	down
-1.70836	down	-1.60998	down
-1.29597	down	-1.60989	down
1.160293	up	1.609884	up
1.201692	up	-1.60958	down
-1.69847	down	-1.60951	down
1.38657	up	1.609503	up
-1.27733	down	-1.6095	down
3.744711	up	1.609347	up
1.464802	up	1.609267	up
2.210269	up	-1.60925	down
3.341206	up	1.609226	up
1.350757	up	1.6092	up
3.212926	up	1.608799	up
-1.73118	down	-1.6086	down
-1.4698	down	-1.60855	down
1.427655	up	1.608524	up
-1.9509	down	1.608303	up
-1.26685	down	-1.60823	down
-1.34388	down	-1.6079	down
-1.29216	down	1.607738	up
1.714668	up	1.607724	up
-2.08951	down	1.607063	up
1.696649	up	1.606934	up
-1.26691	down	-1.60637	down
-1.78254	down	-1.6063	down
1.73098	up	-1.60625	down
-1.68479	down	1.606166	up
-1.49335	down	-1.60595	down
3.545202	up	1.605945	up
1.613476	up	1.605575	up
-2.16462	down	1.605388	up
-1.29337	down	1.605229	up
1.980651	up	1.605066	up
1.45213	up	1.604826	up
2.564795	up	1.604779	up
-1.3046	down	-1.60445	down
1.329279	up	-1.60384	down
-1.64793	down	-1.6031	down

-1.56429	down	-1.60296	down
-2.1107	down	1.602864	up
1.717361	up	1.60275	up
1.643545	up	-1.60262	down
1.487293	up	1.602475	up
-1.25016	down	-1.60223	down
-1.51015	down	-1.60222	down
-1.27182	down	-1.60195	down
-1.35672	down	-1.60182	down
-1.42512	down	-1.60152	down
-2.02771	down	1.601387	up
1.594242	up	1.601269	up
1.390638	up	1.601226	up
-1.30955	down	1.601063	up
1.878987	up	1.600974	up
-1.22324	down	-1.60097	down
-1.58681	down	-1.60093	down
1.630661	up	-1.60087	down
1.288264	up	1.600487	up
-1.64877	down	-1.60028	down
-1.50433	down	-1.59976	down
2.168625	up	1.599602	up
1.179116	up	-1.59955	down
1.244067	up	-1.5994	down
1.56912	up	1.599292	up
1.706002	up	1.599057	up
-1.60143	down	-1.59888	down
-1.65574	down	1.598451	up
-1.30803	down	-1.5979	down
-1.01481	down	1.597707	up
1.319025	up	-1.59757	down
1.345713	up	1.597207	up
1.772582	up	1.597017	up
-1.72996	down	-1.597	down
-1.52501	down	-1.59678	down
-2.05871	down	-1.59629	down
2.16039	up	1.596041	up
1.172198	up	-1.5959	down
-2.01914	down	-1.59562	down
-1.66275	down	-1.59551	down
-1.29467	down	-1.59547	down
-1.23691	down	1.595331	up
-1.17648	down	-1.59522	down
1.665918	up	1.595084	up
1.472131	up	1.594985	up
-1.20847	down	-1.59488	down
-1.41205	down	-1.59484	down

1.433102	up	1.59477	up
-1.48411	down	-1.5946	down
-1.28654	down	-1.59458	down
-1.32607	down	-1.59434	down
-2.30725	down	-1.59421	down
-1.04976	down	-1.59411	down
-1.31605	down	-1.59396	down
1.109113	up	-1.59349	down
1.026702	up	-1.5932	down
3.060922	up	1.592943	up
-1.10309	down	1.592891	up
1.494806	up	1.592709	up
1.570146	up	1.592228	up
-1.36374	down	-1.59204	down
1.95549	up	1.591858	up
-2.99906	down	-1.59176	down
-1.82669	down	-1.59143	down
-1.45111	down	-1.59141	down
-3.44383	down	-1.59115	down
-1.16567	down	-1.59104	down
-1.31484	down	-1.59086	down
-1.26322	down	-1.5908	down
1.628287	up	-1.59078	down
-1.28366	down	-1.59064	down
-1.40209	down	-1.59063	down
-1.98914	down	-1.59021	down
3.234552	up	-1.58995	down
1.507708	up	-1.58994	down
1.355055	up	1.589929	up
-1.32678	down	-1.58969	down
-2.45986	down	1.589499	up
-1.65912	down	-1.58945	down
2.552969	up	1.58899	up
2.378319	up	-1.58869	down
-1.17221	down	-1.58859	down
1.274419	up	1.588382	up
-1.33865	down	-1.58819	down
-1.332	down	1.587746	up
-1.32149	down	-1.58773	down
2.142114	up	1.587598	up
-1.34038	down	-1.58741	down
-1.30762	down	-1.58727	down
1.864906	up	1.587227	up
4.235598	up	1.587151	up
1.304885	up	1.586848	up
1.759254	up	1.586478	up
1.89472	up	1.586197	up

-1.67939	down	-1.58557	down
1.498459	up	1.585498	up
-1.07512	down	-1.58539	down
-2.20296	down	-1.58531	down
-1.76635	down	-1.58517	down
1.553492	up	1.585097	up
-1.60174	down	-1.58479	down
-1.40609	down	-1.58477	down
-1.34288	down	-1.5843	down
-1.52557	down	-1.58402	down
1.399504	up	1.583686	up
1.7332	up	1.583649	up
-1.43911	down	-1.58352	down
-1.30311	down	-1.58335	down
-1.10683	down	-1.58328	down
-1.44597	down	-1.58321	down
1.703395	up	1.582966	up
1.686829	up	1.58264	up
2.894714	up	1.582491	up
-1.23684	down	-1.58237	down
-1.50121	down	-1.58219	down
-1.77435	down	-1.58203	down
-1.45066	down	-1.58106	down
-1.42546	down	1.580589	up
-2.29429	down	-1.58057	down
-1.29203	down	-1.58053	down
1.832398	up	1.580502	up
1.939005	up	1.580354	up
-1.08331	down	1.580257	up
-1.38039	down	-1.57984	down
1.397805	up	1.579835	up
-1.37526	down	-1.57974	down
-1.64351	down	-1.5791	down
1.425509	up	1.579094	up
-2.17951	down	-1.57876	down
-1.34227	down	-1.57873	down
1.989322	up	1.578715	up
1.812109	up	-1.57848	down
1.32247	up	1.578036	up
-1.40491	down	-1.57789	down
1.590663	up	1.577815	up
1.037527	up	-1.57768	down
-1.53609	down	-1.57747	down
-1.59381	down	1.577471	up
-1.27155	down	-1.57736	down
-1.38494	down	-1.57722	down
-1.28828	down	-1.57721	down

-1.13775	down	-1.5768	down
2.176949	up	-1.5764	down
2.051883	up	-1.57611	down
-2.00677	down	-1.57599	down
2.105391	up	1.575914	up
1.634242	up	-1.5755	down
1.434055	up	1.575333	up
-2.79411	down	-1.5753	down
-1.37265	down	-1.57523	down
-2.07267	down	-1.57509	down
1.445627	up	-1.57508	down
-1.78849	down	-1.57494	down
2.073682	up	1.57494	up
-1.37287	down	-1.5748	down
-1.65146	down	-1.57471	down
1.16947	up	-1.57468	down
2.369986	up	1.574669	up
1.00449	up	-1.57456	down
1.122784	up	-1.57452	down
1.1805	up	1.574419	up
-1.6964	down	-1.57431	down
-1.53598	down	-1.57398	down
-1.95281	down	-1.57389	down
1.301885	up	1.573861	up
1.371674	up	1.573807	up
4.970457	up	1.573698	up
1.10774	up	1.573623	up
2.114581	up	-1.57336	down
-1.60429	down	-1.57323	down
1.493934	up	1.572812	up
-1.48941	down	-1.57264	down
-1.89451	down	-1.57254	down
-1.3147	down	-1.57209	down
1.572138	up	1.571239	up
-1.13982	down	-1.57095	down
-1.39767	down	1.570265	up
-1.88145	down	-1.57019	down
2.172841	up	-1.56996	down
6.972023	up	1.569563	up
-1.38976	down	-1.56938	down
-2.61917	down	1.569318	up
-1.42225	down	-1.56927	down
-1.19076	down	1.569259	up
2.017169	up	1.568946	up
-1.40256	down	-1.56887	down
1.824778	up	1.568737	up
-1.8515	down	-1.56864	down

1.156074	up	-1.56862	down
-1.28406	down	-1.56847	down
1.532468	up	1.568223	up
-1.29677	down	-1.56815	down
-1.71822	down	-1.5681	down
1.18448	up	-1.5679	down
1.320978	up	1.567414	up
1.128667	up	-1.56735	down
-1.2985	down	-1.56722	down
-1.31372	down	-1.56704	down
1.244299	up	1.56601	up
1.408119	up	-1.56582	down
-1.32277	down	-1.56502	down
1.416364	up	1.564745	up
1.474349	up	1.564327	up
-1.37521	down	-1.56426	down
2.293701	up	1.564205	up
-1.84811	down	1.564142	up
1.875211	up	1.564012	up
-1.38005	down	-1.56397	down
-1.32544	down	-1.56374	down
1.283517	up	1.563615	up
2.575947	up	-1.56291	down
-2.12312	down	-1.56266	down
1.085651	up	1.562469	up
2.367676	up	1.562427	up
1.419156	up	-1.56242	down
1.574603	up	1.562416	up
4.044466	up	1.562415	up
1.686189	up	1.562255	up
1.399673	up	1.56218	up
-1.40963	down	-1.56212	down
-1.02807	down	-1.56154	down
4.130349	up	1.561528	up
-1.25092	down	-1.56151	down
-1.30251	down	-1.56129	down
1.835748	up	-1.56114	down
-2.01018	down	1.561052	up
1.082812	up	-1.5606	down
-1.31809	down	-1.56053	down
-1.38391	down	-1.56045	down
1.518011	up	1.560441	up
-1.34267	down	-1.56017	down
-9.5896	down	1.560126	up
1.335493	up	1.559833	up
3.092495	up	1.558468	up
-1.56937	down	-1.55782	down

1.580681	up	1.557746	up
-1.36883	down	-1.55743	down
-1.93814	down	-1.55714	down
-1.49194	down	-1.55698	down
1.667199	up	-1.55688	down
-1.73942	down	-1.5568	down
1.950292	up	1.556673	up
-1.93449	down	-1.55651	down
-4.33817	down	1.556466	up
-1.38145	down	1.556284	up
-1.63037	down	-1.55613	down
2.691233	up	1.556085	up
-1.30808	down	-1.55608	down
-1.2879	down	-1.55599	down
1.67929	up	1.55526	up
-2.21031	down	-1.55494	down
8.022327	up	1.554762	up
-1.53719	down	-1.55466	down
2.568292	up	1.554627	up
-1.86983	down	-1.55453	down
-1.33002	down	-1.5545	down
-1.33745	down	-1.5544	down
-3.27325	down	-1.55388	down
1.731945	up	-1.55388	down
3.085138	up	-1.55376	down
-1.33952	down	-1.55349	down
-1.31188	down	-1.55277	down
-1.49352	down	-1.55268	down
-1.46028	down	-1.55259	down
-1.40911	down	-1.55218	down
2.716204	up	1.552123	up
-1.22551	down	-1.55183	down
1.917022	up	1.551717	up
-1.29598	down	-1.5515	down
-1.41063	down	-1.55144	down
1.301169	up	1.551424	up
-1.02188	down	-1.55129	down
-1.9523	down	1.551273	up
1.580043	up	1.551109	up
1.435609	up	-1.55029	down
-1.6122	down	-1.55004	down
1.344946	up	1.549429	up
1.354699	up	1.549392	up
-1.33814	down	-1.54936	down
-1.63419	down	-1.54931	down
-1.16503	down	1.549307	up
-1.87027	down	-1.54929	down

-1.4221	down	-1.54925	down
1.297818	up	1.54921	up
-1.01704	down	-1.54911	down
1.182031	up	-1.54901	down
1.129271	up	-1.54883	down
1.200587	up	-1.54879	down
1.454553	up	-1.54878	down
-1.47453	down	-1.54867	down
1.081603	up	1.5483	up
-1.32019	down	-1.54808	down
1.543962	up	1.547958	up
-1.51172	down	-1.54793	down
-1.4601	down	-1.54778	down
-1.29479	down	-1.54744	down
1.495398	up	-1.54712	down
-1.76414	down	-1.54694	down
1.443746	up	1.546518	up
-1.42727	down	-1.54649	down
1.754606	up	-1.54598	down
1.920377	up	-1.54571	down
1.416561	up	1.545564	up
-1.70437	down	1.545025	up
-1.30671	down	-1.54497	down
-6.10273	down	-1.5448	down
1.819191	up	1.544739	up
-1.31162	down	-1.54462	down
-1.56283	down	-1.54461	down
-1.21154	down	-1.54449	down
-2.27411	down	1.544356	up
1.46745	up	-1.54435	down
3.006455	up	-1.54429	down
-2.13517	down	-1.544	down
1.44439	up	-1.54394	down
-1.08702	down	-1.54391	down
-1.30704	down	-1.54361	down
-3.51985	down	1.54356	up
1.454597	up	1.543407	up
-15.8403	down	1.542154	up
1.895856	up	-1.54211	down
-1.60285	down	-1.54202	down
1.475853	up	1.541458	up
-1.33471	down	-1.54134	down
-1.38736	down	-1.5413	down
1.217417	up	-1.5408	down
-1.33743	down	1.540643	up
-1.33196	down	-1.5406	down
2.682724	up	1.540506	up

1.166916	up	1.540296	up
6.571396	up	1.540281	up
-1.46723	down	-1.54023	down
-1.5643	down	-1.54009	down
-1.40824	down	-1.5397	down
-1.23928	down	-1.53895	down
1.877711	up	1.538261	up
-1.56221	down	-1.53822	down
1.400574	up	-1.53803	down
1.546269	up	1.537723	up
-2.20374	down	1.537712	up
-1.55593	down	-1.53698	down
1.381545	up	-1.53668	down
-3.00125	down	1.536501	up
-1.31618	down	-1.53599	down
-1.94524	down	-1.53569	down
3.044472	up	1.53568	up
-2.26189	down	1.53567	up
-1.7902	down	-1.53528	down
-1.53493	down	-1.5351	down
2.469859	up	1.534825	up
-1.21329	down	-1.53475	down
-1.86275	down	-1.53442	down
-1.2731	down	-1.5343	down
1.354603	up	1.534261	up
-1.40303	down	-1.53426	down
-1.36477	down	-1.53399	down
1.382553	up	-1.53364	down
-1.1775	down	-1.53335	down
-1.9456	down	-1.53311	down
-1.88959	down	1.532822	up
-3.07524	down	1.532759	up
1.742515	up	-1.53274	down
-1.37137	down	-1.53268	down
1.570912	up	1.532616	up
-2.49775	down	1.532532	up
1.131025	up	-1.53247	down
1.25388	up	1.532157	up
-1.60626	down	-1.53214	down
-2.49245	down	1.53184	up
-1.3219	down	-1.53167	down
1.449903	up	-1.53165	down
2.216662	up	1.531494	up
1.351707	up	1.531328	up
2.073256	up	-1.53127	down
2.040783	up	1.531262	up
1.798582	up	-1.53117	down

1.333006	up	1.530676	up
1.457655	up	1.530594	up
1.682753	up	1.530491	up
-2.03652	down	-1.53027	down
-1.38638	down	-1.53014	down
-1.78476	down	-1.52971	down
1.060305	up	1.529694	up
-1.78488	down	-1.52964	down
2.573989	up	1.529611	up
-2.61208	down	-1.52952	down
1.339876	up	1.529484	up
1.304479	up	1.529466	up
1.465313	up	1.529347	up
-2.07674	down	-1.52929	down
1.265243	up	-1.52905	down
-1.42853	down	-1.5288	down
-1.59749	down	-1.52873	down
1.452447	up	1.528711	up
-1.68393	down	-1.52834	down
-1.31524	down	-1.52832	down
3.089983	up	1.528148	up
-1.49089	down	-1.5281	down
-1.74653	down	-1.52803	down
-1.34422	down	-1.5279	down
10.76718	up	1.527888	up
1.596004	up	1.527244	up
1.990504	up	1.527198	up
1.313386	up	-1.52693	down
1.54472	up	1.526931	up
-1.35664	down	-1.52688	down
1.296173	up	1.526734	up
-1.35007	down	-1.52664	down
1.038521	up	-1.52596	down
-2.24515	down	-1.52593	down
-1.68136	down	-1.52579	down
-1.63253	down	-1.52533	down
3.232735	up	1.524977	up
1.47439	up	1.524775	up
-1.12437	down	1.524743	up
2.13527	up	-1.52471	down
1.992591	up	-1.52464	down
1.4489	up	-1.52459	down
-1.54863	down	-1.52434	down
-6.07971	down	-1.52378	down
1.757341	up	1.523734	up
-1.34398	down	-1.52355	down
-1.84781	down	-1.52335	down

-1.33347	down	-1.52306	down
1.216676	up	1.522991	up
1.20335	up	1.522904	up
-1.51791	down	-1.52274	down
-2.00523	down	1.522046	up
-2.22631	down	1.52194	up
-2.25458	down	-1.52168	down
-1.44905	down	-1.52139	down
2.193046	up	1.521253	up
1.434178	up	1.521227	up
1.498983	up	1.520997	up
-1.60531	down	-1.52083	down
-1.40083	down	-1.52079	down
1.371982	up	1.520638	up
2.975638	up	1.520432	up
-2.05365	down	-1.52024	down
1.762239	up	1.52021	up
1.1137	up	1.519988	up
-1.42709	down	-1.5199	down
-1.07746	down	-1.51989	down
-1.34169	down	-1.51972	down
-1.42436	down	-1.5197	down
-1.84297	down	-1.51961	down
3.216505	up	1.519573	up
-1.38254	down	-1.5195	down
1.033572	up	-1.51937	down
-1.76771	down	-1.51929	down
2.799809	up	-1.51916	down
-1.85844	down	-1.51901	down
-1.75039	down	-1.51894	down
-1.32366	down	-1.51851	down
-1.65507	down	-1.51836	down
-2.07338	down	1.518128	up
-1.59516	down	-1.51787	down
-1.39092	down	-1.51784	down
1.35916	up	-1.51772	down
1.55138	up	1.517677	up
1.577378	up	-1.51742	down
1.957008	up	-1.5174	down
2.611396	up	-1.51715	down
-1.1839	down	-1.51693	down
-1.36542	down	-1.51648	down
1.526885	up	1.516351	up
-1.84393	down	1.51622	up
1.335627	up	-1.51604	down
-1.62006	down	-1.51601	down
-1.52196	down	-1.51574	down

-1.4563	down	-1.51564	down
-1.47102	down	-1.51542	down
-2.5266	down	1.51525	up
-1.19874	down	-1.51503	down
-1.83218	down	1.514809	up
-1.50888	down	1.514203	up
-1.85544	down	-1.51417	down
-1.51302	down	1.513866	up
1.689656	up	-1.51381	down
-2.16255	down	-1.51361	down
-1.35196	down	-1.51358	down
1.100302	up	-1.51357	down
1.598468	up	1.513499	up
-1.37541	down	-1.51311	down
-1.64533	down	-1.5131	down
1.298336	up	-1.5131	down
-1.77104	down	-1.51308	down
1.393676	up	-1.51288	down
-1.23335	down	-1.51267	down
-1.73354	down	-1.51244	down
-1.17774	down	-1.51243	down
-1.38885	down	-1.51241	down
-1.35049	down	-1.51219	down
-1.47259	down	-1.51191	down
-2.35134	down	-1.51186	down
1.776004	up	1.511516	up
1.729342	up	1.511429	up
1.233257	up	1.511275	up
1.392959	up	-1.51124	down
-1.34674	down	-1.51122	down
-1.60496	down	-1.51118	down
1.222395	up	1.511174	up
-1.73926	down	-1.51045	down
-1.45468	down	-1.51028	down
1.121198	up	1.510077	up
1.717353	up	1.510031	up
-1.15003	down	-1.51001	down
-1.237	down	-1.50977	down
-1.52188	down	-1.50976	down
-1.24287	down	-1.50961	down
-1.08734	down	-1.5096	down
-2.60408	down	1.509192	up
-1.2221	down	-1.50916	down
1.307175	up	-1.50916	down
-2.11596	down	1.509119	up
-1.20702	down	-1.50911	down
-1.45995	down	-1.50882	down

-1.83847	down	-1.50877	down
-1.76208	down	1.508571	up
-1.46715	down	-1.50846	down
-1.11307	down	-1.50769	down
1.277633	up	-1.5074	down
2.45404	up	1.507329	up
-2.12815	down	-1.5072	down
1.063994	up	-1.50686	down
-2.03553	down	-1.50679	down
-1.46722	down	-1.50673	down
2.004462	up	-1.50671	down
-1.6708	down	-1.50655	down
-1.35498	down	-1.50649	down
2.571129	up	-1.50648	down
3.465883	up	1.506268	up
3.260347	up	-1.5062	down
-1.49461	down	-1.50613	down
6.493362	up	1.505706	up
-1.54477	down	-1.50533	down
1.55378	up	1.505304	up
1.067998	up	1.50523	up
-1.75812	down	1.505192	up
-1.60616	down	-1.50513	down
1.519935	up	1.504706	up
3.059435	up	1.50468	up
-2.00031	down	1.504645	up
2.802345	up	1.50462	up
1.159443	up	1.504491	up
1.552511	up	1.504443	up
-1.30658	down	-1.50426	down
1.70748	up	1.503928	up
-2.16487	down	-1.50385	down
-1.1296	down	1.5032	up
2.002638	up	1.503056	up
1.146986	up	-1.50285	down
2.015808	up	1.502699	up
-1.26721	down	-1.50248	down
1.770613	up	1.502425	up
1.992402	up	1.502026	up
-1.19854	down	-1.50192	down
-1.41739	down	-1.50186	down
1.050024	up	-1.50183	down
1.117032	up	1.501572	up
-4.73997	down	1.501475	up
-2.95481	down	1.501355	up
-1.59661	down	-1.50133	down
1.779324	up	1.501237	up

-1.35186	down	-1.50117	down
2.375854	up	1.500891	up
-1.45108	down	1.500775	up
-1.41283	down	-1.50062	down
1.36557	up	1.500477	up
-1.39259	down	-1.50046	down
1.602605	up	1.500438	up
1.397941	up	1.500354	up
1.721351	up	-1.49991	down
2.916524	up	1.499789	up
-1.53116	down	-1.4997	down
-1.79885	down	-1.49966	down
-1.24717	down	-1.49943	down
-1.77923	down	-1.49937	down
-1.36728	down	-1.49913	down
-1.48563	down	-1.49901	down
-1.38833	down	-1.49901	down
-1.81722	down	-1.49896	down
-1.14403	down	-1.49881	down
2.065752	up	1.498661	up
-1.15676	down	1.49841	up
2.489843	up	1.498314	up
1.186443	up	1.498177	up
-1.78677	down	-1.49811	down
1.261042	up	1.497869	up
-1.74263	down	-1.49779	down
1.363659	up	-1.49717	down
-1.60899	down	-1.49704	down
2.601884	up	1.496651	up
3.188615	up	1.496301	up
1.581546	up	1.496166	up
1.015234	up	-1.49611	down
2.005807	up	-1.496	down
-1.48129	down	-1.49583	down
-2.00108	down	-1.49571	down
1.631654	up	1.495667	up
-1.35835	down	-1.49554	down
-1.24078	down	-1.4951	down
-1.76536	down	-1.495	down
2.502816	up	-1.49487	down
1.966585	up	1.494635	up
-2.25054	down	-1.49445	down
1.456515	up	-1.49442	down
3.104652	up	1.494394	up
-1.3493	down	-1.49424	down
2.378643	up	1.494132	up
1.498703	up	1.493967	up

2.866967	up	1.493819	up
2.059228	up	-1.49341	down
1.518919	up	1.493247	up
2.257701	up	1.49324	up
2.043719	up	1.49311	up
-1.37079	down	-1.49273	down
-1.37137	down	-1.4926	down
-2.64168	down	1.492575	up
-1.31584	down	-1.49255	down
-1.92553	down	-1.4921	down
3.141887	up	1.491627	up
-1.74866	down	-1.49146	down
1.366073	up	-1.49143	down
-1.59976	down	-1.49116	down
-1.19827	down	-1.49097	down
-1.47398	down	-1.49076	down
2.323306	up	-1.49067	down
-1.44124	down	-1.49063	down
-1.48418	down	-1.49055	down
-1.45472	down	-1.4905	down
-1.49824	down	-1.49035	down
-1.45516	down	-1.49015	down
2.046037	up	1.490097	up
-1.05431	down	-1.49002	down
2.961757	up	1.489873	up
1.558272	up	1.489778	up
-1.61436	down	-1.48964	down
-1.6679	down	-1.48957	down
-1.31969	down	-1.48927	down
-1.28268	down	1.489131	up
1.262996	up	1.489129	up
1.916735	up	-1.48891	down
-2.81357	down	1.488703	up
1.503104	up	1.488411	up
-1.97354	down	-1.48822	down
-1.53019	down	-1.48748	down
2.311817	up	1.487155	up
1.428947	up	-1.48697	down
-1.75512	down	-1.48672	down
-2.38137	down	1.48613	up
-1.69373	down	-1.486	down
1.282496	up	-1.48569	down
2.933697	up	1.484606	up
-1.2875	down	-1.48419	down
-1.65688	down	-1.484	down
-1.97528	down	-1.484	down
1.194813	up	-1.48382	down

-1.58627	down	-1.48374	down
-2.00064	down	-1.48371	down
1.579851	up	1.483651	up
-1.05809	down	1.483573	up
-1.40306	down	1.483572	up
2.294159	up	1.48355	up
1.240775	up	1.48355	up
1.646957	up	1.483504	up
1.070913	up	1.48336	up
1.355326	up	1.483354	up
-1.40849	down	-1.48302	down
1.710007	up	-1.48301	down
-2.75279	down	1.48297	up
1.416853	up	1.482821	up
-2.3065	down	1.482768	up
1.542985	up	1.482647	up
1.36825	up	1.482402	up
1.339326	up	1.482354	up
8.114435	up	1.48187	up
-1.335	down	-1.48143	down
-1.42676	down	-1.4814	down
2.303529	up	1.481201	up
-1.39517	down	-1.4812	down
1.558727	up	-1.48108	down
3.086424	up	1.480923	up
-1.38022	down	-1.48059	down
-1.52115	down	-1.48023	down
1.261631	up	1.480079	up
-1.22481	down	-1.47993	down
1.514647	up	1.479798	up
-1.40468	down	-1.47975	down
-1.40537	down	-1.47972	down
1.361244	up	-1.4797	down
-1.73028	down	1.479199	up
1.025563	up	-1.47912	down
-1.38577	down	-1.47899	down
1.985772	up	1.478897	up
-1.38011	down	-1.47866	down
1.508076	up	1.478401	up
1.384439	up	1.478296	up
-1.67741	down	-1.47813	down
-1.59455	down	-1.4781	down
1.387903	up	1.477956	up
-1.00651	down	1.477635	up
-1.57106	down	-1.47762	down
1.787782	up	1.477363	up
1.379771	up	1.476803	up

-1.26208	down	-1.47625	down
1.338015	up	1.476081	up
1.452397	up	-1.47606	down
5.164491	up	1.475999	up
1.932755	up	-1.47583	down
-1.85425	down	-1.47547	down
-2.25025	down	-1.47494	down
-2.09789	down	-1.47463	down
-1.1129	down	-1.47434	down
-2.11371	down	-1.47408	down
8.579556	up	1.474003	up
1.165942	up	1.473726	up
1.398964	up	1.473345	up
-1.44908	down	-1.47313	down
-1.20069	down	-1.47281	down
1.721771	up	1.472749	up
1.032925	up	-1.47244	down
-1.5386	down	-1.47237	down
-2.36853	down	1.472017	up
1.692857	up	1.471713	up
-1.30769	down	-1.47041	down
-1.72524	down	-1.47028	down
-1.57369	down	-1.47026	down
-1.37385	down	-1.46995	down
1.024478	up	1.469875	up
-1.33404	down	-1.46937	down
1.494227	up	1.469304	up
2.799314	up	-1.46921	down
-1.52369	down	-1.46919	down
2.238436	up	1.468771	up
1.46589	up	1.467588	up
-1.7049	down	-1.46747	down
-1.67013	down	-1.46717	down
2.213865	up	1.466886	up
-1.13585	down	1.466549	up
1.471979	up	-1.46652	down
1.570866	up	-1.46602	down
-1.15462	down	-1.46588	down
-1.92069	down	-1.46574	down
-1.33245	down	-1.4657	down
-2.88245	down	-1.46541	down
-1.4741	down	-1.46493	down
-1.50894	down	-1.46466	down
-1.39439	down	-1.46463	down
2.069364	up	1.464395	up
1.663752	up	1.46439	up
-1.63252	down	1.464173	up

-1.45297	down	1.464009	up
1.150802	up	-1.4635	down
2.222366	up	1.46346	up
1.79894	up	-1.46346	down
-1.1856	down	-1.46319	down
-1.66008	down	-1.46314	down
-1.8109	down	-1.46275	down
-1.56954	down	-1.46217	down
-1.60441	down	-1.46188	down
-1.3465	down	-1.46172	down
2.108939	up	-1.46166	down
-1.59449	down	-1.46155	down
1.112158	up	1.460868	up
-1.92139	down	1.460679	up
-1.43833	down	-1.46012	down
2.174888	up	1.460083	up
2.045174	up	-1.45975	down
1.837105	up	1.459727	up
1.221795	up	1.459658	up
-1.78773	down	-1.45829	down
-1.57576	down	-1.45805	down
1.415645	up	1.457988	up
1.552138	up	1.45797	up
1.459712	up	1.457738	up
-2.40098	down	-1.45773	down
9.840168	up	1.457703	up
-2.02026	down	-1.45769	down
-1.24358	down	-1.4576	down
-1.83266	down	-1.45717	down
-1.39766	down	-1.45712	down
-1.45492	down	-1.45695	down
3.118377	up	1.456909	up
1.939528	up	1.45645	up
1.526754	up	1.456294	up
-1.94515	down	1.456206	up
-1.25256	down	-1.45583	down
-1.43966	down	-1.45581	down
-1.56043	down	-1.45576	down
-1.78385	down	1.45563	up
1.636372	up	1.455389	up
1.407871	up	1.455301	up
-1.78718	down	-1.45528	down
-1.85404	down	-1.45487	down
-1.60584	down	-1.45477	down
-1.61924	down	-1.45468	down
1.859137	up	1.454652	up
1.592923	up	1.454506	up

-1.2529	down	-1.45445	down
-1.62093	down	-1.45429	down
-1.32801	down	1.454275	up
-1.74154	down	-1.45382	down
-1.52387	down	1.453762	up
1.824056	up	1.453683	up
1.249919	up	-1.45333	down
-4.19409	down	1.453263	up
-1.16335	down	1.452634	up
-1.36755	down	-1.45239	down
3.100206	up	1.452385	up
1.276389	up	1.45233	up
-1.44614	down	-1.45214	down
1.577778	up	1.451961	up
1.263267	up	1.451957	up
-1.25322	down	-1.45195	down
1.070024	up	-1.45183	down
1.643816	up	1.451807	up
1.726563	up	-1.45175	down
-1.55402	down	-1.45165	down
-1.45729	down	-1.45134	down
1.740391	up	1.451029	up
-1.09381	down	-1.45102	down
1.789353	up	1.450801	up
-1.73294	down	-1.45062	down
1.335384	up	-1.45057	down
1.298006	up	1.450515	up
1.829392	up	1.450376	up
1.516789	up	1.450359	up
-1.81333	down	-1.45021	down
1.407364	up	1.450013	up
1.335231	up	1.44989	up
2.895867	up	-1.44972	down
-1.83712	down	-1.44926	down
-1.42567	down	-1.44888	down
-1.77674	down	-1.44883	down
-1.53952	down	-1.44861	down
1.385885	up	1.448241	up
-1.56364	down	-1.44815	down
-1.81404	down	-1.44806	down
2.308079	up	1.447983	up
-2.43157	down	1.447844	up
1.539856	up	-1.44767	down
-1.18481	down	-1.44761	down
-1.2792	down	-1.44746	down
-1.40112	down	-1.44746	down
-1.58964	down	-1.44743	down

-1.08426	down	1.447008	up
-1.28452	down	-1.44695	down
1.332686	up	-1.44694	down
-1.07922	down	-1.44687	down
-1.49587	down	-1.44675	down
-1.40919	down	-1.44634	down
-1.29607	down	-1.44617	down
-1.41619	down	-1.44615	down
2.915182	up	1.445884	up
1.00613	up	-1.44584	down
1.451493	up	-1.44572	down
1.672412	up	1.445519	up
-1.78832	down	1.444888	up
-1.64119	down	-1.44479	down
-1.15756	down	-1.44452	down
-2.01933	down	-1.44448	down
1.111102	up	-1.44427	down
1.423639	up	1.444019	up
3.106281	up	1.443996	up
-1.38544	down	-1.44387	down
1.825198	up	1.443802	up
1.498409	up	-1.44376	down
-1.4826	down	-1.44351	down
-1.41469	down	-1.44317	down
3.771711	up	-1.44298	down
-1.40631	down	-1.44286	down
-1.46551	down	1.442595	up
1.075063	up	-1.44247	down
2.417829	up	-1.44226	down
-1.53702	down	-1.44221	down
1.447927	up	-1.4422	down
-3.36944	down	1.442064	up
2.296792	up	1.441847	up
1.610305	up	1.441681	up
-1.01976	down	-1.44165	down
-1.3561	down	-1.44165	down
-1.922	down	-1.44152	down
1.553248	up	-1.44148	down
1.42176	up	1.441374	up
-1.98858	down	-1.44127	down
-1.40473	down	-1.44124	down
-2.28767	down	1.44106	up
-1.4306	down	-1.44101	down
1.704465	up	1.440823	up
-1.58143	down	-1.44081	down
1.161899	up	1.44079	up
-1.1888	down	-1.44063	down

-1.74676	down	-1.44059	down
-1.04186	down	-1.44034	down
2.118133	up	1.440002	up
1.217946	up	1.439956	up
-1.85114	down	-1.43992	down
-1.50609	down	-1.43918	down
2.30408	up	-1.43908	down
1.0961	up	-1.43881	down
1.390132	up	1.438741	up
1.224284	up	-1.43857	down
1.413483	up	1.438184	up
1.403318	up	-1.43788	down
1.11505	up	1.437854	up
-1.86014	down	-1.4376	down
-2.0734	down	1.437479	up
1.010195	up	1.437453	up
-1.09379	down	-1.43726	down
3.480004	up	1.437226	up
-1.41766	down	-1.43699	down
-1.40873	down	-1.43671	down
4.136747	up	1.436634	up
-1.55496	down	-1.43653	down
1.069104	up	-1.43601	down
10.93547	up	1.436005	up
-1.00818	down	-1.43598	down
-1.24972	down	-1.43596	down
-1.66922	down	-1.4358	down
-2.03919	down	1.435589	up
-1.0267	down	-1.4351	down
-1.3326	down	-1.43446	down
-1.10237	down	1.434043	up
1.015088	up	-1.43391	down
1.539509	up	1.433785	up
-1.69754	down	-1.43347	down
-2.4785	down	1.433445	up
1.658631	up	1.433355	up
1.87618	up	-1.43302	down
1.074187	up	-1.43298	down
-2.52083	down	-1.43287	down
1.639444	up	1.4327	up
-1.46894	down	1.432621	up
-1.64796	down	-1.43252	down
1.042754	up	-1.43224	down
-2.06117	down	1.432079	up
1.718598	up	-1.43162	down
1.777992	up	-1.43123	down
-1.40197	down	-1.43115	down

-1.23462	down	-1.43111	down
-2.41719	down	-1.43099	down
1.649937	up	1.430596	up
2.197	up	-1.43019	down
-1.54525	down	-1.43018	down
-1.11406	down	-1.43012	down
-1.48511	down	-1.43011	down
2.268459	up	1.429841	up
1.528023	up	1.429609	up
6.475095	up	1.429573	up
-2.14108	down	-1.42952	down
-2.26556	down	-1.42949	down
-1.40642	down	-1.42943	down
1.498746	up	1.428956	up
1.329925	up	-1.42885	down
1.568591	up	1.428799	up
-1.92362	down	1.428483	up
-1.4273	down	-1.42808	down
-1.55157	down	-1.42806	down
-1.47216	down	-1.42787	down
-1.12174	down	-1.42776	down
-1.47293	down	-1.42766	down
-2.0119	down	1.427525	up
-2.33736	down	1.427502	up
1.515979	up	1.42747	up
1.032224	up	-1.42742	down
-1.76533	down	-1.4274	down
-2.48072	down	-1.42692	down
1.128297	up	1.426405	up
-2.43626	down	-1.42633	down
2.704193	up	1.426196	up
-1.41325	down	-1.4258	down
1.547579	up	1.425239	up
1.577753	up	1.424973	up
-1.08198	down	-1.42489	down
-1.60078	down	-1.42477	down
-1.89897	down	1.424528	up
-1.49685	down	-1.42433	down
2.656725	up	1.424173	up
2.504991	up	-1.42407	down
6.183751	up	1.423858	up
-1.26016	down	-1.42381	down
1.986899	up	1.423594	up
2.44852	up	-1.42329	down
-1.27329	down	-1.42329	down
-1.84153	down	-1.42276	down
2.099996	up	1.42263	up

-1.30873	down	-1.42263	down
1.006922	up	1.42235	up
2.752101	up	1.422098	up
-1.63117	down	-1.42196	down
-1.72447	down	1.421953	up
-2.08451	down	-1.42193	down
1.866509	up	1.421911	up
1.350002	up	-1.42166	down
-1.04802	down	-1.42154	down
1.671515	up	1.421308	up
1.440392	up	1.421089	up
1.52025	up	-1.42108	down
-1.80021	down	-1.42082	down
-1.78604	down	1.420787	up
-1.36678	down	-1.4205	down
-1.90737	down	-1.4205	down
1.451923	up	1.420279	up
6.00086	up	-1.4202	down
-1.37157	down	-1.42019	down
-1.30358	down	-1.42011	down
1.540609	up	1.42005	up
-2.0741	down	1.419794	up
-1.52837	down	1.419325	up
1.509314	up	1.419046	up
-1.12632	down	-1.41887	down
-1.76305	down	-1.41839	down
1.479743	up	1.418085	up
-2.58511	down	1.417937	up
-1.31215	down	1.417739	up
-2.06557	down	1.417452	up
-2.14384	down	-1.4173	down
-1.49656	down	-1.41723	down
1.643168	up	1.417064	up
3.647305	up	1.416405	up
-2.12471	down	1.415625	up
-1.23101	down	-1.41544	down
-1.31229	down	-1.41528	down
1.950132	up	1.415252	up
1.308354	up	-1.41514	down
1.361436	up	-1.41499	down
1.31116	up	1.414823	up
-2.13105	down	-1.41479	down
-2.49849	down	-1.41438	down
-2.15871	down	1.413599	up
-1.42475	down	-1.41353	down
1.701967	up	-1.41346	down
1.78919	up	-1.41268	down

-1.42724	down	-1.41245	down
-1.96176	down	1.412334	up
-1.01048	down	-1.41229	down
2.293634	up	1.412251	up
-1.50572	down	-1.4121	down
1.454125	up	1.412067	up
-1.78279	down	-1.41183	down
-1.43888	down	-1.41182	down
-1.70622	down	1.411793	up
-2.21171	down	-1.41118	down
1.479257	up	1.411113	up
-1.53373	down	-1.41065	down
-1.72639	down	-1.41063	down
-1.42117	down	-1.41058	down
-1.1354	down	-1.41053	down
-1.58222	down	-1.41047	down
-1.46428	down	-1.41044	down
-3.29365	down	-1.41039	down
-1.50015	down	-1.41007	down
-1.51203	down	-1.40982	down
-1.88558	down	-1.40959	down
-1.66648	down	-1.40929	down
-1.69504	down	-1.40929	down
-1.42724	down	-1.40916	down
4.108358	up	1.408771	up
-1.78832	down	-1.40876	down
-1.4359	down	-1.40851	down
-1.37981	down	-1.40847	down
-1.73039	down	-1.40843	down
1.321935	up	-1.40804	down
2.660933	up	1.407485	up
1.050484	up	-1.40746	down
-1.15207	down	-1.40736	down
-1.48547	down	-1.40714	down
1.455138	up	1.406723	up
1.288382	up	1.406707	up
-2.00024	down	1.405962	up
1.637861	up	1.405711	up
1.415887	up	1.405427	up
2.343825	up	1.405093	up
-1.51802	down	-1.40507	down
-1.84328	down	-1.40484	down
-1.7005	down	-1.4047	down
-2.0658	down	1.404651	up
-1.39352	down	-1.40462	down
-1.82519	down	-1.40452	down
1.518254	up	-1.40444	down

-1.31321	down	-1.40433	down
1.10188	up	1.404286	up
-1.65627	down	-1.40421	down
-1.66229	down	-1.40416	down
1.997578	up	1.403854	up
1.159031	up	-1.40357	down
-1.8975	down	-1.40321	down
1.932244	up	-1.40273	down
1.105593	up	1.402671	up
1.52513	up	-1.40239	down
1.130398	up	1.402229	up
1.468676	up	1.402111	up
1.916733	up	1.401498	up
5.859417	up	1.401409	up
1.72532	up	-1.40102	down
-1.45965	down	-1.40081	down
-1.75696	down	-1.40057	down
-1.58683	down	-1.4005	down
1.417887	up	1.400064	up
1.486002	up	1.399896	up
-2.00071	down	1.399384	up
-1.49707	down	-1.39903	down
-1.50618	down	-1.39881	down
-1.83981	down	-1.39822	down
1.210083	up	-1.39806	down
-1.99472	down	-1.39754	down
-1.21005	down	-1.3975	down
-1.51754	down	-1.39685	down
-1.51342	down	-1.3963	down
-1.28178	down	-1.3962	down
-1.86998	down	-1.39603	down
1.524376	up	-1.39602	down
-1.44135	down	-1.39597	down
1.576121	up	1.395967	up
7.908361	up	1.395483	up
1.771407	up	-1.39506	down
-2.36941	down	-1.39465	down
-1.85347	down	-1.39464	down
1.774107	up	1.394483	up
1.662739	up	-1.39439	down
1.579266	up	1.394358	up
-1.36553	down	-1.39432	down
-1.75571	down	-1.39389	down
1.346622	up	1.393876	up
1.097851	up	-1.3938	down
-1.31626	down	-1.39365	down
-1.41386	down	-1.39353	down

2.214501	up	-1.3931	down
-1.46442	down	-1.3928	down
-1.18865	down	-1.3927	down
3.006322	up	-1.39247	down
-1.63167	down	-1.39228	down
1.462916	up	-1.39182	down
-1.71115	down	-1.39177	down
1.095079	up	-1.39169	down
-1.12256	down	-1.39167	down
1.00736	up	-1.39165	down
1.004697	up	-1.39147	down
-1.83074	down	-1.39142	down
-1.5657	down	-1.39133	down
-1.50329	down	-1.39121	down
2.104305	up	1.391049	up
1.082506	up	1.390971	up
-1.93089	down	-1.39078	down
-1.76705	down	-1.39078	down
-1.18253	down	-1.39054	down
1.678591	up	1.390531	up
1.139619	up	1.390521	up
1.532898	up	1.390511	up
-1.44537	down	-1.39029	down
-1.46202	down	-1.39013	down
-1.52294	down	-1.39	down
1.226915	up	-1.38999	down
-1.53687	down	-1.38978	down
1.313025	up	-1.38922	down
2.430274	up	-1.38907	down
1.634881	up	-1.38897	down
-1.46609	down	-1.38863	down
-1.60766	down	-1.38832	down
-1.68131	down	-1.38803	down
-1.33889	down	-1.38796	down
5.009911	up	-1.38746	down
-2.98574	down	1.387342	up
1.239759	up	-1.38702	down
1.184441	up	1.386647	up
-1.17658	down	-1.38662	down
2.303386	up	1.386463	up
1.985269	up	1.386217	up
-1.40438	down	-1.38586	down
-1.58098	down	-1.38579	down
-1.45831	down	-1.38561	down
-1.39969	down	-1.38549	down
2.32862	up	1.385159	up
1.861349	up	1.384298	up

1.480653	up	1.384216	up
-1.46638	down	-1.38417	down
-1.40534	down	-1.38416	down
-3.87109	down	1.38411	up
2.319513	up	-1.38385	down
-1.38936	down	-1.38383	down
-1.84119	down	1.383802	up
-3.22842	down	-1.38374	down
1.065142	up	1.383497	up
-1.55917	down	-1.38337	down
1.788277	up	1.383339	up
-1.5332	down	-1.38258	down
1.046965	up	-1.38249	down
-3.14986	down	1.382083	up
-1.52903	down	-1.38196	down
-1.23591	down	-1.38187	down
1.390792	up	1.381155	up
1.773405	up	-1.38105	down
-3.82257	down	-1.38094	down
2.194604	up	-1.38067	down
2.193398	up	1.380528	up
-1.68528	down	-1.38045	down
-1.65341	down	-1.38042	down
-1.31948	down	-1.38035	down
1.999944	up	1.38033	up
-2.07243	down	-1.38012	down
-1.64964	down	-1.37998	down
1.211269	up	1.3799	up
1.652201	up	1.379822	up
-27.7141	down	1.37977	up
-1.59805	down	-1.37973	down
2.120109	up	-1.37937	down
1.451689	up	1.379247	up
1.609465	up	-1.37923	down
2.560876	up	1.379074	up
-2.12077	down	-1.37892	down
11.93535	up	1.378473	up
1.418249	up	-1.37833	down
1.702963	up	1.378313	up
1.198877	up	1.378034	up
1.36381	up	-1.37799	down
1.32931	up	-1.3775	down
2.373395	up	1.377448	up
2.283006	up	1.377397	up
-2.08716	down	1.37711	up
1.565467	up	1.377034	up
-2.03999	down	1.376964	up

-1.5017	down	-1.37605	down
1.371101	up	1.375975	up
2.169452	up	1.375821	up
-1.71359	down	1.375715	up
-2.81358	down	-1.37564	down
-1.57562	down	-1.3756	down
1.259324	up	-1.37547	down
-1.56366	down	-1.37539	down
1.043607	up	-1.37506	down
-1.61913	down	-1.37504	down
-1.54728	down	-1.37496	down
-1.5737	down	-1.37485	down
1.220436	up	1.3746	up
1.134583	up	1.374333	up
1.536286	up	1.374328	up
-1.32333	down	-1.37431	down
1.457155	up	1.373571	up
-1.62288	down	1.373435	up
1.564186	up	1.373295	up
1.844731	up	-1.373	down
1.64676	up	-1.3725	down
1.065139	up	-1.37226	down
1.633833	up	1.37222	up
1.496828	up	-1.37218	down
-1.64497	down	1.372074	up
-1.56515	down	-1.37206	down
1.895333	up	1.371934	up
2.272032	up	1.371555	up
-1.44934	down	-1.37152	down
1.525336	up	1.371365	up
-1.50475	down	-1.37086	down
-1.62065	down	-1.37076	down
-2.03791	down	1.370758	up
-1.92827	down	-1.37019	down
-1.49646	down	1.370135	up
2.147145	up	-1.37009	down
-1.03574	down	-1.36998	down
-1.55049	down	-1.36945	down
-1.49886	down	-1.36923	down
3.239599	up	1.369167	up
-1.91306	down	-1.36893	down
-1.74106	down	-1.36882	down
-1.73748	down	-1.36874	down
-2.3672	down	1.368724	up
-1.66713	down	-1.36859	down
2.695509	up	1.368563	up
-6.08947	down	1.368249	up

1.73593	up	1.368172	up
2.135258	up	1.368091	up
-1.31632	down	1.367854	up
-1.39795	down	-1.36779	down
-1.81002	down	-1.36775	down
1.489584	up	-1.36765	down
-1.42456	down	-1.3671	down
-2.47082	down	1.366722	up
1.579708	up	1.36669	up
-2.02576	down	1.366675	up
-1.03355	down	1.366455	up
1.78647	up	1.366232	up
-1.59081	down	-1.36541	down
-1.4691	down	-1.36514	down
-1.1554	down	1.364781	up
1.621847	up	1.36476	up
-1.24111	down	1.364572	up
1.197536	up	-1.36365	down
1.226223	up	1.363542	up
-1.47595	down	-1.36346	down
2.753311	up	1.363164	up
-1.58691	down	-1.36301	down
2.456394	up	-1.36284	down
-2.14426	down	-1.36266	down
-1.72478	down	-1.36216	down
1.961978	up	1.361722	up
-1.57957	down	-1.36089	down
1.028314	up	-1.36074	down
-1.28	down	-1.36035	down
1.009482	up	-1.36025	down
1.496386	up	1.36021	up
-1.10821	down	-1.3601	down
2.324994	up	-1.36006	down
-1.71653	down	-1.35996	down
2.164285	up	1.359918	up
-1.5647	down	-1.35961	down
-1.47373	down	-1.35957	down
-1.54847	down	-1.35952	down
1.21519	up	-1.35873	down
-1.60345	down	-1.35849	down
1.933914	up	1.358422	up
1.640696	up	-1.3584	down
1.707377	up	-1.35784	down
1.327266	up	1.357409	up
-1.48371	down	-1.35723	down
-1.5397	down	-1.35688	down
1.651168	up	1.356834	up

2.310059	up	1.356733	up
1.165592	up	-1.3563	down
1.786817	up	1.356113	up
-1.52429	down	-1.35598	down
-1.6027	down	-1.35595	down
-1.52691	down	-1.35574	down
1.666609	up	1.355688	up
-3.01416	down	1.35534	up
-1.56227	down	-1.35521	down
2.170281	up	-1.35499	down
-1.03341	down	-1.35476	down
-1.44069	down	-1.3544	down
-1.59104	down	1.354389	up
-1.29345	down	1.354126	up
1.580406	up	1.353948	up
-1.70942	down	-1.35386	down
-1.60106	down	-1.35383	down
2.424835	up	-1.35383	down
-1.43643	down	-1.35376	down
1.374478	up	1.353439	up
-1.59728	down	-1.35294	down
1.287063	up	1.352896	up
2.124754	up	1.352682	up
1.688118	up	1.352319	up
-1.70507	down	-1.3523	down
1.634604	up	1.352297	up
-2.3309	down	-1.35186	down
-1.50563	down	-1.35184	down
-1.16785	down	-1.35179	down
1.203161	up	-1.35171	down
-1.52481	down	-1.35133	down
-1.40679	down	-1.35128	down
-1.18179	down	-1.35117	down
-1.33561	down	-1.35085	down
1.191168	up	-1.3506	down
-2.11782	down	-1.35045	down
1.719962	up	1.350425	up
-1.485	down	-1.35036	down
1.772588	up	1.3503	up
-1.72063	down	1.350006	up
-1.77986	down	-1.34971	down
-2.56549	down	1.349688	up
-1.50689	down	1.349644	up
-1.64127	down	-1.34943	down
1.85093	up	1.348829	up
-1.4058	down	-1.34832	down
-2.31898	down	-1.34791	down

-1.62393	down	-1.34787	down
-1.50522	down	-1.34778	down
-2.04097	down	1.347715	up
-1.38551	down	-1.34767	down
-1.51309	down	-1.34737	down
1.53426	up	1.347249	up
-1.05401	down	1.347172	up
-2.05898	down	1.347011	up
2.118675	up	-1.34687	down
-1.10114	down	1.346817	up
1.60323	up	1.346627	up
-1.70929	down	-1.3466	down
-1.21027	down	-1.34647	down
-16.6489	down	1.346228	up
7.405079	up	-1.3462	down
-1.25585	down	-1.34601	down
-1.4256	down	-1.34598	down
-1.65323	down	-1.34589	down
2.231596	up	1.345835	up
-3.21466	down	1.345702	up
2.053838	up	1.345547	up
1.709795	up	1.345331	up
1.270418	up	-1.3452	down
-1.22945	down	1.34519	up
-1.09471	down	-1.34411	down
-1.92962	down	-1.34406	down
-1.52347	down	-1.34401	down
-1.55096	down	-1.34384	down
-1.32655	down	1.343679	up
-1.67804	down	1.343408	up
-1.25648	down	-1.34326	down
-1.92094	down	-1.343	down
-2.01998	down	-1.34268	down
4.042211	up	1.342566	up
-1.08453	down	-1.34247	down
1.204946	up	-1.34206	down
1.423536	up	-1.34198	down
-1.53522	down	-1.34176	down
-1.09303	down	-1.34171	down
-1.54549	down	-1.34164	down
-1.50301	down	1.341615	up
-2.03533	down	-1.34131	down
-2.11954	down	-1.34058	down
1.03388	up	1.34054	up
1.797801	up	1.340464	up
-3.8214	down	1.340288	up
-1.06394	down	1.339753	up

-1.6913	down	-1.33974	down
-1.89516	down	-1.33968	down
1.830353	up	1.339454	up
1.475402	up	1.339259	up
2.149926	up	-1.33917	down
3.007434	up	1.338942	up
1.26562	up	-1.33885	down
2.290252	up	1.338544	up
-10.6883	down	1.338423	up
-1.64922	down	-1.33815	down
-1.31187	down	-1.33785	down
-2.1118	down	-1.33758	down
-1.60346	down	-1.33748	down
-1.00423	down	-1.33742	down
1.191451	up	-1.33708	down
1.519065	up	-1.33696	down
-1.27235	down	-1.33667	down
-2.05147	down	1.336638	up
-1.66187	down	-1.33658	down
-2.57985	down	-1.3364	down
1.864015	up	1.336149	up
1.343192	up	-1.33601	down
-2.4346	down	1.33583	up
-5.62835	down	1.335787	up
1.518974	up	-1.33578	down
-1.70294	down	-1.33549	down
-1.53562	down	-1.33547	down
-1.27942	down	-1.33544	down
1.215603	up	1.335171	up
1.297097	up	-1.33428	down
1.649125	up	1.334052	up
-1.56404	down	-1.33403	down
-2.38008	down	-1.33371	down
1.611514	up	1.33337	up
-1.52125	down	-1.33318	down
-2.09298	down	1.333094	up
1.140267	up	-1.33307	down
1.554835	up	-1.33259	down
2.972008	up	1.332433	up
-1.7385	down	-1.33221	down
-1.88649	down	-1.3318	down
-1.48079	down	-1.33158	down
1.73537	up	-1.33155	down
1.073582	up	1.331427	up
-1.58067	down	-1.33062	down
1.905891	up	-1.33045	down
1.075354	up	1.330366	up

-1.51817	down	-1.33019	down
1.826599	up	1.329778	up
1.404379	up	1.329777	up
-1.69524	down	-1.32955	down
-2.55265	down	1.329518	up
1.229205	up	-1.32934	down
-1.14641	down	1.328764	up
-1.18943	down	1.328638	up
-1.89076	down	-1.32795	down
-2.4422	down	1.327934	up
1.17668	up	1.327756	up
-1.75861	down	-1.3268	down
-1.35152	down	-1.32669	down
3.281103	up	1.326598	up
-1.43713	down	-1.32657	down
1.620521	up	1.326408	up
2.000286	up	1.326309	up
3.039481	up	-1.32628	down
-2.13752	down	-1.3261	down
-1.55621	down	-1.32595	down
3.018807	up	-1.32588	down
1.81276	up	1.325459	up
-1.45073	down	-1.32541	down
1.557833	up	-1.32539	down
1.347098	up	-1.32534	down
2.713668	up	1.325316	up
2.003853	up	-1.32529	down
1.482839	up	-1.32507	down
1.183177	up	1.324896	up
-1.57437	down	-1.32486	down
-1.59695	down	-1.32484	down
-1.15073	down	-1.32466	down
2.181379	up	-1.32444	down
1.124176	up	-1.3244	down
-2.24758	down	1.324351	up
1.045825	up	-1.32424	down
-1.7184	down	-1.32364	down
-2.03944	down	1.323457	up
1.559428	up	1.323444	up
2.321603	up	1.32292	up
-1.55221	down	-1.32291	down
-1.60092	down	-1.32277	down
1.026084	up	1.322661	up
4.703414	up	-1.32248	down
-3.40133	down	-1.32244	down
-2.05877	down	-1.32218	down
-1.51682	down	-1.32212	down

1.847174	up	1.321739	up
-2.65463	down	1.321088	up
-1.28272	down	-1.3209	down
1.297771	up	-1.32089	down
-1.87294	down	1.320825	up
-1.02433	down	-1.3208	down
4.721606	up	1.320681	up
2.23746	up	-1.32044	down
1.183912	up	-1.32032	down
-1.55584	down	-1.32025	down
-1.99438	down	-1.32012	down
1.712738	up	-1.32011	down
-1.56454	down	-1.31997	down
-2.04839	down	-1.31974	down
-1.53752	down	-1.31963	down
-1.92121	down	-1.31937	down
1.610885	up	-1.31935	down
-1.94728	down	-1.31891	down
-1.57171	down	-1.31863	down
2.069616	up	-1.31829	down
1.066684	up	-1.31823	down
1.467639	up	-1.31802	down
1.929191	up	1.317963	up
-1.62953	down	-1.31794	down
1.00382	up	-1.31778	down
-1.36551	down	-1.31776	down
-1.52155	down	-1.31774	down
2.220756	up	1.317637	up
1.03549	up	-1.31759	down
-1.5523	down	-1.31705	down
2.291381	up	-1.31702	down
-1.16412	down	1.316922	up
2.261501	up	1.316786	up
-2.74684	down	1.316541	up
-1.74606	down	-1.31585	down
-1.87028	down	-1.31581	down
-2.41383	down	-1.3155	down
-1.98429	down	-1.31538	down
1.97055	up	1.314423	up
-2.4452	down	1.314398	up
-1.5232	down	1.314188	up
-1.60523	down	-1.31418	down
-2.02857	down	1.314163	up
-1.43253	down	-1.3138	down
1.324273	up	1.313724	up
-1.41906	down	-1.31364	down
-1.63169	down	1.31344	up

1.733936	up	1.312491	up
-1.18088	down	1.312448	up
1.543169	up	1.312446	up
-1.59139	down	-1.3124	down
-2.33555	down	-1.31238	down
-1.73324	down	-1.31234	down
-1.66265	down	1.312204	up
1.064842	up	1.31193	up
1.255787	up	-1.31155	down
-1.70717	down	-1.31152	down
-1.072	down	-1.31125	down
-1.60204	down	-1.31112	down
-1.99115	down	1.311102	up
-2.63159	down	1.311098	up
1.655628	up	1.31083	up
2.066721	up	-1.31067	down
-1.27763	down	-1.31057	down
-2.19655	down	-1.31051	down
-1.48097	down	-1.31039	down
1.018243	up	-1.31023	down
-1.50098	down	-1.31019	down
-1.23217	down	-1.31018	down
-1.57291	down	-1.3101	down
-2.43127	down	-1.30987	down
-1.4948	down	-1.30983	down
2.882957	up	1.309757	up
-1.17591	down	1.309591	up
-1.22193	down	-1.30952	down
-2.02881	down	1.30947	up
1.203003	up	-1.30923	down
1.686543	up	-1.30905	down
-1.54452	down	-1.30899	down
2.275603	up	-1.30897	down
-1.16606	down	-1.30891	down
-1.41188	down	-1.30883	down
-2.3968	down	1.308094	up
-1.41277	down	-1.30802	down
2.25459	up	1.307041	up
-1.57567	down	-1.30699	down
2.670276	up	1.306887	up
-2.06507	down	-1.30683	down
-1.15596	down	-1.30671	down
1.533779	up	1.306593	up
-2.02994	down	-1.30631	down
1.453478	up	-1.30628	down
-5.22512	down	1.306249	up
-2.33422	down	-1.30624	down

2.075182	up	1.306134	up
-1.99253	down	-1.30601	down
-2.17066	down	-1.30563	down
2.071058	up	1.305483	up
-1.6514	down	-1.30538	down
-1.9952	down	-1.30527	down
-1.64021	down	-1.30518	down
-1.12616	down	-1.30515	down
-1.63066	down	-1.30515	down
-2.62326	down	1.304894	up
-1.86614	down	-1.3047	down
1.167598	up	-1.30468	down
1.152286	up	-1.30458	down
-3.15719	down	-1.30426	down
2.04866	up	1.304239	up
-1.76132	down	-1.30422	down
1.839398	up	1.304111	up
-1.54685	down	-1.30408	down
-1.79647	down	-1.30405	down
-1.54147	down	-1.30395	down
-1.79216	down	-1.30389	down
-3.1264	down	-1.30382	down
-1.222	down	-1.30366	down
1.254974	up	1.303617	up
-2.31634	down	-1.30339	down
-1.34955	down	-1.30339	down
3.136287	up	1.303295	up
1.677412	up	-1.30322	down
-1.21925	down	1.303152	up
-1.46905	down	-1.30313	down
2.160042	up	-1.30313	down
-2.11697	down	1.303114	up
-1.79756	down	-1.30304	down
-16.1555	down	-1.30268	down
2.194848	up	1.302594	up
2.241355	up	1.30256	up
-1.56778	down	-1.30243	down
-1.53662	down	-1.30235	down
-2.03945	down	-1.30227	down
-1.7417	down	-1.30213	down
-1.31515	down	-1.30211	down
-2.02601	down	-1.3021	down
-1.25387	down	-1.3015	down
-1.25364	down	-1.30142	down
2.330519	up	1.301045	up
1.408004	up	1.301029	up
1.970105	up	-1.30003	down

1.579794	up	1.299754	up
-1.59824	down	-1.29971	down
-1.95487	down	-1.29944	down
1.249503	up	1.29922	up
-1.73847	down	-1.2992	down
-2.62282	down	1.299185	up
-1.66272	down	-1.29919	down
-1.22155	down	-1.29905	down
-2.49648	down	-1.29884	down
-1.21821	down	-1.2986	down
2.214075	up	1.298266	up
3.989771	up	1.298114	up
-2.49892	down	-1.29805	down
2.164587	up	-1.2979	down
-1.92821	down	-1.29772	down
-1.53537	down	-1.29769	down
1.642534	up	1.297322	up
-2.0054	down	1.297218	up
-16.9678	down	1.296933	up
-1.40194	down	-1.29692	down
1.583909	up	1.296841	up
1.069842	up	1.296579	up
-1.70863	down	-1.29642	down
1.361301	up	-1.2964	down
6.194767	up	-1.29594	down
1.405468	up	1.295297	up
-1.6422	down	-1.29485	down
-2.75516	down	1.294398	up
2.532579	up	1.294258	up
7.081519	up	1.294121	up
-1.8397	down	-1.29389	down
-1.45444	down	-1.29345	down
-1.44525	down	-1.29333	down
1.862928	up	1.293234	up
1.773095	up	1.29289	up
-2.02432	down	-1.29286	down
2.027621	up	-1.29274	down
1.832488	up	1.292313	up
1.551727	up	1.292254	up
-2.14265	down	-1.2919	down
-1.4226	down	-1.29172	down
-2.86879	down	-1.29166	down
1.56456	up	1.291553	up
-1.53025	down	-1.29131	down
-1.50464	down	-1.2912	down
-2.99889	down	1.290995	up
-1.46341	down	-1.29082	down

-1.66761	down	-1.2907	down
1.774171	up	1.290643	up
-1.46381	down	-1.2903	down
2.25235	up	1.290102	up
-1.61845	down	-1.2898	down
1.953773	up	1.289717	up
-2.31662	down	-1.28961	down
-1.55594	down	-1.28961	down
-1.83169	down	-1.28948	down
1.558303	up	1.289473	up
1.772586	up	1.289383	up
1.236841	up	1.289078	up
-1.05636	down	-1.28874	down
1.142011	up	-1.2886	down
-1.31819	down	-1.2875	down
-1.64892	down	-1.28742	down
-1.65389	down	-1.28734	down
-2.32916	down	1.287266	up
2.689756	up	-1.28711	down
-1.55566	down	-1.28691	down
1.807839	up	1.286889	up
2.64961	up	1.286039	up
-1.64054	down	-1.28508	down
-1.73547	down	-1.28493	down
-2.59622	down	1.284624	up
-1.78607	down	-1.28458	down
-1.46127	down	-1.28456	down
-1.57781	down	-1.28451	down
1.270969	up	1.284382	up
-1.74515	down	-1.28437	down
-1.84097	down	1.28428	up
-1.97226	down	-1.28426	down
-2.49754	down	-1.28416	down
1.17721	up	-1.28411	down
-1.51908	down	-1.28399	down
2.048451	up	1.283876	up
1.428178	up	1.283794	up
-1.46052	down	-1.28273	down
1.506393	up	-1.28261	down
1.70869	up	1.282556	up
-1.58739	down	-1.28209	down
-2.40397	down	1.281882	up
1.537149	up	1.281717	up
-1.01152	down	1.281477	up
1.369095	up	1.281446	up
1.957519	up	1.281441	up
-2.07902	down	-1.28142	down

4.779107	up	1.28134	up
2.417132	up	1.281269	up
-1.71387	down	-1.28124	down
-1.49111	down	-1.28062	down
1.032546	up	1.280611	up
-1.71893	down	-1.28055	down
-2.00704	down	-1.28027	down
2.943833	up	-1.28014	down
-3.2554	down	-1.27981	down
-2.02694	down	-1.27978	down
-1.64303	down	-1.27972	down
4.448703	up	1.279407	up
2.049764	up	1.279323	up
-2.5285	down	-1.27909	down
-1.65666	down	-1.27873	down
1.78849	up	-1.27868	down
2.782683	up	1.278592	up
-1.32421	down	-1.27821	down
-1.2619	down	-1.27793	down
1.421533	up	-1.27768	down
-1.19305	down	-1.27756	down
-2.49384	down	1.277289	up
2.318208	up	-1.27667	down
2.300914	up	1.275314	up
-1.70964	down	-1.27449	down
2.184164	up	1.274034	up
-1.66317	down	-1.27399	down
1.658545	up	1.273879	up
1.589189	up	1.273563	up
-2.25599	down	1.273405	up
-1.92263	down	-1.27324	down
1.621708	up	1.273179	up
-1.66026	down	-1.27301	down
2.477811	up	1.272953	up
-1.55862	down	-1.27283	down
-1.26364	down	-1.27254	down
2.004184	up	-1.27251	down
-1.35961	down	-1.27244	down
-1.80556	down	-1.27239	down
-2.87266	down	1.272238	up
-1.7712	down	-1.27197	down
1.016465	up	-1.27155	down
-1.99	down	-1.27152	down
2.660243	up	1.271378	up
2.040972	up	1.271282	up
-1.90717	down	-1.27117	down
-1.30881	down	-1.27103	down

-1.04199	down	-1.27101	down
-2.02157	down	1.270912	up
1.648189	up	-1.27082	down
-1.05926	down	-1.27082	down
-1.7291	down	-1.2708	down
-1.57976	down	-1.27074	down
-1.21009	down	-1.27063	down
1.596695	up	1.270572	up
2.455637	up	1.270158	up
1.786524	up	1.269923	up
-1.76389	down	-1.26984	down
1.555773	up	1.269794	up
-2.08911	down	1.269702	up
-1.59599	down	-1.26939	down
-1.59105	down	-1.26938	down
1.148775	up	1.269205	up
-2.32034	down	-1.26913	down
2.13417	up	1.269089	up
-1.55429	down	-1.26901	down
-2.20346	down	1.268768	up
-1.41038	down	-1.26863	down
-1.67558	down	1.268575	up
-1.00437	down	1.26846	up
2.075553	up	1.268169	up
-2.96035	down	1.267954	up
-2.52803	down	-1.2677	down
-2.01851	down	-1.26766	down
-2.84658	down	1.267557	up
2.044699	up	1.267409	up
-1.72936	down	-1.26729	down
1.899915	up	-1.26728	down
2.821062	up	-1.26717	down
-1.62862	down	-1.26688	down
-2.08692	down	-1.26668	down
1.620862	up	1.266551	up
-1.87985	down	-1.26622	down
1.710689	up	1.266068	up
2.166104	up	1.265985	up
1.875894	up	1.265867	up
-1.34088	down	-1.2658	down
-1.76498	down	-1.26538	down
9.250532	up	-1.26524	down
-1.73766	down	-1.26498	down
1.892426	up	1.264685	up
-1.68913	down	-1.2644	down
1.556504	up	1.264348	up
-1.63628	down	1.264168	up

1.962936	up	1.263918	up
-2.62251	down	-1.2636	down
1.439625	up	-1.26341	down
-1.94543	down	-1.26318	down
-1.9437	down	-1.26312	down
-4.80179	down	-1.26292	down
-1.61469	down	-1.26217	down
-1.74311	down	-1.26207	down
-1.73341	down	-1.26188	down
3.077369	up	1.261684	up
2.159945	up	-1.2615	down
-1.65994	down	-1.26134	down
-1.31955	down	-1.26087	down
1.530413	up	-1.26059	down
-2.69986	down	-1.26047	down
-3.02599	down	1.260432	up
-3.51206	down	1.260353	up
-1.85977	down	-1.26004	down
-1.26989	down	-1.25967	down
-1.96311	down	-1.2592	down
-1.61114	down	-1.25872	down
2.113622	up	-1.2586	down
-1.61616	down	-1.25828	down
1.229273	up	1.258224	up
-1.65436	down	-1.25801	down
3.416503	up	1.257785	up
-1.01189	down	-1.25776	down
1.906983	up	1.257455	up
1.878573	up	-1.25732	down
-2.3038	down	-1.25676	down
-1.23231	down	1.256548	up
-1.94227	down	-1.25643	down
-1.68865	down	-1.2561	down
-1.5167	down	-1.2561	down
-1.35323	down	-1.25591	down
2.131699	up	-1.25567	down
-1.99217	down	-1.25562	down
-1.90714	down	-1.25559	down
-1.9971	down	-1.25524	down
-1.47367	down	-1.25494	down
-3.33488	down	-1.25468	down
1.203013	up	-1.25466	down
2.005232	up	-1.25458	down
-1.88404	down	-1.25399	down
1.422303	up	-1.25386	down
-1.3007	down	-1.25381	down
3.903153	up	-1.2537	down

1.63674	up	1.253664	up
-1.62471	down	-1.25314	down
1.641	up	1.252781	up
-2.02238	down	1.252565	up
-3.37923	down	1.252391	up
-2.1476	down	1.251842	up
-2.42233	down	1.251748	up
-1.99445	down	-1.25172	down
-1.44774	down	1.251718	up
-1.74155	down	-1.25143	down
-1.92248	down	1.251216	up
-1.44281	down	1.251019	up
1.620227	up	1.251002	up
3.664689	up	-1.25082	down
2.15677	up	-1.25035	down
-1.2891	down	-1.25001	down
-1.63651	down	-1.25	down
-1.22077	down	-1.24981	down
-1.25546	down	-1.24976	down
-1.5121	down	-1.24961	down
5.640478	up	1.249439	up
1.732216	up	-1.24938	down
1.616794	up	1.249271	up
1.695239	up	1.24877	up
-1.70199	down	-1.24864	down
-2.04801	down	-1.24846	down
2.029006	up	-1.24835	down
1.935818	up	1.24768	up
-1.58215	down	-1.24753	down
1.798199	up	1.247335	up
1.430514	up	1.247085	up
-1.47495	down	-1.24667	down
-1.38683	down	1.246588	up
-1.10848	down	-1.24595	down
-2.02282	down	1.245784	up
-1.86934	down	-1.24533	down
2.277559	up	-1.24532	down
4.421477	up	1.244957	up
-1.18773	down	-1.24479	down
1.707627	up	1.244343	up
-1.54489	down	-1.24432	down
-1.67375	down	-1.2441	down
-1.4689	down	1.243739	up
-2.21622	down	1.243686	up
3.516869	up	-1.24368	down
1.808164	up	1.243419	up
-1.72027	down	-1.24327	down

1.971131	up	1.243159	up
-1.70102	down	-1.24307	down
1.469211	up	-1.24303	down
32.31743	up	1.242778	up
1.287366	up	-1.24263	down
1.804899	up	1.242393	up
2.083462	up	1.242351	up
1.149747	up	-1.24183	down
1.391226	up	1.241284	up
7.092676	up	1.241191	up
-2.07924	down	-1.24118	down
-1.4544	down	-1.24116	down
-1.02203	down	-1.24097	down
-1.23578	down	-1.24082	down
-1.82414	down	1.240784	up
2.745008	up	1.240756	up
1.92606	up	1.240512	up
-2.23886	down	-1.24048	down
-1.74642	down	-1.24003	down
2.848913	up	-1.23968	down
-1.91846	down	-1.23943	down
-1.84813	down	-1.23922	down
-3.70816	down	-1.23907	down
1.1767	up	1.238936	up
2.65787	up	-1.23887	down
-1.95042	down	-1.23878	down
-1.56967	down	-1.23877	down
-1.81762	down	-1.23864	down
2.771143	up	1.23854	up
-1.50384	down	-1.2379	down
-1.83629	down	-1.23787	down
-1.73427	down	-1.23765	down
2.158181	up	-1.23727	down
1.829153	up	1.237153	up
-1.19722	down	-1.23695	down
-1.74825	down	-1.23684	down
-1.73821	down	-1.23681	down
-1.82604	down	-1.23675	down
1.293685	up	1.23672	up
1.962112	up	-1.23624	down
-2.0649	down	-1.23601	down
-1.68388	down	-1.236	down
2.701183	up	-1.23533	down
2.372215	up	1.235279	up
1.516036	up	-1.23504	down
-1.69222	down	-1.23443	down
-2.04068	down	-1.23422	down

3.679984	up	1.234054	up
1.662323	up	-1.23374	down
2.192606	up	1.23369	up
-1.69259	down	-1.23361	down
3.7314	up	1.233454	up
1.244445	up	1.233423	up
1.930961	up	1.233307	up
-1.815	down	-1.23328	down
-1.43132	down	-1.23305	down
-1.503	down	-1.23304	down
-1.38587	down	-1.23289	down
1.772804	up	1.232657	up
-1.5942	down	-1.23238	down
-1.64685	down	-1.23213	down
-2.37085	down	-1.23151	down
-2.79568	down	1.231029	up
-1.36443	down	1.2305	up
1.363968	up	-1.23047	down
1.919098	up	1.230133	up
-1.70208	down	-1.22994	down
-2.54428	down	-1.2297	down
-1.96784	down	-1.22943	down
-2.68359	down	1.229301	up
-1.63016	down	-1.22823	down
-2.18289	down	1.227743	up
2.024013	up	1.227132	up
-1.68174	down	-1.22709	down
1.603045	up	-1.22697	down
1.160522	up	1.226883	up
2.133663	up	-1.22682	down
-1.98998	down	-1.22624	down
2.248607	up	-1.22621	down
-1.59385	down	-1.2255	down
-1.22165	down	1.225041	up
-1.73263	down	-1.22482	down
-1.94282	down	-1.22471	down
-1.48438	down	1.224414	up
2.747101	up	-1.22438	down
1.97092	up	-1.22409	down
1.919179	up	1.223805	up
-1.60538	down	-1.22356	down
-1.99179	down	-1.22316	down
-2.28016	down	1.223092	up
2.360163	up	1.222872	up
2.287467	up	1.222417	up
1.120982	up	1.222378	up
-1.57625	down	1.221805	up

-2.35609	down	-1.22177	down
-1.79201	down	-1.22129	down
-1.87368	down	-1.22118	down
-1.84713	down	1.221131	up
-2.02974	down	1.220831	up
-2.26009	down	-1.22062	down
4.025886	up	1.220445	up
4.287704	up	-1.22034	down
1.00797	up	1.220331	up
1.75775	up	1.220158	up
-2.38672	down	-1.22	down
1.00483	up	-1.21973	down
-3.11814	down	-1.2196	down
1.752231	up	1.219454	up
-1.8198	down	-1.21871	down
2.622126	up	-1.21816	down
-1.67455	down	-1.21785	down
-1.71068	down	-1.21779	down
-2.15465	down	-1.21765	down
6.736889	up	1.217367	up
-2.00192	down	1.217106	up
-1.10014	down	1.217026	up
1.727817	up	1.216942	up
-1.18249	down	-1.21693	down
-1.26628	down	-1.21689	down
1.618924	up	1.216885	up
-1.15285	down	-1.21686	down
1.422382	up	-1.21654	down
-1.81624	down	-1.21632	down
1.900254	up	1.215647	up
2.144307	up	1.215623	up
2.388872	up	-1.21562	down
-1.31214	down	-1.2156	down
-1.49666	down	-1.21555	down
-1.2363	down	-1.21555	down
2.839934	up	1.215469	up
3.131156	up	-1.21518	down
2.46582	up	1.215155	up
1.900559	up	1.214943	up
-1.88139	down	-1.21465	down
-1.79301	down	-1.21463	down
1.307748	up	-1.2144	down
3.680189	up	-1.21391	down
2.125558	up	1.213769	up
-1.70634	down	-1.21373	down
-2.05351	down	-1.21369	down
2.000346	up	1.213173	up

-1.28436	down	-1.21276	down
-2.10578	down	1.212649	up
1.50674	up	-1.21257	down
-1.09011	down	-1.21252	down
-2.78613	down	-1.21214	down
2.323132	up	-1.21129	down
-2.36215	down	1.211059	up
1.33712	up	-1.21094	down
-1.6287	down	-1.21078	down
-1.45513	down	-1.21068	down
-1.69366	down	-1.21039	down
-2.17468	down	-1.21038	down
-2.21156	down	1.21026	up
1.256409	up	-1.21005	down
-1.23372	down	-1.20998	down
2.211074	up	-1.20978	down
-1.43518	down	-1.20948	down
-1.48583	down	-1.20904	down
2.273619	up	1.208962	up
1.651545	up	1.208476	up
2.326351	up	-1.20819	down
-1.64144	down	-1.20807	down
-1.37044	down	-1.20789	down
1.442295	up	-1.20784	down
1.234037	up	-1.20779	down
-1.05522	down	1.207553	up
1.193349	up	1.207228	up
2.278899	up	1.206389	up
-2.43766	down	1.206245	up
1.507432	up	1.205341	up
2.030769	up	-1.20459	down
1.962442	up	1.204539	up
1.661936	up	-1.20384	down
1.810208	up	1.203748	up
-1.84202	down	-1.20353	down
2.258786	up	-1.20309	down
-1.84371	down	-1.20306	down
1.230994	up	-1.20257	down
-1.84073	down	1.202542	up
-3.28981	down	-1.2022	down
-2.06275	down	1.202168	up
-1.77499	down	-1.20207	down
3.672399	up	-1.2018	down
1.681112	up	1.201638	up
-1.86765	down	-1.20136	down
2.206262	up	-1.20132	down
1.664693	up	1.20112	up

1.709501	up	1.201086	up
-2.95024	down	1.20067	up
7.931088	up	1.200465	up
2.531965	up	1.200452	up
-1.39525	down	-1.20036	down
6.658943	up	-1.20002	down
-3.16995	down	-1.19982	down
1.037316	up	-1.19981	down
-2.04994	down	1.199692	up
-1.45976	down	-1.19909	down
-1.59266	down	-1.19876	down
2.260255	up	-1.19871	down
-1.4217	down	-1.19854	down
-2.209	down	-1.19795	down
-1.43629	down	-1.19757	down
4.333635	up	1.197246	up
1.819497	up	1.1969	up
1.671546	up	1.196776	up
1.031348	up	1.196279	up
-1.6901	down	1.19585	up
-2.46105	down	1.195785	up
-2.63657	down	1.195624	up
71.74509	up	-1.1956	down
1.362425	up	-1.19554	down
2.223084	up	-1.19551	down
2.269694	up	1.195435	up
-1.35459	down	1.195247	up
2.50538	up	-1.19502	down
1.713707	up	1.19487	up
-1.75954	down	-1.1947	down
-1.32128	down	-1.19462	down
-1.30571	down	-1.19447	down
1.293441	up	-1.19432	down
2.106263	up	1.193996	up
-2.34671	down	1.193614	up
-1.80057	down	-1.19295	down
1.481025	up	1.192895	up
1.707264	up	1.192772	up
1.82392	up	1.192637	up
-1.57998	down	-1.19225	down
1.554379	up	-1.19143	down
1.920922	up	1.190974	up
-1.98438	down	-1.19087	down
-1.39341	down	-1.1908	down
2.002439	up	-1.19018	down
-1.91799	down	-1.19001	down
-1.81635	down	-1.18995	down

1.85913	up	1.189878	up
1.381855	up	-1.18987	down
1.723642	up	1.189799	up
-11.4203	down	-1.18977	down
1.734199	up	1.189724	up
-1.49928	down	-1.18954	down
-1.82233	down	-1.18935	down
-1.32114	down	-1.18899	down
1.67076	up	1.188542	up
-2.45133	down	-1.18808	down
-11.0178	down	-1.18805	down
-1.92015	down	-1.18784	down
1.779043	up	1.18757	up
1.398197	up	-1.18754	down
2.642122	up	-1.18719	down
-1.66788	down	-1.1871	down
1.637174	up	1.186943	up
-1.59625	down	-1.18636	down
1.902761	up	1.186248	up
1.706363	up	-1.1862	down
-2.36234	down	1.185661	up
-2.16839	down	-1.18541	down
2.046809	up	1.185251	up
1.052039	up	1.185239	up
-1.64743	down	-1.18516	down
1.299413	up	-1.18443	down
1.606133	up	1.184415	up
12.71059	up	1.184411	up
1.346304	up	1.184266	up
1.941812	up	1.183658	up
1.827765	up	1.183636	up
1.475216	up	1.183082	up
1.857945	up	1.182883	up
1.794786	up	1.182871	up
-1.5463	down	-1.18265	down
1.848114	up	-1.18252	down
-1.78401	down	-1.1821	down
3.707141	up	1.181997	up
-1.41396	down	-1.18152	down
1.431908	up	1.181177	up
1.022978	up	-1.18114	down
2.116279	up	-1.18097	down
-6.06592	down	1.180615	up
1.857313	up	-1.18034	down
-1.59734	down	-1.18014	down
1.909917	up	1.179911	up
-1.43366	down	-1.1799	down

-1.68989	down	-1.17948	down
-1.65161	down	-1.17925	down
-1.61326	down	1.179137	up
1.808276	up	-1.1788	down
-1.3258	down	1.178793	up
-1.28458	down	-1.17868	down
-1.85934	down	-1.17829	down
-2.53603	down	-1.17825	down
-1.14719	down	-1.17823	down
1.33804	up	-1.178	down
2.110868	up	1.177687	up
-2.07828	down	-1.17742	down
-2.27556	down	1.177188	up
1.207357	up	1.177142	up
-1.79212	down	-1.17714	down
1.512154	up	-1.17665	down
1.013047	up	-1.17647	down
1.150503	up	-1.17625	down
2.155341	up	1.176243	up
2.217137	up	-1.17603	down
1.738009	up	1.17575	up
3.96289	up	1.175515	up
-1.2743	down	-1.17532	down
-1.01574	down	1.175079	up
1.884081	up	1.174782	up
2.125973	up	-1.17469	down
2.0959	up	1.174361	up
40.42667	up	1.174277	up
-1.60083	down	-1.17418	down
-1.42288	down	-1.17415	down
-1.40113	down	-1.17397	down
-1.58864	down	-1.1738	down
2.501413	up	1.173766	up
1.811827	up	1.173724	up
-9.46026	down	1.1737	up
-2.01392	down	-1.17336	down
1.907291	up	1.173224	up
-1.78898	down	-1.17259	down
-2.30216	down	-1.17244	down
1.831041	up	1.172251	up
-1.79002	down	-1.17224	down
-1.56485	down	-1.17183	down
-1.10125	down	-1.17111	down
2.323985	up	-1.1709	down
-1.69841	down	-1.17076	down
-1.78277	down	-1.17048	down
-2.01102	down	1.170235	up

2.494378	up	-1.17003	down
-1.57591	down	-1.16999	down
-1.81605	down	-1.16998	down
8.412665	up	1.169537	up
-1.8773	down	-1.16943	down
-1.68708	down	1.169368	up
-1.74162	down	-1.16936	down
-1.28283	down	-1.16917	down
1.559762	up	1.169071	up
1.425517	up	1.168894	up
1.348651	up	1.168584	up
-1.48446	down	-1.16856	down
1.160682	up	-1.16828	down
1.693548	up	1.167853	up
-1.50783	down	-1.16632	down
-1.69876	down	1.165765	up
-2.44904	down	-1.16568	down
1.939344	up	-1.16562	down
1.184664	up	1.165475	up
1.46843	up	1.165258	up
-1.98013	down	-1.16498	down
1.915411	up	1.164796	up
2.292897	up	1.16423	up
1.067897	up	-1.16394	down
-1.53681	down	-1.16387	down
-1.1734	down	-1.16376	down
1.626628	up	1.163188	up
2.336265	up	-1.16293	down
-1.81265	down	-1.16273	down
-1.24047	down	-1.16266	down
-1.60052	down	-1.16266	down
-1.06947	down	-1.16265	down
-1.0234	down	-1.16259	down
-1.62983	down	-1.16217	down
-1.76916	down	-1.16211	down
-2.31884	down	1.162025	up
2.360309	up	1.161922	up
-1.81624	down	-1.16188	down
1.542116	up	-1.16179	down
2.521662	up	1.161452	up
-1.96424	down	-1.16137	down
1.606631	up	-1.1613	down
-1.44843	down	-1.16121	down
1.868396	up	1.160772	up
2.703676	up	1.160308	up
1.705471	up	1.159157	up
3.790566	up	-1.1591	down

1.951683	up	-1.15888	down
2.034218	up	1.158383	up
-1.94624	down	-1.15824	down
-3.39276	down	-1.15812	down
2.945997	up	1.158007	up
2.641192	up	1.15791	up
-1.07602	down	1.1579	up
2.01825	up	1.157303	up
2.150148	up	1.157151	up
1.60472	up	1.157115	up
-2.32585	down	1.157047	up
-1.84859	down	-1.15687	down
-1.76427	down	-1.15647	down
-1.7924	down	-1.15611	down
2.420084	up	1.155921	up
1.156415	up	1.155886	up
1.917279	up	1.155779	up
2.250476	up	1.155117	up
2.532168	up	1.154758	up
-2.1351	down	1.154689	up
-1.53503	down	-1.15468	down
1.777287	up	1.154427	up
-2.2366	down	-1.1535	down
-2.48868	down	1.153485	up
-1.78963	down	-1.15348	down
4.872914	up	-1.15346	down
1.350264	up	1.153365	up
-1.48047	down	-1.15336	down
1.887228	up	1.152022	up
1.893539	up	1.151866	up
-1.8363	down	-1.15176	down
-2.10834	down	1.151641	up
-1.98719	down	-1.1513	down
-1.58123	down	-1.15124	down
2.152179	up	1.15115	up
1.162628	up	1.151129	up
-2.00204	down	1.151033	up
1.770394	up	1.150296	up
-2.08369	down	1.149651	up
1.819456	up	-1.14959	down
1.780501	up	1.149198	up
2.126757	up	-1.14911	down
-1.33045	down	-1.14896	down
1.935887	up	1.148914	up
1.8386	up	1.148828	up
-1.90902	down	-1.14874	down
2.001472	up	1.14825	up

-2.13709	down	-1.14801	down
1.622286	up	1.147996	up
1.595648	up	1.147848	up
-2.48637	down	1.147499	up
1.607728	up	1.147371	up
-6.45221	down	1.147343	up
1.798957	up	1.147328	up
-1.52665	down	1.147258	up
1.748706	up	1.147171	up
1.487985	up	1.14716	up
-1.39079	down	1.147023	up
-2.17778	down	-1.14671	down
-2.46538	down	-1.14587	down
2.277025	up	1.145641	up
-2.29944	down	1.145569	up
-1.73082	down	-1.14535	down
-1.80487	down	-1.14521	down
-1.19713	down	-1.14501	down
-2.18879	down	1.144987	up
1.796529	up	1.144892	up
-1.88497	down	-1.14486	down
3.603581	up	1.144663	up
-4.45488	down	1.144445	up
-1.69467	down	1.144187	up
2.684075	up	-1.14415	down
-1.13176	down	-1.14405	down
-1.3145	down	-1.14352	down
-1.51668	down	-1.14344	down
-1.98653	down	1.142857	up
-1.79667	down	-1.14233	down
5.459221	up	1.142094	up
5.731956	up	-1.1419	down
1.927379	up	1.141857	up
-1.74956	down	-1.14172	down
-1.94457	down	1.141199	up
2.642584	up	1.14107	up
-1.96986	down	-1.14105	down
4.753702	up	1.140805	up
1.169764	up	-1.14079	down
1.807355	up	-1.14073	down
-1.88016	down	-1.14067	down
-1.97362	down	-1.14034	down
-3.2289	down	1.14017	up
1.689195	up	-1.13954	down
2.240433	up	1.139353	up
7.074349	up	-1.13911	down
-1.74611	down	-1.13878	down

-1.75362	down	1.138662	up
3.886125	up	1.138318	up
2.082814	up	-1.13812	down
5.148092	up	1.13802	up
1.472784	up	1.137874	up
-1.60883	down	-1.13784	down
1.922283	up	1.137776	up
2.17204	up	1.137133	up
1.241426	up	1.137082	up
-1.79985	down	-1.13653	down
-1.34753	down	-1.13603	down
2.266866	up	1.135874	up
-1.58272	down	-1.13577	down
2.131255	up	-1.13495	down
1.884774	up	1.134896	up
-2.06411	down	-1.13479	down
-1.49681	down	-1.13474	down
1.580111	up	-1.13428	down
5.299768	up	-1.13399	down
2.033473	up	1.133857	up
-1.51655	down	-1.13379	down
-3.31897	down	-1.13376	down
-1.78009	down	-1.13329	down
2.593878	up	1.133075	up
1.90253	up	1.131945	up
-2.6134	down	-1.13178	down
3.033089	up	-1.13174	down
-11.388	down	1.131501	up
4.239292	up	1.130894	up
1.342808	up	-1.13064	down
-1.98922	down	-1.13063	down
2.886401	up	-1.13052	down
-1.81037	down	1.130158	up
1.709968	up	1.129443	up
-1.99439	down	1.129156	up
1.428732	up	1.128878	up
-3.03304	down	-1.12881	down
3.442101	up	-1.12873	down
-1.33919	down	-1.12859	down
-1.61064	down	-1.12858	down
-6.8807	down	1.128576	up
-3.74585	down	-1.1284	down
1.532734	up	-1.12806	down
2.112602	up	-1.12797	down
-2.01071	down	1.127765	up
-1.57154	down	-1.12747	down
-2.25003	down	1.127407	up

2.002562	up	1.127376	up
2.980757	up	1.127235	up
2.182736	up	-1.12723	down
-1.33798	down	1.127198	up
-3.31632	down	1.12719	up
-1.9824	down	-1.12693	down
-2.13249	down	-1.12677	down
-1.11229	down	-1.1266	down
-1.08461	down	1.126532	up
-1.87696	down	-1.1265	down
-1.41272	down	1.126221	up
2.971002	up	1.126123	up
-2.08254	down	-1.12578	down
1.91255	up	-1.12569	down
-2.00515	down	1.125144	up
2.67712	up	1.125023	up
2.058745	up	1.124957	up
1.52262	up	-1.12494	down
2.028913	up	1.124567	up
-1.43976	down	-1.12449	down
3.323926	up	-1.1243	down
-1.71485	down	-1.12427	down
-1.67668	down	-1.12392	down
2.604913	up	-1.12357	down
4.401721	up	-1.12352	down
-2.7252	down	-1.12351	down
2.794356	up	1.123457	up
-2.02119	down	-1.12336	down
-1.72202	down	-1.12303	down
-2.7254	down	1.123018	up
-1.54937	down	-1.12271	down
-1.54049	down	-1.12221	down
-1.5169	down	1.12207	up
-2.49896	down	-1.12203	down
1.461544	up	1.121866	up
-1.84437	down	-1.12182	down
1.719214	up	1.121675	up
1.223619	up	1.121473	up
-2.56628	down	-1.12084	down
2.252045	up	-1.12083	down
2.230957	up	-1.12078	down
1.532795	up	1.120742	up
-2.25269	down	-1.12026	down
-1.81962	down	-1.1202	down
2.189918	up	-1.12017	down
-2.25627	down	-1.11997	down
2.017142	up	1.119906	up

4.13228	up	1.11977	up
-1.06273	down	1.119644	up
-1.44356	down	1.11962	up
-3.3209	down	-1.11949	down
-2.00721	down	1.119068	up
-2.3954	down	1.119067	up
-2.14998	down	1.119026	up
2.13437	up	-1.11896	down
-1.17747	down	-1.11895	down
9.011562	up	1.118947	up
1.096024	up	-1.11892	down
2.140039	up	1.11866	up
-1.09021	down	-1.11836	down
1.993362	up	-1.1183	down
5.952239	up	1.118097	up
2.16638	up	1.117719	up
-2.36065	down	-1.11739	down
-1.37146	down	-1.11719	down
1.148807	up	1.117164	up
-1.87174	down	-1.11691	down
1.868775	up	-1.11642	down
-1.20023	down	-1.11635	down
2.756388	up	1.116129	up
-1.80547	down	-1.11597	down
1.299706	up	1.115964	up
-1.97399	down	-1.11575	down
-2.83921	down	-1.11523	down
-1.35292	down	-1.11519	down
3.005447	up	1.114393	up
-1.79287	down	-1.11396	down
1.454383	up	1.113742	up
-4.88537	down	1.113101	up
2.947096	up	1.113092	up
-1.69567	down	-1.11215	down
1.265161	up	1.111821	up
1.316471	up	-1.11157	down
-2.60861	down	-1.11137	down
-1.7007	down	1.111175	up
1.65104	up	-1.11095	down
1.994627	up	1.11081	up
-2.32731	down	1.110795	up
-3.06029	down	1.110522	up
1.401366	up	1.110374	up
-2.37884	down	1.10998	up
-4.15791	down	1.109692	up
-2.56042	down	1.109638	up
-2.01551	down	-1.10958	down

-2.09016	down	-1.10946	down
2.041519	up	-1.10901	down
2.422627	up	-1.10899	down
-1.36162	down	1.108946	up
1.945132	up	1.108881	up
2.40469	up	1.108452	up
-1.31386	down	-1.10823	down
1.996877	up	1.10822	up
-2.1379	down	1.108168	up
-2.87379	down	1.108161	up
-1.54734	down	-1.10797	down
1.822019	up	1.107825	up
-2.02573	down	-1.10762	down
2.357422	up	1.107415	up
3.368942	up	-1.10733	down
-1.23389	down	1.107329	up
-2.99366	down	1.105982	up
1.019824	up	1.105918	up
2.165339	up	-1.10567	down
-1.85776	down	-1.10542	down
-1.6119	down	-1.10523	down
1.569772	up	1.105037	up
-2.0607	down	1.10496	up
3.49388	up	1.104896	up
1.374807	up	-1.10468	down
-2.48011	down	-1.10465	down
1.181928	up	-1.10457	down
1.894549	up	1.104551	up
-2.54486	down	1.104222	up
-2.32036	down	-1.10384	down
1.506542	up	1.103334	up
-1.72271	down	-1.10328	down
1.561279	up	-1.10325	down
-1.23313	down	-1.10281	down
2.035669	up	1.102561	up
2.287182	up	1.10227	up
-1.56751	down	-1.10213	down
2.029774	up	1.101764	up
-1.7467	down	1.101729	up
-1.05715	down	-1.10102	down
-1.51529	down	-1.10058	down
-1.08007	down	-1.10042	down
1.705852	up	-1.10031	down
1.297331	up	1.100083	up
1.409947	up	1.099979	up
-2.04738	down	1.099901	up
2.587809	up	-1.0999	down

-1.92273	down	-1.09935	down
-2.03981	down	1.099318	up
-1.45906	down	1.099128	up
2.71468	up	1.098894	up
-1.61749	down	-1.09869	down
5.15306	up	1.098599	up
2.415642	up	-1.09851	down
2.519636	up	1.097357	up
2.410638	up	-1.09709	down
1.568553	up	1.097051	up
1.836275	up	1.096836	up
4.400109	up	1.096612	up
1.590004	up	1.096585	up
-1.579	down	-1.09639	down
2.094332	up	1.096242	up
-1.60784	down	-1.09621	down
-2.47169	down	1.09618	up
3.349085	up	1.095994	up
1.2166	up	-1.09588	down
1.834869	up	-1.09583	down
2.320601	up	1.095772	up
2.476877	up	1.095481	up
1.671593	up	1.095472	up
-2.28393	down	-1.09514	down
-1.82291	down	-1.0951	down
1.321721	up	-1.09495	down
3.799463	up	1.093903	up
-1.05774	down	1.093895	up
-1.98482	down	-1.09384	down
1.538627	up	1.093639	up
-4.51996	down	-1.09334	down
-1.91972	down	-1.09317	down
1.519951	up	-1.09313	down
-1.53911	down	-1.09308	down
1.220068	up	1.092984	up
1.389673	up	-1.09234	down
-2.72366	down	1.092334	up
2.081799	up	1.092254	up
-1.19939	down	-1.09207	down
-1.49679	down	1.091932	up
2.236177	up	1.091637	up
2.06411	up	1.091498	up
1.290863	up	1.091462	up
-2.12128	down	-1.09144	down
10.98799	up	1.091396	up
2.005709	up	-1.09137	down
2.002465	up	-1.09108	down

-2.90509	down	-1.0908	down
-2.50662	down	1.090591	up
1.275188	up	1.090452	up
3.729648	up	1.089815	up
2.268368	up	1.089514	up
-1.36002	down	1.089418	up
2.093403	up	1.089295	up
1.782075	up	-1.08851	down
1.687411	up	1.087473	up
-1.43322	down	1.087353	up
-1.68233	down	-1.08729	down
1.452845	up	-1.08682	down
-2.12305	down	-1.08668	down
1.997809	up	-1.08653	down
1.97558	up	-1.08542	down
1.360977	up	1.085363	up
-1.62921	down	-1.08449	down
-2.18633	down	1.084459	up
-1.87079	down	-1.08442	down
27.62352	up	-1.08428	down
3.38607	up	-1.08419	down
-2.39718	down	1.083954	up
2.370996	up	1.083502	up
-1.88232	down	-1.0833	down
2.246589	up	-1.08324	down
-1.88483	down	-1.08299	down
14.75743	up	-1.08263	down
-2.61044	down	-1.08255	down
1.806345	up	-1.08251	down
2.528549	up	-1.08225	down
2.133198	up	-1.08186	down
-1.20148	down	1.081521	up
-9.79673	down	-1.0812	down
-2.40639	down	1.081015	up
-3.41075	down	-1.08097	down
-1.3688	down	1.080924	up
3.344097	up	1.080842	up
-1.39886	down	1.08039	up
2.152345	up	-1.08005	down
-2.38094	down	1.079997	up
2.492708	up	-1.07987	down
2.078223	up	-1.0796	down
-2.1998	down	-1.07947	down
2.052877	up	-1.0794	down
-1.2138	down	-1.07903	down
-2.08768	down	-1.07894	down
-1.83992	down	-1.07888	down

-2.10365	down	-1.07883	down
-2.08021	down	-1.0786	down
4.091306	up	1.07854	up
-2.12351	down	1.078276	up
-2.5127	down	1.077901	up
2.549192	up	-1.07764	down
-1.27198	down	1.077482	up
1.919521	up	1.077103	up
1.982145	up	-1.07686	down
1.614239	up	-1.07631	down
1.530009	up	-1.07563	down
2.806228	up	1.075482	up
1.583944	up	-1.07534	down
-1.34731	down	-1.07509	down
-1.08593	down	1.075075	up
-2.10045	down	-1.07477	down
-1.09076	down	-1.07444	down
-3.04793	down	-1.07422	down
-1.80076	down	-1.07411	down
-1.84268	down	-1.07376	down
-2.53104	down	1.073222	up
-2.11751	down	-1.07291	down
-1.66967	down	1.072761	up
2.414117	up	1.072652	up
-1.87599	down	1.072413	up
-1.65717	down	1.072181	up
1.572705	up	-1.07205	down
-1.7163	down	-1.07194	down
2.51469	up	-1.07184	down
-1.75794	down	-1.07171	down
3.999392	up	1.071395	up
-1.1037	down	-1.07092	down
-1.93903	down	-1.07088	down
-1.6102	down	1.070804	up
1.939482	up	1.070763	up
1.883783	up	1.070701	up
2.056446	up	1.070404	up
8.726066	up	1.069536	up
1.763183	up	1.068716	up
1.984533	up	1.068497	up
-1.92554	down	1.068123	up
1.473616	up	1.067955	up
-1.96278	down	1.067826	up
-1.25471	down	-1.06747	down
2.344415	up	1.067221	up
-1.9982	down	-1.06715	down
2.249413	up	-1.06661	down

2.033947	up	1.066395	up
-1.3821	down	-1.06599	down
-2.07325	down	-1.06569	down
2.246051	up	1.065637	up
3.13658	up	1.064904	up
2.620403	up	-1.06466	down
-1.95401	down	1.064376	up
-2.61552	down	1.064253	up
-1.54696	down	1.063992	up
2.270708	up	-1.06387	down
-1.352	down	1.063701	up
-2.13076	down	1.0637	up
1.923934	up	-1.06343	down
-1.63847	down	-1.06295	down
-2.17214	down	-1.06239	down
-1.2729	down	-1.06237	down
1.145734	up	-1.06173	down
-2.64103	down	1.061719	up
2.213466	up	1.061485	up
4.151064	up	1.061377	up
1.413538	up	1.061139	up
-2.08357	down	-1.06095	down
1.843721	up	-1.06025	down
2.142111	up	1.059793	up
1.085811	up	-1.0596	down
1.558499	up	-1.05944	down
2.714059	up	1.059436	up
-1.51613	down	-1.05938	down
-1.52658	down	-1.05936	down
2.664744	up	1.058884	up
-1.33306	down	1.058777	up
-5.06218	down	1.058618	up
-1.92889	down	-1.05858	down
2.038299	up	-1.05847	down
-1.50803	down	-1.05816	down
-2.04611	down	1.057948	up
-1.70124	down	1.057895	up
1.343312	up	-1.05748	down
2.255229	up	-1.05736	down
-2.28574	down	-1.05688	down
-1.83203	down	1.056548	up
-1.61441	down	1.056244	up
-2.54794	down	-1.0553	down
-1.35026	down	-1.05436	down
-1.68452	down	1.054286	up
4.416947	up	-1.05334	down
-1.68008	down	-1.05301	down

1.156113	up	-1.05282	down
2.250392	up	-1.05274	down
-1.7997	down	-1.05263	down
1.934572	up	-1.05255	down
-1.43327	down	-1.05245	down
-1.77461	down	-1.05225	down
-3.27848	down	-1.05135	down
-1.74511	down	-1.05129	down
1.256452	up	1.051271	up
2.276456	up	1.050902	up
2.810659	up	1.050186	up
2.670665	up	1.050057	up
1.22346	up	1.05004	up
1.328644	up	1.049419	up
17.93265	up	-1.04933	down
-2.89303	down	-1.04931	down
-1.18504	down	1.048961	up
1.12243	up	-1.04842	down
-1.24919	down	1.048315	up
-1.92023	down	-1.0483	down
-2.4115	down	-1.04827	down
-1.74057	down	-1.04781	down
6.193117	up	-1.04777	down
-1.21217	down	1.047632	up
-2.06566	down	-1.04729	down
-2.11511	down	-1.04716	down
1.574502	up	-1.04664	down
2.228547	up	1.046603	up
-2.85966	down	-1.04649	down
1.630252	up	-1.0463	down
-1.65236	down	1.045733	up
2.197128	up	1.045624	up
1.097126	up	-1.04481	down
1.731539	up	1.044773	up
2.204547	up	-1.04475	down
-1.74411	down	-1.04388	down
-1.45508	down	1.043579	up
2.027486	up	1.043369	up
2.168662	up	-1.04334	down
-2.03618	down	-1.04324	down
-1.18985	down	-1.04315	down
1.406389	up	-1.04314	down
2.147219	up	-1.04301	down
2.537156	up	1.042897	up
1.222755	up	1.042334	up
2.472207	up	1.04208	up
2.715362	up	-1.04207	down

1.849123	up	1.041881	up
-2.46886	down	-1.04177	down
1.358577	up	-1.04176	down
1.657994	up	1.041381	up
-1.79473	down	1.041214	up
-3.35165	down	-1.04117	down
-1.20057	down	-1.04093	down
4.23818	up	1.03975	up
1.971849	up	-1.03906	down
-2.03573	down	1.038879	up
6.783411	up	1.038173	up
3.37112	up	1.037836	up
1.995577	up	1.037448	up
-1.37758	down	-1.0374	down
-2.43433	down	-1.03729	down
-1.8794	down	-1.03726	down
2.079727	up	-1.03723	down
1.631641	up	1.037169	up
-1.15409	down	-1.037	down
-2.56158	down	-1.0367	down
1.774953	up	1.036657	up
3.121358	up	1.036321	up
-2.15832	down	-1.03573	down
-2.08037	down	-1.03565	down
-2.00973	down	1.035477	up
-1.4006	down	-1.03465	down
2.80114	up	-1.03457	down
-1.93416	down	-1.03453	down
-1.74686	down	1.033999	up
1.249989	up	-1.03321	down
-1.82284	down	-1.03206	down
-7.33385	down	-1.03201	down
-1.6272	down	-1.0317	down
-1.40748	down	1.031413	up
-3.04263	down	-1.03133	down
-1.31016	down	-1.03115	down
3.640898	up	-1.03109	down
1.85719	up	1.030796	up
-1.98283	down	-1.02994	down
-1.0787	down	-1.02977	down
-1.72975	down	-1.02964	down
-2.42244	down	1.029573	up
-1.41618	down	1.029195	up
-1.99083	down	-1.02865	down
-1.42424	down	-1.02865	down
2.066116	up	-1.02844	down
-3.62171	down	-1.0283	down

-1.3744	down	-1.02809	down
-1.58753	down	1.027898	up
2.742884	up	1.027876	up
-2.23754	down	-1.02764	down
-5.81743	down	1.027221	up
2.333243	up	1.027053	up
-2.27776	down	-1.02702	down
2.907029	up	1.02687	up
1.579757	up	1.02661	up
-2.4519	down	1.026354	up
1.043549	up	1.02518	up
-2.05486	down	-1.02514	down
-1.2576	down	-1.02502	down
2.833462	up	-1.02491	down
2.415935	up	1.02489	up
1.963055	up	1.024774	up
-2.32641	down	-1.02435	down
1.865256	up	1.024296	up
-1.51189	down	-1.02419	down
-1.81873	down	1.023978	up
3.569365	up	1.023449	up
2.513687	up	1.023237	up
-6.22245	down	-1.02308	down
2.232845	up	-1.02265	down
1.878443	up	-1.02205	down
-2.13524	down	-1.02183	down
-1.6304	down	-1.02168	down
2.402915	up	-1.02166	down
7.763259	up	1.021207	up
-2.18307	down	-1.02091	down
-1.5364	down	-1.02056	down
-1.81932	down	-1.02046	down
-1.7825	down	-1.01989	down
-1.88895	down	-1.01937	down
1.378979	up	1.018833	up
3.562658	up	1.018691	up
-2.82674	down	1.018571	up
2.38688	up	-1.01846	down
1.921088	up	1.018444	up
1.3161	up	1.018355	up
2.508637	up	-1.018	down
-2.29788	down	1.017658	up
2.066742	up	-1.01724	down
-1.4558	down	1.017208	up
3.715954	up	1.017165	up
7.915718	up	-1.01715	down
3.895129	up	-1.01701	down

-1.55055	down	-1.01678	down
-2.97219	down	-1.01638	down
1.84195	up	1.015588	up
2.041325	up	1.015199	up
2.618071	up	1.015085	up
6.14488	up	1.01494	up
2.527272	up	-1.01454	down
-1.13836	down	1.014498	up
-2.60766	down	1.0143	up
2.466811	up	-1.01399	down
2.154425	up	-1.01383	down
1.243574	up	-1.0131	down
2.032426	up	1.012906	up
1.237465	up	1.011963	up
-1.51618	down	-1.01161	down
-1.89979	down	-1.01154	down
1.912232	up	1.011515	up
8.619837	up	1.011397	up
-1.61096	down	1.01117	up
-1.70197	down	-1.01082	down
2.390417	up	-1.00965	down
2.006647	up	1.009626	up
1.650881	up	1.008985	up
2.910839	up	-1.00884	down
1.720914	up	1.008266	up
-1.81561	down	-1.00778	down
2.158094	up	-1.00774	down
1.650108	up	-1.00767	down
2.176061	up	-1.00763	down
2.236907	up	1.007426	up
2.681653	up	1.00711	up
2.351826	up	-1.00683	down
1.910067	up	-1.00642	down
-2.1757	down	1.00627	up
-2.51544	down	1.006101	up
2.575607	up	1.006096	up
-2.8026	down	-1.00584	down
-1.69974	down	-1.00524	down
2.484668	up	-1.00506	down
2.762901	up	1.004734	up
-1.94439	down	-1.00473	down
2.905284	up	-1.00423	down
-1.8388	down	1.003895	up
2.159445	up	1.003618	up
-1.63018	down	1.00325	up
-1.22028	down	1.003061	up
-2.14757	down	1.002987	up

2.695432	up	1.002942	up
1.487026	up	-1.00282	down
-1.7143	down	1.002736	up
-2.19078	down	-1.00259	down
2.203824	up	1.002587	up
-3.50386	down	-1.00226	down
1.583131	up	-1.00208	down
1.713336	up	1.001845	up
-2.62794	down	-1.00183	down
-1.67254	down	-1.00092	down
-3.70732	down	-1.00021	down
-4.01879	down	-1.00016	down
2.898255	up	1.000148	up
2.672644	up	-1.00005	down
-1.92206	down	1.000017	up
