# Supplementary material for: Physiological, anatomical and transcriptional alterations in a rice mutant leading to enhanced water stress tolerance
Source: AoB Plants. 2015 Mar 27;7:plv023. doi: 10.1093/aobpla/plv023 (PMC4482838; doi:10.1093/aobpla/plv023)
Supplement: Additional Information [file supp_plv023_plv023supp_table3.pdf]

Probe Sets	Matched MSU/TIGR	[MS] vs [M	Regulation	[MC] vs [N	Regulation	[NS] vs [M	Regulation	[MS] vs [N	Regulation
Os.12373.1.S1_at	LOC_Os08g35740.1	2.386172	up	1.712998	up	1.314359	down	4.087508	up
OsAffx.17696.1.S1_at	LOC_Os09g14700.1	2.069751	up	1.847202	up	1.006867	down	3.823249	up
Os.32943.1.S1_at	LOC_Os01g12660.1	1.074514	up	7.051409	down	17.98376	up	6.562418	down
Os.38157.1.S1_s_at	LOC_Os09g39910.1	2.676303	up	2.221092	down	3.741116	up	1.204949	up
Os.27963.1.A1_at	LOC_Os01g72900.1	1.42228	up	5.195618	down	1.554301	up	3.65302	down
Os.47343.2.S1_x_at	LOC_Os09g39740.1	2.422737	down	2.036273	up	2.367353	down	1.18979	down
Os.53355.1.S1_s_at	LOC_Os02g34990.1	3.163599	down	2.80033	up	2.669623	down	1.129724	down
Os.39973.1.S1_s_at	LOC_Os03g60580.1	3.62927	up	2.968476	down	4.752718	up	1.222604	up
Os.17130.1.S1_at	LOC_Os04g57540.1	2.508591	up	1.618408	up	1.205843	up	4.059925	up
Os.17213.2.S1_at	LOC_Os02g17970.1	2.39226	down	1.43836	down	1.097614	down	3.440931	down
Os.4801.1.S1_x_at	LOC_Os01g52010	1.403655	down	2.948458	up	1.420103	down	2.100558	up
Os.10908.1.S1_a_at	LOC_Os08g36910.1	2.43819	up	1.614806	up	1.055697	down	3.937203	up
Os.20892.1.S1_at	LOC_Os12g26290	1.068365	up	2.129121	up	1.310126	up	2.274678	up
Os.26807.1.S1_at	LOC_Os01g12210.1	4.376969	down	3.297222	up	2.999852	down	1.327472	down
Os.11407.1.S1_at	LOC_Os04g57550.1	2.164298	up	1.379097	up	1.002381	down	2.984776	up
Os.50896.1.S1_at	LOC_Os01g66010.1	3.601153	up	1.783995	up	1.007901	down	6.424441	up
Os.19070.1.S1_at	LOC_Os11g09020.1	2.060261	down	1.396126	down	1.245611	up	2.876385	down
Os.6080.1.S1_at	LOC_Os06g04380.1	1.692499	up	1.35399	down	3.682997	up	1.250009	up
Os.22522.1.A1_a_at	LOC_Os04g47190.1	2.128831	down	1.115457	down	1.033337	up	2.374618	down
Os.4377.1.S1_at	LOC_Os02g46970.1	1.202468	up	4.355243	up	2.165712	down	5.237039	up
OsAffx.16365.1.S1_at	LOC_Os07g22840.1	2.105418	up	2.342281	down	2.919858	up	1.112502	down
Os.51731.1.S1_at	LOC_Os12g40780.1	1.825808	up	1.737378	down	5.614204	up	1.050899	up
OsAffx.30872.1.S1_s_at	LOC_Os11g08020.1	2.279438	down	3.06261	up	4.20495	down	1.343581	up
OsAffx.2784.1.S1_s_at	LOC_Os02g29140.1	2.550659	down	2.23995	up	3.054506	down	1.138712	down
Os.38017.1.S1_s_at	LOC_Os05g31750.1	2.444308	up	2.418165	up	2.53369	down	5.910738	up
Os.11915.1.S1_at	LOC_Os02g51750.1	2.958665	up	2.704101	down	3.637528	up	1.09414	up
Os.17247.1.S1_at	LOC_Os02g55970.1	3.464984	up	1.004905	down	1.60387	up	3.448072	up
Os.17746.1.S1_s_at	LOC_Os01g64540.1	1.236234	up	4.611093	up	2.029767	down	5.700388	up
Os.10597.1.S1_at	LOC_Os01g53350.1	2.486742	down	2.107695	up	3.177145	down	1.17984	down
Os.12253.1.S1_at	LOC_Os01g45110.1	2.980098	down	2.755614	up	3.663153	down	1.081464	down
Os.26406.2.S1_x_at	LOC_Os03g03450.1	4.075366	up	1.115603	down	1.141762	up	3.653061	up
OsAffx.27278.1.S1_x_at	LOC_Os05g41760.1	2.878165	up	1.624415	up	1.057782	down	4.675335	up
OsAffx.27278.1.S1_at	LOC_Os05g41760.1	2.627775	up	1.890271	up	1.043477	down	4.967207	up

Os.55837.1.S1_x_at	LOC_Os01g10370.1	1.97256	up	1.283232	down	4.746084	up	1.537181	up
Os.15849.1.S1_s_at	LOC_Os08g36920.1	1.200327	up	6.280318	up	1.147617	down	7.538433	up
Os.52451.1.A1_at	LOC_Os09g28440.1	1.433245	down	3.64674	up	1.183012	down	2.544393	up
OsAffx.17366.1.S1_at	LOC_Os08g36920.1	1.114799	up	2.911963	up	1.052366	down	3.246254	up
Os.14921.1.S1_at	LOC_Os03g56930.1	1.059219	down	12.95672	up	3.027265	down	12.23234	up
Os.7012.1.S1_at	LOC_Os01g72000.1	1.307377	up	2.532696	up	1.089439	down	3.311189	up
Os.18851.2.A1_at	LOC_Os10g05730.2	1.248522	up	2.609593	up	1.022409	down	3.258134	up
Os.18473.1.S1_at	LOC_Os06g35050.1	2.551698	down	1.54995	down	1.289264	up	3.955004	down
Os.50300.1.S1_s_at	LOC_Os06g20040.1	1.007188	up	2.186653	up	1.315536	up	2.20237	up
Os.13623.1.S1_at	LOC_Os01g41550.1	1.499346	up	2.587437	up	1.124783	down	3.879461	up
Os.37797.1.A1_at	LOC_Os02g31960.1	2.590064	down	1.043739	down	1.264439	down	2.703351	down
Os.18003.1.S1_at	LOC_Os02g49460.1	2.032148	up	1.276802	up	1.004342	down	2.59465	up
Os.53237.1.S1_at	LOC_Os07g34070.1	2.637788	up	2.523016	up	2.256527	down	6.655181	up
Os.11580.1.S1_at	LOC_Os06g01972.1	2.070585	up	3.0112	up	2.63656	down	6.234946	up
OsAffx.4511.1.S1_s_at	LOC_Os05g33900.1	2.817768	down	3.543133	up	4.810993	down	1.257425	up
Os.7779.1.S1_x_at	LOC_Os05g01570.1	1.307927	up	1.045517	down	2.373024	down	1.250986	up
Os.27177.1.S1_at	LOC_Os03g16390.1	1.767579	up	3.223794	up	1.191325	up	5.698309	up
Os.38278.1.S1_at	LOC_Os03g06850.1	2.222014	down	1.551217	down	1.475453	up	3.446825	down
Os.27299.1.A1_at	LOC_Os01g04800.1	3.140524	down	3.399087	up	5.591302	down	1.082331	up
Os.24664.1.A1_at	LOC_Os03g18210.1	2.131109	down	1.184885	down	1.142285	up	2.52512	down
Os.1443.1.S1_a_at	LOC_Os01g06640.1	1.080645	down	3.09236	down	1.415015	up	3.341744	down
Os.7751.1.S1_at	LOC_Os02g47660.1	1.432474	down	3.514646	down	1.308476	down	5.034639	down
Os.14560.1.S1_at	LOC_Os02g07930	1.480704	up	1.088667	up	3.036702	up	1.611993	up
Os.49427.1.S1_at	LOC_Os02g03690	2.682904	down	1.14824	up	1.255891	up	2.336537	down
Os.28098.2.A1_at	LOC_Os10g32810.1	2.389247	up	2.061357	down	2.739056	up	1.159065	up
OsAffx.28771.1.S1_at	LOC_Os07g35940.1	1.911132	up	9.506679	up	2.09666	down	18.16852	up
Os.15854.1.S1_at	LOC_Os07g35940.1	1.335163	up	20.40843	up	2.161238	down	27.24858	up
Os.50964.1.S1_at	LOC_Os01g17250	2.548997	down	1.231052	down	1.193657	up	3.137947	down
Os.57345.1.S1_at	LOC_Os02g38120.1	1.065921	down	1.377556	up	2.131437	up	1.292363	up
Os.47694.1.S1_at	LOC_Os03g10880.1	2.947628	down	1.260045	up	1.294094	down	2.339304	down
Os.28427.1.S2_a_at	LOC_Os05g12640.1	1.137114	down	2.492795	up	1.023505	up	2.192212	up
Os.5277.1.S1_at	LOC_Os01g64730.1	3.612033	up	2.420326	down	4.321971	up	1.492375	up
Os.411.2.S1_x_at	LOC_Os01g64730.1	4.580067	up	2.760706	down	4.928227	up	1.659021	up
Os.56880.1.S1_at	LOC_Os01g36220.1	1.140836	down	1.169765	up	2.70537	up	1.025358	up

Os.8561.1.S1_at	LOC_Os02g10860	1.967124	up	1.358646	down	5.157498	up	1.447856	up
OsAffx.4464.1.S1_s_at	LOC_Os05g28950.1	3.629646	down	1.119389	up	1.453905	down	3.242526	down
Os.52189.1.S1_at	LOC_Os06g19260.1	2.044957	down	2.714404	up	3.153561	down	1.327365	up
Os.40342.1.A1_s_at	LOC_Os06g18900.1	1.067043	up	2.787984	up	1.286418	down	2.974899	up
Os.27624.1.S1_at	LOC_Os03g14590.1	2.976753	up	1.19498	down	1.177852	up	2.491047	up
Os.2694.1.S1_at	LOC_Os04g43200.1	6.49781	up	4.599403	down	8.885311	up	1.412751	up
Os.48013.1.A1_at	LOC_Os03g12230.1	1.568237	up	1.329274	down	4.616178	up	1.17977	up
Os.28514.1.S1_at	LOC_Os01g04280.1	2.592416	down	2.72222	up	2.844208	down	1.050071	up
Os.49503.1.S1_at	LOC_Os03g27080.2	3.722625	down	3.205506	up	3.771193	down	1.161322	down
Os.52632.1.S1_at	LOC_Os04g58990.1	2.125038	down	1.185223	down	1.041337	down	2.518644	down
Os.19815.1.S1_at	LOC_Os03g43440.1	3.288305	down	2.25835	up	2.50278	down	1.456065	down
Os.12261.1.S1_a_at	LOC_Os03g20380.1	1.302216	down	2.681387	down	1.088843	up	3.491745	down
Os.52657.1.S1_at	LOC_Os04g42580.1	2.573016	down	1.189371	up	1.245614	down	2.163342	down
OsAffx.23104.1.S1_at	LOC_Os01g03914.2	1.379334	down	2.787689	down	1.008231	down	3.845156	down
OsAffx.29695.1.S1_s_at	LOC_Os09g02710.1	2.170318	up	1.794259	up	1.198849	down	3.894111	up
Os.25613.1.A1_at	LOC_Os02g05470.1	1.005524	down	1.509197	down	4.153893	up	1.517534	down
Os.57511.1.A1_at	LOC_Os02g05470.1	1.057839	down	1.259851	down	2.597192	up	1.332719	down
Os.16422.1.S1_s_at	LOC_Os08g15050.1	1.932104	up	2.635738	up	1.022868	down	5.092519	up
Os.27959.1.A1_at	LOC_Os06g07480.1	2.106506	down	1.553876	down	1.026605	up	3.273249	down
Os.18724.1.S1_at	LOC_Os01g54620.1	1.615625	up	7.136817	up	2.719499	down	11.53042	up
Os.4857.1.S1_at	LOC_Os03g62090.1	1.446888	up	2.226382	up	1.040671	up	3.221324	up
Os.10926.1.S1_at	LOC_Os07g14850.1	2.050252	up	1.531196	up	1.362933	down	3.139337	up
Os.12416.1.S1_at	LOC_Os11g32650.1	1.364115	up	15.48479	up	3.389404	down	21.12305	up
Os.6354.1.S1_at	LOC_Os12g02370.2	1.130001	up	3.16812	up	1.31613	up	3.579978	up
Os.37295.1.S1_at	LOC_Os11g02440.1	1.530405	up	6.759543	up	1.662791	up	10.34484	up
Os.3415.1.S1_s_at	LOC_Os10g39680.1	2.539447	up	2.425664	down	3.284655	up	1.046908	up
Os.37184.1.S1_at	LOC_Os01g43220.1	2.85092	down	1.665476	down	1.015827	down	4.748139	down
Os.11766.1.S1_at	LOC_Os07g36170.1	1.252287	up	2.407521	up	1.032193	down	3.014907	up
Os.28216.1.S1_a_at	LOC_Os01g52240.1	1.609054	up	6.865312	up	1.132417	down	11.04666	up
Os.28216.3.S1_x_at	LOC_Os01g52240.1	1.525648	up	5.732348	up	1.169073	down	8.745545	up
OsAffx.32313.1.A1_at	LOC_Os08g15306.1	1.087884	down	2.070499	down	1.693785	down	2.252464	down
OsAffx.32196.1.A1_at	LOC_Os04g16822.1	1.160951	down	2.612091	down	1.129252	down	3.032511	down
OsAffx.32200.1.A1_x_at	LOC_Os04g16782.1	1.170736	up	2.880068	down	1.026736	up	2.460048	down
Os.54001.1.S1_at	LOC_Os02g08410.1	2.049604	down	1.216	down	1.001905	up	2.492318	down

OsAffx.17611.1.S1_at	LOC_Os09g09230.1	2.124378	up	1.586954	up	1.062759	down	3.371289	up
Os.19186.1.S1_at	LOC_Os02g57620.1	3.88733	down	1.120079	down	1.505895	down	4.354119	down
Os.15633.1.S1_at	LOC_Os10g35460.1	2.524567	up	1.156435	up	1.126358	up	2.919498	up
Os.5154.1.S1_at	LOC_Os07g49080.1	1.035791	down	3.169306	up	1.229346	down	3.059794	up
Os.8447.1.S1_at	LOC_Os04g51786.1	2.126599	down	1.336123	down	1.003332	down	2.841398	down
Os.5842.1.S1_at	LOC_Os12g19030.1	2.073154	down	2.036863	up	2.690478	down	1.017817	down
Os.12821.1.S1_at	LOC_Os03g17100.1	1.041069	up	1.442094	down	4.103559	up	1.385206	down
Os.4755.1.S1_at	LOC_Os12g34510.1	1.949412	up	1.205159	down	4.512065	up	1.617555	up
Os.55303.1.S1_at	LOC_Os03g14669.1	1.444777	down	1.027864	down	2.583189	up	1.485034	down
Os.21442.1.S1_at	LOC_Os02g03960.1	3.016931	down	2.011486	up	2.351775	down	1.499852	down
Os.15210.2.S1_x_at	LOC_Os04g53190.1	2.761445	up	1.090668	up	1.031498	up	3.011821	up
Os.42024.1.S1_at	LOC_Os01g56420.1	2.975903	down	2.253659	up	3.115667	down	1.320476	down
Os.6372.1.S1_at	LOC_Os03g10110.1	1.098882	up	2.454679	down	11.87452	up	2.233798	down
Os.12851.1.S1_at	LOC_Os01g14670.1	1.612483	up	5.362499	up	2.076973	down	8.646936	up
Os.47750.1.A1_at	LOC_Os01g50900.1	1.299303	up	1.307167	up	3.26279	up	1.698406	up
Os.27483.1.S1_at	LOC_Os08g13440.1	1.038702	up	3.702481	up	1.337328	down	3.845774	up
Os.2210.1.S1_at	LOC_Os08g08970.1	1.765403	down	4.045023	up	1.671849	down	2.291275	up
Os.9823.1.S1_at	LOC_Os09g02270.1	2.031519	down	1.594171	down	1.115204	up	3.238589	down
Os.24911.1.S1_at	LOC_Os03g62170.1	2.88696	down	1.057496	up	1.418424	down	2.729998	down
Os.14184.1.S1_at	LOC_Os06g11410.1	2.258201	down	1.28943	down	1.056913	up	2.911791	down
OsAffx.4171.1.S1_s_at	LOC_Os04g53680.1	3.132666	down	1.088258	up	1.426178	down	2.878605	down
Os.53969.1.S1_at	LOC_Os03g22700.1	2.653015	down	1.28273	down	1.017658	up	3.403101	down
Os.26853.1.A1_at	LOC_Os02g04010.1	1.266004	up	2.862009	up	1.335078	down	3.623315	up
Os.41883.1.S1_at	LOC_Os01g51540.1	5.591635	up	1.305624	down	1.950366	up	4.28273	up
Os.52752.1.S1_at	LOC_Os02g42740.1	2.951322	up	1.694682	up	1.179134	down	5.001554	up
Os.22935.1.S1_at	LOC_Os05g12040.1	2.688869	up	1.323201	up	1.03489	down	3.557915	up
Os.10596.1.S1_at	LOC_Os03g45619.1	2.731914	up	4.389689	up	4.022086	down	11.99225	up
Os.14951.1.S1_at	LOC_Os08g39730.1	7.677487	up	1.647192	up	1.117495	down	12.64629	up
Os.7674.1.S1_at	LOC_Os06g19070.1	2.120566	down	5.542815	up	7.998161	down	2.613838	up
Os.52292.1.S1_at	LOC_Os06g19070	2.793857	down	1.332997	down	1.296312	up	3.724204	down
Os.39214.1.S1_at	LOC_Os10g38090.1	3.472191	down	1.578152	down	1.008235	down	5.479646	down
Os.5369.1.S1_at	LOC_Os08g43440.1	2.231001	down	1.010679	down	1.107886	up	2.254825	down
OsAffx.7246.1.S1_x_at	LOC_Os11g29290.1	2.309673	up	1.615788	up	1.026113	down	3.731943	up
OsAffx.19825.1.S1_x_at	LOC_Os12g25660.1	2.211102	up	1.258513	up	1.012333	up	2.7827	up

Os.11417.1.S1_at	LOC_Os02g26810.1	3.371284	up	1.096613	up	1.555985	up	3.696995	up
Os.22485.1.A1_at	LOC_Os11g29720.1	2.840924	down	1.43408	down	1.364719	down	4.074112	down
Os.49566.1.S1_at	LOC_Os06g22340.1	2.495464	down	1.048764	down	1.232745	down	2.617153	down
Os.15692.1.S1_at	LOC_Os03g39760.1	1.115824	up	1.738641	down	7.289079	up	1.558168	down
Os.25153.1.S1_at	LOC_Os10g05490.1	1.180401	up	1.060387	up	2.571378	down	1.251681	up
Os.7370.1.S1_at	LOC_Os03g40540.1	1.372871	up	1.530243	down	3.080225	up	1.11463	down
Os.37093.1.S1_at	LOC_Os01g11340.1	1.068669	down	2.37055	up	1.584575	up	2.218228	up
Os.27525.1.A1_at	LOC_Os09g27500.1	1.451061	down	2.037535	down	1.005775	down	2.956587	down
Os.46776.1.S1_s_at	LOC_Os09g26960.1	1.692173	up	3.067248	up	1.306992	up	5.190314	up
OsAffx.30670.1.S1_at	LOC_Os10g34480.1	1.209981	down	2.356654	down	1.035189	up	2.851507	down
OsAffx.27093.1.S1_at	LOC_Os05g31040.1	3.467979	down	2.339746	up	4.675139	down	1.482203	down
Os.51127.2.S1_x_at	LOC_Os04g37820.1	1.774666	down	1.326154	up	4.049709	down	1.338205	down
Os.56058.1.S1_at	LOC_Os09g02440.1	2.0561	down	1.159015	down	1.131466	up	2.383051	down
Os.9462.2.S1_at	LOC_Os12g41715.1	2.960147	down	2.285179	up	3.792972	down	1.295368	down
OsAffx.18404.1.S1_at	LOC_Os10g26110.1	4.683367	up	1.852105	up	1.073014	down	8.674086	up
Os.10651.1.S1_at	LOC_Os02g41904.1	4.528579	up	1.039766	down	1.543643	up	4.355384	up
Os.19038.1.S1_at	LOC_Os04g14150.1	2.137068	up	1.022639	down	1.25862	down	2.089757	up
Os.24364.1.A1_at	LOC_Os02g51860.1	1.763094	up	2.156412	up	1.052888	up	3.801957	up
OsAffx.31140.1.S1_at	LOC_Os11g26570.1	2.680283	up	10.56313	down	20.26267	up	3.94105	down
Os.51718.1.S1_at	LOC_Os11g26780.1	6.487113	up	7.00066	down	11.77584	up	1.079164	down
Os.7478.1.S1_at	LOC_Os06g07120.1	1.2378	up	1.116031	down	2.953776	up	1.109109	up
Os.54410.1.S1_at	LOC_Os06g13720.1	2.720879	up	7.231846	down	12.48263	up	2.657908	down
Os.6089.1.S1_at	LOC_Os04g15920.1	2.217047	up	1.891381	up	1.992266	down	4.193279	up
Os.8684.1.S1_a_at	LOC_Os02g09490.1	2.457512	up	1.853637	up	1.003538	down	4.555335	up
Os.53814.1.S1_at	LOC_Os09g04050.1	1.774762	up	6.433999	up	2.42208	down	11.41882	up
Os.20865.1.S1_at	LOC_Os05g35520.1	2.183126	down	1.080293	up	1.11659	up	2.020864	down
OsAffx.4527.1.S1_s_at	LOC_Os05g35470.1	1.858068	down	1.808696	up	3.716535	down	1.027297	down
Os.54545.1.S1_at	LOC_Os07g41060.1	2.775078	up	1.121877	up	1.406502	down	3.113297	up
Os.12381.1.S1_x_at	LOC_Os07g44250.1	1.016151	down	8.265244	up	1.875871	down	8.13387	up
Os.12381.1.S1_s_at	LOC_Os07g44250.1	1.110234	down	9.103099	up	1.80771	down	8.199263	up
Os.49763.2.S1_at	LOC_Os03g17220.1	1.244463	up	5.535307	up	1.267539	down	6.888487	up
Os.51866.1.S1_at	LOC_Os11g10770.1	3.946013	up	2.805291	down	3.11898	up	1.406632	up
Os.7468.1.S1_at	LOC_Os06g48700.1	2.108268	down	1.317321	down	1.061945	up	2.777267	down
Os.13051.1.S1_at	LOC_Os12g04980.1	2.483889	down	1.055689	up	1.025366	down	2.352861	down

Os.10963.1.S1_at	LOC_Os01g55030.1	2.483844 down	1.027537 down	1.202886 down	2.552242 down
Os.51942.1.S1_at	LOC_Os12g30510.1	2.824376 down	1.065102 up	1.391357 down	2.651742 down
Os.27407.1.A1_at	LOC_Os01g62200	1.466075 up	2.96688 up	1.276781 down	4.349668 up
OsAffx.28093.1.S1_at	LOC_Os06g44040.1	1.431943 up	2.025214 up	1.059585 up	2.899989 up
OsAffx.17883.1.S1_at	LOC_Os09g24954.1	1.609583 down	2.226966 down	1.086461 down	3.584487 down
Os.49632.1.S1_a_at	LOC_Os09g33460.1	1.074248 down	2.167224 down	1.063084 up	2.328138 down
Os.55694.1.S1_at	LOC_Os02g42920.1	2.386255 down	1.08211 up	1.104901 up	2.205188 down
Os.2250.1.S1_a_at	LOC_Os05g49440.1	1.15876 up	1.737964 down	6.297319 up	1.499849 down
Os.22577.2.S1_x_at	LOC_Os01g32770.1	1.843795 down	1.365656 up	4.655178 down	1.350117 down
OsAffx.19357.1.S1_at	LOC_Os11g43790.1	1.532095 up	4.118962 down	23.00658 up	2.688452 down
Os.5697.1.S1_at	LOC_Os07g33270.1	1.042208 down	4.056003 up	1.923314 down	3.891738 up
Os.22711.1.A1_at	LOC_Os03g06350.1	1.264269 down	1.514907 up	2.193132 up	1.198248 up
Os.10564.1.A1_at	LOC_Os10g35770.3	2.23901 down	1.56845 down	1.564915 up	3.511776 down
Os.57519.1.A1_at	LOC_Os01g14410.1	2.262786 up	3.101757 down	3.386906 up	1.370769 down
Os.27562.1.S1_at	LOC_Os05g01520.1	2.339691 down	1.357189 down	1.031689 up	3.175402 down
Os.37996.1.S1_at	LOC_Os07g43800.1	3.282517 down	2.054012 up	2.914382 down	1.5981 down
Os.6965.1.S1_at	LOC_Os03g19720	1.390594 down	3.975527 up	1.395983 down	2.858869 up
Os.209.1.S1_at	LOC_Os01g16920.1	1.569444 up	1.50093 down	4.193875 up	1.045648 up
OsAffx.26423.1.S1_at	LOC_Os04g41970.1	4.175685 up	1.009627 up	1.537324 up	4.215881 up
Os.54551.1.S1_at	LOC_Os09g23084.1	1.464977 up	2.27427 up	1.050056 up	3.331754 up
OsAffx.30751.1.S1_at	LOC_Os12g16350.1	2.544074 up	1.155752 down	1.237122 up	2.201228 up
OsAffx.22469.1.S1_x_at	LOC_Os07g07040.1	2.249822 up	2.850681 down	3.740674 up	1.26707 down
Os.12429.1.S1_at	LOC_Os07g07040.1	1.765233 up	1.696545 down	4.025639 up	1.040487 up
Os.3417.1.S1_at	LOC_Os01g70860.1	1.157692 up	2.869379 down	7.123366 up	2.478533 down
OsAffx.21616.1.S1_s_at	LOC_Os01g54890.1	1.411166 down	2.920465 up	1.063016 up	2.06954 up
OsAffx.20436.1.S1_x_at	LOC_Os10g22600.1	3.479667 up	1.10941 up	1.004085 down	3.860375 up
Os.49519.1.S1_at	LOC_Os07g46480.1	3.760025 up	1.157626 up	1.145964 up	4.352704 up
Os.53052.1.S1_at	LOC_Os01g67820.1	1.016752 down	2.573499 up	1.220052 down	2.531097 up
Os.53817.1.S1_at	LOC_Os08g13570.1	1.131102 down	2.921324 up	1.435054 down	2.582724 up
Os.47503.1.S1_at	LOC_Os03g63720.1	2.178661 down	1.341149 down	1.016042 down	2.92191 down
Os.2938.1.S1_at	LOC_Os10g39640.1	4.631555 up	1.064404 up	1.432705 up	4.929845 up
Os.2938.1.S1_x_at	LOC_Os10g39640.1	4.487301 up	1.074043 up	1.451029 up	4.819553 up
Os.2367.1.S1_at	LOC_Os03g21820.1	1.760871 up	1.392915 down	5.523145 up	1.264162 up
Os.55253.1.S1_at	LOC_Os05g11610.1	54.08567 up	1.139315 down	1.337871 up	47.4721 up

Os.54698.1.S1_at	LOC_Os03g51530.1	3.552613	down	5.923947	down	4.547498	up	21.0455	down
OsAffx.6171.1.S1_at	LOC_Os09g04339.1	2.253501	up	22.68032	down	25.66281	up	10.06448	down
OsAffx.26838.1.S1_at	LOC_Os05g11600.1	11.57416	up	1.356763	up	1.058406	up	15.70339	up
Os.7078.1.S1_at	LOC_Os02g56930.1	8.478166	up	1.080934	up	1.197224	up	9.164341	up
OsAffx.18503.1.S1_at	LOC_Os10g33450.1	13.32844	up	1.217985	down	1.911704	up	10.94303	up
Os.10590.1.S1_s_at	LOC_Os08g01370	6.331863	up	21.85153	down	37.93533	up	3.451043	down
Os.6683.1.S1_at	LOC_Os01g58130.1	9.138741	up	1.144305	up	1.608805	up	10.4575	up
OsAffx.2803.1.S1_at	LOC_Os02g30190.1	5.186719	up	1.30754	up	1.399536	up	6.781845	up
Os.18712.1.S1_at	LOC_Os08g40940.1	4.509171	up	1.372491	up	1.250017	up	6.188798	up
Os.12052.1.S1_at	LOC_Os10g29514.1	2.03	down	1.372695	down	1.730079	up	2.786571	down
Os.50381.1.S1_at	LOC_Os11g09150.1	3.585654	down	1.838743	down	1.028211	down	6.593097	down
OsAffx.25404.1.S1_at	LOC_Os03g37770.1	5.353383	up	1.095273	down	1.664348	up	4.887714	up
Os.6224.2.S1_at	LOC_Os03g32150	3.490975	down	1.435142	down	1.12304	down	5.010045	down
Os.16563.1.S1_at	LOC_Os07g38840.1	2.366138	up	1.945562	up	1.293008	down	4.603469	up
Os.25117.1.A1_at	LOC_Os01g07890.1	2.968635	down	1.102911	down	1.026712	up	3.274139	down
Os.38760.1.S1_at	LOC_Os05g28090.2	3.564502	down	1.656264	up	1.19384	down	2.152133	down
Os.52183.1.S1_at	LOC_Os02g10400.1	2.458995	down	1.228499	down	1.208699	up	3.020874	down
Os.34118.1.S1_at	LOC_Os10g37870	4.81638	down	1.14025	up	1.670934	down	4.223968	down
Os.5593.1.S1_at	LOC_Os01g32380.1	4.547839	up	1.231002	up	1.59248	up	5.598398	up
Os.57191.1.S1_at	LOC_Os06g05470.1	3.710025	up	1.336474	down	1.3428	up	2.77598	up
Os.13482.1.S1_x_at	LOC_Os02g30600	2.273922	up	1.497802	up	1.207169	down	3.405885	up
Os.37320.1.S1_at	LOC_Os03g62950.1	2.050772	down	1.183385	down	1.328963	up	2.426853	down
Os.33081.1.S1_at	LOC_Os03g39740.1	2.150696	down	1.15936	down	1.266407	up	2.49343	down
Os.54454.1.S1_at	LOC_Os12g25720.1	3.194022	up	1.410352	up	1.196246	up	4.504694	up
Os.10491.1.S1_at	LOC_Os03g04690.1	2.702862	down	1.34509	up	1.018169	down	2.009428	down
Os.26772.2.S1_at	LOC_Os10g38980.1	2.735403	down	1.360677	up	1.042749	down	2.010324	down
Os.53962.1.S1_at	LOC_Os10g18750.1	2.500057	down	1.183093	down	1.045337	up	2.9578	down
OsAffx.16228.1.S1_at	LOC_Os07g12540.1	2.262932	down	1.248797	down	1.153566	up	2.825942	down
OsAffx.28288.1.S1_at	LOC_Os07g05390.1	3.239909	down	1.064138	down	1.255854	down	3.447709	down
Os.27778.2.S1_at	LOC_Os01g40000.1	4.093771	down	1.308458	up	1.60901	down	3.128698	down
Os.49301.1.A1_at	LOC_Os10g26859.1	4.569868	down	1.265188	up	1.812846	down	3.612006	down
OsAffx.27346.1.S1_at	LOC_Os05g46790.1	3.292466	up	1.28007	down	1.309815	up	2.572099	up
Os.8811.1.S1_at	LOC_Os07g42410.1	2.053503	down	1.135185	down	1.223617	up	2.331106	down
Os.26406.1.S1_at	LOC_Os03g03440.1	4.063588	up	1.089268	down	1.632066	up	3.730566	up

Os.8290.1.S1_at	LOC_Os03g26000.1	2.351044	down	1.201594	down	1.048631	up	2.825001	down
Os.53970.1.S1_at	LOC_Os05g46770.1	3.036807	down	1.063754	up	1.24	down	2.854801	down
Os.27759.1.S1_at	LOC_Os01g46720.1	2.764547	down	1.593104	down	1.133022	down	4.40421	down
Os.54220.1.S1_at	LOC_Os12g25720.1	2.343893	down	1.482536	down	1.039408	up	3.474906	down
Os.53241.1.S1_at	LOC_Os01g46530.1	2.622598	down	1.113299	down	1.106185	down	2.919735	down
Os.10525.1.S1_at	LOC_Os10g12750.1	2.012045	up	1.661027	up	1.147131	down	3.34206	up
OsAffx.16741.1.S1_s_at	LOC_Os07g47070	2.743896	down	1.055185	down	1.191032	down	2.89532	down
Os.10743.1.S1_at	LOC_Os06g33200.1	2.392679	down	1.04537	down	1.047614	down	2.501235	down
Os.54497.1.S1_at	LOC_Os04g42200.1	2.437204	down	1.035448	down	1.081906	down	2.523599	down
Os.53455.1.S1_at	LOC_Os12g44130.1	3.19782	down	1.051756	down	1.420912	down	3.363325	down
Os.36647.1.S1_x_at	LOC_Os04g55270.1	3.064844	down	1.117818	down	1.365096	down	3.425939	down
Os.12999.1.S1_at	LOC_Os04g54620.1	2.452387	up	1.162206	down	1.112422	up	2.110114	up
Os.48986.1.S1_s_at	LOC_Os05g04000.1	3.274208	up	1.48186	down	1.485694	up	2.209526	up
Os.7770.1.S1_at	LOC_Os05g05030.1	2.177576	up	1.862696	up	1.002418	down	4.056161	up
Os.51166.1.S1_at	LOC_Os04g28060.1	2.486255	down	1.558725	down	1.14615	down	3.875389	down
Os.4923.1.S1_at	LOC_Os04g21710.1	2.215571	up	1.565708	up	1.021648	up	3.468937	up
Os.8721.1.S1_at	LOC_Os09g02130.1	2.259276	down	1.137724	down	1.041915	down	2.570433	down
Os.9485.1.S1_at	LOC_Os09g32270.1	3.444653	down	1.210792	down	1.590964	down	4.170757	down
OsAffx.4002.1.S1_s_at	LOC_Os04g35580.1	4.197499	up	1.063866	down	1.944668	up	3.945514	up
Os.5066.1.S1_at	LOC_Os01g12000.1	4.154934	up	1.021159	down	1.933333	up	4.068842	up
Os.10797.1.S1_at	LOC_Os02g01380.1	3.158634	down	1.141093	up	1.474174	down	2.768078	down
Os.8550.1.S1_x_at	LOC_Os05g46630.1	2.677139	down	1.143275	down	1.250369	down	3.060706	down
Os.27569.3.S1_at	LOC_Os11g02720.1	2.887481	down	1.466253	down	1.352799	down	4.233778	down
Os.27319.1.A1_at	LOC_Os01g26370.1	2.792084	down	1.079836	down	1.31019	down	3.014994	down
OsAffx.24464.1.S1_s_at	LOC_Os02g27740.1	2.547907	down	1.302133	down	1.200074	down	3.317713	down
Os.14243.1.S1_at	LOC_Os05g34760.1	2.208327	down	1.044714	down	1.040167	down	2.30707	down
Os.49422.1.S1_x_at	LOC_Os05g19470.1	2.554016	down	1.145011	down	1.204285	down	2.924376	down
Os.53334.2.S1_x_at	LOC_Os07g16030.1	3.206568	down	1.180261	down	1.512375	down	3.784588	down
Os.35316.1.S1_at	LOC_Os02g06790.1	2.391396	down	1.195961	down	1.129177	down	2.860016	down
Os.51826.1.S1_at	LOC_Os05g03490.1	3.097899	down	1.363577	up	1.462991	down	2.271891	down
Os.38992.1.A1_x_at	LOC_Os08g45120.1	9.426829	up	3.792055	down	4.465855	up	2.485942	up
Os.38992.1.A1_x_at	LOC_Os08g45120.1	9.426829	up	3.792055	down	4.465855	up	2.485942	up
Os.9536.1.S1_at	LOC_Os03g04930.1	2.060113	up	1.610551	up	1.021979	down	3.317916	up
Os.32478.1.S1_at	LOC_Os01g46400.1	2.548166	up	1.561271	up	1.214338	up	3.978377	up

Os.1423.1.S1_at	LOC_Os01g32130.1	2.78234	down	1.346518	down	1.333654	down	3.746471	down
Os.53016.1.S1_at	LOC_Os03g16580.1	2.165357	down	1.020875	down	1.039254	down	2.21056	down
Os.49292.1.S1_at	LOC_Os09g16714	2.665203	down	1.187857	up	1.28122	down	2.243707	down
Os.8937.1.S1_at	LOC_Os10g35150.1	2.098419	down	1.363022	down	1.012543	down	2.860192	down
Os.53407.1.S1_at	LOC_Os08g06210.1	2.30834	up	1.171287	up	1.114571	up	2.703729	up
Os.53407.1.S1_x_at	LOC_Os08g06210.1	2.266595	up	1.336969	up	1.09531	up	3.030367	up
Os.15813.1.S1_at	LOC_Os03g56610.1	2.884864	up	1.043903	down	1.404621	up	2.763535	up
Os.33610.2.A1_at	LOC_Os01g59320.1	2.251402	down	1.386713	down	1.096293	down	3.122048	down
OsAffx.28461.1.S1_at	LOC_Os07g15500.1	3.112137	down	1.001379	down	1.528163	down	3.116428	down
Os.28176.2.S1_x_at	LOC_Os01g61680.1	3.793317	up	1.138691	down	1.867	up	3.331297	up
OsAffx.23976.1.S1_at	LOC_Os01g68800.1	2.484455	down	1.570874	down	1.227322	down	3.902765	down
Os.22678.1.A1_s_at	LOC_Os02g37834.2	2.581587	down	1.378486	down	1.276428	down	3.558681	down
Os.21264.1.S1_s_at	LOC_Os12g27994.1	2.420242	down	1.06594	up	1.197432	down	2.270524	down
Os.12432.1.S1_a_at	LOC_Os04g53530.1	2.501712	down	1.118646	up	1.24123	down	2.236375	down
Os.57568.1.S1_at	LOC_Os09g24210.1	2.027254	down	1.476398	down	1.013079	down	2.993034	down
Os.52298.1.S1_at	LOC_Os06g02960.1	2.488611	down	3.949824	up	4.882933	down	1.587161	up
Os.4995.1.S1_at	LOC_Os03g55776.1	2.335963	down	2.391035	up	3.803619	down	1.023575	up
OsAffx.26304.1.S1_s_at	LOC_Os04g33310	2.439403	down	2.171476	up	3.848993	down	1.123385	down
Os.11920.1.S1_s_at	LOC_Os04g39320.1	2.889554	down	3.283123	up	4.389419	down	1.136204	up
Os.16964.1.S1_x_at	LOC_Os03g61200.1	2.657722	down	2.035142	up	3.809257	down	1.305915	down
Os.8947.1.S1_at	LOC_Os03g05910.1	2.234633	down	2.059736	up	3.112752	down	1.084912	down
Os.17174.1.S1_at	LOC_Os04g44290.1	2.058076	down	3.610732	up	2.817702	down	1.754422	up
Os.30528.1.S1_at	LOC_Os08g31860.1	2.033923	up	3.16808	down	2.612282	up	1.55762	down
Os.49538.1.S1_at	LOC_Os03g43010.1	2.958518	down	2.030999	up	3.765435	down	1.456681	down
Os.27617.1.S1_at	LOC_Os05g44580.1	2.268875	down	2.322504	up	2.792972	down	1.023637	up
Os.6901.1.S1_at	LOC_Os08g39230.1	3.154999	up	4.085071	down	3.570696	up	1.294793	down
Os.37876.1.S1_at	LOC_Os10g39920.1	2.760681	up	2.495549	down	3.037148	up	1.106242	up
Os.54121.1.S1_s_at	LOC_Os04g02070.1	3.131289	down	2.331232	up	3.347672	down	1.34319	down
Os.55250.1.S1_at	LOC_Os09g32000.1	3.568935	down	3.81814	up	3.72436	down	1.069826	up
Os.55332.1.S1_at	LOC_Os09g04100.1	1.667393	up	6.658877	down	27.74936	up	3.993584	down
Os.16163.1.S1_at	LOC_Os02g15740.1	1.025761	down	3.484889	down	12.00267	up	3.574664	down
Os.51758.2.S1_at	LOC_Os07g47840.1	1.923021	up	1.065034	down	10.52896	up	1.805596	up
Os.51092.1.S1_at	LOC_Os11g24824.1	1.464831	down	1.281904	down	3.59044	up	1.877771	down
Os.28200.1.S1_x_at	LOC_Os03g61160.1	1.198656	down	8.003124	down	3.651195	up	9.592992	down

Os.55394.1.S1_at	LOC_Os03g52410.1	1.073859	up	11.51425	up	4.067321	down	12.36468	up
Os.45928.1.S1_at	LOC_Os01g32460.1	1.640872	up	6.233746	up	2.576106	down	10.22878	up
Os.55266.1.S1_at	LOC_Os07g10620.1	1.42909	down	1.096299	down	2.34334	up	1.56671	down
OsAffx.25073.1.S1_x_at	LOC_Os03g12700.1	1.101427	down	1.145286	up	2.872688	up	1.03982	up
Os.12995.1.S1_at	LOC_Os04g59420.1	1.139117	up	1.536607	down	3.56245	up	1.348946	down
Os.48260.1.S1_at	LOC_Os02g52490.1	1.15157	down	1.290179	up	2.662283	up	1.120365	up
Os.9995.1.S1_at	LOC_Os03g16874.1	1.282586	up	1.229082	down	3.736886	up	1.043532	up
OsAffx.17158.1.S1_at	LOC_Os08g25850.1	1.1557	up	1.602927	down	3.290822	up	1.386975	down
OsAffx.18633.1.S1_s_at	LOC_Os12g02050.1	1.038792	down	1.289179	down	2.61226	up	1.339189	down
Os.34174.1.S1_at	LOC_Os04g38790.1	1.212064	up	4.76535	up	2.22046	down	5.775909	up
Os.11602.1.S1_at	LOC_Os09g38090.1	1.151658	down	1.12356	down	2.309192	up	1.293957	down
Os.8253.1.S1_at	LOC_Os10g09930.1	1.113012	up	1.245001	down	2.865726	up	1.118587	down
Os.30754.1.S1_at	LOC_Os03g35920	1.143187	up	1.228525	down	2.895742	up	1.07465	down
OsAffx.26343.1.S1_x_at	LOC_Os04g35490.1	1.649757	up	1.112433	up	4.003951	up	1.835245	up
Os.9929.1.S1_at	LOC_Os03g60509.1	1.273453	down	1.441297	up	2.898547	down	1.131803	up
OsAffx.20051.1.S1_at	LOC_Os12g40330.1	1.11061	up	1.316015	up	2.500704	up	1.461579	up
Os.7044.1.S1_at	LOC_Os12g05655.1	1.709802	up	1.301048	down	3.8474	up	1.314173	up
Os.56140.1.S1_x_at	LOC_Os01g38100.1	1.031655	up	1.504234	up	2.321306	up	1.551851	up
OsAffx.30275.1.S1_at	LOC_Os10g04380.1	1.705466	up	1.078448	up	3.792233	up	1.839256	up
OsAffx.24777.1.S1_at	LOC_Os02g48150.1	1.671241	up	1.387534	down	3.653507	up	1.204469	up
OsAffx.17329.1.S1_s_at	LOC_Os08g34510	1.386762	up	1.457368	down	3.031298	up	1.050914	down
Os.45924.1.S1_at	LOC_Os07g45460.1	1.396583	up	1.040725	up	3.003268	up	1.453459	up
Os.51772.1.S1_at	LOC_Os01g26130.1	1.951718	up	1.40938	down	4.183351	up	1.384806	up
Os.56237.1.S1_at	LOC_Os02g30410.2	1.64322	down	1.708271	up	3.44923	down	1.039588	up
Os.55461.1.S1_at	LOC_Os03g26490.1	1.491918	up	1.462976	down	3.112255	up	1.019783	up
Os.37687.1.A1_at	LOC_Os05g04150.1	1.3953	up	1.006988	down	2.825945	up	1.385618	up
OsAffx.3968.1.S1_at	LOC_Os04g31710.1	1.225231	up	1.081543	down	2.461672	up	1.132855	up
OsAffx.5283.1.S1_at	LOC_Os07g10440.1	1.633092	up	3.898099	up	1.202068	down	6.365954	up
Os.47369.1.A1_at	LOC_Os10g39660.1	1.231145	up	3.40706	up	1.440983	down	4.194586	up
OsAffx.31976.1.S1_s_at	LOC_Os12g32290.1	1.208532	down	4.713011	down	1.461205	up	5.695825	down
Os.38169.1.S1_a_at	LOC_Os02g43100.1	1.778485	up	3.275195	up	1.054952	up	5.824884	up
Os.9976.1.S1_at	LOC_Os06g33180.2	1.317378	up	2.793781	up	1.224061	down	3.680465	up
AFFX-Os_28SrRNA_at	LOC_Os09g01000.1	1.346212	up	2.962881	down	1.170121	down	2.200902	down
Os.55247.1.S1_at	LOC_Os05g50100.1	1.475359	up	2.406928	up	1.044641	down	3.551083	up

Os.54463.1.S1_at	LOC_Os05g48680.1	1.704387	down	2.053282	down	1.111942	down	3.499586	down
Os.46725.1.S1_at	LOC_Os10g05130.1	1.860108	up	2.581991	up	1.255549	up	4.802781	up
Os.55647.1.A1_at	LOC_Os01g06882.1	1.979786	up	2.296082	up	1.380585	up	4.545751	up
Os.7521.1.S1_at	LOC_Os06g11990.1	1.29229	up	2.879648	up	1.040763	down	3.721341	up
Os.30059.1.S1_at	LOC_Os01g60420.1	1.55061	up	2.587969	up	1.153529	up	4.012929	up
Os.12937.1.S1_at	LOC_Os04g51130.1	1.642591	up	2.377595	up	1.225768	up	3.905416	up
OsAffx.10944.1.S1_at	LOC_Os01g06882.1	1.585571	up	2.797466	up	1.261144	up	4.43558	up
Os.52266.1.S1_at	LOC_Os07g14910.1	1.250303	down	3.256662	up	1.565985	down	2.6047	up
OsAffx.31976.1.S1_at	LOC_Os12g32290.1	1.145189	down	2.272801	down	1.090464	up	2.602788	down
Os.8439.1.S1_a_at	LOC_Os03g48626.1	1.455048	down	2.061497	down	1.169711	down	2.999578	down
OsAffx.27605.1.S1_s_at	LOC_Os06g13190.1	1.20047	up	2.656675	up	1.028591	down	3.189259	up
Os.43043.1.S1_at	LOC_Os01g19880.1	1.950316	up	2.010979	up	1.581822	up	3.922044	up
OsAffx.2403.1.S1_at	LOC_Os01g64440.1	1.575595	up	3.436784	up	1.93848	up	5.414978	up
Os.23649.1.S1_a_at	LOC_Os03g61620.1	1.154386	up	2.162735	up	1.043437	down	2.49663	up
Os.51385.1.S1_at	LOC_Os02g46620.1	1.521649	up	2.224878	up	1.271903	up	3.385483	up
Os.27598.1.S1_at	LOC_Os03g16470.1	1.132245	up	2.254368	up	1.053713	down	2.552496	up
OsAffx.15770.1.S1_at	LOC_Os06g36070.1	1.361914	up	2.118188	up	1.607705	up	2.88479	up
OsAffx.27338.1.S1_at	LOC_Os05g46395.1	1.536584	down	2.330416	down	1.323494	down	3.58088	down
Os.39933.1.S1_x_at	LOC_Os06g02440.1	1.624477	down	3.286296	down	1.401229	down	5.338512	down
OsAffx.15233.1.S1_x_at	LOC_Os06g02440	1.124348	down	2.371061	down	1.266684	down	2.665897	down
OsAffx.23031.1.S1_s_at	LOC_Os01g05460.1	1.048702	down	2.216557	down	1.069232	up	2.324508	down
Os.49583.1.S1_at	LOC_Os03g15080.1	1.129056	up	2.951253	up	1.261437	up	3.332129	up
Os.5318.1.S1_a_at	LOC_Os10g42610.1	1.434485	down	2.234812	down	1.596419	down	3.205803	down
Os.56918.1.S1_at	LOC_Os03g62330.1	1.385004	down	2.799296	down	1.526646	down	3.877036	down
Os.21260.1.S1_at	LOC_Os03g08580.1	1.461251	down	3.975216	down	1.387133	down	5.808787	down
Os.16214.1.S1_at	LOC_Os11g05490.1	1.298829	down	3.172432	down	1.349058	down	4.120447	down
Os.37621.3.S1_at	LOC_Os01g02334	1.770036	up	3.038538	up	1.728222	up	5.378321	up
Os.4683.2.S1_at	LOC_Os01g42520.1	1.095731	up	2.255582	up	1.072121	up	2.471511	up
Os.27186.1.S1_at	LOC_Os11g29840.1	1.142613	down	2.96079	up	1.158358	down	2.591246	up
Os.15580.1.S1_at	LOC_Os03g42600.1	1.136196	down	2.417901	down	1.130732	down	2.74721	down
Os.5601.1.S1_at	LOC_Os09g32290.1	2.863164	down	2.62481	up	3.370547	down	1.090808	down
Os.17961.1.S1_a_at	LOC_Os02g20560.1	3.625102	up	4.538509	up	3.292417	down	16.45256	up
Os.22627.1.S1_at	LOC_Os03g57460.1	2.038563	up	2.545139	up	2.125829	down	5.188426	up
Os.54555.1.S1_at	LOC_Os05g07060.1	1.414187	up	24.02192	up	4.397976	down	33.97147	up

Os.5500.1.S1_s_at	LOC_Os08g23180.1	1.076423	up	2.695032	up	1.310015	down	2.900995	up
Os.10125.1.S1_a_at	LOC_Os02g48560.1	1.287985	up	2.008171	up	1.021043	up	2.586494	up
Os.10829.1.S1_at	LOC_Os01g04260.1	2.257943	down	3.011	up	2.754845	down	1.333514	up
Os.5431.1.S1_at	LOC_Os04g49690.1	2.136046	up	1.84171	up	1.061552	up	3.933978	up
Os.15679.1.S1_s_at	LOC_Os08g35210.1	1.532966	down	1.270187	up	3.340825	down	1.206882	down
Os.14145.1.A1_at	LOC_Os04g36720.1	3.037409	down	1.241274	up	1.461499	down	2.447008	down
Os.12096.4.S1_s_at	LOC_Os11g01530.1	1.080792	down	1.380072	up	4.205704	down	1.276908	up
Os.12096.3.S1_a_at	LOC_Os11g01530.1	1.242037	down	1.686746	up	3.715612	down	1.358048	up
Os.12096.3.S1_x_at	LOC_Os11g01530.1	1.167271	down	1.391674	up	3.302743	down	1.192246	up
Os.9859.1.S1_at	LOC_Os01g08000.2	1.685921	down	2.789363	down	1.025845	up	4.702645	down
Os.50805.1.S1_at	LOC_Os08g20130.1	2.646939	up	1.618824	up	1.51095	down	4.284927	up
Os.31778.1.S1_x_at	LOC_Os01g61610.1	11.76322	up	1.511546	down	1.486058	up	7.782244	up
Os.31778.2.S1_x_at	LOC_Os01g61610.1	12.39472	up	1.563432	down	1.596587	up	7.927891	up
Os.31778.2.S1_at	LOC_Os01g61610.1	8.393523	up	1.482654	down	1.628843	up	5.661147	up
Os.53217.1.S1_x_at	LOC_Os03g03034.3	3.265436	up	1.025666	up	1.086506	up	3.349248	up
Os.11122.1.S1_at	LOC_Os08g37456.1	2.215722	down	1.410726	down	1.046603	up	3.125776	down
Os.52646.1.S1_at	LOC_Os05g03640.1	2.042538	up	1.473694	up	1.019924	up	3.010077	up
Os.28435.4.S1_x_at	LOC_Os10g40934.1	1.743835	down	2.62906	down	1.120778	down	4.584646	down
Os.28435.4.S1_at	LOC_Os10g40934.7	1.260588	down	2.394993	down	1.100555	down	3.019099	down
Os.9073.1.S1_at	LOC_Os01g57460.1	2.038544	up	1.18781	up	1.062709	down	2.421404	up
Os.32462.1.S1_a_at	LOC_Os03g16290.1	2.796748	up	1.027785	up	1.263168	up	2.874457	up
OsAffx.26679.1.S1_at	LOC_Os05g01610.1	1.790146	down	2.018962	down	1.339806	down	3.614237	down
Os.54926.1.S1_at	LOC_Os03g51880.1	3.154592	down	1.20064	down	1.398189	down	3.787527	down
Os.48981.1.S1_at	LOC_Os07g40240.1	1.613927	up	1.364964	down	3.989651	up	1.182395	up
Os.16248.1.S1_at	LOC_Os02g56250.1	1.176406	up	2.033755	up	1.036731	up	2.392522	up
Os.27247.1.S1_at	LOC_Os06g50950.1	2.103611	up	7.2349	up	4.452759	down	15.21941	up
Os.38245.1.S1_at	LOC_Os01g22640.1	2.097909	up	2.918936	up	2.278033	down	6.123662	up
Os.37457.1.S1_at	LOC_Os07g44780.1	2.014291	up	1.679089	up	1.320684	down	3.382173	up
Os.46844.1.S1_at	LOC_Os10g25400.1	4.614318	up	1.110697	down	1.966818	up	4.154433	up
Os.30473.1.S1_at	LOC_Os01g11150.1	1.92916	up	2.317076	up	1.341172	up	4.470012	up
OsAffx.26840.1.S1_s_at	LOC_Os05g11650.1	2.970903	down	1.112287	down	1.253859	down	3.304496	down
Os.52185.1.A1_at	LOC_Os04g35790.1	4.188221	down	2.227493	up	4.05219	down	1.88024	down
OsAffx.25749.1.S1_at	LOC_Os03g57880.1	2.828715	up	1.115773	down	1.312421	up	2.535205	up
Os.49920.2.S1_at	LOC_Os03g61780.1	2.501757	down	1.084213	up	1.02345	up	2.307442	down

Os.17437.1.S1_at	LOC_Os01g53390.1	2.144961	down	2.454603	up	2.032708	down	1.144358	up
Os.48875.1.S1_at	LOC_Os03g50490.1	2.447334	up	4.570608	down	3.027363	up	1.867586	down
Os.5441.1.S1_at	LOC_Os10g38470.1	4.041402	down	1.181218	down	1.347557	down	4.773777	down
Os.9101.1.S1_at	LOC_Os10g38780.1	2.853786	down	2.297942	up	3.409739	down	1.241888	down
OsAffx.11535.1.S1_at	LOC_Os01g49720.1	1.226452	up	1.19678	down	3.370073	up	1.024794	up
Os.9775.1.S1_at	LOC_Os10g38710.1	1.178223	down	2.94638	up	1.174131	down	2.500698	up
Os.57475.1.S1_x_at	LOC_Os02g38920.1	2.534183	up	2.071914	down	2.79049	up	1.223112	up
Os.8022.1.S1_at	LOC_Os03g14010.1	4.72835	up	1.063881	up	1.87094	up	5.0304	up
OsAffx.27816.1.S1_at	LOC_Os06g25010.1	3.993343	up	1.232467	up	1.527718	up	4.921666	up
Os.4867.1.S1_at	LOC_Os08g40680.1	1.449552	up	3.359077	down	6.758835	up	2.31732	down
Os.28531.1.S1_at	LOC_Os01g64100.1	1.578982	up	2.464163	up	1.469073	up	3.890869	up
Os.4159.1.S1_at	LOC_Os01g51570.1	2.016268	up	1.226102	up	1.23231	down	2.472151	up
Os.11843.1.S1_at	LOC_Os04g49110.1	1.370554	up	2.979967	up	1.19648	down	4.084207	up
Os.7276.1.A1_s_at	LOC_Os08g44015.1	2.137664	down	1.014788	up	1.040297	down	2.106513	down
Os.10534.1.S1_at	LOC_Os05g09704.1	2.167186	up	1.275658	up	1.307438	down	2.764589	up
Os.24865.1.A1_at	LOC_Os05g09724.1	1.660791	up	16.90237	up	6.479135	down	28.0713	up
Os.11265.1.S1_at	LOC_Os03g16670.1	2.05255	up	2.289119	down	3.724003	up	1.115256	down
Os.10576.1.S1_at	LOC_Os02g33550.1	1.824157	up	12.30746	up	4.855301	down	22.45074	up
Os.40417.1.A1_at	LOC_Os07g14740.1	1.915994	down	4.174965	up	1.963955	down	2.179007	up
Os.12082.1.S1_at	LOC_Os04g39350.1	3.101336	down	2.405877	up	2.466077	down	1.289067	down
OsAffx.24612.1.S1_at	LOC_Os02g37300.1	2.820068	down	2.875538	up	2.288442	down	1.01967	up
Os.55527.1.S1_at	LOC_Os08g31340.1	1.30079	up	1.350277	down	5.066758	up	1.038044	down
Os.13543.1.S1_at	LOC_Os12g23280.1	1.343909	up	2.810082	up	1.145637	down	3.776495	up
Os.9216.1.S1_at	LOC_Os03g26210.1	2.339619	up	9.889102	down	8.763855	up	4.2268	down
Os.21231.1.S1_at	LOC_Os01g38610.1	3.027265	down	1.256377	down	1.047134	up	3.803386	down
Os.12498.1.S1_at	LOC_Os01g72370.1	1.306386	up	9.50361	down	4.664955	up	7.274733	down
Os.7116.1.S1_at	LOC_Os03g53020.1	1.035298	down	5.841931	up	1.719761	down	5.642757	up
Os.30044.1.S1_a_at	LOC_Os01g01870.1	1.788914	up	2.349517	up	1.256941	up	4.203082	up
Os.31883.1.A1_at	LOC_Os11g06010.1	3.362363	down	1.040926	down	1.400412	down	3.499973	down
OsAffx.23999.1.S1_x_at	LOC_Os01g71320.1	4.210348	up	1.158069	up	1.375516	up	4.875874	up
Os.7893.1.S1_at	LOC_Os09g37910.1	1.96585	down	2.201968	down	1.026753	down	4.328738	down
Os.57484.1.S1_x_at	LOC_Os09g37910	1.553284	down	2.596957	down	1.211649	up	4.033811	down
Os.28034.1.S1_at	LOC_Os08g37580.1	2.23679	down	1.187233	down	1.145204	up	2.65559	down
Os.2362.1.S1_a_at	LOC_Os02g35770.1	1.476618	down	1.110365	down	2.384479	up	1.639585	down

Os.4164.2.S1_a_at	LOC_Os03g03164.1	2.838154	up	2.576063	up	3.259348	down	7.311265	up
Os.37866.1.S1_at	LOC_Os03g12110.1	2.118644	up	1.356583	up	1.002884	down	2.874116	up
Os.46162.1.S1_at	LOC_Os01g07170	2.493357	down	1.111253	up	1.230846	down	2.243734	down
Os.40018.1.S1_at	LOC_Os05g45410.1	1.843028	up	2.638707	up	1.267727	up	4.863211	up
Os.35681.1.S1_at	LOC_Os01g53220.1	1.176137	up	2.728592	down	1.173744	up	2.319961	down
Os.52180.1.S1_at	LOC_Os03g04030.1	2.112722	down	1.503251	down	1.017325	down	3.175952	down
OsAffx.25245.1.S1_at	LOC_Os03g27110.1	1.664693	up	1.424516	down	3.689336	up	1.168603	up
Os.17325.1.S1_at	LOC_Os03g03200.2	1.625653	up	2.709215	up	1.067972	down	4.404244	up
OsAffx.28760.2.S1_at	LOC_Os07g35180.1	1.824345	up	2.28373	up	1.533528	up	4.166311	up
Os.18314.1.S1_at	LOC_Os09g20900.1	1.600325	up	1.20469	down	3.518874	up	1.328413	up
Os.12642.1.S1_at	LOC_Os08g42740.2	3.492732	up	1.080951	up	1.682101	up	3.775473	up
Os.14199.1.S1_at	LOC_Os05g34270.1	1.203931	up	2.537691	up	1.103599	down	3.055205	up
Os.50903.2.S1_x_at	LOC_Os09g08130.1	3.819665	up	1.837215	up	1.130916	down	7.017544	up
Os.50903.1.S1_at	LOC_Os09g08130.2	3.087118	up	1.904007	up	1.033398	down	5.877896	up
Os.38812.1.S1_at	LOC_Os01g42860.1	2.448273	up	8.113127	down	6.420624	up	3.313816	down
OsAffx.25798.1.S1_at	LOC_Os03g62750.1	3.081461	up	1.114477	down	1.222979	up	2.76494	up
Os.20700.1.S1_at	LOC_Os03g62750.1	2.466441	up	1.002071	down	1.176793	up	2.461344	up
Os.46160.2.S1_at	LOC_Os10g30790.2	1.067638	up	3.435324	up	1.249478	down	3.667682	up
Os.46160.2.S1_x_at	LOC_Os10g30790.2	1.069497	up	2.912623	up	1.228773	down	3.115042	up
OsAffx.20681.1.S1_at	LOC_Os06g10750.1	1.589452	down	1.000403	down	3.406255	down	1.590092	down
Os.9660.1.S1_at	LOC_Os09g23300.1	4.145318	down	13.55628	up	15.7131	down	3.270264	up
OsAffx.6372.1.S1_s_at	LOC_Os09g23300.1	2.038805	down	4.880853	up	4.363123	down	2.393977	up
Os.25449.1.S1_at	LOC_Os07g24230.1	1.224577	up	1.727674	down	5.424158	up	1.410834	down
Os.7756.2.S1_x_at	LOC_Os10g36500.2	2.571325	down	5.496622	up	5.786489	down	2.137662	up
Os.7756.1.S1_at	LOC_Os10g36500.1	2.90579	down	2.916613	up	4.277263	down	1.003725	up
Os.12979.3.S1_x_at	LOC_Os01g61720.1	1.529628	down	1.294888	up	3.234917	down	1.181282	down
Os.27797.1.A1_at	LOC_Os03g06570.1	1.183288	down	1.30201	down	3.412315	up	1.540652	down
Os.27581.1.A1_at	LOC_Os02g19640	1.249597	up	4.900516	up	2.141621	down	6.123668	up
Os.6863.1.S1_at	LOC_Os12g14440.1	1.337468	up	11.07103	up	4.39439	down	14.80714	up
OsAffx.2983.1.S1_x_at	LOC_Os02g46930.1	3.944061	up	1.089543	up	1.154415	up	4.297225	up
Os.5373.1.S1_at	LOC_Os02g46930.1	4.314814	up	1.370628	up	1.443678	up	5.914007	up
Os.33145.1.S1_at	LOC_Os07g36570.1	2.76842	up	1.118687	up	1.145333	up	3.096995	up
OsAffx.23899.1.S1_at	LOC_Os01g63220.3	3.343883	down	1.23436	down	1.322673	down	4.127554	down
Os.37822.3.A1_s_at	LOC_Os01g07370.1	1.675308	up	2.471888	up	1.075918	down	4.141172	up

Os.9615.1.S1_at	LOC_Os01g62490.1	1.204048	up	1.101364	up	4.150395	up	1.326094	up
OsAffx.26826.1.S1_at	LOC_Os05g11210.1	3.32506	down	3.09485	up	4.422081	down	1.074385	down
Os.49639.1.S1_at	LOC_Os06g21910.1	1.332342	up	11.91293	down	29.89466	up	8.94134	down
Os.27956.1.S1_at	LOC_Os03g06360.1	1.931017	up	1.826746	down	20.92934	up	1.05708	up
Os.12551.1.S1_s_at	LOC_Os05g46480.1	4.338625	up	16.2564	down	18.0396	up	3.746902	down
Os.20717.1.S1_at	LOC_Os01g61460.1	2.060925	up	1.984353	up	1.020655	down	4.089603	up
OsAffx.12547.1.S1_at	LOC_Os02g48210.1	1.074003	down	4.715024	up	2.191733	down	4.39014	up
OsAffx.24328.1.S1_at	LOC_Os02g19530.1	1.059386	up	2.117254	up	1.535836	up	2.242989	up
Os.49817.1.S1_at	LOC_Os04g15650.1	2.008503	down	2.265272	up	2.048318	down	1.127841	up
OsAffx.17977.1.S1_at	LOC_Os09g31420.1	2.358688	up	1.027351	down	1.126225	up	2.295894	up
Os.21710.1.S1_at	LOC_Os05g32660.1	1.66487	up	2.351129	up	1.056781	up	3.914324	up
Os.16897.1.S1_at	LOC_Os03g61540.1	1.772859	down	1.014005	up	4.143134	down	1.748372	down
Os.24995.1.S1_a_at	LOC_Os07g47250.1	1.070952	up	1.79183	down	3.626956	up	1.673118	down
Os.50126.1.S1_at	LOC_Os11g19340.1	4.728852	up	1.079196	down	1.275606	up	4.38183	up
Os.7665.1.S1_at	LOC_Os04g56240.1	1.004854	up	1.113493	down	2.356979	up	1.108114	down
Os.9355.1.S1_at	LOC_Os01g72490.1	2.130943	up	1.441285	up	1.089472	down	3.071296	up
OsAffx.3569.1.S1_x_at	LOC_Os03g50960.1	3.017306	up	2.494028	down	2.413866	up	1.209813	up
OsAffx.3569.1.S1_at	LOC_Os03g50960.1	3.150347	up	2.515712	down	2.545806	up	1.252269	up
Os.6274.1.S1_at	LOC_Os05g47730.1	2.472425	up	7.678695	down	14.43679	up	3.105734	down
Os.5927.1.S1_at	LOC_Os03g63540.1	3.580567	up	1.037289	down	1.260792	up	3.451853	up
Os.18996.1.S1_at	LOC_Os05g05450.1	2.753411	down	1.170107	down	1.363652	down	3.221785	down
Os.17416.1.S1_at	LOC_Os01g17214.1	2.66863	down	1.971684	down	1.120402	up	5.261694	down
OsAffx.22176.1.S1_x_at	LOC_Os01g17214.1	2.478483	down	1.459899	down	1.167021	down	3.618335	down
Os.8413.2.A1_a_at	LOC_Os04g28520.1	3.877507	up	1.089519	up	1.256713	down	4.224617	up
Os.17762.1.S1_at	LOC_Os06g29844.1	3.670885	down	3.061886	up	2.231576	down	1.198897	down
OsAffx.14324.1.S1_at	LOC_Os04g48290.1	1.788542	up	2.631223	up	1.071316	up	4.706055	up
OsAffx.32325.1.S1_at	LOC_Os04g16734.1	3.41098	down	4.598758	up	4.797165	down	1.348222	up
Os.46819.1.S1_at	LOC_Os10g29220.1	1.557271	up	2.131746	up	1.669049	up	3.319706	up
Os.14667.2.S1_at	LOC_Os05g38250.1	1.144779	up	2.042455	up	1.046957	up	2.33816	up
Os.15732.1.S1_s_at	LOC_Os03g29850.1	2.183363	down	2.991782	up	2.447511	down	1.370264	up
Os.28026.1.S1_at	LOC_Os05g39560.1	1.690769	down	1.724153	up	3.702639	down	1.019745	up
Os.27159.1.S1_at	LOC_Os06g37010.1	1.62321	up	2.659432	up	1.004381	up	4.316818	up
Os.12410.1.S1_a_at	LOC_Os01g74300.1	1.238081	down	1.4417	up	4.07061	down	1.164464	up
Os.12410.1.S1_x_at	LOC_Os01g74300.1	1.276194	down	1.84653	up	4.182207	down	1.446904	up

Os.12410.3.S1_s_at	LOC_Os01g74300.1	1.42277	down	1.963042	up	4.425994	down	1.379733	up
Os.12410.3.S1_x_at	LOC_Os01g74300.1	1.24811	down	1.341219	up	2.748553	down	1.0746	up
Os.25146.1.A1_at	LOC_Os03g10220	1.044165	up	2.76911	up	1.845764	up	2.891407	up
Os.14938.1.S1_at	LOC_Os05g01140.1	3.068714	up	4.674285	up	3.20661	down	14.34405	up
Os.5094.1.S2_at	LOC_Os05g31480.1	2.037387	down	1.292222	down	1.289859	up	2.632757	down
Os.45916.1.S1_s_at	LOC_Os01g14850.1	1.071237	down	2.353059	up	1.350336	up	2.196581	up
Os.27703.1.S1_a_at	LOC_Os09g13650.1	3.083721	up	19.45943	up	23.26575	down	60.00745	up
Os.27703.1.S1_s_at	LOC_Os09g13650.1	2.322572	up	17.29264	up	13.9145	down	40.1634	up
Os.11491.1.S1_at	LOC_Os03g19290.1	2.621756	up	17.01999	down	31.76919	up	6.491828	down
Os.34494.1.S1_at	LOC_Os05g02060.1	2.342667	down	4.432497	up	3.810647	down	1.892073	up
Os.8763.1.S1_at	LOC_Os11g05650.1	1.345021	up	1.050661	up	2.691558	up	1.413161	up
Os.1726.1.S1_at	LOC_Os01g18240.1	2.353093	up	1.784784	up	1.006509	up	4.199762	up
Os.52869.1.S1_at	LOC_Os01g62410.1	3.947863	down	1.073306	up	1.71107	down	3.678228	down
Os.3388.1.S1_x_at	LOC_Os04g43680.1	2.11464	up	1.496628	up	1.048321	up	3.164828	up
Os.7051.1.S1_at	LOC_Os02g49986.1	12.13425	up	6.464623	down	9.010881	up	1.877024	up
Os.3388.2.S1_a_at	LOC_Os04g43680.1	1.841608	up	2.405558	up	1.004368	up	4.430095	up
Os.49746.1.S1_at	LOC_Os01g74020.1	1.77672	down	2.598244	down	1.356676	down	4.616351	down
OsAffx.24769.1.S1_s_at	LOC_Os02g47744.1	1.576373	down	2.108565	down	1.30385	down	3.323884	down
Os.10333.1.S1_at	LOC_Os07g02800.2	1.201117	down	2.388151	down	1.364651	down	2.868449	down
Os.11638.1.S1_at	LOC_Os12g37970.1	1.072105	down	2.388975	down	1.189243	down	2.561232	down
Os.623.3.S1_x_at	LOC_Os01g09640.1	4.007706	up	2.296283	down	2.71669	up	1.745301	up
Os.23087.1.S1_at	LOC_Os05g10690.1	2.531071	up	2.524056	down	3.092072	up	1.002779	up
OsAffx.27267.1.S1_at	LOC_Os05g40960.1	1.205401	down	1.162172	up	4.948198	down	1.037196	down
Os.15138.1.S1_at	LOC_Os01g41900.1	1.574766	up	2.596653	up	1.383265	up	4.089119	up
OsAffx.11956.1.S1_at	LOC_Os02g09480.1	1.010624	down	2.336792	down	1.460523	down	2.361619	down
Os.5549.1.S1_at	LOC_Os07g12340.1	2.165257	down	4.457559	up	4.649281	down	2.058674	up
Os.10131.1.S1_a_at	LOC_Os05g51670.1	3.61788	up	2.09871	down	3.633362	up	1.723859	up
Os.44475.1.S1_x_at	LOC_Os07g37730.1	6.654109	up	1.724997	up	1.10353	up	11.47832	up
Os.47958.1.A1_x_at	LOC_Os01g61410.1	1.534961	up	2.091158	up	1.554285	up	3.209847	up
Os.8821.1.S1_at	LOC_Os01g66720.1	1.789776	up	1.063523	down	4.12308	up	1.682874	up
OsAffx.32221.1.A1_s_at	LOC_Os04g16732.1	1.020565	up	2.952147	down	1.161198	up	2.892659	down
Os.47363.1.A1_at	LOC_Os01g70080	1.820783	up	2.083502	up	1.036893	up	3.793605	up
Os.57563.1.S1_s_at	LOC_Os04g53160.1	1.118459	down	2.561802	up	1.15237	up	2.290475	up
Os.9311.1.S1_at	LOC_Os03g19420.2	1.113025	up	15.9468	up	1.736212	down	17.74918	up

Os.24786.1.S1_s_at	LOC_Os04g48870.1	1.972914	up	15.78855	up	3.90451	down	31.14945	up
OsAffx.26533.1.S1_at	LOC_Os04g48870.1	1.678589	up	14.57876	up	3.522701	down	24.47174	up
Os.45902.1.A1_x_at	LOC_Os06g28550,LOC_Os11g	1.143962	down	7.365033	up	1.609564	down	6.438182	up
Os.7235.1.S1_at	LOC_Os03g21030.1	3.480056	up	1.124625	up	1.264509	up	3.913757	up
Os.17090.1.S1_at	LOC_Os03g56580.1	2.073317	up	1.206766	up	1.130756	down	2.502009	up
Os.34471.1.S1_at	LOC_Os01g64310.1	1.441979	up	2.787719	up	1.207491	down	4.019833	up
OsAffx.12986.1.S1_at	LOC_Os03g22200.1	6.402714	up	2.123357	down	2.422899	up	3.015373	up
OsAffx.12986.1.S1_at	LOC_Os03g22200.1	6.402714	up	2.123357	down	2.422899	up	3.015373	up
Os.4974.1.S1_x_at	LOC_Os11g31190.1	2.349949	down	2.869224	up	3.785498	down	1.220973	up
Os.4974.1.S1_at	LOC_Os11g31190.1	2.369406	down	3.089984	up	3.219019	down	1.304118	up
Os.10401.1.S1_s_at	LOC_Os08g42350.1	1.449265	down	3.417646	up	1.306084	down	2.358192	up
Os.46711.1.S1_at	LOC_Os10g12400.1	3.797983	up	1.317326	up	1.284594	up	5.003182	up
Os.54933.1.S1_at	LOC_Os04g34490.1	3.402246	up	1.435219	up	1.164273	up	4.882968	up
Os.12092.1.S1_at	LOC_Os09g25810.2	1.017218	up	1.163975	down	2.340989	up	1.144274	down
Os.12191.1.S1_s_at	LOC_Os03g12510.1	1.053834	down	8.64106	down	2.940848	up	9.106237	down
Os.3426.1.S1_at	LOC_Os03g07880.1	1.001964	up	1.477854	down	3.323009	up	1.474958	down
Os.7298.2.S1_at	LOC_Os03g12450.1	2.833993	down	1.07208	up	1.357978	down	2.643454	down
Os.8375.1.S1_at	LOC_Os07g37100.1	1.624453	up	2.433667	up	1.111698	down	3.953379	up
Os.47730.2.S1_x_at	LOC_Os11g03230.1	3.061711	up	1.279609	up	1.437667	down	3.917794	up
Os.11908.1.S1_s_at	LOC_Os11g03230.1	2.780617	up	1.735303	up	1.582422	down	4.825213	up
Os.47730.1.S1_x_at	LOC_Os11g03230.2	2.818416	up	1.24136	up	1.196104	down	3.498668	up
Os.52527.1.S1_s_at	LOC_Os06g03560.1	3.15276	down	2.914857	up	4.124927	down	1.081617	down
OsAffx.13783.1.S1_at	LOC_Os04g11970.1	1.168992	up	2.541344	up	5.404311	down	2.970811	up
Os.53458.1.S1_at	LOC_Os09g17560.1	1.510757	down	2.912536	down	1.178603	up	4.400134	down
OsAffx.26358.1.S1_at	LOC_Os04g36850.1	2.082137	down	1.437272	down	1.373426	up	2.992597	down
Os.8593.1.S1_at	LOC_Os04g53930.1	1.153446	up	2.030444	up	1.030698	up	2.342007	up
Os.12701.1.S1_at	LOC_Os04g55650.1	1.904831	down	1.960354	up	4.116179	down	1.029149	up
Os.26441.1.S1_s_at	LOC_Os11g45710.5	1.470398	up	1.064034	up	3.618159	up	1.564552	up
Os.26537.1.S1_a_at	LOC_Os01g70520.1	3.792723	down	1.18597	down	1.115072	down	4.498057	down
Os.26537.1.S1_at	LOC_Os01g70520.1	3.52553	down	1.442686	down	1.077074	down	5.086235	down
Os.26537.2.S1_x_at	LOC_Os01g70520.1	2.91452	down	1.120129	down	1.406165	down	3.264637	down
OsAffx.15538.1.S1_at	LOC_Os06g21570.1	2.994871	down	2.208952	up	4.267804	down	1.355789	down
Os.16401.1.S1_at	LOC_Os03g21380.1	1.581673	up	2.501147	up	1.168374	up	3.955998	up
Os.50550.1.S1_at	LOC_Os06g05580.1	2.22264	down	1.109566	up	1.043119	down	2.003162	down

Os.11474.1.S1_at	LOC_Os02g51350.1	1.888647	up	2.631767	up	1.436286	up	4.970479	up
Os.7147.1.S1_at	LOC_Os07g06670.1	2.203374	down	1.115182	down	1.338966	up	2.457163	down
Os.5865.1.S1_at	LOC_Os02g33400.1	4.09713	down	1.022193	up	1.911769	down	4.008177	down
Os.52157.1.S1_x_at	LOC_Os11g34460.1	2.110785	down	2.087068	up	3.524918	down	1.011363	down
Os.17446.1.S1_at	LOC_Os04g48270.1	1.311882	down	2.047573	down	1.161436	down	2.686173	down
Os.29745.1.S2_at	LOC_Os07g12560.1	2.960764	down	1.508833	down	1.090401	up	4.467299	down
Os.52957.1.S1_at	LOC_Os07g12600.1	4.474104	down	1.233799	up	1.950101	down	3.626283	down
Os.15941.2.S1_x_at	LOC_Os01g65510.1	2.982864	up	1.385498	up	1.111879	up	4.132752	up
Os.23911.1.S1_at	LOC_Os09g30180.1	4.566416	down	1.116768	down	1.643773	down	5.099628	down
Os.27165.1.A1_at	LOC_Os09g32870.1	2.902516	down	2.004873	up	2.878954	down	1.447731	down
Os.46492.1.S1_at	LOC_Os10g04370.1	1.640253	up	1.822385	down	3.831198	up	1.111039	down
Os.52678.1.S1_at	LOC_Os04g41130.1	1.294716	up	2.750028	down	1.231692	down	2.124039	down
Os.55402.1.S1_at	LOC_Os11g43520.1	3.147687	down	1.95661	down	1.088308	up	6.158793	down
Os.11943.2.S1_at	LOC_Os08g45140	1.064208	down	1.408649	up	2.082226	up	1.32366	up
Os.9829.1.S1_at	LOC_Os05g48930.1	1.410822	up	2.40927	up	1.024806	down	3.399052	up
Os.38099.1.S1_at	LOC_Os03g61270.1	1.177009	up	5.188443	down	2.413447	up	4.40816	down
Os.24428.1.S1_at	LOC_Os07g48970	3.500539	down	1.061831	up	1.639463	down	3.296701	down
Os.52261.1.S1_at	LOC_Os04g47360.1	1.301478	up	6.407114	up	2.403393	down	8.338718	up
Os.13968.2.S1_a_at	LOC_Os05g04700.1	2.501199	up	2.418381	down	3.629444	up	1.034245	up
Os.53632.1.S1_at	LOC_Os05g13370.1	1.21557	up	1.771539	down	4.544385	up	1.457373	down
Os.6363.1.S1_at	LOC_Os10g38080.1	2.514768	down	1.294604	down	1.161627	down	3.255628	down
OsAffx.31856.1.S1_at	LOC_Os12g23980.1	1.209832	up	1.708296	down	4.292659	up	1.412011	down
Os.23262.1.A1_s_at	LOC_Os12g23980.1	1.01446	down	1.542451	down	3.347987	up	1.564756	down
Os.35642.2.S1_x_at	LOC_Os01g58280.1	2.298537	down	2.413608	up	3.129001	down	1.050063	up
Os.15516.1.S1_at	LOC_Os04g30240.1	3.984428	down	4.363255	up	3.000296	down	1.095077	up
Os.7017.1.S1_at	LOC_Os01g26280.1	2.238984	down	2.844185	up	3.007655	down	1.270302	up
Os.11469.1.S1_at	LOC_Os04g26870.1	2.291393	up	7.179496	up	4.698967	down	16.45104	up
Os.4666.1.S1_at	LOC_Os10g37330.1	2.079733	up	1.137417	up	1.22573	down	2.365524	up
Os.11837.1.S1_at	LOC_Os04g26920.2	3.158885	down	2.395188	up	4.362471	down	1.318846	down
Os.50961.1.S1_at	LOC_Os03g58890.1	1.125749	up	3.070543	up	1.227595	down	3.456661	up
Os.54307.1.S1_at	LOC_Os11g16410.1	3.164621	down	1.166816	up	1.566662	down	2.712186	down
OsAffx.12379.1.S1_at	LOC_Os02g36850.1	1.06898	up	2.098317	up	1.281661	up	2.243059	up
Os.15918.1.S1_x_at	LOC_Os03g63060.1	1.137294	up	1.551003	down	3.266998	up	1.363766	down
Os.18196.1.S1_at	LOC_Os07g42700.1	4.032504	down	1.4719	up	1.625935	down	2.739659	down

Os.49524.1.S1_at	LOC_Os11g39990.1	2.450054	up	1.392412	up	1.202421	down	3.411484	up
Os.7725.1.S1_at	LOC_Os05g02120	2.290476	down	1.326563	down	1.068399	down	3.038461	down
Os.32108.1.S1_s_at	LOC_Os01g21034.1	1.384576	down	3.186654	up	1.274519	down	2.301537	up
Os.51061.1.S1_at	LOC_Os11g29230.1	2.190589	down	1.480753	down	1.140752	up	3.243721	down
Os.36647.1.S1_at	LOC_Os03g37260	4.396884	down	1.259984	up	1.311856	down	3.489635	down
Os.46446.1.S1_at	LOC_Os10g21470.1	3.60652	down	1.268082	down	1.498702	down	4.573361	down
Os.54966.1.S1_at	LOC_Os06g47620.1	2.693266	up	1.959405	up	1.004091	up	5.2772	up
Os.34674.1.S1_at	LOC_Os01g01360.1	2.046496	down	1.475254	down	1.131858	up	3.019101	down
Os.46546.1.S1_at	LOC_Os10g22560.1	1.046714	up	2.844765	up	1.377612	down	2.977656	up
Os.9321.1.S1_a_at	LOC_Os01g65100.1	5.179151	down	1.229169	down	1.201443	up	6.36605	down
Os.18513.1.S1_at	LOC_Os05g32820.1	1.176043	up	2.106489	up	1.038345	down	2.477322	up
Os.15219.1.S1_at	LOC_Os06g11320.1	1.222969	up	1.307998	down	2.838551	up	1.069527	down
Os.10197.1.S1_at	LOC_Os11g24060.1	1.367937	up	1.496063	down	3.088738	up	1.093664	down
Os.9893.1.S1_at	LOC_Os03g13200.1	2.344924	up	1.300489	up	1.467895	down	3.049548	up
Os.11561.2.S1_a_at	LOC_Os03g22010.1	2.013164	up	1.482811	up	1.44401	down	2.985142	up
Os.7832.1.S1_at	LOC_Os03g02920.1	2.345512	up	1.505598	up	1.147723	up	3.531399	up
Os.27789.1.A1_at	LOC_Os06g20150.1	2.427756	down	2.174126	up	3.299707	down	1.116658	down
Os.17722.1.S1_at	LOC_Os01g22249.1	3.635587	down	2.483632	up	4.677089	down	1.463819	down
Os.47625.1.A1_s_at	LOC_Os05g04500.1	1.71188	up	34.95593	up	11.78206	down	59.84035	up
Os.36995.1.S1_at	LOC_Os07g02440.1	1.218495	up	1.270257	down	4.773978	up	1.04248	down
Os.15894.1.A1_a_at	LOC_Os01g73170.1	1.23342	down	1.99046	up	4.028204	down	1.613773	up
Os.32292.1.S1_at	LOC_Os01g36240.1	1.6152	up	1.312835	down	4.772993	up	1.230315	up
Os.8139.1.S1_at	LOC_Os09g29490.1	1.037227	down	2.838526	up	1.413624	down	2.736649	up
Os.11218.1.S1_at	LOC_Os07g44440	2.400224	down	2.062154	up	2.270465	down	1.16394	down
Os.13972.1.S1_at	LOC_Os07g44430	1.529364	up	1.350043	down	7.417645	up	1.132826	up
Os.4251.1.S1_at	LOC_Os02g09940.1	1.256227	up	3.670268	up	1.492122	down	4.61069	up
Os.49185.1.S1_at	LOC_Os04g45210.1	1.063302	up	1.016851	up	2.348399	up	1.08122	up
Os.9618.1.S1_at	LOC_Os04g59510.1	2.798914	down	1.217252	up	1.250891	down	2.299371	down
Os.140.1.A1_s_at	LOC_Os02g35600.1	2.353115	down	1.172308	up	1.023342	down	2.00725	down
Os.54501.1.S1_at	LOC_Os11g12650.1	3.370988	up	1.49283	up	1.221975	up	5.03231	up
Os.10930.1.S1_at	LOC_Os02g41670.1	2.600999	up	1.535078	up	1.012735	up	3.992735	up
Os.25687.1.S1_at	LOC_Os02g41680.1	1.250238	up	4.687602	up	2.231171	down	5.86062	up
Os.25687.1.S1_x_at	LOC_Os02g41680.1	1.100536	up	5.304674	up	2.070208	down	5.837984	up
Os.37893.1.S1_at	LOC_Os04g43760.1	1.312565	up	3.066838	up	1.05008	down	4.025424	up

OsAffx.17942.1.S1_at	LOC_Os09g28160.1	3.04386	up	1.698157	up	1.055543	down	5.168952	up
Os.52435.1.S1_at	LOC_Os06g29790.1	3.530764	down	2.275328	up	3.625043	down	1.55176	down
Os.14366.1.S1_at	LOC_Os08g37840.1	1.27355	up	2.849116	up	1.086747	down	3.628491	up
Os.17022.1.S1_at	LOC_Os02g18840.1	3.037207	down	1.183703	down	1.481713	down	3.595153	down
Os.49109.1.S2_at	LOC_Os08g38850.1	2.246754	down	2.223626	up	3.064144	down	1.010401	down
Os.46823.1.S1_at	LOC_Os10g41480.1	3.024282	down	2.236425	up	2.375907	down	1.352284	down
Os.7075.1.S1_at	LOC_Os03g27230.1	1.439538	up	2.721908	up	1.305209	down	3.918289	up
Os.11118.1.S1_at	LOC_Os06g45710.1	2.146795	up	4.3072	down	6.6019	up	2.00634	down
Os.50455.1.S1_at	LOC_Os06g40170.1	2.959879	up	5.086209	up	4.294294	down	15.05456	up
Os.29866.1.S1_at	LOC_Os01g52530.1	3.378922	up	1.173948	up	1.483237	up	3.966679	up
Os.7457.1.S1_a_at	LOC_Os04g42520.1	1.156104	down	2.306609	down	1.148021	up	2.66668	down
OsAffx.32257.1.A1_at	LOC_Os01g58049.1	1.001941	down	2.197605	down	1.26069	down	2.20187	down
OsAffx.1590.1.S1_x_at	LOC_Os06g46436.1	1.249391	down	2.111219	down	1.2565	down	2.637738	down
Os.49840.1.S1_at	LOC_Os02g05980.1	3.504456	down	2.028517	up	2.23568	down	1.727595	down
Os.24551.4.S1_at	LOC_Os02g06210.1	3.742085	down	2.33659	up	3.295818	down	1.601516	down
Os.50897.1.S1_at	LOC_Os03g57560.1	3.055042	down	1.049818	down	1.22322	down	3.207238	down
Os.49381.1.S1_at	LOC_Os08g26820.1	4.880503	down	4.020639	up	4.904074	down	1.213862	down
Os.30998.1.S1_at	LOC_Os01g62060.1	1.523729	up	2.779407	up	1.086981	down	4.235063	up
Os.27232.1.S1_at	LOC_Os01g68650.1	2.170471	up	2.56545	down	2.395549	up	1.181979	down
Os.7743.1.S1_at	LOC_Os06g11490.1	1.711769	up	3.043233	up	1.079138	up	5.20931	up
Os.24863.1.A1_at	LOC_Os09g16380	1.896557	up	2.205661	up	1.052718	down	4.183161	up
Os.52493.1.A1_s_at	LOC_Os03g22210.1	1.256127	down	1.675995	up	3.789932	down	1.334257	up
Os.625.1.S1_at	LOC_Os01g09670.1	3.466174	up	1.317273	up	1.370805	up	4.565896	up
OsAffx.23247.1.S1_x_at	LOC_Os01g14630.1	3.300857	up	1.889413	up	1.861601	down	6.236683	up
OsAffx.23247.1.S1_at	LOC_Os01g14630.1	2.486881	up	1.927792	up	1.704662	down	4.79419	up
Os.46093.1.S1_at	LOC_Os10g33900.1	5.846602	down	4.338939	up	5.90204	down	1.347473	down
Os.11789.1.S1_at	LOC_Os03g01880.1	1.174398	down	1.38708	up	2.969362	down	1.181098	up
Os.8081.1.A1_at	LOC_Os07g36390.1	2.523667	down	1.088281	up	1.207406	down	2.318949	down
Os.49173.1.S1_at	LOC_Os02g16650.1	3.510408	down	1.039451	up	1.25996	down	3.377175	down
Os.17834.1.S1_at	LOC_Os02g39820.1	2.443498	down	1.082393	up	1.16332	down	2.257497	down
Os.10162.1.S1_at	LOC_Os01g28790.1	2.250928	down	2.306609	up	2.444082	down	1.024737	up
Os.778.1.S1_at	LOC_Os01g02700.1	3.718182	down	3.076568	up	2.179192	down	1.208549	down
Os.11183.1.S1_at	LOC_Os06g18000.1	1.125991	down	4.078871	up	1.682172	down	3.622473	up
Os.11183.1.S1_s_at	LOC_Os06g18000.1	1.333628	down	5.348746	up	1.921642	down	4.010674	up

Os.21870.1.S1_at	LOC_Os02g45750.1	1.098887	down	3.383156	up	1.564385	down	3.078712	up
Os.14313.1.S1_s_at	LOC_Os01g61620.1	1.43591	up	2.904314	up	1.347783	down	4.170332	up
Os.14313.2.S1_s_at	LOC_Os01g61620.1	1.641476	up	4.053012	up	1.044134	up	6.652921	up
Os.27484.1.S1_at	LOC_Os02g06930.1	1.496602	up	2.615583	up	1.089109	up	3.914487	up
Os.34372.1.S1_at	LOC_Os06g48300.1	7.891869	up	18.48453	down	31.28925	up	2.342225	down
Os.39552.1.A1_s_at	LOC_Os06g48300.1	7.037013	up	15.38511	down	27.72498	up	2.186313	down
Os.7447.1.S1_a_at	LOC_Os03g25600.1	3.0392	down	1.202711	up	1.347001	down	2.526959	down
Os.9705.1.S1_at	LOC_Os02g27220.1	2.316387	up	1.419344	up	1.034824	up	3.287749	up
OsAffx.19579.1.S1_at	LOC_Os12g09640.1	1.51203	up	2.234711	up	1.01056	up	3.37895	up
Os.38299.1.S1_at	LOC_Os07g02330.1	1.080362	up	2.391651	up	1.202543	up	2.583848	up
Os.53707.1.S1_x_at	LOC_Os01g65169.1	2.852177	down	2.376975	up	3.992315	down	1.199919	down
OsAffx.29248.3.S1_s_at	LOC_Os08g17294.1	1.130112	down	1.23034	down	2.292538	down	1.390422	down
Os.27684.1.S2_at	LOC_Os01g62650.1	2.51911	down	1.595583	down	1.595323	up	4.01945	down
Os.14949.1.S1_at	LOC_Os04g12480.1	3.66757	down	1.140871	down	1.09976	down	4.184224	down
Os.52717.1.S1_at	LOC_Os12g30520.1	2.840561	down	3.119262	up	3.28401	down	1.098115	up
Os.4638.1.S1_at	LOC_Os11g26880.1	1.283831	down	1.418781	down	2.980766	up	1.821476	down
OsAffx.14406.1.S1_at	LOC_Os04g54090.1	1.18576	up	1.343773	up	2.643554	up	1.593393	up
Os.53724.1.S1_at	LOC_Os11g26340.1	1.549859	up	1.116669	up	3.198237	up	1.73068	up
Os.22341.1.S1_at	LOC_Os01g25540.1	1.980053	up	2.317436	up	1.095367	up	4.588646	up
Os.9217.1.S1_at	LOC_Os05g48980.1	3.238381	up	1.196502	up	1.558606	up	3.874729	up
Os.57548.1.S1_at	LOC_Os12g05590.1	2.410331	down	1.802606	down	1.063665	down	4.344876	down
OsAffx.19285.1.S1_at	LOC_Os11g40810.1	2.095336	up	1.677308	up	1.003515	up	3.514525	up
Os.26411.2.A1_x_at	LOC_Os08g28710.1	4.851004	up	1.101038	down	1.168617	up	4.405845	up
Os.49819.1.S1_at	LOC_Os11g36200.1	2.182592	down	3.454398	up	3.97113	down	1.582705	up
Os.49827.1.S1_at	LOC_Os11g10310.1	2.812084	down	2.477443	up	3.755894	down	1.135075	down
Os.26870.2.A1_x_at	LOC_Os09g17630.1	2.380357	up	1.398815	up	1.177374	up	3.32968	up
OsAffx.6331.1.S1_at	LOC_Os09g19380.1	2.725521	down	2.574	up	3.462552	down	1.058866	down
Os.52761.1.A1_at	LOC_Os04g39930.1	3.643747	down	1.249191	up	1.804434	down	2.916885	down
Os.27955.1.S1_at	LOC_Os03g63270.1	2.512766	up	1.508616	up	1.017372	up	3.7908	up
Os.27955.2.S1_x_at	LOC_Os03g63270.1	2.346804	up	1.001175	up	1.060239	up	2.349561	up
Os.16903.1.A1_at	LOC_Os05g06920.1	1.533174	up	2.936003	up	1.143202	down	4.501404	up
Os.46647.1.S1_at	LOC_Os10g36000.3	1.165336	down	2.079044	down	1.029344	up	2.422784	down
Os.5404.3.S1_a_at	LOC_Os01g61880.1	1.447135	down	1.69075	up	3.940643	down	1.168343	up
Os.9708.1.S1_at	LOC_Os06g35630.1	1.017912	down	2.192273	up	1.337837	up	2.153696	up

Os.7487.1.S1_at	LOC_Os05g24770.1	1.098447	up	6.451502	down	1.287167	up	5.873294	down
Os.4281.1.S1_x_at	LOC_Os06g03830.1	1.826062	down	3.358202	down	1.036653	up	6.132286	down
Os.6767.1.S1_at	LOC_Os08g16830.1	2.283651	down	2.158743	up	2.890235	down	1.057861	down
Os.10497.1.S1_s_at	LOC_Os07g23640.1	4.516145	up	1.970624	down	1.485828	up	2.291733	up
Os.40007.1.S1_x_at	LOC_Os12g12080.2	4.81833	up	2.590121	down	3.505571	up	1.860272	up
Os.19740.1.S2_at	LOC_Os03g02874.1	4.618869	up	2.325705	down	3.765994	up	1.986008	up
Os.25409.1.S1_x_at	LOC_Os06g45184.1	2.262508	up	3.260824	down	2.089474	up	1.441243	down
Os.39995.1.S1_x_at	LOC_Os01g37200.1	1.325309	up	2.881007	down	1.287957	up	2.173839	down
Os.40001.1.A1_at	LOC_Os12g12080.1	1.676917	up	8.65722	down	1.676685	up	5.162581	down
Os.56875.1.S1_at	LOC_Os08g34390.1	2.602746	up	10.26002	down	17.24599	up	3.941999	down
Os.30032.1.S1_at	LOC_Os01g03180.1	6.228989	down	2.084614	up	2.456193	down	2.988077	down
Os.30032.1.S1_at	LOC_Os01g03180.1	6.228989	down	2.084614	up	2.456193	down	2.988077	down
Os.5236.1.S1_at	LOC_Os01g04880.1	2.745411	down	1.098084	up	1.091425	down	2.500183	down
Os.14173.1.S1_at	LOC_Os04g50050.1	2.53116	down	1.163462	down	1.252891	down	2.94491	down
OsAffx.32166.1548.S1_s	LOC_Os01g20740.1	2.204918	down	2.358786	up	3.352027	down	1.069784	up
Os.46107.1.S2_a_at	LOC_Os02g32250.1	3.83656	down	2.389824	up	3.731801	down	1.605373	down
Os.6654.1.S1_at	LOC_Os11g04300	1.641988	up	18.51346	up	6.323765	down	30.39889	up
Os.27592.1.A1_at	LOC_Os11g42490.1	1.992968	up	1.207805	down	4.675945	up	1.650075	up
OsAffx.22999.1.S1_at	LOC_Os07g32710.1	1.702798	up	2.149322	up	1.059339	up	3.659861	up
Os.51711.1.S1_at	LOC_Os01g29507	1.758887	up	1.233346	down	4.092361	up	1.426109	up
OsAffx.28932.1.S1_at	LOC_Os07g46520.1	1.524425	up	1.354167	down	3.275353	up	1.12573	up
Os.45924.1.S1_x_at	LOC_Os07g46520.1	1.82054	up	1.360163	down	3.707088	up	1.338472	up
Os.7335.1.S1_at	LOC_Os02g36340.1	1.712927	up	2.287251	up	1.42117	up	3.917893	up
Os.35921.2.S1_x_at	LOC_Os01g60730.1	2.40411	up	1.114765	up	1.261627	down	2.680017	up
Os.35921.1.S1_at	LOC_Os01g60730.1	2.18234	up	1.881404	up	1.006294	up	4.105863	up
Os.4788.1.S1_at	LOC_Os12g05370.1	2.240179	down	1.15201	down	1.077403	down	2.580708	down
Os.30433.1.S1_at	LOC_Os01g55110.1	2.599167	up	2.10667	down	2.520166	up	1.23378	up
Os.48002.1.A1_x_at	LOC_Os01g48720.1	3.044891	down	1.160571	down	1.218693	down	3.533811	down
Os.14271.1.S1_at	LOC_Os07g43810.1	1.695268	down	1.155529	up	3.393169	down	1.467092	down
Os.22590.1.A1_at	LOC_Os03g17760.1	1.947549	up	2.626884	up	1.007806	up	5.115984	up
Os.6656.1.S1_at	LOC_Os02g18410.1	1.506212	up	2.0867	up	1.024549	down	3.143011	up
OsAffx.27612.1.S1_at	LOC_Os06g13470.1	19.74499	up	1.404842	up	1.1502	up	27.73859	up
Os.11831.1.S1_at	LOC_Os06g13560.1	1.188451	up	3.703318	down	1.510208	up	3.116087	down
Os.2436.1.S1_at	LOC_Os11g10290.1	1.157955	up	3.512662	up	1.440256	down	4.067504	up

Os.9199.1.S1_at	LOC_Os06g50330.1	1.231678	up	2.222691	up	1.01632	up	2.737638	up
Os.51809.1.S1_at	LOC_Os11g06780.1	1.138441	up	3.344278	up	1.369858	down	3.807265	up
Os.44598.1.S1_x_at	LOC_Os07g46930.1	2.30711	up	1.530534	up	1.57812	down	3.53111	up
Os.49410.1.A1_at	LOC_Os04g33240.1	2.589833	up	1.130879	up	1.027034	up	2.928788	up
Os.40030.1.S1_s_at	LOC_Os05g16420.1	1.284211	down	2.024633	down	1.300452	down	2.600056	down
OsAffx.6495.1.S1_s_at	LOC_Os09g36830.1	3.435303	down	1.057383	down	1.581111	down	3.632431	down
Os.21394.1.S1_at	LOC_Os02g24080.1	1.184548	up	2.208488	up	1.101862	down	2.61606	up
Os.38169.1.S1_at	LOC_Os02g43110.1	1.958952	up	2.121548	up	1.101723	up	4.156011	up
Os.4530.1.S1_at	LOC_Os09g31482.1	4.547062	up	2.324195	down	4.5872	up	1.956403	up
Os.6085.1.S1_at	LOC_Os05g46760.1	2.399315	up	1.355529	up	1.079622	up	3.252342	up
Os.32366.1.S1_at	LOC_Os01g50420.1	1.270758	down	11.29911	up	2.785595	down	8.891633	up
Os.9515.1.S1_x_at	LOC_Os07g08750.1	3.008026	down	1.049483	down	1.575773	up	3.156873	down
Os.9515.1.S1_at	LOC_Os07g08750.1	2.303763	down	1.237619	down	1.658765	up	2.851181	down
Os.9515.3.S1_x_at	LOC_Os07g08750.1	2.033422	down	1.534908	down	1.087679	up	3.121116	down
OsAffx.3180.1.S1_at	LOC_Os03g09940.1	2.644215	down	3.0061	up	4.607584	down	1.136859	up
Os.52260.1.S1_at	LOC_Os03g57310.1	1.290896	up	2.199649	up	1.040192	up	2.839517	up
Os.9681.1.S1_at	LOC_Os11g38010.1	2.08955	up	1.927104	up	1.439274	down	4.02678	up
Os.57103.1.S1_at	LOC_Os02g02930.1	3.866763	up	7.951527	up	4.17771	down	30.74668	up
Os.51227.1.S1_s_at	LOC_Os04g27790.1	1.343985	down	1.98686	up	7.18301	down	1.478335	up
Os.26820.1.A1_at	LOC_Os08g07080.1	1.279114	up	8.325118	up	4.002504	down	10.64878	up
Os.51227.1.S1_x_at	LOC_Os04g27790.1	1.17633	down	1.724534	up	5.086228	down	1.466029	up
OsAffx.29395.1.S1_at	LOC_Os08g28214.1	2.347279	down	1.305825	down	1.005022	down	3.065136	down
Os.52379.1.S1_s_at	LOC_Os02g10940.1	1.005565	up	2.507641	up	1.223761	down	2.521594	up
OsAffx.19428.1.S1_s_at	LOC_Os11g47680.1	3.552671	down	2.121246	up	2.54626	down	1.674804	down
Os.12761.1.S1_at	LOC_Os03g46060.1	1.105589	down	3.658999	down	1.654183	up	4.04535	down
Os.18490.1.S1_x_at	LOC_Os03g47610.2	3.165248	down	2.97635	up	5.945184	down	1.063466	down
Os.32267.1.S1_at	LOC_Os01g56330.1	1.58904	up	2.052436	up	1.063753	up	3.261402	up
Os.7632.1.S1_at	LOC_Os05g41370.1	1.851875	up	2.391727	up	1.025623	down	4.42918	up
Os.23264.1.A1_at	LOC_Os10g04720.1	5.71137	down	3.858335	up	6.722137	down	1.480268	down
Os.49818.1.S1_s_at	LOC_Os07g35310.1	3.276719	down	3.139376	up	5.703347	down	1.043749	down
Os.27431.1.A1_at	LOC_Os07g35310.1	2.770702	down	2.87046	up	3.566907	down	1.036005	up
OsAffx.10965.1.S1_at	LOC_Os01g08460	1.141558	up	1.204672	up	2.498814	up	1.375203	up
OsAffx.14364.1.S1_s_at	LOC_Os04g51320.1	3.193595	up	1.177027	down	1.40706	up	2.713272	up
OsAffx.11931.1.S1_at	LOC_Os02g07800.1	3.293891	down	1.124963	down	1.148182	down	3.705506	down

Os.17334.1.S1_s_at	LOC_Os04g38780	1.095874	up	1.503318	up	2.421731	up	1.647448	up
Os.10765.1.S1_at	LOC_Os11g07960.1	4.122187	down	2.543847	up	4.877424	down	1.620454	down
Os.19547.1.S1_at	LOC_Os08g02030.1	1.268181	down	2.331422	down	1.079077	down	2.956666	down
Os.51085.1.S1_at	LOC_Os04g47420.1	2.380879	down	2.060761	up	2.88106	down	1.15534	down
OsAffx.3504.1.S1_at	LOC_Os03g43720.6	2.200718	up	2.683221	down	2.399245	up	1.219248	down
Os.17497.1.S1_a_at	LOC_Os01g63290.1	2.309004	down	1.16896	down	1.002974	down	2.699134	down
Os.51696.1.S1_at	LOC_Os02g43620.1	1.236846	up	1.428818	down	3.649183	up	1.155211	down
OsAffx.4763.1.S1_at	LOC_Os06g08170.1	1.381771	up	2.007805	up	1.094531	up	2.774327	up
Os.4700.1.S1_at	LOC_Os07g47100.1	2.724446	up	2.454105	down	2.239394	up	1.110159	up
Os.48856.1.S1_at	LOC_Os08g04110.1	2.940618	up	1.346656	up	1.010675	down	3.960002	up
Os.35365.1.S1_at	LOC_Os06g30950.1	1.777885	up	5.236086	up	2.010635	down	9.309159	up
Os.47871.1.S1_at	LOC_Os06g51440.1	1.089115	up	5.6376	down	15.75423	up	5.176315	down
OsAffx.2070.2.S1_at	LOC_Os01g23850.1	1.204118	up	2.873614	up	1.06089	down	3.460169	up
Os.27497.1.S1_at	LOC_Os12g12390	1.419497	down	3.846331	down	1.124378	down	5.459855	down
Os.53320.1.S1_s_at	LOC_Os09g30270.1	1.60216	down	2.519348	down	1.065399	down	4.036398	down
Os.53320.2.S1_s_at	LOC_Os09g30270.1	1.494665	down	2.457534	down	1.110897	down	3.67319	down
Os.48053.1.A1_at	LOC_Os07g04860	5.1727	up	103.8992	down	143.3569	up	20.08607	down
Os.51849.1.S1_at	LOC_Os02g18370.1	3.019635	down	1.065477	up	1.391114	down	2.83407	down
Os.17140.1.S1_x_at	LOC_Os04g41220.1	2.338308	down	1.092067	up	1.088816	down	2.141175	down
Os.51810.1.S1_at	LOC_Os03g50900.1	3.65318	down	1.111641	up	1.774497	down	3.286295	down
Os.26884.1.S1_a_at	LOC_Os01g67810.1	2.325839	down	3.502098	up	2.517736	down	1.505735	up
Os.28110.3.S1_at	LOC_Os04g55710.1	1.490941	up	2.819032	up	1.043522	down	4.20301	up
Os.38110.1.S1_at	LOC_Os10g37660.1	3.925182	up	2.80631	down	2.194691	up	1.398698	up
Os.27235.1.S1_at	LOC_Os01g35170.1	3.608403	down	1.110864	down	1.769943	down	4.008444	down
Os.53243.1.S1_x_at	LOC_Os04g58690.1	2.007716	down	1.313969	down	1.009114	up	2.638076	down
Os.19406.1.S1_at	LOC_Os06g08440.1	2.483178	down	1.33659	down	1.103512	down	3.318991	down
Os.49855.1.S1_at	LOC_Os02g57560.1	2.747385	down	2.097523	up	2.290644	down	1.309823	down
Os.322.1.S1_at	LOC_Os06g30970	1.229803	up	2.715713	up	1.136236	down	3.339791	up
Os.8251.1.S1_at	LOC_Os03g13010.1	3.268484	down	1.329942	up	1.249649	down	2.457614	down
OsAffx.4833.1.S1_x_at	LOC_Os06g13870.1	1.097655	down	2.996729	up	1.436007	down	2.73012	up
Os.8045.1.S1_at	LOC_Os01g08440.1	3.110933	down	3.079837	up	2.864766	down	1.010097	down
Os.17160.1.S1_at	LOC_Os04g01780.1	2.464457	down	1.119401	down	1.090423	down	2.758715	down
Os.27290.1.A1_at	LOC_Os06g07600.1	2.576738	down	1.550502	down	1.013717	up	3.995238	down
Os.5338.1.S1_at	LOC_Os10g30150.1	1.766747	up	1.383659	down	7.837902	up	1.276866	up

Os.11995.1.S1_at	LOC_Os02g07150.3	2.383482	up	2.647599	down	2.571054	up	1.110811	down
Os.49796.1.S1_at	LOC_Os04g51030.1	2.453172	down	2.754627	up	4.684796	down	1.122884	up
Os.51744.1.A1_at	LOC_Os08g01680.1	2.700263	up	1.81959	up	1.490924	down	4.913371	up
Os.24954.1.A1_s_at	LOC_Os03g02110.1	3.435202	down	1.084897	up	1.209914	down	3.166386	down
Os.7232.1.S1_at	LOC_Os05g13520.1	2.144423	down	1.101954	down	1.0648	down	2.363054	down
Os.52820.1.S1_at	LOC_Os11g22350.1	1.231407	up	1.354243	down	4.356844	up	1.099753	down
Os.9913.1.S1_at	LOC_Os04g54230.1	2.205543	up	2.617968	down	2.950969	up	1.186995	down
Os.30512.1.S1_at	LOC_Os01g43650.1	4.070257	up	1.084119	up	1.374271	up	4.412642	up
Os.50015.1.S1_at	LOC_Os06g44010.1	1.332457	up	20.24944	up	2.319233	down	26.98152	up
OsAffx.30783.1.S1_at	LOC_Os11g02530.1	2.396696	up	1.456067	up	1.114149	down	3.48975	up
Os.8961.1.S1_s_at	LOC_Os05g46020.1	1.897929	up	3.046183	up	1.025289	up	5.781439	up
Os.12032.1.S1_at	LOC_Os02g08440.1	1.254187	up	3.506797	up	1.18282	down	4.398179	up
Os.25606.1.S1_at	LOC_Os09g25060.1	1.341911	up	2.412521	up	1.037385	down	3.237389	up
Os.30657.1.S1_at	LOC_Os01g40260.1	3.888007	up	3.083287	down	5.188138	up	1.260994	up
Os.37620.1.S1_at	LOC_Os01g55820.1	3.626782	up	1.235527	up	1.389083	up	4.480987	up
Os.9015.1.S1_at	LOC_Os01g73980.1	3.08419	up	3.269404	up	2.921857	down	10.08346	up
Os.6873.1.S1_at	LOC_Os08g34010.1	3.236173	down	1.023248	up	1.612629	down	3.162647	down
Os.9923.1.S1_s_at	LOC_Os03g08330.1	1.220013	up	11.18024	up	1.478796	down	13.64004	up
Os.46849.1.S1_at	LOC_Os10g25230.1	1.048229	up	20.80446	up	1.701111	down	21.80784	up
Os.29084.1.S1_at	LOC_Os01g53650.1	5.086082	up	2.767321	down	2.707605	up	1.837908	up
Os.11534.1.S1_at	LOC_Os05g10670.1	8.079985	up	5.937354	down	11.25619	up	1.360873	up
Os.31975.1.S1_x_at	LOC_Os05g10670.1	6.262105	up	5.015062	down	7.28505	up	1.24866	up
Os.31975.1.S1_at	LOC_Os05g10670.1	6.324755	up	5.022676	down	6.729147	up	1.25924	up
Os.38796.1.S1_s_at	LOC_Os02g10920.1	2.111293	up	1.803236	up	1.0011	up	3.807158	up
Os.23770.1.A1_s_at	LOC_Os01g49280.1	1.599026	up	3.303315	down	8.149099	up	2.06583	down
Os.18434.1.S1_at	LOC_Os02g40664.1	1.727039	up	2.104657	up	1.337881	up	3.634824	up
Os.27330.1.A1_at	LOC_Os04g49160.1	2.060473	up	1.48303	up	1.782308	down	3.055744	up
Os.14848.1.S1_at	LOC_Os06g48040.1	2.061173	up	1.9547	up	1.189166	down	4.028975	up
Os.18247.1.S1_a_at	LOC_Os03g15000.1	2.339113	up	1.74013	up	1.0961	up	4.07036	up
Os.1438.1.S1_at	LOC_Os01g06590.1	2.492919	up	1.356015	up	1.23873	up	3.380437	up
Os.52944.1.A1_at	LOC_Os06g09310.1	2.363785	down	3.745804	up	2.693814	down	1.584663	up
Os.51359.1.S1_at	LOC_Os07g48680.1	1.415229	down	2.069536	down	1.140649	down	2.928867	down
Os.33605.2.S1_x_at	LOC_Os01g50750.1	1.056645	up	2.297006	up	1.137694	down	2.42712	up
OsAffx.28216.1.S1_at	LOC_Os06g12560.1	2.650364	up	1.548316	up	1.077069	up	4.103602	up

Os.14686.1.S1_at	LOC_Os04g50120.1	3.983708	down	1.535722	up	1.965382	down	2.594029	down
Os.12710.1.S1_at	LOC_Os07g42370.1	1.566552	up	2.540952	up	1.128351	down	3.980533	up
Os.53413.1.S1_at	N/A	2.55981	up	1.130176	up	1.134961	down	2.893036	up
Os.9324.1.S1_at	N/A	3.333135	down	1.037752	down	1.378929	down	3.458968	down
Os.50841.1.S1_at	N/A	3.197549	down	1.074853	down	1.446653	down	3.436895	down
Os.23097.1.A1_at	N/A	2.278451	down	1.169254	down	1.095927	down	2.664088	down
OsAffx.21798.1.S1_at	N/A	2.133359	up	2.553181	down	2.462082	up	1.196789	down
Os.28110.4.S1_x_at	N/A	2.018204	down	4.368647	up	6.580046	down	2.164621	up
Os.51491.1.S1_at	N/A	2.148562	up	28.12385	down	23.67235	up	13.08962	down
Os.12430.1.S1_at	NA	4.053709	up	30.68101	down	29.7293	up	7.568626	down
Os.57327.1.S1_at	N/A	1.344277	up	1.035178	up	3.052708	up	1.391566	up
Os.50179.1.S1_at	N/A	1.239841	up	1.674829	down	3.72187	up	1.350841	down
Os.11147.1.S1_at	N/A	1.196572	up	1.102255	up	2.573804	up	1.318927	up
Os.46758.1.S1_at	N/A	1.249687	up	1.193611	down	2.948285	up	1.04698	up
Os.48873.1.S1_at	N/A	1.205787	up	1.152397	down	3.125876	up	1.04633	up
Os.9152.1.S1_at	N/A	1.384333	up	1.415032	down	2.94728	up	1.022176	down
Os.5357.1.S1_at	Not Valid	1.162061	up	1.160387	down	2.366095	up	1.001443	up
AFFX-r2-Bs-dap-5_at	N/A	1.557265	down	1.066882	up	3.245458	down	1.459641	down
OsAffx.15233.1.S1_at	N/A	1.766555	down	2.568367	down	1.275917	down	4.537161	down
OsAffx.32310.1.A1_at	N/A	1.024412	down	6.462949	down	1.287679	down	6.62072	down
OsAffx.32309.1.A1_at	N/A	1.020279	down	2.577191	down	1.262216	down	2.629453	down
OsAffx.8351.1.S1_at	N/A	1.130353	up	2.577133	down	1.183508	up	2.279936	down
Os.45902.1.A1_at	N/A	1.007417	down	7.167253	up	1.840779	down	7.114486	up
Os.54417.1.S1_at	N/A	1.430145	up	2.170882	up	1.244784	up	3.104676	up
Os.54317.1.S1_at	N/A	1.181578	down	2.501426	up	1.069394	down	2.117022	up
Os.15809.1.S1_at	N/A	1.074526	down	2.11694	down	4.371525	up	2.274707	down
Os.10416.1.S1_at	NA	1.418837	down	12.83815	up	3.056286	down	9.048362	up

[MS] vs [NS	Regulation	[NS] vs [NC	Regulation	Putative Function
3.136287	up	1.303295	up	12-oxophytodienoate reductase, putative, expressed
2.083965	up	1.834603	up	2-aminoethanethiol dioxygenase, putative, expressed
16.73664	down	2.550377	up	AAA-type ATPase family protein, putative, expressed
1.397867	down	1.684359	up	ABC transporter, ATP-binding protein, putative, expressed
1.092823	down	3.342736	down	abscisic stress-ripening, putative, expressed
1.023395	down	1.162591	down	ACT domain containing protein, expressed
1.185036	down	1.048961	up	ACT domain containing protein, putative, expressed
1.309552	down	1.601063	up	actin-depolymerizing factor, putative, expressed
2.080363	up	1.951546	up	adenylate kinase, putative, expressed
2.17951	down	1.578764	down	AGC_PKA/PKG_like.1 - ACG kinases include homologs to PKA, PKG and PKC, expressed
1.011718	up	2.076229	up	alliin lyase precursor, putative, expressed
2.573989	up	1.529611	up	alpha-amylase precursor, putative, expressed
1.226291	down	2.789417	up	alpha-DOX2, putative, expressed
1.459061	down	1.099128	up	aluminum-activated malate transporter, putative, expressed
2.169452	up	1.375821	up	amine oxidase, flavin-containing, domain containing protein, expressed
3.629605	up	1.770011	up	amino acid transporter, putative, expressed
2.566284	down	1.120836	down	amino acid transporter, putative, expressed
2.17607	down	2.720106	up	aminomethyltransferase, putative, expressed
2.199799	down	1.079471	down	aminotransferase domain containing protein, putative, expressed
2.604199	up	2.010999	up	AMP-binding domain containing protein, expressed
1.386831	down	1.246588	up	anaphase-promoting complex subunit 11, putative, expressed
3.074915	down	3.231424	up	ankyrin repeat domain-containing protein 44, putative, expressed
1.844731	up	1.372996	down	ankyrin repeat family protein, putative, expressed
1.197536	up	1.363649	down	ankyrin, putative, expressed
6.193117	up	1.047774	down	annexin, putative, expressed
1.229449	down	1.34519	up	annexin, putative, expressed
2.16039	up	1.596041	up	ANTH, putative, expressed
2.509265	up	2.271736	up	anthocyanidin 3-O-glucosyltransferase, putative, expressed
1.277633	up	1.507402	down	anthocyanidin 5,3-O-glucosyltransferase, putative, expressed
1.229205	up	1.329342	down	anthocyanin 3-O-beta-glucosyltransferase, putative, expressed
3.569365	up	1.023449	up	anthranilate phosphoribosyltransferase, chloroplast precursor, putative, expressed
3.044472	up	1.53568	up	AP2 domain containing protein, expressed
2.742023	up	1.811512	up	AP2 domain containing protein, expressed

2.406054	down	3.698539	up	AP2 domain containing protein, expressed
1.377515	up	5.472486	up	AP2 domain containing protein, expressed
1.211522	down	3.082589	up	AP2 domain containing protein, expressed
1.173176	up	2.767064	up	AP2 domain containing protein, expressed
2.858017	up	4.280007	up	app1, putative, expressed
1.424308	up	2.324771	up	armadillo repeat-containing protein, putative, expressed
1.2765	up	2.552396	up	armadillo/beta-catenin repeat family protein, putative, expressed
3.289814	down	1.202197	down	arogenate dehydrogenase 1, chloroplast precursor, putative, expressed
1.306148	down	2.87662	up	aspartic proteinase nepenthesin-2 precursor, putative, expressed
1.686439	up	2.300387	up	aspartic proteinase, putative, expressed
2.04839	down	1.319744	down	ATP/GTP binding protein, putative, expressed
2.040972	up	1.271282	up	auxin-independent growth promoter protein, putative, expressed
5.952239	up	1.118097	up	auxin-induced protein 5NG4, putative, expressed
5.459221	up	1.142094	up	auxin-induced protein 5NG4, putative, expressed
1.707377	up	1.357836	down	auxin-induced protein 5NG4, putative, expressed
3.103741	up	2.481036	down	auxin-induced protein 5NG4, putative, expressed
1.483708	up	3.840587	up	avr9/Cf-9 rapidly elicited protein, putative, expressed
3.278476	down	1.05135	down	B3 DNA binding domain containing protein, expressed
1.780373	up	1.644943	down	B3 DNA binding domain containing protein, expressed
2.434333	down	1.037294	down	basic helix-loop-helix, putative, expressed
1.529129	down	2.185391	down	basic helix-loop-helix, putative, expressed
1.094765	down	4.598829	down	basic helix-loop-helix, putative, expressed
2.05085	down	3.305957	up	B-box zinc finger family protein, putative, expressed
3.369435	down	1.442064	up	beta-amylase, putative, expressed
1.14641	down	1.328764	up	beta-amylase, putative, expressed
4.006994	up	4.534203	up	beta-amylase, putative, expressed
2.885604	up	9.442935	up	beta-amylase, putative, expressed
3.042629	down	1.031328	down	BRASSINOSTEROID INSENSITIVE 1-associated receptor kinase 1 precursor, putative, expressed
2.271943	down	2.936174	up	BTBN3 - Bric-a-Brac, Tramtrack, Broad Complex BTB domain with non-phototropic hypocotyl 3 NPH3 dc
2.277755	down	1.027022	down	BTBN5 - Bric-a-Brac, Tramtrack, Broad Complex BTB domain with non-phototropic hypocotyl 3 NPH3 an
1.163842	down	2.551388	up	BURP domain containing protein, expressed
1.196548	down	1.785698	up	bZIP transcription factor domain containing protein, expressed
1.076016	down	1.785134	up	bZIP transcription factor domain containing protein, expressed
3.086382	down	3.164647	up	bZIP transcription factor domain containing protein, expressed

2.621847	down	3.796058	up	bZIP transcription factor domain containing protein, expressed
2.496482	down	1.298838	down	CAAX amino terminal protease family protein, putative, expressed
1.542116	up	1.161788	down	cadmium tolerance factor, putative, expressed
1.372663	up	2.167247	up	cadmium tolerance factor, putative, expressed
2.527272	up	1.014542	down	calcium-binding EF hand family protein, putative, expressed
1.367432	down	1.93184	up	caleosin related protein, putative, expressed
2.943546	down	3.472707	up	caleosin related protein, putative, expressed
1.097126	up	1.044812	down	calmodulin binding protein, putative, expressed
1.013047	up	1.176474	down	calmodulin-binding transcription activator 2, putative, expressed
2.040682	down	1.234217	down	CAMK_CAMK_like_ULKh_APGy.3 - CAMK includes calcium/calmodulin depedent protein kinases, expre[illegible]
1.313861	down	1.108234	down	CAMK_KIN1/SNF1/Nim1_like.17 - CAMK includes calcium/calmodulin depedent protein kinases, expres[illegible]
1.417908	down	2.462603	down	CAMK_KIN1/SNF1/Nim1_like.2 - CAMK includes calcium/calmodulin depedent protein kinases, express[illegible]
2.065661	down	1.047288	down	carboxyvinyl-carboxyphosphonate phosphorylmutase, putative, expressed
1.368074	down	2.810634	down	cation efflux family protein, putative, expressed
2.601884	up	1.496651	up	CBS domain containing membrane protein, putative, expressed
4.17684	down	2.752386	up	CCT motif family protein, expressed
2.747412	down	2.061508	up	CCT motif family protein, expressed
1.976287	up	2.576811	up	CCT/B-box zinc finger protein, putative, expressed
2.162549	down	1.513607	down	CDK-activating kinase assembly factor MAT1 family protein, expressed
4.39369	up	2.624313	up	CESA4 - cellulose synthase, expressed
1.390342	up	2.31693	up	CESA5 - cellulose synthase, expressed
2.794356	up	1.123457	up	CESA6 - cellulose synthase, expressed
4.623538	up	4.568589	up	chalcone synthase, putative, expressed
1.164716	down	4.169658	up	chalcone--flavonone isomerase, putative, expressed
1.086504	down	11.23971	up	chalcone--flavonone isomerase, putative, expressed
1.293453	down	1.354126	up	CHIT14 - Chitinase family protein precursor, expressed
2.806501	down	1.691836	down	chitinase domain-containing protein 1 precursor, putative, expressed
1.292602	up	2.332433	up	chitin-inducible gibberellin-responsive protein, putative, expressed
1.822121	up	6.062529	up	chlorophyll A-B binding protein, putative, expressed
1.783593	up	4.903329	up	chlorophyll A-B binding protein, putative, expressed
1.556953	up	3.506981	down	chloroplast 50S ribosomal protein L20, putative, expressed
1.028072	down	2.949708	down	chloroplast 50S ribosomal protein L22, putative, expressed
1.140251	up	2.805073	down	chloroplast 50S ribosomal protein L23, putative
2.053509	down	1.213688	down	chorismate mutase, chloroplast precursor, putative, expressed

2.257701	up	1.49324	up	cinnamoyl CoA reductase, putative, expressed
2.581409	down	1.686722	down	Citrate transporter protein, putative, expressed
2.241355	up	1.30256	up	COBRA, putative, expressed
1.186867	up	2.578043	up	COBRA-like protein 7 precursor, putative, expressed
2.119536	down	1.340576	down	containing DUF163, putative, expressed
1.297771	up	1.320893	down	copine, putative, expressed
3.94168	down	2.845555	up	Core histone H2A/H2B/H3/H4 domain containing protein, putative, expressed
2.314577	down	3.743957	up	Core histone H2A/H2B/H3/H4 domain containing protein, putative, expressed
3.732133	down	2.513163	up	core histone H2A/H2B/H3/H4, putative, expressed
1.282831	down	1.169173	down	CPuORF1 - conserved peptide uORF-containing transcript, expressed
2.67712	up	1.125023	up	CPuORF12 - conserved peptide uORF-containing transcript, expressed
1.046965	up	1.382493	down	ctr copper transporter family protein, putative, expressed
10.806	down	4.837502	up	cupin domain containing protein, expressed
3.349082	up	2.581882	up	Cupin domain containing protein, expressed
2.511185	down	4.26501	up	Cupin domain containing protein, expressed
1.389085	up	2.768567	up	cupin domain containing protein, expressed
1.055958	down	2.419491	up	Cupin domain containing protein, expressed
2.265558	down	1.429489	down	cyclase family protein, putative, expressed
2.035329	down	1.341306	down	cyclase/dehydrase family protein, expressed
2.386722	down	1.219996	down	cyclin, putative, expressed
2.196547	down	1.310514	down	cyclin, putative, expressed
2.699861	down	1.260472	down	cyclin-dependent kinase C-2, putative, expressed
1.690214	up	2.143702	up	cyclin-T1-1, putative, expressed
2.866967	up	1.493819	up	cytidine/deoxycytidylate deaminase, putative, expressed
3.480004	up	1.437226	up	cytochrome b5-like Heme/Steroid binding domain containing protein, expressed
2.782683	up	1.278592	up	cytochrome P450 51, putative, expressed
10.98799	up	1.091396	up	cytochrome P450, putative, expressed
8.579556	up	1.474003	up	cytochrome P450, putative, expressed
3.771711	up	1.442978	down	cytochrome P450, putative, expressed
3.62171	down	1.0283	down	cytochrome P450, putative, expressed
3.443832	down	1.591148	down	cytochrome P450, putative, expressed
2.471694	down	1.09618	up	cytochrome P450, putative, expressed
2.369986	up	1.574669	up	cytochrome P450, putative, expressed
2.184164	up	1.274034	up	cytochrome P450, putative, expressed

2.166656	up	1.706314	up	cytochrome P450, putative, expressed
2.081692	down	1.957116	down	cytochrome P450, putative, expressed
2.024316	down	1.292858	down	cytochrome P450, putative, expressed
6.532462	down	4.1924	up	cytochrome P450, putative, expressed
3.035257	up	2.424943	down	cytochrome P450, putative, expressed
2.243637	down	2.012899	up	cytochrome P450, putative, expressed
1.693386	down	3.756316	up	cytochrome P450, putative, expressed
1.442729	down	2.049302	down	cytochrome P450, putative, expressed
1.294708	up	4.008868	up	cytochrome P450, putative, expressed
1.252559	down	2.276544	down	cytochrome P450, putative, expressed
1.348088	up	1.99814	down	cytokinin dehydrogenase precursor, putative, expressed
2.281955	up	3.053724	down	cytokinin-O-glucosyltransferase 2, putative, expressed
2.326407	down	1.024349	down	DCL, chloroplast precursor, putative, expressed
1.281346	up	1.659814	down	DEAD-box ATP-dependent RNA helicase, putative, expressed
5.025316	up	1.726078	up	decarboxylase, putative, expressed
2.933697	up	1.484606	up	DEF7 - Defensin and Defensin-like DEFL family, expressed
2.689756	up	1.287114	down	dehydration response related protein, putative, expressed
1.674532	up	2.270459	up	dehydration response related protein, putative, expressed
7.559901	down	1.918246	up	dehydrin, putative, expressed
1.815267	down	1.682105	up	dehydrin, putative, expressed
2.386311	down	2.646678	up	dehydrodolichyl diphosphate synthase, putative, expressed
4.58772	down	1.726064	up	dehydrogenase E1 component domain containing protein, expressed
4.416947	up	1.05334	down	dehydrogenase, putative, expressed
2.466206	up	1.847103	up	dehydrogenase, putative, expressed
4.298616	up	2.656395	up	dehydrogenase, putative, expressed
2.437655	down	1.206245	up	deoxynucleoside kinase family, putative, expressed
2.000215	up	2.054815	down	dienelactone hydrolase family protein, expressed
3.903153	up	1.253704	down	dihydroflavonol-4-reductase, putative, expressed
1.846055	up	4.406084	up	dirigent, putative, expressed
1.628224	up	5.035708	up	dirigent, putative, expressed
1.577406	up	4.366971	up	dirigent, putative, expressed
1.265161	up	1.111821	up	disease resistance protein RGA3, putative, expressed
2.238864	down	1.24048	down	DNA binding protein, putative, expressed
2.422442	down	1.029573	up	DNA repair protein Rad51, putative, expressed

2.064905	down	1.23601	down	DNA-binding protein-related, putative, expressed
2.029944	down	1.306313	down	DNA-directed RNA polymerase III subunit RPC9, putative, expressed
1.871857	up	2.323718	up	domain of unknown function DUF966 domain containing protein, expressed
1.351419	up	2.145885	up	DOMON domain containing protein, expressed
1.481491	down	2.419513	down	double-stranded RNA binding motif containing protein, expressed
1.142017	down	2.03862	down	double-stranded RNA binding motif containing protein, expressed
2.636575	down	1.195624	up	DTA2, putative, expressed
5.434534	down	3.623388	up	DUF1264 domain containing protein, putative, expressed
2.524781	up	3.408749	down	DUF260 domain containing protein, putative, expressed
15.01643	down	5.585529	up	DUF581 domain containing protein, expressed
1.845422	up	2.108861	up	DUF584 domain containing protein, putative, expressed
2.772708	down	3.32239	up	DUF623 domain containing protein, expressed
3.503859	down	1.00226	down	E2F-related protein, putative, expressed
1.496786	down	1.091932	up	early light-induced protein, chloroplast precursor, putative, expressed
2.413832	down	1.315502	down	ECT protein, putative, expressed
1.126317	down	1.418873	down	EF hand family protein, putative, expressed
1.003875	up	2.847833	up	EF hand family protein, putative, expressed
2.672205	down	2.794185	up	embryonic protein DC-8, putative, expressed
2.716204	up	1.552123	up	endoglucanase, putative, expressed
1.395142	up	2.388111	up	endoglucanase, putative, expressed
2.056446	up	1.070404	up	enoyl-CoA hydratase/isomerase family protein, putative, expressed
1.662653	down	1.312204	up	erythrocyte binding protein 3, putative, expressed
2.280515	down	2.372845	up	erythrocyte binding protein 3, putative, expressed
6.153073	down	2.482547	up	esterase, putative, expressed
1.500093	down	3.104502	up	ethylene-responsive transcription factor 2, putative, expressed
3.49388	up	1.104896	up	ethylene-responsive transcription factor, putative, expressed
3.281103	up	1.326598	up	eukaryotic aspartyl protease domain containing protein, expressed
1.19995	up	2.109336	up	exo70 exocyst complex subunit domain containing protein, expressed
1.268722	up	2.03569	up	exo70 exocyst complex subunit family protein, putative, expressed
2.144263	down	1.362664	down	exosome complex exonuclease, putative, expressed
3.232735	up	1.524977	up	expansin precursor, putative, expressed
3.092495	up	1.558468	up	expansin precursor, putative, expressed
3.136599	down	3.96517	up	expansin precursor, putative, expressed
40.42667	up	1.174277	up	expressed protein

16.1555	down	1.302683	down	expressed protein
11.38797	down	1.131501	up	expressed protein
10.93547	up	1.436005	up	expressed protein
7.081519	up	1.294121	up	expressed protein
6.972023	up	1.569563	up	expressed protein
5.99118	down	1.736049	up	expressed protein
5.680453	up	1.840963	up	expressed protein
3.706027	up	1.82995	up	expressed protein
3.607289	up	1.715637	up	expressed protein
3.512062	down	1.260353	up	expressed protein
3.487274	down	1.890616	down	expressed protein
3.216505	up	1.519573	up	expressed protein
3.108503	down	1.611723	down	expressed protein
3.059435	up	1.50468	up	expressed protein
3.047933	down	1.074216	down	expressed protein
2.985745	down	1.387342	up	expressed protein
2.972185	down	1.016382	down	expressed protein
2.882447	down	1.465411	down	expressed protein
2.855823	up	1.960345	up	expressed protein
2.762901	up	1.004734	up	expressed protein
2.745008	up	1.240756	up	expressed protein
2.7254	down	1.123018	up	expressed protein
2.723658	down	1.092334	up	expressed protein
2.670037	up	1.687128	up	expressed protein
2.654631	down	1.321088	up	expressed protein
2.623261	down	1.304894	up	expressed protein
2.613402	down	1.131782	down	expressed protein
2.610441	down	1.082554	down	expressed protein
2.579845	down	1.336401	down	expressed protein
2.544279	down	1.229699	down	expressed protein
2.520826	down	1.432866	down	expressed protein
2.513687	up	1.023237	up	expressed protein
2.512702	down	1.077901	up	expressed protein
2.489843	up	1.498314	up	expressed protein

2.465378	down	1.145869	down	expressed protein
2.449039	down	1.165682	down	expressed protein
2.439977	down	1.805021	down	expressed protein
2.436262	down	1.426327	down	expressed protein
2.370849	down	1.231515	down	expressed protein
2.308079	up	1.447983	up	expressed protein
2.303797	down	1.25676	down	expressed protein
2.283931	down	1.095144	down	expressed protein
2.252695	down	1.120258	down	expressed protein
2.250541	down	1.494452	down	expressed protein
2.245149	down	1.52593	down	expressed protein
2.204547	up	1.044753	down	expressed protein
2.203824	up	1.002587	up	expressed protein
2.182841	up	1.858202	up	expressed protein
2.169224	down	1.786532	down	expressed protein
2.168625	up	1.599602	up	expressed protein
2.168387	down	1.185412	down	expressed protein
2.165136	down	1.926326	down	expressed protein
2.158466	up	1.827925	up	expressed protein
2.149105	up	1.893273	up	expressed protein
2.142646	down	1.291897	down	expressed protein
2.141079	down	1.429516	down	expressed protein
2.134449	down	1.983546	down	expressed protein
2.131053	down	1.414791	down	expressed protein
2.123125	down	1.562656	down	expressed protein
2.123051	down	1.086677	down	expressed protein
2.120774	down	1.378919	down	expressed protein
2.12022	down	1.784997	down	expressed protein
2.117822	down	1.350452	down	expressed protein
2.11751	down	1.072907	down	expressed protein
2.110868	up	1.177687	up	expressed protein
2.110868	up	1.177687	up	expressed protein
2.105391	up	1.575914	up	expressed protein
2.098399	up	1.89591	up	expressed protein

2.086253	down	1.79579	down	expressed protein
2.08357	down	1.060948	down	expressed protein
2.080206	down	1.078598	down	expressed protein
2.072425	down	1.380118	down	expressed protein
2.071058	up	1.305483	up	expressed protein
2.069364	up	1.464395	up	expressed protein
2.053838	up	1.345547	up	expressed protein
2.05365	down	1.520243	down	expressed protein
2.036522	down	1.53027	down	expressed protein
2.031771	up	1.639602	up	expressed protein
2.02429	down	1.927968	down	expressed protein
2.022509	down	1.759538	down	expressed protein
2.021194	down	1.123358	down	expressed protein
2.01551	down	1.109583	down	expressed protein
2.001081	down	1.495708	down	expressed protein
1.962112	up	1.23624	down	expressed protein
1.628287	up	1.590784	down	expressed protein
1.577842	up	1.772523	down	expressed protein
1.519065	up	1.336964	down	expressed protein
1.433279	up	1.87174	down	expressed protein
1.392959	up	1.511238	down	expressed protein
1.369095	up	1.281446	up	expressed protein
1.284356	down	1.212763	down	expressed protein
1.272744	up	1.853982	down	expressed protein
1.230994	up	1.202569	down	expressed protein
1.131759	down	1.144054	down	expressed protein
1.100145	down	1.217026	up	expressed protein
1.069104	up	1.436009	down	expressed protein
1.043549	up	1.02518	up	expressed protein
16.64236	down	4.167274	up	expressed protein
12.31187	down	3.444203	up	expressed protein
5.475219	down	9.886033	up	expressed protein
5.259385	down	2.800866	up	expressed protein
4.376525	down	2.19192	down	expressed protein

4.367728	up	2.830917	up	expressed protein
4.227061	up	2.419833	up	expressed protein
3.348844	down	2.137501	up	expressed protein
3.164056	down	3.290049	up	expressed protein
3.127378	down	2.318387	up	expressed protein
3.065805	down	3.43482	up	expressed protein
2.913556	down	3.040388	up	expressed protein
2.84747	down	2.053008	up	expressed protein
2.713594	down	2.026297	up	expressed protein
2.691339	up	2.14611	up	expressed protein
2.659398	down	2.055245	up	expressed protein
2.574747	down	2.301785	up	expressed protein
2.533043	down	2.357088	up	expressed protein
2.426994	down	4.454128	up	expressed protein
2.276132	up	2.011068	down	expressed protein
2.25165	down	3.290965	up	expressed protein
2.250202	down	2.957155	up	expressed protein
2.250078	down	3.491786	up	expressed protein
2.223576	down	4.089724	up	expressed protein
2.186104	down	2.633093	up	expressed protein
2.185881	down	2.079981	up	expressed protein
2.15044	down	3.125576	up	expressed protein
2.14342	down	2.96822	up	expressed protein
2.099068	up	2.019136	down	expressed protein
2.086076	down	2.127346	up	expressed protein
2.025332	down	2.806335	up	expressed protein
2.009149	down	2.276074	up	expressed protein
1.963087	up	3.242828	up	expressed protein
1.774059	up	2.364401	up	expressed protein
1.765913	down	3.225428	down	expressed protein
1.685844	up	3.455174	up	expressed protein
1.612551	up	2.282387	up	expressed protein
1.575231	up	3.466929	down	expressed protein
1.541221	up	2.304072	up	expressed protein

1.532802	down	2.28313	down	expressed protein
1.48151	up	3.241815	up	expressed protein
1.434019	up	3.169937	up	expressed protein
1.344967	up	2.766864	up	expressed protein
1.344231	up	2.985297	up	expressed protein
1.34005	up	2.91438	up	expressed protein
1.257248	up	3.528007	up	expressed protein
1.252485	up	2.079625	up	expressed protein
1.248788	down	2.084251	down	expressed protein
1.243938	down	2.411356	down	expressed protein
1.234793	up	2.582829	up	expressed protein
1.232955	up	3.181011	up	expressed protein
1.230316	down	6.662136	up	expressed protein
1.204529	up	2.072703	up	expressed protein
1.196356	up	2.829829	up	expressed protein
1.193061	up	2.139452	up	expressed protein
1.180475	down	3.405422	up	expressed protein
1.161006	down	3.08429	down	expressed protein
1.159323	down	4.604854	down	expressed protein
1.126595	up	3.003385	down	expressed protein
1.121306	down	2.073037	down	expressed protein
1.117249	down	3.722818	up	expressed protein
1.112887	up	3.567696	down	expressed protein
1.102268	up	4.273533	down	expressed protein
1.053432	down	5.514153	down	expressed protein
1.038672	up	4.279795	down	expressed protein
1.024195	up	5.251268	up	expressed protein
1.022022	up	2.418257	up	expressed protein
1.01378	up	2.556024	up	expressed protein
1.004832	down	2.733999	down	expressed protein
1.17721	up	1.284111	down	FAD dependent oxidoreductase domain containing protein, expressed
11.93535	up	1.378473	up	fasciclin domain containing protein, expressed
4.333635	up	1.197246	up	fasciclin domain containing protein, expressed
6.219558	up	5.46204	up	fasciclin domain containing protein, expressed

1.41013	up	2.057253	up	fasciclin-like arabinogalactan protein 8 precursor, putative, expressed
1.26144	up	2.05043	up	fatty acid desaturase, putative, expressed
1.220068	up	1.092984	up	fatty acid hydroxylase, putative, expressed
2.012191	up	1.955072	up	FERONIA receptor-like kinase, putative, expressed
2.179322	up	2.630183	down	ferric reductase, putative, expressed
2.078283	down	1.177418	down	ferric-chelate reductase, putative, expressed
3.891317	up	3.047452	down	ferritin-1, chloroplast precursor, putative, expressed
2.991546	up	2.202829	down	ferritin-1, chloroplast precursor, putative, expressed
2.829456	up	2.373216	down	ferritin-1, chloroplast precursor, putative, expressed
1.729494	down	2.719088	down	fibronectin type 3 and ankyrin repeat domains 1 protein, putative, expressed
3.999392	up	1.071395	up	flavonol sulfotransferase, putative, expressed
7.915718	up	1.017151	down	flavonol synthase/flavanone 3-hydroxylase, putative, expressed
7.763259	up	1.021207	up	flavonol synthase/flavanone 3-hydroxylase, putative, expressed
5.15306	up	1.098599	up	flavonol synthase/flavanone 3-hydroxylase, putative, expressed
3.005447	up	1.114393	up	flavonol synthase/flavanone 3-hydroxylase, putative, expressed
2.318981	down	1.34791	down	flavonol synthase/flavanone 3-hydroxylase, putative, expressed
2.002638	up	1.503056	up	flavonol synthase/flavanone 3-hydroxylase, putative, expressed
1.555915	down	2.946591	down	flavonol synthase/flavanone 3-hydroxylase, putative, expressed
1.145411	down	2.635821	down	flavonol synthase/flavanone 3-hydroxylase, putative, expressed
2.16638	up	1.117719	up	frataxin, putative, expressed
2.214075	up	1.298266	up	fringe-related protein, putative, expressed
1.336124	down	2.705017	down	FYVE zinc finger domain containing protein, expressed
2.256199	down	1.678721	down	GA15368-PA, putative, expressed
2.472014	down	2.922898	up	GASR9 - Gibberellin-regulated GASA/GAST/Snakin family protein precursor, expressed
1.134727	up	2.108457	up	GATA zinc finger domain containing protein, expressed
9.366871	up	1.624813	up	GDSL-like lipase/acylhydrolase, putative, expressed
4.779107	up	1.28134	up	GDSL-like lipase/acylhydrolase, putative, expressed
2.660243	up	1.271378	up	GDSL-like lipase/acylhydrolase, putative, expressed
2.346083	up	1.770796	up	GDSL-like lipase/acylhydrolase, putative, expressed
1.438413	up	3.107599	up	gibberellin 2-beta-dioxygenase, putative, expressed
2.369408	down	1.394651	down	GIL1, putative, expressed
1.03357	down	1.819171	down	GLTP domain containing protein, putative, expressed
2.155341	up	1.176243	up	glucan endo-1,3-beta-glucosidase precursor, putative, expressed
2.560424	down	1.109638	up	glucan endo-1,3-beta-glucosidase-related, putative, expressed

1.055223	down	1.207553	up	glucosyltransferase, putative, expressed
1.237005	down	1.509765	down	glutamine synthetase, catalytic domain containing protein, expressed
2.999058	down	1.591759	down	glutathione S-transferase, putative, expressed
1.194813	up	1.483823	down	glutathione S-transferase, putative, expressed
2.747822	down	2.815951	up	glutathione S-transferase, putative, expressed
1.003485	down	2.509414	up	glutathione S-transferase, putative, expressed
1.10114	down	1.346817	up	glyceraldehyde-3-phosphate dehydrogenase, putative, expressed
2.527259	up	1.990457	up	glycosyl hydrolase family 10 protein, putative, expressed
2.613928	up	1.882862	up	glycosyl hydrolase, putative, expressed
4.662705	down	2.012111	up	glycosyl hydrolase, putative, expressed
1.074815	up	3.620036	up	glycosyl hydrolase, putative, expressed
2.484668	up	1.005063	down	glycosyl hydrolases family 17, putative, expressed
1.639841	up	2.490612	up	GRAS family transcription factor domain containing protein, expressed
2.054858	down	1.025138	down	growth regulator related protein, putative, expressed
2.833462	up	1.024913	down	HAD superfamily phosphatase, putative, expressed
10.76049	up	2.608739	up	HAD superfamily phosphatase, putative, expressed
1.814329	down	1.626828	up	haloacid dehalogenase-like hydrolase family protein, putative, expressed
8.856831	up	2.53485	up	harpin-induced protein 1 domain containing protein, expressed
1.025032	up	2.125795	up	harpin-induced protein 1 domain containing protein, expressed
1.257599	down	1.025022	down	heavy metal associated domain containing protein, expressed
1.232309	down	1.256548	up	heavy metal associated domain containing protein, expressed
3.89514	down	3.752384	up	heavy metal-associated domain containing protein, expressed
1.539632	up	2.452856	up	heavy metal-associated domain containing protein, expressed
3.745846	down	1.128396	down	helix-loop-helix DNA-binding domain containing protein, expressed
3.169953	down	1.199824	down	helix-loop-helix DNA-binding domain containing protein, expressed
3.570886	down	2.037235	down	helix-loop-helix DNA-binding domain containing protein, expressed
1.661127	up	3.396944	up	helix-loop-helix DNA-binding domain containing protein, expressed
1.423228	up	2.953203	up	helix-loop-helix DNA-binding domain containing protein, expressed
2.400982	down	1.457725	down	helix-loop-helix DNA-binding protein, putative, expressed
3.060922	up	1.592943	up	hexokinase, putative, expressed
1.914628	down	2.260877	down	HMG1/2, putative, expressed
1.882034	down	2.143325	down	HMG1/2, putative, expressed
2.56158	down	1.0367	down	homeobox associated leucine zipper, putative, expressed
3.520965	down	2.147474	up	homeobox associated leucine zipper, putative, expressed

9.250532	up	1.265244	down	homeobox protein knotted-1, putative, expressed
2.124754	up	1.352682	up	homocysteine S-methyltransferase protein, putative, expressed
2.025727	down	1.107619	down	HORMA domain containing protein, putative, expressed
1.453805	up	3.345161	up	HSF-type DNA-binding domain containing protein, expressed
1.002038	up	2.32469	down	HSF-type DNA-binding domain containing protein, expressed
2.076744	down	1.529294	down	HVA22, putative, expressed
2.216226	down	2.589887	up	hydrolase protein, putative, expressed
1.736152	up	2.536785	up	hydrolase, alpha/beta fold family protein, putative, expressed
1.189639	up	3.502164	up	hypothetical protein
2.19885	down	2.92098	up	hypoxia-responsive family protein, putative, expressed
2.076411	up	1.818269	up	IBR domain containing protein, putative, expressed
1.328657	up	2.299468	up	inactive receptor kinase At1g27190 precursor, putative, expressed
4.319721	up	1.624537	up	indole-3-glycerol phosphate synthase, chloroplast precursor, putative, expressed
3.190221	up	1.842473	up	indole-3-glycerol phosphate synthase, chloroplast precursor, putative, expressed
2.622512	down	1.263604	down	inhibitor I family protein, putative, expressed
2.519636	up	1.097357	up	inner membrane protein, putative, expressed
2.0959	up	1.174361	up	inner membrane protein, putative, expressed
1.33399	up	2.749409	up	inorganic phosphate transporter, putative, expressed
1.31417	up	2.37035	up	inorganic phosphate transporter, putative, expressed
2.143037	up	3.407625	down	integral membrane protein DUF6 containing protein, expressed
3.790566	up	1.159101	down	integral membrane protein, putative, expressed
2.140039	up	1.11866	up	integral membrane protein, putative, expressed
4.429414	down	3.139573	up	integral membrane transporter family protein, putative, expressed
2.250392	up	1.052735	down	invertase/pectin methylesterase inhibitor family protein, putative, expressed
1.471979	up	1.466517	down	invertase/pectin methylesterase inhibitor family protein, putative, expressed
2.114839	up	2.498221	down	IQ calmodulin-binding motif domain containing protein, expressed
4.037751	down	2.620806	up	IQ calmodulin-binding motif family protein, putative, expressed
2.676163	up	2.288227	up	IQ calmodulin-binding motif family protein, putative, expressed
5.877354	up	2.519355	up	Jacalin-like lectin domain containing protein, putative, expressed
3.416503	up	1.257785	up	jmjC domain containing protein, expressed
2.988766	up	1.978746	up	jmjC domain containing protein, expressed
2.417132	up	1.281269	up	KI domain interacting kinase 1, putative, expressed
2.528125	down	1.632654	down	kinase, pfkB family, putative, expressed
1.802493	up	2.297469	up	KIP1, putative, expressed

3.447036	down	4.571094	up	laccase precursor protein, putative, expressed
1.329925	up	1.428851	down	lachrymatory factor synthase, putative, expressed
22.43767	down	2.50943	up	late embryogenesis abundant group 1, putative, expressed
10.83851	down	11.45717	up	late embryogenesis abundant protein D-34, putative, expressed
4.157907	down	1.109692	up	late embryogenesis abundant protein, group 3, putative, expressed
2.103493	up	1.944196	up	leaf senescence related protein, putative, expressed
2.040714	up	2.151276	up	lectin-like protein kinase, putative, expressed
1.449741	down	3.251754	up	lectin-like receptor kinase, putative, expressed
1.019824	up	1.105918	up	Leucine Rich Repeat family protein, expressed
2.094332	up	1.096242	up	Leucine rich repeat N-terminal domain containing protein, putative, expressed
1.575416	up	2.484628	up	leucine-rich repeat family protein, putative, expressed
2.336979	up	4.08591	down	lipase class 3 family protein, putative, expressed
3.386663	down	2.024163	up	lipase precursor, putative, expressed
3.707141	up	1.181997	up	lipase, putative, expressed
2.345594	down	2.116745	up	lipase, putative, expressed
2.321603	up	1.32292	up	LRP1, putative, expressed
1.249989	up	1.033209	down	LTPL118 - Protease inhibitor/seed storage/LTP family protein precursor, expressed
1.237465	up	1.011963	up	LTPL118 - Protease inhibitor/seed storage/LTP family protein precursor, expressed
5.839119	down	1.880109	up	LTPL153 - Protease inhibitor/seed storage/LTP family protein precursor, expressed
2.839934	up	1.215469	up	lysine-rich arabinogalactan protein 19 precursor, putative, expressed
2.019145	down	1.595619	down	MA3 domain containing protein, expressed
2.989939	down	1.7598	down	major facilitator superfamily antiporter, putative, expressed
2.123769	down	1.703733	down	major facilitator superfamily antiporter, putative, expressed
4.872914	up	1.153457	down	male sterility protein, putative, expressed
1.644975	down	1.372074	up	MATE efflux family protein, putative, expressed
1.669483	up	2.818871	up	MATE efflux family protein, putative, expressed
1.406389	up	1.043144	down	maturase K, putative, expressed
1.071778	down	3.557989	up	MBTB48 - Bric-a-Brac, Tramtrack, Broad Complex BTB domain with Meprin and TRAF Homology MATH [illegible]
1.093435	up	2.138362	up	membrane associated DUF588 domain containing protein, putative, expressed
1.120982	up	1.222378	up	metal cation transporter, putative, expressed
2.189915	up	2.147512	down	metal cation transporter, putative, expressed
1.61613	up	2.671083	up	metal cation transporter, putative, expressed
3.287839	up	2.823479	down	metallothionein, putative, expressed
3.277093	up	2.2649	down	metallothionein, putative, expressed

3.11083	up	2.254661	down	metallothionein, putative, expressed
2.202172	up	2.049295	down	metallothionein, putative, expressed
1.767695	down	5.111124	up	methyladenine glycosylase, putative, expressed
9.840168	up	1.457703	up	methyltransferase, putative, expressed
2.627941	down	1.001833	down	methyltransferase, putative, expressed
1.44653	down	3.17742	up	MFS18 protein precursor, putative, expressed
71.74509	up	1.195603	down	microtubule-associated protein, putative, expressed
32.31743	up	1.242778	up	microtubule-associated protein, putative, expressed
12.11752	down	1.866581	up	mitochondrial import inner membrane translocase subunit Tim17, putative, expressed
1.626628	up	1.163188	up	mitochondrial import inner membrane translocase subunit Tim17, putative, expressed
2.001127	down	2.827915	up	mRNA-decapping enzyme, putative, expressed
2.337876	up	1.796401	up	MYB family transcription factor, putative, expressed
2.307248	down	1.594206	down	MYB family transcription factor, putative, expressed
2.017169	up	1.568946	up	MYB family transcription factor, putative, expressed
1.346622	up	1.393876	up	MYB family transcription factor, putative, expressed
1.833599	up	2.416066	up	MYB family transcription factor, putative, expressed
1.309612	down	3.524975	down	MYB family transcription factor, putative, expressed
1.209015	down	2.749251	down	MYB family transcription factor, putative, expressed
1.136151	up	3.258992	down	MYB family transcription factor, putative, expressed
1.10926	up	2.841073	down	MYB family transcription factor, putative, expressed
1.475216	up	1.183082	up	Myb transcription factor, putative, expressed
1.221646	down	1.225041	up	Myb transcription factor, putative, expressed
4.105022	up	4.257714	down	Myb transcription factor, putative, expressed
1.138441	up	3.591859	up	Myb transcription factor, putative, expressed
1.445169	up	3.412938	down	myb-like DNA-binding domain containing protein, putative, expressed
2.147219	up	1.043011	down	NAC domain-containing protein 67, putative, expressed
1.004279	down	1.731236	up	NAD dependent epimerase/dehydratase family protein, putative, expressed
6.029842	up	1.903585	up	NADH-ubiquinone oxidoreductase, mitochondrial precursor, putative, expressed
1.012589	down	3.250256	up	NADH-ubiquinone oxidoreductase, mitochondrial precursor, putative, expressed
2.303685	down	3.876813	up	NADP-dependent oxidoreductase, putative, expressed
1.137799	down	2.54233	down	NADPH-dependent oxidoreductase, putative, expressed
1.755998	up	2.160369	up	NB-ARC domain containing protein, expressed
1.288878	down	2.952144	up	NBS-LRR disease resistance protein, putative, expressed
1.932448	up	9.184818	up	nicotianamine synthase, putative, expressed

7.703261	up	4.043671	up	nitrilase-associated protein, putative, expressed
5.913166	up	4.138518	up	nitrilase-associated protein, putative, expressed
1.407009	up	4.575794	up	nmrA-like family domain containing protein, expressed
2.752101	up	1.422098	up	no apical meristem protein, putative, expressed
2.344415	up	1.067221	up	no apical meristem protein, putative, expressed
1.741177	up	2.308688	up	no apical meristem protein, putative, expressed
2.642584	up	1.14107	up	nodulin MtN3 family protein, putative, expressed
2.642584	up	1.14107	up	nodulin MtN3 family protein, putative, expressed
1.610885	up	1.319345	down	nodulin MtN3 family protein, putative, expressed
1.358577	up	1.041759	down	nodulin MtN3 family protein, putative, expressed
1.109627	down	2.616712	up	nodulin MtN3 family protein, putative, expressed
2.956564	up	1.692228	up	nodulin, putative, expressed
2.922206	up	1.670987	up	nodulin, putative, expressed
2.301365	down	2.011202	up	nodulin, putative, expressed
3.099164	down	2.938289	down	non-symbiotic hemoglobin 2, putative, expressed
3.316496	down	2.248537	up	nuclear transcription factor Y subunit, putative, expressed
2.086922	down	1.266676	down	nucleoporin, putative, expressed
1.805901	up	2.189145	up	nucleoside transporter, putative, expressed
4.401721	up	1.12352	down	nucleoside-triphosphatase, putative, expressed
4.400109	up	1.096612	up	nucleoside-triphosphatase, putative, expressed
3.37112	up	1.037836	up	nucleoside-triphosphatase, putative, expressed
1.308354	up	1.415139	down	oligopeptide transporter, putative, expressed
6.317597	up	2.126556	down	O-methyltransferase, putative, expressed
1.780584	down	2.471175	down	O-methyltransferase, putative, expressed
2.859661	down	1.046487	down	ORG4, putative, expressed
1.119092	up	2.092774	up	organic cation transporter protein, putative, expressed
2.160916	up	2.099713	down	oryzain alpha chain precursor, putative, expressed
2.460668	down	3.849843	up	Os11bglu36 SFR2 homologue, expressed
3.401326	down	1.322442	down	Os1bglu5 - beta-glucosidase homologue, similar to G. max isohydroxyurate hydrolase, expressed
3.273249	down	1.553879	down	Os1bglu5 - beta-glucosidase homologue, similar to G. max isohydroxyurate hydrolase, expressed
2.072673	down	1.575085	down	Os1bglu5 - beta-glucosidase homologue, similar to G. max isohydroxyurate hydrolase, expressed
1.425038	up	1.93205	down	Os6bglu24 - beta-glucosidase homologue, similar to G. max isohydroxyurate hydrolase, expressed
1.353739	up	2.922274	up	OsCML27 - Calmodulin-related calcium sensor protein, expressed
2.130764	down	1.0637	up	OsFBDUF30 - F-box and DUF domain containing protein, expressed

1.314952	up	3.77997	up	OsFBK10 - F-box domain and kelch repeat containing protein, expressed
2.950242	down	1.20067	up	OsFBL35 - F-box domain and LRR containing protein, expressed
2.14311	down	1.870262	down	OsFBL9 - F-box domain and LRR containing protein, expressed
1.669956	up	1.688932	down	OsFBO10 - F-box and other domain containing protein, expressed
1.129534	down	2.378126	down	OsFBX148 - F-box domain containing protein, expressed
3.228421	down	1.383741	down	OsFBX224 - F-box domain containing protein, expressed
2.294293	down	1.580566	down	OsFBX226 - F-box domain containing protein, expressed
2.682724	up	1.540506	up	OsFBX31 - F-box domain containing protein, expressed
2.778009	down	1.835713	down	OsFBX331 - F-box domain containing protein, expressed
1.008184	down	1.435979	down	OsFBX336 - F-box domain containing protein, expressed
2.335736	down	2.102299	up	OsFBX357 - F-box domain containing protein, expressed
1.594691	up	3.387186	down	osFTL6 FT-Like6 homologous to Flowering Locus T gene; contains Pfam profile PF01161: Phosphatidyle
3.425651	down	1.797846	down	OsGrx_C17 - glutaredoxin subgroup III, expressed
2.215921	down	2.933126	up	OsGrx_S12 - glutaredoxin subgroup I, expressed
1.44582	up	2.350952	up	OsGrx_S2 - glutaredoxin subgroup III, expressed
2.050492	down	2.149806	down	OsMan04 - Endo-Beta-Mannanase, expressed
2.135175	down	1.543996	down	OsPOP17 - Putative Prolyl Oligopeptidase homologue, expressed
3.127964	up	2.665862	up	OsPOP9 - Putative Prolyl Oligopeptidase homologue, expressed
1.451082	down	1.500775	up	OsRCI2-6 - Hydrophobic protein LTI6B, expressed
3.738481	down	2.565219	up	OsRhmbd12 - Putative Rhomboid homologue, expressed
2.164868	down	1.503846	down	OsSub61 - Putative Subtilisin homologue, expressed
3.548146	down	2.512832	up	OsSub63 - Putative Subtilisin homologue, expressed
3.396401	down	2.170564	up	OsSub63 - Putative Subtilisin homologue, expressed
1.361301	up	1.2964	down	OsSub8 - Putative Subtilisin homologue, expressed
1.328011	down	1.454275	up	OsWAK60 - OsWAK receptor-like protein kinase, expressed
1.343312	up	1.057475	down	OsWAK8 - OsWAK receptor-like protein kinase, expressed
10.76718	up	1.527888	up	oxidoreductase, aldo/keto reductase family protein, putative, expressed
2.549192	up	1.077644	down	oxidoreductase, aldo/keto reductase family protein, putative, expressed
1.381016	up	1.821348	down	oxidoreductase, aldo/keto reductase family protein, putative, expressed
1.381964	up	2.501266	up	oxidoreductase, putative, expressed
2.019977	down	1.342681	down	oxidoreductase, short chain dehydrogenase/reductase family, putative, expressed
1.198957	down	2.689332	up	oxygen evolving enhancer protein 3, identical, putative, expressed
2.872605	down	2.106377	up	P21-Rho-binding domain containing protein, putative, expressed
2.480114	down	1.104651	down	paramyosin, putative, expressed

2.945997	up	1.158007	up	patatin, putative, expressed
2.14384	down	1.417298	down	pectinacetylesterase domain containing protein, expressed
1.086352	down	2.500279	up	pectinesterase, putative, expressed
2.498917	down	1.298051	down	pentatricopeptide repeat protein PPR986-12, putative, expressed
3.351651	down	1.041169	down	pentatricopeptide, putative, expressed
2.406429	down	1.900476	down	pentatricopeptide, putative, expressed
2.682293	up	1.967421	up	peptidase, putative, expressed
2.316342	down	1.303392	down	peptide transporter PTR2, putative, expressed
1.441966	up	2.064998	up	peptide transporter PTR2, putative, expressed
6.222453	down	1.023077	down	peptide transporter, putative, expressed
1.221138	up	2.028699	up	peptide-N4-asparagine amidase A, putative, expressed
2.321034	down	2.170149	up	peptidyl-prolyl cis-trans isomerase CYP40, putative, expressed
2.257954	down	2.064577	up	permease domain containing protein, putative, expressed
3.442101	up	1.128725	down	peroxidase precursor, putative, expressed
2.907029	up	1.02687	up	peroxidase precursor, putative, expressed
2.043623	up	1.728009	up	peroxidase precursor, putative, expressed
1.35916	up	1.517717	down	peroxidase precursor, putative, expressed
1.286474	up	1.883165	down	peroxidase precursor, putative, expressed
20.16946	up	2.966879	up	peroxidase precursor, putative, expressed
3.91793	down	3.758279	up	peroxidase precursor, putative, expressed
3.265883	up	2.023756	down	peroxidase precursor, putative, expressed
2.955048	down	3.635639	up	peroxidase precursor, putative, expressed
1.362888	up	2.007978	up	peroxidase precursor, putative, expressed
1.057151	down	1.101016	down	peroxiredoxin, putative, expressed
4.850151	down	5.494378	up	peroxiredoxin, putative, expressed
1.874444	up	2.459764	up	peroxiredoxin, putative, expressed
2.20859	down	2.387971	up	peroxisomal biogenesis factor 11, putative, expressed
2.237536	down	1.027635	down	PHD finger family protein, putative, expressed
2.299442	down	1.145569	up	PHD finger protein, putative, expressed
2.758639	up	1.8242	up	PHD-finger domain containing protein, putative, expressed
2.568292	up	1.554627	up	phenylalanine ammonia-lyase, putative, expressed
2.789496	up	2.10096	up	phenylalanine ammonia-lyase, putative, expressed
2.278338	up	2.562387	up	phenylalanine ammonia-lyase, putative, expressed
1.378299	up	2.920575	up	phenylalanine ammonia-lyase, putative, expressed

3.212926	up	1.608799	up	phosphate carrier protein, mitochondrial precursor, putative, expressed
1.026702	up	1.593196	down	phosphate transporter 1, putative, expressed
1.384026	up	2.621693	up	phosphate-induced protein 1 conserved region domain containing protein, expressed
2.049795	down	1.753909	down	phosphatidylinositol 3- and 4-kinase family protein, putative, expressed
1.36381	up	1.377995	down	phosphatidylinositol transfer, putative, expressed
1.272896	down	1.062369	down	phospho-2-dehydro-3-deoxyheptonate aldolase, chloroplast precursor, putative, expressed
1.878898	up	2.085419	up	phospho-2-dehydro-3-deoxyheptonate aldolase, chloroplast precursor, putative, expressed
3.075235	down	1.532759	up	phosphoglycerate kinase protein, putative, expressed
12.71059	up	1.184411	up	phospholipase D, putative, expressed
2.278072	up	1.741244	up	phosphoribosyl transferase, putative, expressed
1.327232	down	2.009205	down	phosphoribosyl transferase, putative, expressed
1.258248	up	2.770499	down	photosystem I assembly protein ycf4, putative, expressed
1.00569	up	2.652746	down	photosystem I iron-sulfur center, putative, expressed
1.567512	down	1.102125	down	phytosulfokine receptor precursor, putative, expressed
1.135404	down	1.410525	down	phytosulfokine receptor precursor, putative, expressed
2.497541	down	1.284159	down	piwi domain containing protein, expressed
1.00483	up	1.219725	down	plant protein of unknown function domain containing protein, expressed
1.656265	up	2.556996	up	plant-specific domain TIGR01589 family protein, expressed
1.1037	down	1.070924	down	plant-specific domain TIGR01615 family protein, expressed
1.586237	up	3.284069	up	plastocyanin-like domain containing protein, putative, expressed
1.996539	up	2.095207	up	pleiotropic drug resistance protein, putative, expressed
3.017158	up	2.261303	down	POEI46 - Pollen Ole e I allergen and extensin family protein precursor, expressed
2.528568	up	1.805724	up	pollen-specific protein SF21, putative, expressed
6.14488	up	1.01494	up	polyprenyl synthetase, putative, expressed
4.239292	up	1.130894	up	polyprenyl synthetase, putative, expressed
1.009482	up	1.36025	down	possible lysine decarboxylase domain containing protein, expressed
2.528411	up	2.140729	down	possible lysine decarboxylase domain containing protein, expressed
2.090156	down	1.109462	down	PPR repeat containing protein, expressed
2.786127	down	1.21214	down	PPR repeat domain containing protein, putative, expressed
2.100453	down	1.074766	down	PPR repeat domain containing protein, putative, expressed
1.085811	up	1.0596	down	PRAS-rich protein, putative, expressed
1.70622	down	1.411793	up	protein kinase domain containing protein, expressed
1.493948	up	2.424765	up	protein kinase domain containing protein, expressed
1.440913	up	2.783425	up	protein kinase domain containing protein, expressed

1.423609	up	2.16261	up	protein kinase domain containing protein, expressed
1.935295	up	2.154882	up	protein kinase family protein, putative, expressed
1.572093	up	4.231888	up	protein kinase family protein, putative, expressed
1.374153	up	2.848655	up	protein kinase, putative, expressed
3.964746	down	1.692727	up	protein phosphatase 2C, putative, expressed
3.93988	down	1.802066	up	protein phosphatase 2C, putative, expressed
2.256272	down	1.119971	down	protein phosphatase 2C, putative, expressed
2.238436	up	1.468771	up	protein phosphatase 2C, putative, expressed
1.496229	up	2.25831	up	protein phosphatase 2C, putative, expressed
1.113093	down	2.876063	up	protein phosphatase 2C, putative, expressed
1.399743	up	1.679578	down	proton-dependent oligopeptide transport, putative, expressed
2.028594	up	2.8206	down	PSF3 - Putative GINS complex subunit, expressed
4.018794	down	1.000163	down	pumilio-family RNA binding protein, putative, expressed
3.334882	down	1.254684	down	pumilio-family RNA binding repeat containing protein, expressed
1.156113	up	1.052817	down	pumilio-family RNA binding repeat containing protein, expressed
3.8268	down	2.100934	up	RALFL14 - Rapid ALkalinization Factor RALF family protein precursor, expressed
2.229417	down	3.552338	up	RALFL18 - Rapid ALkalinization Factor RALF family protein precursor, expressed
2.063566	down	3.571372	up	RALFL24 - Rapid ALkalinization Factor RALF family protein precursor, expressed
1.807661	up	2.538443	up	RALFL6 - Rapid ALkalinization Factor RALF family protein precursor, expressed
2.077741	up	1.864876	up	ras-related protein, putative, expressed
2.266063	down	1.917368	down	RCN3 Centroradialis-like1 homogous to TFL1 gene; contains Pfam profile PF01161: Phosphatidylethan
2.087996	up	1.683205	up	receptor kinase, putative, expressed
4.151064	up	1.061377	up	receptor protein kinase CRINKLY4 precursor, putative, expressed
1.819456	up	1.149587	down	receptor-like protein kinase 2 precursor, putative, expressed
1.335627	up	1.516037	down	receptor-like protein kinase 2 precursor, putative, expressed
2.021751	up	1.646928	up	receptor-like protein kinase 2, putative, expressed
1.270418	up	1.345203	down	receptor-like protein kinase precursor, putative, expressed
2.01933	down	1.444482	down	receptor-like protein kinase, putative, expressed
2.469859	up	1.534825	up	regulatory protein, putative, expressed
2.213466	up	1.061485	up	regulatory protein, putative, expressed
1.752728	up	2.568228	up	relA-SpoT like protein RSH4, putative, expressed
1.199531	down	2.019775	down	remorin C-terminal domain containing protein, putative, expressed
2.723065	up	2.330707	down	respiratory burst oxidase, putative, expressed
1.3618	down	2.932903	up	reticuline oxidase-like protein precursor, putative, expressed

1.171807	down	5.012171	down	reticulon domain containing protein, putative, expressed
1.892993	down	3.239466	down	retinol dehydrogenase, putative, expressed
1.26562	up	1.338851	down	retrotransposon protein, putative, SINE subclass, expressed
3.039481	up	1.32628	down	retrotransposon protein, putative, Ty3-gypsy subclass, expressed
1.374478	up	1.353439	up	retrotransposon protein, putative, Ty3-gypsy subclass, expressed
1.226468	up	1.619291	up	retrotransposon protein, putative, Ty3-gypsy subclass, expressed
1.082812	up	1.560596	down	retrotransposon protein, putative, Ty3-gypsy subclass, expressed
1.029001	up	2.236883	down	retrotransposon protein, putative, Ty3-gypsy subclass, expressed
1.000138	up	5.163294	down	retrotransposon protein, putative, Ty3-gypsy subclass, expressed
6.626076	down	1.680892	up	retrotransposon protein, putative, unclassified, expressed
2.536034	down	1.178248	down	retrotransposon protein, putative, unclassified, expressed
2.536034	down	1.178248	down	retrotransposon protein, putative, unclassified, expressed
2.515437	down	1.006101	up	retrotransposon protein, putative, unclassified, expressed
2.020256	down	1.457692	down	retrotransposon protein, putative, unclassified, expressed
1.52025	up	1.421081	down	retrotransposon protein, putative, unclassified, expressed
1.028072	down	1.561538	down	retrotransposon protein, putative, unclassified, expressed
10.38355	up	2.927601	up	retrotransposon protein, putative, unclassified, expressed
2.346221	down	3.871441	up	retrotransposon protein, putative, unclassified, expressed
1.607416	up	2.276861	up	retrotransposon protein, putative, unclassified, expressed
2.326677	down	3.318096	up	retrotransposon, putative, centromere-specific, expressed
2.148582	down	2.418722	up	rhythmically expressed gene 2 protein, putative, expressed
2.036257	down	2.725473	up	rhythmically expressed gene 2 protein, putative, expressed
1.205293	up	3.250572	up	riboflavin biosynthesis protein ribAB, chloroplast precursor, putative, expressed
3.033089	up	1.131742	down	RING-H2 finger protein, putative, expressed
2.168691	up	1.893245	up	RING-H2 finger protein, putative, expressed
2.079239	down	1.241179	down	RING-H2 finger protein, putative, expressed
1.031348	up	1.196279	up	RING-H2 finger protein, putative, expressed
2.498489	down	1.41438	down	RNA polymerase III subunit RPC82 family protein, putative, expressed
2.001554	up	2.936464	down	RNA recognition motif containing protein, putative, expressed
1.932465	up	2.647389	up	RNA recognition motif containing protein, putative, expressed
1.543187	up	2.036701	up	salt stress root protein RS1, putative, expressed
17.16657	up	1.61585	up	SAM dependent carboxyl methyltransferase, putative, expressed
1.270736	down	2.45219	down	SAM dependent carboxyl methyltransferase, putative, expressed
1.667751	up	2.438915	up	S-domain receptor-like protein kinase, putative, expressed

1.211899	up	2.258965	up	senescence-associated protein, putative, expressed
1.559503	up	2.441332	up	serine/threonine-protein kinase BRI1-like 1 precursor, putative, expressed
3.640898	up	1.031092	down	sex determination protein tasselseed-2, putative, expressed
2.521662	up	1.161452	up	sex determination protein tasselseed-2, putative, expressed
1.012646	up	2.632937	down	SHR5-receptor-like kinase, putative, expressed
2.172715	down	1.671839	down	Skp1 family, dimerisation domain containing protein, expressed
1.305209	up	2.004323	up	SNARE domain containing protein, putative, expressed
1.778082	up	2.337357	up	sodium/calcium exchanger 1 precursor, putative, expressed
1.008827	down	1.973673	up	splicing factor U2AF, putative, expressed
2.222366	up	1.46346	up	STE_MEKK_ste11_MAP3K.19 - STE kinases include homologs to sterile 7, sterile 11 and sterile 20 from y
2.192073	up	4.056266	up	STE_MEKK_ste11_MAP3K.7 - STE kinases include homologs to sterile 7, sterile 11 and sterile 20 from ye
4.739967	down	1.501475	up	STE_PAK_Ste20_Slob_Wnk.1 - STE kinases include homologs to sterile 7, sterile 11 and sterile 20 from y
3.821402	down	1.340288	up	STE_PAK_Ste20_Slob_Wnk.1 - STE kinases include homologs to sterile 7, sterile 11 and sterile 20 from y
2.21171	down	1.411178	down	STE_PAK_Ste20_Slob_Wnk.1 - STE kinases include homologs to sterile 7, sterile 11 and sterile 20 from y
1.742515	up	1.532745	down	sulfate transporter, putative, expressed
1.241016	up	2.288057	up	syntaxin, putative, expressed
3.007434	up	1.338942	up	targeting protein for Xklp2, putative, expressed
16.15422	up	1.903322	up	terpene synthase, putative, expressed
5.34456	up	3.615257	down	terpene synthase, putative, expressed
5.119659	up	2.079978	up	terpene synthase, putative, expressed
4.323813	up	2.949336	down	terpene synthase, putative, expressed
2.335549	down	1.312383	down	tesmin/TSO1-like CXC domain containing protein, expressed
1.230571	up	2.049126	up	tetratricopeptide repeat domain containing protein, expressed
1.395251	down	1.20036	down	thaumatin family domain containing protein, expressed
1.828847	down	2.211968	down	thaumatin family domain containing protein, expressed
1.878268	up	1.997475	down	thiamine biosynthesis protein thiC, putative, expressed
1.493806	up	2.183284	up	TKL_IRAK_CrRLK1L-1.5 - The CrRLK1L-1 subfamily has homology to the CrRLK1L homolog, expressed
1.899326	up	2.331974	up	TKL_IRAK_DUF26-la.1 - DUF26 kinases have homology to DUF26 containing loci, expressed
1.176975	up	1.742238	down	TKL_IRAK_DUF26-la.5 - DUF26 kinases have homology to DUF26 containing loci, expressed
1.740567	up	1.816714	down	TKL_IRAK_DUF26-lc.12 - DUF26 kinases have homology to DUF26 containing loci, expressed
1.287366	up	1.242625	down	TKL_IRAK_DUF26-lc.12 - DUF26 kinases have homology to DUF26 containing loci, expressed
2.188949	down	3.01025	up	TMS membrane protein/tumour differentially expressed protein, putative, expressed
2.269694	up	1.195435	up	transcription factor TF2, putative, expressed
2.868788	down	1.291663	down	transcription factor, putative, expressed

2.209863	down	3.640633	up	transcription factor, putative, expressed
1.183213	up	1.917342	down	transferase family protein, putative, expressed
1.175246	down	2.515784	down	transferase family protein, putative, expressed
1.210083	up	1.398056	down	transmembrane amino acid transporter protein, putative, expressed
1.09021	down	1.118361	down	transporter family protein, putative, expressed
2.302157	down	1.172437	down	transporter, major facilitator family, putative, expressed
2.950395	down	2.553988	up	transporter, major facilitator family, putative, expressed
1.262433	up	2.197604	up	transporter, major facilitator family, putative, expressed
1.2166	up	1.095879	down	transporter, monovalent cation:proton antiporter-2 family, putative, expressed
2.972008	up	1.332433	up	transporter, putative, expressed
3.574678	up	2.604195	up	transporter-related, putative, expressed
14.46518	down	2.794493	up	transposon protein, putative, CACTA, En/Spm sub-class, expressed
1.277437	up	2.708681	up	transposon protein, putative, CACTA, En/Spm sub-class, expressed
1.262474	down	4.324728	down	transposon protein, putative, CACTA, En/Spm sub-class, expressed
1.503812	down	2.684111	down	transposon protein, putative, Mutator sub-class, expressed
1.345458	down	2.730067	down	transposon protein, putative, Mutator sub-class, expressed
27.71414	down	1.37977	up	transposon protein, putative, unclassified, expressed
2.17066	down	1.305626	down	transposon protein, putative, unclassified, expressed
2.14757	down	1.002987	up	transposon protein, putative, unclassified, expressed
2.058713	down	1.596286	down	transposon protein, putative, unclassified, expressed
1.082506	up	1.390971	up	transposon protein, putative, unclassified, expressed
1.55583	up	2.701458	up	transposon protein, putative, unclassified, expressed
1.78849	up	1.278681	down	trehalase precursor, putative, expressed
2.038711	down	1.966166	down	tRNA methyltransferase, putative, expressed
2.026015	down	1.302101	down	tRNA-specific adenosine deaminase 1, putative, expressed
2.250252	down	1.474942	down	two-component response regulator, putative, expressed
1.199394	down	1.092071	down	tyrosine protein kinase domain containing protein, putative, expressed
1.397346	up	2.390096	up	ubiquitin-conjugating enzyme, putative, expressed
2.615522	down	1.064253	up	U-box domain containing protein, expressed
1.30825	up	2.086849	up	U-box protein CMPG1, putative, expressed
1.085929	down	1.075075	up	UDP-glucoronosyl and UDP-glucosyl transferase domain containing protein, expressed
2.260092	down	1.220621	down	uncharacterized ACR, COG1399 family protein, expressed
2.612082	down	1.529523	down	uncharacterized glycosyltransferase, putative, expressed
4.436347	down	5.664622	up	universal stress protein domain containing protein, putative, expressed

1.078696	down	1.029772	down	urease accessory protein F, putative, expressed
1.90969	up	1.700701	down	wall-associated kinase 1, putative, expressed
4.025886	up	1.220445	up	WD domain, G-beta repeat domain containing protein, expressed
2.839211	down	1.115234	down	WD domain, G-beta repeat domain containing protein, expressed
2.01392	down	1.17336	down	white-brown complex homolog protein 12, putative, expressed
3.538103	down	3.21718	up	white-brown complex homolog protein, putative, expressed
1.337979	down	1.127198	up	wound induced protein, putative, expressed
2.961757	up	1.489873	up	WRKY11, expressed
3.090279	up	8.731094	up	WRKY28, expressed
2.670276	up	1.306887	up	WRKY40, expressed
1.851116	up	3.123218	up	WRKY7, expressed
1.483477	up	2.964778	up	WRKY71, expressed
1.392079	up	2.325579	up	WRKY76, expressed
1.334395	down	1.682665	up	WRKY77, expressed
2.610919	up	1.716249	up	X8 domain containing protein, expressed
9.011562	up	1.118947	up	xylem cysteine proteinase 2 precursor, putative, expressed
2.006769	down	1.57599	down	ZF-HD protein dimerisation region containing protein, expressed
1.804151	up	7.560363	up	ZIM domain containing protein, putative, expressed
1.783154	up	12.22992	up	ZIM domain containing protein, putative, expressed
1.878443	up	1.022055	down	zinc finger CCCH type family protein, putative, expressed
1.393095	down	1.895826	up	zinc finger CCCH type family protein, putative, expressed
1.163355	down	1.452634	up	zinc finger CCCH type family protein, putative, expressed
1.063938	down	1.339753	up	zinc finger CCCH type family protein, putative, expressed
2.108974	up	1.805218	up	zinc finger family protein, putative, expressed
5.096291	down	2.466946	up	zinc finger family protein, putative, expressed
1.290876	up	2.815782	up	zinc finger family protein, putative, expressed
3.672399	up	1.201802	down	zinc finger, C3HC4 type domain containing protein, expressed
2.451077	up	1.643757	up	zinc finger, C3HC4 type domain containing protein, expressed
2.134031	up	1.907358	up	Zinc finger, C3HC4 type domain containing protein, expressed
2.01248	up	1.679737	up	zinc finger, C3HC4 type domain containing protein, expressed
1.139619	up	1.390521	up	zinc finger, C3HC4 type domain containing protein, expressed
1.240722	down	2.360615	down	zinc finger, C3HC4 type domain containing protein, expressed
1.202139	up	2.019002	up	zinc finger, C3HC4 type domain containing protein, expressed
2.460719	up	1.667644	up	zinc finger, C3HC4 type, domain containing protein, expressed

2.026939	down	1.279777	down	zinc-binding protein, putative, expressed
1.767619	up	2.251918	up	zinc-finger protein, putative, expressed
2.905284	up	1.004234	down	
2.417191	down	1.430986	down	
2.210309	down	1.554939	down	
2.079016	down	1.281418	down	
1.154087	down	1.037001	down	
3.260347	up	1.506198	down	
11.01777	down	1.188046	down	
7.333852	down	1.032012	down	
2.270892	down	3.160096	up	
3.001891	down	2.222239	up	
2.150982	down	2.836988	up	
2.359219	down	2.470055	up	
2.592396	down	2.7125	up	
2.129025	down	2.082836	up	
2.03612	down	2.039057	up	
2.084076	up	3.042002	down	
1.384538	down	3.277022	down	
1.256993	up	8.3222	down	
1.237128	up	3.252971	down	
1.047025	down	2.177539	down	
1.827227	up	3.893597	up	
1.14891	up	2.702279	up	
1.104904	down	2.339105	up	
4.697317	down	2.065021	up	
2.154078	up	4.200573	up	

omain, expressed
ıd coiled-coil domains, expressed

ssed
sed
ed

domain, expressed

:thanolamine-binding protein, expressed

olamine-binding protein, expressed

/east, expressed
east, expressed
/east, expressed
/east, expressed
/east, expressed

Probe Sets	Putative Function
Os.12373.1.S1_at	12-oxophytodienoate reductase, putative, expressed
OsAffx.17696.1.S1_at	2-aminoethanethiol dioxygenase, putative, expressed
Os.17130.1.S1_at	adenylate kinase, putative, expressed
Os.10908.1.S1_a_at	alpha-amylase precursor, putative, expressed
Os.11407.1.S1_at	amine oxidase, flavin-containing, domain containing protein, expressed
Os.50896.1.S1_at	amino acid transporter, putative, expressed
Os.17247.1.S1_at	ANTH, putative, expressed
Os.26406.2.S1_x_at	anthranilate phosphoribosyltransferase, chloroplast precursor, putative, [illegible]
OsAffx.27278.1.S1_x_at	AP2 domain containing protein, expressed
OsAffx.27278.1.S1_at	AP2 domain containing protein, expressed
Os.18003.1.S1_at	auxin-independent growth promoter protein, putative, expressed
Os.27624.1.S1_at	calcium-binding EF hand family protein, putative, expressed
OsAffx.29695.1.S1_s_at	CBS domain containing membrane protein, putative, expressed
Os.10926.1.S1_at	CESA6 - cellulose synthase, expressed
OsAffx.17611.1.S1_at	cinnamoyl CoA reductase, putative, expressed
Os.15633.1.S1_at	COBRA, putative, expressed
Os.15210.2.S1_x_at	CPuORF12 - conserved peptide uORF-containing transcript, expressed
Os.41883.1.S1_at	cytidine/deoxycytidylate deaminase, putative, expressed
Os.52752.1.S1_at	cytochrome b5-like Heme/Steroid binding domain containing protein, expressed
Os.22935.1.S1_at	cytochrome P450 51, putative, expressed
Os.11417.1.S1_at	cytochrome P450, putative, expressed
Os.14951.1.S1_at	cytochrome P450, putative, expressed
OsAffx.7246.1.S1_x_at	cytochrome P450, putative, expressed
OsAffx.19825.1.S1_x_at	cytochrome P450, putative, expressed
OsAffx.18404.1.S1_at	decarboxylase, putative, expressed
Os.10651.1.S1_at	DEF7 - Defensin and Defensin-like DEFL family, expressed
Os.19038.1.S1_at	dehydration response related protein, putative, expressed
Os.8684.1.S1_a_at	dehydrogenase, putative, expressed
Os.6089.1.S1_at	dehydrogenase, putative, expressed
Os.54545.1.S1_at	dihydroflavonol-4-reductase, putative, expressed
OsAffx.26423.1.S1_at	endoglucanase, putative, expressed
OsAffx.30751.1.S1_at	enoyl-CoA hydratase/isomerase family protein, putative, expressed
OsAffx.20436.1.S1_x_at	ethylene-responsive transcription factor, putative, expressed
Os.49519.1.S1_at	eukaryotic aspartyl protease domain containing protein, expressed
Os.2938.1.S1_at	expansin precursor, putative, expressed
Os.2938.1.S1_x_at	expansin precursor, putative, expressed
Os.5066.1.S1_at	expressed protein
Os.5593.1.S1_at	expressed protein
Os.32478.1.S1_at	expressed protein
Os.6683.1.S1_at	expressed protein
Os.28176.2.S1_x_at	expressed protein
OsAffx.2803.1.S1_at	expressed protein
Os.13482.1.S1_x_at	expressed protein
Os.7078.1.S1_at	expressed protein
Os.26406.1.S1_at	expressed protein
Os.9536.1.S1_at	expressed protein
OsAffx.25404.1.S1_at	expressed protein
Os.15813.1.S1_at	expressed protein
Os.4923.1.S1_at	expressed protein

OsAffx.4002.1.S1_s_at	expressed protein
Os.12999.1.S1_at	expressed protein
Os.48986.1.S1_s_at	expressed protein
Os.7770.1.S1_at	expressed protein
OsAffx.26838.1.S1_at	expressed protein
Os.55253.1.S1_at	expressed protein
OsAffx.27346.1.S1_at	expressed protein
Os.57191.1.S1_at	expressed protein
Os.16563.1.S1_at	expressed protein
Os.53407.1.S1_at	expressed protein
Os.53407.1.S1_x_at	expressed protein
Os.18712.1.S1_at	expressed protein
Os.10525.1.S1_at	expressed protein
OsAffx.18503.1.S1_at	expressed protein
Os.54454.1.S1_at	expressed protein
Os.5431.1.S1_at	FERONIA receptor-like kinase, putative, expressed
Os.50805.1.S1_at	flavonol sulfotransferase, putative, expressed
Os.31778.2.S1_x_at	flavonol synthase/flavanone 3-hydroxylase, putative, expressed
Os.31778.1.S1_x_at	flavonol synthase/flavanone 3-hydroxylase, putative, expressed
Os.31778.2.S1_at	flavonol synthase/flavanone 3-hydroxylase, putative, expressed
Os.53217.1.S1_x_at	flavonol synthase/flavanone 3-hydroxylase, putative, expressed
Os.52646.1.S1_at	flavonol synthase/flavanone 3-hydroxylase, putative, expressed
Os.9073.1.S1_at	frataxin, putative, expressed
Os.32462.1.S1_a_at	fringe-related protein, putative, expressed
Os.37457.1.S1_at	GDSL-like lipase/acylhydrolase, putative, expressed
Os.46844.1.S1_at	GDSL-like lipase/acylhydrolase, putative, expressed
OsAffx.25749.1.S1_at	glucan endo-1,3-beta-glucosidase precursor, putative, expressed
Os.8022.1.S1_at	glycosyl hydrolase family 10 protein, putative, expressed
OsAffx.27816.1.S1_at	glycosyl hydrolase, putative, expressed
Os.4159.1.S1_at	glycosyl hydrolases family 17, putative, expressed
Os.10534.1.S1_at	HAD superfamily phosphatase, putative, expressed
OsAffx.23999.1.S1_x_at	hexokinase, putative, expressed
Os.37866.1.S1_at	homocysteine S-methyltransferase protein, putative, expressed
Os.12642.1.S1_at	IBR domain containing protein, putative, expressed
Os.50903.2.S1_x_at	indole-3-glycerol phosphate synthase, chloroplast precursor, putative, expressed
Os.50903.1.S1_at	indole-3-glycerol phosphate synthase, chloroplast precursor, putative, expressed
OsAffx.25798.1.S1_at	inner membrane protein, putative, expressed
Os.20700.1.S1_at	inner membrane protein, putative, expressed
Os.5373.1.S1_at	jmjC domain containing protein, expressed
OsAffx.2983.1.S1_x_at	jmjC domain containing protein, expressed
Os.33145.1.S1_at	KI domain interacting kinase 1, putative, expressed
Os.20717.1.S1_at	leaf senescence related protein, putative, expressed
OsAffx.17977.1.S1_at	Leucine rich repeat N-terminal domain containing protein, putative, expressed
Os.50126.1.S1_at	lipase, putative, expressed
Os.9355.1.S1_at	LRP1, putative, expressed
Os.5927.1.S1_at	lysine-rich arabinogalactan protein 19 precursor, putative, expressed
Os.8413.2.A1_a_at	male sterility protein, putative, expressed
Os.1726.1.S1_at	MYB family transcription factor, putative, expressed
**Os.3388.1.S1_x_at**	**MYB family transcription factor, putative, expressed**
Os.44475.1.S1_x_at	NADH-ubiquinone oxidoreductase, mitochondrial precursor, putative, expressed

Os.7235.1.S1_at	no apical meristem protein, putative, expressed
Os.17090.1.S1_at	no apical meristem protein, putative, expressed
Os.54933.1.S1_at	nodulin, putative, expressed
Os.46711.1.S1_at	nodulin, putative, expressed
Os.47730.2.S1_x_at	nucleoside-triphosphatase, putative, expressed
Os.11908.1.S1_s_at	nucleoside-triphosphatase, putative, expressed
Os.47730.1.S1_x_at	nucleoside-triphosphatase, putative, expressed
Os.15941.2.S1_x_at	OsFBX31 - F-box domain containing protein, expressed
Os.4666.1.S1_at	oxidoreductase, aldo/keto reductase family protein, putative, expressed
Os.49524.1.S1_at	patatin, putative, expressed
Os.54966.1.S1_at	peptidase, putative, expressed
Os.7832.1.S1_at	peroxidase precursor, putative, expressed
Os.9893.1.S1_at	peroxidase precursor, putative, expressed
Os.11561.2.S1_a_at	peroxidase precursor, putative, expressed
Os.54501.1.S1_at	PHD-finger domain containing protein, putative, expressed
Os.10930.1.S1_at	phenylalanine ammonia-lyase, putative, expressed
OsAffx.17942.1.S1_at	phosphate carrier protein, mitochondrial precursor, putative, expressed
Os.29866.1.S1_at	phosphoribosyl transferase, putative, expressed
Os.625.1.S1_at	pollen-specific protein SF21, putative, expressed
OsAffx.23247.1.S1_x_at	polyprenyl synthetase, putative, expressed
OsAffx.23247.1.S1_at	polyprenyl synthetase, putative, expressed
Os.9705.1.S1_at	protein phosphatase 2C, putative, expressed
Os.9217.1.S1_at	ras-related protein, putative, expressed
OsAffx.19285.1.S1_at	receptor kinase, putative, expressed
Os.26411.2.A1_x_at	receptor protein kinase CRINKLY4 precursor, putative, expressed
Os.26870.2.A1_x_at	receptor-like protein kinase 2, putative, expressed
Os.27955.1.S1_at	regulatory protein, putative, expressed
Os.27955.2.S1_x_at	regulatory protein, putative, expressed
Os.10497.1.S1_s_at	retrotransposon protein, putative, Ty3-gypsy subclass, expressed
Os.35921.2.S1_x_at	RING-H2 finger protein, putative, expressed
Os.35921.1.S1_at	RING-H2 finger protein, putative, expressed
OsAffx.27612.1.S1_at	SAM dependent carboxyl methyltransferase, putative, expressed
Os.49410.1.A1_at	sex determination protein tasselseed-2, putative, expressed
Os.44598.1.S1_x_at	sex determination protein tasselseed-2, putative, expressed
Os.6085.1.S1_at	STE_MEKK_ste11_MAP3K.19 - STE kinases include homologs to sterile 7, sterile 1
Os.9681.1.S1_at	targeting protein for Xklp2, putative, expressed
OsAffx.14364.1.S1_s_at	transcription factor TF2, putative, expressed
Os.48856.1.S1_at	transporter, putative, expressed
Os.51744.1.A1_at	WD domain, G-beta repeat domain containing protein, expressed
Os.30512.1.S1_at	WRKY11, expressed
OsAffx.30783.1.S1_at	WRKY40, expressed
Os.37620.1.S1_at	X8 domain containing protein, expressed
Os.38796.1.S1_s_at	zinc finger family protein, putative, expressed
Os.1438.1.S1_at	zinc finger, C3HC4 type domain containing protein, expressed
Os.18247.1.S1_a_at	Zinc finger, C3HC4 type domain containing protein, expressed
Os.27330.1.A1_at	zinc finger, C3HC4 type domain containing protein, expressed
Os.14848.1.S1_at	zinc finger, C3HC4 type domain containing protein, expressed
OsAffx.28216.1.S1_at	zinc finger, C3HC4 type, domain containing protein, expressed
Os.53413.1.S1_at	NA

ed

l1 and sterile 20 from yeast, expressed

Probe Sets	Putative Function
Os.34674.1.S1_at	peptide transporter PTR2, putative, expressed
Os.5236.1.S1_at	retrotransposon protein, putative, unclassified, expressed
Os.46162.1.S1_at	HORMA domain containing protein, putative, expressed
Os.25117.1.A1_at	expressed protein
Os.17416.1.S1_at	major facilitator superfamily antiporter, putative, expressed
OsAffx.22176.1.S1_x	major facilitator superfamily antiporter, putative, expressed
Os.50964.1.S1_at	BRASSINOSTEROID INSENSITIVE 1-associated receptor kinase 1 precursor, putative, expre
Os.27319.1.A1_at	expressed protein
Os.1423.1.S1_at	expressed protein
Os.27235.1.S1_at	tRNA methyltransferase, putative, expressed
Os.21231.1.S1_at	helix-loop-helix DNA-binding domain containing protein, expressed
Os.27778.2.S1_at	expressed protein
Os.37184.1.S1_at	chitinase domain-containing protein 1 precursor, putative, expressed
Os.53241.1.S1_at	expressed protein
Os.27759.1.S1_at	expressed protein
Os.48002.1.A1_x_at	RNA polymerase III subunit RPC82 family protein, putative, expressed
Os.10963.1.S1_at	DNA-binding protein-related, putative, expressed
Os.33610.2.A1_at	expressed protein
Os.52869.1.S1_at	MYB family transcription factor, putative, expressed
Os.27684.1.S2_at	pumilio-family RNA binding protein, putative, expressed
OsAffx.23899.1.S1_a	kinase, pfkB family, putative, expressed
Os.17497.1.S1_a_at	transporter, major facilitator family, putative, expressed
Os.9321.1.S1_a_at	peptide transporter, putative, expressed
OsAffx.23976.1.S1_a	expressed protein
Os.26537.1.S1_a_at	Os1bglu5 - beta-glucosidase homologue, similar to G. max isohydroxyurate hydrolase, ex
Os.26537.1.S1_at	Os1bglu5 - beta-glucosidase homologue, similar to G. max isohydroxyurate hydrolase, ex
Os.26537.2.S1_x_at	Os1bglu5 - beta-glucosidase homologue, similar to G. max isohydroxyurate hydrolase, ex
Os.10797.1.S1_at	expressed protein
Os.49427.1.S1_at	beta-amylase, putative, expressed
Os.35316.1.S1_at	expressed protein
OsAffx.11931.1.S1_a	transcription factor, putative, expressed
Os.54001.1.S1_at	chorismate mutase, chloroplast precursor, putative, expressed
Os.52183.1.S1_at	expressed protein
Os.49173.1.S1_at	PPR repeat domain containing protein, putative, expressed
Os.17213.2.S1_at	AGC_PKA/PKG_like.1 - ACG kinases include homologs to PKA, PKG and PKC, expressed
Os.51849.1.S1_at	transposon protein, putative, unclassified, expressed
Os.17022.1.S1_at	phosphatidylinositol 3- and 4-kinase family protein, putative, expressed
OsAffx.24464.1.S1_s	expressed protein
Os.37797.1.A1_at	ATP/GTP binding protein, putative, expressed
Os.5865.1.S1_at	OsFBL9 - F-box domain and LRR containing protein, expressed
Os.140.1.A1_s_at	PHD finger protein, putative, expressed
Os.22678.1.A1_s_at	expressed protein
Os.17834.1.S1_at	PPR repeat domain containing protein, putative, expressed
Os.55694.1.S1_at	DTA2, putative, expressed
Os.19186.1.S1_at	Citrate transporter protein, putative, expressed
Os.24954.1.A1_s_at	WD domain, G-beta repeat domain containing protein, expressed

Os.52180.1.S1_at	HVA22, putative, expressed
Os.10491.1.S1_at	expressed protein
Os.38278.1.S1_at	B3 DNA binding domain containing protein, expressed
Os.47694.1.S1_at	BTBN5 - Bric-a-Brac, Tramtrack, Broad Complex BTB domain with non-phototropic hypoc
Os.7298.2.S1_at	nucleoporin, putative, expressed
Os.8251.1.S1_at	U-box domain containing protein, expressed
Os.53016.1.S1_at	expressed protein
Os.24664.1.A1_at	basic helix-loop-helix, putative, expressed
Os.53969.1.S1_at	cyclin-dependent kinase C-2, putative, expressed
Os.7447.1.S1_a_at	protein phosphatase 2C, putative, expressed
Os.8290.1.S1_at	expressed protein
Os.6224.2.S1_at	expressed protein
Os.36647.1.S1_at	pentatricopeptide, putative, expressed
Os.33081.1.S1_at	expressed protein
Os.51810.1.S1_at	transposon protein, putative, unclassified, expressed
Os.54926.1.S1_at	GA15368-PA, putative, expressed
Os.50897.1.S1_at	piwi domain containing protein, expressed
Os.49920.2.S1_at	glucan endo-1,3-beta-glucosidase-related, putative, expressed
Os.24911.1.S1_at	cyclase/dehydrase family protein, expressed
Os.37320.1.S1_at	expressed protein
Os.47503.1.S1_at	exosome complex exonuclease, putative, expressed
Os.17160.1.S1_at	uncharacterized ACR, COG1399 family protein, expressed
Os.14949.1.S1_at	pumilio-family RNA binding repeat containing protein, expressed
Os.51166.1.S1_at	expressed protein
Os.14145.1.A1_at	ferric-chelate reductase, putative, expressed
OsAffx.26358.1.S1_a	ORG4, putative, expressed
Os.52761.1.A1_at	receptor-like protein kinase, putative, expressed
Os.17140.1.S1_x_at	transposon protein, putative, unclassified, expressed
Os.54497.1.S1_at	expressed protein
Os.52657.1.S1_at	carboxyvinyl-carboxyphosphonate phosphorylmutase, putative, expressed
Os.22522.1.A1_a_at	aminotransferase domain containing protein, putative, expressed
Os.14173.1.S1_at	retrotransposon protein, putative, unclassified, expressed
Os.14686.1.S1_at	zinc-binding protein, putative, expressed
Os.8447.1.S1_at	containing DUF163, putative, expressed
Os.12432.1.S1_a_at	expressed protein
OsAffx.4171.1.S1_s_	cyclin, putative, expressed
Os.36647.1.S1_x_at	expressed protein
Os.53243.1.S1_x_at	tRNA-specific adenosine deaminase 1, putative, expressed
Os.52632.1.S1_at	CAMK_CAMK_like_ULKh_APGy.3 - CAMK includes calcium/calmodulin depedent protein
Os.9618.1.S1_at	PHD finger family protein, putative, expressed
Os.27562.1.S1_at	ECT protein, putative, expressed
Os.7725.1.S1_at	pectinacetylesterase domain containing protein, expressed
Os.51826.1.S1_at	expressed protein
Os.18996.1.S1_at	MA3 domain containing protein, expressed
OsAffx.26840.1.S1_s	GIL1, putative, expressed
Os.7232.1.S1_at	white-brown complex homolog protein 12, putative, expressed
Os.49422.1.S1_x_at	expressed protein

Os.38760.1.S1_at	expressed protein
OsAffx.4464.1.S1_s_	CAAX amino terminal protease family protein, putative, expressed
Os.5094.1.S2_at	methyltransferase, putative, expressed
Os.14243.1.S1_at	expressed protein
Os.20865.1.S1_at	deoxynucleoside kinase family, putative, expressed
Os.8550.1.S1_x_at	expressed protein
Os.53970.1.S1_at	expressed protein
Os.50550.1.S1_at	OsFBDUF30 - F-box and DUF domain containing protein, expressed
Os.27959.1.A1_at	CDK-activating kinase assembly factor MAT1 family protein, expressed
Os.27290.1.A1_at	uncharacterized glycosyltransferase, putative, expressed
Os.19406.1.S1_at	two-component response regulator, putative, expressed
Os.14184.1.S1_at	cyclin, putative, expressed
Os.52292.1.S1_at	cytochrome P450, putative, expressed
Os.49566.1.S1_at	cytochrome P450, putative, expressed
Os.10743.1.S1_at	expressed protein
Os.18473.1.S1_at	arogenate dehydrogenase 1, chloroplast precursor, putative, expressed
Os.7468.1.S1_at	DNA binding protein, putative, expressed
OsAffx.28288.1.S1_a	expressed protein
Os.7147.1.S1_at	OsFBL35 - F-box domain and LRR containing protein, expressed
Os.9515.1.S1_x_at	STE_PAK_Ste20_Slob_Wnk.1 - STE kinases include homologs to sterile 7, sterile 11 and st
Os.9515.1.S1_at	STE_PAK_Ste20_Slob_Wnk.1 - STE kinases include homologs to sterile 7, sterile 11 and st
Os.9515.3.S1_x_at	STE_PAK_Ste20_Slob_Wnk.1 - STE kinases include homologs to sterile 7, sterile 11 and st
OsAffx.16228.1.S1_a	expressed protein
Os.29745.1.S2_at	OsFBX224 - F-box domain containing protein, expressed
Os.52957.1.S1_at	OsFBX226 - F-box domain containing protein, expressed
OsAffx.28461.1.S1_a	expressed protein
Os.53334.2.S1_x_at	expressed protein
Os.8081.1.A1_at	PPR repeat containing protein, expressed
Os.8811.1.S1_at	expressed protein
Os.18196.1.S1_at	paramyosin, putative, expressed
OsAffx.16741.1.S1_s	expressed protein
Os.24428.1.S1_at	OsPOP17 - Putative Prolyl Oligopeptidase homologue, expressed
OsAffx.29395.1.S1_a	tesmin/TSO1-like CXC domain containing protein, expressed
Os.6873.1.S1_at	ZF-HD protein dimerisation region containing protein, expressed
Os.11122.1.S1_at	flavonol synthase/flavanone 3-hydroxylase, putative, expressed
Os.28034.1.S1_at	homeobox associated leucine zipper, putative, expressed
Os.5369.1.S1_at	cytochrome P450, putative, expressed
Os.7276.1.A1_s_at	growth regulator related protein, putative, expressed
Os.8721.1.S1_at	expressed protein
Os.9823.1.S1_at	cyclase family protein, putative, expressed
Os.56058.1.S1_at	DCL, chloroplast precursor, putative, expressed
Os.49292.1.S1_at	expressed protein
Os.57568.1.S1_at	expressed protein
Os.23911.1.S1_at	OsFBX331 - F-box domain containing protein, expressed
Os.9485.1.S1_at	expressed protein
OsAffx.6495.1.S1_s_	Skp1 family, dimerisation domain containing protein, expressed
Os.53962.1.S1_at	expressed protein

Os.46446.1.S1_at	pentatricopeptide, putative, expressed
Os.49301.1.A1_at	expressed protein
Os.12052.1.S1_at	expressed protein
Os.8937.1.S1_at	expressed protein
Os.10564.1.A1_at	E2F-related protein, putative, expressed
Os.34118.1.S1_at	expressed protein
Os.6363.1.S1_at	OsSub61 - Putative Subtilisin homologue, expressed
Os.39214.1.S1_at	cytochrome P450, putative, expressed
Os.5441.1.S1_at	glutathione S-transferase, putative, expressed
Os.26772.2.S1_at	expressed protein
Os.27569.3.S1_at	expressed protein
Os.31883.1.A1_at	helix-loop-helix DNA-binding protein, putative, expressed
Os.19070.1.S1_at	amino acid transporter, putative, expressed
Os.50381.1.S1_at	expressed protein
Os.54307.1.S1_at	oxidoreductase, short chain dehydrogenase/reductase family, putative, expressed
Os.51061.1.S1_at	pentatricopeptide repeat protein PPR986-12, putative, expressed
Os.22485.1.A1_at	cytochrome P450, putative, expressed
Os.55402.1.S1_at	OsGrx_C17 - glutaredoxin subgroup III, expressed
Os.13051.1.S1_at	DNA repair protein Rad51, putative, expressed
Os.4788.1.S1_at	RING-H2 finger protein, putative, expressed
Os.57548.1.S1_at	RCN3 Centroradialis-like1 homogous to TFL1 gene; contains Pfam profile PF01161: Phos
Os.54220.1.S1_at	expressed protein
Os.21264.1.S1_s_at	expressed protein
Os.51942.1.S1_at	DNA-directed RNA polymerase III subunit RPC9, putative, expressed
Os.53455.1.S1_at	expressed protein
Os.9324.1.S1_at	N/A
Os.50841.1.S1_at	N/A
Os.23097.1.A1_at	N/A

essed

pressed
pressed
pressed

otyl 3 NPH3 and coiled-coil domains, expressed

kinases, expressed

erile 20 from yeast, expressed
erile 20 from yeast, expressed
erile 20 from yeast, expressed

:phatidylethanolamine-binding protein, expressed

Probe Sets	Putative Function
OsAffx.32325.1.S1_at	maturase K, putative, expressed
Os.34494.1.S1_at	mitochondrial import inner membrane translocase subunit Tim17, putative, expressed
Os.15516.1.S1_at	OsWAK60 - OsWAK receptor-like protein kinase, expressed
Os.46093.1.S1_at	possible lysine decarboxylase domain containing protein, expressed
Os.49381.1.S1_at	plant protein of unknown function domain containing protein, expressed
Os.52298.1.S1_at	expressed protein
Os.23264.1.A1_at	TKL_IRAK_DUF26-la.5 - DUF26 kinases have homology to DUF26 containing loci, expresse
Os.55250.1.S1_at	expressed protein
Os.52944.1.A1_at	zinc finger, C3HC4 type domain containing protein, expressed
Os.17174.1.S1_at	expressed protein
OsAffx.4511.1.S1_s_at	auxin-induced protein 5NG4, putative, expressed
Os.26884.1.S1_a_at	transposon protein, putative, unclassified, expressed
Os.49819.1.S1_at	receptor-like protein kinase 2 precursor, putative, expressed
Os.27299.1.A1_at	B3 DNA binding domain containing protein, expressed
Os.26807.1.S1_at	aluminum-activated malate transporter, putative, expressed
Os.11920.1.S1_s_at	expressed protein
Os.49503.1.S1_at	calmodulin-binding transcription activator 2, putative, expressed
Os.49818.1.S1_s_at	TKL_IRAK_DUF26-lc.12 - DUF26 kinases have homology to DUF26 containing loci, express
Os.52717.1.S1_at	pumilio-family RNA binding repeat containing protein, expressed
OsAffx.26826.1.S1_at	lachrymatory factor synthase, putative, expressed
Os.4974.1.S1_at	nodulin MtN3 family protein, putative, expressed
Os.8045.1.S1_at	UDP-glucoronosyl and UDP-glucosyl transferase domain containing protein, expressed
Os.778.1.S1_at	protein kinase domain containing protein, expressed
OsAffx.30872.1.S1_s_a	ankyrin repeat family protein, putative, expressed
Os.17762.1.S1_at	MATE efflux family protein, putative, expressed
Os.10829.1.S1_at	fatty acid hydroxylase, putative, expressed
OsAffx.3180.1.S1_at	sulfate transporter, putative, expressed
Os.15732.1.S1_s_at	metal cation transporter, putative, expressed
Os.18490.1.S1_x_at	thiamine biosynthesis protein thiC, putative, expressed
Os.7756.1.S1_at	invertase/pectin methylesterase inhibitor family protein, putative, expressed
Os.52527.1.S1_s_at	oligopeptide transporter, putative, expressed
OsAffx.24612.1.S1_at	heavy metal associated domain containing protein, expressed
Os.27431.1.A1_at	TKL_IRAK_DUF26-lc.12 - DUF26 kinases have homology to DUF26 containing loci, express
Os.4974.1.S1_x_at	nodulin MtN3 family protein, putative, expressed
Os.7017.1.S1_at	OsWAK8 - OsWAK receptor-like protein kinase, expressed
Os.53355.1.S1_s_at	ACT domain containing protein, putative, expressed
Os.12253.1.S1_at	anthocyanin 3-O-beta-glucosyltransferase, putative, expressed
Os.49796.1.S1_at	wall-associated kinase 1, putative, expressed
Os.28514.1.S1_at	calmodulin binding protein, putative, expressed
Os.52189.1.S1_at	cadmium tolerance factor, putative, expressed
Os.5601.1.S1_at	FAD dependent oxidoreductase domain containing protein, expressed
OsAffx.6331.1.S1_at	receptor-like protein kinase precursor, putative, expressed
Os.10765.1.S1_at	transferase family protein, putative, expressed
Os.17722.1.S1_at	peroxidase precursor, putative, expressed
Os.49827.1.S1_at	receptor-like protein kinase 2 precursor, putative, expressed
Os.17437.1.S1_at	glucosyltransferase, putative, expressed

Os.35642.2.S1_x_at	OsSub8 - Putative Subtilisin homologue, expressed
Os.12082.1.S1_at	heavy metal associated domain containing protein, expressed
Os.11837.1.S1_at	oxidoreductase, aldo/keto reductase family protein, putative, expressed
Os.4995.1.S1_at	expressed protein
Os.46107.1.S2_a_at	retrotransposon protein, putative, unclassified, expressed
Os.53707.1.S1_x_at	proton-dependent oligopeptide transport, putative, expressed
OsAffx.32166.1548.S1_	retrotransposon protein, putative, unclassified, expressed
OsAffx.27093.1.S1_at	cytokinin dehydrogenase precursor, putative, expressed
Os.24551.4.S1_at	phytosulfokine receptor precursor, putative, expressed
Os.54121.1.S1_s_at	expressed protein
Os.27617.1.S1_at	expressed protein
Os.10162.1.S1_at	PRAS-rich protein, putative, expressed
Os.9101.1.S1_at	glutathione S-transferase, putative, expressed
Os.9462.2.S1_at	DEAD-box ATP-dependent RNA helicase, putative, expressed
Os.52435.1.S1_at	phosphate transporter 1, putative, expressed
Os.49817.1.S1_at	Leucine Rich Repeat family protein, expressed
Os.19815.1.S1_at	CAMK_KIN1/SNF1/Nim1_like.17 - CAMK includes calcium/calmodulin depedent protein k
Os.42024.1.S1_at	ctr copper transporter family protein, putative, expressed
OsAffx.2784.1.S1_s_at	ankyrin, putative, expressed
Os.46823.1.S1_at	phospho-2-dehydro-3-deoxyheptonate aldolase, chloroplast precursor, putative, express
Os.52185.1.A1_at	GLTP domain containing protein, putative, expressed
Os.49109.1.S2_at	phosphatidylinositol transfer, putative, expressed
OsAffx.15538.1.S1_at	Os6bglu24 - beta-glucosidase homologue, similar to G. max isohydroxyurate hydrolase, e
Os.27789.1.A1_at	peroxidase precursor, putative, expressed
OsAffx.26304.1.S1_s_a	expressed protein
Os.6767.1.S1_at	retrotransposon protein, putative, SINE subclass, expressed
OsAffx.19428.1.S1_s_a	thaumatin family domain containing protein, expressed
Os.10597.1.S1_at	anthocyanidin 5,3-O-glucosyltransferase, putative, expressed
Os.49855.1.S1_at	tyrosine protein kinase domain containing protein, putative, expressed
Os.52157.1.S1_x_at	OsFBO10 - F-box and other domain containing protein, expressed
Os.11218.1.S1_at	peroxiredoxin, putative, expressed
Os.51085.1.S1_at	transmembrane amino acid transporter protein, putative, expressed
Os.8947.1.S1_at	expressed protein
Os.37996.1.S1_at	EF hand family protein, putative, expressed
Os.5842.1.S1_at	copine, putative, expressed
Os.47343.2.S1_x_at	ACT domain containing protein, expressed
Os.16964.1.S1_x_at	expressed protein
Os.49538.1.S1_at	expressed protein
Os.49840.1.S1_at	phytosulfokine receptor precursor, putative, expressed
Os.21442.1.S1_at	CPuORF1 - conserved peptide uORF-containing transcript, expressed
Os.27165.1.A1_at	OsFBX336 - F-box domain containing protein, expressed

ed

sed

sed

‹inases, expressed

ed

›xpressed

Probe Sets	Putative Function
Os.51718.1.S1_at	dehydrin, putative, expressed
Os.7051.1.S1_at	MYB family transcription factor, putative, expressed
Os.11534.1.S1_at	zinc finger CCCH type family protein, putative, expressed
Os.31975.1.S1_at	zinc finger CCCH type family protein, putative, expressed
Os.31975.1.S1_x_at	zinc finger CCCH type family protein, putative, expressed
Os.2694.1.S1_at	caleosin related protein, putative, expressed
Os.48875.1.S1_at	glutamine synthetase, catalytic domain containing protein, expressed
Os.6901.1.S1_at	expressed protein
Os.25409.1.S1_x_at	retrotransposon protein, putative, Ty3-gypsy subclass, expressed
Os.30528.1.S1_at	expressed protein
Os.57519.1.A1_at	early light-induced protein, chloroplast precursor, putative, expressed
Os.30657.1.S1_at	WRKY77, expressed
Os.39973.1.S1_s_at	actin-depolymerizing factor, putative, expressed
OsAffx.22469.1.S1_x	erythrocyte binding protein 3, putative, expressed
Os.38110.1.S1_at	trehalase precursor, putative, expressed
Os.51866.1.S1_at	disease resistance protein RGA3, putative, expressed
Os.29084.1.S1_at	zinc finger CCCH type family protein, putative, expressed
Os.411.2.S1_x_at	bZIP transcription factor domain containing protein, expressed
Os.11915.1.S1_at	annexin, putative, expressed
OsAffx.3504.1.S1_at	transporter family protein, putative, expressed
Os.11995.1.S1_at	urease accessory protein F, putative, expressed
Os.9913.1.S1_at	wound induced protein, putative, expressed
Os.40007.1.S1_x_at	retrotransposon protein, putative, Ty3-gypsy subclass, expressed
Os.27232.1.S1_at	plant-specific domain TIGR01615 family protein, expressed
OsAffx.21798.1.S1_at	
Os.23087.1.S1_at	Myb transcription factor, putative, expressed
OsAffx.3569.1.S1_at	LTPL118 - Protease inhibitor/seed storage/LTP family protein precursor, expressed
Os.37876.1.S1_at	expressed protein
OsAffx.3569.1.S1_x_	LTPL118 - Protease inhibitor/seed storage/LTP family protein precursor, expressed
Os.4700.1.S1_at	transporter, monovalent cation:proton antiporter-2 family, putative, expressed
Os.3415.1.S1_s_at	CHIT14 - Chitinase family protein precursor, expressed
Os.5277.1.S1_at	bZIP transcription factor domain containing protein, expressed
Os.13968.2.S1_a_at	OsRCI2-6 - Hydrophobic protein LTI6B, expressed
OsAffx.16365.1.S1_a	anaphase-promoting complex subunit 11, putative, expressed
Os.19740.1.S2_at	retrotransposon protein, putative, Ty3-gypsy subclass, expressed
Os.4530.1.S1_at	splicing factor U2AF, putative, expressed
Os.623.3.S1_x_at	Myb transcription factor, putative, expressed
Os.11265.1.S1_at	haloacid dehalogenase-like hydrolase family protein, putative, expressed
Os.38157.1.S1_s_at	ABC transporter, ATP-binding protein, putative, expressed
Os.30433.1.S1_at	RING-H2 finger protein, putative, expressed
Os.10131.1.S1_a_at	NAD dependent epimerase/dehydratase family protein, putative, expressed
Os.57475.1.S1_x_at	glyceraldehyde-3-phosphate dehydrogenase, putative, expressed
Os.28098.2.A1_at	beta-amylase, putative, expressed

Probe Sets	Putative Function
Os.27703.1.S1_a_at	microtubule-associated protein, putative, expressed
Os.27703.1.S1_s_at	microtubule-associated protein, putative, expressed
Os.9660.1.S1_at	integral membrane protein, putative, expressed
Os.57103.1.S1_at	terpene synthase, putative, expressed
Os.27247.1.S1_at	GDSL-like lipase/acylhydrolase, putative, expressed
Os.11469.1.S1_at	oxidoreductase, aldo/keto reductase family protein, putative, expressed
Os.7674.1.S1_at	cytochrome P450, putative, expressed
Os.7756.2.S1_x_at	invertase/pectin methylesterase inhibitor family protein, putative, expressed
Os.50455.1.S1_at	phospholipase D, putative, expressed
OsAffx.6372.1.S1_s_at	integral membrane protein, putative, expressed
Os.14938.1.S1_at	methyltransferase, putative, expressed
Os.17961.1.S1_a_at	fasciclin domain containing protein, expressed
Os.5549.1.S1_at	NAC domain-containing protein 67, putative, expressed
Os.10596.1.S1_at	cytochrome P450, putative, expressed
Os.38992.1.A1_x_at	expressed protein
Os.9015.1.S1_at	xylem cysteine proteinase 2 precursor, putative, expressed
Os.11580.1.S1_at	auxin-induced protein 5NG4, putative, expressed
Os.38245.1.S1_at	GDSL-like lipase/acylhydrolase, putative, expressed
Os.4164.2.S1_a_at	homeobox protein knotted-1, putative, expressed
Os.22627.1.S1_at	fasciclin domain containing protein, expressed
Os.53237.1.S1_at	auxin-induced protein 5NG4, putative, expressed
Os.38017.1.S1_s_at	annexin, putative, expressed
OsAffx.12986.1.S1_at	nodulin MtN3 family protein, putative, expressed
Os.30032.1.S1_at	retrotransposon protein, putative, unclassified, expressed
Os.28110.4.S1_x_at	N/A

Probe Sets	Putative Function
Os.30032.1.S1_at	transposon protein, putative, unclassified, expressed
Os.38812.1.S1_at	expressed protein
Os.11491.1.S1_at	expressed protein
OsAffx.12986.1.S1_at	protein phosphatase 2C, putative, expressed
Os.9216.1.S1_at	mitochondrial import inner membrane translocase subunit Tim17, putative, expressed
Os.54698.1.S1_at	late embryogenesis abundant protein, group 3, putative, expressed
Os.12551.1.S1_s_at	protein phosphatase 2C, putative, expressed
Os.6274.1.S1_at	dehydrin, putative, expressed
Os.54410.1.S1_at	retrotransposon protein, putative, unclassified, expressed
Os.11118.1.S1_at	helix-loop-helix DNA-binding domain containing protein, expressed
Os.34372.1.S1_at	inhibitor I family protein, putative, expressed
Os.39552.1.A1_s_at	LTPL153 - Protease inhibitor/seed storage/LTP family protein precursor, expressed
Os.48053.1.A1_at	dehydrogenase E1 component domain containing protein, expressed
Os.10590.1.S1_s_at	expressed protein
Os.56875.1.S1_at	phosphoglycerate kinase protein, putative, expressed
Os.38992.1.A1_x_at	expressed protein
OsAffx.6171.1.S1_at	nodulin MtN3 family protein, putative, expressed
OsAffx.31140.1.S1_at	retrotransposon protein, putative, unclassified, expressed
Os.51491.1.S1_at	N/A
Os.12430.1.S1_at	N/A

Probe Sets	Putative Function
Os.27956.1.S1_at	late embryogenesis abundant protein D-34, putative, expressed
Os.15692.1.S1_at	cytochrome P450, putative, expressed
Os.51758.2.S1_at	expressed protein
Os.2250.1.S1_a_at	DUF1264 domain containing protein, putative, expressed
Os.51092.1.S1_at	expressed protein
Os.13972.1.S1_at	peroxiredoxin, putative, expressed
Os.5338.1.S1_at	universal stress protein domain containing protein, putative, expressed
Os.25449.1.S1_at	integral membrane transporter family protein, putative, expressed
Os.25613.1.A1_at	CCT motif family protein, expressed
Os.27797.1.A1_at	IQ calmodulin-binding motif family protein, putative, expressed
Os.12821.1.S1_at	Core histone H2A/H2B/H3/H4 domain containing protein, putative, expressed
Os.36995.1.S1_at	peroxidase precursor, putative, expressed
Os.55527.1.S1_at	heavy metal-associated domain containing protein, expressed
Os.4638.1.S1_at	RALFL14 - Rapid ALkalinization Factor RALF family protein precursor, expressed
Os.53632.1.S1_at	OsRhmbd12 - Putative Rhomboid homologue, expressed
Os.55303.1.S1_at	core histone H2A/H2B/H3/H4, putative, expressed
OsAffx.31856.1.S1_at	OsSub63 - Putative Subtilisin homologue, expressed
Os.52820.1.S1_at	white-brown complex homolog protein, putative, expressed
Os.2362.1.S1_a_at	homeobox associated leucine zipper, putative, expressed
Os.9615.1.S1_at	laccase precursor protein, putative, expressed
Os.23262.1.A1_s_at	OsSub63 - Putative Subtilisin homologue, expressed
Os.24995.1.S1_a_at	lipase precursor, putative, expressed
Os.55266.1.S1_at	expressed protein
Os.3426.1.S1_at	nuclear transcription factor Y subunit, putative, expressed
OsAffx.25073.1.S1_x_a	expressed protein
Os.2367.1.S1_at	expansin precursor, putative, expressed
Os.12995.1.S1_at	expressed protein
Os.56880.1.S1_at	bZIP transcription factor domain containing protein, expressed
Os.51731.1.S1_at	ankyrin repeat domain-containing protein 44, putative, expressed
Os.48260.1.S1_at	expressed protein
Os.32292.1.S1_at	peroxidase precursor, putative, expressed
Os.51696.1.S1_at	transporter, major facilitator family, putative, expressed
Os.48013.1.A1_at	caleosin related protein, putative, expressed
Os.9995.1.S1_at	expressed protein
Os.15918.1.S1_x_at	P21-Rho-binding domain containing protein, putative, expressed
OsAffx.17158.1.S1_at	expressed protein
Os.22711.1.A1_at	DUF623 domain containing protein, expressed
OsAffx.11535.1.S1_at	glutathione S-transferase, putative, expressed
Os.57511.1.A1_at	CCT motif family protein, expressed
OsAffx.18633.1.S1_s_a	expressed protein
Os.209.1.S1_at	embryonic protein DC-8, putative, expressed
Os.11602.1.S1_at	expressed protein
Os.8561.1.S1_at	bZIP transcription factor domain containing protein, expressed
Os.8253.1.S1_at	expressed protein
Os.30754.1.S1_at	expressed protein
Os.47750.1.A1_at	Cupin domain containing protein, expressed

Os.48981.1.S1_at	GASR9 - Gibberellin-regulated GASA/GAST/Snakin family protein precursor, expr
Os.26441.1.S1_s_at	Os11bglu36 SFR2 homologue, expressed
OsAffx.26343.1.S1_x_a	expressed protein
Os.55837.1.S1_x_at	AP2 domain containing protein, expressed
Os.7478.1.S1_at	dehydrodolichyl diphosphate synthase, putative, expressed
Os.27592.1.A1_at	retrotransposon protein, putative, unclassified, expressed
Os.7665.1.S1_at	lipase, putative, expressed
Os.46492.1.S1_at	OsFBX357 - F-box domain containing protein, expressed
Os.51711.1.S1_at	retrotransposon, putative, centromere-specific, expressed
Os.15219.1.S1_at	peptidyl-prolyl cis-trans isomerase CYP40, putative, expressed
Os.4755.1.S1_at	Core histone H2A/H2B/H3/H4 domain containing protein, putative, expressed
Os.8821.1.S1_at	NADP-dependent oxidoreductase, putative, expressed
Os.12092.1.S1_at	nodulin, putative, expressed
Os.12429.1.S1_at	erythrocyte binding protein 3, putative, expressed
Os.57345.1.S1_at	BTBN3 - Bric-a-Brac, Tramtrack, Broad Complex BTB domain with non-phototrop
Os.10197.1.S1_at	permease domain containing protein, putative, expressed
OsAffx.20051.1.S1_at	expressed protein
Os.7044.1.S1_at	expressed protein
Os.56140.1.S1_x_at	expressed protein
Os.7370.1.S1_at	cytochrome P450, putative, expressed
OsAffx.14406.1.S1_at	RALFL18 - Rapid ALkalinization Factor RALF family protein precursor, expressed
OsAffx.30275.1.S1_at	expressed protein
OsAffx.25245.1.S1_at	hydrolase protein, putative, expressed
Os.11943.2.S1_at	OsGrx_S12 - glutaredoxin subgroup I, expressed
Os.17334.1.S1_s_at	transcription factor, putative, expressed
Os.49185.1.S1_at	peroxisomal biogenesis factor 11, putative, expressed
Os.18314.1.S1_at	hypoxia-responsive family protein, putative, expressed
OsAffx.10965.1.S1_at	TMS membrane protein/tumour differentially expressed protein, putative, expre
OsAffx.24777.1.S1_at	expressed protein
OsAffx.17329.1.S1_s_a	expressed protein
Os.6080.1.S1_at	aminomethyltransferase, putative, expressed
Os.45924.1.S1_at	expressed protein
OsAffx.28932.1.S1_at	rhythmically expressed gene 2 protein, putative, expressed
Os.51772.1.S1_at	expressed protein
Os.55461.1.S1_at	expressed protein
Os.53724.1.S1_at	RALFL24 - Rapid ALkalinization Factor RALF family protein precursor, expressed
Os.14560.1.S1_at	B-box zinc finger family protein, putative, expressed
Os.45924.1.S1_x_at	rhythmically expressed gene 2 protein, putative, expressed
Os.37687.1.A1_at	expressed protein
OsAffx.3968.1.S1_at	expressed protein
Os.8763.1.S1_at	mRNA-decapping enzyme, putative, expressed
Os.57327.1.S1_at	N/A
Os.50179.1.S1_at	N/A
Os.11147.1.S1_at	N/A
Os.46758.1.S1_at	N/A
Os.48873.1.S1_at	N/A
Os.9152.1.S1_at	N/A

Os.5357.1.S1_at	N/A

essed

ɔic hypocotyl 3 NPH3 domain, expressed

ɜssed

Probe Sets	Putative Function
Os.7779.1.S1_x_at	auxin-induced protein 5NG4, putative, expressed
Os.25153.1.S1_at	cytochrome P450, putative, expressed
Os.51127.2.S1_x_at	cytokinin-O-glucosyltransferase 2, putative, expressed
OsAffx.4527.1.S1_s_at	dienelactone hydrolase family protein, expressed
Os.22577.2.S1_x_at	DUF260 domain containing protein, putative, expressed
Os.9929.1.S1_at	expressed protein
Os.56237.1.S1_at	expressed protein
Os.15679.1.S1_s_at	ferric reductase, putative, expressed
Os.12096.4.S1_s_at	ferritin-1, chloroplast precursor, putative, expressed
Os.12096.3.S1_a_at	ferritin-1, chloroplast precursor, putative, expressed
Os.12096.3.S1_x_at	ferritin-1, chloroplast precursor, putative, expressed
OsAffx.20681.1.S1_at	integral membrane protein DUF6 containing protein, expressed
Os.12979.3.S1_x_at	IQ calmodulin-binding motif domain containing protein, expressed
Os.16897.1.S1_at	lipase class 3 family protein, putative, expressed
Os.28026.1.S1_at	metal cation transporter, putative, expressed
Os.12410.1.S1_a_at	metallothionein, putative, expressed
Os.12410.1.S1_x_at	metallothionein, putative, expressed
Os.12410.3.S1_s_at	metallothionein, putative, expressed
Os.12410.3.S1_x_at	metallothionein, putative, expressed
OsAffx.27267.1.S1_at	Myb transcription factor, putative, expressed
Os.12701.1.S1_at	oryzain alpha chain precursor, putative, expressed
Os.15894.1.A1_a_at	peroxidase precursor, putative, expressed
Os.52493.1.A1_s_at	POEI46 - Pollen Ole e I allergen and extensin family protein precursor, expressed
Os.11789.1.S1_at	possible lysine decarboxylase domain containing protein, expressed
OsAffx.29248.3.S1_s_a	PSF3 - Putative GINS complex subunit, expressed
Os.5404.3.S1_a_at	respiratory burst oxidase, putative, expressed
Os.14271.1.S1_at	RNA recognition motif containing protein, putative, expressed
Os.51227.1.S1_s_at	terpene synthase, putative, expressed
Os.51227.1.S1_x_at	terpene synthase, putative, expressed
AFFX-r2-Bs-dap-5_at	N/A

Probe Sets	Putative Function
Os.40001.1.A1_at	retrotransposon protein, putative, Ty3-gypsy subclass, expressed
Os.7487.1.S1_at	reticulon domain containing protein, putative, expressed
Os.27963.1.A1_at	abscisic stress-ripening, putative, expressed
OsAffx.31976.1.S1_s_at	expressed protein
Os.21260.1.S1_at	expressed protein
Os.27497.1.S1_at	transposon protein, putative, CACTA, En/Spm sub-class, expressed
Os.11831.1.S1_at	SAM dependent carboxyl methyltransferase, putative, expressed
Os.12761.1.S1_at	thaumatin family domain containing protein, expressed
Os.7751.1.S1_at	basic helix-loop-helix, putative, expressed
Os.4281.1.S1_x_at	retinol dehydrogenase, putative, expressed
Os.39933.1.S1_x_at	expressed protein
Os.16214.1.S1_at	expressed protein
Os.1443.1.S1_a_at	basic helix-loop-helix, putative, expressed
AFFX-Os_28SrRNA_at	expressed protein
OsAffx.32221.1.A1_s_at	NADPH-dependent oxidoreductase, putative, expressed
Os.53458.1.S1_at	O-methyltransferase, putative, expressed
Os.39995.1.S1_x_at	retrotransposon protein, putative, Ty3-gypsy subclass, expressed
OsAffx.32200.1.A1_x_at	chloroplast 50S ribosomal protein L23, putative
Os.56918.1.S1_at	expressed protein
Os.9859.1.S1_at	fibronectin type 3 and ankyrin repeat domains 1 protein, putative, expressed
OsAffx.23104.1.S1_at	cation efflux family protein, putative, expressed
Os.52678.1.S1_at	osFTL6 FT-Like6 homologous to Flowering Locus T gene; contains Pfam profile P[illegible]
Os.35681.1.S1_at	HSF-type DNA-binding domain containing protein, expressed
Os.12261.1.S1_a_at	CAMK_KIN1/SNF1/Nim1_like.2 - CAMK includes calcium/calmodulin depedent pr[illegible]
Os.28435.4.S1_x_at	flavonol synthase/flavanone 3-hydroxylase, putative, expressed
OsAffx.32196.1.A1_at	chloroplast 50S ribosomal protein L22, putative, expressed
Os.49746.1.S1_at	MYB family transcription factor, putative, expressed
Os.57484.1.S1_x_at	HMG1/2, putative, expressed
Os.53320.1.S1_s_at	transposon protein, putative, Mutator sub-class, expressed
Os.53320.2.S1_s_at	transposon protein, putative, Mutator sub-class, expressed
Os.15580.1.S1_at	expressed protein
Os.28435.4.S1_at	flavonol synthase/flavanone 3-hydroxylase, putative, expressed
Os.11638.1.S1_at	MYB family transcription factor, putative, expressed
Os.10333.1.S1_at	MYB family transcription factor, putative, expressed
OsAffx.15233.1.S1_x_at	expressed protein
OsAffx.30670.1.S1_at	cytochrome P450, putative, expressed
OsAffx.11956.1.S1_at	myb-like DNA-binding domain containing protein, putative, expressed
Os.19547.1.S1_at	transferase family protein, putative, expressed
OsAffx.27338.1.S1_at	expressed protein
Os.7457.1.S1_a_at	phosphoribosyl transferase, putative, expressed
OsAffx.31976.1.S1_at	expressed protein
Os.5318.1.S1_a_at	expressed protein
OsAffx.17883.1.S1_at	double-stranded RNA binding motif containing protein, expressed
OsAffx.23031.1.S1_s_at	expressed protein
Os.7893.1.S1_at	HMG1/2, putative, expressed
OsAffx.32257.1.A1_at	photosystem I assembly protein ycf4, putative, expressed

Os.49632.1.S1_a_at	double-stranded RNA binding motif containing protein, expressed
OsAffx.1590.1.S1_x_at	photosystem I iron-sulfur center, putative, expressed
OsAffx.24769.1.S1_s_at	MYB family transcription factor, putative, expressed
Os.46647.1.S1_at	remorin C-terminal domain containing protein, putative, expressed
OsAffx.32313.1.A1_at	chloroplast 50S ribosomal protein L20, putative, expressed
Os.51359.1.S1_at	zinc finger, C3HC4 type domain containing protein, expressed
Os.8439.1.S1_a_at	expressed protein
Os.54463.1.S1_at	expressed protein
Os.17446.1.S1_at	OsFBX148 - F-box domain containing protein, expressed
Os.27525.1.A1_at	cytochrome P450, putative, expressed
Os.40030.1.S1_s_at	SHR5-receptor-like kinase, putative, expressed
OsAffx.26679.1.S1_at	FYVE zinc finger domain containing protein, expressed
OsAffx.15233.1.S1_at	N/A
OsAffx.32310.1.A1_at	N/A
OsAffx.32309.1.A1_at	N/A
OsAffx.8351.1.S1_at	N/A

F01161: Phosphatidylethanolamine-binding protein, expressed

otein kinases, expressed

Probe Sets	Putative Function
Os.46849.1.S1_at	ZIM domain containing protein, putative, expressed
Os.9311.1.S1_at	nicotianamine synthase, putative, expressed
Os.9923.1.S1_s_at	ZIM domain containing protein, putative, expressed
Os.12381.1.S1_s_at	dirigent, putative, expressed
Os.12381.1.S1_x_at	dirigent, putative, expressed
Os.45902.1.A1_x_at	nmrA-like family domain containing protein, expressed
Os.28216.1.S1_a_at	chlorophyll A-B binding protein, putative, expressed
Os.37295.1.S1_at	chalcone--flavonone isomerase, putative, expressed
Os.15849.1.S1_s_at	AP2 domain containing protein, expressed
Os.7116.1.S1_at	helix-loop-helix DNA-binding domain containing protein, expressed
Os.28216.3.S1_x_at	chlorophyll A-B binding protein, putative, expressed
Os.49763.2.S1_at	dirigent, putative, expressed
Os.11183.1.S1_s_at	protein kinase domain containing protein, expressed
Os.40417.1.A1_at	harpin-induced protein 1 domain containing protein, expressed
Os.11183.1.S1_at	protein kinase domain containing protein, expressed
Os.5697.1.S1_at	DUF584 domain containing protein, putative, expressed
Os.14313.2.S1_s_at	protein kinase family protein, putative, expressed
Os.2210.1.S1_at	Cupin domain containing protein, expressed
Os.6965.1.S1_at	EF hand family protein, putative, expressed
OsAffx.5283.1.S1_at	expressed protein
Os.27483.1.S1_at	cupin domain containing protein, expressed
Os.4251.1.S1_at	peroxiredoxin, putative, expressed
Os.52451.1.A1_at	AP2 domain containing protein, expressed
Os.2436.1.S1_at	S-domain receptor-like protein kinase, putative, expressed
Os.12032.1.S1_at	WRKY71, expressed
OsAffx.2403.1.S1_at	expressed protein
Os.46160.2.S1_at	inorganic phosphate transporter, putative, expressed
Os.10401.1.S1_s_at	nodulin MtN3 family protein, putative, expressed
Os.47369.1.A1_at	expressed protein
Os.21870.1.S1_at	protein kinase domain containing protein, expressed
Os.51809.1.S1_at	serine/threonine-protein kinase BRI1-like 1 precursor, putative, expressed
Os.38169.1.S1_a_at	expressed protein
Os.52266.1.S1_at	expressed protein
Os.27177.1.S1_at	avr9/Cf-9 rapidly elicited protein, putative, expressed
Os.32108.1.S1_s_at	pectinesterase, putative, expressed
Os.5154.1.S1_at	COBRA-like protein 7 precursor, putative, expressed
Os.6354.1.S1_at	chalcone--flavonone isomerase, putative, expressed
Os.50961.1.S1_at	oxidoreductase, putative, expressed
Os.46776.1.S1_s_at	cytochrome P450, putative, expressed
Os.37893.1.S1_at	phenylalanine ammonia-lyase, putative, expressed
Os.8961.1.S1_s_at	WRKY7, expressed
Os.7743.1.S1_at	plastocyanin-like domain containing protein, putative, expressed
Os.37621.3.S1_at	expressed protein
OsAffx.4833.1.S1_x_at	U-box protein CMPG1, putative, expressed
Os.11843.1.S1_at	GRAS family transcription factor domain containing protein, expressed
Os.27407.1.A1_at	domain of unknown function DUF966 domain containing protein, expressed

Os.27186.1.S1_at	expressed protein
Os.49583.1.S1_at	expressed protein
Os.4801.1.S1_x_at	alliin lyase precursor, putative, expressed
Os.9775.1.S1_at	glutathione S-transferase, putative, expressed
Os.16903.1.A1_at	relA-SpoT like protein RSH4, putative, expressed
Os.53817.1.S1_at	exo70 exocyst complex subunit family protein, putative, expressed
OsAffx.21616.1.S1_s_at	ethylene-responsive transcription factor 2, putative, expressed
Os.46160.2.S1_x_at	inorganic phosphate transporter, putative, expressed
OsAffx.17366.1.S1_at	AP2 domain containing protein, expressed
Os.14313.1.S1_s_at	protein kinase family protein, putative, expressed
Os.7521.1.S1_at	expressed protein
OsAffx.2070.2.S1_at	transposon protein, putative, CACTA, En/Spm sub-class, expressed
Os.26853.1.A1_at	cyclin-T1-1, putative, expressed
Os.14366.1.S1_at	phosphate-induced protein 1 conserved region domain containing protein, ex
Os.46546.1.S1_at	peptide transporter PTR2, putative, expressed
Os.8139.1.S1_at	peroxidase precursor, putative, expressed
Os.28110.3.S1_at	transposon protein, putative, unclassified, expressed
Os.13543.1.S1_at	heavy metal-associated domain containing protein, expressed
OsAffx.10944.1.S1_at	expressed protein
Os.9976.1.S1_at	expressed protein
Os.40342.1.A1_s_at	cadmium tolerance factor, putative, expressed
Os.34471.1.S1_at	no apical meristem protein, putative, expressed
Os.30998.1.S1_at	plant-specific domain TIGR01589 family protein, expressed
Os.25146.1.A1_at	methyladenine glycosylase, putative, expressed
Os.7075.1.S1_at	phospho-2-dehydro-3-deoxyheptonate aldolase, chloroplast precursor, putati
Os.322.1.S1_at	ubiquitin-conjugating enzyme, putative, expressed
Os.17325.1.S1_at	hydrolase, alpha/beta fold family protein, putative, expressed
Os.5500.1.S1_s_at	fasciclin-like arabinogalactan protein 8 precursor, putative, expressed
Os.27159.1.S1_at	metal cation transporter, putative, expressed
OsAffx.27605.1.S1_s_at	expressed protein
Os.40018.1.S1_at	HSF-type DNA-binding domain containing protein, expressed
Os.16422.1.S1_s_at	CCT/B-box zinc finger protein, putative, expressed
Os.11474.1.S1_at	OsFBK10 - F-box domain and kelch repeat containing protein, expressed
OsAffx.14324.1.S1_at	MATE efflux family protein, putative, expressed
Os.22590.1.A1_at	RNA recognition motif containing protein, putative, expressed
Os.27484.1.S1_at	protein kinase, putative, expressed
Os.18851.2.A1_at	armadillo/beta-catenin repeat family protein, putative, expressed
Os.15138.1.S1_at	Myb transcription factor, putative, expressed
Os.30059.1.S1_at	expressed protein
Os.13623.1.S1_at	aspartic proteinase, putative, expressed
Os.46725.1.S1_at	expressed protein
Os.53052.1.S1_at	exo70 exocyst complex subunit domain containing protein, expressed
Os.57563.1.S1_s_at	NBS-LRR disease resistance protein, putative, expressed
Os.12710.1.S1_at	zinc-finger protein, putative, expressed
Os.14199.1.S1_at	inactive receptor kinase At1g27190 precursor, putative, expressed
Os.7012.1.S1_at	armadillo repeat-containing protein, putative, expressed
Os.52379.1.S1_s_at	tetratricopeptide repeat domain containing protein, expressed

Os.16401.1.S1_at	OsCML27 - Calmodulin-related calcium sensor protein, expressed
Os.28427.1.S2_a_at	BURP domain containing protein, expressed
Os.37822.3.A1_s_at	KIP1, putative, expressed
Os.28531.1.S1_at	glycosyl hydrolase, putative, expressed
Os.8375.1.S1_at	nucleoside transporter, putative, expressed
Os.25606.1.S1_at	WRKY76, expressed
Os.9829.1.S1_at	OsGrx_S2 - glutaredoxin subgroup III, expressed
Os.11766.1.S1_at	chitin-inducible gibberellin-responsive protein, putative, expressed
Os.55247.1.S1_at	expressed protein
Os.3388.2.S1_a_at	MYB family transcription factor, putative, expressed
Os.7632.1.S1_at	TKL_IRAK_DUF26-la.1 - DUF26 kinases have homology to DUF26 containing lo
Os.38299.1.S1_at	protein phosphatase 2C, putative, expressed
Os.12937.1.S1_at	expressed protein
Os.37093.1.S1_at	cytochrome P450, putative, expressed
Os.45916.1.S1_s_at	MFS18 protein precursor, putative, expressed
Os.21710.1.S1_at	leucine-rich repeat family protein, putative, expressed
Os.30044.1.S1_a_at	helix-loop-helix DNA-binding domain containing protein, expressed
Os.22341.1.S1_at	RALFL6 - Rapid ALkalinization Factor RALF family protein precursor, expressed
Os.30473.1.S1_at	gibberellin 2-beta-dioxygenase, putative, expressed
Os.33605.2.S1_x_at	zinc finger, C3HC4 type domain containing protein, expressed
Os.55647.1.A1_at	expressed protein
Os.7335.1.S1_at	riboflavin biosynthesis protein ribAB, chloroplast precursor, putative, expresse
OsAffx.28760.2.S1_at	hypothetical protein
Os.54551.1.S1_at	endoglucanase, putative, expressed
Os.4683.2.S1_at	expressed protein
Os.27598.1.S1_at	expressed protein
OsAffx.19579.1.S1_at	protein phosphatase 2C, putative, expressed
Os.4857.1.S1_at	CESA5 - cellulose synthase, expressed
Os.51385.1.S1_at	expressed protein
Os.9199.1.S1_at	senescence-associated protein, putative, expressed
Os.21394.1.S1_at	SNARE domain containing protein, putative, expressed
Os.24863.1.A1_at	pleiotropic drug resistance protein, putative, expressed
Os.52260.1.S1_at	syntaxin, putative, expressed
Os.9708.1.S1_at	reticuline oxidase-like protein precursor, putative, expressed
Os.50300.1.S1_s_at	aspartic proteinase nepenthesin-2 precursor, putative, expressed
Os.23649.1.S1_a_at	expressed protein
Os.24364.1.A1_at	dehydration response related protein, putative, expressed
OsAffx.22999.1.S1_at	retrotransposon protein, putative, unclassified, expressed
Os.46819.1.S1_at	MBTB48 - Bric-a-Brac, Tramtrack, Broad Complex BTB domain with Meprin an
Os.20892.1.S1_at	alpha-DOX2, putative, expressed
Os.38169.1.S1_at	sodium/calcium exchanger 1 precursor, putative, expressed
OsAffx.15770.1.S1_at	expressed protein
OsAffx.24328.1.S1_at	lectin-like receptor kinase, putative, expressed
Os.18513.1.S1_at	peptide-N4-asparagine amidase A, putative, expressed
Os.18434.1.S1_at	zinc finger family protein, putative, expressed
OsAffx.12379.1.S1_at	oxygen evolving enhancer protein 3, identical, putative, expressed
Os.47958.1.A1_x_at	NADH-ubiquinone oxidoreductase, mitochondrial precursor, putative, express

Os.6656.1.S1_at	salt stress root protein RS1, putative, expressed
Os.47363.1.A1_at	NB-ARC domain containing protein, expressed
Os.32267.1.S1_at	TKL_IRAK_CrRLK1L-1.5 - The CrRLK1L-1 subfamily has homology to the CrRLK1
Os.14667.2.S1_at	membrane associated DUF588 domain containing protein, putative, expresse
Os.16248.1.S1_at	GATA zinc finger domain containing protein, expressed
Os.8593.1.S1_at	organic cation transporter protein, putative, expressed
OsAffx.28093.1.S1_at	DOMON domain containing protein, expressed
Os.43043.1.S1_at	expressed protein
Os.10125.1.S1_a_at	fatty acid desaturase, putative, expressed
OsAffx.4763.1.S1_at	transporter, major facilitator family, putative, expressed
Os.45902.1.A1_at	N/A
Os.54417.1.S1_at	N/A
Os.54317.1.S1_at	N/A

pressed

ve, expressed

ci, expressed

ed

d TRAF Homology MATH domain, expressed

sed

.L homolog, expressed
d

Probe Sets	Putative Function
Os.32943.1.S1_at	AAA-type ATPase family protein, putative, expressed
Os.6372.1.S1_at	cupin domain containing protein, expressed
OsAffx.19357.1.S1_at	DUF581 domain containing protein, expressed
Os.3417.1.S1_at	esterase, putative, expressed
Os.16163.1.S1_at	expressed protein
Os.28200.1.S1_x_at	expressed protein
Os.55332.1.S1_at	expressed protein
Os.4867.1.S1_at	glycosyl hydrolase, putative, expressed
Os.12498.1.S1_at	helix-loop-helix DNA-binding domain containing protein, expressed
Os.49639.1.S1_at	late embryogenesis abundant group 1, putative, expressed
Os.12191.1.S1_s_at	non-symbiotic hemoglobin 2, putative, expressed
Os.38099.1.S1_at	OsMan04 - Endo-Beta-Mannanase, expressed
Os.47871.1.S1_at	transposon protein, putative, CACTA, En/Spm sub-class, expressed
Os.23770.1.A1_s_at	zinc finger family protein, putative, expressed
Os.15809.1.S1_at	N/A

Probe Sets	Putative Function
Os.4377.1.S1_at	AMP-binding domain containing protein, expressed
Os.17746.1.S1_s_at	anthocyanidin 3-O-glucosyltransferase, putative, expressed
Os.14921.1.S1_at	app1, putative, expressed
Os.15854.1.S1_at	beta-amylase, putative, expressed
OsAffx.28771.1.S1_a	beta-amylase, putative, expressed
Os.18724.1.S1_at	CESA4 - cellulose synthase, expressed
Os.12416.1.S1_at	chalcone synthase, putative, expressed
Os.12851.1.S1_at	Cupin domain containing protein, expressed
Os.53814.1.S1_at	dehydrogenase, putative, expressed
Os.55394.1.S1_at	expressed protein
Os.45928.1.S1_at	expressed protein
Os.34174.1.S1_at	expressed protein
Os.54555.1.S1_at	fasciclin domain containing protein, expressed
Os.24865.1.A1_at	HAD superfamily phosphatase, putative, expressed
Os.10576.1.S1_at	harpin-induced protein 1 domain containing protein, expressed
Os.27581.1.A1_at	IQ calmodulin-binding motif family protein, putative, expressed
Os.6863.1.S1_at	Jacalin-like lectin domain containing protein, putative, expressed
OsAffx.12547.1.S1_a	lectin-like protein kinase, putative, expressed
Os.24786.1.S1_s_at	nitrilase-associated protein, putative, expressed
OsAffx.26533.1.S1_a	nitrilase-associated protein, putative, expressed
OsAffx.13783.1.S1_a	O-methyltransferase, putative, expressed
Os.52261.1.S1_at	OsPOP9 - Putative Prolyl Oligopeptidase homologue, expressed
Os.47625.1.A1_s_at	peroxidase precursor, putative, expressed
Os.25687.1.S1_x_at	phenylalanine ammonia-lyase, putative, expressed
Os.25687.1.S1_at	phenylalanine ammonia-lyase, putative, expressed
Os.6654.1.S1_at	retrotransposon protein, putative, unclassified, expressed
Os.32366.1.S1_at	STE_MEKK_ste11_MAP3K.7 - STE kinases include homologs to sterile 7, sterile 11 and sterile
Os.26820.1.A1_at	terpene synthase, putative, expressed
Os.35365.1.S1_at	transporter-related, putative, expressed
Os.50015.1.S1_at	WRKY28, expressed
Os.10416.1.S1_at	N/A

e 20 from yeast, expressed
