# Supplementary material for: Physiological, anatomical and transcriptional alterations in a rice mutant leading to enhanced water stress tolerance
Source: AoB Plants. 2015 Mar 27;7:plv023. doi: 10.1093/aobpla/plv023 (PMC4482838; doi:10.1093/aobpla/plv023)
Supplement: Additional Information [file supp_plv023_plv023supp_table4.pdf]

Cis element/Motif Name	Frequency of overrepresentation
MYBCORE	95%
POLASIG1	88%
MYB1AT	87%
PBF	85%
CGACGOSAMY3	74%
PYRIMIDINEBOXOSRAMY1A	73%
SITEIIATCYTC	72%
MYCATERD1	70%
MYCATRD22	70%
POLASIG2	65%
AACAmotifI	63%
CAATmotifI	56%
DRECRTCOREAT	54%
CAREOSREP1	52%
AuxRE	51%
TATABOXOSPAL	49%
MYB2AT	48%
MYBGAHV	43%
AACACOREOSGLUB1	40%
ABREmotif	39%
TATCCAYMOTIFOSRAMY3D	38%
G-box-like	35%
ACGTABREMOTIFA2OSEM	34%
ACGTABOX	29%
CATC-relatedmotif	28%
ABREOSRAB21	26%
TATAboxII	25%
PROLAMINBOXOSGLUB1	23%
ACGTOSGLUB1	21%
WUSATAg	21%
P-box	20%
tGCN4	20%
TATAboxIV	18%
AGCBOXNPGLB	18%
TATC-box	16%
TATAboxIII	15%
GCrichrepeatII	15%
RYREPEATVFLEB4	14%
GARE1OSREP1	14%
LEAFYATAG	14%
GCN4OSGLUB1	12%
BP5OSWX	11%
GARE2OSREP1	8%
DOF3	8%
ABREZMRAB28	7%
GCAAmotif	5%

SITEIOSPCNA	5%
HSE-steptype	4%
GCrichrepeatIV	4%
motifB	4%
motifI	3%
motifA	3%
unfolding-protein-like	3%
C-box	3%
motifIIb	2%
HSE-perfect-type	2%
SITEIIBOSPCNA	2%
HSE-variantXL	2%
HSE-gaptype	2%
GCrichrepeatIII	2%
TATABOX1	2%
E2F1OSPCNA	1%
SITEIIAOSPCNA	1%
Oshox1	1%
ABADESI1	1%
